# Origin and evolution of the Notch signalling pathway: an overview from eukaryotic genomes

**DOI:** 10.1186/1471-2148-9-249

**Published:** 2009-10-13

**Authors:** Eve Gazave, Pascal Lapébie, Gemma S Richards, Frédéric Brunet, Alexander V Ereskovsky, Bernard M Degnan, Carole Borchiellini, Michel Vervoort, Emmanuelle Renard

**Affiliations:** 1 Aix-Marseille Universités, Centre d'Océanologie de Marseille, Station marine d'Endoume - CNRS UMR 6540-DIMAR, rue de la Batterie des Lions, 13007 Marseille, France; 2 School of Biological Sciences, University of Queensland, Brisbane, QLD 4072, Australia; 3 Institut de Génomique Fonctionnelle de Lyon, Université de Lyon, CNRS UMR 5242, INRA, IFR128 BioSciences Lyon-Gerland, Ecole Normale Supérieure de Lyon, 46, Allée d'Italie, 69007 Lyon, France; 4 Department of Embryology, Faculty of Biology and Soils, Saint-Petersburg State University, Universitetskaja nab. 7/9, St Petersburg, Russia; 5 Institut Jacques Monod, UMR 7592 CNRS/Université Paris Diderot - Paris 7, 15 rue Hélène Brion, 75205 Paris Cedex 13, France; 6 UFR de Biologie et Sciences de la Nature, Université Paris 7 - Denis Diderot, 2 place Jussieu, 75251 Paris Cedex 05, France

## Abstract

**Background:**

Of the 20 or so signal transduction pathways that orchestrate cell-cell interactions in metazoans, seven are involved during development. One of these is the Notch signalling pathway which regulates cellular identity, proliferation, differentiation and apoptosis *via *the developmental processes of lateral inhibition and boundary induction. In light of this essential role played in metazoan development, we surveyed a wide range of eukaryotic genomes to determine the origin and evolution of the components and auxiliary factors that compose and modulate this pathway.

**Results:**

We searched for 22 components of the Notch pathway in 35 different species that represent 8 major clades of eukaryotes, performed phylogenetic analyses and compared the domain compositions of the two fundamental molecules: the receptor Notch and its ligands Delta/Jagged. We confirm that a Notch pathway, with true receptors and ligands is specific to the Metazoa. This study also sheds light on the deep ancestry of a number of genes involved in this pathway, while other members are revealed to have a more recent origin. The origin of several components can be accounted for by the shuffling of pre-existing protein domains, or *via *lateral gene transfer. In addition, certain domains have appeared *de novo *more recently, and can be considered metazoan synapomorphies.

**Conclusion:**

The Notch signalling pathway emerged in Metazoa *via *a diversity of molecular mechanisms, incorporating both novel and ancient protein domains during eukaryote evolution. Thus, a functional Notch signalling pathway was probably present in Urmetazoa.

## Background

The emergence of multicellularity, considered to be one of the major evolutionary events concerning life on Earth, occurred several times independently during the evolution of Eukaryota in the Proterozoic geological period [1]. Multicellular organisms are not only a superimposition of the fundamental unit of life, namely the cell; the emergence of multicellularity further implies that cells must communicate, coordinate and organise. In Embryophyta and Metazoa, higher levels of differentiation and organization of cells resulted in the emergence of organs and their organisation into complex body plans. Reaching this critical step required the elaboration of sophisticated intercellular communication mechanisms [2,3]. Cell-cell interactions through signal transduction pathways are therefore crucial for the development and the evolution of multicellular organisms. The modifications of these signal transduction pathways explain the macroevolution process observed. In metazoans, fewer than 20 different signal transduction pathways are required to generate the observed high diversity of cell types, patterns and tissues [4]. Among them, only seven control most of the cell communications that occur during animal development: Wnt; Transforming Growth Factor β (TGF-β); Hedgehog; Receptor Tyrosine Kinase (RTK); Jak/STAT; nuclear hormone receptor; and Notch [5,6]. These pathways are used throughout development in many and various metazoans to establish polarity and body axes, coordinate pattern formation and choreograph morphogenesis [4]. The common outcome to all of these pathways is that they act, at least in part, through the regulation of the transcription of specific target genes by signal-dependent transcription factors [6].

The Notch signalling pathway is a major direct paracrine signalling system and is involved in the control of cell identity, proliferation, differentiation and apoptosis in various animals (reviewed in [7-12]). Notch signalling is used iteratively in many developmental events and its diverse functions can be categorized into two main modalities "lateral inhibition" and "boundaries/inductive mechanisms" [8,13]. During lateral inhibition, Notch signalling has mainly a permissive function and contributes to binary cell fate choices in populations of developmentally equivalent cells, by inhibiting one of the fates in some cells and therefore allowing them to later adopt an alternative one. Lateral inhibition is a key patterning process that often results in the regular spacing of different cell types within a field. The Notch pathway may also have more instructive roles, whereby signalling between neighbouring populations of different cells and induces the adoption of a third cell fate at their border, establishing a developmental boundary [14,15].

**Figure 1:**
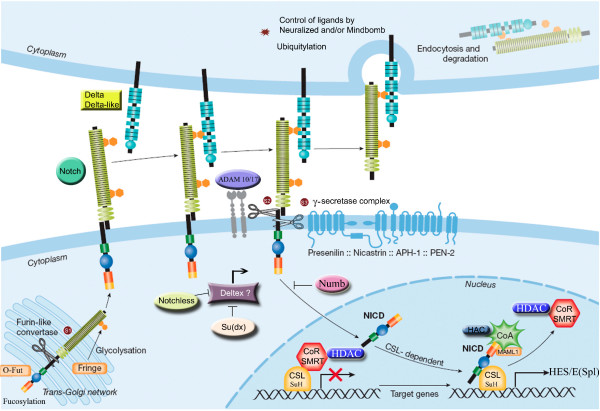
**Major components and auxiliary factors of the Notch signalling pathway as described in Bilateria (modified from **[16]**)**. Most of the mentioned components are studied hereafter. S1 to S3 represent the cleavage sites. See Table 1 for complete names and functions of the components.

A large number of studies, mainly conducted on *Drosophila, Caenorhabditis *and vertebrates, have characterized the molecular properties and functions of the main components and auxiliary factors of the Notch pathway. These are strongly conserved in bilaterians (Figure 1 modified from [16]). Both the Notch receptor and its ligands (Delta or Jagged/Serrate also known as DSL proteins) are type I transmembrane proteins with a modular architecture. In eumetazoans, the Notch protein is classically considered to be composed of an extracellular domain (NECD) that comprises several EGF and LNR motifs, an intracellular domain (NICD) that includes ANK domains and a PEST region [7,8,17,18]. The Notch protein is synthesized as an inactive precursor that has to be cleaved three times and to undergo various post-translational modifications to become active [19-22]. In the Golgi apparatus, the first cleavage (S1) is done by the Furin protease resulting in two fragments (NICD and NECD) that subsequently undergo *O*-fucosylation (by *O*-Fucosyltransferase) and glycosylation (by Fringe). Upon ligand binding, the second cleavage (S2) occurs by the metalloproteases ADAM 10 and 17 [19-21]. The final cleavage (S3) is performed by the γ-secretase complex (Presenilin-Nicastrin-APH1-PEN2). These cleavages result in the release, upon ligand binding, of NICD into the cytoplasm and its subsequent translocation to the nucleus. There, NICD interacts with the CSL (CBF1, Su(H), Lag-1)/Ncor/SMRT/Histone Deacetylase (HDAC) transcriptional complex and recruits the coactivator Mastermind and a Histone Acetylase (HAC), thus activating the transcription of target genes in particular the HES/E(Spl) genes (Hairy/Enhancer of Split) [9].

**Table 1:** Proteins implicated in the Notch pathway and their known functions

**Component type/role**	**Component name and abbreviation**
Receptor	Notch

Ligands	Delta/Jagged

Fucosyltransferase	*O*-fucosyltransferase (*O-*fut)

Glycosyltransferase	Fringe

Cleavage S1	Furin

Cleavage S2	ADAM 17 = TACE

Metalloproteases	ADAM 10 = Kuzbanian

Cleavage S3	Presenilin (Pres)
	Nicastrin
γ-secretase complex	Anterior Pharynx defective 1 (APH1)
	Presenilin Enhancer 2 (PEN2)

Transcriptional complex	CSL (CBF1, Su(H), Lag-1)
	Silencing Mediator of Retinoid and Thyroid receptors (SMRT)

Targets	Hairy Enhancer of Split (HES)

Ligand	Neuralized (Neur)
Regulation	Mindbomb (Mib)

Receptor	Deltex
Regulation	NEDD4/Suppressor of Deltex (Su(dx))
	Mastermind (MAM)
	Numb
	Notchless (Nle)
	Strawberry notch (Sno)

In addition to these core components of the Notch pathway, several other proteins are used to regulate Notch signalling in some cellular contexts, and act either on the receptor Notch or on the ligand DSL (Figure 1). Some of these regulators modulate the amount of receptor available for signalling [23]. Numb, the NEDD4/Su(dx) E3 ubiquitin ligases, and Notchless are important negative regulators, while Deltex is considered to antagonize NEDD4/Su(dx) and therefore to be an activator of Notch signalling [24,25]. Strawberry Notch (Sno), another modulator of the pathway whose role is still unclear, seems to be active downstream and disrupts the CSLrepression complex [26]. Regulation may also occur at the level of ligand activity via the E3 ubiquitin ligases Neuralized and Mindbomb [27,28].

**Figure 2:**
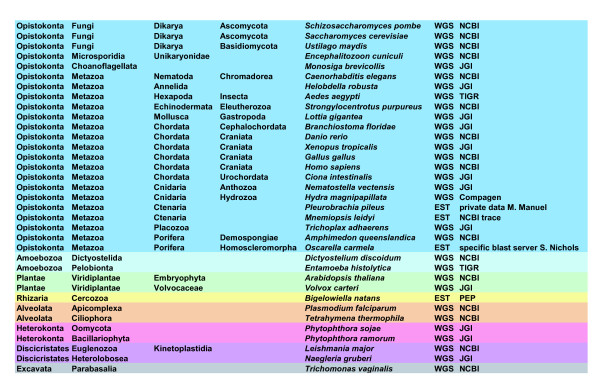
**List of the 35 species selected for the study, representing the 8 major clades of eukaryotes**. Colour code: Opistokonta (blue); Amoebozoa (light blue); Plantae (green); Rhizaria (yellow); Alveolata (orange); Heterokonta (pink); Discicristata (violet); Excavata (grey). Data sets and data sources are also indicated. WGS: whole genome available; EST: only EST available. *O. carmela*:  Compagen: ; PEP: . JGI: . NCBI: . TIGR: .

Most of what we know about the Notch signalling pathway comes from studies conducted on a few bilaterian species. Recently, studies have shown the existence of a Notch signalling pathway in non-bilaterian species, such as the cnidarian *Hydra *and the sponge *Amphimedon*, and its putative functions in the former species [29,30]. However, the ancestral structure, functionality and emergence of this complex multi-component signalling system are still open questions. Few studies have been initiated to understand how signalling pathways appeared and evolved beyond the Bilateria [4-6] but the recent sequencing of the first sponge genome, *Amphimedon queenslandica *has opened new perspectives for studying the origin and evolution of signalling pathways in the Metazoa [31-35]. With the goal of illuminating the early evolution of the Notch pathway, we have therefore undertaken a comparative genomic study of the components of this pathway across the Eukaryota. Our study encompasses 35 species (31 with fully sequenced genomes) covering the 8 major clades of eukaryotes [36] (Figure 2), and includes the 22 main components of the Notch pathway (Table 1). We have also paid special attention to the evolution of domain composition (within the Metazoa) of the multidomain proteins Notch, Delta, Mindbomb, Su(H) and Furin, to investigate whether domain shuffling has occurred during their evolution, as in other signalling pathways [31,37].

This wide genomic comparison reveals that most of the Notch components are present in all the metazoan species studied, including putative basal metazoans such as sponges and placozoan, suggesting that a functional Notch pathway was already present in the last common ancestor of present-day metazoans and was subsequently strongly conserved during metazoan evolution. While many of the Notch pathway components are also shared with non-metazoan eukaryote lineages, thus suggesting a more ancient origin, nine of the components are metazoan-specific, including the Notch receptor and the DSL ligands. This indicates that while the Notch pathway is a metazoan synapomorphy, it has been assembled through the co-option of pre-metazoan proteins, and their integration with novel metazoan-specific molecules acquired by various evolutionary mechanisms.

## Results

### Genome-wide identification of the main Notch signalling pathway components in eukaryotes

To understand more precisely the evolution of the Notch pathway at the scale of the eukaryotes, we systematically searched for all the main Notch pathway elements in completely sequenced genomes and Expressed Sequence Tag (EST) data of 35 different eukaryote species (Figure 2). Table 1 lists the 22 genes that we analysed and summarizes their functions in the Notch pathway (see also Figure 1). We included in this list both genes that encode core components of the Notch pathways (such as receptor, ligands, and molecules involved in receptor processing) and genes that encode modulators of the pathway not used in all cases of Notch signalling (such as *Numb *and *Notchless*) [23]. We selected 35 species representative of the major clades of eukaryotes [36]: 18 metazoans, 4 fungi (including one microsporidia), 1 choanoflagellate, 2 amoebozoans, 2 species of plants (one embryophyta and one volvocale), 2 alveolates, 2 heterokonts, 2 species of discicristates, 1 species of excavates and 1 rhizaria. Figure 2 provides the full list of the chosen species with their assumed phylogenetic position and internet links to the genomic databases.

**Figure 3:**
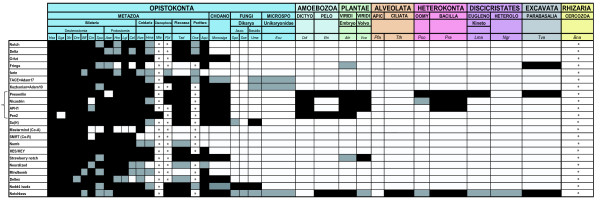
**Presence or absence of Notch signalling pathway components and auxiliary factors in eukaryotes**. Colour code: In black: genes present. In white: genes absent. In grey: not all diagnostic domains found. In white with an asterisk: incomplete data (EST) do not allow definitive conclusions. In curly bracket: the four members of the γ-secretase complex. Asco = Ascomycota; Basidio = Basidiomycota; CHOANO = Choanoflagellata; DICTYO = Dyctiostellida; PELO = Pelobionta; VIRIDI = Viridiplantae; Embryo = Embryophyta; Volvo = Volvolcaceae; APIC = Apicomplexa; OOMY = Oomycota; BACILLA = Bacillariophyta; EUGLENO = Euglenozoa; Kineto = Kinetoplastida; HETEROLO = heterolobozoa.

We performed BLAST searches [38] to assess the presence or absence of Notch pathway genes in the sampled species, as described in the methods section. In most cases, the Notch pathway elements are multidomain proteins and share some of their domains with other proteins. For each target protein, only the combined occurrence of all requisite domains was considered diagnostic for identification. We systematically defined a diagnostic domain organization for each target protein (Table 2) and identified genes as detailed in the methods section. We also constructed multiple alignments for each protein and performed phylogenetic analyses to confirm the orthology relationships (Additional files 1 and 2). Figure 3 summarizes the output of our analyses: genes were scored as "present" when all the domains were identified, "incomplete" when some domains were missing, or "absent" when blast searches gave no significant result. For EST libraries, as the absence and the incomplete status of genes cannot be definitive due to the partial nature of this type of data, we chose to indicate "present" only when all domains were retrieved. Detailed domain composition for each gene in each species is presented in the Additional file 3.

**Table 2:** Domains considered as diagnostic for each protein

**Proteins**	**Diagnostic domains**	**References**
Notch	LNR/EGF/ANK	[23]

Delta	DSL/EGF	[23]

Fringe	Fringe	[80]

Adam 10/17	ZNMc/Disin	[139]

Pres	Peptidase A22	[140]

Nicastrin	M20-dimer superfamily	[141]

APH1	APH1 superfamily	[63]

PEN2	-	[63]

CSL/Su(H)	lag1/IPT RBJ Kappa/beta trefoil	[142]

MAM	Maml - 1	[143]

Numb	numbF/PH-like	[144]

Sno	ABC-ATPase	[26]

Neur	Neur/Zinc finger Ring	[145]

Mib	Mib-herc2/ANK/ZF ring/ZZ Mind	[28]

Deltex	WWE/ZF ring	[25]

NEDD4/Su(dx)	C2/WW/HECT	[146]

Nle	NLE/WD40	[72]

Furin	subtilisin/proprotein convertase/furin	[130]

*O*-fut	-	[147]

SMRT	SANT	[148]

HES	HLH/Hairy orange	[149]

Our data confirm the strong evolutionary conservation of the Notch pathway in bilaterians as all components are present in almost all the analysed bilaterian species (Figure 3). There are two exceptions to this rule, *Fringe *which is absent from the genome of two protostomes, *Caenorhabditis *and *Helobdella*, and *Mastermind *is not found in 5 of the studied bilaterian species across both protostomes and deuterostomes. The latter case is puzzling given the documented importance of *Mastermind *in both vertebrates and *Drosophila *[39,40] and its presence in the non-bilaterian species *Nematostella *(Figure 3). This suggests that *Mastermind *has been repeatedly lost in various bilaterian species. Alternatively, as the sequence similarity between the *Mastermind *genes in *Drosophila *and vertebrates is quite weak [41], these genes may be difficult to track by sequence similarity searches. Our data also indicate that the overall Notch pathway conservation extends to non-bilaterian species. Indeed, most pathway components can be identified in the cnidarians *Nematostella *and *Hydra*, in the placozoan *Trichoplax *and the sponge *Amphimedon *(Figure 3). We can therefore conclude that most Notch pathway components were already present in the last common ancestor of all metazoans, the Urmetazoa.

Four genes were not found complete outside bilaterians, *SMRT, Furin, Numb *and *Neuralized*, suggesting that these genes are specific to bilaterians (Figure 3). Genes encoding proteins with a SANT domain (which is found in bilaterian SMRT) are found in non-bilaterians, but the sequence similarity is too weak to establish that some are *bona fide SMRT *genes. The case of the Furin protein is puzzling. This protein pertains to the proprotein convertase subtilisin/kexin family (PCSK) [42]. In the demosponge *Amphimedon *and the choanoflagellate, there are some proteins of the PCSK family and some of them possess the diagnostic domains of the Furin proteins (data not shown). Nevertheless, the phylogenetic analysis revealed that these proteins do not group with bilaterian Furins, however the latter are paraphyletic in the phylogenetic tree (Additional file 2). In the case of Neuralized, while Neuz domains are found in non-bilaterians, they are only found in association with a RING binding domain in the Bilateria. In the same way, Ph-like domains of Numb are only found in association with a NumbF domain in bilaterians. The gene *Mastermind *is only found in eumetazoans and two others, *Fringe *and *Mindbomb*, are found in *Amphimedon *but not in *Trichoplax *(Figure 3).

The absence of some components in some non-bilaterian species may represent a progressive elaboration of the pathway during early metazoan evolution, or else may correspond to secondary losses in some lineages. However, these data can be difficult to interpret in terms of the evolution of the Notch pathway, as the phylogenetic relationships of the aforementioned non-bilaterian species are still controversial [43-45]. Nevertheless, we decided to base our discussion on the metazoan relationships hypothesised in the most recent phylogenomic study [46] as we believe it to be the most robust and complete analysis to date.

Our data so far indicate that most of the Notch pathway components were already present in Urmetazoa. Interestingly, among the 22 targeted genes, only nine are specific to metazoans (*Notch, Delta, Furin, Mastermind, Numb, Neuralized, Mindbomb, HES *and *SMRT*). Strikingly, among these, nine are the genes encoding the ligand and the receptor, suggesting that the canonical Notch pathway only exists in metazoans. Indeed, in the genome of the choanoflagellate *Monosiga*, no Notch gene has been found, only cassettes of some protein domains encoded on separate genes have been reported [47]. Of note, we also found another gene in this species that possesses the domain arrangement of a *Notch *gene (1 signal peptide, 1 EGF domain, 2 LNR domains, a transmembrane domain and 3 ANK domains, Additional file 4). While this gene contains the minimum set of diagnostic Notch domains, it has very weak sequence similarity to *Notch *genes, and in the absence of further evidence we choose here to name it "*Notch*-like". Nevertheless, we can not exclude that a "protoNotch" receptor might have been already present in Holozoa.

**Figure 4:**
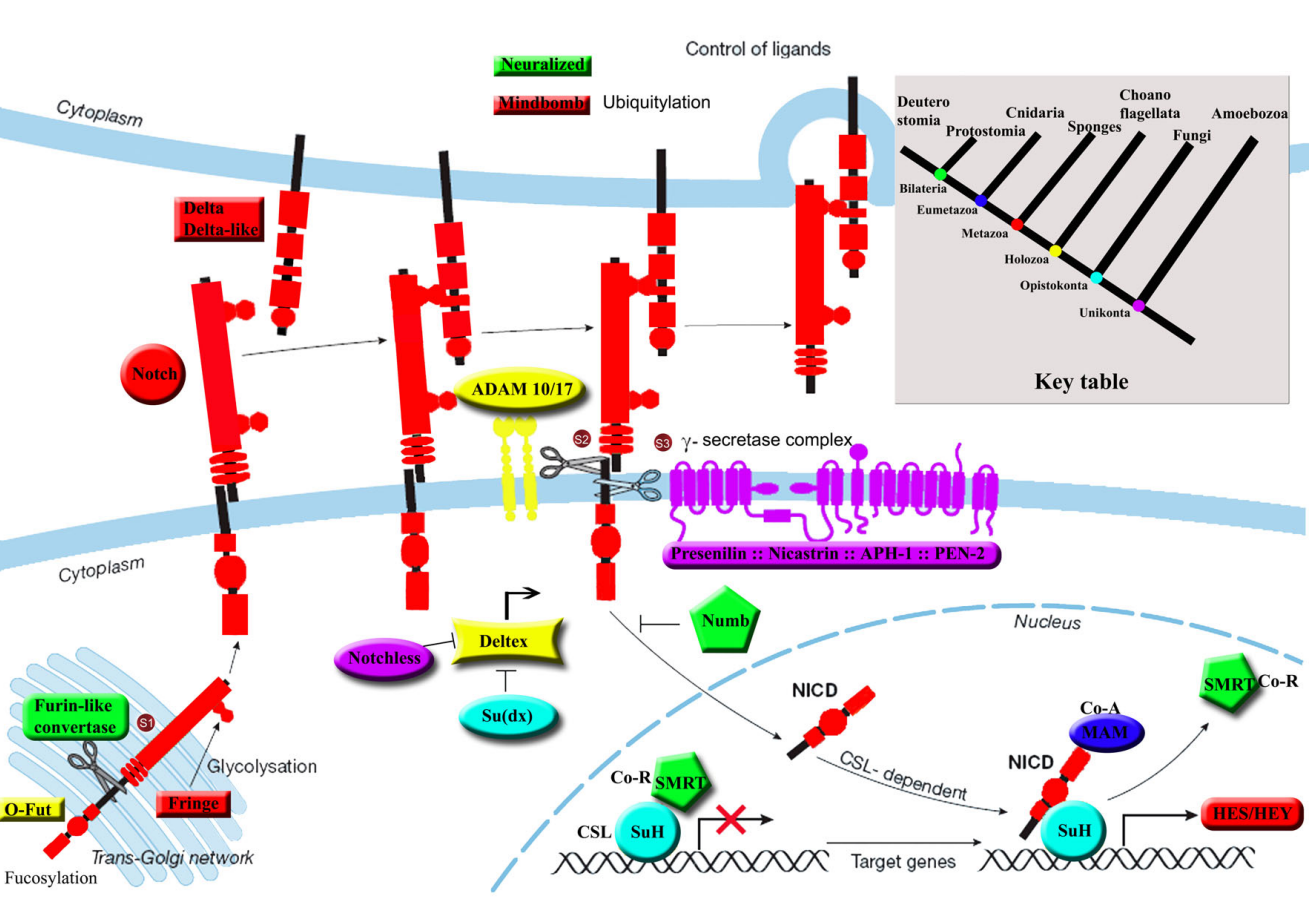
**Origin of Notch signalling pathway components in Unikonta (modified from **[16]**)**. Each colour represents the origin (see figure inset for the colour code) of the gene inferred from our study on the basis of the phylogenetic hypothesis proposed in [45,46].

13 components are found in various other eukaryote taxa; some are likely to have appeared during early eukaryote evolution and may even have been present in the last common ancestor of present-day eukaryotes (LECA). Others seem to have specifically appeared in the opisthokont lineage. Figure 4 represents the possible scenarios of emergence and loss of the various Notch components, inferred from the most recent and robust phylogenetic hypotheses [45,46] and on the basis of the parsimony principle. Four genes of this pathway seem to have appeared early in evolution and are inferred to have been present in the LECA. These include *Notchless (Nle) *and three of the four genes coding for proteins implicated in the so-called "γ-secretase complex". Indeed, the *Presenilin *gene is shared by all eukaryotes (except Fungi + microsporidies and alveolates) whereas *Nicastrin *and *APH1 *are found in the Holozoa, the Amoebozoa, the Plantae and the Heterokonta. Interestingly, none of these genes are found in species of Fungi + Microsporidia and Alveolata, suggesting a secondary loss. For other genes (3) that are shared by fewer taxa, it is difficult to state whether they were present in the LECA and lost various times, or acquired independently: this is the case for *PEN2*, *Strawberry Notch *(*Sno*), the role of which in the Notch pathway is not yet clear, and *Fringe *(a *Fringe*-like gene is present in plants and Excavata). All other genes originated more recently, in the last common ancestor of opisthokonts (*Suppressor of Hairless *(*Su(H)*)[48]; *Suppressor of Deltex (NEDD4*/*Su(dx)*). This might also be the case for the two *Adam *genes. We cannot confidently assign the *Adam *genes we found in Fungi and Microsporidia to a particular Adam group; however these genes are most closely related to metazoan Adam 10 and 17 families (see Additional file 2) as already reported by another study on *Aspergillus fumigatus *[49]. Other genes are specific to holozoans (*O-Fut*; *Deltex*).

For the ctenophore species (*Mnemiopsis *and *Pleurobrachia*) as well as the homoscleromorph sponge *Oscarella*, only a few target genes were identified in the available non exhaustive EST databases and we were unable to conclude whether or not the remaining genes are present in those taxa.

### Focus on *Notch *and *DSL *proteins evolution in metazoan: phylogenetic analyses and domain composition arrangement

We chose to focus our further analyses on the two main molecules of the pathway, Notch and DSL, and study their evolution in metazoans. We first performed phylogenetic analyses using both Maximum Likelihood (ML) and Bayesian Inference (BI) approaches and then investigated the domain organizations of each. Topologies obtained in our phylogenetic analyses are not fully resolved, as previously noticed for the Notch ligands [50-52]. Long branch attraction bias (LBA) may be suspected in some cases and, as previously reported by different authors [53,54], ML appears more sensitive to LBA than BI. Concerning domain composition and organization, generally all the diagnostic domains in *Delta *or *Notch *genes are present in bilaterian sequences, but some domains seem to be lacking in a few species. In these cases, we can not state whether this is due to prediction errors, sequencing gaps in the available genome sequences or secondary losses. When the available software prediction is equivocal, important conserved residues can often be identified in the regions where domains would be expected, suggesting functional conservation. Despite these technical limits, our analysis presents several features of interest.

**Figure 5:**
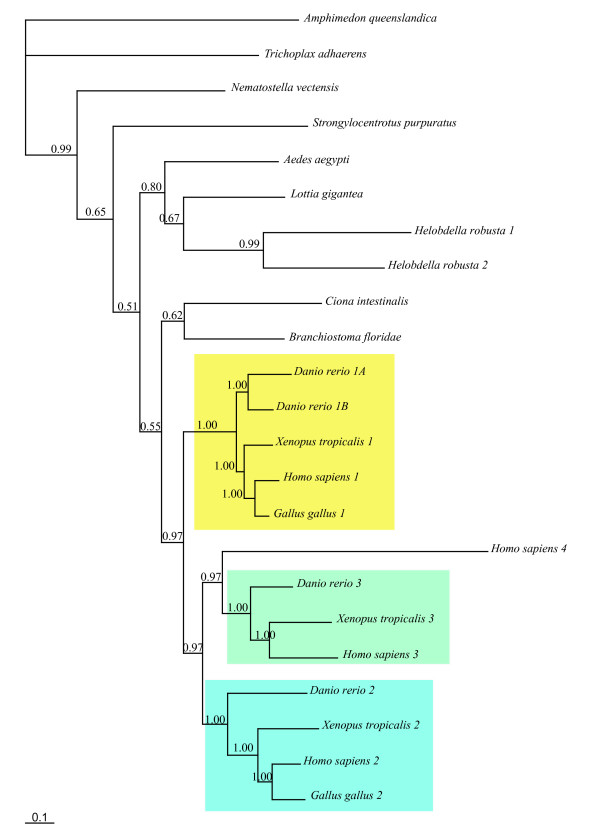
**Bayesian phylogram of Notch representative proteins**. Posterior probabilities (greater than 0.50) are indicated next to the node. The Notch families 1, 2 and 3 are presented respectively in yellow, blue and green boxes.

**Figure 6:**
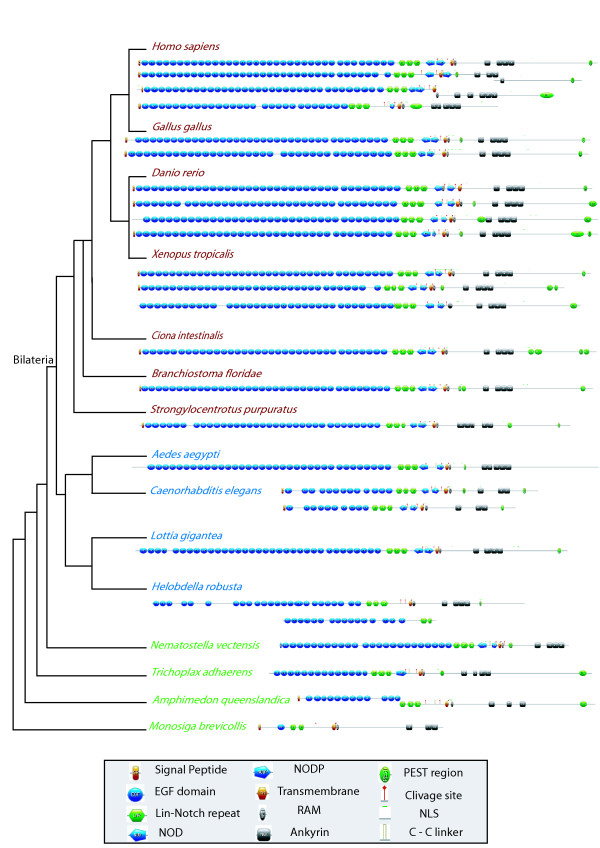
**Domain arrangement of Notch proteins in metazoans**. Deuterostomes, protostomes and non-bilaterian metazoans are presented in red, blue and green respectively. Phylogenetic hypothesis based on [45,46]. See figure inset for the domain legends.

First, a single *Notch *gene is found in most species (Figure 5, Additional file 5), except in vertebrates (from 2 to 4 genes) and in the annelid *Helobdella *(2 genes). In the former case, this is most probably due to the well documented occurrence of two whole genome duplication events in this lineage [55]. In the latter case, it may correspond to a recent duplication, limited to an annelid lineage. Indeed, the two *Helobdella *genes form a monophyletic group in our trees (Figure 5, Additional file 5) and only a single *Notch *gene has been found in the genome of another annelid species, *Capitella *sp. I [56]. In the Bayesian analysis, we note that the sequences from bilaterians form a monophyletic group and the cnidarian sequence clearly appears more related to bilaterian sequences than to other non-bilaterian sequences (PP: 0.99). The receptor Notch is considered to be made of several domains: EGF repeats (Epidermal Growth factor: about 30 to 40 amino acids, containing six conserved cysteines), three LNR (lin-notch repeat) or Notch domains, two enigmatic domains NOD and NODP (their roles are unknown), a RAM 23 domain, a PEST domain and several Ankyrin repeats (Figure 6). The number of EGFs is variable and spans from 10 in *Caenorhabditis *to 36 in humans. The NOD and the NODP are absent in sponges and in some bilaterian species. The absence of a signal peptide is observed in 8 of the 25 sequences, and the PEST domain is absent at the C-terminus of three sequences (Figure 6).

**Figure 7:**
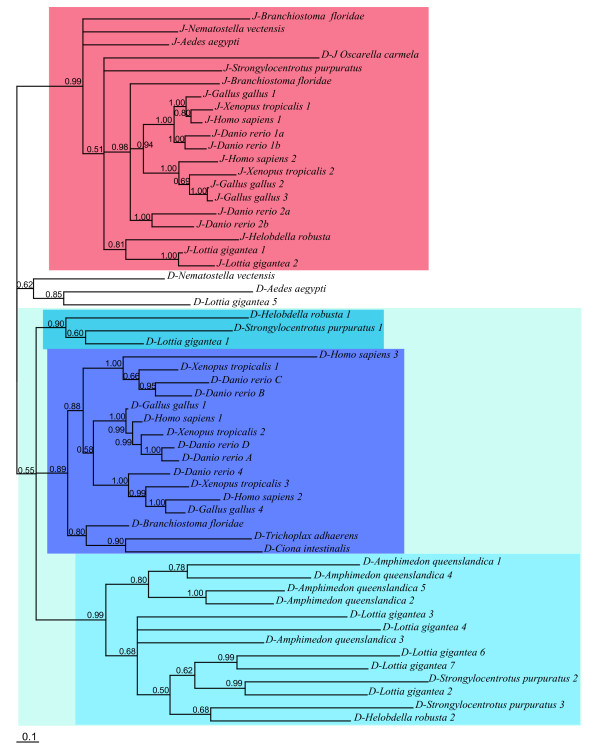
**Bayesian phylogram of DSL representative proteins**. Posterior probabilities (greater than 0.50) are indicated next to the node. In blue boxes, most of the Delta sequences split in three clades. In red box, the Jagged representatives.

Second, in the DSL family (Figure 7, Additional file 6) *Jagged *is absent from placozoans and sponges and present (1 to 4 copies) in all other studied metazoans. *Delta *is present in all metazoan species of our study, and in contrast to *Jagged*, is also found in non-eumetazoans. The number of copies of *Delta *is also variable but is notably high (7 copies) in the gastropod snail *Lottia*. We also note that 5 copies are present in the sponge genome, which is remarkably high in comparison to the other non-bilaterians such as *Trichoplax *and *Nematostella*. In spite of the differences between the ML and BI topologies and the weak statistical support of deep nodes in both analyses, we are able to draw some conclusions about the evolution of the DSL family. In the Bayesian tree (with better resolution), a strongly supported clade (PP: 0.99) contains all eumetazoan *Jagged *sequences plus one sequence from *Branchiostoma *and one from the sponge *Oscarella *(not supported by the ML tree; Additional file 6). This suggests the existence of a subfamily of Serrate/Jagged proteins that is found in all the major animal lineages and therefore is of ancient origin. Most of the other studied proteins (32 out of 35 remaining sequences) form another large monophyletic group (although of weak support, PP: 0.55) likely corresponding to what may be called a Delta clade (Figure 7) since it includes the known Deltas from vertebrates as well as from the main metazoan lineages, including the sponge *Amphimedon *and the placozoan *Trichoplax*. Nevertheless, the internal branching of the Delta sequences makes no sense in the light of species phylogenies. Our phylogenetic analysis rather suggests that the last common ancestor of all eumetazoans already possessed at least one *Delta *and one *Jagged/Serrate *gene and that the Urmetazoa possessed at least one sequence of *Delta*. The position of the of *D/J-Oscarella carmela *in the Bayesian tree is puzzling, the BLAST analysis revealed a higher similarity between this sponge (unfortunately incomplete) sequence and Delta sequences but we cannot definitively rule out the possibility that it might be an incomplete Jagged protein.

**Figure 8:**
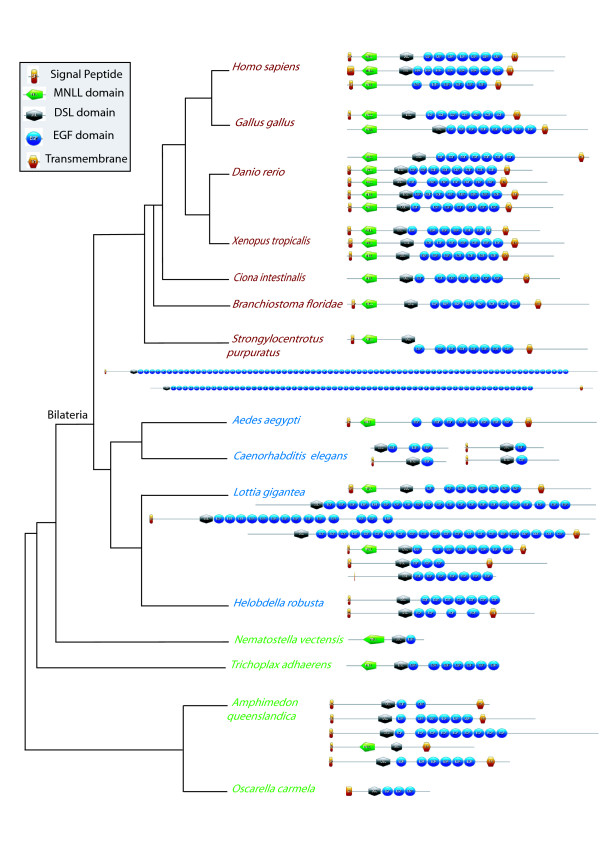
**Domain arrangement of Delta proteins in metazoans**. Deuterostomes, protostomes and non-bilaterian metazoans are presented in red, blue and green respectively. Phylogenetic hypothesis based on [45,46]. See figure inset for the domain legends.

**Figure 9:**
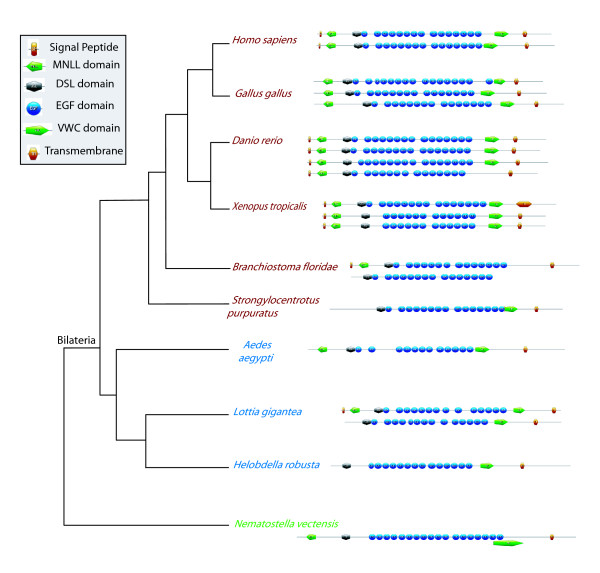
**Domain arrangement of Jagged proteins in metazoans**. Deuterostomes, protostomes and non-bilaterian metazoans are presented in red, blue and green respectively. Phylogenetic hypothesis based on [45,46]. See figure inset for the domain legends.

To support our phylogenetic analyses, we also systematically investigated the domain arrangements of the DSL family proteins (Figures 8, 9). DSL proteins are usually considered to be composed of several domains, namely a signal peptide (secretion signal), a MNLL domain (a conserved region at the N terminus), a DSL domain (Delta-Serrate-Lag-2: about 50 amino acids, containing six conserved cysteines and a YYG motif), a variable number of EGF repeats and a transmembrane region. In addition to these domains, the Serrate/Jagged proteins also contain a supplementary domain, the Von Willebrand factor C domain (VWC) [57]. Interestingly, all but two proteins included in the Jagged group in our phylogenetic trees contain the VWC domain, confirming that they are *bona fide *Jagged proteins (Figure 9). The exceptions are the two *Branchiostoma *genes; phylogenetic analyses highly support their belonging to the Jagged clade, suggesting that the absence of the VWC domain may be due to incorrect protein predictions, incomplete genome assemblage or secondary loss.

Third, in our ligand domain analyses (Figures 8, 9), we found that the MNLL, the signal peptide and the transmembrane domains are not present (or detected) across all metazoan Delta ligands but we could find them in the majority of Jaggeds. Most deuterostome Delta sequences possess the MNLL domain, in contrast to the protostome sequences. Among the sponge species a MNLL is only predicted in one copy of the *Amphimedon *Deltas. The number of EGF repeats ranges from 0 (in the sponge *Amphimedon*) to 75 in Delta and from 12 to 18 in Jagged. An average of 7-8 EGF motifs are present in deuterostome Delta sequences; it is much more variable in protostomes. We note that motifs expected at the N or C terminus are more often lacking. As this absence of a signal peptide or a transmembrane region appears incongruous and hardly compatible with the conservation of functionality, the assembly and annotation of the concerned genomes may require a re-examination [58].

Surprisingly, in cnidarian genomes, in addition to the Delta and Jagged sequences, we found genes composed only of DSL domains (from 1 to 11 repeats) and one gene composed of a MNLL domain associated to 3 DSL domains (Additional file 7). It remains to be seen either these are true genes with unique functionalities, or represent misassembled regions of the genome.

### Origin and evolution of protein domains involved in the pathway

**Figure 10:**
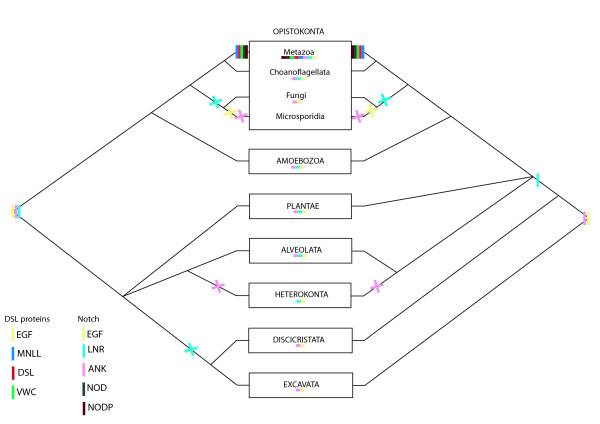
**Scenarios proposed for the emergence of the constitutive domains of DSL ligands and Notch receptors during eukaryote evolution**. These scenarios are inferred from our analyses on the basis of the phylogenetic hypotheses of Baldauf 2003 [36] and the application of the principle of parsimony. The left and the right halves of the figure represent the two rooting hypotheses for the eukaryotes. A line represents the appearance of a domain, a cross represents the loss of a domain. Each colour corresponds to a specific domain (see figure inset). Domain presences are summarized under each taxa.

**Figure 11:**
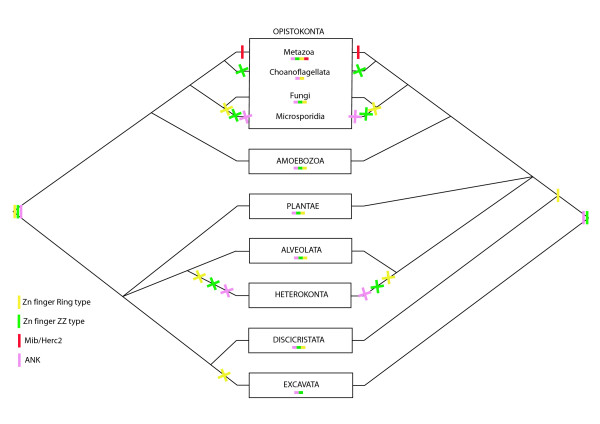
**Scenarios proposed for the emergence of the constitutive domains of the receptor regulator Mindbomb during eukaryote evolution**. These scenarios are inferred from our analyses on the basis of the phylogenetic hypotheses of Baldauf 2003 [36] and the application of the principle of parsimony. The left and the right halves of the figure represent the two rooting hypotheses for the eukaryotes. A line represents the appearance of a domain, a cross represents the loss of a domain. Each colour corresponds to a specific domain (see figure inset). Domain presences are summarized under each taxa.

**Figure 12:**
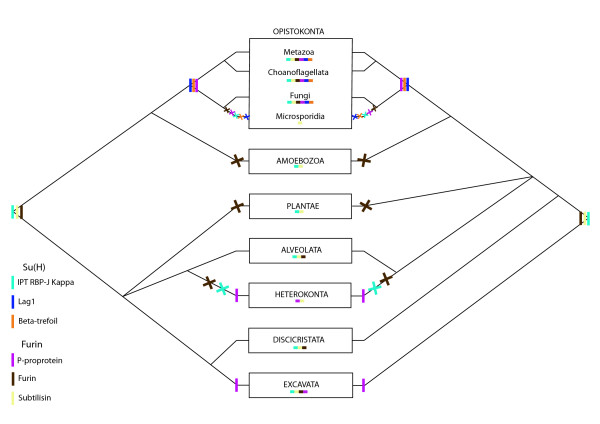
**Scenarios proposed for the emergence of the constitutive domains of Su(H) and Furin during eukaryote evolution**. These scenarios are inferred from our analyses on the basis of the phylogenetic hypotheses of Baldauf 2003 [36] and the application of the principle of parsimony. The left and the right halves of the figure represent the two rooting hypotheses for the eukaryotes. A line represents the appearance of a domain, a cross represents the loss of a domain. Each colour corresponds to a specific domain (see figure inset). Domain presences are summarized under each taxa.

We focused on five genes that encode multidomain proteins in the pathway: DSL, Notch, Mindbomb, Su(H), Furin. We mapped the possible acquisition(s) and loss events of the different domains during eukaryote evolution according to the phylogenetic hypothesis of Baldauf (2003) [36] (Figures 10, 11, 12).

On one hand, it appears that various domains have an ancient origin; they are shared by several eukaryote lineages, so we can hypothesize their presence in the LECA (or in the ancestor of eukaryotes bearing mitochondria: all eukaryotes except discicristates and excavates). This is the case for: EGF repeats of Notch and DSL (only present in eukaryotes [59]), ANK repeats of Mindbomb and Notch (present in eukaryotes, Archaea and Bacteria); the LNR domain of Notch; both ZZ and Ring type ZN finger domains of Mindbomb; the IPT RBP-JKappa domain of Su(H); the Subtilisin domain and the Furin domain. In all of these cases, a hypothesis of ancestrality followed by one or more secondary losses is most parsimonious.

On the other hand, several domains appear to have originated more recently since they are specific to opisthokonts or even to metazoans: the MNLL, DSL and VWC domains involved in *DSL *composition; the NOD and NODP domains of Notch; the Mib/Herc2 domain of Mindbomb; the Lag1 and Beta-trefoil domains of Su(H). Thus, a total of six domains may represent synapomorphies of the Metazoa. In the case of the P-proprotein of Furin, the more parsimonious inference is that it may have appeared convergently three times in Excavata, Heterokonta and in Opistokonta (with a secondary loss in Microsporidia).

## Discussion

A functional Notch pathway seems to have been present in the Urmetazoa and comprised at least 17 components [30]. The later addition of five other components (in Eumetazoa or Bilateria) can thus be considered as facultative and responsible for additional regulation properties of the pathway. Our study indicates that the presence of the Notch pathway is a synapomorphy of metazoans as this is the only kingdom to possess all the key components of the pathway, most importantly the receptor and ligands. Our analysis also sheds light on the molecular mechanisms that may have been invoked in the formation of this pathway. Indeed, as we discuss hereafter, our study shows that Notch signalling has originated by cooption of pan-eukaryotic ancestral genes; modification of ancestral functions by new protein-protein interactions (mediated by novel metazoan domains); lateral gene transfer; formation of new proteins by both exon shuffling and duplications + divergence.

### Cooption of pre-existing genes and ancestral functions

This study, at the scale of the Eukaryota super-kingdom, reveals the presence of Notch components in diverse eukaryotic organisms, and thus their ancient origin. Certain highly conserved genes, despite their ancestrality, seem to be absent in Fungi and Microsporidia. This is consistent with previous genomic analyses that have documented massive gene losses in the LCA of Fungi + Microsporidia, and a further round of losses in microsporidies in relation to their parasitic life style [60,61].

#### The origin of Presenilin and of the γ-secretase complex

One of the most striking features uncovered by our study is the evolutionary conservation of the γ-secretase complex [22,62]: the four proteins composing this large transmembrane complex (Nicastrin, APH1, PEN2 and Presenilin [63,64]) are present in both plants and unikonts (except in Fungi + Microsporidia). While the entire γ-secretase complex does not seem to be pan-eukaryotic, our analysis nonetheless supports an altered evolutionary scenario than that formerly proposed for its main player, Presenilin. Previously, authors have hypothesized (based on an early view of the tree of life) a convergent acquisition of this gene in the metazoan and the plant lineages [65]. Our study reveals instead that Presenilin was present in the LECA, and then lost independently twice (in the LCA of Fungi + Microsporidia and in Alveolata). The APH1 and Nicastrin proteins may also be ancestral to Eukaryota, but our data is inconclusive for PEN2 on this point (found only in Unikonta and Plantae). Until now, functional analyses of this complex are available only in Embryophyta [66] and Metazoa, where it is known to be involved in the cleavage of Notch and other proteins such as ErbB4 [67] and APP (amyloid precursor protein, implicated in Alzheimers disease [68]). But the lack of evidence for a complete γ-secretase complex in the LECA (because of the possible later emergence of *PEN2*) parallels recent functional data indicating that in both mammals [69] and a bryophyte (*Physcomitrella patens *[66]), Presenilin is also involved in various γ-secretase-independent functions such as protein degradation and trafficking. The association of PEN2 (present either in the LCA of unikonts and plants or acquired independently in these two lineages) is considered to be necessary to acquire the proteolytic activity of Presenilin *via *conformational changes [70]. These changes may result in the accessibility of the two catalytic motifs Y/WD and GXGD, which are conserved at the eukaryotic scale [71]. This suggests that proteolysis might not have been the ancestral function of Presenilin (alone or in association with Nicastrin [66]), but might have been acquired secondarily by its co-option into the four protein γ-secretase complex (including PEN2). This challenging evolutionary scenario requires further investigations to be tested.

#### The origin of the Notchless inhibitor

*Notchless *encodes a protein containing a NLE domain and WD40-repeats [72]. In Eumetazoa, this member of the WD-repeat (WDR) protein superfamily [73,74] modulates the Notch pathway by binding the NICD [75] but also by interacting with *Deltex *and *Su(H*) [72]. Our analysis shows that Notchless was probably present in LECA. Nevertheless, in some of the studied species, the NLE domain is missing, and we cannot define whether this is due to secondary loss or to a high level of sequence divergence obscuring domain prediction. The high conservation of NLE sequences seems to be compatible with functional conservation as shown by transgenic experiments between a plant, *Solanum chacoense *and an animal, *Drosophila *[76,77]. However, while both plant and yeast Notchless proteins share an involvement in ribosome biogenesis, until now no such role has been reported in animals [78]. These observations have led authors to propose that either Notchless was primarily involved in ribosome biogenesis in eukaryotes and was secondarily recruited in the metazoans for a new function (regulator of Notch pathway), or that this role may still exist in animals despite the lack of experimental evidence [79].

#### Ancestrality or lateral gene transfer?

Two other members of the Notch pathway show an ambiguous history, in which the eventuality of lateral gene transfer (LGT) cannot be excluded. This is the case for both *Fringe *[80] and *Strawberry Notch (Sno)*. Our analyses reveal that *Fringe *is present in Metazoa, but also in plants and parabasalia (*Trichomonas*). A fringe domain alone was also identified in the studied Ascomyceta species; however no complete *Fringe *or *Fringe-like *gene seems to be present in this taxon (data not shown). We could hypothesize that the *Fringe *gene was present in the LECA and then lost several times; nevertheless, the most parsimonious scenario suggests three independent acquisitions. We can speculate that LGT might have occurred, favoured by either the tight association existing between Parabasalia and Metazoa lineages or *via *bacterial transfers [81]. However, we failed to find any specific relationships or signatures (Additional file 2) between the *Fringe *genes of *Homo *and its parasite *Trichomonas *as well as we failed to detect *Fringe *outside Eukaryota to strongly argue for a LGT hypothesis.

In our analysis, *Sno *is shown to be present in Holozoa and Plantae. Unexpectedly, *Sno *has been reported recently in a nuclear and cytoplasmic large DNA virus (NCLDV) of the haptophyte (taxon related to Heterokonta [36]) *Emiliania huxleyi *[82]. As our analysis on the genomes of the two chosen heterokonts (*Phytophthora sojae *and *ramorum*) failed to reveal the presence of *Sno*, we chose to extend our research to other heterokonta related species. Interestingly, *Sno *is not only present in the genome of the haptophyte *Emiliania*, suggesting a LGT between this species and its virus EHV (*Emiliania huxleyi *virus), but also in two other heterokonta, *Aureococcus anophaegefferens *and *Thalassiosina pseudonana *(Additional file 8). Another interesting feature is that *Sno *has been shown to be derived from the SNF2/SWI2 ATPases encoding gene of α-proteobacteria [83]. The presence of *Sno *or *Sno*-related genes in both NCLDV and α-proteobacteria may suggest LGT events in the history of these genes because i) α-proteobacteria are often found in tight associations with various eukaryote taxa (e.g: *Wolbachia*/Metazoa; nodosities of Fabaceae plants) and ii) NCLDVs have been reported from Amoebozoa, Haptophyta, Discicristata and Viridiplantae however their ecological distribution and importance is still largely unknown and newly described virophage of NCLDVs may also be involved in LGT [82-84].

In the two cases (*Fringe *and *Sno*), further analyses (on more species) are needed to shed light on the origin and history of these genes and to state whether they were acquired by LGT or not.

### The Notch pathway is specific to Metazoa

The cooption or acquisition (by LGT) of "old" genes is not sufficient to explain the formation of the canonical Notch pathway. One of the pivotal steps in the evolutionary history of the Notch pathway seems to be the transition between the choanoflagellates and the animals [85]. Indeed, this study reveals that the majority of Notch components appeared in the LCA of the Holozoa. Nevertheless, several molecular components critical for signal transduction are lacking in choanoflagellates, in particular, the ligand Delta and the receptor Notch (although we found a gene that possesses a domain arrangement similar to that of the metazoan *Notch *genes, it has very weak sequence similarity to these genes), thus we consider the Notch pathway as a synapomorphy of the Metazoa (this study, [47]).

An increase in the complexity of this pathway has also occurred after the divergence between sponges and other metazoans. Several Notch components are absent from the demosponge *Amphimedon *(Furin, Mastermind, SMRT, Numb and Neuralized), yet the pathway may still be functional in this species [30]. This suggests that these components were not critical for the function of the pathway and may constitute additional regulatory elements that were subsequently added to the pathway in eumetazoans. Nevertheless, the possible pan-metazoan ancestry of these genes (and their subsequent loss in *Amphimedon*) cannot be excluded; data from other sponges may help to resolve this issue.

The absence of Furin in *Amphimedon *is not really unexpected; although Furin has a critical role for the maturation of the receptor Notch in vertebrates, it has been shown in *Drosophila *that Furin is not essential for Notch signalling. Indeed, the Notch receptor can still be trafficked to the membrane without this initial cleavage [86]. Furin belongs to the PCSK superfamily which contains diverse families of proteases. Several PCSK proteins are present in *Amphimedon *although none seem to be *bona fide *Furins (as they do not group with bilaterian Furin in the phylogenetic tree; additional file 2). Nevertheless, we cannot exclude the possibility that one of these PCSKs may perform the S1 cleavage in the *Amphimedon *Notch pathway instead of Furin. Indeed, all PCSKs share the same canonical cleavage site R-X-R/K-R and (presently scarce) available functional data suggest that some of them may play similar roles in different cellular lineages [87].

The absence of a complete Neuralized in non-bilaterians is not incompatible with a functional pathway, due to the functional redundancy of Neuralized and Mindbomb [88]: the latter being present in non-bilaterians. Indeed, these two components are both E3 ubiquitin ligases involved in ligand endocytosis and regulation [27,28,89]*via *ubiquitylation [90], and were shown to be able to rescue each other in *Drosophila *[91,92]. A functional study of the Notch pathway in Placozoa, which lacks both a complete Neuralized and Mindbomb, would allow a better understanding of the effects of the absence of E3 ubiquitin ligase regulation.

Regarding the inhibitor Numb, it inhibits Notch *via *endocytosis and regulates cell fate acquisition by asymmetric cell division or by lineage decision processes [93-95]. Functional studies in sponges would be necessary to state whether another protein replaces Numb function. Nonetheless, it appears that the mechanism of Numb-mediated asymmetric cell fate acquisition is a synapomorphy of Notch pathway activity in Bilateria.

The co-activator Mastermind (MAM) is classically considered an integral part of the co-activation complex. Its non critical nature is highly unexpected and its absence from the demosponge species, as well as several bilaterian species, is puzzling. The high sequence divergence of the MAM proteins in bilaterians (MAM proteins share little sequence similarity apart from the N-terminal region [23], the region which interacts with Su(H) and NICD [96]) could make searching for them by sequence similarity alone inconclusive. Alternatively, these proteins may have been secondarily lost in several species, indicating that MAM proteins may be facultative for pathway function or replaceable by other proteins. In the absence of functional data on species that apparently lack MAM, we cannot distinguish between these two hypotheses.

### Recent acquisition of new functions: intervention of domain shuffling

It is clear from our data that novelty arose either in the LCA of Holozoa or in the metazoan stem lineage, which resulted in the assembly of disparate components into the functional Notch signal transduction pathway in animals. Our study further enables us to partly understand the molecular evolutionary mechanisms that may have facilitated these events.

Hereafter, we focus on the origin of the two main players, the receptor Notch and the ligands Delta-Jagged, all of which are metazoan specific multidomain proteins.

#### The origin and evolution of *Notch*

In the light of the recent data concerning sponges [30] and the present study, we can infer that Notch is a synapomorphy of Metazoa and consists of 3 core protein domains: EGF, ANK and LNR. Interestingly, these 3 domains exist in all eukaryotes. Proteins composed of EGF domains, LNR domains or ANK domains have been reported on separate chromosomes in *M. brevicollis *[47]. It has been proposed that the presence of these domains in separate *Monosiga *proteins suggests that Notch is the result of a new recombination of existing domains, known as exon or domain shuffling [97,98]. Data concerning the role of the LNR domains also found in the pregnancy associated plasma protein A (PAPP-A) are too scarce to infer the ancestral function of this domain [99]. The only common feature that we can note between the LNR domains of Notch and PAPP-A is a calcium binding capability [100]. In contrast, EGF and ANK are modular protein subunits, that are very common in eukaryote proteins and that are known to be involved in protein-protein interactions [101]. The ANK repeat is one of the most common protein-protein interaction motifs in living beings [102,103]. It has been primarily reported in eukaryotes, although examples from prokaryotes and viruses are also known and may be the result of lateral gene transfer [104]. ANK domains are not only part of the composition of Notch (3 to 5 ANK repeats) but also of Mindbomb (1 to 6). The ANK repeat is a relatively well conserved motif with strongly conserved residues (a Thr-Pro-Leu-His tetrapeptide motif and Val/Ile-Val-X-Leu/Val-Leu-Leu motif) and 2 α-helices [103]. We note that the Mindbomb ANK motifs are less well conserved than the Notch ANKs, suggesting that the structural integrity of the ANK motifs of Mindbomb are less constrained than in Notch. ANK motifs in Notch have a crucial role; they are involved in the assembly and stability of the complex with Su(H) and Mastermind [105,106]. When ANKs are deleted, the Notch signalling pathway is not functional in mice [105]. Mindbomb ANK repeats are important for the Delta internalization process but are not necessary for Delta ubiquitination [107]. As already mentioned, Mindbomb can be functionally replaced by Neuralized; this flexibility may have led to weaker evolutionary constraints on the Mindbomb ANK repeats than on those of Notch.

The two enigmatic domains NOD and NODP, the roles of which are still unknown, seem to be an innovation of Eumetazoa. Our analysis does not allow us to infer the process by which they appeared.

#### The origin of DSL proteins: *Delta *and *Jagged*

Notch has two possible ligands encoded by the two paralogous genes *Delta *and *Jagged*. Our analyses show that *Delta *was ancestrally present in Metazoa, while a complete *Jagged *is absent from Placozoa and Porifera. Phylogenetic analyses of the ligands do not provide conclusive results. As already mentioned, we can envisage that the ligands are evolving in a rapid and divergent way in each lineage, and this could cause the loss of ancient phylogenetic signals.

Experimental data suggest that Delta and Jagged may be complementary, functionally interchangeable or antagonistic [108,109]. They share two protein domains, MNLL and DSL, associated with the EGF repeats that they have in common with Notch, and are directly involved in receptor/ligands interactions. While EGF repeats represent an ancient domain, as previously discussed, MNLL and DSL domains are absent outside Metazoa. Their origin cannot be clarified by our study. Nevertheless, we may speculate that the DSL domain shares ancestry with the LNR domains of the Notch receptor. Indeed, comparison of cysteine patterns from these two domains revealed that, for 4 of the 6 cysteines, positions and spacing are conserved.

Despite the common characteristics of Delta and Jagged, they differ by two main features: i) a VWC domain present in Jagged and absent in Delta, the function of which is not clear but it may be involved in protein complex formation; ii) the number and spacing of EGF repeats differ between Delta and Jagged (an average of 7 and 14 respectively). Nevertheless, no correlation between the number of EGF repeats in the ligand and the affinity to the Notch receptor has been reported. Instead, Notch ligand choice is modulated by other proteins such as Fringe and *O*-fucosyltransferase that modify Notch EGF residues [110].

**Figure 13:**
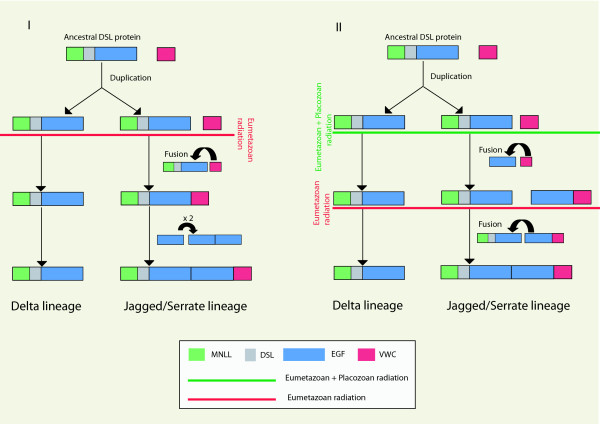
**Alternative scenarios concerning the evolution of the DSL ligands (Delta, Serrate and Jagged) in Metazoa, based on the phylogenetic hypothesis of **[46]. The EGF+VWC association found in the genome of *Trichoplax *may be considered either as specific to the placozoan lineage (scenario I) or as an intermediate step involved in the subsequent formation of Jagged/Serrate (scenario II). Domains are represented in different colours as indicated in the figure inset (signal peptide and transmembrane domain are excluded for clarity of presentation). Red and green lines indicate proposed occurrence period of the events.

It is worth noting that the sponges and the placozoan possess complete *Delta *genes (with or without MNLL domains) but *Jagged *genes seem to be absent. Nevertheless, in the case of the sponges (*Amphimedon *and *Oscarella*) and of *Trichoplax*, the VWC domain, (the specific domain of Jagged) is indeed present in the genomes, but it is never found in association with a DSL domain (data not shown). Intriguingly though, in *Trichoplax*, the VWC domain is found in association with EGF domains (7). These observations lead us to propose two possible evolutionary scenarios for the Notch ligands (Figure 13):

- An ancestral *Delta *gene duplicated before the radiation of the Eumetazoa, followed by an association of the VWC domain to one of these *Delta *copies. The number of EGFs increased either by tandem duplications within a gene (where a segment is duplicated and the copy inserted next to its origin), exon shuffling (which may be responsible for internal duplications of repeats) or DNA slippage (due to the formation of DNA hairpins) [98,111].

- An ancestral *Delta *gene duplicated before the radiation of the Eumetazoa, at which time EGF repeats were already independently associated with a VWC domain (the state observed in Placozoa). One copy of the ancestral *Delta *joined the EGF+VWC motif to create Jagged. This second hypothesis could explain the higher number of EGFs in Jagged compared to Delta (as the result of the addition of two series of EGF repeats). The fact that EGF motifs from Jagged seems to be physically separated into two groups, as shown in Figure 9, may support this hypothesis. This second scenario is also convincing because the shared possession of motif repeats (EGFs) between independent genes was previously reported to favour domain shuffling (non homologous recombination) with likely consequences the creation of new exon combinations and thus new proteins [97,111].

As we failed to find any specific signature in EGF repeats that could allow us to favour one of these two scenarios, the sequencing of additional non-bilaterian genomes may help to resolve this question. Nevertheless we have to keep mind that currently the placozoan phylogenetic position is still controversial [44,45,112,113].

## Conclusion

This study focusing on the Notch signalling pathway provides for the first time a complete description of Notch components and auxiliary factors across the Eukaryota. These investigations have enabled us to re-assess the ancient origin of some components such as the γ-secretase complex and *Notchless*. *Fringe *and *Sno *are probably old genes that were convergently acquired by lateral gene transfer. Several new functions of the Notch pathway likely originated in the last common ancestor of Holozoa, which already possessed 12 genes of the pathway. Nevertheless, the core genes needed for a functional pathway are only present in metazoans and it apparent that the two main players, *Notch *and *Delta*, emerged *via *both the shuffling of old domains (EGF, ANK, LNR), and the invention of new ones (MNLL, DSL).

At present, functional data on non-bilaterian models are scarce, but such efforts need to be realized in order to understand the emergence of functionality in the Notch pathway. More largely this will pertain to an understanding of the emergence of signal transduction pathways during the acquisition of multicellularity in the Metazoa.

## Methods

### Data sources and sequence retrieving

Genomic data (including 31 complete genomes) were used when available. If not, EST trace files were scanned instead; as was the case for four species: *Oscarella carmela *(Porifera), *Pleurobrachia pileus *(Ctenophora), *Mnemiopsis leidyi *(Ctenophora) and *Bigelowiella natans *(Rhizaria). As the *Amphimedon queenslandica *genome is still not annotated, sequences were identified and concatenated following the previously published procedure [85,114].

Regardless of the origin of the sequence data, TBLASTN or BLASTP searches [38] were carried out on genome data (including 31 complete genomes) when available with a cut-off E-value threshold of e^-25 ^or less. When BLASTs against genome data gave results, the sequences obtained were systematically reciprocally BLASTed against the NCBI database. In this way, we could confirm the validity of the sequences retrieved with the initial BLAST searches (reciprocal best hits [115]).

### Sequences analyses

Genes were scored "present", "absent" or "incomplete" (Figure 3). Genes were annotated "incomplete" when the domain composition considered as diagnostic was not recovered (for details see Table 2 and procedure for domain arrangement analysis hereafter) Genes were scored as "absent" only when BLAST searches against a complete genome gave no result. Abbreviations for species names are as follows: *Aae: Aedes aegypti; Aqu: Amphimedon queenslandica; Ath: Arabidopsis thaliana; Bfl: Branchiostoma floridae; Bna: Bigelowiella natan; Cel: Caenorhabditis elegans; Cin: Ciona intestinalis; Ddi:Dictyostelium discoidum; Dre: Danio rerio; Ecu: Encephalitozoon cuniculi; Ehi: Entamoeba histolytica; Gga: Gallus gallus; Hma: Hydra magnipapillata; Hro: Helobdella robusta; Hsa: Homo sapiens; Lgi: Lottia gigantea; Lma: Leishmania major; Mbr: Monosiga brevicolis; Mle: Mnemiopsis leidyi; Ngr: Naegleria gruberi; Nve: Nematostella vectensis; Oca: Oscarella carmela; Pfa: Plasmodium falciparum; Ppi: Pleurobrachia pileus; Pra: Phytophthora ramorum; Pso: Phytophthora sojae; Sce: Saccharomyces cerevisiae; Spo: Schizosaccharomyces pombe; Spu: Strongylocentrotus purpuratus; Tad: Trichoplax adherens; Tth: Tetrahymena thermophila; Tva: Trichomonas vaginalis; Uma: Ustilago maydis; Vca: Volvox carteri; Xtr: Xenopus tropicalis;*

For phylogenetic analyses, 18 alignments (one alignment for each gene except for *O-fut, SMRT *and *HES*, the latter having been recently reported in [116]) were performed using the online software Muscle ([117,118]) and subsequently corrected by eye in Bioedit Sequence Alignment Editor 5.09 [119] (additional file 1). The alignments were then treated with the program GBLOCKS with the least-stringent settings to release positions of uncertain homology [120].

For Notch and DSL proteins, the number of EGF domains is variable so they were excluded from the phylogenetic analyses. For the analysis of Notch, the alignment used includes only a part of the sequence from LNR domain to the end (749 bp). The DSL alignment used includes also partially the DSL protein sequence from the beginning to the end of the DSL domain (169 bp). Five sequences were incomplete in the DSL alignment (Delta: *O. carmela*; *S. purpuratus 3*; Jagged: *L. gigantea 2*; *H. robusta*; *B. floridae*). For ligand nomenclature, all genes that contain the VWC domain were named Jagged (prefixed with *J-*).

Phylogenetic trees were constructed from the protein alignments using the maximum likelihood method (ML) with the PHYML program under a WAG model of amino acid substitution [121]. To take into account rate variation among sites, we computed likelihood values by using an estimated gamma law with four substitution rate categories and we let the program evaluate the proportion of invariant sites (WAG+I+ Γ4). Node robustness was tested by bootstrap (BP) analysis [122] with 1,000 replicates. In addition, for DSL and Notch phylogenetic analyses, Bayesian analysis was performed with MrBayes 3.1, using the WAG fixed model [123]. Two sets of six independent simultaneous metropolis-couples Markov chains Monte Carlo were run for five million generations and sampled every hundredth generation. The runs were monitored for convergence and an adequate burn-in was removed (above 25% of tree and parameters). Bayesian posterior probabilities (PP) were used for assessing the confidence value of each node [124].

### Domain arrangements and composition

For multidomain protein coding genes, the presence of specific protein domains and the domain arrangements were checked by scanning sequences with Prosite [125], CDD [126] and InterProscan  online software [127].

In addition, for *Notch*, *Delta *and *Jagged *genes we used PSORTII [128] and PESTfind [129] software for identifying the nuclear localisation signal (NLS) and the PEST region respectively. For other regions and/or domains characteristic of the Notch receptors and Delta ligand that cannot be detected by the previous software (C-C linker, RAM motif, cleavage sites) conserved regions were identified "by eye" on the basis of sequence alignments and previous works [19,62,96,130]. It is worth noting that the prediction of cleavage sites was confounded by sequence divergence, such that these sites cannot always be stated with full confidence. For designing the Notch, Delta and Jagged compositions, MyDomains Image Creator from Prosite was used.

Five major genes of the Notch pathway were selected for more detailed domain composition analyses: the receptor Notch, the ligand DSL, Suppressor of Hairless, the ligand regulator Mindbomb and the enzyme responsible for the S1 cleavage, Furin. We used multiple software platforms for gene domain prediction (Prosite, Interproscan, SMART [131,132], Pfam [133], Superfamily (supfam.org/SUPERFAMILY/) [134]). Evolution of these five genes among eukaryotes was discussed according to two previously proposed rooting hypotheses [36,135]. Two conflicting hypotheses for the position of the root of the eukaryote tree are currently recognized [36,136]: subdivision of eukaryotes between opisthokonts + amoebozoans and bikonts (all remaining eukaryotes, on the left of Figures 10, 11, 12) [135], and the more classical rooting on excavates (on the right of Figures 10, 11, 12) [137,138].

## List of abbreviations

ADAM: A Disintegrin and Metalloprotease; APH1: Anterior PHarynx defective 1; APP: Amyloid Precursor Protein; ANK: Ankyrin; BI: Bayesian Inference; BLAST: Basic Local Alignment Search Tool; BP: Bootstrap; CA: Common Ancestor; CBF1: C-Repeat/Dre Binding Factor 1; CDD: Conserved Domain Database; CSL: CBF1, Su(H), Lag-1; DSL: Delta Serrate Lag-2; EGF: Epidermal Growth Factor; EHV: *Emiliana huxleyi *Virus; ErbB4: Erythroblastic leukemia viral oncogene homolog 4; E(Spl): Enhancer of Split; EST: Expressed Sequence Tag; HAC: Histone ACetylase; HDAC: Histone DeACetylase; HECT: Homologous to the E6-Ap Carboxyl Terminus; Herc2: Hect domain and RLD 2; HES: Hairy/Enhancer of Split; HEY: Hairy/Enhancer of split related with YRPW motif 1; IPT RBP-J Kappa: Recombination signal Binding Protein for Immunoglobulin *kappa J *region; LBA: Long Branch Artefact; LCA: Last Common Ancestor; LECA: Last Eukaryote Common Ancestor; LGT: Lateral Gene Transfer; LNR: Lin12/Notch repeats; LUCA: Last Universal Common Ancestor; MAM: Mastermind; Mib: Mindbomb; ML: Maximum Likelihood; NCBI: National Center for Biotechnology Information; Ncor: Nuclear receptor corepressor; NCLDVs: Nuclear and Cytoplasmic DNA virus; NECD: Notch Extracellular Domain; NEDD4: Neuronal precursor cell-Expressed Developmentally, Downregulated 4; NICD: Notch Intracellular Domain; Nle: Notchless; NLS: Nuclear Localization Signal; O-fut: *O-*fucosyltransferase; PEN2: Presenilin Enhancer 2; PHYML: Phylogenies by Maximum Likelihood; PP: Posterior Probabilities; RTK: Receptor Tyrosine Kinase; SMART: Simple Modular Architecture Research Tool; SMRT: Silencing Mediator of Retinoid and Thyroid receptors; Sno: Strawberry notch; Su(dx): Suppressor of Deltex; Su(H): Suppressor of Hairless; TGF-β: Transforming Growth Factor β; VWC: Von Willebrand Factor type C; WAG: Whelan and Goldman.

## Authors' contributions

EG, FB, GR, PL, and BDM retrieved the sequences used in the study. EG, GR, and PL made the sequence alignments and performed the phylogenetic analyses. EG and GR performed domain analyses. EG, ER, AEV and CB conceived the study. EG, ER and MV designed and coordinated the study. EG, ER, CB and MV drafted the manuscript and all authors participated in the editing of the manuscript. All authors read and approved the final manuscript.

## Supplementary Material

Additional file 1 **Alignments used for the phylogenetic analyses**. The data provided represent the alignments used for the 18 phylogenetic analyses. Click here for file

Additional file 2 **Phylogenetic analyses**. In this file we provide the phylogenetic trees constructed from the protein alignments using the maximum likelihood method (ML) with the PHYML program for 16 Notch components (excepted Notch and Delta/Jagged). Click here for file

Additional file 3 **Diagnostic domains table**. In this table we report the presence or absence of the domains that compose each protein in all species. Click here for file

Additional file 4 **"Notch-like" in *Monosiga brevicollis***. The sequence of *Monosiga brevicollis *presenting a domain arrangement of a *Notch *gene is provided. Click here for file

Additional file 5 **Notch phylogenetic tree**. Notch phylogenetic tree constructed from the protein alignments using the maximum likelihood method (ML) with the PHYML program. Click here for file

Additional file 6 **DSL phylogenetic tree**. DSL proteins phylogenetic tree constructed from the protein alignments using the maximum likelihood method (ML) with the PHYML program. Click here for file

Additional file 7 **Unusual arrangement of DSL domains in *Nematostella vectensis***. In this file we report the sequences of *Nematostella vectensis *presenting an unusual arrangement of DSL domains. Click here for file

Additional file 8 **Strawberry notch sequences**. In this file we report the Strawberry notch sequences identified in two heterokonts. Click here for file
