# Supplementary material for: Origin and evolution of the Notch signalling pathway: an overview from eukaryotic genomes
Source: BMC Evol Biol. 2009 Oct 13;9:249. doi: 10.1186/1471-2148-9-249 (PMC2770060; doi:10.1186/1471-2148-9-249)
Supplement: Additional file 1 — Alignments used for the phylogenetic analyses. The data provided represent the alignments used for the 18 phylogenetic analyses. [file 1471-2148-9-249-S1.DOC]

**ADAM**

>Ecu

KAIPVE------------IPSSSDFVMAFMAFGRKFRLRLS-DSSLKIACLGEEKCYGSASFCN-SIHGLFVSAGTVYQIRS---------------AIKVFLINDFDRVFEKNKW-NIRGIELSLNGIELSIEMLRTLSRMGVEMLEKSNLVLLLQTSIKINGLTFVG-------GMGSAAKR---------FGIIKVSE----DSYFYKGKVLAHEIAHALGAEHE----------EGGRCLMREEESPLEKEESAALSHESIEKIESFISRN-ESKFGEID--TCGNGIMDGKKECDAGL----NGSVCCT-SKCKLRAWAQCDDRNGRCCK-DCGLLPKNTVCRGRTSNCERESYCDGKSPACRARSASEGI--CKKIMCE------RRFSSPKCLSRD---RLWC-----YDVC----------------SLSSPIML

>Mbr1

VDLNLDLHAEHGRVRRSG-RRDATLPFSLDVFGLHFAADLKPDHSMIGKPHGMPGSSVHVTMFNGTLHGRIHTRTETYNIHPYRSSDVAYNMSHD--CCEMALVADHRFIYKFTDFDIGTGIQLNVATIIIPNLFLDQL-----------SSVSWGDFAEGVLGLARVG-------QICQTPYSATTTGLSLNTGITTTINYGST-NEDQSKLVFAHEVGHNFGASHDNPGRCEADGSENNYFIMWPSSVDGSKSNNDQFSDCSISSMSSQINSNSGQCFTNRADAICGNGIVELNEECDCGSTDTCAADKCCT-TNCTLAVGAECSPEAGTCCNDNCELTGYDTRCYTYADDCYTDIYCLELLGRCPSLCNDDAN-VCTIG-CTGSLCSLYTDRDNTCESTDNPCDVACEFK--SGTCGKACGFCDGNGQCYQASGASPLDL

>Mbr2

IRIHPDTPSRYTRDLTPK---TRSFSLNIPVYDEELTLIVQPNTELFGYVAQYPASHLHLRVDKGSCFAQIITPTEMYAVEPYRLSDHVNPVSPNHSDCDVAVIADRNFLYEDNNIADGVFVGIKIGRIEVGEDFLHNFAGENWEDYCLAHLVTHQEFSDGLLGLAYLG--DSTPGGICQKKSS-----VYTNTAFSTTLNYGSAVPELTHWLVIAHEIGHNFGSTHD-----DPSGSANGEYLMYPVSVDGSESNNFLFSPESKSLMTAVVEAK-GGCFVDSDASICGNLVQEGDEECDCGTSG-CAADECCNVDNCNLRAGKQCSPTNGACCKSDCSFQNDGFVCREESQ-CVREATCT--TGECPTYCSSYKDKMCSEGRCTLSICDAFGLEEGFFSTE-NQCKVSCVEDGEVNAAGSKCGVCTTEGNCESAEGGRT---

>Mbr3

LPFGAL--TTRARRRRSY-DEAAGLMVRLEALNTTLELELEQNQHLFGRVVGESSSRVWLYMDGDHIHGRIQLADDTIYLEPYRKSDLRPDGWRMGQCCTVTVVADYRFIFRATTFNSYSGLGVAVDTVIIVSDMLRQFSESDFSDTCLAHLYTREDFDGGTLGLAWVGSPSNRYGGICYSSSS-----QSLNTGLTTNINYGNTIPFATTALVSAHEIGHNWGSSHDTSTQCVPSDSEGGKYIMYAVAVDGSDPNNDNFSPCSTASISSVIAAK-GDCFIVPPAGRCGNSRLEPGRDGDTSTNDECTADNCCN-TECKLKSSSTCSPANDECCSPTCRGFLQSDTCFVPYDYCRALAVCTSGEPACQSVCGNGGR--CTGDRCI-TFCERFGAVNCTCSDEEEECSTCCQHNDEDNPCGTICGYCNFAGSCE--TPDTGTAR

>Mbr4

LSFSRR---------------PLPSLALCAAGTRNLTLAFRPDDDLLGTATEHPHSVARGVLLEGIFQGHLVFSGRVLHFEHYYRDALRPEALAPINCCASS-------SLKRTNWSSLVGIGVAVQSLTIVEALLSAFSDNDHSASCLAHLFTHQDFAAGVLGLAYTGSPTRATAGVCAVETS-----GTLNTALTTDLNSGTTQPDGLVSLVTTHETGHNFGASHDNGTDCDPSG---NYYVMHTFAVDGSQPNNNDFSDCSKESILEVLESPDTNCFVPTPAAICGNGVWEPGPDDEAGT----SDDEECDAADCKLTSGSSCSDYNQDCCQ-DCQYSATR-ICYQSFDLCFATTTCEPGVFECPTPCLNLGS--CT-PRCR-PFCERFSATDCDCSAE-DSCRLCCRHDASCPRNGTSCGYCNEMGVCVA--ATDSLDD

>Hma1

-------------------------------------------HEIFGEVEGHQKSHVLGKIDDGRFEGKIHLGKDEYYIEPYNLNDVEHER-----KCNLKLVADQLFIFRNQTFYSPEGIGFRIKEVHVAYELLEQFSRMNHSSVCLAYLFTDRSFEDGVLGLSWIA--YGQPGGICDPYANYGGIWKTYNTGLVTVRLYNREAPMAITEISFAHELGHSFGAQ------------------VYAKKMIKYFKF-------------------------------------------------------------------------------------------------------------------------------------------------------------------------------------------

>Hma2

ITYDTE--DVHRRSRRALLSPYANVHLKFNAFNRNFTLHMRRNHEIFGEVEGHQKSHVLGKIDDGRFEGKIHLGKDEYYIEPYNLNDVEHERKF----CNLKLVADQLFIFRNQTFYSPEGIGFRIKEVHVAYELLEQFSRMNHSSVCLAYLFTDRSFEDGVLGLSWIAYQYGQPGGICDPYANYGGIWKTYNTGLVTVQLYNREAPMAITEISFAHELGHSFGAQHDANKDCQPGIERGGNFIMYDKSTSGYLKNNKLFSRCSIARMIPVINAKTIGCFTDRYWAICGNGAFEEGEICDCGTESTC-VEKCCTPTPCTFRNPAQCSPSKGPCCLNTCTIAPQYLQCFDGTE-CMESLVSNGLHFTCP---------------------------------------------------------------------------

>Hma3

-----------------------------------------------------------------------------------------------------------------------------------PDNLLAAFSRSDWSKVCLAHLFTYQDFDNGVIGLAYVATQTNEHGGICSNNYTDATGLRHLNCALSXSFNWGRKLLTVEADLVTAHELGHNFGSNHDVSPPCVSST-SRGNYIMYATAVSGEHDNNKRFSPCSIDAISKVLKAKKDKCFE------------------------------------------------------------------------------------------------------------------------------------------------------------------

>Cel1

IRFQRS---------------TRQSVVHFEFLDQEYVVDLEPNHSTFGTV--EPAGRAVLTQLENVYIGSLYFVDDTLHLEPYFESDLDL-------LCTLKLVADYSFIYTPINWGRFQNMGFSIKEIKVVERLLREFAFAEGSKFCLVHLVTARTFEVATLGLAYVSYKDETAGGICSKQETFNGRVAYINVLLSTSFASEQSLITKEIDIVVSHEYGHAWGATHDDVEECSPNDQNGGKYLMSQYAQKGYDANNVLFSPCSRKLIRDVLIGKWESCFQEEMTSFCGNGIVEDGEECDNGVTD--NEFNCCD-KFCRLAVGAKCSPLNHICCTPTCQFHNSTHVCLPGDSLCKADAVCNGFSGECPQECLEGGE--CLNGVCL-PFCEKMGKKSCICEDLELSCRLCCRDY--NGTCGVRCGSC-RDRKCVNEVVDNVRNY

>Cel2

LNYRATVANQVTRRKRSISHYQEPIGFRFNAYNRTFHVQLHPDDSLFGYLKDDPNSHVHGSVFDGVFEGHIQTEGRRYSIDKYRDDEINHRKWRVKRCCSLYMQADHKLIYEGTNFNGIKGLHFVIQRTSIVSNFLNLNSQRNHSAFCLAYALTFRDFVGGTLGLAWVASPFNTAGGICQVHQRYNEGSRSLNTGIVTLVNYGNRVPARVSQLTLAHEIGHNFGSPHDFPAECQPGL-PDGNFIMFASATSGDKPNNGKFSPCSVKNISAVLTRNKRNCFQERTSAFCGNQIYEPGEECDCGFQADCMGDKCCVPGPCKRKPGAQCSPSQGYCCNPTCSLHGKNEICRQESE-CSNLQTCDGRNAQCPIPC-QDSTKVC-SGQCNGSVCAMFGLEDCFLTEGKELCFLACIK---DGKCGSPCGYCDIFRKCRSVDANGPLAR

>Aqu17

LHKDNVRHKIVRDNMPRL--EDNLIELHLDIHSKYFRLQLSPTIESFGHVFGAEFSDVTAHMTEGIMTAIIVLDIETYYVEPYKASDIKYNMTGDANCCPMFLVGSTSFIYRTTDWATPGTKGLQIAGVNISENLLREFSRTDWSRYCLAHLFIGSNFDNSRLGVAYIADASFYLGGVCSRRTSINGVAFSGNTGISSFRARSRLLLLAEAVIITAHEIGHNWGAQHDSSSECSPSPENGGKYLMYPIAQDGTADNNNVFSPCSVRGVSRVLNTHESDCFTAPSNNLCGNFIVDVGEQCDPGPTGSG-GDMCCT-VGCRYKGNATCSDTNHECCQ-GCFISREGSLLRQYGNLCLANETCNGMDKTCPTPCGFEGI--CVDNECR-QFCEQMDLETCHCTGE-DQCKICC-----SGACGSFCGSC-QQGTCVL-SDPDLVTF

>Aqu10

VKYDSRLLRQHYKLRRGNGGPGQHIKLAFSSHGRDFDLRLHPDSRYLGYDANDPETTVHGNLAFGLFDGAIHTKDEIYHIEPYKHSDVNLSGGQGHHCCWINVVADHFFIFRGEDFGTPDNINFAIQKITIVETLLNEFSKADWSDFCLSYVFTNRDFEGGVLGLAFVANP--RNGGICEDMYG-----KTLNTGVVTMVNYNQRVSNAAATLTFAHEAGHNFGSEHD-MGSCNPSD---NPYIMTAFANDGTKSNNDNFSPCSIDAMKVIIASKRNGCFRESTD-TCGNYLLEEGEDCDCGVHTICDNDPCCNATSCKVVNGRECSPQSSSCCYSNCSIIPVDSVCSDETE-CAFQQMCNGSVQ-CPISC-NNGANYCVNGQCTGSICVPLRLSDCECFSDGEACHVCCSV---EGTCGYPCGYCDFYERCRQVQKSGALAR

>Tad1

LQLADFTSQNTRSNTNGCGLSCRIESILFTAFDRQFNLNLWPRSGLFCSLDGDLASEGSFYIDEYTILGTFEAINETYAIEPYRQRDLKYNSYNNSRCCKLALVADYRFMYRSESFDNMPRIGFQISKIIVIQSHLKLFSLKDWSQVCLAHLFTYQDFADGTIGLAYVASQQDTVGGICSPRIRNGNDYVSANVGLSTPLNWGKRMTTLEFQLVLAH--GHSFGSEHDLEGVCSPSEAKGGKYLMFPAALSGLNSNNKLFSSCSKDSIKGVLQAKSQLCFTERSKSRCGNYEVEEGEECDAGIGNKG-RSSCCT-PNCKFRPNAVCSSQ-------------------------------------CPAPCIGGGV--CQNGICL-TFCEKYDKSPCICSEDNISCQICCKDNA-SAICDNPCGIC-KKGKCSK-TVQDFITR

>Tad2

INYDAEFAEHHERSRRSP---SAHTMLEFHAYHRHFQLRLKRDTSLYGHLVDDAKSLVHGTIVDGIFQGKIYTEEEQYYIEPYRRSDVRVKKSYQVGCCQLLVQADHTFIYEVIDFSSADGIGFIVRRMKIVSSYLDITSTANHDPYCLAYTFADRDFDNGVLGLAWVGS-ATGNAGICAKNSAYKGQKMSLNAGIVTILNYGTRVSTAVTEVTFAHELGHNFGSQHDTNTQCAPGS-PQGNYIMYPQATTGRDPNNKQFSSCSKNYMQPVINAKSSGCFVNRATSICGNGVVEGNEQCDCGYRSDC-QDNCCNAQACTLKAGSVCGQSSGICCNPNCRYYESSDLCTVSTE-CSQNAYCNGSRYQCPTSCRKGSN-VCSNGDCDGSICLAADLASCACTTQADYCNTCCKS---GNTCGSSCGYCDVFGKCRAVNENGPLTK

>Tad3

LNYDTTRLRKSNRIKRSV--GDSPVSLNFRAFDRHFKLRLEPDNGLFGKLADESDSEVYGTIVDDKFHGKIISNGDEYYVESYRKSDIRFKRSHPISCCQLLVQADHTFMYASTDFGLADNIGFIIKRIKIVSKYLDIASQANHNDFCLAVTFANRDFENGVLGLAWVA--AGGAGGICDRFQPYKGQSMSLNSAIVTILNYGSHVSTAVTEVTLAHELGHNFGSQHDTNPTCAPGG-SVGNFIMYPKATAGNKVNNKKFSSCSKNYIKPILNSRSGGCFRSREAAICGNGVVEAGEACDCGYQR-C-KDSCCNAKPCQLVTGKTCSPSAGPCCNPTCNYQPAAGSCAKAGE-CSLEAFCDGTNYTCPTTC-ANGVKVCKEGQCEGSICEKFGQVSCTCTVTTDLCSICCMNKTSKCAVGTPCGYCDVFGKCRQVNEDGPLNR

>Cin1

-------------------------QISFNAFNKTFRLSLLPSPEVFGTVLGDSKSHVIAHLQDGRLTASIDTDNETYIVEPYRRSDVKLQSLNDPHCCKITLVADHRFIYKNTDWGVYKGYGFQIDNITIAKQLLEAFS--EKKRNCLSHLFTYVDFARGLLGLAYVGSGKDDVGGICTKPYNRGNGDLYLNTGLTTTVNWGQRILTTEADLVTAHELGHNFGAEHDSADNCRPGQANGGNYIMCPAAVTGEYPNNKVFSVCSKRNILRSLKNKAPLCFQEEKNSFCGNFQVETDETCDVGYTGFSNEDRCCL-FNCTLKPGAKCSNKNYKCCTEDCQIAGPTKMCRAHIPGCLKDVYCNGVDKVCPTMCGDLGR--CRNGVCE-PFCRTKGLQPCLCSLLEQFCSRCCAPLGVTNLCNSRCGYC-RKGKCHK-QTQDVIER

>Cin2

LKYDVKLAYQHERTRRSVSSSTKPVKLKFQAHGRNFDIHLHQDRTIFGVLYGQRESYVHGSIISGIFRGVIHENVGKFYVEEYHENDVIHPKKFVPLCCLLYIQTDHILIYTRTLFTTIRDINFMVQRIRIVEKFLDIASLANHNDYCLAYVFTNRDFDNGVLGLAWVGSPTGTSGGICEKYRTYTDRSKSLNTGIVTFKNYGTVMPAVVTHITFAHELGHNFGSPHDGGVECTPGEKKQGNFIMYARATSGKEPNNDVFSGCSIRNMSRVLEAK-MSCFVSSDTPRCGNNLIDEGEDCDCGYDTDI-GDVCCTPRRCKLKAGKSCSITQGPCCNGTCEAKNSNYRCSEETQ-CQLAQVCKPFKAVCPEIC-NHGTQICVQGTCSGSICAKHGLEKCECEIPPLLCHTCCKLPGNSSTCGSACGYCDVFSKCRKVDADGPLSR

>Aae1

LHADDL---THRITKRGAHPFNTIKEVEFKVLGRNFRLILHPHKEVLGRVFGESDSHVNAHLENGILTASVVLPDETYHIEPYKQSDIKFSWENVDACCPLLLVADYRFIYNDTIWEGFKGMGFVIKKIVVVRNLLEVFREYSHKDFCLAHLFTDLKFEGGILGLAYVGSPRNSVGGICTPEYFYT---LYLNSGLSSSRNYGQRVITREADLVTAHEFGHNWGSEHDDIPECSPSASQGGSFLMYTYSVSGYDVNNKKFSPCSLRSIRKVLQAKSGRCFSEPEESFCGNLRVEGDEQCDAGLGT--DNDGCCD-KNCKLRQGAVCSDKNSPCCQ-NCQYMMSGVKCREAQYACEQEARCTGNHADCPTMCQERGQ--CRNGKCV-PYCETQGLQSCMCDTMADACKRCCRQSI-NETCGTPCGFC-NKGMCEK-TIQDVVER

>Aae2

LPLDLEVPYNDDYSLTRNSRIITRTSLTNGGSGQTVNKNI--NTNLFNTYNGDPNQRIITRTRN--VIGNVYDNGNQSNSNPTKNGSTKKNIIGNNYLCMLYLQADHTFIYRKTDFGKPDNITFMIKRIKVVEKFLELFSEEDYDAFCLAYMFTYRDFEMGTLGLAWTG--LKNAGGVCEKNGHYRGSLKSLNTGIVTLLNYGKHVPPAVSHVTLAHEIGHNFGSPHD-PEQCTPGG-EDGNFIMFARATSGDKRNNNRFSPCSLKAIEPVLNAKAKGCFTEPQASICGNGVVEHGEQCDCGWEEDC-KDSCCYPKPCTLTPKAQCSPSQGPCCTLECTLKTGDK-CRDDNG-CRDPAYCDGSMPLCPTICN--KEYVCY-GECTGSICLAYGLESCQCAVGPRACELCCKLPGEDKPCGTPCGYCDVSQKCREVDPSGPLAT

>Aae3

LSYDHKLHASHSRAKRSV-TKDHHVYLRFKAHGRDFNIRLRRDLSTFGELLGDPDSHVFGSIIDGVFEGKVISSRDSYYVERYNEKHVDDPYRQRRTCCSLYIQTDPLIIYRNTKFIEHRNIRFEVQRIKIVSNFLNLHSLGNHEIFCLAYVFTYRDFTGGTLGLAWVASASGASGGICEKYKTYTETVRSLNTGIITFVNYNSRVPPKVSQLTLAHEIGHNFGSPHDYPAECRPGG-INGNYIMFASATSGDRPNNSKFSTCSVRNISNVLDSKKRNCFQASEGAFCGNKIVEIGEECDCGFDEEC-ADKCCYPKGCTRRARTQCSPSQGPCCDSSCKFVSSFSTCKEETE-CSWSSTCNGTTAECPTKC-NNGTQLCIKGECAGSICLLWNMTECTSNIIPKLCELACQNGNDTSTCGSPCGYCDVFLKCRAVDAEGPLVR

>Lgi1

LSTLED-VKRTKRSTDGI-VRNTDRDIKFSAFGRTFDLLLKNNDDILGRLAGEKDSMVDAHVENGKWTANIYTAQESYHIEPYRASDIKSNF-----KCQIYVVADHLFIFRRTKWTDLTNIGFQIKEIKITRDLLEVFSRERYHDFCLAHLVTYQKFSGGVLGLAYIASPQRTVGGICSPAYMKDKIRLKLNTGWSSSKNNGDRLLTKEAILVTTHELGHNWGSEHDETDECAPSSTGKGKFVMYPYAVKGYDENNDKFSPCSRRYVRDVLIAKASNCFTDLNAPYCGNGKIDSGEECDGGRGK--GNDPCCS-KDCKLIGNAVCSNVNYECCV-DCQVASTDTVCREAFKGCTKTAKCSGSLE-CPTQCLEGGE--CYDGICV-SYCELNNAEPCRCENNEDACHRCCRDQ--QGKCGRACGSCSADGVCKR-NEVDMVQR

>Lgi2

LNYDTKFHANHERVKRSI---EPHLYLKFKAFDRDFNIKLKQDKTIFGHLTDVPTSYAHMAVINGTARGFIRIGDAIYHIDPYSEKHMDLDPYQFGPICNLYLRSDPMLIYSRTVFIQWSGVNFVVQRTSIVSNFLNLNSLDNHDDFCLAYVFTYRDFNQGTLGLAWVGAATRAAGGICEKYKKYPENDKSLNTGIVTIVNYGKKVPSRVSHLTFAHEVGHNFGSPHDTGVECAPYGAAMGNYIMFSSATMGDKDNNDNFSKCSKDNITRVLDAVKVNCFQSSTTAFCGNGLVEEGEECDCGYSD-C-TDKCCYAKECTRRPNKICSPTAGPCCSVECEFLPKSEVCRPSDS-CTKESLFTGGSSSCPTFC-NDKTQVCLEGECRGSVCLRIDWGECTSTEGDELCFIACKNPQ-TGDCGSPCGYCDVFHRCRGVDAEGPLAR

>Nve1

---------------------------------RHFTLQLSPNSGLFGTVQGEKDSHANLHVDDGVLTAMISTPNETYVIEPYRHSDVKFNFTGNKTCCPLALVADYRFIYKSTVWVNYGGFGFEIGQVIVIKKLLETFYEKTWRDYCLAHLFTYQDFADGVIGLAYVGNARNAVGGICTEEYRTSSRTLYLNTGLSSSVNWGRRVLTEEADIVTAHEFGHNFGSEHDEGEECAPDERNGGKFIMYPASVSGQRDNNKKFSPCSKRQVSYVLKSKSELCFKEPRDIICGNYKLEEGEECDPGNLGVT-GTKCCT-RDCKLVKDTQCSDGDSPCCE-GCQFKAKDKICRXADPQCTVETFCTGKSASCPYTCIEGGK--CRSGKCV-EFCITEGLEPCLCNEGADACRVCCQ----NAPCGRICGMC-RQGKCEK-TTQDVIER

>Nve2

LDYNPTFHAIHRRSVLDG----SHYELSFEAFGRERRIRLRRNTGVFGEVVGEPGSAVYGTMEDGKFQGKIQSNTESFYVEPYRSEDVE--------ACTLALYADHLFIYKNAFNYSPYGITFRVKKMVIIDVMLNLLSSDDHSDVCEAFMFTDRDFDNGILGLAWIGK-PNFLGGICSRYSKVGGQYISYNTGVVTLKLYRLFTPPKVSEVTFAHELGHGFGSEHDEDGDCSPGG-KDGNYVMYSKATSGDRPNNDVFSSCSLKAIRDNINDKYSGCFISADTPICGNRIIEGNEQCDCGDENSCAEGGCCNPQACRLTLPATCSPSQGPCCGRDCRYVGNDISCRNKTD-CLDKAMCSGSSVECPTVC-DKGRRVC-SGECAKSICTKYALEECQCTADNDLCTVCCKEPGRDDTCGSPCGYCDAFAVCRRLDLDGPLKR

>Nve3

LSYNSQVEKQHGRHRRSVDPNSNPIILNFLAHERKFKLRLRRDTSIFGEVLGHKKSLVHGFILDGVFEGKIHIGDDEYHVEHYHTQAVEYPYPYGR-CCRLYMQADHLYIYQGTDFGNADLITFVIQRFLVVAKLLELNSQQKKNEYCLSYIFTYRDFDDGVLGLAWVGDTTGSSGGICENWKSFTDGHKILNTGVVTFINYGKDVPQKVSEITFAHEAGHNFGSPHDITSACSPGD-SDGNYIMFPRATSGEKSNNRKFSTCSRDKMYLVLQAKEKCCFKDSQEAICGNRVVEEGESCDCGYQDSCTADKCCLGTGCTYKNGATCSPSQGLCCNGTCSPYPGNSLCQNETE-CRNQSFCNGINATCPTIC-EDGRKLCINGTCQGSICMKYGYKECQCTAEPKQCDLCCDVG--DGECGSPCGYCDVFLKCRKVDADGPLNR

>Nve4

------------------------------------------------------------------------------------------------------------------------------------------------------------NF----------------------------------NTAVVSLKNFGARVLRKGSVLTTAHELGHSFGSEHDDNTLCRPEG-EPGYFIMYDLAVDGKKPNNFLFSECSKAQMWSVIFNKGPKCFIAQNQAFCGNSIVEGGEECDCGAPQRCEVDVCCHPPECRVKPEYQCSPQKGLCCESNCTLSAHKT--------CRKETECSGATAVCPTTCGDDGN-TCLDGACVGPVCARY-LDQCECQGQDQMCLVCC-----KGTCGSTCGYCNQLGQCITVKTGDQLLR

>Ppi

PCYPSS----------------STSSLPPMLYRRSHPFTPTPLTHIFGQIDGHHDSEVVLHVQDGDVMGTVEMGGRVWYLEPYERNQTRFNISGEF-CCNVILVADHKFMFRTSVFESFTGFGMQIKEIIIETDLLDSFYGGKWEDVCLAHLFTYQDFSRGVLGLAYVAHPRNKVGGICATPYINRQ--RMLNVGLTTSVNYKRTLLSTEMELVTAHEFGHNWGSSHDGSRICAPKD---NHFVMFPSAVDGSRANNKVFSPCSRSAIGSVLQYK-SHCFTERSSNVCGNGLVEDGEDCDPGE----QDDPCCT-ETCKFTKGSLCSDRDSECCE-GCRFAGNSKVCYRNSGTCVEEKRCSNTGAVCEEPCFDQGS--CDNGVCL-DFCASQGLQLCRVSS--APCHWSC-----GNVCGKMCGVC-SNGTCLVEAQP-----

>Ocax

-------------------------------------------------------------------------------------------------------IESQQS-------------------------------------FCLVHVFTYQDFSGGLLGLAPIASSQNKLGGICSSPYIRFQKLHHTNVGYSTGINWDRKLLIMEQQIVLQHELGHNWGSPHD-PQSC-----QNGKFIMSASAVSGGDPNNFKFSTCSRASALGVLRSK-SSCFTAKETSFCGNKYREPGEECDGGGLGSSAGDQCCD-ENCRYRNGAVCSDENDECC-------------------------------------------------------------------------------------------------------------

>Oca14

----------------------------------------------------------------------------------------------------------------------------------------------NHD---LAFLITAIDFDFSTVGVAHYN-------GACTSTSA-----------VAVIHNTGFT---QSTATTIAHEMGHSLGMAHDDDRSCDICP-PDSICIMAGSSVIGHPPS--LFSQCSEQDYSDFLEAGHGTCLFNIPTTICGNGLIEDGEECDCG-------------TSCTLQPGVQCA--SGECCNTQCTFLAQGVRCRENVTVCDLPEYCTGTSGKCP---------------------------------------------------------------------------

>Bfl2

LKTTDLRHHFLSRHKRSPHPPPYPRQMEFDTLGKHFHLYLEPRTGLLGQVLGEPGSTVEAHLEEGVLSARVTVGEDIYFIEPYRARDVKFNFTGAGAACPMQLVADYRFIFRCTQWKDMGGFGFEIKEIKVVRDLLANFSLHDYSEFCLAHLFTYQDFDLGVLGLGWVGTDKAGIGGICTDVYRVT---LYLNTGLTSTLNWNRRILTREADLVTAHELGHNFGSEHDDDPECSPNPEQGGKYIMYQVAVSGEERNNKLFSNCSKRSIYKVLSAKHSLCFVEPQKSLCGNYRVEEGEDCDPGHGN--NTDPCCT-GDCKFKGNAVCSG-----------------QCRNGT---------------CM------------------PFCESRGLQSCLCQNVSESCNVCCMNMSHLGMCGKPCGYCNMEGKCEK-QAQDLVNR

>Hro1

----------------------------------MYLGTLLQSAERASHVDGKKYSNTIFKYQDGPYTGFTFAVHR---VQ----------------CSSSFSAQQHHHTNPFCKW---------------VSYFLNLNSLSNHDLFCLAYVFTYRDFSHGTLGLAWVG--LRASGGLCERYKHYNENHKSLNTGIVTLVNYGSRVPLKVSTLTFAHEVGHNFGSPHDFGESCIPSLNLNGNYIMFASATSGDKPNNHEFSTCSINNITLVLEAVKDNCFSQSEEAFCGNGMVEEGEECDCGFLD-CRMDRCCYEKHCTRKPNATCSPSEGPCCLGRCNYVQASPVCMHEQE-CSETSYCKPFHLSCPTLC-NFKTQICKNGECAGSVCEMVNWSVCEAPVEDQLCYVACLNPV-TSACGAPCGYCDVFHKCRAVDEEGVLFR

>Hro2

----------------------------------MYLGTLLQSAERASHVDGKKYSNTIFKYQDGPYTGFTFA---VHRVQ----------------CSSSFSAQQHHHTNPFCKW---------------VSYFLNLNSLSNHDLFCLAYVFTYRDFSHGTLGLAWVG--LRASGGLCERYKHYNENHKSLNTGIVTLVNYGSRVPLKVSTLTFAHEVGHNFGSPHDFGESCIPSLNLNGNYIMFASATSGDKPNNHEFSTCSINNITLVLEAVKDNCFSQSEEAFCGNGMVEEGEECDCGFLD-CRMDRCCYEKHCTRKPNATCSPSEGPCCLGRCNYVQASPVCMHEQE-CSETSYCKPFHLSCPTLC-NFKTQICKNGECAGSVCEMVNWSVCEAPVEDQLCYVACLNPV-TSACGAPCGYCDVFHKCRAVDEEGVLFR

>Spu1

IHSSSVRHHYAKRSADEM--EMGVRRMSLQMLGRQFNMELTKREGLFGYLEDDPDSVVKMHLEGDDITARIYTREEQYTIEPYRSSDIKQN------CCPMLLTADYRFIYKTTEWPGYSGFQFQIKKIVIVQELLEVYSKEDHSAFCLAHLFTYQDFSNGVLGLAYIGTPSNAVGGICTSVYYAGNGKQYLNTGLTTTVNWGRRVLTEEADLVTAHELGHNFGSEHDEGDECAPGNSRGGNFLMYPASVTEPS------------------QTS-----------LCGNYRLEQGEQCDVGIDN--NPDECCT-ANCRLKPGKLCSDKNSVCCE-NCYYAPPSKVCSDATEQCKAKSYCTGRDIRCPAGCIDNGT--CV-GNCI-AFCEVNNFRSCICSPLEQSCYFCCVFEP-NGPCGKPCGGC-VQGHCEG-RTQDLIAR

>Spu2

LSYNTHLHAKHERSRRSV-DALQEVELDFEAHGRPFQLRLRQGAPYIGNLKDKPKSKVHGGITNGMFQGVIYDEDDEYHIEQYKASDVTHP------CCSLYLQADHTYIYIDTEFETIRDINFFIKRIR----------------VCTS-------------------------GGICEKSSNFQGVYQSLNTGVVTIQNYGSTVASKVSHITFAHELGHNFGSPHDYPERCRPGDRSDGNYIMYASATSGDKRFNDEFSDCSIANMTAVIREN-GGCFDRSDLPICGNLIVDGEEECDCGYEDQC-DDQCCTAATCMLTPNAQCRD-----------FEEQQP--------KSQSQVC------CPTEC-NEHTQVC-EGECQKSICTKFGLEECFCANAEEACHLCCQNPNSSATCGAPCGYCDVFYKCRNVDSNGPLSR

>Gga1

LSQSSIQHSLKKRDLQPE--THVERLLSFSALQRHFKLYLTATAEHFGHVVGEHNSKVVAHIGDEDFTVRINTDGEEYNIEPYRSEDIKDFSRLQSPLCKMLVVADHRFIYRNTPWEAFKGYGIQIEQIIIVKMLLEQFSFDEKAAVCLAHLFTYQDFDMGTLGLAYVGSPPNSHGGICPKAYHVKKD-IYLNSGLTSTKNYGKTILTKEADLVTTHELGHNFGAEHDSLPECAPTEDQGGKYVMYPIAVSGDHENNKMFSSCSKKSIHRTIEVKAQECFKERNNKVCGNSRVDEGEECDPGLYQ--RVDPCCS-ADCKLKDGAKCSDRNSPCCK-GCQFESAQKKCQEAINACKGESFCTGNSSECPTICVDMGK--CKDGECI-PFCERENLRSCACNETDNSCKVCCRDE--QDRCGKPCGFCDSNGKCEK-QVQDVIER

>Gga2

LSYDVDLHQKHQRAKRAVSHEDQFLRLDFHAHGRQFNLRMKRDTSLFGHIYGEQGSFSHGSVIDGRFEGFIQTHSGTFYVEPYHEDDIKYPHKYGPQGCQLYIQTDHLFIYRSTDFSGIRNISFMVKRIRIVEKFLELNSEQNHDDYCLAYVFTDRDFDDGVLGLAWVGAPSGSSGGICEKSKLYSDGKKSLNTGIITVQNYGSHVPPKVSHITFAHEVGHNFGSPHDSGMECTPGEKENGNYIMYARATSGDKLNNNKFSLCSIRNISQVLEKKRNNCFVESGQPICGNGLVEEGEQCDCGYSDQC-KDECCYDKKCKLKPGKSCSPSQGPCCTAQCNFKLKTDKCRNDSD-CAREGMCNGYSALCPTEC-NRRTQVCIKGQCTGSICEKYDLEECTCASSDELCHVCCMRKMDPDTCGSPCGYCDVFMRCRLVDADGPLAR

>Gga3

LSYDVDLHQKHQRAKRAVSHEDQFLRLDFHAHGRQFNLRMKRDTSLFGHIYGEQGSFSHGSVIDGRFEGFIQTHSGTFYVEPYHEDDIKYPHKYGPQGCQLYIQTDHLFIYRSTDFSGIRNISFMVKRIRIVEKFLELNSEQNHDDYCLAYVFTDRDFDDGVLGLAWVGAPSGSSGGICEKSKLYSDGKKSLNTGIITVQNYGSHVPPKVSHITFAHEVGHNFGSPHDSGMECTPGEKENGNYIMYARATSGDKLNNNKFSLCSIRNISQVLEKKRNNCFVESGQPICGNGLVEEGEQCDCGYSDQC-KDECCYDKKCKLKPGKSCSPSQGPCCTAQCNFKLKTDKCRNDSD-CAREGMCNGYSALCPTEC-NRRTQVCIKGQCTGSICEKYDLEECTCASSDELCHVCCMRKMDPDTCGSPCGYCDVFMRCRLVDADGPLAR

>Hsa1

--------------------------------------------------------------------------------------------------------------------------------IRIVKMLLEQFSFDEEASVCLAHLFTYQDFDMGTLGLAYVGSPANSHGGVCPKAYYVGKKNIYLNSGLTSTKNYGKTILTKEADLVTTHELGHNFGAEHDGLAECAPNEDQGGKYVMYPIAVSGDHENNKMFSNCSKQSIYKTIESKAQECFQERSNKVCGNSRVDEGEECDPGIYL--NNDTCCN-SDCTLKEGVQCSDRNSPCCK-NCQFETAQKKCQEAINACKGVSYCTGNSSECPTVCLDLGK--CKDGKCI-PFCEREQLESCACNETDNSCKVCCRDL--SGRCGKPCGFCDMNGKCEK-RVQDVIER

>Hsa2

LSLSNIQHSVRKRDLQTS--THVETLLTFSALKRHFKLYLTSSTERFGHVVGEPDSRVLAHIRDDDVIIRINTDGAEYNIEPYKSEDIKNVSRLQSPCCKLLVVADHRFIYRNTSWAGFKGYGIQIEQIRIVKMLLEQFSFDEEASVCLAHLFTYQDFDMGTLGLAYVGSPANSHGGVCPKAYYGKKN-IYLNSGLTSTKNYGKTILTKEADLVTTHELGHNFGAEHDGLAECAPNEDQGGKYVMYPIAVSGDHENNKMFSNCSKQSIYKTIESKAQECFQERSNKVCGNSRVDEGEECDPGIYL--NNDTCCN-SDCTLKEGVQCSDRNSPCCK-NCQFETAQKKCQEAINACKGVSYCTGNSSECPTVCLDLGK--CKDGKCI-PFCEREQLESCACNETDNSCKVCCRDL--SGRCGKPCGFCDMNGKCEK-RVQDVIER

>Hsa3

LSYNVDLHQKHQRAKRAVSHEDQFLRLDFHAHGRHFNLRMKRDTSLFGHIYGEEGSFSHGSVIDGRFEGFIQTRGGTFYVEPYHEDDINYPHKYGPQGCQLYIQTDHLFIYQTTDFSGIRNISFMVKRIRIVEKFLELNSEQNHDDYCLAYVFTDRDFDDGVLGLAWVGAPSGSSGGICEKSKLYSDGKKSLNTGIITVQNYGSHVPPKVSHITFAHEVGHNFGSPHDSGTECTPGEKENGNYIMYARATSGDKLNNNKFSLCSIRNISQVLEKKRNNCFVESGQPICGNGMVEQGEECDCGYSDQC-KDECCFDRKCKLKPGKQCS-------------------------------------------------TVCIQ--------------------------------------------------------------

>Hsa10

LSYNVDLHQKHQRAKRAVSHEDQFLRLDFHAHGRHFNLRMKRDTSLFGHIYGEEGSFSHGSVIDGRFEGFIQTRGGTFYVEPYHEDDINYPHKYGPQGCQLYIQTDHLFIYQTTDFSGIRNISFMVKRIRIVEKFLELNSEQNHDDYCLAYVFTDRDFDDGVLGLAWVGAPSGSSGGICEKSKLYSDGKKSLNTGIITVQNYGSHVPPKVSHITFAHEVGHNFGSPHDSGTECTPGEKENGNYIMYARATSGDKLNNNKFSLCSIRNISQVLEKKRNNCFVESGQPICGNGMVEQGEECDCGYSDQC-KDECCFDRKCKLKPGKQCSPSQGPCCTAQCAFKSKSEKCRDDSD-CAREGICNGFTALCPTDC-NRHTQVCINGQCAGSICEKYGLEECTCASSDELCHVCCMKKMDPSTCGSPCGYCDVFMRCRLVDADGPLAR

>Hsa17

LSLSNIQHSVRKRDLQTS--THVETLLTFSALKRHFKLYLTSSTERFGHVVGEPDSRVLAHIRDDDVIIRINTDGAEYNIEPYKSEDIKNVSRLQSPCCKLLVVADHRFIYRNTSWAGFKGYGIQIEQIRIVKMLLEQFSFDEEASVCLAHLFTYQDFDMGTLGLAYVGSPANSHGGVCPKAYYGKKN-IYLNSGLTSTKNYGKTILTKEADLVTTHELGHNFGAEHDGLAECAPNEDQGGKYVMYPIAVSGDHENNKMFSNCSKQSIYKTIESKAQECFQERSNKVCGNSRVDEGEECDPGIYL--NNDTCCN-SDCTLKEGVQCSDRNSPCCK-NCQFETAQKKCQEAINACKGVSYCTGNSSECPTVCLDLGK--CKDGKCI-PFCEREQLESCACNETDNSCKVCCRDL--SGRCGKPCGFCDMNGKCEK-RVQDVIER

>Xtr3

LSLSSIQHSLRKRDLQSQ--SQLERMLSFTALQRHFKLYLTSSTELVGHVVGEHNSKVLAHIGDGDFTARITTDGEEYNIEPYRSGDIKDFSRLKSSCCKMLVVADHRFIYRNTSWNQWKGYGVQIEQIIVVKQLLEQFSYDEKAAVCLAHLFTYQDFDMGTLGLAYVGSHPNTHGGICPKAYESKKA-VYLNTGLTSTKNYGKTILTKEADLVTTHELGHNFGSEHDSMEACAPSEDHGGKFVMYPIAVSGDHENNKMFSSCSRESILRTLMAKSPICFKERNNKVCGNSRVDEGEECDPGLHQ--HNDPCCT-SDCKFQPGVHCSDRNSPCCR-GCQFESAQKKCQEAINACKGESYCTGSSSECPTVCVDLGK--CVNGECR-PFCEIENLKSCACNDTENSCKVCCRDE--NGVCGKPCGFCDANGKCEK-QVQDVIER

>Xtr2

LSFDNHLDQKHKRSKRASEEEKEPIYLDFFAYKRKFALILRRDLNVFGALKDEFGSFCHGSIIDGHFEGFIQTKNGTFYVEYYHEEDIG--------LCLLYLKADYLFIYEQANFNGIKDINFKIKTLNLIPNILNTKTGK--------HLLINKAF-------QFTSMFPGNSGGICSKYSQNPNTFVTLNTGIVTIQKYGQYLPPRLIHITLAHELGHSLGAPHDESEECARFDSPNGNYLMFPYAMDGNQYNNDKFSSCSIYYIGNLLRVKKDQCFVESDRPTCGNQIVEEGEQCDVGYDN----DPCCYGLQCTLKPGKQCSPSQGLCCSHLCSYMPKSQRCQDEAE-CTLENNCTGDSAKCPTLC-NLGTRICINGMCRQSVCAKFGLEQCDCDSESEKCQLCCQEPDNVYSCGSPCGYCDKFHICRLVDADGPIAR

>Xtr1

LNYNMDLHQKHQRAKRAVSQEDQFVHLDFQAHGRQFNLRMKRDTSLFGQLFGEQGTLSHGSVVDGRFEGFIKTHQGTFYLEPYHEDDIKYPHKYGSEGCQLYIQTDHLFIYQSTDFSGIRNISFMVKRIRIVEKFLELNSEQNHDDYCLAYVFTDRDFDDGVLGLAWVGAPSGSSGGICERNKLYSDGKKSLNTGIITVQNYGSHVPPKVSHITFAHEVGHNFGSPHDSGNECTPGEKENGNFIMYARATSGDKLNNNKFSICSVRNISQVLDKKRNSCFVESGQPICGNGLVEQGEQCDCGYSDQC-KDDCCYDLKCTLKPGKQCSPSQGPCCNSGCTFKLRSEKCREESD-CAKMGTCNGVSAQCPTEC-NRATQVCIKGQCSGSICEKYDLEECTCGSSDELCHVCCMEKMKPHTCGSPCGYCDVFMRCRLVDADGPLAR

>Dre1

LPASGLLHSVRKRDADAH--SHVERLVSFTALQRNFKLYLTTNTQLFGHVVGEEHSRVQAHIDGDEFSAHIITEETEYNVEPYRSEDIKNISRLSSPVCTLLLVADHRFIYRNTSWDDFKGYGVQIQQIIIVKKLLEQFSYDANASACLAHLFTYQDFDDGTLGLAYVAPSKPGLGGLCPKPYYSVKKPSYLNTGLTSTMNYGKTILTKEADLVTTHELGHNFGAEHDNIASCAPSDDQGGKYVMYPIAVSGDHVNNKHFSTCSKISVSKTLRIKVNQCFVERSSKLCGNSRVEEDEDCDPGLHI--NDDPCCT-SNCKFRKQAQCSDRNSPCCK-NCMFESADKVCQEVITACKGTSQCTGNSSECPTECVDKGR--CQNGQCR-PFCEAVNLESCACNETENSCKVCCRKDEL---CGKPCGFCDGAGKCMK-QVQDVIER

>Dre2

LQLSSLQHSVRKRDVQSQ--THAERLLGFTALQRHFKLYLTTNTELFGHVIGEENSRVQAHIGDNDFTAHILTDEAEYNIEPYRSEDIRNVSRLAATCCPLLLVADYRFIYRNTSWDEYKGYGVQIQQIIIVKKLLEQFSIDDNASVCLAHLFTYQDFDEGTLGLAYVA-PPGFPGGLCSEKCPNDNRAIYLNTGLTSTKNYGKTILTKEADLVTTHELGHNFGAVHDDMSYCAPREDQGGKYVMYPIAVSGDHTNNKLFSNCSKMSIAKRLRAKASTCFKERNSNVCGNSRVEEGEECDPGLHL--NSDLCCT-ANCKLKPGVQCSDRNSPCCK-DCRFEKQNKVCQEPMEACKGRSNCSGYGSECPTICVDNGR--CLNGECI-PFCEAVNLQSCACNETHNSCKVCCKDK--NGVCGKPCGFCDGAGKCMK-QVQDVIER

>Dre3

LSYDREQHQHRIRRDANP--NKQDLHLDFSAFQREFHLRLTPVNNGFGVLEDDSKSSCQGSVLEGQFEGSITTSNGTFYVEPYHEDDVDDTPLRSGHCCLLHLHTDHRYVYDKANFDGIELINFKVKFLTLPEKLLSLFSETNWNDFCLSYLLTDRDF-SGVLGLAWEGKPGYNWGGICSKMILKSGRNCSHNTGLVTLQTYGHYLSTKHVHLTFAHELGHSLGAPHDENSNCGDLESGKGRFLMFPKAASRIEENSDKFSPCSLRHMSHLLNVK----------------------------------------------KDT-----------------------------------------------------------------------------------------------------------------------

>Dre4

LSYDREVKQHQHRIRRDANPNKQDLHLDFSAFQREFHLRLTPVNNGFGVLEDDSKSSCQGSVLEGQFEGSITTSNGTFYVEPYHEDDVDDTPLRSGHCCLLHLHTDHRYVYDKANFDGIELINFKVKFLTLPEKLLSLFSETNWNDFCLSYLLTDRDF-SGVLGLAWEGKPGYNWGGICSKMILKSGRNCSHNTGLVTLQTYGHYLSTKHVHLTFAHELGHSLGAPHDENSNCGDLESGKGRFLMFPKAASRIEENSDKFSPCSLRHMSHLLNVKKDTCFVDSDQPICGNRIVEEGEECDVGHDS----DPCCHSIECRLKLGKQCSPSQGLCCNSQCVFKKAGLMCEGNSE-CRNKSVCAGSSAVCPTIC-SNGTRVC-SGECGLSLCALHNMVQCDCPGQSEKCHMCCQQRDKPNTCGAPCGYCDQFKVCRILDADGPIAR

>Dre5

LSYDTELHSDHQRAKRAATHTERTLQLEFHAHGRHFNLRMRRDTGLFGEIYGEQGTLTHGSVQDGRFEGFILTHRGTFYVEPYHEDDIDYPHKYGPAGCQLFIQTDHLFIYQGTDFMGIRNISFMVKRIRIVEKFLELNSEQNHDDYCLAYVFTDRDFDDGVLGLAWVGAPAGSSGGICEKNKLYSDGKKSLNTGIITVQNYASHVPPKVSHITFAHEVGHNFGSPHDSGSECTPGEKERGNYIMYARATSGDKLNNNKFSVCSIKNISQVLEKKRGSCFVESGQPICGNGLVEPGEQCDCGYSDQC-KDDCCYDKKCKLKPGKVCSPSQGPCCTFECSYKGHNEKCREESE-CAHQGLCSGSSAQCPTAC-HGNTQVCLNGGCSGSICEKYGLEVCTCASVEELCHVCCMEKMNPSTCGSPCGYCDVFMKCRLVDADGPLAR

>Dre6

LSYDTDLHSKHQRAKRALSHQDQFIHLEFHAHGRHFNLRMKRDTSLFGEIYGEKDSLTHGSVVDGKFEGFIKSHHGTYYVEPYHEDDIDYPHKYGSEGCQLFIQTDHLFIYQGTDFQGIRNISFMVKRIKIVEKFLELNSEQNHDEYCLAYVFTDRDFDDGVLGLAWVGAPSGSSGGICEKNKQYSDGKKSLNTGIITVQNYASHVPPKVSHITFAHEVGHNFGSPHDSGSDCTPGEKEKGNYIMYARATSGDKFNNNKFSICSVRNISQVLDKKRSACFVESGQPICGNGLVETNEECDCGYNDQC-KDQCCYSKKCKLKPETFCSPSQGPCCTPQCTYRTGNE-CRPESD-CAFKGLCNGLSAQCPTAC-HANTQVCINGGCSGSICEKHKLDVCTCATEDELCHVCCMEKGKPSTCGSPCGYCDVFMKCRLVDADGPLAR

>Afua

HSVRTSSETRLENIIIHTVDAYSPFELSFSLPGQALKLSLEPNHDILGVVVGWARIYIHRDGVDPLFDGIFGVAGQQYQVTIHYHSSYQTWDRLAPRNALVGIATDCTYVYERT-----FNISLALHDLTILNWRLNRFSWRSLNDNAYWTLMTGCP-SGQEVGVSWIG-------QLCSSNRA------RQSAGANVVAR------SRSEWQVFAHESGHTFGAVHDAQSQCCPLSDANGQYIMNPVSTA----SQTAFSPCTIRNICSQLSSGSTRCLVSNSNTQCGNGIVEVGEECDCGA--TCDQNSCCDGSTCRLRAGALCDDAASPCCT-NCQFASADTVCRPSTGPCDVEEMCTGNSTICPQRCGGEGG----S-----------GNPSC------------------------------SNGECRRNET------

>Afub

LEHAVI--------NTPSHLTDFDVTFELPDKHQTIKLELEPNHDILGRSNGWRVGWARVYVQRGLFEGVFSIDNDNHHVELYRDSDMIRQVRSELKLKQVAIAADCSFVYEKS-----FNISIGLRNLTMITQRLNLFQWRQQSDNAYWTLMSNCP-TGSEVGLAWLG-------QLCNTEVT--------GDGSNSV--SGTNRVSGGGWQVFAHESGHTFGAVHDASSQCCPYNDANGKYIMNPST----GADITAFSPCTIGNICSALGRNKSSCLSDNRNVQCGNGIVEAGEDCDCGGSS-CGDNPCCDAKTCKFKSGAVCDDANDSCCS-QCQFSPAGTVCRASLGECDLQETCTGNSSTCPEKCGDTSGLTCASQQCRTVMGSLIDNNTYACPQFDSSCELICTSPS-LGSCGTPCGYC-HNGRCDG---------

>Spo

--------------------------------MR--------LVLLF-------AEIILAHSDE-NLLSRTKNLS-------Y--------------YLWIGVVADCSYLYEDS-----FNINVQIHSLILIEEKLEIFKWKPGNNFEAISPHERESFSEPQVSVLFTSSPVSWFATICSETHI-----NEWHVGPLSVVSYPND------RLVVAHEIGHILGLIHDHSEACCPLSDAQELYIMNP--SNSYTYANLRFSDCSILQLHSLVEKKSLSCLSKPSESTCGNGIVEDGEECDCGE--DCENNPCCDGKTCKLTKGSLCDDQQDACCY-QCHFKNAGTLCRQSTNPCDKPEFCTGISSKCPRICQSLGMGSCASVQCKKLT----NFSSLSCHS--DSCKVSCQNE--DGTCGTRCGLC-YNGVCVPIEGSSA---

>Uma

LQRIARKVERIPRSEHLHSDTGSSLRLSFRAFQQDFHLHLQPEHMLHDHDLGVVRAAIMLHDDGVSFSGSFDWFGNVHYIETHRDSDITLEPRDDTFCARVVVASDCAYLYRST-----FNVSIGVVELDVIDERLSDFRWRQPGNIGLWHLMTACN-SGREIGVAWLG-------TVCMTDAS--------SSSGQTVSGTGVSSLTTQQWQVMAHEMGHNFGAIHDGGAPCCPLSNANAQYIMNPSSSS----NIQSFSQCSIGNICSLLGQGDTSCIQTPGQSQCGNGILEPGEECDAGP----NGSQCCT-SQCRLASGAQCDPATSACCSNSCTFAPSSQMCRPAVDECDSAEYCTGTSAECPSSCG--SRLSCANYQCQQASTGQLNFQTA-CSASNNSCQLTCQSPNSANSCGTSCGRC-KGGECKSGSWQNTFRS

>Hsa11

LVRESSKQQLDTRVRQEPPVHLAQVSFVIPAFNSNFTLDLELNHHLLGKLRGNPHSFAALSTCQ-GLHGVFSDGNLTYIVEPYRTPLLPDPLGCREPCVELIVINDHQLIYKEQ-----LNTRIVLVAMETLLETLARLVYRGLPESDATHLFSGRTFQSTSSGAAYVG-------GICSLSHG-------------GGVNYGNM---GAMAVTLAQTLGQNLGMMWNSAGDCKCPD-----IWLGCIMEDTGFYLPRKFSRCSIDEYNQFLQEGGGSCLFNK--PECGNGFVEAGEECDCGSVQECAGGNCC--KKCTLTHDAMCS--DGLCCR-RCKYEPRGVSCREAVNECDIAETCTGDSSQCPYYCDEQGR--CYGRQCQVLWGHAAADRFCQCSKQDLCGFLLCVNISGLVGDGTACGVCSNEGKCICQPDWTGKDC

>Hsa30

GEFDSYEKLSFRGEVQGV---VSPVSYLLQLKGKKHVLHLWPKRLLLGSV--ESDSKATISTCMGGLRGVFNIDAKHYQIEPYLLKKEQFGNQ----CLELILLFDQSRYFQDVRM------RIHLKALEVLAEVLGRFIYKNARLSDWAHLYLQRKYNDA---LAWSF------GKVCSLEYA---------GSVSTLLD----TNILAPATWSAHELGHAVGMSHD-EQYCQCRG--RLNCIM--------GSGRTGFSNCSYISFFKHISSG-ATCLNNIPGGRCGNKIVEDNEECDCGSTEECQKDRCCQ-SNCKLQPGANCS--IGLCCH-DCRFRPSGYVCRQEGNECDLAEYCDGNSSSCPTPCKYEGR--CFRGQCQSIFGPDAAPSECKCESANICGRLQCINVETEHTTGTSCGVCNNRKNCHCMYGWAPPFC

>Hsa29

PP----IPVRITGTTRGM-TPPGWLSYILPFGGQKHIIHIKVKKLLFGYVEGDPESLVSLSTCFGGFQGILQINDFAYEIKPYKMDSEEKQFSTMRSGVEIVVVIDNYLILD------VIGVKVLLFGLEIVRKSVHLYKWKQHDT---SHLFT----TLGLRGLSGIG----AFRGMCTPHRS---------CAIVTFMN----KTLGTFSIAVAHHLGHNLGMNHD-EDTCRCSQ---PRCIMHE-----GNPPITKFSNCSYGDFWEYTVER-TKCLLETVHTRCGNGVVEEGEECDCGPKH-CAKDPCCL-SNCTLTDGSTCA--FGLCCK-DCKFLPSGKVCRKEVNECDLPEWCNGTSHKCPIPCKERGY--CYESQCRRIFGAGAASETCKCNISDQCGRIQCENVTEDHTTGTECGICNNKHHCHCNYLWDPPNC

>Hsa25

PEVVIPLQVTGNRPMWAM----GWLTYSLHFGGQKHFIYMKAKKFLVGYMDGDPEFMVAITTCSGGFQGILQVNGTVYEIKPHKIDREGTELLPMR-CLDLALVIDRERIYQTLDL------ELVLLGVELIDVLLYEFIWKNFRINDIAHLFVNHHF-GIYLGLAYVG-------TVCM---------PSLNCGIDRLIG----HNLFYFGHIIAHEMGHNLGMQHD-EGSCTCGA---KDCLMAP-----TDSGIQKFSNCSYSAFWTTYATA--NCMRKETKPRCGNGVVDDGEQCDCGSAEMCARDPCCK-SSCTLKDGATCA--FGLCCT-HCRVMPSGTVCREQVNECDLPEWCNGHSYKCPSPCRGGGY--CYERQCKQIFGQEAAAQSCRCHDSDLCGRLQCENVTGDHSTGTDCGVCNNLHHCHCDLGWDPPYC

>Hsa1

SARLASEEIVFPEKLNGSSGAPARLLCRLQAFGETLLLELEQDSGVQGTINGDPESVASLHWDGGALLGVLQYRGAELHLQPLRRKSPASGQGPM--CVETLVVADDKMAFKHPSI--RNPVSLVVTRLVIAAQTLRSFAWQSDPDFDTAILFTRQDL-CGVSTCDTLG--MADVGTVCDPARS---------CAIVEDDG-------LQSAFTAAHELGHVFNMLHDNSKPCISLNLSTSRHVMAPVMAHVDPEEP--WSPCSARFITDFLDNGYGHCLLDKPEAFPGKDYDAD-RQCQLTFPDHCLPPPCAALKHSPWADGTPCGP--AQACMGRCLHMDQLQFNIPQAGGCSRT--CGQFSSRCTKYCERRTRFRSCNEDCPTGSALTFREEQCTGVAPQDQCKLTCQAQ-----AGTPCGSKKKFDKCMVCGGDGSGCS

>Hsa9

------------------------------------------------------HPLLLATVLD---SGLLHLENASYGIEPYRMDDVY--------CVELFIVVDKERMY------IMLNIRIVLVGLEIAGDVLGNFQWRRHDS---AQLVLKKGF-GGTAGMAFVG-------TVCSRSHA----------GGINV--FGQ-ITVETFASIVAHELGHNLGMNHDDGRDCSCGA---KSCIMNS-----GASGSRNFSSCSAEDFEKLTLNKGGNCLLNIPKPSCGNKLVDAGEECDCGTPKECELDPCCEGSTCKLKSFAECA--YGDCCK-DCRFLPGGTLCRGKTSECDVPEYCNGSSQFCQYPCQNKAY--CYNMQCQVIFGSKAAPKDCFIEVNS--------------------G--DRFGNC-----------

>Hsa8

VLPRRL---PGPRVRRALGLHPERVSYVLGATGHNFTLHLRKNRDLLGHVEGYPDSAASLSTCA-GLRGFFQVGSDLHLIEPYQAEHLL--------CVELYVVVDNAELYQKLNF------RVVLVGLEIPSVTLENLTWQLHDN---VQLITGVDFTGTTVGFARVS-------AMCSHSSG----------AVNQDHS----KNPVGVACTMAHEMGHNLGMDHDNVQGCRCQEFEAGRCIM---AGSIGSSFPRMFSDCSQAYLESFLERPQSVCLANAPDLVCGNLFVERGEQCDCGPPEDC-RNRCCNSTTCQLAEGAQCA--HGTCCQ-ECKVKPAGELCRPKKDMCDLEEFCDGRHPECPTPC-SGGY--CYNAQCQAFWGPGGAEESCGCKASRMCGVLQCKGGQQLGRAGTRCGVCNHKQECHCHAGWAPPHC

>Hsa2

LRMDSNVQITVPEKIRSIEGIESQASYKIVIEGKPYTVNLMQ-KNFLGYIEGYPKSVVMVSTCT-GLRGVLQFENVSYGIEPYQVKHKK--------SIEMHVIVEKQLIFVSFNI------TIILSSLELANELLHTFRWKLVLRHDVAFLLVYREK------SNYVG--ATFQGKMCDANYA---------GGVVL---HPRTISLESLAVILAQLLSLSMGITYDDINKCQCSG---AVCIMNPEAI--HFSGVKIFSNCSFEDFAHFISKQKSQCLHNQPRDVCGNAKLEAGEECDCGTEQDCIGETCCDIATCRFKAGSNCA--EGPCCE-NCLFMSKERMCRPSFEECDLPEYCNGSSASCPHPC-GLNQWICIDVQCTDTFGKEVGPSECQCEADNQCGKLICKYVGKPRATGTSCGVCNNKKHCHCSASYLPPDC

>Hsa19

GSPKLQHELIIPQWKTSEEKHPLKAELRVMAEGRELILDLEKNEQLFGTV--ETLSSVTLSTCR-GIRGLITVSNLSYVIEPYRSEHLKPP------CVELYLVADYLEFYRSLNI------RIALVGLEV------SFSWRLAQKHDNAQLITGMSFHGTTIGLAPLM-------AMCS---------VYQSGGVNMDHS----ENAIGVAATMAHEMGHNFGMTHDSADCCSASA-ADGGCIM---AAATGHPFPKVFNGCNRRELDRYLQSGGGMCLSNMPDTRCGNGYLEDGEECDCGEEEEC-NNPCCNASNCTLRPGAECA--HGSCCH-QCKLLAPGTLCREQARQCDLPEFCTGKSPHCPTPC-EGGQAYCYNMQCQQLWGPGAAPDLCKCNMRDKCGKIQCQSSEAESNAGTKCGVCNNNQNCHCLPGWAPPFC

**APH1**

>Vca

MTI-DFFGLIFLGLGPGIAFFLVVLARKSFLVLLSLFS----AFLWLVVLLIISAIFYAGLLIASVAIEEAVRYGVWRLHKTLEPLALAWGYGHSASHAVFFFLSFLPLTVSD-GTYYNPAIFLVGALYSLSFGLILTSLMVIAFDGAPLLHLGASCLTLLNCVVSMPVLVGLGLLLVLYTIGISWR-

>Ath

MTVAAGIGYALVALGPSLSLFVSVISRKPFLILTVLSS----TLLWLVSLIILSGLWYALLVITSVCFQEGLRFLFWKVYRLEDVLALAGGLGHGVAHAVFFCLSLLTPAFGP-ATFYVERFFLISAIIALAFVTIHTFSMVIAFEGVPVIHLTAGMLTLVNCVIGVPLLYLVASLTLVHCGKMVWQR

>Pra

VNWPLLFGCILVGFGPLAALFFVVVAKRAQLVILALSG----AFVWLVAILVTASLWLSATVPVGVVVQEAFRLLFFHLYRTEAAVSLAGGVGFSVMHALLMFGSLVGSSTGSRGAAFSASLVFSAAISTLALTALDVALMVVAFDGVFVIHMGVALSALANCYISIPVHYAGAALASAGAMTIVW--

>Cel

MGYLLTIACYIASFSPSIALFCSFIAHDPVRIILFFLG----SFFWLVSLLFSSLAWFLLSLTVCIIAQELSRVAYFMLLKAQRGLALVCGLGMGVISALFYTMNAFAIFSGP-GTIGLPNLPLCYTLSAILLTLFHVTWTIMVWDSAVVSHLLVTFLSSLNHVLVFAVQFLILLICIAYCNVIMGGT

>Ddi

MTQVLFYGCLFITFSPILAFFFMVIAKNSQLVILTIGG----SFFWLVSILIAAIWWWWFIISFSVLFQEIFRYIFFRLYKQAQQQSAAIGVGSGVAYGFIMFGSILWESTGP-GTLFSPALFMLSSIITLFMTLLHVVYNVLAFQGVIITHFVTTYLTLLNCVGSILPIGIITVFSVGFCIFSLLKS

>Mbr

-----------------------------------------------------------FALIASVLLQELFRYLWWRLLRTEHDLAMVSGLGYGLMSSTMMTCNLLDIMSGP-GILAARGHFTISSTTAAMIGLTHVFWGIIAFSGVFFTHLFVSLLTLNNCAGTMVPIALVMIASGIFAWFAAG--

>Hro

MSMVEFLGCFLITIGPVLVLFFMTIAHDPILVILCIARLANFAFFWMLSLLVSSAWWVGFGVVFAVLFQEMFRFLLFKLMKAEHGLAYVAGLGFGSIHCIFNMVNLLAAMFGP-GTAGLRGFFITSSFLSLGFLLLNILWGIIFFHGVLISHFMVSACSILSVAWSIMLTLAMVISMAFYTYKVLGGS

>Aqu

MTVLTAFAYGIYTFGPPVALFFVTVARHPHEIITMILG----AFFWLLALLFASLVWPAFTLPISVILQEVFRWLYFKLLKADHLLAYVGGLGFGLIAGIVMFANVLSVASGP-GTV---RFVTVSAFSTQVMIILHICWGVIFFAGVPISHMFISCLSLLIYFLSF--GYFLCVVFVALAFFAAGAR

>Tad

A-FMSWFGYTFIAFGPSLAIFTMTIADDATQIIIMFTS----AFFWLVSLLFSSIIWLGISVALCVAIQELFRYLFYLLMKADKGLAYVAGFGFGVMSGAFSVVNLLATASGP-GTVGIKGFFLSSAIFTSIFILLNIFWSVIIFDGVFATHELTSLLTLVNYSASITTSCLVLLITTIVALYTAGGN

>Cin

MPLMVFFGCTMIAFGPSLAMFILTIAKYPLKIILLVVG----AFCWLLALLFSALLWLAFSLVFSVIFQEGMRYGLFRMMNAENGLSYTTGFGFGLMYGLFSIVNVLSQSIGP-GSVGIDGFMLISAFLTSSTILLHTFWNVIVFDAVVCLHMLVSCCSLLNAYVSVIVSYIVLIGTTVWAFKAAGGS

>Aae

MTVVEFFGCSFLAFGPPLAMFSLTIAHDPIRIIILIAA----SFFWLVSLLFSSTVWIAFGLICSVFIQEAFRYLMYKLLKTERGLSYVCGLGFGIISGAFSLVNILADSVGP-ATVGLKAFIVISAAQSLCMILLHTFWSVIFFNAVVVSHLFVSCITLLNFAVSLTASYIVMLVTSVIAFRVVGGN

>Hma

MTLESFFGCAFVAFGPALSMFAITVFNDAQQVLVLISS----AFFWLLSLLVSAMWWLVFSMIFSVIFQEMFRLFLWMVLRAEEGLHYVSGLGYGLMSGLFAMVNILADITGP-GTVGLFGFVIVSAFLTNCFVLLHTCWGILFFDAVCTSHLAISLLTLLNYWPSLITGYILLVVMGIWSFRVVGGS

>Nve1

MTLMVFFGCTFIAFGPALAMFSLTIAGDAQQVIVLIAS----AFFWLLSLLISSIWWLAFGMTFSVLFQELFRFAYFKIIKADEGLAYVAGLGYGLMSGLFAMVNVLADISGP-GSIGLHGFLIVSAFLTSCFVLLNTFWGVIWFDGVVASHLFVSLMTLLNYIATLLSAYFTLVVMAVLAFKVAGGS

>Nve2

-----------------------------------------------------------------------------------------AGLGYGLMSGLFAMVNVLADISGP-GSIGLHGFLIVSAFLTSCFVLLNTFWGVIWFDGVVASHLFVSLMVSLRH-----------------EFSVVHGL

>Spu

MTLLQLFGSMFIAYGPAFSLFLFTISREPLRIIVMMAG----MFFWLCSLLTSSVLWISFGLTFSILFQELFRFIYYKILKAEDGLAYVAGLGFGTMSGIFSFINVLADSKGP-GTVGIHGFLITSAFLTSCFILLHIMWNVIFYWGVVASHFLVSELTLLNYSITLPVAYVTLAVMTLFAFFIAGGS

>Lgi

MTLMEFFGCAFIAFGPPFAMFIFTIARDPLRIIVLIAS----GFFWLLSLLLSSILWLAFGLVFSVIFQELFRFLFYKLLKADEGLAYVSGLGYGILSGAFSIVNVLADMAGP-GTIGIKGFFLTSAFLTLCFILLHTFWGVIFFNAVVASHMLVSCLTLFNYLISLITAYVVMIVMAFFAFITAGGS

>Bfl

MTVKEFFGCTFIAYGPAFALFIFTIAREPLRIIILIAG----CFFWLLSLLFSSVLWLAFGVVFSVLFQEIFRFFIYKLLKADEGLAYVAGLGFGLMSGAFSLVNVLAMATGP-GTVGIHGFFLTSALMTSCFILLHTFWNVIFFWGVVGFHMLISCLTLIRQPATIIPAYCILLVTMAIAYKLVGGS

>Hsa1

MTAAVFFGCAFIAFGPALALYVFTIATEPLRIIFLIAG------------------------------------------RVETCK---------------------PDCASPSGSL----FLLVGVSTDFVPCLVH---------------------------------------------------

>Hsa2

MTAAVFFGCAFIAFGPALALYVFTIATEPLRIIFLIAG----AFFWLVSLLISSLVWLIFGAFVSVYIQEMFRFAYYKLLKASEGL--------------------------P-GE-----RLLAYAFMTLVIILLHVFWGIVFFDGVLLTHLLVSAQTFISYGINLASAFIILVLMGTWAFLAAGGS

>Hsa3

MTAAVFFGCAFIAFGPALALYVFTIATEPLRIIFLIAG----AFFWLVSLLISSLVWLIFGAFVSVYIQEMFRFAYYKLLKASEGLAYVSGLGFGIMSGVFSFVNTLSDSLGP-GTVGIHGFFLYSAFMTLVIILLHVFWGIVFFDGVLLTHLLVSAQTFISYGINLASAFIILVLMGTWAFLAAGGS

>Hsa4

MGAAVFFGCTFVAFGPAFALFLITVAGDPLRVIILVAG----AFFWLVSLLLASVVWLIFGAAVSVLLQEVFRFAYYKLLKADEGLAYVSGLSFGIISGVFSVINILADALGP-GVVGIHGYFLTSAFLTAAIILLHTFWGVVFFDAVVGSHLLTSGLTFLNYEASLLPIYAVTVSMGLWAFITAGGS

>Dre1

MTVAVFFGCTFIAFGPAIALFMFTIARDPLRVIFLIAG----AFFWLVSLLLSSLVWLIFGVVLSVLLQEAFRYGYYRLLKANEGLAYVSGLGFGFMSGAFSVVNILSDSLGP-GTVGIHGYFISSAFMTLAIILLHMFWGVVFFEAVVASHLVVSCLTFVNYQGSLIPTYIILSVMAVWAYLCAGGS

>Xtr

MALAVFFGCTFVAFGPALSLFILTIAVDPLKVIILVAG----SFFWLVSVLLSSLIWLIFGAAVSVLLQETFRYAYYRLLKADEGLAYVSGFSFGIISGVFSVINILADAIGP-GIVGVHGYFLTSAFLTMAIVFLHTFWGIVFFAAVVLSHLVASGLTFLNYEASLIPIYIITLGMALWAFVAAGGN

>Dre2

MTLAVFFGCAFIAFGPAFALFVFTVAKDPLRVIILIAG----AFFWLLSLLLSSLVWLIFGVFFSVLLQEVFRFAYYRLLKATEGLAYVAGLGFGVMRGAFSMINILSDSLGP-GTVGIFGYFITAALMTLALTLLHTFWGVLFFEGVVSLHLLVAGLSLLNYEGSLPPVYCITLLMGIWAFFSSGGS

>Gga

MTLAVFFGCTFIAFGPAFSLFLFTIARDPLRIIILIAG----AFFWLVSLLLSSLIWLIFGVMFSVLLQEAFRFLYYKLLKAIEGLAYVAGVGFGLMSGAFSMINLLADALGP-GTVGIHGYFLTSAFMTMVLIFLHTFWGILFFHGVVVMHLAVSGSTFCNYV------------------------

**DELTEX**

>Mbr

VPTAALKGTAASAPP------DIEEADSGGGGGIK-NQSFR-CYVCSAVHGKP-LT--GNQPP-GEMRVHLS-QQPLPGFDSV-GHYIIDYEFPPGLQGPEHPTPGAYFN--GDRRRAYLPATD---KGLETLKR---LKTAFERRLTFTIGLS--------LTLGPRAGERIWNGIHHKT------SLSGG--AFGFPDPTYFERVDEELRAGI

>Aae

CPMCMEELLSRCQHLMHLN--CLNELILGQQKENQKSLYIE-CPICMSVYGEK--I--GDQPP-GTMSWIVI-PRSLPGHEGQ-NTIQITYNIASGLQSKEHPHPGRAFFAVGFPRTCFLPDGL---LGRKILRY---LKIAFDRRLLFSIGRS--------ATTG--REDVVWNGVEHKT------QYSM------FPDPQYMQRCMQQLVHGV

>Tad

CIICFEEVLSQCKHYFHDE--CIFACV---QSKT--SEFLE-CPKCRTLHGIK--T--GNQPE-GTMTYVVQNGMQIPGFNST-SAIVITYNFRSGIQGPQHPNPGARYHANAFPRVAYLPNTA---KGQEVLRL---LQIAWARSLIFAIGRS--------STSG--ADNVIWNDIHHKT------EWQSNYSGHGYPDPDYIKRVTEELHAGI

>Hro1

CCICCDSLLDKCRHHFHLL--CLKAMY---EAGTK-HGSLQ-CPHCKHIYGVK--V--GNCPA-GRMVCVTI-PQSLPGYEQY-DTLYITYNILSGVQEPGHPNPGKKYSARGFPRCGYLPNNK---KGQKVLRL---LVKAWNRRLTFTIGTS--------VTTG--ESNTVWNEIHHKT------EML-NKHGHGYPDANYLDNVISELAAGV

>Hro2

CCICCDSLLHKCSHVFHYN--CLLAMY---NSGKK-EGSIQ-CPSCKYIYGIK--Y--GDCPS-GSMSYEILNNCSLSGYPDC-GVIKITYDIKNGIQESFHPNPGKQYTATGFPRYGYLPHNL---DGLKILQL---LKKAWFRRLTFTIGTS--------VTTG--RANVVWNEIHHKT------EMD-NKSGHGYPDPEYLNNVTKELTFGV

>Bfl1

CPICCENLLQHCNHVFHGV--CLLAMY---NSGSK-DGSLQ-CPTCKTIYGEK--C--GNQPP-GTMDYHVI-PYPLPGYVDC-HTIRIIYTIPHGIQGPEHPTPGKKYTARGFPRLCYLPDTE---KGRKVLQL---LIKAWERRLIFTIGRS--------TTTG--EDNTVWNEIHHKT------EFGSNVTGHGYPDPEYLDNVIAELAAGV

>Bfl2

CPICCENLLQHCNHVFHGV--CLLAMY---NSGSK-DGSLQ-CPSCKTIYGDK--C--GNQPP-------------TP--------------YHHGIQGPEHPTPGKKYTARGFPRLCYLPDTE---KGRKVLQL---LIKAWERRLIFTIGRS--------TTTG--EDNTVWNEIHHKT------EFGSNVTGHGYPDPEYLDNVIAELAAGV

>Amq

CPICMNNLLRKCGHMLHRS--CLITYS---KNSTDAKGCLR-CPTCKVIHGVK--R--GDMPSTGTMTVSKK-RYSLPGHPRC-GTIEIVYSFQPGVQNGVH------YRANGFPRTCYLPDST---KGQKVLQL---LRVAWERRLTFTIGTS--------VTTG--ATNTIWNEIHHKT------ESTSNHSGHGYPDENYLDNVLAELAAGV

>Lgi2

CGICLDEILDKCGHKFCQG--CVKHAF---VCKPV-------CPECGMIYGVI--I--GDQPRGGKMIHKLITSHKLEGYKDADGILEIIYTFNDGIQQDNHPNPGQPYS--GLRRKAYLPNNK---EGQEILQM---LYKAFDARLIFTIGES--------RTTG--QTGVLWNDIHHKT------SITGGALSHGYPDLGYLKRVREELALGI

>Lgi1

KVMSSDKMISYHNVKDTEG--KILGLS---ETEALRDLHKPVCPSCGTVYGVT--I--GNMPA-GTMNVHKS-SLNIEGYKRF-GSYCINYDFNSGIQGSKHPNAGVRFK--GTSRVAYLPANP---EGIEVCKM---LKVAFRRRLTFTIGRS--------TTTG--QENVVWNDIHHKT------SMNGGPTNFGYPDATYLSRIKEELASGV

>Spu

CPICFEKLLDRCRHFFHQS--CLYAMY---NSGPK-DGSIQ-CPTCKAIYGVK--C--GNQPP-GSMDYHVI-PHSLPGFPSC-GTIRIIYNIPPGTQGPEHPSPGRRYSSRGFPRMCYLPDNE---ISRKILRL---LIIAWERRLIFTIGTS--------VTTG--EPNTVWNEIHHKT------EFGSNVTGHGFPDPNYFTNILGELASGV

>Cin1

CCICLDDFELKCKHKFHVH--CIEQAL---KQKSF-------CPMCNTPVGAP--C--GNQPLGGRMTWSMNNRIHLPGYDNF-GAIVIRYSFSGGTQQHNHPHPGQRYS--GTTRIAYLPDSH---EGQEVLAL---LTRAFNARLIFTVGRS--------ITSG--RDNQIWNDIHHKT------RTHGGAHGYGYPDPEYLKRVKEDLRAGI

>Cin2

CAICLDDITLPCKHKFHET--CVNQAL---KVNNL-------CPICKQAVGKV--K--GNQPN-GTMRDRIDHNTHLPGYERY-GAIVIDYNFPSGIQGREHPNPGVQYT--GTSRTAYLPDNG---EGREVLRL---LRKAFDARLIFTIGRS--------VTTG--MENQVWNDIHHKT------NPYGGATGFGYPDPDYLNRVKDELKAGI

>Cin3

CVICMTSLLKTCKHIFHAE--CLIASM---QSGA--NDSFT-CPCCKVIYGVK--T--GNQPP-GQMHWSVI-QTSVPGYEPH-HTIRITYDIRAGVQTNEHPNPGRRYSVHGFPRIAYLPDNT---DGNKVLRL---LEVAWNRKLIFTVGDS--------VTSG--AKDTVWNEIHHKT------SIT-NKHGHGYPDPNYLTNVLNELKAGV

>Cin4

CVICQDT-VLNCNHKFHEA--CINRAL---AVKPV-------CPCCNTCVGKP--Q--GVVLE-GATMMHMIEKTSLPGYEGC-NTICIVYNIPDGTQGAQHPHPGKTYH--GTNRVAYLPDNE---DGRHVLGL---LSQAFVAGLVFVVGRS--------LTTG--LDDSIWNDIHHKT------HHTGA---HGYPDAGYLQRVKEDLRAGI

>Nve1

-----------CGHTFCRP--CITEAS---KSSKL-------CPTCRDPFGVQ--Q--GNMPW-GTMNWKIDHYKHLPGYERW-GTIVIFYYFPGGTQDQGHPHPGRFYT--GTSRTAYLPDNV---EGREVLGL---LRKAFAARLTFTIGTS--------VTTG--LTDTVWNDIHHKT------NVHGGPTAFGYPDPGYLNRVKQELAAGI

>Nve2

CPICYKRL---CLHKFCAS--CLTQAT---KQFPH-------CPVCRAPQETQQVIVQGNQPD-GSMLHRTQ-PHILPGYEPD-GSIVIQYAFPSGIQYPHHPNPGQPYD--GTLRTAYLPDTP---EGREVLRL---LRKAFDARLVFKVGRS--------LSSG--LDNQIWNIL-HKT------NMHGGPENYGYPDPFYLSDVKEELADGI

>Nve3

CAICIEKLLTKCNHCFHEE--CLLELL---KN-SK-GGYIQ-CPTCKTIHGHK--I--GTQPD-GSMTVHST-SHSLPGYPDC-GMITIRYDFRPGIQGPDHPHPGNRYHTSGFPRTCYLPDNQ---KGQKVLRL---LREAWSRRLIFTIATS--------VTTG--LEDTVWNEIHHKT------DAFTNHSGHGYPDPKYLDNVLAELASGV

>Dre1

CTICMERLLGKCGHLYHLL--CLVAMY---NNGNK-DGSLQ-CPTCKAIYGEK--T--GTQPP-GKMEYHII-PHSLPGHSDI-KTIRIVYDIPAGVQTTEHPNPGKKYSARGFPRHCYLPDNE---KGRKVLKL---LITAWDRRLIFTIGTS--------STTG--ESDTVWNEIHHKT------EFGSNLTGHGYPDPKYLDNVMRELEAGV

>Dre2

CIICMERLLTKCAHTFHML--CMLAMY---NNGTK-DGSLQ-CPSCKTIYGEK--T--GTQPK-GKMEIYSI-PQSLPGHPDC-GTIQIIYNIPPGIQGPEHPNPGQPYTCRGFPRFCFLPDND---KGRKVLEL---LKVAWTRRLIFTVGTS--------STTG--EPDTVWNEIHHKT------EMMSNVSGHGYPDPNYLDNVLSELASGV

>Dre3

CPICLETILTKCQHRFCKD--CLDTAF---QLKPA-------CPICGEIYGSL--T--GTQPKGGTMTVSRD-RSCLPGYKGY-GTIVITYYIPSGSQGVEHPNPGMPYH--GASRIAYLPDST---EGTHVLKL---LQRAFDQRLTFTIGRS--------STTG--KNNVVWNDIHHKT------SRDGGPTHYGYPDPDYLKRVQDELKAGI

>Dre4

CSICMGEMLDKCGHAFCRS--CLEQAF---QVKKA-------CPVCRLVYGQL--I--GNQPANGSMMVERDPDLELPGHEGY-GCICIIYSFPPGLQAQEHPNPGVRYP--GTDRVAYLPDNP---EGNRVLRM---LRRAFEQRLIFTIGTS--------MTTG--MHNVIWNDIHHKT------SIWGGPRCFGYPDPTYLVRVTEELREGI

>Hsa2

CIICMEKLLTKCSHAFHLL--CLLAMY---CNGNK-DGSLQ-CPSCKTIYGEK--T--GTQPQ-GKMEVLRF-QMSLPGHEDC-GTILIVYSIPHGIQGPEHPNPGKPFTARGFPRQCYLPDNA---QGRKVLEL---LKVAWKRRLIFTVGTS--------STTG--ETDTVWNEIHHKT------EMDRNITGHGYPDPNYLQNVLAELAAGV

>Hsa4

CTICMERLLSRCGHVYHIY--CLVAMY---NNGNK-DGSLQ-CPTCKTIYGVK--T--GTQPP-GKMEYHLI-PHSLPGHPDC-KTIRIIYSIPPGIQGPEHPNPGKSFSARGFPRHCYLPDSE---KGRKVLKL---LLVAWDRRLIFAIGTS--------STTG--ESDTVWNEVHHKT------EFGSNLTGHGYPDANYLDNVLAELAAGI

>Hsa1

CTICMERLLGRCGHMYHLL--CLVAMY---SNGNK-DGSLQ-CPTCKAIYGEK--T--GTQPP-GKMEFHLI-PHSLPGFPDT-QTIRIVYDIPTGIQGPEHPNPGKKFTARGFPRHCYLPNNE---KGRKVLRL---LITAWERRLIFTIGTS--------NTTG--ESDTVWNEIHHKT------EFGSNLTGHGYPDASYLDNVLAELTAGV

>Hsa3

CPICLGEILEKCRHSFCEG--CITRAL---QVKKA-------CPMCGRFYGQL--V--GNQPQNGRMLVSKDATLLLPSYEKY-GTIVIQYVFPPGVQGAEHPNPGVRYP--GTTRVAYLPDCP---EGNKVLTL---FRKAFDQRLTFTIGTS--------MTTG--RPNVIWNDIHHKT------SCTGGPQLFGYPDPTYLTRVQEELRAGI

>Hsa5

CTVSTQEHLKKGEHQILVDEKPVPIFLVPTENSIKKNTRPQISPTCQTSYGIQ--K--GNQPE-GSMVFTVS-RDSLPGYESF-GTIVITYSMKAGIQTEEHPNPGKRYP--GIQRTAYLPDNK---EGRKVLKL---LYRAFDQKLIFTVGYS--------RVLG--VSDVIWNDIHHKT------SRFGGPEMYGYPDPSYLKRVKEELKAGI

>Hsa6

CTVSTQEHLKKGEHQILVDEKPVPIFLVPTENSIKKNTRPQISSLTQSQ---------AETPS-GDMHQH---EGHIPNAVDS------------CLQKEEHPNPGKRYP--GIQRTAYLPDNK---EGRKVLKL---LYRAFDQKLIFTVGYS--------RVLG--VSDVTWNDIHHKT------SRFGGPEMYGYPDPSYLKRVKEELKAGI

>Gga2

CPICMEKILTKCKHVFCKS--CIKKAL---EYKQT-------CPVCNTVYGLV--Q--GDQPD-GRMNFKRT-SLSLPGYPNC-GTIEIEYVMQSGVQTQSHPNPGKTYY--GITRKAYLPDNK---EGQEVLQL---LRRAFNQKLIFTVGNS--------RTTG--AEDVIWNDIHHKT------SIYGGPMQFGYPDPDYLKRVRSELKAGI

>Gga3

CIICMEKLLTNCQHSFHML--CVLAMY---SNGNK-DGSLQ-CPSCKTIYGEK--T--GTQPK-GKMEVSTF-PQSLPGHRDC-GTIQIVYHISRGIQGPEHPNPGMPYTARGFPRYCYLPDNE---KGRKVLEL---LKVAWNRRLIFTVGTS--------NTTG--ESNTVWNEIHHKT------EMDTNLSGHGYPDPNYLDNVLAELAAGV

>Gga4

CTICMERLLAKCGHIFHLH--CLVAMY---NNGNK-DGSLQ-CPTCKTIYGVK--T--GTQPP-GKMEYHII-PHALPGHSDC-KTIRIIYNIPPGVQGPEHPSPGKSFTARGFPRHCYLPDSE---KGRKVLKL---LLVAWDRRLIFAIGTS--------STTG--ESDTVWNEIHHKT------EFGSNLTGHGYPDINYLDNVLAELAAGI

>Xtr3

CILCLKPLLYRCCHSHHVR--CLAHLY---KNLNE-DGILR-CPSCQTLYGSK--I--GSQPP-GKMCYHLI-PYSLPGHADC-QTIRIIYHISPGIQGPGQPNPGTKFTVPDFPLHCYLPNTD---KGRKVLRL---LIQAWERRILFPVIPS--------KVPG--IPDSVISRFPHKT------EFGSNLTGKGFPDSQYLDSVLRQLQDGV

>Xtr4

CIICMETLLKKCGHTFHQL--CVLAMY---NNGNK-DGSLQ-CPACKTIYGEK--T--GTQPK-GNMEYSLI-PQSLPGHQDC-GTIHIVYTIHPGTQGPGHPNPGKPYSARGFPRHCYLPDNE---KGRLVLEL---LKLAWARRLIFTIGVS--------STTG--ESDTVWNEIHHKT------EMNSNISGHGYPDPNYMDNVVAELAAGV

>Xtr5

CTICMERLLGKCNHMYHVL--CLVAMY---NNGNK-DGSLQ-CPTCKAIYGEK--T--GTQPP-GKMEFHVI-PHSLPGYPDC-KTIRIVYDIPSGMQGPEHPNPGKKFTARGFPRHCYLPDND---KGRKVLRL---LLAAWERRLIFAIGTS--------STTG--ESNTVWNEIHHKT------EFGSNLTGHGYPDPNYLDNVLAELHRGV

>Xtr6

CPICLSGFLEKCKHSFCGD--CISRAL---QVKKA-------CPICGCLYGEL--T--GNQPD-GKMEFVRDASLHLPGYEQY-GAIIIRYTFQPGIQGPKHPNPGVRYP--GTTREAFLPDSP---RGNKVLKL---FEKAFNQRLTFTIGTS--------VTTG--RSNVIWNDIHHKT------NCTGGPQMFGYPDPTYLRRVEEELEAGL

**DSL proteins**

>DDre4

SGVFELDLHEFKNFKGLLANGNSCTDCRTFFRVCLKNYQVVVSPGD-CIFGSALTPVLGTNSFRIFSTPIRLPFNFGWPGSFSLIIEAWHQISFFAIQRKLEVGAEWSQDVQSGKQTELRYSYRFICNENYYGDSCSKKCTPRDDRFGHYTCNPDGQLSCLPGWKGEYC

>DGga4

SGVFQLKLHEFVNSRGALASGEPCAHCRTFFRVCLKHFQAVVSP-GSCTFGSIITPVLGVNSFSIFDSPIKLPFNFTWPGTFSLIIQAWHLISQMAIQRSLAVGEVWSQDVQSSPLTQLRYSYRVVCSENYYGESCSRLCKRRDDRFGHYVCEADGSLTCLPGWTGEYC

>DLgi7

KGVIKVQLRTFKNPGGKGNNGHCCGACDHRFEICLDKVNGDHRITS-CQYGKITTKDVPDRNYIYVRNPMVFTLN-SIPSSIRVKIGVWDVDDSSSHDKNINLTPGKSEYIMLSNRIVLTGNVWSACLPDYYGSDCSVYCKASSNMWGFYTCDKTGAKKCLAGYTGRNC

>DLgi5

SGVFEVKIRSLFNKHGRTVSGRCCTTCRTFFTLCLLHYQKPVPVYPACTFGETKTQIVGANNIGIPAFSVAIPFSFSWPGNFAFILDAWRIILRASRAKRLKPSPLWVEDERLTRTSRLEFSYRVTCDENYHGLGCDKWCKERDDQFGHYRCTQGGVKVCSQGWQGELC

>DHsa3

AGVFELQIHSFGPGPGPGAPRSPCRPCRLFFRVCLKPGLSEEAAESPCALGAALSPGAPAPDLPLPDGLLQVPFRDAWPGTFSFIIETWRLLARVAGRRRLAAGGPWARDIQRAGAWELRFSYRARCEPPAVGTACTRLCRPRSASPEHGFCEQPGECRCLEGWTGPLC

>JBfl

SGYFELQINSIQNIAGQLSSGNCCDACDTYFRVCLKEYQVKPSSSGTCTFGRYTSPVLGGNSFSLNAGRLRLSF-VSWTLSAELVVTQPMVSSNTSSDGVMIISSNVCGD-HGNCISQPDGDFTCACNRGYTGT----FCQENKNECSPNPCRNNGTCVCKRPWKGRTC

>DLgi6

SGILEFKFVSYYNDEGDGANGHCCPECDHQFKLCVDRLPGMSRVSCYTSGEVVNQDNIPSFGSYIGNQPNYFKVHFSWPGKALVRVKIVDFVETLVKSYRLPTKPPRYTAELLAARTRLELDVRVSCDTHYYTRNCAVFCKPQSNENGFYTCNQTGAKICNEGWTGENC

>DLgi3

LQQLEVRISTFENTDGKNMNGTCCDKCKTMMKACI-----YTSVPDRCVTDFVES-ILGGNMII-GSKSIKIDKA-NRLQDIGIKIQMVSTGPSIYKTRYSTIDR--VKGVYPTNDAKLQIQARLYCADNYFGRDCNRYCLPDPD---RYDCDSEGNKLCSNNWTGQDC

>DLgi4

DFIVSLDTSNFRRSNLNLDQTVTCERCAGLLYYCIQTAGALSTSKEVCSYGYKHV-ATGDAVLNADSIPIRGSF-----RPISIRIEVFLLTYRVEFKESMNNYQDWYPVNLDDAVIQEDVSIKISCSGNYYGSDCSVYCSTYND--IHKHCRSNGTKVCAPGWTGDNC

>DSpu3

MDVY----------TGLVGEDMSA-----------------------INFS----SLVGN-----TTNPILSTFN-TWPGTVELRITVVDVDDDDSYTTIISGTPETSVDVASVETLFLEADYAFYCDEFYYSISCGTYCKDTDDDSGHYTCDSDGSKECLEGYEDSAS

>DOca

SGALKPQLI-----------------------LAIHVYESKTR------------QILG---------------DFHWTEANALSLTSQ-YFTRALSFGSLVSDEPMTRW--------LALRLHVDCSEYYDGNDCSTYCKPRDDEEGHFTCETNGTKKCLDEYI----

>DAqu1

DYTAEIRIFSYGNPRGNSLTYCCCDNCNTKVLICI--------ADKYCVTSTYMA--QGNQTFTNLANPIYINVPGALPFQVKQLFQFFNAAVRLDIMREFFNEENF---------VSLHYNITYICSNDYYGTTCSLYCKAYNDTNGHYTCNSAGQKTCLAGYTTNNC

>DSpu2

TVIVTFHLLTYLNPSSKQLDGVCCDKCDNKFEICFGPLGNDTTTED-CPFGKYKTRKIHEDDLFLFPTPLPGGVPEEWKGGFRLQMNVMDLVDVYRRNFHMRPGRSNRMVLDGEGSSRLVIDVKLYCDSHYYGPECSVYCRPRGDGIDHFYCDLTGGIICDAGYTGQRC

>DHro2

GGNLEWTILKYVNPGGKISNGQICEPCDHVFTFGLDDVNGDDNPLTTSAFNNEDT-----NTFTSVFNPIIYPFTF-WKGGIRLKVSVVDYVDGLVYKLVAKPSASYLSAVSVTGRTKLYFTYRVYCDANYYGDDCAKNCVGHDDLNGHYSCDSFGNKVCLKGWENEG-

>DLgi2

TGKVEAKLVKFKNREGKQYDGSCCDKCDHKFVICLGNV-TESSDMESCEFGRQETEPIESNEIIFTTNPLSFGFN-QWPGVVKVKIDVLHFEYQYTTKQMTREIDAPSKKLDLGRNTNFDIVLKVYCDLNYYGEDCNVFCEAKDDNTGHFICEDTGNKLCIQGWSGDDC

>DAqu3

RYTLLVKAVQYHNPDNQDWNDGCCDNCDLYFKFCIRNKGLSASNINNCTYGDVST-----NNYYFIWNDLTFNRNEPWPGSVQVLVESLDLIDRNAFNLDLSPNGQWSNELYAGDRAQFKIRVRLFCQQNYYGSNCNVYCQQNDDTNGHYTCGSDGAKICNNGYTG-NC

>DAqu4

VFTLKLEFHNYDNPNGWNSVKVCC-DCETKFSICLRN-GNTSQIDKSCPSSSDITSIVPGDSLTFLPNPLTFYLDSLHFSGVQVYIKIEDLIDELFVQIPSNGRNAAYRMMYGHGVASINASYQLACDDNYYGNDCSVYCKPQNDSAGHYSCNTTGEKICLSGYV----

>DAqu5

TGDFTLSFHRYSNPSDRDVNGNCCDSCDTHFKLCLRNGGTSHSQTGQCIPGTVLSPGVGGSSVNFHIGVISNPFGY-NKSGFQLYLEVWDILNRTSSQISAIG---------VFNRVSLLSSYRLLCSVNYYGFDCSVLCIPYNDTNGHYTCNSTGAKKCREGWQVTNC

>DAqu2

-GDYELLFRRYSNPT--DNNDNCCDHCDTLFRLCLRN--RDSNSEPQCIPGTVYDPTVGDNSITFISNPIIYERPGSIPSGFQLYMEVFNIPAQPTTERQVVGED---------GKISLELSYSLSCSQNYYGSDCSQQCIPRNDTNGHYTCNTTGGIICREGWQITNC

>DHro1

MSTFELQLTLFQTSQSSSSSSSSSSRCNVFFKICLTHQQPVMEAGPDCTFGSTVTDLWRNNSSN-MHHTVRLPISYKWTHKFALIIEAYGLLLKLILLKNVTSEQGWITESFHNGSTKLDYRYRLVCLHNIYGPFCSNSCVARDDSLGHYRCLRNGTKSCLQGWTGEWC

>DAae

SGLFELKLKYFKNERGVDNEGNCIGACKTRFRACLKHYQVTIDTSSPCTFGDVITPVLEGNSLNLTENPIRFPFEFGWPGTFTLIVEAW-LLHEKIRRTGPRHDRIWIGGAKFRRPSSGGGTRRTSGQPNAHGARCTPGCDEKHGHCSHGTCKKPWECLCNEGWGGLFC

>DSpu

AGTFELRLNSFSNDQAKDINGQCCGPCRTYFTVCLLHFLREIPEEPQCTFAFRNTSVLGENSFEVHDGLIQIDFDIDWPRDFSLALDAWRRLETLRIHRSLNVSTTWSNYTYPTSLHTLDYSYRVVCAETYYGSQCRDACIPKDDLFGHYLCDKDGGRVCMDGWEGNWC

>DNve

QGVFEVKLRRVWNPGGRNFTRQCC-SCRTYVRACLRQYSPGHPTD--CTFGNMLTSELGQDSFTARDHVIRFDIASSWPGSFSMIVELG-LIARTEIRRTFFPGKLWANTSHIGVSSNFSLSFRMVCNPNQYGDSCSKFCEPRDDKFGHMTCDANGTHVCLPGWQGPYC

>DTad

VGTFQFKLNSITNQLGLTAAKKCC-HCQTFFKICLRD--QVDSSDGMCGFGNVTTPVLGENSFTLFHNPISIHFNFTWPGAFILVVEALDTIDKVEYRDAIYSGAGWNERETTNGHVEMAFEYRLVCDRHYYGPGCDIACTPRNDLLGHYTCNDKGQKECLPGWKGKFC

>DCin

EGSFQLKLLDLVNRNGVTGAGVCCSQCRSSIRVCLKHYQAIVEPEAPCTFGNYSTPAIGGNSFVPRRHPIRFDFNFRWPGSFSMIVDILMIVSNGNLLTQAHNDDTWHTPPPYSHGVELRYAYRVKCKVHLYGSDCATHCHPRNDVFGHFTCDMHGNKVCMPGWMGKYC

>DLgi1

LGTFELKLTSFLNAHGLNSDGNCCNSCKTFFRVCLTHYQSEISNNPECTFGSKTTSVLGNNTIQFFKNPVQFEFQFSWPGSFSLIIEAWELIARVAVQRSAEVGKDWYTFKHDTPYSEINYSYRIICGEYYYGAGCSEFCRPRDDQFGHYKCSTNGTKICLDGWSGDMC

>DDreC

SGVFELKVLSFTSTS------SVCKDCQIFFRVCLKHSQALILPEPPCTYGTGMSEILSADSIS-SSAYISVPFNFKWPGIVSLIIETWNMISRLATKRRLAISEDWSQDVHLGRQSQLRFSYRVVCDEFYHGEECSDFCRPRNDTFGHFNCDAAGNRICLPGWKGDYC

>DXtr1

-GVFELKIHSFSTPR------PACASCNIFFRVCLKHAQPVVSPDPPCTFGSAVSDILPSDSKAIDSSPIRVPFHFKWPGIFSLIIESWNLLSRLATRRRLSIGEDWSQDIHLG-QSELRYSYHVSCDEHYYGDSCSDYCRPRDDNFGHYTCDEQGNRLCMSGWKGEYC

>DDreB

SGVFELKVHSFSTTR------RFCRDCNIFFRICLKHSEDVISAEPPCTFGTGQTSVLRADQSSISSAAIRVPFHFKWPGTFSLIIEAWNLISRLATRRRLAVGEDWSQDVHFGDQSELRYSYHVFCDEFYFGEACSDYCRPRDDTLGHYTCDENGNKECLVGWQGDYC

>DBfl1

SGVFELRLESFSNPTGSTQAGACC-SCKTFFRVCLKHYQAHVSPDPPCTFGSLETPVLGGNSFDIFANPIRLPFSFTWPGTFSLIVEAWTLITRLATQRHLSVGEVWHEDTHIEGQQQLSYAYRVVCDEHNYGEGCSVYCRPRNDVFGHYTCNEEGEKVCREGWKGQYC

>DDreD

SGVFELKLQEFLNKKGVTGNANCCKECKTFFRICLKHYQANVSPDPPCTYGGAVTPVLGSNSFQVFTNPIPFAFGFTWPGTFSLIIEALRLISRMTTQRHLTVGEEWSQDLQVGGRTELKYSYRFVCDEHYYGEGCSVFCRPRDDTFGHFTCGERGEIICNSGWKGQYC

>DDreA

SGVFELKLQEFLNKKGVQGNKNCCKECKTFFRICLKHYQPNASPEPPCTYGGTVTPVLGSNSFQVFTNPIRMNFGFTWPGTFSLIIEALRIISTMTTQRHLTVGEDWSQDLHSVGRTELKYSYRFVCDEHYYGEGCSVFCRPRDDAFGHFTCGERGEIICDAGWKGQYC

>DGga1

SGVFELKLQEFVNKKGLLSNRNCCRDCKTFFRVCLKHYQASVSPEPPCTYGSAITPVLGANSFSVFSNPIRFPFGFTWPGTFSLIIEALRLISRLATQRHLAVGEEWSQDLHSSGRTDLKYSYRFVCDEHYYGEGCSVFCRPRDDRFGHFTCGERGEKVCNPGWKGQYC

>DHsa1

SGVFELKLQEFVNKKGLLGNRNCCRACRTFFRVCLKHYQASVSPEPPCTYGSAVTPVLGVDSFSLFSNPIRFPFGFTWPGTFSLIIEALRLISRLATQRHLTVGEEWSQDLHSSGRTDLKYSYRFVCDEHYYGEGCSVFCRPRDDAFGHFTCGERGEKVCNPGWKGPYC

>DXtr2

SGLFELRLQEFLNKKGLLGNMNCCRDCKTFFRICLKHYQSNVSPEPPCTYGSAVTPVLGTNSFVVFSNPIRFPFGFTWPGTFSLIIEAIRLISRLATQRHLTVGEQWSQDLHSSDRTELKYSYRFVCDEHYYGEGCSDYCRPRDDAFGHFSCGEKGEKLCNPGWKGLYC

>DXtr3

SGVFQLELHEFINTNGMLVNGKSCLNCRTFFKICLKHYQTVVSP-GSCTFGSVITPVLGSNTFSIFTNPIKLPFNFTWPKTFSLIIEAFIKISQFTIQKPLNVGEEWSRDIQSGGQIQLKFSYRVVCSENYYGESCSRLCKPRDDRFGHYICEPDGRVSCLKGWKGEYC

>DHsa2

SGVFQLQLQEFINERGVLASGRPCEGCRTFFRVCLKHFQAVVSP-GPCTFGTVSTPVLGTNSFAVGRNPLQLPFNFTWPGTFSLIIEAWALISKIAIQGSLAVGQNWLLDEQTSTLTRLRYSYRVICSDNYYGDNCSRLCKKRNDHFGHYVCQPDGNLSCLPGWTGEYC

>JSpu

DGHFELDVISYVDPRGELADGSCCDECDVAFRACLTNFQPRTTYDGRCVFGEGSTDVVGGNTLLSKDTTIKFPFDFAWPTTFSLILEALQLIERAYHSGILVPSSSWVALRHDGATATVLYRVRLQCDDDFYGTTCLNYCTPRDDQFGHYTCDDDGGRVCDHGWTGSHC

>JNve

AGNFEVQFVSIQNVAGELRNGKCCDACETFFTICLKPSDGQASNSGTCTFGQYTTKVLGGNSFTVSEQIVSLRFTFSWLTTYLLVLEARQLIDETSLRGILFPNKTWESHSYNGPTASIKYNVRVVCDEHYYGRTCTILCKPRDDIFGHYTCDEQGHKICLPGWRGDHC

>SAae

SGFFEMQILEIANTNSHLLSGYCCGACATAFRLCLKEYQGTTSSSGGCAFGNASTPTLGGSSFVLEVGRVTLPFTFRWTKSFTLILQALRLIEETSFSGVILPSHEWNTLDHTGKNARITYRVRVQCADNYYNTTCTTFCRPRNDQFGHYTCGEQGNKVCLPGWQGANC

>DJBfl2

MSSFHLLTLQ---------------------------------------------------------------------SGYTLILEAWGLIDSYSVSGMIHPGNTWQNFQYNGPTAVIDYKIRVLCDEHYYGSNCSRLCRPKDDFVGHFTCDQNGNKVCREGWMGADC

>JXtr1

SGQFELEILSMQNPNGELQSGNCCDECDTYFKVCLKEYQSRVSAGGACSFGTGYTPVIGGNSFNLERNRIVLPFSFAWPRSYTLVVEAWDLIDKALHSGMINPSRQWQTLKQNAGMTHFEYQIRVICDEHYYGFGCNKFCRPRDDFFGHYTCDQNGNKTCLEGWMGPEC

>JGga1

SGQFELEILSVQNVNGVLQNGNCCDECDTYFKVCLKEYQSRVTAGGPCSFGSKSTPVIGGNTFNLEKNRIVIPFSFAWPRSYTLLVEAWRIIEKASHSGMINPSRQWQTLKHN-GAAHFEYQIRVTCAEHYYGFGCNKFCRPRDDFFTHHTCDQNGNKTCLEGWTGPEC

>JHsa1

SGQFELEILSMQNVNGELQNGNCCGECDTYFKVCLKEYQSRVTAGGPCSFGSGSTPVIGGNTFNLDRNRIVLPFSFAWPRSYTLLVEAWSIIEKASHSGMINPSRQWQTLKQNTGVAHFEYQIRVTCDDYYYGFGCNKFCRPRDDFFGHYACDQNGNKTCMEGWMGPEC

>JDre1a

SGHFEMQVLSMQNVNGELQSGACCDQCDTFFRVCLKEYQSRVSSGGPCSYGSGSTPVIGGNTFSVDKTRIVLPFSFAWPRSYTLIVEALQVIEKAVQSGMINPNRQWQVLKHNGPVAQFQYQIRVTCDEHYFGFGCNKFCRPRDDFFGHYTCDHNGNKTCLEGWAGPEC

>JDre1b

SGHFELEILSMQNANGELQNGACCDECDTYFKVCLKEYQSRVSSAGACSFGTGSTPVLGGNKFSTEKSRIVLPFSFAWPRSYTLIVEALKLIEKAYHSGMINPNRQWQRLTHNGPVAQFEYQIRVTCLEHYYGFGCNKFCRPRDEFFGHYTCDQNGNKTCLEGWTGPDC

>JXtr2

TGYFELQLISLKNANGELLNGECCDECDTYLKVCLKEFQAKITPTGPCNYGSGFTSVLGGNTIYLESGRITIPFQFAWTRSFTLILEAWLLIDRVTYAGVTSPEDQWTLLPLTGHVAQFDVKIRVKCDENYYGSMCNKFCRPRNDFVGHYTCDSNGKKACMEGWMGAEC

>JHsa2

MGYFELQLSALRNVNGELLSGACCDECDTYVRVCLKEYQAKVTPTGPCSYGHGATPVLGGNSFYLDPGLVVIPFQFAWPRSFTLIVEAWLLIERVSHAGMINPEDRW-KSLHFGHVAHLELQIRVRCDENYYSATCNKFCRPRNDFFGHYTCDQYGNKACMDGWMGKEC

>JGga2

TGYFELQLNSVRNVNGELLNGECCDECDTYVKVCLKEYQAKISPGGPCSYGSGSTPVLGGNILYLETGRIVIPFQYAWPRSFTLILEAWLLIERVAHAGMINPEDRWKTLQFNGPVANFEVQIRVKCDENYYSALCNKFCGPRDDFVGHYTCDQNGNKACMEGWMGEEC

>JDre2a

SGYLELQLISVENDRAELANGNCCDQCDTYLKVCLKEYQVEVTTSGFCTYGTGSSSVVGGNTFQLDLGTVIIPFQFAWPRAYTLIVEAWQLIMRSVNRGEMNPGEERQVIQHKGRTASIQYSVRVRCDRHYYGNKCNKQCRPRDDYFGHYTCDQSGNQQCMEGWTGQDC

>JHro

--------------------------MAMHFKISFWH------SKETCEFYVGTD------------------------------ADSY-LIERAHYSGVILPRSEWQTMSFAGHTAILKYKIRVQCDLHHFNVTCTKFCRPRNDTFGHYTCDSNGDKVCLNGWIGTNC

>JLgi1

SGYFHLQVNSVSNPRGEIADGSCCNKCNTFFRVCLKEYQERVTTTGTCTFGNQSTVALGGNSFTYSRAMLKLPFEFAWTRSYTLMVEAWSLIERAAQTGMILPGQDWHTIIHTGPTASLVYKIQVVCDEHYYNTTCTKFCRPHNDRFGHYTCDSNGDKVCLHGWMGQEC

>JLgi2

HGTIHITIP---------------GRYNTYYNT-------------------------------------------RVMRSYTLMVEAWSLIERAAQTGMILPGQDWHTIIHTGPTASLVYKIQVVCDEHYYNTTCTKFCRPHNDRFGHYTCDSNGDKVCLHGWMGQEC

>JGga3

TGYFELQLNSVRNVNGELLNGECCDECDTYVKVCLKEYQAKISPGGPCSYGSGSPPVLGGNILYLETGRIVIPFQYAWPRSFTLILEAWDLIERVAHAGMINPEDRWKTLQFNGPVANFEVQIRVKCDENYYSALCNKFCGPRDDFVGHYTCDQNGNKACMECWMGEEC

>JDre2b

SGYFELQLIAVENVNGELWDGECCDECDTYFKVCLKEYQSEVTTTGQCTFGSGSTDVLGGNIISFDVGKIIIPFHFAWPRSYTLILEAWNLIERHIHASMVNPGDHWQSIRHPGITAHIEYRIRVRCDENYYGSKCNKQCRPRDDYFGHYRCDPSGNIVCLDGWMGEDC

**FRINGE**

>Tva

NLYRVLTEYDPNEKKVLGHFYCSWSKVVYGVEDEDKCLLFAQG------GAGV---AISNAYFKVIAPYLT--GCNNNF-----TDRNYAMRFAKCSEDHV----GKD---W-----DD-GYIISRRNEEFFS-CDP-VTEINFGEVNLPPVNSDWIRATDNQSVFVDWTNISGKAYS------------MFYGPSNL-EYYYRFGWTISVS-------MIGGVVG------------AASSPLVPQ------------FADWK-KDKPIGFIQNFSDT-----------ATVEI------DSV---PDLDV-EFVDSTNRDMLYFTMKMKCPPVEEY--KW-

>Ath

HIVFGIAASSVLWETRKEYI-KSW----WRPGK-TRGVVWIDKRVRTYRNDPLPEIRISQDTSRFRYLLIS--TISDVF-----Y-KKSLVRISRVVTETL--RLGKKGVRWFVMGDDD-TVFVVDNVVNVLSKYDH-TQFYYVGSSSEA-HV-SYSMAFGGGGFAIS-YALALELLRMQDRCIQRYPGLYGSDDRIQ-ACMTELGVPLTKEPGFHQYDVYGDLLGLLGAHPVAPLVSLHHIDVVQPIFPKMKRSRALRHLMSS-AVLDPA---SIFQQSICYDQNRFWSISVSW-----GCRWRLDSPGKIDSVVVLKRPDPLRW-HKVGPHLLFASLLTTC

>Aqu1

DIFISVKTSS-LNAYRQQTVFLTW----AQTVPIDQISFTTD-------KATNWTDAFAAHG----YKINTAPHCGLGH-----T-DYSLCKSGVEYDQFYKAIEAGRNFSWMCHIDDD-EYMNVWKLKRMLIKYDP-NKPWYIGKSHHEYWKKIYKFNTGNV-YCLS-KQIMKETEK------------YFRGRNFI-KTCEKAHWIDDVTIAI----VIVAVLG----------YEPTEEKQMWS------------HYEVL-DQLPKS---ESLKM-----------INFGY-----GGRWN--TSLNLPNPRFSYNADPTRF-LSYHCMMYPKL--KWC

>Aqu5

DIYFSVKTASIYPD-RLNTVMLTW----AQTVSIEQISFTTD-------KATNWTDAFAARG----YKINLAPHCGLGH-----EHYFSLCKSGVEYDHFYKSIESGKNYNWLCHIDDD-QYYNVWKLKRMLIKYDP-NKPWYIGKSH---HGHDYKFNTGNV-YCLS-KQTMKEIEK------------YLRAKNFP-KSCDKTRQPDDVTIAI----IIVGVLG----------HTPTEEKQMWS------------HLELL-DQLPKE---ESMKM-----------ISFGY-----GGRWN--TSLNLPNPRFSYNADPTRF-LSYHCMVYPKL--KWC

>Aqu6

DIYFSVKTASIYPD-RLNTVMLTW----AQTVSIEQISIITD-------ATTNWTDAFSSRG----YSISAATQCGLGH-----S-VQSLCKSGVEYDRFYKSIESGKKYNWFCHIDDD-QYYNVWKLKRMLIKYDP-NKPWYIGKSHHGPTPNNYKFNTGNV-YCLS-KQMMKETEK------------YLRGGNFD-KSCSKTTRIDDVTISI----IIVGVLG----------HKQTEEKQMWS------------HLELL-DQLPKE---ESMKM-----------ISFGY-----GGRWN--TSLNLPNPRFSYNADPTRHGISLLS-LYGVS-----

>Aqu2

DVYFSIKSTEKYHESRLQLLVLTW----FQTVHPHNLHIVTDT------EDTTTELLRDFGY----TVHIA--DCPKGH-----T-HHPICKCGVEFEQFYIAKENGSHYNWYCHLDDD-MYLNVLSLFELLDTLNP-LDSHYLGQRSIN-RRDSYYFATGAI-YCIS-TPLMEQLEY------------YLRGKEKMMRYSGAAGIPDDMLIGL----IIEGVLG----------YNLTEVSTMNS------------HLFPL-KRISTT---DLVKQ-----------ITISY-----GNGKEVKNTISI-EDLFDENEDPTRF-LSYHCLLHPST--QWC

>Aqu3

NIMIAIRTTKKFHQKRLPYLYDTW----LNKVNGSNVFLVTDA------EDEEY--QERSKQLGIHYVVSS--LCGESLLWPIPS-RWYLCRTGEALTLMY--KPQNIQYDWFCYLDDD-IYLIMENLIKLIAKFPK-DELSYIGRPGTP-WEKHYHFASGGF-YCLS-RTILDKIKP------------WIVGGHNLGDTCRQLLEPDDLTIGC----AVELLGG----------GKLSRTLLFHH------------HGMNLAKAVNAN---TLKDQ-----------IAIPY-----GGIVD--NAIEVPNAAFSFDEDPSR------------------

>Aqu4

NIMITIRTTRKFHQKRLPYMYDTW----LNKVNGSNVFLVTDA------EDEEYQEKSRQLG---IHYKII--SCGKDY-----S-RWSLCKSGEEMALMH--RPENKQYSWFCHLDDD-IYIILKNLVNLLSKFDPLKEPIYMGRAGTH-WKHPFHFAVGGM-YCLS-RAMLDKVKP------------WLGNGETMGDTCNKLLQPEDVAVGA----TVELLAK----------ERLSRTKLFHP------------HGLILSRFVNPR---TLKDQ-----------IGFAY-----GGYKN--NAIKVPNARFPFSEDPSRF-KSLHCHLFPDS--ALC

>Cin1

DIYITVRTSRKFHESRVAPIVQTW----FNLAR-KQTYIFTDG------DDDKL--NETTGG----HIINT--HCGQEY-----N-RPHLCKTGTVFDKYL--ASGKK---WWCRFDDD-NYVNPPRLVNLVNGYNW-TQDICIGKLSVP--SEIYQFAHGGAGCCIS-RPLALKMQP------------WCGREKLV-ETTEDVGRHEDCTLGF----IITNRLK----------IDLTLTDLLHS------------TRESL-RDLNPG---TLHEQ-----------VSIGQ-----GNTVNL-DQAKN-AKIFSHDVDPTRF-ITLHCFLYPTS--SIC

>Cin2

DIYISVKTSRKFHESRLDPIVKTW----FKLAK-KQTYFFTDG------DDDKL--NETTEG----HVINT--HCGHTK-----T-RPHLCKMGTEFDTYL--ASGKK---WWCRFDDD-NYVNPPRMVHLVNGYNW-TQDICIGKLSVP--LEKYKFAHGGAGCCIS-RPLALKMKP------------WCGREKLV-VTTRETHMNDDCALGF----IITNRLK----------IYITITDLLHS------------TREKL-KNLNPD---TLHEQ-----------VSLGQ-----ANTVNL-NKVKS-SKIFNHDVDPTRF-MTLHCFLFPTA--DIC

>Cin3

DIFISVRTSRKFHESRIDPIVKTW----FNLAR-EQTYIFTDG------DDDKL--NETTGG----HVINT--HCGRDY-----N-RSHLCKTGTVYDTYL--ASGKK---WWCRFDDD-NYVNPRRVVHLVNGYNW-TQDICIGKLSVP--FEIYQFAHGGAGCCIS-RPLALKMKP------------WCGREELV-VTTREAGMQEDCALGF----IITNRLK----------INLTLTDLLHS------------TRESL-EDLNPD---TLHEQ-----------ASIGQ-----SNTVNL-DKVKN-VKIFNHDVDPTRF-ITLHCFLFPTA--NIC

>Cin4

DIFISVRTSKKFHEPRIVPIVKTW----FNLAK-KQTYIFTDG------EDDKL--NETTGG----HVINT--NCGPTS-----S-REHLCKTGTVFDTYL--ASGKK---WWCRFDDD-NYVNPPRLVHLVNGYNW-TQNICIGKLSVP--SETYQFAHGGAGCCIS-RPLALKMKP------------WCGRKNLV-VTTREAVMNEDCALGF----IITNRLK----------VALTLTDLLHS------------TRERL-KDLNVD---TLHEQ-----------VTIGQ-----GNTVNL-DKAKS-GKKFNPNVDPTRF-MSLHCFLYPTA--SIC

>Cin5

DIFISVKTSGKFHKTRLNVIIDTW----FRDAN-QQTYFFTDT------DDDGL--SKKTDG----HMVNT--MCNSSH-----I-RRDLCKLGAEYDFYI--KSNKR---WWCHFDDD-NYVNVDQLVMLLRDYDH-NMDFYIGKPSLN-YPVGFWFATGGAGVCIS-KALAQRMKP------------WCSNGGLY-RTSEHLNAPDDCTLGF----VVSNRLA----------VELTSSNLLHS------------HLETL-GQLNPA---TLTEQ-----------VTLSY-----GNRNNIIQLQES-SHNLPISKDPTRF-RSLHCKRKPDT----C

>Cin6

---------------RLTFIINTW----FNQAK-DQIFFVTDD------DDIDL--HLKTNG----HVVNS--HCGNTH-----E-LQDLCKTGKEYDLFM--TTNKK---WWCHFDDD-NYVNINALVKFLGTFNW-QEDFYIGRRSVT-RKVKFIFATGGAGVCIS-SALAKKMSP------------WCKNGEFL-KTSKHLKHNDDCTLGF----IITNLLK----------VDLTLTKLFHS------------HLETL-DTLNPA---SFSTQ-----------VTLAH-----GNVIIINNNTTN-TTVFNTDLDPSRF-YSLHCLIFPGL--PMC

>Cin7

DIFITVKTSKQFHCSRLGVIVSTW----FAEAK-NQTYFITDG------ADAEL--NHTTNG----HVVPS--KCATDH-----S-LSALCKLGVEYDTFM--KSDKK---WWCRFDDD-NYVNVKLLVKFLREFNW-KNDLYIGRRSRT-EPVDIFFTTGGAGVCIS-SPLANKMKP------------WAASGEFL-RTSQALGHSDDCTVGF----IIINRLK----------VNLTESILFHS------------HMELL-SDIPIT---TFREQ-----------ITFSH-----RNIINITPNNTD-TPVFDFKSDPTRF-LSLHCYIHPAY--QIC

>Spu

DVFIGVKTTEKYHSSRLQLILDTW----YSLAP-EQTYFFTDV------DDSDY--QDKSNG----HMVNT--ECQGTH-----S-RLALCKTSKIFSMFY--KSDKR---WLCHVDDD-NYLNVPELMKLLRQFDH-NQDHYLGRASLS-HPVSFWFATGGAGFCIS-KALATKMMV------------YASSGTFE-RMCQRVRLPDDVTIGF----IIEVLLK----------KPLTKVQTFNS------------HLQQL-ARIPTK---QLQNQ-----------LTLSY-----SKRR---NVVNM-VSIMS--DDPTRF-KSFHCTYFRGYGNGNC

>Csp1

----------------------------------MTTYFFTDE------EDEEF--SKRTKG----HLINT--NCTAGH-----T-RRCVK--------------------W------------RWSTIRLLPQRKG--------------HPIAFWFATGGAGFCIS-RGLALKMMP------------HTSGGRLK-TVCEHIRLPDDCSIGY----IISFKLK----------KELTIVKDFHS------------HLEGL-WKINHR---NIEDQ-----------ITMSYMCMSSGPSRNGCNSVDI-SSGFPPHVDPTRF-LSIHCLLYPNL--AMC

>Bfl

DIFIGVKTTEKYHRHRMDLLMDTW----VSLAK-DQTFVFTDS------DDEQL--RSRLGD----HLINT--NCSSSH-----M-RQALCKMAVEYDMFL--QQDKR---WFCHVDDD-NYLNVHELVKLLNQYKH-TDDIYLGRPSIN-HPVNFWFATGGAGFCIS-KGLALKMIP------------YASGGKFM-SSCERIRLPDDCTLGF----IIERLLR----------VKLVQIQQFHS------------HLEWL-KFIKKE---ELPHQ-----------VSLSY-----GSAR---NVIGL-DEVFSESDDPTRF-KSLHCLLYPYV--DLC

>Nve1

------------------------------------------------------------------------------Y-----S-RAALCKMQAELNYFW-EKSNER---WFCHFDDD-NYVNFPALIKLLREHNH-TYPHYIGKPSIN-HKVSFWFATGGAGLCLS-RVLVARMAE------------YFRNHAFV-QTCDRIGLPDDCVLGY----IAEYALG----------VKLKKSNLFHS------------HLEGL-RFIRQE---ELRDQ-----------VTFSH-----GSFY---NRLKI-SGPFSEAIDPSRF-MSVHCLLYPGT--IWC

>Nve2

DVFLAIKTTRNYHQSRMQVLMKTW----ISLAK-EQVYVFSDG------DDPDL--NKLLGK----FLIV---HXSYYY-----S-RAALCKMQAELNYFW-EKSNER---WFCHFDDD-NYVNFPALIKLLREHNH-TYPHYIGKPSIN-HKVSFWFATGGAGLCLS-RVLVARMAE------------YFRNHAFV-QTCDRIGLPDDCVLGY----IAEYALG----------VKLKKSNLFHS------------HLEGL-RFIRQE---ELRDQ-----------VTFSH-----GSFY---NRLKI-SGPFSEAIDPSRF-MSVHCLLYPGT--IWC

>Hsa1M

DVFIAVKTTRAFHRLRLELLLDTW----VSRTR-EQTFVFTDS------PDKGL--QERLGS----HLVVT--NCSAEH-----S-HPALCKMAAEFDTFL--ASGLR---WFCHVDDD-NYVNPRALLQLLRAFPL-ARDVYVGRPSLN-RPVQFWFATGGAGFCIN-RKLALKMAP------------WASGSRFM-DTSALIRLPDDCTMGY----IIECKLG----------GRLQPSPLFHS------------HLETL-QLLRTA---QLPEQ-----------VTLSY-----GGKL---NVIKL-QGPFSPEEDPSRF-RSLHCLLYPDT--PWC

>Hsa2L

DVFIAVKTTKKFHRARLDLLLETW----ISRHK-EMTFIFTDG------EDEAL--ARHTGN-----VVIT--NCSAAH-----S-RQALCKMAVEYDRFI--ESGRK---WFCHVDDD-NYVNLRALLRLLASYPH-TRDVYVGKPSLD-RPVHFWFATGGAGFCIS-RGLALKMSP------------WASGGHFM-NTAERIRLPDDCTIGY----IVEALLG----------VPLIRSGLFHS------------HLENL-QQVPTS---ELHEQ-----------VTLSY-----GNKR---NAVHV-KGPFSVEADPSRF-RSIHCHLYPDT--PWC

>Hsa3R

DVFIAVKTTRKNHGPRLRLLLRTW----ISRAR-QQTFIFTDG------DDPEL--ELQGGD----RVINT--NCSAVR-----T-RQALCKMSVEYDKFI--ESGRK---WFCHVDDD-NYVNARSLLHLLSSFSP-SQDVYLGRPSLD-HPVKFWFATGGAGFCLS-RGLALKMSP------------WASLGSFM-STAEQVRLPDDCTVGY----IVEGLLG----------ARLLHSPLFHS------------HLENL-QRLPPD---TLLQQ-----------VTLSH-----GNPQ---NVVNV-AGGFSLHQDPTRF-KSIHCLLYPDT--DWC

>Dre1M

DIFIAVKTTGRFHKSRLALLLETW----ISETK-EHTYIFTDS------PDVDI---SSEGF----NVVVT--NCSPEH-----S-HQALCKMAAEYDYFM--ASYKK---WLCHVDDD-NYLNPGALLSLLMAFPA-DGDIYVGKPSLD-RPVHFWFATGGAGFCLS-RNLAERMAP------------WASGPRFE-QTSAVIMLPDDCTVGF----IVERRLG----------ISMIHSNMFHS------------HLENL-LLLSPS---DIPKQ-----------VTLSY-----GSKM---NSVEL-KGVFTKDEDPSRF-RTVHCLLYPTT--SWC

>Dre2L

DIFIAVKTTKKFHRSRLDLLLDTW----ISRNM-RQTYIFTDG------EDEEL--KKKIGS----HAINT--NCSAAH-----S-RQALCKMAVEYDKFI--ESGKK---WFCHVDDD-NYVNTKTLVKLLSNYPH-TQDMYIGKPSLD-RPVNFWFATGGAGFCIS-RGLALKMSP------------WASGGHFM-NTAEKIRLPDDCTIGY----IIESVLG----------VSLTRSSLFHS------------HLENL-QQVSKS---EVHKQ-----------ITLSY-----GNKR---NIINM-KGAFSVEEDPSRF-KSVHCLLYPDT--PWC

>Dre3

DIFIAVKTTRKYHKSRLQLLSQTW----VSRAK-EQTFIFTDG------EDKEL--RLKAGL----NIINT--NCSAAH-----T-RQALCKMSVEYDKFI--ESQKK---WFCHVDDD-NYVILPSLLELLSSYSH-TQDVYLGRPSLD-HPVKFWFATGGAGFCIS-RGLALKMSP------------WASLGNFI-TTAEKIRLPDDCTIGY----IIEALLE----------VPLTHTGLFHS------------HLENL-QRLPAE---NILRQ-----------VTLSY-----GNRR---NVVSV-GGAFSLAEDPTRF-KTVHCKLYPDT--EWC

>Gga1

DVFVAVKTTKRFHQSRMELLLDTW----ISRAR-EQTYVFTDE------EDDAL--KRRMGD----HVVFT--NCSTEH-----S-HSALCKMAAEFDAFL--SSDQS---WFCHLDDD-NYLNPEALLKLLSSYSA-MKDVYVGKPSLN-RPVRFWFATGGAGFCIS-RKLARKMMP------------WASGKNFL-STSELIRLPDDCTIGY----IIECKVG----------GQLLPNRLFHS------------HLENL-QLIPTS---DLMQQ-----------VTLSY-----GNKL---NVIKL-SGPFSPQEDPSRF-RSLHCHLYPDT--PWC

>Gga2

DNLLIMGMLGHGQSPAVSALWSSK----VPRLF-FQTFIFTDG------EDEEL--KKQAR-----NVINT--NCSAAH-----S-RQALCKMAVEYDKFI--ESGRK---WFCHVDDD-NYVNVRTLVKLLSSYPH-TQDIYIGKPSLD-RPVHFWFATGGAGFCIS-RGLALKMSP------------WASGGHFM-STAEKIRLPDDCTIGY----IIESVLG----------VKLIRSNLFHS------------HLENL-HQVPKT---EIHKQ-----------VTLSY-----GNKR---NSIHM-KGAFSVEEDPSR------------------

>Gga3L

DVFIAVKTTKKFHKARLELLLDTW----ISRNR-DMTFIFTDG------EDEEL--KKQAR-----NVINT--NCSAAH-----S-RQALCKMAVEYDKFI--ESGRK---WFCHVDDD-NYVNVRTLVKLLSSYPH-TQDIYIGKPSLD-RPVHFWFATGGAGFCIS-RGLALKMSP------------WASGGHFM-STAEKIRLPDDCTIGY----IIESVLG----------VKLIRSNLFHS------------HLENL-HQVPKT---EIHKQ-----------VTLSY-----GNKR---NSIHM-KGAFSVEEDPSRF-RSVHCLLYPDT--PWC

>Gga4R

DIFIAVKTTRKYHKTRLELLFQTW----ISRAR-GQTFIFTDW------EDREL--RLKAGD----HMINT--NCSAVH-----T-RQALCKMSVEYDKFL--ESGQK---WFCHVDDD-NYVNPRTLLRLLSAFSP-SQDVYVGRPSLD-HPVKFWFATGGAGFCIS-RGLALKMSP------------WASLGNFI-STAERVRLPDDCTIGY----IIEGLLE----------VKLLHSPLFHS------------HLENL-QRLQGE---SVLQQ-----------VTLSY-----GNKH---NVVSV-GGVFGLQQDPTRF-KSVHCLLYPDT--IWC

>Xtr1

DVFIAVKTTKKFHRSRMDLLMDTW----ISRNK-AQTFIFTDG------EDEEL--QKKTG-----NVIST--NCSAAH-----S-RQALCKMAVEYDKFI--ESNKK---WFCHVDDD-NYVNVQTLVKLLSRYSH-TNDIYIGKPSLD-RPVNFWFATGGAGFCIS-RGLALKMSP------------WASGGNFM-NTAEKIRLPDDCTIGY----IIESVLG----------VKLIRSNLFHS------------HLENL-HQVPQS---EIHNQ-----------VTLSY-----GNKR---NAILM-KGAFSVEEDPSRF-RSVHCLLYPDT--PWC

>Xtr2

DLFIAVKTTKKYHRNRLNLLMQTW----ISRAK-EQTFIFTDW------EDQEL--RQKAGD----HMVNT--NCSAVH-----T-RQALCKMAVEYDKFV--LSDKKA--LFLNLDPCLLYMNMLYEMKLRNKGIPFTQRVN--------NRHFYWFATGGAGFCIS-RGLALKMSP------------WASMGNFI-STAEKVRLPDDCTIGY----IIEGMLE----------VKMQHSTLFHS------------HLENL-QRLPPK---SLLKQ-----------VTLSY-----GNKW---NVVRV-SGAFSLADDPTRF-KSVHCLLYSDT--DWC

>Xtr3

DLFIAVKTTKKYHRNRLNLLMQTW----ISRAK-EQTFIFTDW------EDQEL--RQKAGD----HMVNT--NCSAVH-----T-RQALCKMAVEYDKFV--LSDKKA--LFLNLDPCLLYMNMLYEMKLRNKGIPFTQRVN-NRFSTE-HLMKMWFATGGAGFCIS-RGLALKMSP------------WASMGNFI-STAEKVRLPDDCTIGY----IIEGMLE----------VKMQHSTLFHS------------HLENL-QRLPPK---SLLKQ-----------VTLSY-----GNKW---NVVRV-SGAFSLADDPTR------------------

>Lgi

DVFISVKTTARNHQLRLRILLNTW----ILLAR-EQTHIFTDT------DDPALDKERENGV----EIINT--KCPPNH-----S-RRALCKMAIEYDTFL--ASKKR---WFCHVDDD-NYVNIPQLVKLLRQYNH-TQDWYLGKPSLR-KPIAFWFGTGGAGLCIS-RSLALKMMP------------YASGGRLM-TIGESIRLPDDCTMGY----IISHLMK----------KQLTVIEQFHS------------HLESL-NLMSKR---DLVNQ-----------ITYSY-----KKTK---NILNI-DG-FSIKQDPTRL-WSLHCFLFPTF--REC

>Aae

--------------MRVRTI-------------------------------------------------------------------------------------------WWCHFDDD-NYVNVPRLVRLLDEYSP-TQDWYLGKPSIS-SPVTFWFATGGAGFCIS-RALALRMLP------------IASSGKFV-AIGDKIRFPDDVTMGF----IIEHILN----------VPLTVVDAFHS------------HLEPM-EFIRPE---TFHDQ-----------VSFSY-----ANEW---NVVKV-DG-FDLKTDPKRI-YSLHCYLYPFF—SIC

**FURIN**

>Bfl16

WNL-------SRVRVLTDKGDYMLMSHDRTVIEDYLFR----ERNGIRRN----------------VDQRGALLRAES--------------------NVHMNVLPAWVRGYYGQGVVVGVVDDGIFLENPDLMPNIAEGLSYSVVDDSLDPTPSAAVF---SHGTRCAGIVAAKANNNFCGVGVAPKAKIAGMKLFVGASAD-LTDAEEALALSHSYNDISIYSCSFGPSDYNNVLEGPDTLTYAAMKLSGRNGKGSIYMFSAGNGGNYGDSCAYNGYINNIYAIGISAVLTDGSLARYDEACTSIFGVTYSRQYDN--TLVVPYGPGGCKTKFSATSAAAAMGSGVIALVLSANEALSARDVQHLIARTSKNNGIGN--TWKMNAAGFRVSDYCGFGLLDAGKLTSLAVTWRSVSEQVVCSLKQ--EDRVIPRQLETNVTVSPKNCENG--TIQQLEHVLLTVNITFPRRGHLRIAITTPENTTSVIVPGRPTD--EEPDLAWTFMTIHHWGERTEGTWLLHVENTHPHRQPAYLNEFRLAEPSTLTTT--

>Bfl17

WAVQLEGGR-QEALRVAQKAGVTLVKKI--FGDYYLFKEEINARQTRLKIDKDREKILKADQSVK--MVKEQLTHTRQDPMWGSQY--LHTG------TVHMNVLPAWERTQKGEGVVVGVIDDGIFTNQPDLRDNL----------DTSDSTPNIPQL---SHGTNCAGIIAAVHNNSFCGVGVAYRAKVAGIKMFSGAITE-LSDAQEASALSHEHQRISIYSCSWGPSDWNNALEGPDTVAKEALTMTGRSGRGSIFVFSTGNGGSYNDSCAYNGYINTNNTIGIGGLLQDGSIPSFAESCTSVFAVTYSRDYGDTANLVVPSRASGCGTTFSGTSPAAAMAAGVFTLVLSANDQLSVRDVQHLVTRTSRSTGICG-QTWKENSAGFRVSDYCGFGLLDAGQLTEMATNWNCVPEQDICTEQG--VRMNIPQEVHTSITVQQNSCV-----VNYLEHVLLTVRITFPHRGHLQIRLTSPGGTVSDIVPGRATD-MEADL-EWTFMTLHHWGESAVGTWELSIENTHPHSSTGVLEGWTLALLGTATDP--

>Bfl11

LRIRLTSPGGIVPGRATDEWTFMTLHHWGESAVGWRLSQPHLSSTGVLETGTGICNTQSLDPGAIY-NRCHWLSTITKVKTSAKEWFINKNGDTNDTGPVHMNVLPAWERTQEGEGVVVGVIDDGIFTNQPDLRDNLDLGLSYDIFEETTDPTPNIEQL---SHGTNCAGIIAAVHNNSFCGVGVAYKAKVAGIKMFSGAMTD-ISDAQEASALSHEHQHISIYSCSWGPSDWNNALDGPDTVAREALVMAGRNGKGSIFVFSTGNGGTYNDSCAYNGYVNTNNTIGIGGLLQDGSIPSFAEACTGVFAVTYSRDYGDTADLVVPYRSSGCRTSFSGTSPAAAMAAGVFSLVLSANDRLSVRDVQHLVTRTSRNSGICG-QTWKENSAGFRVSDYCGFGLLDAGELTAMAVNWKSVPDQVSCIEQRESLSVNIPQEVHTSISVPQDSCV-----VNYLEHVLLTVRITFPHRGHLRIRLTSPGGTISDIVPGRATD-MEPDL-EWTFMTLHHWGESAVGTWQLSVQNTHPQSSTGILEAWTLVSLGTTADP--

>Bfl12

-----------------------------------------------------------------------------------------------------MNVLPAWERTRKGEGVVVGVIDDGIFTNQPDLRDNLDLGLSYDIFEETTDPTPNIEQLVFSSHGTNCAGIIAAVHNNSFCGVGVAYKAKVAGIKMFSGAITD-ISDAQEASALSHEHQHISIYSCSWGPSDWNNALEGPDTVAREALVMAGRDGKGSIFVFSTGNGGTYGDSCAYNGYINTNNTIGIGGLLQDGSIPSFAEACTSVFAVTYSRDYGDTANLVVPYRSSGCRTSVSGTSPAAAMAAGVFSLVLSANDGLSVRDVQHLVTRTSKNSGICG-QTWKENSAGFRVSDYCGFGLLDAGELTAMAANWKSVPDQVSCTEEGGSLRVNIPQEVHTSITV-QSSCV-----VNYLEHVLLTVRITFPHRGHLQVRLTSPGGTISDIVPGRATD-MEPDL-EWTFMTLHHWGESAVGPWRLSIQN--------------------------

>Bfl13

WAVQVEGGQ-EEAVRLADKHGFTYGGKI--FGDYHLLRHS-HVSRRSTSDHEKHYGLLKENS-VRW-EQQVWKERRVTDPDWPKQWYMNHCA------GLDMHVSGAWVRGYTGEGVNLAVVDDGLQTDHPDL--NFDAALSTDVVDGDRDPSHPADSTG---HGTKVAGVATAVANNSLCGVGVAPAAKVGGSFVSLT---SEVNDAQDATAYSFGQDAVDVYISSWGPNDFAE-VDGPGPLLKQALRNGGRSGRGNVYIFSSGNGGQYPDSCAFDGFVNNPFSLAFGSVSGTGRRAAHSEACSALIASVFAEDRNDGQEMVTTKITSECTGDFYQSSAGAPLAGGILALAINANAALTWRDLKHLIVQTSQPDSRLTSTDWMTNAAGHRVSSYFGFGLLDASKLVDAAKTWTTVPGLQTCEME---HTLAAPG-LTLTVDYSPSSCSGV-N-IRYLEVAVLTLDYHYQRRGHLEGSLTSPMGTTSLVLRHRDVDLASPPVENQDFMSVQFWGENPVGTWTFELKN--------------------------

>KEX2_Sce

QYFAVESNETEEM-----HPNWKYEHDVRGLPNHYVFSELLKLGKRSSLEGDNNDHILSVHDL--F-PRNDLFKRLPVDPLFERQWHLVNPSFP----GSDINVLDLWYNNITGAGVVAAIVDDGLDYENEDLKDNFCAEGSWDFNDNTNLPKPRLS---DDYHGTRCAGEIAAKKGNNFCGVGVGYNAKISGIRILSG---D-ITTEDEAASLIYGLDVNDIYSCSWGPADDGRHLQGPSDLVKKALVKGGRDSKGAIYVFASGNGGTRGDNCNYDGYTNSIYSITIGAIDHKDLHPPYSEGCSAVMAVTYSSGSEY---IHSSDINGRCSNSHGGTSAAAPLAAGVYTLLLEANPNLTWRDVQYLSILSAVGLEKNADGDWRDSAMGKKYSHRYGFGKIDAHKLIEMSKTWENVNAQTWFYLPTYVQSTNSTELESVITISEKSLQDAN---FKRIEHVTVTVDIDTEIRGTTTVDLISPAGIISNLGVVRPRDVSSEGFKDWTFMSVAHWGENGVGDWKIKVKTTENG-HRIDFHSWRLKLFGESIDS--

>Spo

FLVQVEPE--VDPVVAAEAIGAKYVRPLLNLKYHHLIKSDDSVQSSIRKRDAGILELERQTPR--W--------RYKRDPLFYGQWHIFNSNNP----GHDLNLREVWDAGYFGENVTVAFVDDGIDFKHPDLQAAYTSLGSWDFNDNIADPLPKLS---DDQHGTRCAGEVAA-AWNDVCGVGIAPRAKVAGLRILSA---P-ITDAVESEALNYGFQTNHIYSCSWGPADDGRAMDAPNTATRRALMNGGRNGLGSIFVFASGNGGHYHDNCNFDGYTNSIFSATIGAVDAEHKIPFYSEVCAAQLVSAYSSGSL---SILTTNPEGTCTRSHGGTSAAAPLASAVYALALSIRPDLSWRDIQHITVYSASPFDSPS-QEWQKTPAGFQFSHHFGFGKLDASKFVEVAKDWQVVNPQTWLIAPEN-VNKSFGSNETITEMVSEFTVTKDIEKFKRLEHVTVRVCIPFNRRGALEILLESPSGIRSILASERPYDENSKGFLDWTFMTVQHWAEPPEGVWKLLVNDRSGGKHEGTFENWQLALWGESENP--

>Uma

HYYVIEVHARPDPRAIAEALGAEFVERAGELQNHWLVRFPELSDTRAKRGPESQDPVLQRWSRIRRVERQEVRRRHKRDPIFTDQWHLANDRKT----GNDLNVTAIWEQGILGKGIKVCLIDDGLDMHSPDLRDNFYAPGSYDFNS--HTELPEPRES-DDQHGTRCAGEIAAVK-NDVCGVGVAYEAKVSGVRILSG---P-ISDVDEAASLNYAYQENDIYSCSWGPPDDGRSMDAPKGLIAKAMLNGGRDGKGSVFVFAGGNGGASDDQCNFDGYTNSIYSMTIAAVDREGQHPWYSEMCSAIIATSWSSGSGD--HIHTTDVVNRCTGSHGGTSAAAPLAAGVIALGLSVRPELTWRDVQHIAVRSAVKFNPEDP-DWQQTQAGHHFNHKYGYGLLDAYQFVQEAKRHKLVNPQAWYESPNTLATETLITGTESTYTVTEDHLKGA---LASVEHVTVRVWITHQRRGDVNVELISPHGTKSALARSRRYDDATTGFPGWSFMTLKHWGESPTGEWKLRVFDPAHPNRVGNIYAWSMSLWGESIDP--

>Tad3

WAVQLKEDSNTSVEYLDKQYSLKLYGRIGELANYYLYY---SSVDSHAGHVLQLNQKLQNHPFIQWHQLQENLVRHKRDPFYPDQWHLHNLRDP----NHDINVTNVWNHNITGKDVVVAIIDDGVQWKNEDLVDNYEPKGSWDVIDNDDDPVPAFAYPQTNYHGTRCAG-VASAVTNQYCGVGVAYGSKFSAIRLLDT---T-TTDAKESAAFSTKSQTNDIYSCSWGPLDDGKTVDGPRELTKKAILAGGRKKLGNIFVVAAGNGGTL-DDCNYDGFANSVYTLTIGAVTENLSPTPYSEPCSAMHAVAFGGSSSA--SITTTDWNSGCTRSFTGTSAAAPMAAGIVALMLSTNFCLTWRDVQYIIALSSVKIRAND--RWMTNTAGFHHHPLFGFGLISAWNAVKLAATWENLPPSQQWQSDNIGDSGHVLITHTSSVNVNFTGL------VYTLEHVVVSVSLEHSFRGNIEIYIRCPSGIEAKLASSRTTDNSREGFNNWEFSTVRCWGESPYGLWELRIVDTGSR-KDGVLKSWFLRLFGSTMLT--

>Cin5

WSVKISHTNHKDLEEVNKSLGMKNLGQVGELVGHYVFT----SHDYSNAD-----NALHDHSEIEWHAQQEILLRDKRDPLYKDQWHLHNKQG-----GQDCNVTGVWANNITGRGVVVAVVDDGVQWTHPDLKDNYCPEGSFDLNSDDDDPSPEPDKDDENKHGTRCAGEIAAVV-NDVCGVGIAYQARFSGIRILDG---P-MTDSIEATAFNKHMDVNDVYSCSWGPEDDGKTVDGPRSLAQIALKHGGREGFGSIFVVASGNGASKGDNCNYDGYANSIYTITIAAVDEFGYTPSYAEECTSMLASTVSSGN-GSRSICTTDWKDRCTYEHTGTSAATPLVAGMVALMLEARPCLSWRDVQHIFAMTAIPLDKKS--KWEKNSAGYTHSNNHGFGVLSSWRLVNAAKVWEPVPWLTSLKPTCGTLVIPMDSPLVVTATIFKTESNGH---LSTLEHILITVTIDHHSRGNLRFVFICPNGTRS-MIPTRQKDTSDAGLTDWTFSTVKCWGEKPFGKYQLKIFDTSKTVKAGKLHSWSLTLYGSQ-----

>Bfl7

WAVRLKPNCRDQHRLANHELSLQNLGQVGLLEDHFLLVHKTMEMLEHKMEGRRITAALRGHPCVEWSEQQRIHRRVKRDPYYSLQWHLHNQFHS----KMDLNVLNVWKKNITGSGVTVAVIDDGIEWTNPDLQDNYSPEGSFDLNSNDPDPMPEFDTKGQNHHGTRCAGEIAAVA-NSVCAVGVAFHAKISGIKILDG---P-MTDNLEAAAFNKNMHINDVYSCSWGPDDDGKTIDGPHYLARKALLYGGRQGYGSIFVVASGNGGSAGDNCNYDGYANSIYTVTIGAVDEAGGIPYYAESCSSMLAVTFSSGDFH--GIVTTDWGTGCTTGHSGTSAAAPLAAGMVALMLQVRPCLTWRDVQHIIVLTAKLINESS--EWTVNAAGFYHSHQHGFGLMDAWRLVNAAKVWEPVSWLMSLNSPT--LQEDYVFGHTVTFFYNVTKQDAD-I-LYSLEYVLVTVSISHSSRGNLELKLVCPSGTKSVIAAPRSKDKMVEDAMDGRFLNSNLSAPCPPPE--PPAYDETKAVLTGGMTVLLALYYTVEQAF--

>Bfl8

----------------------------------------MEMLEHKME-GRRITAALREHPCVEWSEQQRIHRRVKRDPYYSLQWHLHNQFHS----KMDLNVLNVWKKNITGSGVTVAVIDDGIEWTNPDLQDNYSPEGSFDLNSNDPDPMPEFDTKGQNHHGTRCAGEIAAVA-NSVCAVGVAFHAKISGIKILDG---P-MTDNLEAAAFNKNMHINDVYSCS-------------------------------------GNGGSAGDNCNYDGYANSIYTVTIGAVDEAGGIPYYAESCSSMLAVTFSSGDFH-KGIVTTDWGTGCTTGHSGTSAAAPLAAGMVALMLEVRPCLTWRDVQHIIVLTAKLINEQSS-EWTVNAAGFYHSHQHGFGLMDAWRLVNAAKVWEPVSWLMSLNSPT-LEDYVFTGGHTVKFYYNVTKQDADIH-LYSLEYVLVTVSISHSSRGNLELKLVCPSGTKSVIAAPRSKDKSKDGIKEWTFSTVRCWGEAAVGIWELLITDHGKI-----TFFWSLY----------

>Nve10

-------MNGALADRIALEAGLTNRGQIGGLSGHYLFLTNKSAIDRDELNHRAISNLLRNHPNIIWSRQEVVRKRHRRDQYFPSQWHLDNIRYV----GHDINVTGVWENNITGQGVVVSVIDDGVEWTNPDILDNYSPEGSWDINSNDEDPMPRADDAGLNHHGTRCAGEIAAVP-NTYCAVGVAYGAKVSGVRILDG---P-MTDSLEAMAFNTKMHVNDIYSCSWGPDDNGKTVDGPHQLAQAALAHGGRHGYGSIFVVASGNGGHFKDNCNFDGYANSIYTVTIGAIDELGDMPYYAEHCAAMLAVTYSSGQ-GQRNIVTTDWGTGCTDKHTGTSAAAPLAAGMIALMLQARPCLTWRDVQHVIAITAVKHDVDDD-DYHSNGANYHHSHKYGFGVMDSWRLVNTAKVWRGVPWMTSWSSPVIHVNRAVPANKLVQKYTVSKQSVME---VVTLEHVTVTVNIHHRYRGNLIVNLVSPQGTTSKLATARHHDRSSDGLNDWTFSTVRNWGESPVGTWQLVVIDNGKSIARGFVKTWRVTLYGSSMTP--

>Dre6

WAVQLQPDSLGSPDLLAQDVGLRSHGQIGQLEGHYLLC---QDPAQGEDW-RWMEAALDRHPHVAWHSQENVLRRSKRDPKFPSQWHLHNDMKR----GMDINVTGVWERNITGAGVTVVVVDDGIQHNLADIQPNYSPEGSYDLNSNDPDPMPHPDGPSDNHHGTRCAGEIAAVSNNSFCAVGVAYGSRVAGIRVLDG---P-LTDSMEAIAFNKHYQVNDIYSCSWGPDDDGRTVDGPHPLGKAALQHGGRKGFGSIFIVASGNGGQNQDNCNYDGYANSIYTVTIGAVDESGRKPSYAEECASMLAVTFSSGN-TLRSIVTSDWGTGCTSGHTGTSAAAPLAAGMVALMLQVRPCLSWRDVQHIITYTATQHDLQA--DWVTNGAGFHHSHKYGFGLLNAWRLVNAAKVWESVPFLVSYQSPVR-VNEVITTSTNLTQTWNVSESDLQ-R-MQTLEHVSVTLSIQHPRRGNLQILLLCPSGISSLIGARRALDVDSAGLTDWTFSTVRCWGERAEGQYSLLIMDDAPL-SSGILKSWKLTLYGSSLSH--

>Xtr7

WAVHLDSSGSEKMDAFAESTGLQNRGVIGELKDHYLFAIQCQEDARRKAH-----ELFTQHEGVRWYSEQKLLKRSKRDPKYPQQWHLHNARNP----GMDINVTGVWERNVTGRGVTVVVVDDGVQHTIQDIQPNYSPEGSYDLNSNDPDPMPHPDGGSDNRHGTRCAGEIAAVPNNSFCAVGVAFGSRIAGIRVLDG---P-LTDSMEAIAFNKHYQINDIYSCSWGPDDDGKTVDGPHQLGKAALQHGGRKGFGSIFVVASGNGGQFSDNCNYDGYANSIYTVTIGAVNEAGKMPFYAEECASMLAVTFSSGD-KMRSIVTSDWGTGCTEGHTGTSAAAPIAAGMIALMLQVRPCLTWRDIQHIIVFTATKYEDRQA-AWETNGAGFSHSHQHGFGLLNAWRLTNAAKVWESVPYLASYISPVKEKQIPLMP-NTLEVYWNVTTADLHSG-MKTLEHVAVTVTIAHPCRGNLEIRLFCPSGMSSLIGATRRIDMDPNGFSDWTFSTVRCWGESAEGTYRMVVSDIGDELRPGVLQQWQLTLYGSSWSS--

>Hsa6

WAVHLESLEGETLEQQADAAGLVNAGRIGELQGHYLFVHRPALEVEAIRQ--QVEAVLAGHEAVRWHSEQRLLRRAKRDPKYPQQWHLNNRRSP----GRDINVTGVWERNVTGRGVTVVVVDDGVEHTIQDIAPNYSPEGSYDLNSNDPDPMPHPDVENGNHHGTRCAGEIAAVPNNSFCAVGVAYGSRIAGIRVLDG---P-LTDSMEAVAFNKHYQINDIYSCSWGPDDDGKTVDGPHQLGKAALQHGGRQGFGSIFVVASGNGGQHNDNCNYDGYANSIYTVTIGAVDEEGRMPFYAEECASMLAVTFSGGD-KLRSIVTTDWGTGCTEGHTGTSAAAPLAAGMIALMLQVRPCLTWRDVQHIIVFTATRYEDRA--EWVTNEAGFSHSHQHGFGLLNAWRLVNAAKIWTSVPYLASYVSPVKEKAIPQSP-RSLEVLWNVSRMDLE-M-LKTLEHVAVTVSITHPRRGSLELKLFCPSGMMSLIGAPRSMD-------SWLCVECSRHQGQTKAVRECHEWKIPAR----------------------

>Gga9

WAVSLDVPEEQRAEQLARTAGLVNMGRIGELKGHYLFSRPDSHAAPEPEARRSVDTLFAQHDSVRWHSEQKLLKRSKRDPKYPQQWHLNNRKSP----GKDINVTGVWERNVTGRGVTVVVVDDGVEHTIKDIQPNYSPEGSYDLNSNDPDPMPHPDEENGNHHGTRCAGEIAAVPNNSFCTVGVAYGSRIAGIRVLDG---P-LTDSMEAIAFNKHYQINDIYSCSWGPDDDGKTVDGPHQLGKAALQHGGRRGFGSIFVVASGNGGQHNDNCNYDGYANSIYTVTIGAVDEMGSMPFYAEECASMLAVTFSGGDKMMRSIVTTDWGTGCTEGHTGTSAAAPLAAGMIALMLQVRPCLTWRDVQHIIVFTATKYEDRHA-KWDVNRAGFSHSHQHGFGLLNAWRLVNAAKIWESVPYLASYVSPVKERSIPLLP-QELEATWNVTTTDLQSG-MRTLEHVAVTVTITHPRRGNLEIRLFCPSGMMSLIGTTRSMDSDPNGFADWTFSTVRCWGEEAQGTYRLVIRDTGDQLRSGTLKQWQLTLYGSSWSP--

>Nve5

---------------------------------------------------------------------------------------------------------------------------------------------------MD--------------------------------------------------------------------------------------------------------------------------------------------------------------------------------------------------------------------------------------E-GWKRNGAGIPYNHKFGFGRLDAMRMVERAVRWTNVGKHRTCQGARHNVTRYIPSSGSLILNANTSACSGSSE-IRKLEHVQVVVTLKHRNRGDLVITLISPSGTRSELLTTRRNDQSKAGLKDWVFMTVHCWGEDPKGVWTLVITD--------------------------

>Gga6

------------------------------------------------------------------------------------------------------------------------------------------------------------------------------------------------------------------------HYQINDIYSCSWGPDDDGKTVDGPHQLGKAALQHGGRRGFGSIFVVASGNGGQHNDNCNYDGYANSIYTVTIGAVDEMGSMPFYAEECASMLAVTFSGGDKMMRSIVTTDWGTGCTEGHTGTSAAAPLAAGMIALMLQVRPCL--------------------------------------------------------------------------------------------------------------------------------------------------------------------------------------------------

>Gga7

------------------------------------------------------------------------------------------------------------------------------------------------------------------------------------------------------------------------HYQINDIYSCSWGPDDDGKTVDGPHQLGKAALQHGGRRGFGSIFVVASGNGGQHNDNCNYDGYANSIYTVTIGAVDEMGSMPFYAEECASMLAVTFSGGDKMMRSIVTTDWGTGCTEGHTGTSAAAPLAAGMIALMLQVRPCL--------------------------------------------------------------------------------------------------------------------------------------------------------------------------------------------------

>Cin2

---------------------------------------------------------------------------------------------------------------------------------------------------------------------------------------------------------------------------------------------------------------------------------------------------------------------------------------------------------------------------MQHLVVRTAKPDGL-SVDDWQQNGVGKRVSHAFGYGLMDAYGMVTLARNWTNVPQQNRCNISV--SPEETPRRSTEPLRVNVEVLVGCPNGVERLEHVQAELTLRNERRGDLTILLTSPMGTTSQLLEPRRNDISARGFTKWAFMTTHSWDEDPRGTWTLEIRDRNQRPVSGTLSLFNLVLYGTSERD--

>Hro7

-------MNREEARHIAKRNGFSYVMPVLRSKREFHFEHKLVQRARTKRS-VDLSRKLKTEPAVSAAIQQPGYLRSKRDPLYPKQWYINNTGQAGGVPKLDLNVEPAWALGFTGKGVTTAIMDDGIDYMHPDLADNFNAEASYDFSSNDPFPYPRYTDDWFNSHGTRCAGEIAAMKNNEICGVGVAYESKVAGLRMLDQ---PYMTDLIEANSMGHMPQNIDIYSASWGPTDDGRTVDGPRNATMRAIVKGGRNGSGSIYVWASGDGGP-NDDCNCDGYAASMWTISINSATNDGQTASYDESCSSTLASTFSNGKGDDAGVATTDLYGKCTTRHSGTSAAAPEAAGVFALALEANNKLTWRDVQHLTVLTSKRNHLDDHHNWTINGAGLEFNHLFGYGVLDAGDMVDMARNWITVPERWHCSADV--ITGNYPFGNPLTLTIDTTACQGQENQVNYLEHVQAFITLRATRRGDVTIHLVSPMNTTSMLLGKRVNDNSKSGFTKWPFMTTHAWAENPKGRWRLIISLDGNEPQSGTLLEWNLLLHGTRTSP--

>Cel2

FHVHLKEGGGEDAHRIAKRHGFINRGQVAASDNEYHFVQPALVHARTRRS-AGHHAKLHNDDEVLHVEQLKGYTRTKRDPLYGYQWYLKNTGQAGGKARLDLNVERAWAMGFTGKNITTAIMDDGVDYMHPDIKNNFNAEASYDFSSNDPFPYPRYTDDWFNSHGTRCAGEIVAARDNGVCGVGVAYDGKVAGIRMLDQ---PYMTDLIEANSMGHEPSKIHIYSASWGPTDDGKTVDGPRNATMRAIVRGGRNGLGSIFVWASGDGGE-DDDCNCDGYAASMWTISINSAINNGENAHYDESCSSTLASTFSNGGRNETGVATTDLYGRCTRSHSGTSAAAPEAAGVFALALEANPSLTWRDLQHLTVLTSSRNSLFDGSEWQMNGVGLEYNHLFGFGVLDAAEMVMLAMAWKTSPPRYHCTAGLDT-PHEIPADGNLILEINTDGCAGSQFEVRYLEHVQAVVSFNSTRRGDTTLYLISPMGTRTMILSRRPKDDSKDGFTNWPFMTTHTWGENPTGKWRLVARFQGPGAHAGTLKKFELMLHGTREAP--

>Aae2

FLVKFKRNVDQKAHEIADRNGFVNLGPLAGSAGRFHFKHMALPHARTRRS-IAHMRVLKKEALVNTAVQQVGFKRVKRDPYFPLQWYLRNTGQNGGKPRLDLNVQAAWDQGITGKNVTTAIMDDGVDYMHPDLKFNYNAEASYDFSSNDPYPYPRYTDDWFNSHGTRCAGEVAAARDNGICGVGVAYDSKIAGIRMLDQ---PYMTDLIEANSMGHEPHKIHIYSASWGPTDDGKTVDGPRNATMRAIVQGGRNGLGNIYVWASGDGGE-EDDCNCDGYAASMWTISINSAINDGQNAHYDESCSSTLASTFSNGAKDNTGVATTDLYGKCTTTHSGTSAAAPEAAGVFALALEANPSLTWRDIQHLTVLTSKRNSLFDAFHWTMNGVGLEFNHLFGFGVLDAGAIVSLAKKWRTVPPRYHCEAGAMD-PHPISSTGAVMLRIKTDACRGTDTEVRYLEHVQAVITANATRRGDLELFVTSPMGTRSMILSKRANDDHRDGFTKWPFMTTHTWGEYPQGTWLLEATFNSKEPRSGWIKEFSLVLHGTKDPP--

>Cin1

FLVTIKSAEPDHADWLAARHGFENRGMVVGDEGLYHFRHRTLDSASESPS-LRYIWNLRLSPKVQKVRQLEGYGRLKRDPLYPYQWYINNTGQAGGKPGLDLNVQAAWNMGYTGKGVTVAIMDDGLDYLHPDLRDNYSPEASYDFSSNDPYPYPRYTFNWFNSHGTRCAGEVSSVADNGICGVGVAYDSKIAGIRMLDQ---PFMTDVIEAASMSFKPNLIDIYSASWGPTDDGKTVDGPRQLTLRAIVNGGRNGLGSIYVWASGDGGA-DDDCNCDGYAASMWTISVNSAINDGETALYDESCSSTLASTFSNGRQRGSGVATTDLYGQCTLRHSGTSAAAPEAAGVFALALDANKNLTWRDVQHLTVLTSTPNLLHDDHRWQSNGVGLMFNHLFGFGVLNAQKMVKMAKTWTTVPPRFRCEAGVVEQMFDIPSDGVLELTIDTDACDGGNNHVRYLEHVQAFLTIASSRRGDLTINMTSPFGTDSILLNRRPNDDSSQGFRKWPFMTTHTWGEDPRGTWKLRVALNGEFPQTGRLLRWGLLLHGTQQAP--

>Bfl4

FAVQIRDGKPDTAELLARKYGYLNLGQIREQRDLYHFRHRGVPHVRRRRS-AAQQSRLENDMRVRAAVQQQGFRRRKRDPLFPKQWYLLNTGQADGKAGLDLNVLEAWEMGYTGEGVTIAIMDDGVDYLHPDLADNYNADASYDFSSNDAFPYPRYTDDWFNSHGTRCAGEVVGKINNGLCGVGVAYGARVAGIRMLDQ---PFMTDIIEASSMGHKPQEIDIYSASWGPTDDGRTVDGPRELTVQAMADGGRGGKGSIYVWASGDGGS-QDDCNCDGYASSMWTISINSAINDGRTALYDESCSSTLASTFSNGRRHEAGVATTDLYGNCTLKHSGTSAAAPEAAGVFALALEANPNLTWRDMQHLTVLTSKRNQLYDPHEWRRNGVGLEFNHLFGFGVLDAGSMVKMAQDWNTVPKRFHCTGTSMSDAKPIPVEGKVVVKLTTDACEGQENFVRYLEHVQAVVTLRSTRRGDVNINMTSPMGTQSILLSSRPNDDSVKGFDRWPFMTTHTWGEDPRGDWVLEVGFQGDEPQEGDLLEWTLRLHGTQSAP--

>Dre4

-----------------------------------------------------------------------------------------------------------------------------------------NAEASFDFSSNDPYPYPRYTDDWFNSHGTRCAGEVSAVSNNNICGVGVAYNSKVAGIRMLDQ---PFMTDIIEASSISHMPQVIDIYSASWGPTDDGKTVDGPRELTLQAMADGGRGGKGSIYVWASGDGGSY-DDCNCDGYASSMWTISINSAINDGRTALYDESCSSTLASTFSNGRRNEAGVATTDLYGNCTLRHSGTSAAAPEAAGVFALALEANPNLTWRDLQHLTVLTSKRNKLHDEHQWRRNGVGLEFNHLFGYGVLDAGGMVKLARDWKTVPERFHCVAGSMQDIHKIQSGNKLLLSISTDACQGKDNFVRYLEHVQAVITVNASRRGDLNINMTSPMGTKSILLSRRPRDDAKVGFDKWPFMTTHTWGEDPRGPWLLEVGFQSQSMQSGLLKEWTLMLHGTQSAP--

>Xtr1

FLVDLREGGEAEAKQLAAEYGFSGTRKLPFTQSLYHFYGNGKTTSRSRRS-VNKKKHLAMDPKINKVVQQEGFHRKKRDPLFTKQWYLINTGQADGTPGLDLNVAEAWELGYTGRGVTIAIMDDGIDYLHPDLASNYNAEASYDFSSNDPYPYPRYTDDWFNSHGTRCAGEVSAAANNNICGVGVAYNSKVAGIRMLDQ---PFMTDIIEASSISHMPQVIDIYSASWGPTDDGKTVDGPRELTLQAMADGGRGGKGSIYVWASGDGGSY-DDCNCDGYASSMWTISINSAINDGRTALYDESCSSTLASTFSNGRRNEAGVATTDLYGNCTLRHSGTSAAAPEAAGVFALALEANPGLTWRDLQHLTVLTSKRNQLHDEHKWRRNGVGLEFNHLFGYGVLDAGAMVKMANEWKTVPERFHCIGGAIQEPRRIPSDGKLILTLSTDACEGKENFVRYLEHVQAVITVNSTRRGDLNINMTSPMGTKSILLSRRPRDDSKVGFDKWPFMTTHTWGEDPRGTWVLEVGFVGSMPEKGVLKEWTLMLHGTQSAP--

>Xtr8

FLVDLREGGEAEAKQLAAEYGFSGTRKLPFTQSLYHFYGNGKTTSRSRRS-VNKKKHLAMDPKINKVVQQEGFHRKKRDPLFTKQWYLINTGQADGTPGLDLNVAEAWELGYTGRGVTIAIMDDGIDYLHPDLASNYSFSLLYAYKP--LQPIAYQSFLLYFSHGTRCAGEVSAAANNNICGVGVAYNSKVAGIRMLDQ---PFMTDIIEASSISHMPQVIDIYSASWGPTDDGKTVDGPRELTLQAMADGGRGGKGSIYVWASGDGGSY-DDCNCDGYASSMWTISINSAINDGRTALYDESCSSTLASTFSNGRRNEAGVATTDLYGNCTLRHSGTSAAAPEAAGVFALALEANPGLTWRDLQHLTVLTSKRNQLHDEHKWRRNGVGLEFNHLFGYGVLDAGAMVKMANEWKTVPERFHCIGGAIQEPRRIPSDGKLILTLSTDACEGKENFVRYLEHVQAVITVNSTRRGDLNINMTSPMGTKSILLSRRPRDDSKVGFDKWPFMTTHTWGEDPRGTWVLEVGFVGSMPEKGVLKEWTLMLHGTQSAP--

>Hsa2

FLVELHKGGEDKARQVAAEHGF-GVRKLPFAEGLYHFYHNGLAKAKRRRS-LHHKQQLERDPRVKMALQQEGFDRKKRDPLFTKQWYLINTGQADGTPGLDLNVAEAWELGYTGKGVTIGIMDDGIDYLHPDLASNYNAEASYDFSSNDPYPYPRYTDDWFNSHGTRCAGEVSAAANNNICGVGVAYNSKVAGIRMLDQ---PFMTDIIEASSISHMPQLIDIYSASWGPTDNGKTVDGPRELTLQAMADGGRGGKGSIYVWASGDGGSY-DDCNCDGYASSMWTISINSAINDGRTALYDESCSSTLASTFSNGRRNEAGVATTDLYGNCTLRHSGTSAAAPEAAGVFALALEANLGLTWRDMQHLTVLTSKRNQLHDEHQWRRNGVGLEFNHLFGYGVLDAGAMVKMAKDWKTVPERFHCVGGSVQDPEKIPSTGKLVLTLTTDACEGKENFVRYLEHVQAVITVNATRRGDLNINMTSPMGTKSILLSRRPRDDSKVGFDKWPFMTTHTWGEDARGTWTLELGFVGSAPQKGVLKEWTLMLHGTQSAP--

>Gga1

---------------------------------------------QVKKA-----------------VQQEGFSRRKRDPLFTKQWYLINTGQADGTPGLDLNVAEAWELGYTGKGVTIGIMDDGIDYLHPDLASNYNAQASYDFSSNDPYPYPRYTDDWFNSHGTRCAGEVSAAANNNICGVGVAYNSKVAGIRMLDQ---PFMTDIIEASSISHMPQVIDIYSASWGPTDNGKTVDGPRELTLQAMADGGRGGKGSIYVWASGDGGSY-DDCNCDGYASSMWTISINSAINDGRTALYDESCSSTLASTFSNGRRNEAGVATTDLYGNCTLRHSGTSAAAPEAAGVFALALEANLDLTWRDMQHLTVLTSKRNQLHDEHRWRRNGVGLEFNHLFGYGVLDAGAMVKMAKDWKTVPERFHCVGGSIQEPEKIPPSGKLVLTLTTDACEGKENFVRYLEHVQAVITVNSTRRGDLNINMTSPMGTKSILLSRRPRDDSKVGFDKWPFMTTHTWGEDPRGTWVLEIGFVGGVPQRGVLKEWTLMLHGTQSAP--

>Hro8

WAVKINGDE-QVARLVADRNGFRFVASV--IPGYFRFSNP-SVKKRSLTKWMFH-TKLVGDEKVIWAKQQISKRRGVRDPFSPDMWY-LNSG--------KMNIMEAYTSNVSGSGINVVVVDDGLEWDHPDLAKNYNSLISYDVTDGDADPRPVLNLGSESSHGTCCAGIIAAEANNSICMAGVAYNAKIGAIRINVF---E-MSDVEESQALTYKLHLTDIYSNSWGPSDSGQVLDRPEEMLSQAIFYGGRNGLGNIYVWASGNGGI-HDGCGADGFTNSMFTISVSATTRAHTIPAYSEPCSGIMVSAYSG------FISTTTTRKKCTQSFGGTSASGPFVSGIIALALQINKRLTWRDVQYLLITTAQPIL--KTNDWVTNGIGLKVSHFFGFGLVDALEMVRQSSSWSQVPEQHHCRSEVGYLYEYVCVRVCVCVRVCVCVCVCVCNRVLYLEHVLCVITLNAEFRGQVEIFLMSPTGTTSQLMFIRPKDLTNEGFNEWSFLSLFYWGEDPSGLWILKIKNRKTS----------------------

>Bfl10

WVVRVSGGE-TAARHVAARNGFTYLHKFDHLADHYTLLHE-RTEARSKRSDDVN-SKLASDPQVHWLSQQVAHKRVKRDPEYSRMWYLHNDGQTEGPKGLDINVIPAWVNGYTGKGIVATIVDDGFDYTHPDLKRNYDPEASFDYSDLNGNPMPVPQSDDPM----------------------------------------------------NHKRDHIDVYSCCWGPSDDGKTVQKPGELVTRILEDSGRNKRGNVYVWASGNGGSNDDDCGADGYVSSIYTIAVGAISVDGLSSYYSESCSPTMAVVPTGGEHRDSNVITTDQNHRCTDHFQGTSSAAPLATGIVALTLQANPDLTWRDVQHIVVRGAKVPNP-EEPGWNLNGADLPVHHNPDLTWRDVGAKVPNPEGWNLNGADLPVH------HKDLLAGGEVELELQTTGCHDTRDQVEVLEHVQSVMTIDHQRRGDLSIKLTSPKGTESQLMSTRSRDDSTDGFQDWPFLTVYNWGEDPSGKWKVTVKDNSGTEPVGTVSAWSLVLWGTRDE---

>Bfl6

WVVRVPGGE-TAARNVAARNGFKYLHKFDHLADHYTLIHE-KTEARSKRSADDENAKLASDSQVHWLAQQVAHERVKRDPGYSRMWYLHNDGQTKGPRGLDINVIPAWVNGYTGKGIVATIVDDGLDYTHPDLKRNYDPEASFDYSDLNGNPMPVATARDQMHHGTRCAGEVAMEANNGNCGVGVAYEAKVGGIRLITG---P-TSDAQEAASLSHKRDHIDVYSCCWGPPDSGRKVQKPGELVTRILEDSGRNGRGNVYVWASGNGGHKDDDCGADGYVSSIYTLAVGSISVDGLSAYYSESCSPTMAVVPTGGQHRDNNVITTDQGHRCTDRFQGTSSAAPLATGIVALTLQANPDLTWRDVQHIVVRGAKVPNPSER-GWNLNGAGLPVHHLYGFGMMDAGAMVKLAQEWTPVGHQRRCTVKY-DPERDIPAGGEVELELRTAGCHDTRDQVEVLEHVQSVMTIDHERRGDLSVKLTSPKGTESQLMSTRSRDDSTDGFQEWAFMTVYNWGEDPLGTWKVTV----------------------------

>Bfl9

WVVRVPGGE-TAARNVAARNGFKYLHKFDHLADHYTLIHE-KTEARSKRSADDENAKLASDPQVHWLAQQVAHERVKRDPGYSRMWYLHNDGQTKGPRGLDINVIPAWVNGYTGKGIVATIVDDGLDYTHPDLKRNYDPEAR---------------------------------------------------------------------------------------------------------------------------NGGHKDDDCGADGYVSSIYTLAVGSISVDGLSAYYSESCSPTMAVVPTGGQHRDNNVITTDQGHRCTDRFQGTSSAAPLATGIVALTLQANPDLTWRDVQHIVVRGAKVPNPSER-GWNLNGAGLPVHHLYGFGMMDAGAMVKLAQEWTPVGHQRRCTVKY-DPERDIPAGGEVELELRTAGCHDTRDQVEVLEHVQSVMTIDHERRGDLSVKLTSPKGTESQLMSTRSKDDSTDGFQEWAFMTVYNWGEDPLGTWKVTVKDNPSEGPVGTVRAWSLVLWGTRDED--

>Hma2

----------------------------------------------------------------------------------------------------------------------------------------------------DTDP--------YNAHGTKCAGTVAAVANNSICGVGIAFNAKIGAIRMLDG---K-ATDLIEADALSYHRDHIDIYSCSWGPKDNGVTFGRPGPLGRLALAQGGRKGRGSLFVWATGNGGMNKDDCNADGYVNSIYTLGIGSVNEHGVSTYYGEKCAAMIAVTYCSGASGNGVVITTYLHHQCTDSFVGTSSAAPLAAGIFALVLEANPLLTWRDIQHLVFQTAVKTSPDL--GWAVNGCGKPYNHKFGFGLLDAFALVKQALNWTLVSPQKSCHFKL-SDNGYIPSGHHFKLSFTTDGCQSCKNKITKLEHVVVNVTLKHRRRGDLSIDLISPAGTVSHMLHERPYDDSTTGLKGWTLMTLFNWCENPKGTWQLLFIDKNVSLEDEYIKELDEKKKEGTDEK--

>Cin3

WAVEIYGGN-DVADEVAAAHGFVNHGLIMKSKDFYRFSHP-KLKKRSLDSYEHHQNLLTNEK-VTWAEQQRSKRRVKRDPEWSQMWYLVPTTIP------SMRVVEAWNEGYSGKDVSVTILDDGIEHSHPDLHANYDPLASSDINSHD-------------------------------------------------------------------------------------------------------------------------------------------------------------------------DD-----------------------------------------------------------------------------------------------------------------------------------------------------------------------------------------------------------------------------------------

>Hro10

-------------------------------------------------------------------------------------------------------------------------------------------------------------------HGTRCAGQIAAVANNKKCIVGVAFKATIGGIRMLQE---V-QTDAQEAHALSFKPNFIDIYSNSWGPNDGGKIVDGPKTLTQTALLKGGRRGKGSIFIWASGNGGGPHDSCGADGYASSMYTISISGVTDKNTKPFFLEKCSSTLASAYSGGDIGMQNIVTTDIRNQCGE-HTGTSSSAPIAAALCALALQANPDLTWRDMQHIIVETSRTEPLKD-AKWVTNGAGKLVSHMYGFGLMDGLAMTQLAEQWRTVPKRHTCVGPYHRVDKYVPFQKGINFNITTNGCEDVTDDINYLEHVQVVVWLEYDFRGYLQITLESPAGTQSTLMYPRDMDYLKGAFSNWTFMSVFYWGENPRGTWKLNVTNVGLLGNGQLFVSVCLVVFCVSFLL--

>Nve11

WAVELDSDDEELAKEVARKHGFKYAGRIGELPGHFHLYRDEHDGEDSKRG-GIVYPELKEHPNVKWHEQQRTLSLHKRDPMLQDQWYLKSFGRNGVPMNNDMKVMDAWADGYTGKGIVVTVMDDGLDHTNDDL-KHYDPKASLNLNGGDKDPIPR--DEPDQYHGTKCAGEIGAKSNNGFCGVGVAPDVNLGAIRMLDG---K-VTGMTEGHAFSHNPQYVDIYSASWGPKDNGKTLAGPSSLGRKALEHGGRNGKGSLYVWASGNGGASSDDCNCDGYTSSIYTIAVGSVSAAGGSTFYDEKCPSTLAVVYSGDSLSTKKLVTTGPHNSCVEHFGGTSAAAPLAAGVVALTLQANPELTWRDMQHLITRSTDQLQK-DDPSWKRNAAGFLVSNKFGFGLLNAHKLTTNALKWKRVPDQKRCEIEGIPPVPLIKRNKEVVLRVRTNGCEGSENAIKRLEHVQAIITLAHRKRGVLSIDIRSPRKTASRLLSTRPLDESASGIKNWPFMTVQMWGEDPKGEWEVVIRDN-------------------------

>Nve7

WAVQLDSA-EEAADRIAAKHGFTNLGQVGSLKGYYHFKHVHKPGNRARREVHEKTRLLMQEDEVLWAQRQHVLARTRKDPKFHDQWYLFNDGQSTGPAGVDIDVVPAWNRNITGRGVVVSILDDGVDHTHPDLRDNFDQKASYDFNDMDPDPRPR-DSDPDNCHGTRCAGEVAA-ANNDVCGVGVAYNANIGGVRMLDG---Q-ATDVLEGSSLSFQSAYIDIYSNCWGPKDDGKTFGKPGKLAQEALMQGGRGGRGNIYVWATGNGGLTDDDCNCDGYTTSIYTISIGCIGDHGLSAYYTELCSSTLGVTFNGGSHRERKMVTTDLHHKCTEEFKGTSSAAPLAAGMIALVLEANPNLSWRDVQHLVVETAQVTSPVDE-GWMKNGAGYHFNHKFGFGRLDADAMVKRAKTWKSVAPQRICHGPSST-QQEIPTGGTLSITIDTIACSGTDKMLTKLEHVTLTVSFQHRRRGDVSIDLFSPSGTRNEMLSTRRYDDSKNGLHDWTFMTVHNWGENPKGEWVMNVTDN-LSL-NKGVDPNGYVQHDTSKQE--

>Nve8

WAVEIEHPFEKYVREISKRHGFTIIGKIGNLKGHYHLRDELNKEHNRDKT-----DRLLLEDNVRWAEQQKILHRVRKDPMFTEMWYLMNTGQTGGPVGVDINVIPVWKRGITGRGVVVTVLDDGMDHNHPDLKPNYEPCASHDFNDNDQDPAPRDIDP-DNCHGTRCAGEIAAVANDSICGAGVAYNAKIGGVRMLDG---K-ATDALEACSLGFANQVTDVYSNCWGPKDDGKTFGKPGPLAAKALKSGGRGGKGNIFVWATGNGGLTDDDCNCDGYTTSIYTVSIGCIGDHGLSAYYTELCSSTLAVTFNGGAREEEKMITTDLKGKCTEQFKGTSSAAPLAAGMVALMLEQNPSLTWRDVQHIIVHTAKMTSPDD--GWRTNGAGLHFNHKFGFGRLDADAMVEKSRVWKNVPVQRTCTGASVTDEKDIPVGGSLDIQIPTGGCMGTIAEINHLEHVVLTVSFIHRRRGDVSLLLTSPSGTKNEMLSTRRYDDSKEGLDKWSFMTVHCWGENPRGFWKLNVIDNPLNIGDRVVNGFGTLHLDTSKQD--

>Nve6

WAVQLDSNDGSLADAIARAHGFKNLGGIGTLTGHYEFVHD-STGSRMRRSESRT-KRLISHPRVIWAKQQRILDRQKRDPMFAKQWYLQNTGQFNIPEGNDIGVLPVWERGFTGKGVVVSILDDGLDHTHPDLKRNYDPKASWDFNDKDDDPFPN-DVDPYNAHGTKCGGEVGAQADNDICGAGVAPNVSLGGIRMLDG---V-ATDALEANALSYKPQYIDIYSNCWGPKDDGKTFGRPGKLGQKALEDGGRGGKGSIYVWATGNGGLVDDDCNCDGYTSSIYTISIGAISSYGLSTYYDEQCSSTMAVTFTGDSRSEETLVTTNLHHECTDTFRGTSSAAPLAAGIFALVLEANPNLTWRDLQHLVVHSAEKTSP-LDQGWKVNGAGIHFNHKFGFGRLHATRLVANALKWKHVPAQHICQVEGQARKEIIKRNGKLILKVHTDGCAGTKNAVKRLEHVQATVSLKHNRRGALSIEIRSPMGTTSQLLSTRKYDTSTNGLKDWSFMTVHFWGEDPAGEWEVIITDNTDS------------MFNKRE----

>Lgi9

FAVQCKDGR-QTIDELVESYGFKIERELGDIGVFLSHP---TVHYRSKRSSNSILNKLQNHPQVIHAQQEQELLRVKRDPLYKDQWYINNVGQSGGVKDVDLNVMVAWKQGFTGTGVVISILDDGVDHTHKDLLTNYDPDASTDLNGHTDDPMPNVTTH-ANAHGTRCAGSAAAYANNNVCGVGIAHTAKIGGVRILDG---R-VTDSLEAAALNFNIQHIDIFSASWGPTDDGATMQAPKHFTKAALRKGGRKGKGNIYVWATGNGGANDDDCSADGYVSSIETLSIGSISDKGRKPFFMENCTSTIAVVPSGGEVKEEKVVTTDIKNGCIENFQGTSSAAPLGAGCIALLLQANGDLRWRDVQHIVIQSSKIPSLDT--SWIINGAGLHVSHKFGYGVMDCGKMVELAQHWNNVPEQHTCSYNR--SDSNIKIRSSIETQIYVDGCKDSDSEIDRLEHVQIHVKITHSRRGDIQIFLTSPAGTRSEMLSPRPHD-DFKGKWEFTFMSVHTWGEHPNGLWKLEINDNPTSTNEGYLSDWSLIMYGFSGSR--

>Tad

WIVKIPGGR-KRADEVAHKLGYTNLGRISDL-DLYNFRHD-DVPRRSKRAHDRT-RRLAEDDNVEWVEQELIKYRYKRDYLWPKQWYLHDKRDA----RFDINVLPVWQKNITGKGVVVTILDDGLEHSHPDIKYSYDPEASYDYNDYDNDPEPRYDSRSSNRHGTRCAGEITMKPNNKMCGVGVAFGARIGGIRMLDG---S-ITDGVEASSLGHNMQHVDIYSASWGPNDNGRTVDGPGDIVMKILRKGGRGGLGSIYAWASGNGGRRGDNCNCDGYVNSIYTIAVSSASYNGTPPWYAEDCTPALASTYSSGADALKKVASIDLRGGCTTSHTGTSASAPMAAGIYALVLEANPNLTWRDVQHATVWTSNRKNL-TKSGWARNGASRLYHHKFGFGILDAAALVDIVDVWKTVPEQKFCPLTADKIEREISSEGIVRIQIKSHGCQDSKDSINYLEHVQLISTIDFSRRGDLDITLISPSGTRSNLLSRRLRDSSASGFNSWPFMSTHFWGENPRGTWTVEIKNVGMTNEHGFVRETMLVFYGTETMP--

>Lgi

-----------------------LFFQLRGFDDNYLLRHK-DVPSRSRRSADHHTKRLIDDSRVEWADQQYTKIRVKRDPLWEKQWYLHDDREDLSESKKDLHVIPAWKKGYTGKGITVSILDDGLERLHTDLADNYSPEASYDYNGDDDDPTPRYDPTNENKHGTRCAGEVAMIANNGKCGVGIAFNAKIGGIRMLDG---E-VTDTLEGQALQHAIDVVDIFSASWGPNDDGNTLEGPGKLAHKAFENGGRKGKGVIYVWASGNGGRLYDNCDCDGYTGSIYTLSISSASQVFKRPWYGELCASTMGTTYSSGSIDDR-VISTDLNDTCTKSHTGTSAAAPLAAGIIALLLESNPDLTWRDVQHLVTWTSQAGPLIQQSGWTRNGAGFLVNTAFGFGILDTAGLIDAAETWKSVPKQMKCTINSSK--SNLPHNQRLEIQIYSSGCEGQENEINYIEHVQLHLTLDYTKRGAISVHITSPSNTTTMLLSERPYDKSTKGFQSWPLMSVHNWGEDPKGTWKVVLEDTTDDKNNGILQDLRLVIYGTTEQP--

>Lgi7

-----------------------LFFQLRGFDDNYLLRHK-DVPSRSRRSADHHTKRLIDDSRVEWADQQYTKIRVKRDPLWEKQWYLHDDREDLSESKKDLHVIPAWKKGYTGKGITVSILDDGLERLHTDLADNYSPEASYDYNGDDDDPTPRYDPTNENKHGTRCAGEVAMIANNGKCGVGIAFNAKIGGIRMLDG---E-VTDTLEGQALQHAIDVVDIFSASWGPNDDGNTLEGPGKLAHKAFENGGRKGKGVIYVWASGNGGRLYDNCDCDGYTGSIYTLSISSASQVFKRPWYGELCASTMGTTYSSGSIDDR--ISTDLNDTCTKSHTGTSAAAPLAAGIIALLLESNPDLTWRDVQHLVTWTSQAGPLIQQSGWTRNGAGFLVNTAFGFGILDTAGLIDAAETWKSVPKQMKCTINSSK--SNLPHNQRLEIQIYSSGCEGQENEINYIEHVQLHLTLDYTKRGAISVHITSPSNTTTMLLSERPYDKSTKGFQSWPLMSVHNWGEDPKGTWKVVLEDTTDDKNNGILQDLRLVIYGTTEQP--

>Lgi6

MTGFGVTTVRITREVTEALVETMWQNCLRGFDDNYLLRHK-DVPSRSRRSDHHT-KRLIDDSRVEWADQQYTKIRVKRDPLWEKQWYLHDDREDLSESKKDLHVIPAWKKGYTGKGITVSILDDGLERLHTDLADNYSPEASYDYNGDDDDPTPRYDPTNENKHGTRCAGEVAMIANNGKCGVGIAFNAKIGGIRMLDG---E-VTDTLEGQALQHAIDVVDIFSASWGPNDDGNTLEGPGKLAHKAFENGGRKGKGVIYVWASGNGGRLYDNCDCDGYTGSIYTLSISSASQVFKRPWYGELCASTMGTTYSSGSAIDDRVISTDLNDTCTKSHTGTSAAAPLAAGIIALLLESNPDLTWRDVQHLVTWTSQAGPLIQQSGWTRNGAGFLVNTAFGFGILDTAGLIDAAETWKSVPKQMKCTINSSKSNLPQNLNQRLEIQIYSSGCEGQENEINYIEHVQLHLTLDYTKRGAISVHITSPSNTTTMLLSERPYDKSTKGFQSWPLMSVHNWGEDPKGTWKVVLEDTTDDKNNGILQDLRLVIYGTTEQP--

>Lgi10

-----------------------------------------------------------------------------------------------------------------------------------------------------------------------------MIANNGKCGVGIAFNAKIGGIRMLDG---E-VTDTLEGQALQHAIDVVDIFSASWGPNDDGNTLEGPGKLAHKAFENGGRKGKGVIYVWASGNGGRLYDNCDCDGYTGSIYTLSISSASQVFKRPWYGELCASTMGTTYSSGSAIDDRVISTDLNDTCTKSHTGTSAAAPLAAGIIALLLESNPDLTWRDVQHLVTWTSQAGPLIQQSGWTRNGAGFLVNTAFGFGILDTAGLIDAAETWKSVPKQMKCTINSSKSNLPQNLNQRLEIQIYSSGCEGQENEINYIEHVQLHLTLDYTKRGAISVHITSPSNTTTMLLSERPYDKSTKGFQSWPLMSVHNWGEDPKGTWKVVLEDTTDDKNNGILQDLRLVIYGTTEQP--

>Hro

--------------------------------------MSHDQVIRSRAQ-------------IKLAELQRPLKRYKRDDFWENQWYLHDTKPARMLPKIDLHVIPVWANNITGRGVVVTIIDDGLEHNHTDLHRNYDPKASWDFNDNDSDPFPRYDESDENKHGTRCAGEVAMVENNNFCAVGIAFNANIGGIRVLDG---V-TTDRLEALSLSYNVDHIDIMSASWGPSDDGKSLEKPGMLAQAALEKGGRGGKGVIYVWASGNGGISKDNCNCDGYTGSIYTLSISSASEWFNSPWYAEHCPSTIASTYSSGV-YEQRVISTDLHNQCTTAHSGTSASAPLAAGIIALVLEANPNLTWRDVQHLVAWTSEYEPLRKNKGWMVNGAGFWVNNRFGFGLLNAAMLVEAADVFKSVPKQSLCIAMPPSLPRKLMSKTSLEVAVWSDACETADNLVNYIEHVILVLDMNYTRRGNVQVHLKSPSGMVTEMLTVRKTDNATTGFPKWPLSSVHNWGEDPRGLWITTIVDNVEGNHWGYLKGLQIQFYGTSQIP--

>Hro9

------------------------------------------------------------------------------DPHFKDQWYLLGGVV-----GHDLNVYPAWVKGYTGKGVVVSILDDGIDHSHPDLQKNYDPEASADLNDRSNDPMPNKLNP-YNNHGTRCAGEVGAQANNSNCCVGVAFNSKIGGIRMLDG---S-VTDLLEGESLLYNNQHIDIYSASWGPNDDGRTMEAPNRYCRLALEEGGRKGLGSIFVWASGNGGMFQDDCSCDGYVSSIHTLAIGSVTDEGMSTYYGEVCPSVMGVVFAGVYRV--KIIWLSLFQCCFH------FSFPNLNDKRKKNTHISANLTWRDVQHLVVETSVIPSEKED-GWKVNAAGYHVNHRFGFGVLDCGRMVEAAENWIGIQEQLKCVVKSGPRILYKDSTEVLSTTSTSSDCNHNSRAIDHLEHVQIHIKLVHRRRGDVTISLTSPQETTSNILKVRPLDRSGNGI-DFVFMTVHSWGENPSGNWTLTIKDNS------------------------

>Mbr

WAVHVPHGE-AHARDIARRHGFLFHGQIGGLDGYYHLENASGAHCVPRHESHHLTRRLVDEEHVGWAEQQKVLRRSRRDPMFEDQWYLYNSKRD------DHNVIPVWQRGITGKGVVVTIVDDGIEYTHADLKANYDPKASHDMNGRDNDPMPN-ELDPINRHGTRCSGQVAA-GKNKVCGTGIAYDANIGGVRMLDG---A-VTDAVEAGSLGLNPGYIHIYSNSWGPNDDGRTLEGPGPLARKALENGGRQGKGSIFVFASGNGG-SSDSCNCDGYTNSMYTISIGALDESNSEPYYNERCASTHAVAYSSGS--GRSISTVDLHNGCTRSHTGTSAAAPSAAGFIALALSANPDLTWRDMQHIIINTARKVNP-YDTTWTENGAGFKHSDKFGFGLIDAEKLVDAALAWRTVGPQLQLEQSKANHALETSAVGTITVEATQTG-------ITTLEHVQVHVLCDAYKRGTVTISIESPTGVVSELLPYRSHDYEHSGI-DWTFMTLRHWGESPIGTWTLRANVKGSS--TALLKTWKLIAYGTGSAG--

>Ppi

WSIIIRPG--HSPHDIARDLGLEYVGPIGQIPFHHHFKHRPSPRKSPTDS-GHVTRTMLAHLGVISAVQQKLLSRKRRDPLYSAQWFLQAKYN---------NVTGAWAQGVSGEGVVVTVLDDGLEYAHPDLEANYEPLASWDMNDNDRDPAPRYDPSNENKHGTRCAGEIASVADNSYCGVGAAYKTKIGGIRMLDG---D-VTDIVEASALGLNPDIVDIYSSSWGPDDDGQTVDGPGKVTEKTFKLGGRRGLGSIFVWASGNGGATWDNCNCDGYTNSIWTISIGAVSMRGEKPWYAEECSSTIAVTYSSGS-HEPKIVSTDLHSQCTEAHTGTSAAAPLAAGILALTLHANPGLGWRDTQHLLVRSSKVRDTVG--DWARNAAGHEFSHQFGFGVIDAGGLVEMARRWTNVGPKLECDGSI-SPAQVIPARSTLNFTSRATGCG-----IKHLEHVQAVVTIAASRRGDISLTLISPSNTPSQVLNSRPKDPSGKGFSEWPFMSVHFWGEPAMGTWTLSVTNSGDG--SMKLQRWWLVHHGTEVLP--

>Hro5

------------------------------------------MKKRSLSSSYHQ-NLFKQNPKVIWLEQQKVKIRMKRDPLWPNMWY-MNEKVHP-----SMNIMDAYKRNMTGDGIAITILDDGIEKNHTDLFANYDPQASYDFNDNDTDPHPRYDFTDFNRHGTRCAGEVAAIANNNKCGVGIAYKAGIGGIRMLDG---D-VTDALEAQALSYHLQHIDIFSASWGPTDDGKTLDGPGTLAQKAILMGGRNGLGTIYVWASGNGGRHGDNCNCDGYTNSMYTLSVSSVTSTYEQPDYLERCSSTLAATYSSGSDRRTQIITTDLHDGCTDTHSGTSASAPIAAAIIALALQKNKFLTWRDVQHLVVRSSRRSTL-KSDDWILNGAGRWFSHAYGYGLMDASKLVELAHIWKTSPKRISCFFAPVITNRTVHGGLETANVVNVSSCTDNNEVVKYIEHVQAQITFSTAKRGDCELHLLSPAGTRSKLLMKRPLDTSSEGIINWPFTSTHFWGENPEGVWSLTAMCSKEN---GELTNWVLSIHGTSIDP--

>Dre1

WAVEIPGGV-QNARSIADEFGYQLVRQIGALENHYLFK-RHSHPSRTKRSADHITKRLSEDDRVSWAEQQYEKRRAKRDPMWNQQWYLQDTRTSSSLPKLDLHVIPVWKKGITGKGVVITVLDDGLEWNHTDIYPNYDPAASYDFNDNDPDPFPRYDSTNENKHGTRCAGEIAMQADNNKCGVGVAYNSKVGGIRMLDG---I-VTDAIEASSIGYNPDHVDIYSASWGPNDDGKTVEGPGRLAQKAFEYGGRGGKGSIFVWASGNGGRQGDNCDCDGYTDSLYTISISSASQQGLSPWYAEKCSSTLATAYSSGD-YDQRITSADLHNECTETHTGTSASAPLAAGIFALALEQNPDLTWRDLQHLVVWTSEFDPLANNPGWKRNGAGLMVNSRFGFGLLNAKALVDLADVWKHVPEKKQCIVRDETQPRPLKAAGEISIEIPTKACAGQANSVMSLEHVQVEVSIEYTRRGDLHITLTSPSGTTTVLLAERERDTSSNGFRNWAFMSVHTWGENPTGTWILKITDTSGRENEGQIISWKLILHGTSEKP--

>Xtr4

WAAEIPGGP-EEAQLLALELDYDYLGQIGSLENHYLFRHK-AHPRRSRRSAPHLTKRLADDGRVIWAEQQYSKERNKRDPLWNRQWYLQDTRSNPSLPKLDLHLLPVWKKGITGKGVVITVLDDGLEWNHTDIYANYDPEASYDFNDNDNDPFPRYDITNENRHGTRCAGEIAMVANNNKCGVGVAYNAKVGGIRMLDG---V-VTDAKEASSIGFNPQHVHIYSASWGPNDDGKTVEGPGRLAQKAFEYGGRNGKGSIFVWASGNGGRQGDNCDCDGYTDSIYTISISSASQQGLSPWYAEKCSSTLATAYSSGD-YDQRIISADLHNECTETHTGTSASAPLAAGIFALALEFNPDLTWRDMQHLVVWTSEYDPLANNAGWKKNGAGLMVNSRFGFGLLNAKALVDLADTWKGVAEKKECIV----EDNEFSPDGEVTIQIPTKACEGQDNHIKSLEHLQLEATIEYTRRGDLHITLTSPLGTNTVLLTERERDTSPYGFKSWDFMSVHTWGEDPAGTWTLKISDVTNGKTIGML----------------

>Gga4

WAAEVPAGP-DAARAIAEELDYDLVGQIGSLKNHYLFRHK-SHPRRSRRSAIHITKRLSDDERVSWAEQQYEKKRTKRDPMWNQQWYLQDTRITPSLPKLDLHVIPVWQKGITGRGVVITVLDDGLEWNHTDIYANYDPRASYDFNDNDYDPFPRYDPTNENKHGTRCAGEIAMQANNRKCGVGVAYNSKVGGIRMLDG---I-VTDAIEASSIGFNPEHVDIYSASWGPNDDGKTVEGPGRLAQKAFEYGGRNGKGSIFVWASGNGGRQGDNCDCDGYTDSIYTISISSASQQGLSPWYAEKCSSTLATAYSSGD-YDQRITSADLHNECTGTHTGTSASAPLGVG---LALEYSPNLTWRDMQHLVVWTSEYDPLAGNPGWKKNGAGLMVNSRFGFGLLNANALVDLADRWKGVPEKRECIVQDSFEPRLLRANEEVIIEIPTKACEGQENSIASLEHVQLEATIEYSRRGDLHVTLVSPSGTSTVLLAERERDKSPNGFKNWDFMSVHTWGENPTGTWVLRITDVSKR-NEGRIVNWKLILHGTDTQP--

>Hsa3

WAAEIPGGP-EAASAIAEELGYDLLGQIGSLENHYLFKHK-NHPRRSRRSAFHITKRLSDDDRVIWAEQQYEKERSKRDPMWNQQWYLQDTRMTAALPKLDLHVIPVWQKGITGKGVVITVLDDGLEWNHTDIYANYDPEASYDFNDNDHDPFPRYDPTNENKHGTRCAGEIAMQANNHKCGVGVAYNSKVGGIRMLDG---I-VTDAIEASSIGFNPGHVDIYSASWGPNDDGKTVEGPGRLAQKAFEYGGRQGKGSIFVWASGNGGRQGDNCDCDGYTDSIYTISISSASQQGLSPWYAEKCSSTLATSYSSGD-YDQRITSADLHNDCTETHTGTSASAPLAAGIFALALEANPNLTWRDMQHLVVWTSEYDPLANNPGWKKNGAGLMVNSRFGFGLLNAKALVDLADTWRSVPEKKECVVKDNDEPRALKANGEVIIEIPTRACEGQENAIKSLEHVQFEATIEYSRRGDLHVTLTSAAGTSTVLLAERERDTSPNGFKNWDFMSVHTWGENPIGTWTLRITDMSGRQNEGRIVNWKLILHGTSSQP--

>Cin4

FAVEIPPD--RDANEVARNHGFMNLGQVGNLEGWYIFKDH-RIRKRSLEAHRDTPKLKRLKRDVIMVERLRVLKRVKRDPSWDEMWY-MHCGNNRSQSPSSMRIEEAWIAGFHGEGVVVTILDDGLETTHPDLIRNYDRFASIDINDRDTDPTPRYDPSNENKHGTRCAGVVAAVLDNHICIVGVAHGASIGGVRMLDG---E-VTDRVEAHSLNFAQDRIDIYSASWGPDDDGETVEGPGPLAKAAFKSGGRGGNGSIFVWASGNGGHNKDSCSCDGYINSIYTIGISSVSERGNRPWYLEGCASTLATTYSSGEINEGKVITTDLHRRCTHEHTGTSASAPMAAGIIALMLQANMALTWRDVQHVIVRTTKSQGL-HGHDWVVNGAGFNVSHVFGFGLLDAAALTHVAGRWKRVPEQHECITIPQRLALEIVPREVTEVTMDTRACHRKTSEVLYLEHVVLRITLSHPRRGSLKIFLVSPSGTVSNILQRRDYDRSSGGFNDWEFMTTHHWGENPMGVWLLRIEDIPYRPRRGMLRKWQLLFYGTQTHP--

>Nve13

WAVHLDNADPDSVEKIATRHGFVNLGQIGSLPGYYHFVKRSTEMRRRRSL-DEHADRLQDEPQVTWVEQQRILERSKRDPLYKNQWYLQNVGQSSGPAGIDINVLPVWAQGYSGKGVVVSVLDDGVDYTHPDLKRNYDPDASFDFNDFDADPKPLDKNS-QNSHGTKCAGEVAAEANNGICGVGVSFNASIGGIRMLDG---K-LTDTIEGSALSYRSDYIDIYSSCWGPKDDGKRFGKPGILASKALQIG---------------AERSNEEL-------LIFRLS----------------------------------------------------------LALFTL----------------------------------------LALVTGYVILDE--------------------------------------------------QARQLYHLE-------------------------------------------------------------------------------------------

>Nve12

---------------------------IGSLPGYYHFVKRSTEMRRRRSL-DEHADRLQDEPQVTWVEQQRILERSKRDPLYKNQWYLQNVGQSSGPAGIDINVLPVWAQGYSGKGVVVSVLDDG--------------------------------------------------------------------------------------------------------------------------------------------------------------------------------------------------------------------------------------------------------------------------------------------------------------------------------------------------------------------------------------------------------------------------------------------

>Bfl

WALHVEGGT-AAADRLAFKHGFVNKGQIGSLEDHYLFVHR-RTWKRSLRSSHRH-ALLQREPEVRWLQQQVVKRRVKRDEKWDKMWY-LHCDRPDFACSSDMNVEAAWKKGYTGKGVVVSILDDGIETDHPDLAGNYDPDASSDINGGTLDPTPRYEYTNENRHGTRCAGEVAAMGNNSFCSVGVAYKASIGGVRMLDG---D-VTDSVEAASLGLNPQHIMIYSASWGPDDDGKTVDGPANLAKKTFQAG-----ASKLCYVQCNGGRTHDSCGCDGYTNSIYTISVSSASEQGKVPWYLEPCASTLATTYSSGAPHERKVITTDLRKGCTESHTGTSASAPMAAGICALALEANPMLTWRDLQYIVVMAANPTPLDKASDFITNGAGLRVSHNFGFGLMDAGKMVELAESWRKVPEQHVCEEDPNAQQRAITRGETIIDTKTTGGCNGTDH-VKYLEHVVVEISLDHPCRGHLSIHITSPSGTRSTLLPERQFDSSSDGLKDWAFMTTHCWGEQPDGDWILEVKDLGQQTVLPVLRKWKLILYGTAEHP--

>Bfl3

WALHVEGGT-AAADRLAFKHGFVNKGQIGSLEDHYLFVHR-RTWKRSLRSSSHRHALLQREPEVRWLQQQVVKRRVKRDEKWDKMWYLHCDRPDFACQSSDMNVEAAWKKGYTGKGVVVSILDDGIETDHPDLAGNYDPDASSDINGGTLDPTPRYEYTNENRHGTRCAGEVAAMGNNSFCSVGVAYKASIGGVRMLDG---D-VTDSVEAASLGLNPQHIMIYSASWGPDDDGKTVDGPANLAKKTFQAG-----ASKLCYVQCNGGRTHDSCGCDGYTNSIYTISVSSASEQGKVP--------------------------------C-------------------------PMLTWRDLQYIVVMAANPTPLDKDSDFITNGAGLRVSHNFGFGLMDAGKMVELAESWRKVPEQHVCEEDPNAQQRAITRGETIIDTKTTGGCNGTDHHVKYLEHVVVEISLDHPCRGHLSIHITSPSGTRSTLLPERQFDSSSDGLKDWAFMTTHCWGEQPDGDWILEVKDLGQQTVLPVLRKWKLILYGTAEHP--

>Hro2

WAVQIEGGI-AEAKNVAQRHGFELVNEI--MPDFYLLRNK-RISKRSLVSDSYA-NKLLSDAQVEWVEQQVVLKRVKRDPKWPRMWY-LNRGNGL-----DMNIIPAWRSGITGKGVVVCILDDGIEKDHPDIRKNYDPEASYDVNDQDPDPQPRYEYSNENRHGTRCAGEVAAEANNNICSIGVAYEARIGGIRMLDG---D-VTDAVEAQSLSWKRQHISIYSSSWGPDDDGVTVDGPAPLAWRALVEGGRRSLGSIFIWASGNGGNNSDSCNCDGYTNSIYTLSISSATEFGDVPWYSEACSSTLATTYSSGSGTERQIVTTDLRRGCTEGHTGTSASAPIAAGICALVLQASPYLTWRDMQNIVVLTSRPESL-NANDWTKNALGRKVSHHFGYGLLDVAAMINLAQNWTTLPPQRQCHLTYGNGIPGRGAFGRLELELLSEGCRGTPNKVSYLEHVQAIITLSTSNRGEVQIFLTSPSGTKSTLLSQRPKDTSAEGFTNWAFMTTHCWGESSTGTWKLEVTAGSSI----------------------

>Hro3

WAVQIDGNE-TVAKSLASKHGFFYITRI--LENYYLFRHP-RIAKRSLRETSDHLSSLQHENKVKWIEQQRVRRRVKRDNKWPKMWY-LNGAVNF-----DMNLLRAWEAGYTGEGVVVTVLDDGIEKDHPDLRNNYDEDASYDVNDQDPDPQPRYEHSNENRHGTRCAGEIAAEANNGICSVGIAYRCRIGGVRMLDG---D-VSDAVEAQSLTLHPHYIDIFSISWGPDDDGKTVDGPGPLARKAFSDGGRKGLGSIFIWASGNGGKEGDGCNCDGYTNNIYTLSISSATETGQVPWYSEACSSTLATTFSSGTNTDRQIVTTDLRKGCTEGHTGTSASAPLAAGVCALVLQANSGLTWRDLQYIIVLTAQPNNL-LAEDWQTNGVGKLVSHYFGYGLLDAHTMIRLALNWSNVPEQHRCEIVSKNSHRNIPMYSQIENILYSDGCHDDKLHVQYLEHVIVTITLTSTRRGELEVYITSPRGTRSTLLARRPVDSSAEGFNNWSFMTTHCWGETSEGAWKLEVKNSGSY---AKLVSWSLVLHGTEYYP--

>Hro4

WAVKIEGGV-GVAKYIARKNGFEFVTEI--MPNYYLFKHR-RLSRRSIMPQIFE-SKLARDSRVNWVEQQTILKRVKRDPKWPQIWY-LNRESAI-----DMNVVPVWKSGITGKRIVVTILDDGIEKDHPDLEQNYDARASYDVNNQDDDPQPRYEQTNENKHGTRCAGEVAAAANNKICGVGVAFEASIGGIRMLDG---E-ITDAVEAQSLSYKPNYISIYSASWGPDDEYITVDGPAMLANRALYEGGRNYSGSIFVWASGNGGKFGDSCNCDGYTNSIYTLSISSATERGKIPWYSEACSSTLATTYSSGEDLDKKIITTDFKKGCTESHTGTSASAPLAAGICALVLQANNHLTWRDIQHLVVATSRPANL-ESNDWVTNGAGKKVSHYFGYGLLDAYAMVEMGRNWTNVPPQKKCQIIYRGNEMGLPVNGIISLELSTDNCQINNNPITSIEHVQAVITLSASNRGQVQIDLKSGMSTNTTLLHRRVKDTSNEGFTNWAFMSTHFWGEKLGSTWELTVYNGNSV---STLKNWTLVLYGTGSST--

>Nve2

WVIYTDKGQ-EYVDNLAAKHGFKSHRDGGGLEGFYILEHQ-RTSKRMRRSLVHHSTNFLRDPHVSYAKQQKILRRQKRDPLFNDQWYLNNYGQTPGPKGLDINVLPVWRKNITGKNVVVTILDDGIEYTHPDLQQNYDKEASYDYNHYDSDPFPRYSPDNINKHGTRCAGEVAAIK-NTHCGVGVAYNARIGGIRMLDG---D-VTDIVEGKSLSLKTGYIDIYSSSWGPDDDGRTVDGPGPMAKRAFRDGGRRGLGSIFVWATGNGGRYNDYCNCDGYITSIYTISIGAINDKGKSPWYAENCPSTLGVTYSSGQNGDLQIVTTDLHHKCTKEHTGTSAAAPLAAGIFALVLEANPKLTWRDLQHLVVNTSKKTDAGDS-EWITNGAGHHVNNKYGFGVLDSAALVELAQKWRTAEEQHVCREPGSS-SQEIPKNGELTLTLDATGCSGKSNCVTRLEHVRVYVTLRHDRRGAISIVLISPSGTRSDLLKQRSKDFSNNGFKNWPFMTVFSWNENPVGKWKLVVRNHAST--AGTFDRWFIKFFGTCQRP--

>Cel3

WAVRIAGGKVEEANRLANKYGYTNLGPIIPGDEYYLFRDDRKKSRSSRKTSLSA-NQLQHEEDVMWMEQQVAKRRVKRDPLWTDMWY-LNRGEHHSDSRMDHNVKEAWDLGYTGKGVVVTILDDGLERTHPDISPNYDERASYDVNDRDNDPMPRYEFSDENRHGTRCAGEVAAIFNNSLCIVGIAYNANIGGIRMLDG---D-VTDAVEAASVGHNADYIDIYSASWGPDDDGRTVDGPAKLTRSAFEKGGRKGKGSIFVWASGNGGKDADSCNCDGYTNSIYTLSISSATENGNIPWYSEACSSTLATTYSSGATGEKMILTTDLHHACTNMHTGTSASAPLAAGIVALALEANPNLTWRDLQHIVIRTAKPINL-RAGDWTTNGVGRNVSHSFGYGLMDAGAMVKLAKIWKKVDEQHRCRQFYPSRYKNIPNGNRLQLQLYSDGCYGGADEVSYVEHVQAIVTLKAPKRGDLQIYLTSPSGTKSTLLTKRARDTSRSGFTDWAFMTTHNWGEQAAGLWILEIDNDGWD--DAELVKWELVLYGTDRET--

>Xtr2

WAVQVPTGP-KEVERIAKKLGFISLGQ------------------------------------VHWFEQQTINQRYKRDPCFWKQWY-LNNDVKP-----NLGVLTAWSQGYTGAGVVVTVLDDGIEKDHPDLSANYDPMASYDFNSNDPDPQPRYNPSDENRHGTRCAGEVAAAAYNNICGAGIAFNSKIGGIRMLDG---L-ITDVIEAQSLSLNPQHIHIYSASWGPEDDGKRVDGPGPLAEEAFFWGGRGGLGSIFVWASGNGGLQYDNCNCDGYTNSIYTLSVGSTTEHGNVPWYSEACASTLTTTFSSGISTERKILTTDIRLRCTDQHSGTSASAPLAAGIIALALEANPALTWRDLQHIVVRASNPSNL-SAEDWAVNGVGRKVSHYYGYGLLDAGRIVDLAQKWQTAGVQRICVVKVLTTPQVL--TSNHIVRQHVDGCTGTSS-IQSLEHVQAKISLSYSRRGDLEISLISPMGTRSVLVALRPYDTSTKGYKNWTFMSTHTWDEKPQGTWTLKFVNKGDFTNTGFLHDFTLVLYGTDEDM--

>Bfl2

WAVEIYGGP-EKADLLAREHGYENLGQIGNLEDHYLFRHK-DVPHRSRRGAHQHTKRLGDDER-------------------------HDTRTSTNLPKLDLHVLPVWRKGITGKGIVVAVLDDGIEKDHPDLVDNYDPDASFDFNDNDADPQPRYEETNENKHGTRCAGEIAMAANNSECGVGIAFNARIGGVRMLDG---V-VTDAVEANSIGFNIQHVDIYSASWGPNDDGKTVEGPEKLARAAFEKGGRRGKGSIFAWASGNGGSNGDNCDCDGYTSSIYTVSISSASQQGRSPWYGEKCASTLATAYSSGE-YDQKISSTDLHHECTDSHTGTSAAAPLAAGVLALALEANPDLTWRDVQHLIVWTSEYDPLSGNPGWFQNGAGLWVNSRFGFGLLNAEAMVDMAQTWKTVPEKNTCEVRIENQPRNLGNGEEITIEMETDGCRGN-NHVEALEHVQVKTTIDYTRRGDLRIVLTSPSGTSTTLLDTRRQDKSQMGFQDWPFMSTHNWGEKPQGKWTLTIEDKSDHENNGVVKDVVLILHGTPEQP--

>Tad2

WVIHVPGGL-TNAKLVATTHASTILHQVGSLEDHYLIRHA-GTPHRSKRSSYKS-WNLRLDTRVLYVEQQISKLRIKRDPLWPKQWYLENTGQASGPPHLDHNIIPAWKSGATGIGIVVSILDDGLYYEHPDLRRNYDPEASFDINGNDHDPTPRKTGNDENRHGTRCAGEVAAIANNSICTVGAAYNAKIGGIRMLDG---D-VTDAVEATSLSWKPQHIDIYSSSWGPEDDGRNIDGPGPLAKKAFIDGGRHGLGSIFVWASGNGGSVHDNCNCDGYVISIYTISISAATDTGNVPWYTESCSSILATTFSSGNNGQNKIITDDLHDRCTESHTGTSAAAPLAAGMFALALEANPRLTWRDLQHIVVITSKPDKL-HTDDWTINGVGRKISNWFGYGLLDAAALVSTARNWKTVPIQRSKSFKFKTKKNYIPPNGRLIVSINVEKSEA----VNYLEHVQAVISLSHNQRGLITIQLISPAGTKSVLFDRRQQDTSRAGFKNWPLMTTHFWGEKSAGNWTLIIINKDSN--SGKLKGWKLVLFGTTSKP--

>Cel1

HTVIRLAKRDELARRIAADHDMHVKGDPF-LDTHYFLYHS--ETTRTRRHRAIV-ERLDSHPAVEWVEEQRPKKRVKRDPLYKDQWY-LHGGAVG---GYDMNVRQAWLQGYAGRNVSVSILDDGIQRDHPDLAANYDPLASTDINDHDDDPTPQ--NNGDNKHGTRCAGEVAALAGNNQCGVGVAFKAKIGGVRMLDG---A-VSDSVEAASLSLNQDHIDIYSASWGPEDDGKTFDGPGPLAREAFYRGGRGGKGNIFVWASGNGGSSQDSCSADGYTTSVYTLSISSATYDNHRPWYLEECPSSIATTYSSADFRQPAIVTVDVPGGCTDKHTGTSASAPLAAGIIALALEANPELTWRDMQHLVLRTANWKPLENNPGWSRNGVGRMVSNKFGYGLIDGGALVNMAKTWKTVPEQHICTYEYLANPNPRPIRFQLNFTLDVNGCESGTP-VLYLEHVQVHATVRYLKRGDLKLTLFSPSGTRSVLLPPRPQDFNANGFHKWPFLSVQQWGEDPRGTWLLMVESVTTNAATGTFHDWTLLLYGTADPA--

>Lgi2

FAVHIKGGR-EGLNRVLRSTGLINKGQIGSIDDHYFLEIP-NRQRRSATHPEHH-ALLSNHQDVHWFEQQVHKPRYKRDSLYGAQWYLHGGGRGGF----DMNVIPAWQKGYSGRNVVITILDDGIERNHPDLQQNYDPYASYDVNDHDNDPMPRYDPTDENRHGTRCAGEVSAVANNTVCGVGIAFNSKIGGVRMLDG---E-VYDAVEAASLSFNSTHIDIYSASWGPDDDGRVVDGPGPLAKKAFINGGRQGKGSIYVWASGNGGNAQDSCNCDGYTNSVYTLSISSTSEHGSKPWYLEECSSTLASTYSSGSYSEKQIVTVDLHGRCTETHTGTSASAPLAAGVVALVLEANPSLTWRDVQYITLMTANPKPM-VDGNWIVNGMGRKVSTRYGYGLMDASAMVDLAEVWTNVPEQHICEVFASANNIQLD-GSKFVGYIDTKACDGLPN-VNHLEHVQVKISLHHKKRGDVIIHIKSPMGTKSLMLPRRPNDRQEGEFKSWPFLSVHFWGENPTGIWTLEIEDAKTYSTGGVLKSWSIILHGTEKEP--

>Lgi3

-----------------------------------------------------------------------------------------------------MNVIPAWQKGYSGRNVVITILDDGIERNHPDLQQNYDPYASYDVNDHDNDPMPRYDPTDENRHGTRCAGEVSAVANNTVCGVGIAFNSKIGGVRMLDG---E-VYDAVEAASLSFNSTHIDIYSASWGPDDDGRVVDGPGPLAKKAFINGGRQGKGSIYVWASGNGGNAQDSCNCDGYTNSVYTLSISSTSEHGSKPWYLEECSSTLASTYSSGSYSEKQIVTVDLHGRCTETHTGTSASAPLAAGVVALVLEANPSLTWRDVQYITLMTANPKPM-VDGNWIVNGMGRKVSTRYGYGLMDASAMVDLAEVWTNVPEQHICEVFASANNIQLD-GSKFVGYIDTKACDGLPN-VNHLEHVQVKISLHHKKRGDVIIHIKSPMGTKSLMLPRRPNDRQEGEFKSWPFLSVHFWGENPTGIWTLEIEDAKTYSTGGVLKSWSIILHGTEKEP--

>Aqu

WVVELASDQGDAAEELAKTHGFTNMGQVGNLEGYYHFIDH-ESSTRTTRSDTKH-LNLMNDQQVVSAEQQVSQKRVKRDTMWSRQWY-LNRESK------NMNVMPVWDQGITGKGVVVTILDDGLEHTHPDLKANYDAEASYDFNANDHDPFPRYDVTNENRHGTRCAGEVAASLNN-VCSVGVAYHAKIGGVRMLDG---Q-VTDIVEAKSLSFGYEHIDIYSSSWGPNDDGKTVDGPAKLAKKAFLNGGRNGKGSIFVWASGNGGSYSDSCNCDGYVLSPFTIAIGSITEYNNFPWYAERCSSIMAITYSSGSSNEKKIISTDLHGLCTDSHTGTSAAAPMAAGMIALALEIRPDLTWRDVQYIIVYSSSSAI--DDDEWITNGAGLRVSSKYGYGLLDGAALVNRARHWVMVPERQNCTVHVLTEEQQSQSVIQIPIDITKESCPG----VLYLEHVQAITSLSVNKRGNIAISLRSPSGTLSKLLPTRHLDRHSTGFDQWPFMSVMNWGESPMGRWVYTVTTVANA--EFTMTDLTLVLYGVASKP--

>Hro6

WVIEVKGGE-ETAKEVAREHHLVYIDKV--MDDFYLVKHR-RLSKRSIKSYKFN-RELSTDSHITWFQQQVAKVRVKRDPKWPRMWY-MNQENGN-----GMNIMVAWKRGYTGRNVVVSILDDGIEKDHPDLKKNYDPEASYDVNDHDPDPQPRYDNTNENKHGTRCAGEVAAQFNNSLCGIGVAYNARIGGVRMLDG---D-VTDAVEAKSLSWKSDYVQIYSASWGPDDDGKTVDGPGPITAKAFEDGGRNGLGSIFVWASGNGGQLGDDCNCDGYTNLIYTLSISGVSENGNVPWYSEACSSALASAYSSGNSSERQIVTTDLNKQCTENHTGTSASAPLAVGMIALTMEANTQLTWRDVQHIVVLGAKRSNL-VDADFKLNGANKYVSHRFGFGLMDVGRMVELAENWTRVPAQRKCVVPRTISQKGLSVNSKLNISLTHNGCLDTED-INYLEHVQVIISLASQKRGELEIFLISPSGTKSTLLGKRPNDFSTDGFLNWTFMTTHCWGETAAGTWKLEIRNSASLVSPSRLLRWTLVLYGTKKEP--

>Aae3

---------------MNKKHQLSLSALIGPLKGYYLFHHS-HVRKRSLDREVHH-SALNTEPEVRWMQQQHEKIRRKRDPLFKEQWY-LNGGAKD---GLDMNIGPAWQKGYTGKGVVVSILDDGIQRNHPDLLQNYDPDASYDINGNDSDPMPR--DNGDNKHGTRCAGEVAAVAFNNYCGVGVAYNASIGGVRMLDG---T-VNDAVEAKALGLNPDHIHIYSASWGPEDDGSTVDGPGPLARRAFIFGGRQGKGSIFIWASGNGGRYTDSCNCDGYTNSIFTLSISSATQGGYKPWYLEECSSTLATTYSSGTGHDKSVATVDMDRICTVEHTGTSASAPLAAGIAALALEANPSLTWRDMQYLVVLTSRPEPLEKESGWILNGVKRKVSHKFGYGLMDAGSMVSLAEQWTSVPPQHICKSREINEDRPIEGGYTLQTHMDVNGCAGTVN-VRFLEHVQCKITLRFFPRGNLRILLTSPMGTTSTLLFERPRDVTKSNFDDWPFLSVHFWGEKAEGRWTLQILNGGRRSQSGILTKWQLIFYGTESNP--

>Gga3

WAVLVPAGP-LEANRLARKHGFLNLGPI--FGDYYHFQHR-GVVKRSLSPQPWH-SRLAREPQVHWLEQQVAKRRTKRDPKFPQQWYLYNTNQR------DLKVHQAWEQGYTGKGIVGSILDDGIEKNPPDLEANYDPGASFDVNDQDPDPQPRYTQMNDNRHGTRCAGEVAAVANNGICGVGVAYNARIGGVRMLDG---E-VTDAVEAHSLGLNPNHIHIYSASWGPEDDGKTVDGPARLAEEAFFRGGRGGLGSIFVWASGNGGREHDSCNCDGYTNSIYTLSISSTTQYGNVPWYSEACSSTLATTYSSGNQNEKQIVTTDLRQKCTESHTGTSASAPLAAGIIALALEANKNLTWRDMQHLVVQTSKPAHL-NANDWVTNGVGRKVSHSYGYGLLDAGAMVSLARNWITVGPQRKCVIDVLTEPKDI--GKRLEVRRKVDACLGKAN-ISRLEHAQARLTLSYNRRGDLAIHLVSPMGTRSTLLAARPHDYSADGFNDWAFMTTHSWDEDPSGEWLLEIENTSDANNYGTLTKFTLVLYGTATDP--

>Hsa

WAVRIPGGP-AVANSVARKHGFLNLGQI--FGDYYHFWHR-GVTKRSLSPRPRH-SRLQREPQVQWLEQQVAKRRTKRDPKFPQQWYLSGVTQR------DLNVKAAWAQGYTGHGIVVSILDDGIEKNHPDLAGNYDPGASFDVNDQDPDPQPRYTQMNDNRHGTRCAGEVAAVANNGVCGVGVAYNARIGGVRMLDG---E-VTDAVEARSLGLNPNHIHIYSASWGPEDDGKTVDGPARLAEEAFFRGGRGGLGSIFVWASGNGGREHDSCNCDGYTNSIYTLSISSATQFGNVPWYSEACSSTLATTYSSGNQNEKQIVTTDLRQKCTESHTGTSASAPLAAGIIALTLEANKNLTWRDMQHLVVQTSKPAHL-NANDWATNGVGRKVSHSYGYGLLDAGAMVALAQNWTTVAPQRKCIIDILTEPKDI--GKRLEVRKTVTACLGEPN-ITRLEHAQARLTLSYNRRGDLAIHLVSPMGTRSTLLAARPHDYSADGFNDWAFMTTHSWDEDPSGEWVLEIENTSEANNYGTLTKFTLVLYGTA--P--

>Aae4

WAVHIPEGGGETAEQVADEHGFINHGKI--FDGYYHFEHR-HLQKRSLNPGHHQ-RRLDGDDRDRWAKQQRAKRRPKRDPKWGEMWY-LNRGNGL-----DMNVIPACKEGVTGKGVVVTILDDGLESDHPDLEHNYDPKASYDVNGNDGDPMPHCDLTDSNRHGTRCAGEVAATANNSKCAVGIAYGARVGGVRMLDG---D-VTDVVEAKSLGLNSQHIDIYSASWGPDDDGKTVDGPGDMATRAFIEGGRGGKGSIFIWASGNGGREHDNCNCDGYTNSIWTLSISSASQEGLVPWFSEMCSSTLATTYSSGNTNEKQVITTDLHHSCTSSHTGTSASAPLAAGIAALVLEANPNLTWRDLQHIVVRTAKPGNL-KDPTWSKNGVGRRVSHSFGYGLMDAAAMVKLARTWKTVPEQQICEINAPHLDKQIPPRTKVTLQLVVEHCKG----VNYLEHVQAKITLTSQRRGDIQIFLTSPSGTRVTLLTPRSHDLSRSGFNQWPFMSVHTWGEAPHGTWQLEIHNEGRL--LAQITHWNLIFYGTETPA--

>HsaPCSK4

WAVQVSQGN-REVERLARKFGFVNLGPIFSDGQYFHLRHR-GVVQQSLTPWGHR-LHLKKNPKVQWFQQQTLQRRVKRDPWFSKQWY-MNSEAQP-----DLSILQAWSQGLSGQGIVVSVLDDGIEKDHPDLWANYDPLASYDFNDYDPDPQPRYTPSKENRHGTRCAGEVAAMANNGFCGVGVAFNARIGGVRMLDG---T-ITDVIEAQSLSLQPQHIHIYSASWGPEDDGRTVDGPGILTREAFRRGGRGGLGTLFIWASGNGGLHYDNCNCDGYTNSIHTLSVGSTTQQGRVPWYSEACASTLTTTYSSGVATDPQIVTTDLHHGCTDQHTGTSASAPLAAGMIALALEANPFLTWRDMQHLVVRASKPAHL-QAEDWRTNGVGRQVSHHYGYGLLDAGLLVDTARTWLPTQPQRKCAVRV--QSRPTPILPLIYIRENVSACAGLHN-IRSLEHVQAQLTLSYSRRGDLEISLTSPMGTRSTLVAIRPLDVSTEGYNNWVFMSTHFWDENPQGVWTLGLENKGYYFNTGTLYRYTLLLYGTAEDM--

>Hma1

WAIKLKGDDTERAKRIADKYGFDKVTKVGSLEGFYHLEHPHHPTRSKRSS-ESRTDTLLQDEEIFWAEQQHEYKRERRDPLWEEQWYLDDKRSQM---DLDVNVIPVWKSGISGKGVVVTILDDGIEHNHTDLERNYDPAASWDVNDNDPDPFPRYDPTNENKHGTRCAGEVAAQANNSKCGVGVAYNARIGGVRMLDG---R-VTDRVEAESLSLNPQYIDIYSASWGPSDDGMTVEGPGTLASAAFLNGGRGGLGSIYIWASGNGGRHDDSCNCDGYTASIYTLSISSSSDHGESPWYSEACASTLATTLSSGAHGEKRIVTTDLHNQCTERHTGTSASAPLA-G--------------------------------------------------------------------------------------------------------------------------------------------------------------------------------------------------------------

>Nve3

WAIHVNGGI-EEAKEVAKAHGFEKVEPIGTLEGYYHLEHP-SHPSRSRRSEDRT-DQLRQHRHVTWVEQQHEKVREKRDPLWDAQWY---LDDKRKDTDLDIHVIPVWKKGISGKGVVVTILDDGIEHNHTDLIKNYDPNASWDVNDGDNDPFPRYDPTNENKHGTRCAGEVAAEANNTKCGVGVAFNARIGGVRMLDG---R-VTDKVEAESLSLNPQYIDIYSASWGPSDDGATVEGPGTLATAAFLNGGRGGLGSIFVWASGNGGRHGDSCNCDGYTDSIYTLSISSASDHGESPWYSESCSSTLATTFSSGAHGEKRIVTTDLHNTCTERHTGTSASAPLAAGIFALALEVNPKLTWRDMQHIVVHTSNHLPLKHDQDWSKNGIDLMVNRKFGFGLLVAEKIVDMANTYVTVPEKRFCRGQINQDAKVFKWDKPLTIQLPATGCSGTGD-IRYLEHVQLMVSLDYVRRGDLVIYLTSPMGTKSCLLSPRKEDSSNEGFSKWPFMTTHSWGEDPRGTWTLEIKDLGDNANHGTLREWQLILHGTKDKP--

>Dre2

WAVHIEGGE-EEADRIARKHSFVNHGNV--FGDYYHFRHR-TVVKRSLSERGTH-IRLHTEPQVMWAEQQVVKRRKKRDPKFAQQWY-LYNQDHL-----DLNVKNAWKQGVTGQGVVVSILDDGIEKNHPDLVQNYDPDASYDVNDGDPDPQPRYTQLNDNRHGTRCAGEVAAVADNDICGVGVAYNARIGGVRMLDG---E-VTDVVEAQSLSLNPQHIDIYSASWGPEDDGKTVDGPAKLAKEAFQRGGRGGRGSIFVWASGNGGRMKDSCNCDGYTNSIYTLSISSSTQNGNVPWYSEACSSTLASTYSSGGVNEKQIVTTDLRQKCTDSHTGTSASAPLAAGIIALALEANKNLTWRDMQHLVVRTSNPAHL-TTNDWKINGVGRRVSHSYGYGLLDAGAIVALAKNWTNVGPQHKCVLSLVSEPRNI--GSYLVINKTVDACTGMAN-VSSLEHVQAQLTLSYNRRGNLAIYLISPQGTRSTLLPPRPHDYSSEGFNDWAFMTTHSWDEDPRGEWTLEIKNVAGTSDYGTLTQFTLVLYGTASSL--

>Xtr9

WAVHISGGS-AEADRLAKKYGFINHGLR--FEDYYHFAHR-AVMKRSLAPKSRQ-VLLKREPQVHWLEQQVAKKRKKRDPKFMQQWYLLDTNRH------DLHVKEAWEQGFTGKGIVVSILDDGIEKNHPDLQANYDPAASYDVNDQDPDPQPRYTQLNDNRHGTRCAGEVAAAANNGICGVGIAYNANIGGVRMLDG---E-VTDAVEARSLGLNPNHIHIYSASWGPEDDGKTVDGPAKLAEEAFYRGGRGGLGSIYVWASGNGGREHDSCNCDGYTNSIYTLSISSTTQMGNVPWYSEACSSTLATTYSSGNQNEKQIVTTDLRQKCTDSHTGTSASAPLAAGIIALALEANKNLTWRDMQHLVVQTSNPAGL-NANDWITNGVGRKVSHSYGYGLLDAGAMVALAKDWVTVGPQRKYVIDILSEPKDI--GKRLEVRRKVEPCAGMSN-ISTLEHVQARLSLSYNRRGDLAIYLTSPMGTRSCLLAPRPHDYSADGFNDWSFMTTHSWDEDPAGEWVLEIENVSNNNNYGTLTQFVLVLYGTAS----

>Dre3

WAVHIEGGV-EEAERIAQKHGFVSHGQV--FGDFYHFQHR-GVVRRSLSSRGVH-VRLHTEPQVLWAEQQIAKRRRKRDPKFPQQWYLYNPSHG------DLNVKEAWAQGFTGRGVVVTILDDGIEKDHPDLAKNYDPDASYDVNDRDPDPQPRYTQLNDNRHGTRCAGEVAAAANNGVCGVGVAYNAKIGGVRMLDG---E-VTDVVEAQSLSLNSQHIHIYSASWGPEDDGKTVDGPAKLAKEAFLQGGRSGLGSIFVWASGNGGRERDSCNCDGYTNSIYTLSISSTTQYGSVPWYSEACSSTLATTYSSGNLNEKQIVTTDLRKKCTDSHTGTSASAPLAAGIIALALEANMNLTWRDMQHLVVRTSRPAHL-ITNDWRTNGVGRLVSHSYGYGLLDATAMVALAQNWTSMGPQHKCVINMLTEPRDIKNH--LTFSRSVEACSGQPD-VSSLEHVQARLTLSYNHRGNLAVHLISPLGTRSTLLAPRPQDNSAEGFNDWAFMTTHSWDEDPRGEWTLEIENVAGLNDYGVLSQFTLILYGTGSSA--

>Spu

YAVHIEGGP-AEAAIVAKAHNFVYLGEI--LQDHYHLLDEHHTVKRSTSHQVRH-GHLSEEPKVLWFEQQIARSRQKRDPKWP-IWY-LARGPGI-----DMNILPAWEAGYTGKGVVVSILDDGIERDHPDLMKNYRKNASYDVNGKDDDPEPRYNFSNENRHGTRCAGEVAAQANNSICSVGVAYNAGIGGVRMLDG---D-VTDAVEAQSLSLNPQIIDIYSASWGPDDDGQTVDGPGKLAKIAFLNGGRDGLGSIFVWASGNGGRSDDSCGCDGYTNSIFTISVSSASENGGVPWYSENCASTLATTYSSGSGTEKQVVTTDLRKKCTDSHSGTSASAPLAAGICALALEANPQLNWRDLQHIIVMTSRPDNI-HTSDWTVNGVGRSVSHDYGYGLMDAGAMVMLAKNWTHVPEQRLCTINSNGNSQKINGHSGLVVHAQTTGCQETPDTVRFLEHAVSRISLDFPIRGDLSIALISPSGTRSSLLPRRPHDRNKKGFKSWEFMTTHTWGENPQGEWTLEIQNHGAAGMSGVLHDWTLLLYGTQPHP--

>Oca

-------------------------------------------------------------------------------------WYLLNTGQASGPSGLDHNVVPVWKEGLTGKGVVVSILDDGIEYTHPDLKDNYDPKASYDYNGNDADPFPRYDVTNENRHGTRCAGEVAA-AMNEVCGVGVAFNAKIGGVRMLDG---D-VTDVIESRSLSLNPQHVDIYSSSWGPDDDGRTVDGPAALAQKAFKQGGRGGLGSIFVWASGNGGRAGDCCNCDGYTNSIYTLSISSATDQGGKP-YLERCSSTLATTYSSGS-----------------------------------------------------------------------------------------------------------------------------------------------------------SGQKQIT---------------------------------------------------------------------------

>Gga2

----------------------LSFFQIGSLENYYHFHHS-KTFKRSTLSRGPH-SFLRMDPKVEWLQQQEVRRRVKRDPVWPNMWY-LHCGDRNSRCRSEMNVVAAWQRGYTGRNVVVTILDDGIERNHPDLLQNYDPLASYDVNGNDHDPTPRYDASNENKHGTRCAGEVAAAANNSYCIVGIAYNARIGGIRMLDG---D-VTDVVEAKSLGIRPDYIDIYSASWGPDDDGKTVDGPGLLAKQAFEHGGRRGLGSIFVWASGNGGREGDYCSCDGYTNSIYTISISSTTENGYKPWYLEECASTLATTYSSGAFYERKIVTTDLRHRCTDGHTGTSVSAPMVAGIIALALEANPLLTWRDVQHLLVRTSRPVHL-RAADWKTNGAGHKVSHLYGFGLVDAEAIVVEAKKWKTVPPQHVCVGSLDRVPKYIRPDHVLRASTLSSACSEQREQVLYLEHVVVRLSIAHPRRGDLQISLVSPAGTRSQLLARRVFDHSNEGFKGWEFMTVHCWGERAAGEWTLEIQDTPSQTAQGKLKEWSILFYGTAEHP--

>Hsa1

WAVQVLGGP-AEADRVAAAHGYLNLGQIGNLEDYYHFYHS-KTFKRSTLSRGPH-TFLRMDPQVKWLQQQEVKRRVKRDPIWSNMWY-LHCGDKNSRCRSEMNVQAAWKRGYTGKNVVVTILDDGIERNHPDLAPNYDSYASYDVNGNDYDPSPRYDASNENKHGTRCAGEVAASANNSYCIVGIAYNAKIGGIRMLDG---D-VTDVVEAKSLGIRPNYIDIYSASWGPDDDGKTVDGPGRLAKQAFEYGGRQGLGSIFVWASGNGGREGDYCSCDGYTNSIYTISVSSATENGYKPWYLEECASTLATTYSSGAFYERKIVTTDLRQRCTDGHTGTSVSAPMVAGIIALALEANSQLTWRDVQHLLVKTSRPAHL-KASDWKVNGAGHKVSHFYGFGLVDAEALVVEAKKWTAVPSQHMCVAASDKRPRSIPLVQVLRTTALTSACAEHSDQVVYLEHVVVRTSISHPRRGDLQIYLVSPSGTKSQLLAKRLLDLSNEGFTNWEFMTVHCWGEKAEGQWTLEIQDLPSQEKQGKLKEWSLILYGTAEHP--

>Dre5

WAVKITGGP-EAADLIAEKYGFVNMGQIGGLVDHYQFQHS-GIIKRSTIKKGNH-SLITMETKVEWIQQQMVQKRVKRDPKWDSMWY-IHCDHN---CLTDMNIQAAWRRGYTGKGVVVSILDDGIERQHPDLKQNYDARASYDVNGNDPDPTPRYDVTNENKHGTRCAGVVAASANNSLCIVGIAYNAKIGGVRMLDG---D-MTDMVEAQSLNLRQQYIDIYSSSWGPDDDGRTVDGPGPLARLALENGGRKGRGSIFVWASGNGGQSQDHCSCDGYTNSIYTISVGSTTQSGRKPWYLEECSSTLATTYSSGDSHSPGVVTTDLRQRCTDEHSGTSASAPMAAGIIALTLEANPALTWRDIQHIIVKTSSRGHL-SASDWQSNGAGYDVSHLYGFGLLNAEAMVKNAETWKQVPSQHICEENIGQNARIISPERVLRSVLKSSGCSAQRLQVVYLEHVIVRVTITHPHRGDLSITLTSPSGTTSQLLANRPNDHSSEGFIKWEFMTTHCWGERLAGDWILDIRDTPSPRLQGKLVEWSLVLYGTSTHP--

>Nve9

------------------------------------------------------------------------------DPLFEEQWHLAGYE------AFDHHVLPVWQMGVYGKDVVVSILDDGIEYTHDDLKENYDKLASYDFNSNDADPAPRYTWNDENRHGTRCAGEVAAQMNNSVCGVGVAPKAKVGGVRMLDG---D-VTDAVEAGSLSLNPQHIDIYSASWGPDDDGKTVDGPARLARKAFTDGGRGGKGSIYVWASGNGGRTSDNCNCDGYTNSIYTLSISSVTEIGNSPWYSEACSSTLASAFSSGSWNNRKIVTVDVRNRCTKTHTGTSASAPLAAGILAMALEVNRNLTWRDMQHIVVRTCTMDKLNM-HDVVTNGVGRLVSHTFGYGLLDATRLVKLARVWRTVPPQRVSFLFI---YRKIPDDGALTVRTEATGCTGTANEIRYLEHVECIISLDSVKRGDISIYLTSPRGTRSTLLGKRIRDNSQNGFHDWAFMTTHSWEENPTGTWTLEIFNEKTP----------------------

>Hsa7

----------------------------------------------------------------------------------------------------------------------------------------------------------------------------------------------------------------------------------------------------------------------------------------NSIYTISISSTAESGKKPWYLEECSSTLATTYSSGESYDKKIITTDLRQRCTDNHTGTSASAPMAAGIIALALEANPFLTWRDVQHVIVRTSRAGHL-NANDWKTNAAGFKVSHLYGFGLMDAEAMVMEAEKWTTVPRQHVCVESTDRQIKTIRPNSAVRSIYKASGCSDNPNRVNYLEHVVVRITITHPRRGDLAIYLTSPSGTRSQLLANRLFDHSMEGFKNWEFMTIHCWGERAAGDWVLEVYDTPSQKTPGKLKEWSLVLYGTSVQP--

>Hsa8

-----------------------------------------------------------------------------------------------------------------------------------------------------------------------------------------------------------------------------------------------------------GRRGLGSVFVWASGNGGRSKDHCSCDGYTNSIYTISISSTAESGKKPWYLEECSSTLATTYSSGESYDKKIITTDLRQRCTDNHTGTSASAPMAAGIIALALEANPFLTWRDVQHVIVRTSRAGHL-NANDWKTNAAGFKVSHLYGFGLMDAEAMVMEAEKWTTVPRQHVCVESTDRQIKTIRPNSAVRSIYKASGCSDNPNRVNYLEHVVVRITITHPRRGDLAIYLTSPSGTRSQLLANRLFDHSMEGFKNWEFMTIHCWGERAAGDWVLEVYDTPSQKTPGKLKEWSLVLYGTSVQP--

>Hsa4

WAVKIAGGF-PEANRIASKYGFINIGQIGALKDYYHFYHS-RTIKRSVISRGTH-SFISMEPKVEWIQQQVVKKRTKRDPKWPSMWY-MHCSDNTHPCQSDMNIEGAWKRGYTGKNIVVTILDDGIERTHPDLMQNYDALASCDVNGNDLDPMPRYDASNENKHGTRCAGEVAAAANNSHCTVGIAFNAKIGGVRMLDG---D-VTDMVEAKSVSFNPQHVHIYSASWGPDDDGKTVDGPAPLTRQAFENGGRRGLGSVFVWASGNGGRSKDHCSCDGYTNSIYTISISSTAESGKKPWYLEECSSTLATTYSSGESYDKKIITTDLRQRCTDNHTGTSASAPMAAGIIALALEANPFLTWRDVQHVIVRTSRAGHL-NANDWKTNAAGFKVSHLYGFGLMDAEAMVMEAEKWTTVPRQHVCVESTDRQIKTIRPNSAVRSIYKASGCSDNPNRVNYLEHVVVRITITHPRRGDLAIYLTSPSGTRSQLLANRLFDHSMEGFKNWEFMTIHCWGERAAGDWVLEVYDTPSQKTPGKLKEWSLVLYGTSVQP--

>Xtr5

--------------------------QIGNLENYYHFYHS-RTIKRSTYARGTH-SFLRMHPKVEWMQQQEVKKRVKRDPIWPNMWY-LHCSDKSSRCRSEMNVMSAWQRGYTGKNVVVSILDDGVEKNHPDLIQNYDPHASHDVNGDDQDPSPRYDASNENKHGTRCAGEVAASANNSHCIVGIAYNARIGGIRMLDG---D-VTDVVEAKSLGVRPDYIDIYSSSWGPDDDGKTVDGPGPLARKAFEDGGRKGLGSIFVWASGNGGREGDYCSCDGYTNSIYTISISSTTENGYKPWYLEECASTLATTYSSGAFYERKIVTTDLRQGCTDDHTGTSVSAPMVAGVIALALEANPVLNWRDVQHLLVKTSRSVHL-RAPDWKTNGAGRKVSHLYGFGLVDADAMVVEAKKWRTVPPQHVCIGASDRRPRFIRAVQLIRTTTQTNACVDNAGHVAYLEHVVVRVTISHPRRGDLQIYLISPSGTKSQLLAKRTFDSSNEGFKNWEFMTVHCWGEKAEGEWTLEIHDSSSQEIQGKLKEWTLIFYGTSEHP--

>Lgi5

-----------------MRTLFIYSQQNMTCSINYLRV-------------------------IKWLEQQVAKKRIKRDPKWPIMWYLNRGS------GLDMNVSHAWRMGYTGRGVVVTILDDGIEKDHPDLAKNYDHDASYDVNGHDHDPQPRYDYSNENRHGTRCAGEVAAQADNGVCNVGVAFNARIGGVRMLDG---D-VTDSVEAQSLSLNPQHIDIYSASWGPDDDGRTVDGPATLARKAFYDGGRDGKGSIFVWASGNGGRDGDNCNCDGYTNSIYTLSISSVTENGNIPWYSEACSSTLATTYSSGSGGEKQIVTTDLRRGCTENHTGTSASAPLAAGLIALGLEANPRLTWRDMQHIVVETAKPYRLKA-SDWVTNGVGKNVSHSFGFGLMDAGSIAALARNWTTVPAQHICEIRSPDHNRIIPTNGKITVPLYTNSCQGSASHVKYLEHVQARITMTSSRRGEIQIFLTSPAGTKSTLLAKRVRDTSREGFNNWAFMTTHNWGEFAEGTWFLEIENGASS----------------------

>Lgi1

WAVHVEGGE-RVARSLADKYGFVYLDQI--MPDYYHFQHR-KVAKRSIQPSNYHHKTLASDPNIKWLEQQVAKKRIKRDPKWPIMWYLNRGS------GLDMNVSHAWRMGYTGRGVVVTILDDGIEKDHPDLAKNYDHDASYDVNGHDHDPQPRYDYSNENRHGTRCAGEVAAQADNGVCNVGVAFNARIGGVRMLDG---D-VTDSVEAQSLSLNPQHIDIYSASWGPDDDGRTVDGPATLARKAFYDGGRDGKGSIFVWASGNGGRDGDNCNCDGYTNSIYTLSISSVTENGNIPWYSEACSSTLATTYSSGSGGEKQIVTTDLRRGCTENHTGTSASAPLAAGLIALGLEANPRLTWRDMQHIVVETAKPYRLKA-SDWVTNGVGKNVSHSFGFGLMDAGSIAALARNWTTVPAQHICEIRSPDHNRIIPTNGKITVPLYTNSCQGSASHVKYLEHVQARITMTSSRRGEIQIFLTSPAGTKSTLLAKRVRDTSREGFNNWAFMTTHNWGEFAEGTWFLEIENGASSFRPVRLRDWVLVLYGTERHP--

>Lgi4

---------------------------------------------------------------------------------------------------------------------------------------------------------------------------------------------------MLDG---D-VTDSVEAQSLSLNPQHIDIYSASWGPDDDGRTVDGPATLARKAFYDGGRDGKGSIFVWASGNGGRDGDNCNCDGYTNSIYTLSISSVTENGNIPWYSEACSSTLATTYSSGSGGEKQIVTTDLRRGCTENHTGTSASAPLAAGLIALGLEANPRLTWRDMQHIVVETAKPYRL-KASDWVTNGVGKNVSHSFGFGLMDAGSIAALARNWTTVPAQHICEIRSPDHNRIIPTNGKITVPLYTNSCQGSASHVKYLEHVQARITMTSSRRGEIQIFLTSPAGTKSTLLAKRVRDTSREGFNNWAFMTTHNWGEFAEGTWFLEIENGASS---SELKEWTLVVMGTGTHP--

>Lgi8

---------------------------------------------------------------------------------------------------------------------------------------------------------------------------------------------------------------------------------------DDGRTVDGPATLARKAFYDGGRDGKGSIFVWASGNGGRDGDNCNCDGYTNSIYTLSISSVTENGNIPWYSEACSSTLATTYSSGSGGEKQIVTTDLRRGCTENHTGTSASAPLAAGLIALGLEANPRLTWRDMQHIVVETAKPYRL-KASDWVTNGVGKNVSHSFGFGLMDAGSIAALARNWTTVPAQHICEIRSPDHNRIIPTNGKITVPLYTNSCQGSASHVKYLEHVQARITMTSSRRGEIQIFLTSPAGTKSTLLAKRVRDTSREGFNNWAFMTTHNWGEFAEGTWFLEIENGASS---SELKEWTLVVMGTGTHP--

>Dre7

WAVRIAGGP-EQAEHIANKYGYRNLGQIGDLKDYYHFFHS-RTIKRSTLFRGMH-SFISMEPKVEWVQQQVVKRRIKRDAKWSSMWY-IHCNDNMHNCQSDMNIVGAWKRGYTGKDVVVTILDDGIERNHPDLIQNYDNEASYDVNGNDVDPMPRYDASNENKHGTRCAGEVAASANNSHCTVGIAYNAKIGGVRMLDG---D-VTDMVEAKSLSLHPQHIDIYSASWGPDDDGKTVDGPASLARQAFENGGRKGRGSIFVWASGNGGRSRDHCSCDGYTNSIYTISISSTAESGRKPWYLEECSSTLTTTYSSGENYDRKIITTDLRQRCTDSHTGTSASAPMAAGIIALALEANPFLTWRDVQHIVVKTSRAGHL-SAPDWKTNAAGYNVSHLYGFGLMDAEAMVKEAEHWKQVPLQHICVENADKQIRTIRPEHVVRSVYKATGCTDNANHVIYLEHVVVRITITHPRRGDLSINLTSPSGTKSQLLANRLFDHSMEGFKNWEFMTTHCWGEKAAGDWILEIYDSPSQKAPGKLKEWSLVLYGTSTHP--

>Dre8

WAVRIAGGP-EQAEHIANKYGYRNLGQIGDLKDYYHFFHSRTIKRSTLFS-RGMHSFISMEPKVEWVQQQVVKRRIKRDAKWSSMWY-IHCNDNMHNCQSDMNIVGAWKRGYTGKDVVVTILDDGIERNHPDLIQNYDNEASYDVNGNDVDPMPRYDASNENKHGTRCAGEVAASANNSHCTVGIAYNAKIGGVRMLDG---D-VTDMVEAKSLSLHPQHIDIYSASWGPDDDGKTVDGPASLARQAFENGGRKGRGSIFVWASGNGGRSRDHCSCDGYTNSIYTISISSTAESGRKPWYLEECSSTLTTTYSSGENYDRKI--------------------------------------------------------------------------------------------------------------------------------------VSYQEHCKTIVL------ASLALS-------------------------------------------------------------------------

>Xtr6

-----------------------------------------------------------------------------------------------------------------------------------------------------------------------------------------------------------------------------------------------------------GRRGFGSVYVWASGNGGRSRDHCSCDGYTNSIYTISISSTTESGKKPWYLEECASTLATTYSSGESYDRKVITTDLRQRCTDSHTGTSASAPMAAGIIALALEANPFLTWRDVQHIIVRTSRQRHL-NAPDWKTNAAGYKVSHLYGFGLMDAEAMVIEAEKWTTVPAQHICVENTDRQIKTIRPDNVVRSVYKATGCADNTNHVVYLEHVVVRVSITHPRRGDLAIYLTSPSGTRSQLLANRLFDHSMEGFKNWEFMTTHCWGERASGDWTLEIYDTPSQKTPGKLKEWSLVLYGTSVHP--

>Xtr3

WAVRIAGGE-TEANRIASKYGYTNMGQIGALQDYYHFFHS-KTIKRSILSRGTH-SFISMEPKVEWIQQQVVKKRIKRDPKWPSMWY-MHCSENVHHCQSDMNIVGAWKRGYTGKNVVVTILDDGIERNHPDLMQNYDAQASTDINGNDFDPMPRYDASNENKHGTRCAGEVAATANNSHCTVGIAFNAKIGGVRMLDG---D-VTDMVEAKSLSLNPQHVHIYSASWGPDDDGKTVDGPASLAREAFENGGRRGFGSVYVWASGNGGRSRDHCSCDGYTNSIYTISISSTTESGKKPWYLEECASTLATTYSSGESYDRKVITTDLRQRCTDSHTGTSASAPMAAGIIALALEANPFLTWRDVQHIIVRTSRQRHL-NAPDWKTNAAGYKVSHLYGFGLMDAEAMVIEAEKWTTVPAQHICVENTDRQIKTIRPDNVVRSVYKATGCADNTNHVVYLEHVVVRVSITHPRRGDLAIYLTSPSGTRSQLLANRLFDHSMEGFKNWEFMTTHCWGERASGDWTLEIYDTPSQKTPGKLKEWSLVLYGTSVHP--

>Gga5

--------------------------QIGTLKDYYHFYHS-KTIKRSVLSRGTH-SFISMEPKVEWIQQQVVKRRIKRDPKWPSMWY-MHCSDNTHHCQSDMNIVGAWKRGYTGKNVVVTILDDGIERNHPDLMQNYDSQASFDVNGNDFDPMPRYDASNENKHGTRCAGEVAATANNSHCTVGIAFNAKIGGVRMLDG---D-VTDMVEAKSLSLNPQHIHIYSASWGPDDDGKTVDGPASLARQAFENGGRRGLGSVFVWASGNGGRSRDHCSCDGYTNSIYTISISSTAESGKKPWYLEECASTLATTYSSGESYDRKIITTDLRQRCTDSHTGTSASAPMAAGIIALALEANPFLTWRDIQHIIVRTSRAGHL-NANDWKTNAAGYKVSHLYGFGLMDAEAMVIEAEKWTTVPPQHVCVENTDRQIKTIRPDSVVRSIYKATGCSDNPNHVIYLEHVVVRITITHPRRGDLAIYLTSPSGTRSQLLANRLFDHSMEGFKNWEFMTTHCWSEKAAGDWILEICDTPSQKTPGKLKEWSLVLYGTSIQP--

>Gga8

-----------------------------------------------------------------------------------------------------------------------------------DLMQNY------DAN--------------ENKHGTRCAGEVAATANNSHCTVGIAFNAKIGGVRMLDG---D-VTDMVEAKSLSLNPQHIHIYSASWGPDDDGKTVDGPAPLTRQAF-----------------------------------------------------------------------------------------------------------------------------------------------------------------------------------------------------------------------------------------------------------------------------------------------------------------

**MASTERMIND**

>Nve

PKHRVVVDKLRVRLTGYREHHNTCQSKQDRLQALREDQVRQ-DAAMLHQRAIDRSAVLKRKYDQGLSNTVNILNNDKENGQGSESLSKYQRTDEFSNTGLQSHELQGMNLGSQHDQHSTAASSQQLDSIETLSNPNLSTGGHFVN---DGSRSSPLLTNNTEAYNVRLQSQSPNTLQQAPSEFKQEPRQIVVSHEAM-ANGVSISNTSVRTSENNNTNDGFAGQGLQQELK-DFEDVLRKYQSQSYKGSPINNNEIS------------PQQHRQL-----QRPSQPGQMDNVTRETRSAQQSPSH--LQQLARQAQSQRARGFIENPNMSQYSAQNMQYYDMRFASSGSQAQVPNTGTM-PQVTPAGR--LRPPMPPSTSDQYGNM--YSSKDQNQSPSNASL-----QYQRMMHSQASQQALYNRMNAQQNMSQRLSHPSIPEQQAYNPYGNQ----------VMSQAPPQVNNMSMQNM-PAYSS-------------QRQLSMPVMSQGYPNQEQYAQQMQQYQVNEQQGPMPQQQQPQGDMNCGSRAMSQPAGAYGQA------MRGMPGQAPSMIEGQGHPDHGMAYG-SAMRDLNSQS-VTQDN-GMPEAGSVPVSRNN-QFNAFSPLAALEKAPSGNLTN-------LETTYELLGDIIGQGV-------------

>Bfl

PKRRAIVDRLRARIELYRKHHNECQARDVQTQAARLQQERQ-DTLVLHQRVCDSRTKLLLTLKRKLGGSGSQASDSAADGVEENHTTKKTKTSEATNQSHNLHGFPVGNPVE-NVGTIKQSHTPDAVMKSEAGLEDTRPQDHVAE---AAAANSHSPATNLPV------------------ECKQEPQLEDNSKAALSSSDINMSDKDFMNILDELENPEWPEEDLRSQIN-EFDKILDSIREKGDAGLLDGAVNGEQQGSPFHPDQKQPIDHILPTSDASFSKAVQDSIQNQCSPMNYGQNDPNSLAAEQLKQMAAQKQSGTEHWNTMQANPHPGIPSQAQPSRTTTCSGYMDGAGPMGSPHGFHPEQQPIM----HLEQNMPGGP-----LNSHPA-SEANR-----AITSLTMANTKPLSHYS---------GPEMKPQLPPISQMHAFGQQ----------VRPPPPTMKQNVT-----QYHPQLPTHSQAAVDKTSSIAARNAVQAQLVQRQQQQQQQQQQQQHPGMPEHLMEQHQHYLNRPPPDNFQAMKAGVPAMG------RQAEVKQEIGWSQPQAHSQSSMQGF--GEMPLDGRQ-----NP--PTTQPLPPHMAGATISTAQRVYTTSACPATIIYGAQVQPNSMACNWDYINRYALRNIS------------

>Dre

PRHSAVMERLRRRIELFRRHHTGCENRYDNTAMERLEMERQ-QTIALQQRFLQTKAKLQETVKRKLESAESPLGRDQVNGFTDG--YP---PNKKSCVEDAMGGLNG---VS-NGVVPPLSPLDTKHNVNT--DAMMANGNHRVV---GSEHNGASLKDNGAVRGS---ESDFRL-----KEPKQEPVDDALSNNSL-FPDLNLNDQDWSEIMEEFNRS-VPYEDIQELFSVSFGDRKDPELTSAAAQSLISQDLPNVKTEISPATATSAFEQDSCNGSPQMRPTSSGPPLHTNSPVTAPATSPAS-PAQQLQQLA---QQRAILHSQHSTSWPQNAPTQSQMGGTFGLE---KPTSPSLYPQDFPNPKTLLMP---NKGSPKAGAPAGYMQPGGHANPSNPGA-----QAAMLDFNNTKPLSHYDDGPPGAPRGPPTTQNHNKPNQAILNMMRQ----------IQKQRAP---TMNFRPAHLPHTALKQQQIQLMNQQKQFQLQRQIMVEQEKQRQQQEQQLQRHLTRPPPQYQDQQNVSQFTSVPPCRMFSQTQGMMGIGY----GQNVLAAQQQAHLKNQPGSANGAMPAQSRLTKLPNNTPFPQGNPGMPQQLPPPVQQTAPDLGPFSQA---QAVPQAYQLN-RAANQQLQFSYDLVDSLLKDQSTQQWMDDIDELLA

>Xtr1

PKHSTVVERLRQRIEGCRRHHVSCESRYQQAQAEQLDMERR-DTVNLYQRSLEQRAKLQEAVHRKLEGARSPQNGEQQNGVCDGRFSP---SAKRRRKELP-GMDTLNN-LS-NMPLPSVSPLHQLDIKPH----IQNSGIHSGG-LEDVSKNGGQQDIKLAVNGSSDLEESLNILQN--KEIKHEPLDDASVQNKL-FSDINLNDQEWQELIDELANT-VPEDDIQDLFNEDFEEKKELDFPRPSAQTPLPHESASVKSDPSHS----PFPSVSI-GSPQVRPSSSGPSF---SNVSSVSSIPSS-PAEQLKQMAAQQQQRAKLIQQQPSSWSPVGPPSSPYGGAYGTE---KPTSPMMYPQVFNNQT-PIVSPMANNPPKTTMNN--YLPQS-HMNTNNLGTNSISKPSNMLSYGNTKPLSHFS-AEMSQRMTPPLANQNKNSLIPYIQQQPPHLSEEQKRLLIMKQKGLMNQSMGYPPL-SAHGQMPNQPQAAMMK--QMFIEQRAQLMEQQKQQLLREQRQQQILAEQTNFVLPQQQPHLNRQHLQRIIVQNAGMMPMGYNMNTGRQPSSGQPLGLVGGFGGD---IGAYQSGQPRMPKQH-FQQGNPGRQMIQSLPGQQLNPSMPSFNPSPS-QQMPQGQVYD-RNPSQDIAYSYDLVDSIMNRGPGDEWMQELDELFG

>Gga1

---------------------------------------------------------LQETVKRKLEGARSPLNGEQQNGVCDGSFSP---TSKRIRKDVP-GIEAINSLPN-NLPLPAVSPLHQLDMKAS--LPLQNSGTHGSG-LEDLGKNGGLSEIKLPVNGCNELDDGFNVLQN--KELKQEPLDDPTCQNKL-FSDINLNDQEWQELIDELANT-VPEDDIQDLFNEDFEEKKEPDLTRPATET---QESASVKSDPSHS----PFAHVPL-GSPQVRPSSSGPPF---SNVSTASSVTSS-PAEQLKQMAAQQQQRAKLMQQQASSWSPVGPPSSPYGGPFSAE---KPNSPMMYPQAFNNQN-PIVPPMANNPQKTTMNN--YLPQN-HMNPNNLGTNSLNKQPNMLSYGNTKPLTHFN-AELNQRMTPPMANPSKNPMMPYIQQQQSHLSEEQKRMLIMKQKSMLNQPMAYATL-PSLAQLGNQPQAAIMK--QMLIEQRAQLHVIEQQKQQFLREQRQ---QQQQILAEQQLQQQSHLPRQRMMAQNASMMAMGYSMSTGRQTASGQAVGMVSGFGGE---IGPFQSGQPRLSKQH-FPQGNPGRQLLQPLPGQQGTPTMSGFSQPPT-QQMPQGQAYE-RNPTQDIPYSYDLVDSIIKSGPGDEWIQELDELFG

>Hsa1

---------------------------------------------------------------------------------------------------------------------------------------------------------------------------------------------------------------------------------------------------------------------------------------------------------------------------------------------------------------------------------------------------------------------------------------------------------------------------------------------------------------------------------------------------------------------------------------------------------GE---LGPFQAGQPRLTKQH-FPQGNPGRQMMPSLPGQQGTPGMPAFSQPPAQQQIPQSQAYE-RNAPQDVSYNYDLVDSIIKGGPGDEWMQELDELFG

>Hsa2

---------------------------------------------------------LQETVKRKLEGARSPLNGDQQNGACDGNFSP---TSKRIRKDISAGMEAINNLPS-NMPLPSASPLHQLDLKPS--LPLQNSGTHTPGLLEDLSKNGRLPEIKLPVNGCSDLEDSFTILQS--KDLKQEPLDDPTCQNKL-FSDINLNDQEWQELIDELANT-VPEDDIQDLFNEDFEEKKEPEFSQPATETPLSQESASVKSDPSHS----PFAHVSM-GSPQARPSSSGPPF---STVSTATSLPSS-PAEQLKQMAAQQQQRAKLMQQQTSNWSPLGPPSSPYGAAFTAE---KPNSPMMYPQAFNNQN-PIVPPMANNLQKTTMNN--YLPQN-HMNPNNLGTNSLNKQHNILTYGNTKPLTHFN-ADLSQRMTPPVANPNKNPLMPYIQQQQQHLSEDQKRLLLMKQKGVMNQPMAYAAL-PSHGQ----------------------------------------------------------------------------------------------------------------------------------------------------------------------------------------------------

>Hsa3

PKHSTVVERLRQRIEGCRRHHVNCENRYQQAQVEQLELERR-DTVSLYQRTLEQRAKLQETVKRKLEGARSPLNGDQQNGACDGNFSP---TSKRIRKDISAGMEAINNLPS-NMPLPSASPLHQLDLKPS--LPLQNSGTHTPGLLEDLSKNGRLPEIKLPVNGCSDLEDSFTILQS--KDLKQEPLDDPTCQNKL-FSDINLNDQEWQELIDELANT-VPEDDIQDLFNEDFEEKKEPEFSQPATETPLSQESASVKSDPSHS----PFAHVSM-GSPQARPSSSGPPF---STVSTATSLPSS-PAEQLKQMAAQQQQRAKLMQQQTSNWSPLGPPSSPYGAAFTAE---KPNSPMMYPQAFNNQN-PIVPPMANNLQKTTMNN--YLPQN-HMNPNNLGTNSLNKQHNILTYGNTKPLTHFN-ADLSQRMTPPVANPNKNPLMPYIQQQQQHLSEDQKRLLLMKQKGVMNQPMAYAAL-PSHGQLGSQPQAAIMK--QMLIDQRAQLIEQQKQQFLREQRQQQ---QQQQQILAEQQLQQSHLPRQRLMAQNAGMMGIGYNMSTGRQPASGQGVGMVSGFGGE---LGPFQAGQPRLTKQH-FPQGNPGRQMMPSLPGQQGTPGMPAFSQHPAQQQIPQSQAYE-RNAPQDVSYNYDLVDSIIKGGPGDEWMQELDELFG

>Hsa4

RPRLEVGSKRRLGVGPSGAGGKPAAAREPAPLPSGAWRGREAERGSRGERSPQCQPALHDTVKRNLDSATSPQNGDQQNGYGD--LFP---GHKKTRREAPLGVAIS------SNGLPPASPLGQSD-KPSGADALQSSGKHSLGL--DSLNKKRLADSSLHLNGGSNPSESFPLSLN--KELKQEPVEDLPCQSNL-MPDLNLNEQEWKELIEELNRS-VPDEDMKDLFNEDFEEKKDPESSGSATQTPLAQD-INIKTEFSPA----AFEQEQL-GSPQVRAGSAGQTFLGPSSAPVSTDSPSS-SAHQLQQIAAKQKREQMLQNPQMSTWQQTGPSHSSLDVPYPME---KPASPSSYKQDFTNSKLLMMPSV-NKSSPRPGGP--YLQPS-HVNPSNLNQNSANNQGSVLDYGNTKPLSHYK-ADCGQ--GSPGSGQSKPALMAYLPQQL-HISHEQNSLFLMKPKPG---NMPFRSL-VPPGQLSSQQQAAVMKQHQLLLDQQKQREQQQKHLQQQQFLQRQ------QHLLAEQQRHLTRPPPQ----------------------------------------VGQF------------------AQGLSCALPMIHST------------SSLCQ-----------------------------RSW---------

>Hsa5

PRHSAVMERLRRRIELCRRHHSTCEARYEAVSPERLELERQ-HTFALHQRCIQAKAKLHDTVKRNLDSATSPQNGDQQNGYGD--LFP---GHKKTRREAPLGVAI----SS-N-GLPPASPLGQSD-KPSGADALQSSGKHSLGL--DSLNKKRLADSSLHLNGGSNPSESFPLSLN--KELKQEPVEDLPCQSNL-MPDLNLNEQEWKELIEELNRS-VPDEDMKDLFNEDFEEKKDPESSGSATQTPLAQD-INIKTEFSPA----AFEQEQL-GSPQVRAGSAGQTFLGPSSAPVSTDSPSS-SAHQLQQIAAKQKREQMLQNPQMSTWQQTGPSHSSLDVPYPME---KPASPSSYKQDFTNSKLLMMPSV-NKSSPRPGGP--YLQPS-HVNPSNLNQNSANNQGSVLDYGNTKPLSHYK-ADCGQ--GSPGSGQSKPALMAYLPQQL-HISHEQNSLFLMKPKPG---NMPFRSL-VPPGQLSSQQQAAVMKQHQLLLDQQKQREQQQKHLQQQQFLQRQ------QHLLAEQQRHLTRPPPQRVFPQAGNLMPMGYGMASGRQTNVGQNTSVSAAYGGNSN-VSPFQQAHLKMSSPQ-FSQASSVGSLLPPVSAQQRTPELGAFSQSPA-SQMGQAYPV--RTAGQELPFAYDLIDSLLKNRTSEEWMSDLDDLLG

>Xtr2

PRHSAVMERLRRRIELCRRHHGTCESRYEALAGERLELERQ-HTFQLHQRCLQAKAKLHETVKRKLDNAASPQNGDQQNGYGDMFSVP-----KKLRHDDGLGGVIG---SS-N-GMPPVSPLNQLDNKPPSGDSMQLNGNHSIGL--DSHSKKCLPDGSLQLNGNGD-ADDFSLC------VKQEPVDDLPCQNNL-MPDLNLNEQEWKELIEELNKS-VPDEDMKDLFNDDFEDKKDVDASNSATQTPLAQD-IHVKTEFSPA----AFDQESH-GSPQVRSASSGP-FVGAPSAPASSASPVS-SAQQLQQIAAKQKRDQLLQSQQMPNWPQSRTSQSPLGVPYTME---KPTSPSVYSQDFTNQK-IVMPNL-NKSSPRGGAN--YMQPN-HVNANNLNTNPAAAPNAMLDYGNTLPLSHFE-VDC----GPGVVNQNKPGMLPYPQRQQQHMAEEQNQMFLLKRKAG----MQYRPL-VPHSQLSGQQQAVMKQ--QLLLEQQKQRE--QKQLLLEQHKQQIQMAQRQQHLLAEQNLFRVRMLPQRMFSQTQQMMQMGYNMASGRQASIGQGNPLPAGYGGN-SGLGAFQQTHHKMGNQQ-FGQGNP-GQMMPNITSQQRTADITPFSQNTG-QQMTQNYQV--RSASQDLPFGYDLLDSLLKNRTSEEWMNDLDELLG

>Gga2

PRHSAVMERPFQRIELCRRHHSACESRYQAVSPERLELERQ-QTFALHQRCLQAKAKLHETVKRKLDSASSPQNGDQQNGFGD--VFS---VSKKLRRDDGLGGVSG---SS-N-GMPPVSPLHHLDKKSGSGDTLQLNGKHPMGL--DGISKKCLPDSSLQLNGGGDADDSFPLSLN--KELKQEPVDDLPCQNNL-MPDLNLNEQEWKELIEELNRS-VPDEDMKDLFNEDFEEKKDADSSNSAAQTPLPQD-INIKTEFSPA----PFEQEQM-GSPQVRSTSSGSAFIGAASVPVSATSPVS-SAHQLQQIAAKQKRDQMLQNQQMSTW-QSGPSHSPLAVPYTME---NPTSPSVYQPDFNNQK-LMMPNMGNKSSPRAGGN--Y-----HVN-NSMNQNPVNSQGSMLDYGNTKPLSHYK-AECEQG-V-AVPGQNKTPMLAYIQQRQQHVSDDQNGMILLKPKSG---NITYR---LPHSQLSNQQQAAVMKQHQMLMDQQKQREQQQKHLLMEQQKQQFLMEQRQQHLLVEQQRHLTRPPPQRMFPQTQNMIQMGYSIPSGRQGSLGQGAAVPAGYGGNS-GLGPFQQTHLKMANQQ-FAQGNAGQMLSSSLGTQQRTPDLNAFNQNPN-QQMPQGYPV—RTSSQELPFAYDLIDSLLKNRTSEEWMNDLDELLG

**MINDBOMB**

>Nve1

AGIRVVRGPDWKWGNQDGGEGSVGTVVIVQWDTGNRTNYRCGYQGVYDLYLYDNGGVEHSHISCSECHQQG-IKGMRWQCADCEGYNLCTACYMGDKHELQHGFYLHESPDSSSVPVGKRYGMEKCQ-SRGIFKGAQVARGLDWDWGDQDGGIGKVGRVTGVKGWDKESYRSVVSVNW-SLKGENVYRLGHKGKV----DLTCVNATPGGYYYREHLPKVGLLVGDQVKMELEEHLLKSLQCGHGNWNEKM------------------------------------------RMVSTM-------------------------------------------------------------------------------------------------------------------------------------------------------------------------------------------------------------------------------------------------------------------------------------------------------------------------------------------------------------------------------------------------------------------------------------------------------------------------------------------------------------------------------------------------------------------------------------------------

>Cel

------------------------------------------------------------------------MNGV---AGESPSYD---------------EYLTYFTPGDRILISENHPNHAQRL-------------------------------------------NSYNNVAW---TKTHLALLGHIGYIESINEHRHTANVR-VYYAVPQNPEL-FKLSTEWPLD-ALEFPQCIDHSKGDL----------VAITRGD-PEKTTIGI--KEPLNLGGIPKLKSASNPTITREIQVAVNANTG---WTDER------------------RDIF-------LCP---VYSGPRLEPSVHTYKHTIHYTPDTLMQVIANWGEDALSYALLVESIRSVRTLFDGQLPLFRAVADDLRNVVVMLVALGADRTARDSENRTIIHVAAERGLDKMLDTVMLLLPKD----------INSQAANGLTPLH-LAARHAHAACIDRLLGTSTCIPCV-----PNNFGDTLLHEVCRLPESSNKKAAISRILTNTRAN-----IHHVNNSNMTPIQIAIMSGHVSTV--EQLLLLRAS---YRNTTSKTGMSALHFAAASGHANVVNKLISLG-LEVHRRDRGVLHYALRLAAIQALVKAGAPSNIIDLNGQTPVF--QLIREMLSNSEQYPASLVLATICFLVANGADLNVKDRRGMT---VMDLC-------EESSFRSIIMMAMSE-DKFD---------STEVTMCTFS------CLNSVATVKLDPCGHRVACVDCTEKVAIRRCPVCLRKRLEQLELE-TNCAICMDLKIAVVF-NCGHT-ACVDCADKLKKQCHICR--------KTIETMQPI

>Aqu

VGTRVVRGPDWKWGKQDGGEGRVGTLVMVIWDAGTAANYRCA--SHFDLRILDNSGVHHETYSCNGCQSTS-IYGIRWECMECPTINLCSLCYHGDKHSLRHQFYRISTPSSKKVSVPVRKKSKKIS-SRGIFPGARVVRGLDWNWDDQDGGEGHKGKVTKIESWNGASPRSAASVVW-DHGVENLYRIGFEGMV----DVKVVTEAKGYTYYRDHLPLVGYEVGDNVCVDSDPDTVRYLQHGHGGWAESMREVIGVVGVISGI-DDDGDIVVQYRWTFNPVALKKYLPGDVVKVHSDREYVKRQQQGHGDWVESMTMVRNMNNTVTLGQVGRVIEVIQSGDIIAVCGTNWTFSPAVLQNVSVMLRQVFENPVEELFKAAAS-GDIGRVEEVL----SQVDESFDGQTPLHIACQNGYRDVVRFLINKGANPEEEDKDGDQAIHFAT-IG--DEPDIIELLASHGVD--------LNTRNRRQQTPLH-IAVTKGYNIVIECLLKHN-CHPSL-----QDAGGDTPLHDA--ISKKRDD---ITELLLVGGAD-----------------------------------------------------------------------------------------------ITLLVEEDANVNIPDRYGDTPLH-CAIRQHTLTQLKELKESPQSSVAIFLASHGADFSLTNSSNQR---PLDLC-------SDPSLIKTLCMKCHS-LDRDVLFSP----CGHIAVCSICSAECLVCSQRLADVLFKPCLHMVACESCAIV--MKKCVHCLQQQLQVLREKGLIVP------------------TSQSNLSRL-SGCKLKM--------LSIDRELEF

>Nve2

-----------------------------------------------------------------------------------------------------------------------------------------------------------------------------------------------------------------------------MTKNDQSE-----------------------------------------------------------------------------------------------------------------------------------------------------------------------------------------------------------------------------------------------------------------------------------------------------------------------------------------------------------------------------------------------------------------------------------------------------------------------------------------------------------------------------CMVCSD-NKRDTLFGP----CGHVATCSLCSPECMVCSEKKSQLLFKPCNHMVACEGCGSL--MKKCIQCLQQQLQDMKEQ-TMCPVCMDRRKNLIF-LCGHG-TCQLCGDRM-QECPMCR--------KTVERRILL

>Hma

--------------------------------------------------------------------------------------------------------------------------------------------------------------------------------------------------------------------------------------------------------------------------------------------------------------------------------------TLGKLGCVTKVYHDGDLKVVNGTSWTYNPKCLTNSASKILHFLEDPAELFIKAAAE-GNVKNLEELC----HFINVLYAGHTALQLACQNGKLESIKFLLQLNADVEVEDIDGDKAVHHAT-FY--DESRTFALLKEANAD--------LNSRNKRRQTALH-VAVNKGHIGNIKALLDAG-VHVNL-----QDSEGDTALHDA--ISKKRDD------------------------------------------------------------------------------------------------------------IVELLLNSGTDISL-----------------------------------------------------------------------------------------------------------------------------------------------------------------------------------------------------------------------

>Bfl

--------------MGNALDAELGRIVKVEHDLMTLQLLQRG-HGGFTLPML------------ECVSTEGTVEGVDQDGDIVVRY-----------------------PSGNRFCLNP-DALTKVG-----GDDSTALRSGDWVRVSDDHGRVQR--------------QQAGHGGWNNDMAASLGKVGRVVHVFPDRDMKVDVGGRVWCFNPASLTKVSLSVGDLVKIDGNASRVRTMQEGHGGWVPAMAASLGQVGRVVAVGADRAKVEI--GWLYNPDVLTK-----------------------------------------LGKVGRVVKVDSDNDMTVVDGTDWLFNPGSLEKTSMCAKGTHQEDIGGLMRHLAAGRDQLTVLDLLKAALERGEGTPRGIKTLSPEERREKLYEHLPKMSKAIDMVKSKRDGGSVSTIKDTLE--ESLDGPYKTFPKKAERKIMGDHLANIGGARALTDYLKFLMNGKHTEEQVQLKCLH-VVRSL-----LWNFSDASFEFCRELGKSGLLTIVSQDLANFEGADLKHEVIHLVVLSALAVMHNCAKVPENRHFFQDIKAVDRIRPYLQVEDLNLKVPAVLTMAYLVEDRNSSLIEVDDSVADYVIRTELAMALGAKKGALKQLIALAQDGDSTDKEQAAKAL-QAFLHDPDVKAQLKKDREAYCRECHLSRGDQDYYKRGDPQKDYGLPVGWCRFSLKVPPRATALGVFTMEGSRLSERDGHYKDSWKPEGFDTKQVFVSPGTTYEAKVALQLYIKPGSYKVAASTVARK---------LDENLAMAWKGLWLCIFIATVGYSVAA-PVGREEALSSPMDSDAEDADIGSDYSYDTNFEDTRQRI--

>Hro

VGARVIRGPDWKWGKQDGGEGHVGTVVVVVWDNGTAANYRCS--GSYDLRIIDSAGQRHDGTMCDTCHQNP-IYGIRWKCAECNNYDLCCICYHGDKHNLRHRFFRINSPGCERALLDPRRKSKKIT-TRGIYPGARVVRGVDWQWEEQDGGAGRRGKVSDVQDWNNSCPRSAAYVLW-DTGTKNLYRVGFEGMS----DLKAVSDAKGNSYYRDHLPNLGLKVGDQ--------------------------CLGTTGSVVGI-DEDHDIVVSYRWTFNPAVLTKFCVGDFVKVCSDVERMKVLQRGHGEWAEAM--------VPTLGKVGRVQQVYQDNDLKVVCGTSWTYNPQAVERLSVLLKKLFEDVNEELVKAAAN-GDLQK-----------------------------------------------DKDGDRAVHHAA-FG--DEPEVIKLLQMGGAD--------LNARNKRRQTPLH-IGVNKGHIGVVKVLLELG-CHPSL-----QDSEGDTALHDS--VSKKRDD---ILQILLEHSAD-----MTITNNNGFNTLHHAALRGNPSAM---RILLSKLSRPWL-----------------------------------------------------LLVREGCNLNISDKDGDTPLH-EALRHHTLSQLRQLQDMQDASIACFLAANGANLDFKNKKGQS---ALDLC-------PDPNLCKALCMVCSD-TKRDTLFGP----CGHVTTCSLCSPECVVCSDRKASVLFRPCGHMCACETAMTS--TSAATAALQQQLQDIKDQ-TSCPVCMDRLKNMIF-LCGHG-TCQMCGDRM-LECPICR--------KTIEKRILL

>Aae1

VGSRVIRGPDWKWGKQDGGEGHVGTVVVVVWDNGTAANYRCA--GAYDLRILDSAGIKHEGTMCDTCRQTP-IFGIRWKCAECNNYDLCSICYHGDKHHLRHRFHRISTPGGEKTLLEPRRKSKKIA-VRGIFPGARVVRGVDWQWEDQDGGNGRRGKVNEIQDWSSASPRSAAYVVW-DNGAKNLYRVGFEGMA----DLKVVNDSKGNNVYRDHLPLLGFQIGDQVTVDLEIEIVQSLQHGHGGWTDGMYECLNTTGTVVGI-DEDHDIVVAYRWTFNPTVLTIFAVGDFVKICSDLERIKILQRGHGEWAEAM--------VPTLGKVGRVQQVYHDNDLKVVCNTSWTYNPLAVERLSAILKKLFEDTTEELVKAAAN-GDVAKVEEFLSGSSAQVNGVFAGHTALQAASQNGHLEVIQVLLRYNADVEIEDKDGDRAVHHAA-FG--DEPGVMGLLAKAGAD--------LNARNKRRQTALH-IAVNKGHFNVVKTLLELS-CHPSL-----QDSEGDTPLHDA--ISKEHDN---MLSLLLDFGAD-----ITLTNNNGFNALHHAALKGNPSAM---KILLTKTNRLWIVEEKKEDGYTALHLAALNNHVEIAELLVKMGKANMDCQNQTALHLAVHVQIVKLLVREGANLNIPDKDGDTPLH-EALRHHTLSQLRQLQDVEGASIACFLAANGADLTIKNRKLQT---PLDLC-------PDPNLCKTLCLLCSD-QKRDTVFKP----CGHVVCCDNCGPECLVCSDRKASVFFKPCGHMVACDNCAQI--MKKCVQCLQQQLQDIKEQ-TMCPVCFDRMKNMVF-MCGHG-TCQMCGDQI-EGCPICR--------KTVEKRILM

>Aae2

-----------------DGEGHVGTLVVVNWDSGHRTNYRVGYQKQYDLIVVDNAGVKHPNIICDGCNKPG-IAGIRFRCADCANYDLCATCYGNDVHDLEHSFIRYQTANSVGVRVPPRKGALKIQ-LKGIFVGARVVRGPDWEWNNQDGGPNKTGRVMEIRGWDNESCRSVANVSW-ASGSTNVYRLGHKGNV----DLRFVQPAVGGYYYKDHMPVLGFNVGDRVQVAIPEERLMSLQQGHGGWNPRMAEYLSKIGIVHRI-TDKGDIRVQYRWTFHPAALIKFNVGDIVTFITDAVKMQQLQKGHGEWVETM--------HNVLGKSGKVIKIYGDGDLRVDDDLAWTVNPKCVTERSNSMMDLSNSAADRLVREAAQ-GNMNFVQNYL----SAIDCVSGGKTCLQVAAHQGHVDLVKHLLQLGANVNVVDKEGDSTLHYAA-FG--NQPEIMRILLQHNAN--------IDVLNSSHCSALH-ISAHKKPPHCVKVLLEFG-ANVNV-----QDAYGDTALHDA--IGKENTE--VVELLCACSTLD-----LTIRNKRGFNALHHASLKGNVHAA---RNIIRLARQ--LVNVRKDDGFSALHLAALNGHSKVVEVLVKEGQADINIRNQTPFLLAVHTAAIEKLVELKCEIAAKDEDGDNAMHLCIIKKANLVQDVTATDSPKYALLCFLAKEGCPLDV-NYKGAR---VLDWI-------PSQPVKDIIPNVVQEEDSKRMNIEP-------LAPSEASGPECIVCNEQMLLIIFDPCQHQISCEECGIR--MKKCLSCLESKIMEIEET-HCCSICMERRRNVAF-LCGHG-ACSKCAETL-KICHMCR--------KTITKKINL

>Spu

VGARVTRGLDWKWGKQDGGEGHVGTVVVVVWDNGTAANYRCA--GAYDLRILDSAGIKHDGSMCDTCRMQP-IYGMRWKCAECPNYDLCSVCYHGDKHHLRHRFYRINTPNSERVVLEPRRKSKKIM-ARGIYPGARVVRGVDWEWEDQDGGMSRRGKVTEVQDWSATSPRSAAYVIW-DNGAKNLYRVGYEGMS----DLKVVSDAKGGYFYRDHLPNLGFSLGDQVNVDLELEIVQTLQHGHGGWTDGMFETLGTTGTVVGI-DEDHDVVVSYRWTFNPAVLTKFQIGDLVQICSDLERMKILQRGHGEWAEAM--------LPTLGKIGRVQQIYHDNDLKVVCGTSWTYNPTAVERLSALLKKLFEDVNEELVKAAAN-GDAHKCEEIM----QRVNVQFAGHTALQAASQNGHRDVLKVLIQ------ISDKDGDRAIHHAA-FG--DEPHVIELLHEESAD--------LNARNKRRQTALH-IGVNKGHYGVVKILLELA-CHPSL-----Q----------------------------------------------------------------------------------------------------------------------------I----------------------------------------------------------------------------------------------------------------------------------------------------------------------------------------------------------------------------------------

>Lgi1

--------------------------------------------------------IKHDGTMCDTCRQQP-IFGIRWKCAECPNYDLCSVCYHGDKHNLRHRFYRITVPGNERVLLEPRRKTKKTT-ARGIYPGARVVRGVDWQWEDQDGGNGRRGKVTEIQDWSAASPRSAAYVLW-DNGAKNLYRVGFEGMA----DLKVVNDAKGGSFYREHLPLLGLAIGDQVNVDLDLEIVQSLQHGHGGWTDGMFECLGTTGTVVGI-DEDHDIVVSYRWTFNPAVLTKFAVGDLVQICNDVERIKVLQRGHGEWAEAM--------IPTVGRIGRVQQIYHDNDLKVVCGTSWTYNPSAVERLSALLKKLFEDVNEELVKAAAN-GDASKVDEIL----QRANGVFAGHTALQAASQNGHIEVIKVLMKYKVDMEVEDKDGDRAVHHAA-FG--DEPAVIDLLHRGNAD--------LNARNKRRQTPIH-IGVNKGHIGVVRMLLDLG-CHPSL-----QDSEGDTALHDA--ISKKRDD---MITLLLEHNGD-----ITIANNNGFTALHHSALRGNPSAM---RILLNKLPRPWIVDEKKDDGYTALHLAALNNHVEVAELLVQQGKANMDIQNQTPLHLAVHTQIVRLLVRESCNLNIPDKDGDTPLH-EALRHHTLSQLRQLQDMQDASIACFLAANSADLNIKNKKGQT---PLDLC-------PDPNLCKALCMVCSD-MKRDTLFGP----CGHIATCSLCSPECVVCSDKKATILFKPCGHMCACDGCAAL--MKKCVQCLSQQLQDIKDQ-TSCPVCMDRLKNMIF-LCGHG-ACQLCGDRL-NECPICR--------KAVEKRILL

>Lgi2

--------------------------------------------------------------MCDTCRQQP-IFGIRWKCAECPNYDLCSVCYHGDKHNLRHRFYRITVPGNERVLLEPRRKTKKTT-ARGIYPGARVVRGVDWQWEDQDGGNGRRGKVTEIQDWSAASPRSAAYVLW-DNGAKNLYRVGFEGMA----DLKVVNDAKGGSFYREHLPLLGLAIGDQVNVDLDLEIVQSLQHGHGGWTDGMFECLGTTGTVVGI-DEDHDIVVSYRWTFNPAVLTKFAVGDLVQICNDVERIKVLQRGHGEWAEAM--------IPTVGRIGRVQQIYHDNDLKVVCGTSWTYNPSAVERLSALLKKLFEDVNEELVKAAAN-GDASKVDEIL----QRANGVFAGHTALQAASQNGHIEVIKVLMKYKVDMEVEDKDGDRAVHHAA-FG--DEPAVIDLLHRGNAD--------LNARNKRRQTPIH-IGVNKGHIGVVRMLLDLG-CHPSL-----QDSEGDTALHDA--ISKKRDD---MITLLLEHNGD-----ITIANNNGFTALHHSALRGNPSAM---RILLNKLPRPWIVDEKKDDGYTALHLAALNNHVEVAELLVQQGKANMDIQNQTPLHLAVHTQIVRLLVRESCNLNIPDKDGDTPLH-EALRHHTLSQLRQLQDMQDASIACFLAANSADLNIKNKKGQT---PLDLC-------PDPNLCKALCMVCSD-MKRDTLFGP----CGHIATCSLCSPECVVCSDKKATILFKPCGHMCACDGCAAL--MKKCVQCLSQQLQDIKDQ-TSCPVCMDRLKNMIF-LCGHG-ACQLCGDRL-NECPICR--------KAVEKRILL

>Lgi3

VGARVVRGPDWKWGKQDGGEGHVGTVVVVVWDNGTAANYRCA--GAYDLRALDSAGIKHDGTMCDTCRQQP-IFGIRWKCAECPNYDLCSVCYHGDKHNLRHRFYRITVPGNERVLLEPRRKTKKTT-ARGIYPGARVVRGVDWQWEDQDGGNGRRGKVTEIQDWSAASPRSAAYVLW-DNGAKNLYRVGFEGMA----DLKVVNDAKGGSFYREHLPLLGLAIGDQVNVDLDLEIVQSLQHGHGGWTDGMFECLGTTGTVVGI-DEDHDIVVSYRWTFNPAVLTKFAVGDLVQICNDVERIKVLQRGHGEWAEAM--------IPTVGRIGRVQQIYHDNDLKVVCGTSWTYNPSAVERLSALLKKLFEDVNEELVKAAAN-GDASKVDEIL----QRANGVFAGHTALQAASQNGHIEVIKVLMKYKVDMEVEDKDGDRAVHHAA-FG--DEPAVIDLLHRGNAD--------LNARNKRRQTPIH-IGVNKGHIGVVRMLLDLG-CHPSL-----QDSEGDTALHDA--ISKKRDD---MITLLLEHNGD-----ITIANNNGFTALHHSALRGNPSAM---RILLNKLPRPWIVDEKKDDGYTALHLAALNNHVEVAELLVQQGKANMDIQNQTPLHLAVHTQIVRLLVRESCNLNIPDKDGDTPLH-EALRHHTLSQLRQLQDMQDASIACFLAANSADLNIKNKKGQT---PLDLC-------PDPNLCKALCMVCSD-MKRDTLFGP----CGHIATCSLCSPECVVCSDKKATILFKPCGHMCACDGCAAL--MKKCVQCLSQQLQDIKDQ-TSCPVCMDRLKNMIF-LCGHG-ACQLCGDRL-NECPICR--------KAVEKRILL

>Aqu1

IGVRVVRGPDWKWGQQDGGEGYVGTVVLVQWDNGSRCNYRCGIDGKYDLLLYDNAAVRHPNITCDSCRQNG-IEGLRYKCVNCFDFDLCFSCYMSSKHSMEHKFILQEAPEAPFVNLPLRCDSSRLV-AKGLFKDAEVTRGYDWLWGDQDGGIGNIGHLVTIKGWEKDTFRSVAEVEWKKGGKKNVYRVGHKGKV----DIKAITPGEYGYYFPDHLPVLGIAAGDQVRVQLDVDVFKALQEGHGGWNDDMAQLIEQMGTVHNV-LDSGDIRVRYTWTLNPASLTKFAVGDVIKIIDDIALVHDLQEDHGGWVDDM--------ALTLGQAGRVVRVFPSGDLRVVNGRSWTFNPLCMVDIETQLRLLTLENPAVVVAAAAA-NDTNALREFL----VKVNAKAAGKAALHCAAVAGHIEIIKCLLEFKANLEIEDEDGDRPLHLCA-YG--DEEEAAQLLIDNGAD--------VNARSKRGMTALN-LSAIKGHTSILKVLIRDQNIDLSA-----ADSEGNTPLHCA--VLAQKLE---AIVLLLDAGGD-----PSLVNFRLFTPLHEAARIGFLPGV---DLFIKRNPE--CVNLKKDDGLTPLHLACLNNHLDVAT----------------------------------------------------------------------------------------------------------------------------------------------------------------------------------------------------------------------------TIAEC-----------------------------

>Dre1

VGARVIRGPDWKWGKQDGGEGHVGTVVVVVWDNGTAANYRCS--GAYDVRILDSAGIKHDGTMCDTCRQQP-IIGIRWKCAECTNYDLCTTCYHGDKHHLRHRFYRITTPGSERVLLESRRKSKKIT-ARGIFAGGRVVRGVDWQWEDQDGGNGRRGKVTEIQDWSAASPHSAAYVLW-DNGAKNLYRVGFEGMS----DLKCVQDAKGGTFYRDHCPVLGLQIGDLVNIDLDLEIVQSLQHGHGGWTDGMFETLTTTGTVCGI-DEDHDIVVQYRWTFNPAVLTKFMVGDLVQICYDIDRIKLLQRGHGEWAEAM--------LPTLGKVGRVQQIYSDSDLKVVCGTSWTYNPAAVERLSQLLKKLFEDINEELVKAAAN-GDLAKVEDIL----KRVNGQCAGHTAMQAASQNGHVDVLKLLLKHSVDLEAEDKDGDRAVHHAS-FG--DEGSVIEVLHRGGAD--------LNARNKRRQTPLH-IAVNKGHLQVVKTLLDFG-CHPSL-----QDSEGDTPLHDA--ISKKRDD---MLSVLLEAGAD-----VTITNNNGFNALHHAALRGNPSAM---RVLLSKLPRPWIVDEKKDDGYTALHLAALNNHVEVAELLVHQGNANLDVQNQTALHLAVHTQIVRLLVRAEAKLDVQDKDGDTPLH-EALRHHTLSQLRQLQDMQDASIACFLAANGADLTIRNKKGQS---PLDLC-------PDPSLCKALCMVCSD-MKRDTLFGP----CGHIATCSLCSPECVVCSDKKAAVLFQPCGHMCACENCASL--MKKCVQCLQQQLQDIKEQ-TMCPVCLDRLKNMIF-MCGHG-TCQLCGDRM-SECPICR--------KAIERRILL

>Dre2

VGMRVVRGADWKWANQDDGEGHVGTVVVVQWDSGTRTNYRSGYQGAFDLLLYDNAGVRHSNIICDSCKKHG-IMGMRWKCKVCFDYDLCTQCYMNNKHGLTHAFERYETAHSQPVSLTPRQNLSRII-LKGIFQGVKVVRGPDWDWGNQDGGEGKVGKVVDIRGWDQESGRSVASVTW-SNSTTNVYRMGHKGKV----DLKYVSDVQGGFYYKEHLPKLGFQQGDKVKCLLEVDILRQMQEGHGGWNPKMAEYISRIGTVHRI-TDRGDVRVQYRWTFHPGALTKFAVGELVKVLDDIDSVKRLQVGHGEWTDSM--------APALGQVGKVLKVYADGDLRVFGGQTWTFNPACLSESSSTVISVLEDNPGRLVIEAAH-GSAAKVRELL----QKVDIKNQGKTALQVAAHQGHVEVVKVLLQANSSIEAKDEDGDAALHYTA-FG--NQAEIARLLLSKGAS--------VNLLNNSMCTALH-IAVNKGFTDVVRVLTEHS-ADVNL-----QDSYGDTPLHDA--IAKDFRS--IIEILTVVPNID-----FTQQNNRGFNLLHHAALKGNKLAT---EMILSRARQ--LADVKKEDGFSALHLAALNNHRDVAEILLKEGRCDINIRNQTPLQLAVHMALVALLVMEGADVNAEDEDGDTAMH-TSLSRQQLTTV--ISSAEGAAIACFLAQEGADISYANHKGKS---PLDLV-------TDSSVQTLISSSSSS-SLRRVHTTPN--TMTNLAVPSVTGPECLICSELALLVLFSPCQHSVACEECAHR--MKKCIRCLQSRYRQMEER-ITCPICIDNHIRLVF-QCGHA-SCIDCSSAL-KTCPICR--------QTIRERIQL

>Dre3

------------------------------------------------------------------------------------------------------------------VSLTPRQNLSRII-LKGIFQGVKVVRGPDWDWGNQDGFERKVTSLFGLSLLMIQYLNSQKQIPT--------YYDYYKPKP----FFYYYSYSYFIFYYYLLLLFSCFEVLECVCVISSISCYRQMRYIRERARERLSDVISTCFLPGKF-TDFYDLRSFSQTYLCKETNDKFIELNLYHIIFFFDSKTHTHTHRDEHTNTP--------PPALGQVGKVLKVYADGDLRVFGGQTWTFNPACLLLSTGTVISVLEDNPGRLVIEAAH-GSAAKVRELL----QKVDIKNQGKTALQVAAHQGHVEVVKVLLQANSSIEAKDEDGDAALHYTA-FGEISSPEVCRLLLYVCVC--------VCLFFVYMCVSLYFVLVQKKLLDCVHVCV----CVVCV-----QDSYGDTPLHDA--IAKDFRS--IIEILTVVPNID-----FTQQNNRGFNLLHHAALKGNKLLVFLGKMILSRARQ--LADVKKEDGFSALHLAALNNHRDVAEILLKEGRCDINIRNQTPLQLAVHMALVALLVMEGADVNAEDEDGDTAMH-TSLSR-QQLTTVISSAEGDAAIACFLAQEGADISYANHKGKS---PLDLV-------TDSSVQTLISSSSSS-SLRRVHTTPN--TMTNLAVPSVTGPECLICSELALLVLFSPCQHSVACEGLVHL--YTKCIRCLQSRYRQMEER-ITCPICIDNHIRLVF-QCGHA-SCIDCSSAL-KTCPICR--------QTIRERIQL

>Cin1

VGSRVVRGPDWKWGRQDGGEGHVGTVVVIVWDNGTAANYRCS--GQYDLRILDSAGLKHNGSMCDTCRLQP-IFGIRWKCAECHNYDLCSACYHADKHHLRHRFYRIVTPDGERVLMESRRKSKKIS-SRGIFPGARVVRGVDWQWEDQDGGNGRKGKITKIQDWTSQHLRSAAYVLW-DVGAKNLYRVGFEGMM----DLKIVTDAKGPSFYRDHLPCLDFQLNDQVNIDLDLEIVQSLQHGHGGWTEGMFETLGTTGTVCGI-DEDHDIVVSYVAHYTPGPIRKGLVGDLVQISSHVEHVKTLQRGHGEWSESM--------LAAVGKVGRVQQVYRDSDVKVVSGTSWIFNPLLLENLSLLLKKLFEDINEELVKAAGS-GDVQKCEEIL----QRVNGQFAGHTAMQAACLNGHLDVVSCLIQHDADLEIEDKDGNRAIHHSA-FV--DEAGVVELLVREGCD--------ANARNKRRQTALH-IAVNRGHVWVVQTLLELG-CHASL-----QDAEGDTPLHDA--ILKKRDD---MLTFLLDAHAD-----VTVTNNNGFNVLHHASLRGNPSAM---CILLNKLTRPWIVDEKKDDGYTALHLAALNNHLEVAELLVKLGHANLDIQNQTPLHLAVHTQIVRLLVREGACVNLTDKDGDSPLH-EALRHHTLWQLRTLQDKHDASIAQFLAANGADLNLKNKDQHN---PLDLC-------PDPNLCKSLCMICSD-SPRDTILEP----CGHSLACASCSDECCVCSDKQSSVVFKPCGHICACQACSNL--MKKCIRCLQQQLQDIKEQ-VMCPVCMDRIKNMIF-LCGHG-TCQLCGDRM-TECPICR--------KPVEKRILL

>Cin2

VGSRVVRGPDWKWGRQDGGEGHVGTVVVIVWDNGTAANYRCS--GQYDLRILDSAGLKHNGSMCDTCRLQP-IFGIRWKCAECHNYDLCSACYHADKHHLRHRFYRIVTPDGERVLMESRRKSKKIS-SRGIFPGARVVRGVDWQWEDQDGGNGRKGKITKIQDWTSQHLRSAAYVLW-DVGAKNLYRVGFEGMM----DLKIVTDAKGPSFYRDHLPCLGFQLNDQVNIDLDLEIVQSLQHGHGGWTEGMFETLGTTGTVCGI-DEDHDIVVSYPPVFGLSLSRMADVGDLVQISSHVEHVKTLQRGHGEWSESM--------LAAVGKVGRVQQVYRDSDVKVVSGTSWIFNPLLLENLSLLLKKLFEDINEELVKAAGS-GDVQKCEEIL----QRVNGQFAGHTAMQAACLNGHLDVVSCLIQHDADLEIEDKDGNRAIHHSA-FV--DEAGVVELLVREGCD--------ANARNKRRQTALH-IAVNRGHVWVVQTLLELG-CHASL-----QDAEGDTPLHDA--ILKKRDD---MLTFLLDAHAD-----VTVTNNNGFNVLHHASLRGNPSAM---CILLNKLTRPWIVDEKKDDGYTALHLAALNNHLEVAELLVKLGHANLDIQNQTPLHLAVHTQIVRLLVREGACVNLTDKDGDSPLH-EALRHHTLWQLRTLQDKHDASIAQFLAANGADLNLKNKDQHN---PLDLC-------PDPNLCKSLCMICSD-SPRDTILEP----CGHSLACASCSDECCVCSDKQSSVVFKPCGHICACQACSNL--MKKCIRCLQQQLQDIKEQ-VMCPVCMDRIKNMIF-LCGHG-TCQLCGDRM-TECPICR--------KPVEKRILL

>Cin3

VGSRVVRGPDWKWGRQDGGEGHVGTVVVIVWDNGTAANYRCS--GQYDLRILDSAGLKHNGSMCDTCRLQP-IFGIRWKCAECHNYDLCSACYHADKHHLRHRFYRIVTPDGERVLMESRRKSKKIS-SRGIFPGARVVRGVDWQWEDQDGGNGRKGKITKIQDWTSQHLRSAAYVLW-DVGAKNLYRVGFEGMM----DLKIVTDAKGPSFYRDHLPCLGFQLNDQVNIDLDLEIVQSLQHGHGGWTEGMFETLGTTGTVCGI-DEDHDIVVSYPPVFGLSLSRMADVGDLVQISSHVEHVKTLQRGHGEWSESM--------LAAVGKVGRVQQVYRDSDVKVVSGTSWIFNPLLLENLSLLLKKLFEDINEELVKAAGS-GDVQKCEEIL----QRVNGQFAGHTAMQAACLNGHLDVVSCLIQHDADLEIEDKDGNRAIHHSA-FV--DEAGVVELLVREGCD--------ANARNKRRQTALH-IAVNRGHVWVVQTLLELG-CHASL-----QDAEGDTPLHDA--ILKKRDD---MLTFLLDAHAD-----VTVTNNNGFNVLHHASLRGNPSAM---CILLNKLTRPWIVDEKKDDGYTALHLAALNNHLEVAELLVKLGHANLDIQNQTPLHLAVHTQIVRLLVREGACVNLTDKDGDSPLH-EALRHHTLWQLRTLQDKHDASIAQFLAANGADLNLKNKDQHN---PLDLC-------PDPNLCKSLCMICSD-SPRDTILEP----CGHSLACASCSDECCVCSDKQSSVVFKPCGHICACQACSNL--MKKCIRCLQQQLQDIKEQ-VMCPVCMDRIKNMIF-LCGHG-TCQLCGDRM-TECPICR--------KPVEKRILL

>Cin4

LGYRVVRGTDWAWDNQDNGEGNVGTVLVVQWDCGTRTNYRIGYQGAFDLLVFDSAGIKHPSKQCDGCAERTMIRGTRWQCAQCKDYDLCTYCYMGGRHEKSHRFRRYETETSRGQEVSPRDAARAVIQSKGLFKGARVMRGYHWEWGEQDGGPTKLGKIVEIRGYQSESYRSVAQVTWDSGGTKNIYRVGHKGKV----DLKYVGPGYGPSYYREHLPVCGFKVGEKVIVTLEVEVLKPMLEGHGGWNSKMANIRGKVGVVHRL-TTAGDVRVQYRWTFHPAALE--------RVIDTAEQASKFQSSQ-------------------SKAGTSSSQSQHESLPLLGDYS-------LMPSVDSVRNSMEGTEGKTPLLEALDGPTGLDHKLI----NR------GVYPVHVVAQYFEKDRLIALQNKGANMEVVDLKGRTPLHCAV-VG--NKPQNVRWLMSAMKE--------RNLPDKNGYTPLH-RAVILDHTECCKEILSVK-AERTLYANTKDEIKENTALHLC--VSGHCNL--DIVHLVVEAGAD-----VDELNGAGVTCVALLVEKST-------ENLLSRIR------------------------------LPHSGKLWNSLQS---------------------KHNLVDKDGFVPRI--------------------LATLCYLVLNGARVENAASKL-----------------PFSNWQQCVTLRCSQ-ESFPGDNEP----STSRYPCRSC--------NQTACVKFDPCGHVVVCKKCSYI--VKKCLQC-LANVESTEDS-NSCTICMDRKINTVLSPCNHMLSCQECSKML-KQCPVCR--------EPIDKRVKV

>Xtr1

VGARVARGPDWKWGKQDGGEGHVGTVVVVVWDNGTAANYRCS--GAYDLRILDSAGIKHDGTMCDTCRQQP-IIGIRWKCAECTNYDLCTVCYHGDKHHLRHRFYRITTPGSERVLLESRRKSKKIT-ARGIFAGARVVRGVDWQWEDQDGGNGRRGKVTEIQDWSASSPHSAAYVLW-DNGAKNLYRVGFEGMS----DLKCVQDAKGGSFYRDHCPVLGLLIGDLVNIDLELEIVQSLQHGHGGWTDGMFETLTTTGTVCGI-DEDHDIVVQYRWTFNPAVLTKFQVGDLVQICYDLERIKLLQRGHGEWAEAM--------LPTLGKVGRVQQIYSDNDLKVVCGTSWTYNPAAVERLSQLLKKLFEDLNEELVKAAAN-GDVAKVEDLL----KRVNGQCAGHTAMQAASQNGHVDILKLLLKHSVDVEAEDKDGDRAVHHAA-FG--DEGTVVEVLHRGGAD--------LNARNKRRQTPLH-IAVNKGHLQVVKKLLDFG-CHPSL-----QDSEGDTPLHDA--ISKKRDD---ILAVLLEAGAD-----VTITNNNGFNALHHAALRGNPSAM---RVLLSKLPRPWIVDEKKDDGYTALHLAALNNHVEVAELLVHQGNANLDIQNQTALHLAVHTQIVRLLVRAEAKLDIQDKDGDTPLH-EALRHHTLSQLRQLQDMQDASIACFLAANGADLTIRNKKGQS---PLDLC-------PDPNLCKALCMVCSD-LKRDTLFGP----CGHIATCSLCSPECVVCSDKKAAVLFQPCGHMCACENCASL--MKKCVQCLQQQLQDIKEQ-TMCPVCLDRLKNMIF-MCGHG-TCQLCGDRM-SECPICR--------KAIERRILL

>Xtr2

VGMRVVRGIDWKWSNQDNGEGSMGTVVVVQWDHGTRTNYRTGFQGAYDLLLYDNAGVRHPNIICDCCKKHG-IRGMRWKCKVCFDYDLCTQCYMNNKHDLSHIFERYETADSRPVILSTRQGLPRVV-LKGIFQGAKVVRGPDWEWGNQDGGEGKVGRVVDIRGWDVETGRSVASVTW-ADGTTNVYRVGHKGKV----DLKCITDAPGGHYYRDHLPKLGFQHGDKVKCLLDVEILREMQEGHGGWNPKMAEFIGQTGTVHRI-TERGDVRVQYRWTFHPGALTKFWVGDVVRVMDNMDAVKKLQVGHGEWTDDM--------VSALGQIGKVIKVFGDGDMRVVGGQSWTFNPACLKESKNTLVSILEESPTSLVIEAAQ-GNTAKVREML----QKVDIKNQGRTALQVASHLGYMEVVKVLLQANANIDLRDDEGDTALHYAA-YG--NQAGVVRVLLSKGAN--------AELLNNAKCTALY-IAVNKGFTEVVQVLCNPN-CAINM-----QDSFGDTPLHYA--ITADFRS--IIEILTEVPNID-----FTVQNNQGFNLLHHSALKGNVLAV---SKILERARQ--LVDSKKEDGFTALHLATLNNHQEVVEILIKEGRCDVNLRNQTPLHLAVHISLVHLLVTEGADVNAEDEDGDTPMH-IVFVRQHLKSIDSQQEGNGSAMACFLALSGADINYANHRGKS---PLDLI-------ADGRITHLVSPTVSC-SLRRVHTTPN--TMTNLSPCSAASPECLVCSELAVLVSFFPCQHSIVCEECSRR--MKKCIKCLQNRYRQMEER-ITCPICIDNHIKLVF-QCGHG-SCTECSTSL-TACPICR--------QLIRERIQI

>Hsa1

VGARVVRGPDWKWGKQDGGEGHVGTVVVVVWDNGTAANYRCS--GAYDLRILDSAGIKHDGTMCDTCRQQP-IIGIRWKCAECTNYDLCTVCYHGDKHHLRHRFYRITTPGSERVLLESRRKSKKIT-ARGIFAGARVVRGVDWQWEDQDGGNGRRGKVTEIQDWSASSPHSAAYVLW-DNGAKNLYRVGFEGMS----DLKCVQDAKGGSFYRDHCPVLGLQIGDLVNIDLDLEIVQSLQHGHGGWTDGMFETLTTTGTVCGI-DEDHDIVVQYRWTFNPAVLTKFQVGDLVQVCYDLERIKLLQRGHGEWAEAM--------LPTLGKVGRVQQIYSDSDLKVVCGTSWTYNPAAVERLSQLLKKLFEDLNEELVKAAAN-GDVAKVEDLL----KRVNGQCAGHTAMQAASQNGHVDILKLLLKQNVDVEAEDKDGDRAVHHAA-FG--DEGAVIEVLHRGSAD--------LNARNKRRQTPLH-IAVNKGHLQVVKTLLDFG-CHPSL-----QDSEGDTPLHDA--ISKKRDD---ILAVLLEAGAD-----VTITNNNGFNALHHAALRGNPSAM---RVLLSKLPRPWIVDEKKDDGYTALHLAALNNHVEVAELLVHQGNANLDIQNQTALHLAVHTQIVRLLVRAGAKLDIQDKDGDTPLH-EALRHHTLSQLRQLQDMQDASIACFLAANGADLSIRNKKGQS---PLDLC-------PDPNLCKALCMVCSD-MKRDTLFGP----CGHIATCSLCSPECVVCSDKKAAVLFQPCGHMCACENCANL--MKKCVQCLQQQLQDIKEQ-TMCPVCLDRLKNMIF-LCGHG-TCQLCGDRM-SECPICR--------KAIERRILL

>Hsa2

VGMRVVRGVDWKWGQQDGGEGGVGTVVVVQWDQGTRTNYRAGYQGAHDLLLYDNAGVRHPNIICDCCKKHG-LRGMRWKCRVCLDYDLCTQCYMHNKHELAHAFDRYETAHSRPVTLSPRQGLPRIP-LRGIFQGAKVVRGPDWEWGSQDGGEGKPGRVVDIRGWDVETGRSVASVTW-ADGTTNVYRVGHKGKV----DLKCVGEAAGGFYYKDHLPRLGFQHGDKVKCLLDTDVLREMQEGHGGWNPRMAE---------------------------------FWVGDVVRVIGDLDTVKRLQAGHGEWTDDM--------APALGRVGKVVKVFGDGNLRVVAGQRWTFSPSCLRENKSSLSVALDEHPGRLVVEVAL-GNAARALDLL----RRVDTKNQGRTALQVAAYLGQVELIRLLLQARAGVDLPDDEGNTALHYAA-LG--NQPEATRVLLSAGCR--------ADAINSTQSTALH-VAVQRGFLEVVRALCERG-CDVNL-----PDAHSDTPLHSA--ISAGTGASGIVEVLTEVPNID-----VTATNSQGFTLLHHASLKGHALAV---RKILARARQ--LVDAKKEDGFTALHLAALNNHREVAQILIREGRCDVNVRNQSPLHLAVHVGLVPLLVDAGCSVNAEDEEGDTALH-VALQRHQLLPLVADGAGGDAAVACFLALEGADVSYTNHRGRS---PLDLA-------AEGRVLKALAAPGP----RQTLGTPN--TVTNLHVGAAPGPECLVCSELALLVLFSPCQHRTVCEECARR--MKKCIRCLQSRYRQMEER-ITCPICIDSHIRLVF-QCGHG-ACAPCGSAL-SACPICR--------QPIRDRIQI

>Hsa3

VGMRVVRGVDWKWGQQDGGEGGVGTVVVVQWDQGTRTNYRAGYQGAHDLLLYDNAGVRHPNIICDCCKKHG-LRGMRWKCRVCLDYDLCTQCYMHNKHELAHAFDRYETAHSRPVTLSPRQGLPRIP-LRGIFQGAKVVRGPDWEWGSQDGKPAELQR-----------------------------RVSADSQP--------------------------FQHGDKVKCLLDTDVLREMQEGHGGWNPRMAEFIGQTGTVHRI-TDRGDVRVQFRWTFHPGALTKFWVGDVVRVIGDLDTVKRLQAGHGEWTDDM--------APALGRVGKVVKVFGDGNLRVVAGQRWTFSPSCLRENKSSLSVALDEHPGRLVVEVAL-GNAARALDLL----RRVDTKNQGRTALQVAAYLGQVELIRLLLQARAGVDLPDDEGNTALHYAA-LG--NQPEATRVLLSAGCR--------ADAINSTQSTALH-VAVQRGFLEVVRALCERG-CDVNL-----PDAHSDTPLHSA--ISAGTGASGIVEVLTEVPNID-----VTATNSQGFTLLHHASLKGHALAV---RKILARARQ--LVDAKKEDGFTALHLAALNNHREVAQILIREGRCDVNVRNQSPLHLAVHVGLVPLLVDAGCSVNAEDEEGDTALH-VALQRHQLLPLVADGAGGDAAVACFLALEGADVSYTNHRGRS---PLDLA-------AEGRVLKALAAP----GPRQTLGTPN--TVTNLHVGAAPGPECLVCSELALLVLFSPCQHRTVCEECARR--MKKCIRCLQSRYRQMEER-ITCPICIDSHIRLVF-QCGHG-ACAPCGSAL-SACPICR--------QPIRDRIQI

>Hsa4

VGMRVVRGVDWKWGQQDGGEGGVGTVVVVQWDQGTRTNYRAGYQGAHDLLLYDNAGVRHPNIICDCCKKHG-LRGMRWKCRVCLDYDLCTQCYMHNKHELAHAFDRYETAHSRPVTLSPRQGLPRIP-LRGIFQGAKVVRGPDWEWGSQDGGEGKPGRVVDIRGWDVETGRSVASVTW-ADGTTNVYRVGHKGKV----DLKCVGEAAGGFYYKDHLPRLGFQHGDKVKCLLDTDVLREMQEGHGGWNPRMAEFIGQTGTVHRI-TDRGDVRVQFRWTFHPGALTKFWVGDVVRVIGDLDTVKRLQAGHGEWTDDM--------APALGRVGKVVKVFGDGNLRVVAGQRWTFSPSCLRENKSSLSVALDEHPGRLVVEVAL-GNAARALDLL----RRVDTKNQGRTALQVAAYLGQVELIRLLLQARAGVDLPDDEGNTALHYAA-LG--NQPEATRVLLSAGCR--------ADAINSTQSTALH-VAVQRGFLEVVRALCERG-CDVNL-----PDAHSDTPLHSA--ISAGTGASGIVEVLTEVPNID-----VTATNSQGFTLLHHASLKGHALAV---RKILARARQ--LVDAKKEDGFTALHLAALNNHREVAQILIREGRCDVNVRNQSPLHLAVHVGLVPLLVDAGCSVNAEDEEGDTALH-VALQRHQLLPLVADGAGGDAAVACFLALEGADVSYTNHRGRS---PLDLA-------AEGRVLKALAAPGP----RQTLGTPN--TVTNLHVGAAPGPECLVCSELALLVLFSPCQHRTVCEECARR--MKKCIRCLQSRYRQMEER-ITCPICIDSHIRLVF-QCGHG-ACAPCGSAL-SACPICR--------QPIRDRIQI

>Gga2

VGMRVVRGVDWKWGSQDSGEGNVGTVVVVQWDQGNRTNYRTGFQGAYDLLLYDNAGVRHPNIICDCCKKHG-IRGMRWKCKMCFDYDLCTQCYMNNKHDLSHAFERYETAHSQPVLVSPRQNLTRIT-LKGTFQGAKVVRGPDWEWGNQDGGEGKTGRVVDIRGWDVETGRSVASVTW-SDGTTNVYRVGHKGKV----DLKCTVEASGGFYYKEHLPKLGFQHGDKVKCLLDIDILREMQEGHGGWNPKMAEFIGQTGTVHRI-TDRGDVRVQFRWTFHPGALTKFWVGDVVRVIDDMETVKRFQPGHGEWTDEM--------APTLGHIGKVIKVYGDGDLRVVGDQSWTFNPACLKESKSTLITVLEDHAGCLVIWAAL-NNAAKVRELL----QKVDNKNQGRTALQIASYQGHLDVVKILLQAHATVNLRDEEGDTALHYAA-FG--NQADVARVLMAKGAG--------ADLLNNAKCTALY-VAVSQGFTEVVQALCELN-CDVNL-----PDSHGDTPLHYA--ITADYKV--IIEILTEVPNID-----FTVQNCQGFNLLHYSALKGNKLAI---KKILARARQ--LVDSKKEDGFTALHLAALNNHKEVAEILIKEGRCDVNVKNQTPLHLAIHVGLVQLLVSEGSDVNAEDEDGDTAMH-IALERQQLMSVLMEKREGETAIACYLAQEGADINYANHRGKS---PLDLI-------TDGRIVQIICSAITC-SLRRVHTTPN--TMTNLSVSSVAVPECLVCSELALLIHFFPCQHSIVCEECSRR--MKKCIKCLQNRYRQMEER-ITCPICIDDQIKLVF-QCGHG-SCPDCSTAL-TVCPICR--------QAIRERIQI

>Gga1

VGARVVRGPDWKWGKQDGGEGHVGTVVVVVWDNGTAANYRCS--GAYDLRILDSAGIKHDGTMCDTCRQQP-IIGIRWKCAECTNYDLCTVCYHGDKHHLRHRFYRITTPGSERVLLESRRKSKKIT-ARGIFAGARVVRGVDWQWEDQDGGNGRRGKVTEIQDWSASSPHSAAYVLW-DNGAKNLYRVGFEGMS----DLKCVQDAKGGSFYRDHCPVLGLQIGDLVNIDLDLEIVQSLQHGHGGWTDGMFETLTTTGTVCGI-DEDHDIVVQYRWTFNPAVLTKFQVGDLVQVCYDLERIKLLQRGHGEWAEAM--------LPTLGKVGRVQQIYSDSDLKVVCGTSWTYNPAAVERLSQLLKKLFEDLNEELVKAAAN-GDVAKVEDLL----KRVNGQCAGHTAMQAASQNGHVDILKLLLKQNVDVEAEDKDGDRAVHHAA-FG--DEGAVIEVLHRGSAD--------LNARNKRRQTPLH-IAVNKGHLQVVKTLLDFG-CHPSL-----QDSEGDTPLHDA--ISKKRDD---ILAVLLEAGAD-----VTITNNNGFNALHHAALRGNPSAM---RVLLSKLPRPWIVDEKKDDGYTALHLAALNNHVEVAELLVHQGNANLDIQNQTALHLAVHTQIVRLLVRAGAKLDIQDKDGDTPLH-EALRHHTLSQLRQLQDMQDASIACFLAANGADLSIRNKKGQS---PLDLC-------PDPSLCKALCMVCSD-MKRDTLFGP----CGHIATCSLCSPECVVCSDKKAAVLFQPCGHMCACENCASL--MKKCVQCLQQQLQDIKEQ-TMCPVCLDRLKNMIF-LCGHG-TCQLCGDRM-SECPICR--------KAIERRILL

**NEDD 4**

>Mbr9

------------------------------------------------------------------------------------SVP--------------------SRVLKDAVRIQFINELGMEEAGIDQEGLFKEFLEQTLKEGFNPDYGLFCLT--ADNKLYPSSTSEVHQEHLRLFEYLGRMLGKMLYEGIVVDIPLAHFFLNALLARPNTLDELSTLDADLARNLMVKTYDDVED--LGLVFAVDEEVLGERHTVPLRPGGSAVDVTNENRVLYVHLMADYKLNQQLRRQVQACRHGFNEFVHGSWLSFFNAPELQRLVSGDDVLDVNDLRHHANYEAGFHSSHRVIKWLFEVVKDLSREEQEQFLRFVTSCSKPPVLGFAALQGEGQADQYTLRNFGTDTTLPTSSTCFNTLKLPMYKSKRVLREKLKAAISSSSGFE

>Mbr7

------------------------------------------------------------------------------------------------------------------------------------------------MPLLDPDA--------DRH-----------------YKFLGTIVGRALRAEMLVDLTFCNFFLTQILGGRVSLNELRDLDAELYQNLQVKDYADVSE--LDLTFTVAGSEVTGHRLYNLVPDGANVAVTNENRIRYVYHMADFYLRRRTRQQIVAFQQGLEQAVPAALLRLFAPAELRKLIRGEKQIDVEEFRRHVVYN-GFRADDVPIQLFWSVVHEMDNDAREQLLRFITSCPRPPILGFQAMH-----PRIAIANSQDPSRLPSAATCMNLLKLPPYTSREILKDRLYKAIYETEGFG

>Mbr6

PPEYPGQTNDPAH----CTAQSPDSVWCPPLPAGWSSVIQA-GRHLFHADRRTTFVDPRLPPRFQRSQWAL-AHFPWLERTT--LLQMSSLLVLQREGLMRDSWLA-MTALPVHQLQRRLFVSFEGEAGRDFGGLAREWMDLMCNGMS------------HHLPLALVPTMTPTKEALDTIEFLGRVVGLALFHGRLINPHFSLIVYKMLLDQPCASLDLATVDPEMHHGY--V---------LAICSN-------DVCRTDLFSVGSTKPVTDANKRDYVDCLLQWRLNRGVAELLEAFKIGLTSFIPLSALAGFTAPRLRLLVSGLQQIDVREWRTATAYVNGYGEDAIQVQWFWQWVDQADEAQRAKLLTFCTGSTQLPAQGFHGLSGIHGYCPFVIARVGDVDRYPAAHTCVNRLDLPAYASRDVLHARLSFAVQETEGFG

>Mbr3

---------------------------------MPGQLSAPN-----RELRFLQFLEAH--RP---FEVKE-KFFRKKIKPDRHRVP----LRIRRDYLFEDSYQRVMQLNAGELR-GRLNVQFQGEEGIDAGGLLREWYYTISQSIMNPNYALFCQSTPGSETYQPNQHSSINVDHLRYFQFCGRVVAKAIFDHQLLDCHFTRAFYKQILGMHVSWRDLAAVDSSLYKNLLFILENDVTPFEGDFTFSLDVDRFGKLETIDLKPGGRDLNVTEENKKEYVRLVADMKLTEAIKDQIKAFQKGFYEVIPQTDIALFNESELELLISGLPEVDIDDLRANTDYHSGLSASTPVIQWFWRAVRSFSRDERIKLIQFVTGTGRIPVGGFSKLVGMSGPQKFNIQKRSGPQRLPQAHTCFNQLDLPEYESYEQLREALKLAIMASEGFG

>Mbr12

-------------------------------------------------------------------------------------------------------------------------------------------------------------------------------------------------------------------------------------------------------------VFGEKQHHELCPGGAKRSLSQANKHEFVRLYTDWVLNVSVEHQFAAFKYGFTVMAQSATLRMIDVSEMRALICGLEDLDLNQLREVARYEGGYNANSPVIKWFWEVALNFSDNEKRAFLQFTTGTRRAPVGGLRTMK-------FIIAKQGADSELPTSHTCFNALMIPEYSSKEKLEQLLKKAIQYSEGFG

>Mbr11

-----------------------------P---EWLQEHLPG----------------------------------RLAAAESNRAQHSPTLTVDRSALLDSSCQRLLRMDALELQ-RALDITFADEAG-QGQGVVREWFNLLSKELFNPDYALFVDSED---GAIIHSGSHVNADHLSYYRFAGRLLALAIIHRITPGIQLSRVLICQMMGLPPSLQDLRLLDPQFVQSTQWLLDHDVEQAGLDLTFAVDVFEYGQQTTVELIPRGASIAVT--------------------------------EVV----------DEVQALVAGQHQLDVNAWRAHTEYR-------------W----GMSEAQRALLLQFATGSSKLPHGGFPALVRANGA-------------------SLGQELLGSY--------QM-----------

>Mbr5

---------------------------------------------------------------------------------------------------------------------------------------------------------------------------------------------MALFNGKLLDAYFIRPFYKMMLDLPITLEDIEAVDIEYYNSLRWLLDNDPEP--LCLTFQVDHDEYGEMVHTDLKPGGADIDVTKANRQEYVDLVIKHRFVNRIQEQMTAFMRGLTMIIPQEDLSVFDPSELELLIGGISAIDVNDWRTHTKFLDGYTTSSPPVKWFWEAVHSFTKEQRARLLQFVTGTSRVPIGGFAELYGSNGAQKFCIARRGVPPELPRSHTCFNRIDLPPYESYAILRKKLLLAVENTQGYD

>Mbr4

--------------------------------------------------------------------------------------------------------------------------------------------------------------------------------------MVGRVLGLAVWHGHYVDGGFVMPLYKHLLGKPVTLDDMAHVDEMFYNSLVWMLENDITGI-IENNFVDEYDAFGVQETIELKPGGSDLPVTESNKNEYVQLIVRHRLNFGIEEQVKALKQGFNDVVPHAYVSMFDEAELELIICGLGEIDVGDWSSNTEYR-HCEPTDEQIAWFWDVLRSFDTELRARVLQFVTGTSRVPVTGFRDLRGAQGPKLFTIETNAVRNGLPRAHTCFNRIDLPPYDSQDQMRERLLQAVENAIGFG

>Mbr1

PEGWEIRRDAR-GRVYYVDHNSRSTTWQRPLPPGWEQRQTPQGRAYYHPARHTQWEDPRLPPRYHRFKHKVWCLHKHYCPQ----VQGQFKMPIRRSSLFQDSFDCIMSEHPDETGFRRLFITFQGEQGLDYGGVAREWFFLISHEMLDPMYCLFEYATANNYQLQINPNSHVNPEHLQYFRFVGRVVALAIYHKKFIDNGFTLPFYKRLLNKKLVLQDLETVDPDFYKNLYWLLNNEIDDLELGLVFTADSNEFGAVKEVELKAGGKDIEVTDANKQEYVELMANFRLKRGVEEQTEAFLMGFHEILPHQAIEFFDEREMELLLIGMAEFDVDAWEKHTIYR-NYRKKDRQVAWFWEVVREFTQEQRARLLQFVTGSCRLPVGGFAELQGSNGPQPFCIERYNDHGALPRSHTCFNRLDLPPYKTKEAMKQKLTMAIEETEGFG

>Mbr2

-----------------------------------------------------------------------------------------------------------MRLQRKDLR-RRLMVQFHGEGGLDYGGLAREWFYLLGIEVFDPRLGMFSYCNEQDYLLQINPSSSADQDHTLFFHFTGRLIGLAILHRHFLDVTFVSSFYKQILGQSITLADLQDSDPDVHRSLIWILENDVSEV-PDLTFSTDEDELGDIRTHELVPGGANKAVTEENKFEFAKLMVEWKLIKSSSRQMCALLTGLNEVIPIENFRTFTVKELRFLISGSHEYDLEDWKRNTEYK-GYESNDQIIEWLWEIVEAWDHDNQARFLQFCTGSSRVPIEGFQALQGSDGPRRFCIQKLEDLTRLPSAHTCFNRLDLPEFPVRRMLEERLALALKNAQGFT

>Hro5

---------------------MMLSVFEEE-----------------DSSENNENYDNNFGPA---LEVKLAEFYNKLEAKGYSKGPVKYRLIVRRDHILEDAFVKVMSLTKKELIKSKLAIAFKGEDGLDYGGPSREFFFLLSRELFNPYYGLFAYSANDTYTVQVSPSSNQIENNLQWFRFAGRMIALALVHQYLLDVFFTRVFYKQLLHIKPHLNDLESLDMEFYRSLTWIQDEKQDDLEMALTFSI------RVIERDLKQNGRHIRVTERNKKEYIERMVDWRIKRGTQKQMKVLVQGFNEILDPRLVSKFDANELELVIAGSVDIDIEDWHDNTVYRSGYSESHQVIMWFWKVVSNYNNDQRLRLLQFVTGTSSIPVEGFAALRGSTGPKKFCIEKWGKVTDLPRAHTCFNRLDLPPYTSFEILLEKLSIAVQETATFG

>Lgi2

ATGWEARVDSH-GRIFYIDHINRQTTWQRPVPRRWEMKLDRTGKTFFHKMKITTFIDPRVPPVYKRFHAKLRNFYRKLESKGYGQGPGKLKLTVRRDH---EGFTV----------KDPFYQLVLGQTSLDYGGPSREFFFLLSRELFNPYYGLFEYSANDTYTVQISPMSAFVENAHEWFRFAGRVLGLALVHQYLLDAFFTRPFYKFILRVPWSLEDVETIDAEFYQSLLWIKDNDITESDLDLTFSVSEEVFGQVTERELKQGGSKLPVTERNKKEYTEKMAKWRLERGVSEQIESLIRGFHEVVEARIVSIFDARELELVIAGTVEIDIADWRKNTEYRSGYHDQHPVIQWFWTAISKFDNERRLRLLQFVTGTSSIPYEGFSALRGSNGPRKFCIEKWGKTTSLPRAHTCFNRLDLPPYSSCDMLFEKLVMAVEETSTFG

>Hro3

NAYNNSKIIIIITSMILMTMIPFWILFLMSLPEGWELNIDKRGRVYYRRLRITQWEDPRLPPRYERFKWKM-NQFRLNCVNH--ATPGFVKIILSREHLFEDSFIQ-ISRIPASDLKRKLFISYQDEDALDYGGVARDWFMRLSHEVCNPIFSLFEYVNNHNYRLQINPASFVNPDYLKYFTFIGRFVGMALFHGKFIDSGFSLSFYKSILNRKFCLKDVESHDVHMYNSLLWLKTHNIDDADLEIYFNDDIDVLGQLEHYELKEGGDNIKVTEQSKLEYIELKINSLFTRGVDEQMKAFKSGFHEIIPKQWLILLDERELELMLCGMQEINVDDWQKNTIYR-HYTPTSKTIIHFWKFVHNLDNERRTRLLQFVTGTCRLPLGGFSHLIGNNGPQKFCIEKMGKESWLPRSHTCFNRIDLPPYKTYDQLAEKLTYAIEETEGFG

>Tad1

PSGWERRHDQY-NRPYYVDHNTRTTTWHRPLPPGWERRIDHNGRVYFHNTKTTQWEDPRLPPRYG-FDWRL-AGFRQMCQDS--ALPGHTKIAISRNTIFEDSYNAIISLKPYDLR-KRLYIMFKGEDGLDYGGLAREWFFQLSHEMLNPMYCLFEYANQNNYSLQINAASSVNPDHLKYFKFVGRVIAMALYHGKFIDNGFTLPFYKRLLNRGVSINDLEQVDPEFYNSLNWIKDNNIDECDMEMFFTADMEIFGEIKTYELKTGGSDIKVTDENKEEYINLMSHWRFTRGVEDQTKAFMEGFYEVVPLRWLEFFNEKELEMMLCGMQEIDVDDWQQNTVYK-HYTKNSKQVMWFWQFVRDRKNEQRIRLLQFITGTCRVPIGGFSHLMGSNGPQKFCIEKVGKESWLPRSHTCFNRLDLPPYKSYDQLVEKLNFAIEETEGFG

>Hro2

PAGWERRLDQR-GRVYYVDHNTKTTTWQSPLPEGFEKRIDSNNRVYFHRNRTTQWQDPRLPPRYERFRWKL-GQFRYLCQTN--STSGYVKINVNRDTLFEDSFVQIMKQPPYELR-KRLFITYKGEEGLDYGGVARDWFFNVSHEVLNPMYCLFQYANDKNYCLQINPASFINPDHLQYFKFMGIFIAMALFHGRFIDSGFTMPFYKKLLNKKLFLADIETIDPELHSSMVWVKENNIDECNLDLCFASDFELLGKIVQHELKPGGLDIVVDEQNKEEYLNLMTQWVFNRGVEEQMKAFLVGFNEVVPLQWLQYFDEREIELMLCGMQEIDVDDWENNTIYR-HYHKSSKQIVWLWKFIRELDNEKRTRFLQFVTGTCRLPVGGFRELIGNNGPQLFCVEKVGKESWLPRSHTCFNRLDLPPYKSYEQLVEKLNFAIENTEGFG

>Hro1

PPGWEMRLDEH-GRPYYVDHNTRTTTWQKPLPEGWEKRVDPNNRAYFHRTKTTQWEDPRLPPRYERFRWKL-SQFRILCQSN--NPQGHVKINVTRENLFEDSFQQIMRLQPMDLR-RRLFIVIKGEEGLDYGGIAREWFFQVSHDVLNPMYCLFQYANNNNYGLQINPASYINPDHLMYFKFIGRFIAMALYHGKFIDSGFTMPFYKRMLNKSLTINDIETVDPEFFNSLVWVRDNDIDECDIELYFVSDYEILGKIEEHELVPGGSKIKVTDANKKEYIEAMTKWLFSRGVVEQMKAFLDGFNEIVPLTWLQYFDERELELMLCGMQEIDVNDWELHSIYR-HYNKSSRQVQWFWKFVRELDNEKRMRLLQFVTGTCRLPVGGFAELMGRNGPQQFCIEKVGKDSWLPRSHTCFNRLDLPPYKSYEQLVEKLNYAIEETEGFG

>Lgi1

PQGWERRVDQR-GRVYYVDHNTRTTTWQRPLPEGWEKRVDANARVYYHKNRTTQWEDPRLPPRYERFVWKL-GQFRYLCHSN--SLPGHVKITVARENMFEDSFAQIMRLKPFDLR-RRLYMIFKGEEGLDYGGLAREWFFHLSHEVLNPMYCLFEYASNSNYCLQINPASSVNPDHLHYFRFIGRFIAMALYHGKFIDSGFTLPFYKRMLSKKLSIKDLESVDQEFYSSLLWIKENSIEECGLELYFCADFEILGKIEQHELKPGGADIKVTDENKEEYINLMTNWRFTRGVEEQTKAFLEGFNEVVPLQWLHYFDERELELMLCGMQEIDVDDWERNTKYR-HYQRNSKQIGWFWKFVREIDNEKRTRLLQFVTGTCRLPVGGFAELMGSNGPQCFCIEKVGKETWLPRSHTCFNRLDLPPFRSYEQLVEKLTYAIEETEGFG

>Spu2

---WERRKDPQ-GRIYYVDHNTRTTTWQRPMPSGWEKRTDPHQRVYFHLNRTTQWEDPRLPPRYERFRWKL-GQFRYLCQIN--ALPSHVKISVTRNTLFEDSFHQIMRLQAFDLR-RRLYIIFRGEEGLDYGGVAREWFFMLSHEVLNPMYCLFEYANKNNYCLQINPASSVNPDHLQYFRFVGRFIAMALYHGKFIYSGFTMPFYKRMLNKPLSLRDLESIDPEFYNSLVWIKDNDIDELDMEMAFVADFEILGKVETVDLKEGGKDIDVSEENKEEYIHLMTQFRFNRGIEEQTKAFLEGMNEVVPLQWLQYFDERELELMLCGMQEFDVDDWYRCTIYR-HYTRESKQVQWFWRAVREMDNEKRARLLQFVTGTCRLPVGGFTELMGSNGPQKFCIEKVGKETWLPRSHTCFNRLDLPPYKSYEQLTEKLTFAIEETEGFG

>Spu3

-----------------------------------------------------------IP--YERFRWKL-GQFRYLCQIN--ALPSHVKISVTRNTLFEDSFHQIMRLQAFDLR-RRLYIIFRGEEGLDYGGVAREWFFMLSHEVLNPMYCLFEYANKNNYCLQINPASSVNPDHLQYFRFVGRFIAMALYHGKFIYSGFTMPFYKRMLNKPLSLRDLESIDPEFYNSLVWIKDNDIDELDMEMAFVADFEILGKVETVDLKEGGKDIDVSEENKEEYIHLMTQFRFNRGIEEQTKAFLEGMNEVVPLQWLQYFDERELELMLCGMQEFDVDDWYRCTIYR-HYTRESKQVQWFWRAVREMDNEKRARLLQFVTGTCRLPVGGFTELMGSNGPQKFCIEKVGKETWLPRSHTCFNRLDLPPYKSYEQLTEKLTFAIEETEGFG

>Lgi3

-----------------------------------------------------------------------------------------------------------MNIKDTEHLKTRLWIEFDGETGLDYGGVAREWFYLLSKEMFNPYYGLFEYSATDNYTLQINPLSWMANEHLSFFEFIGRVAGMAIYHGKLLDGFFIRPFYKMMLRKPITLRDMESVDSEYYNSLIWIQDNDPED--LELHFSVEEDQFGEIQEFELKANGANIRVTNENKLEYIKEVIKYRFVSRVESQMKSLMKGFNTIIPQNLIQIFDENELELLMCGLQDIDVNDWKSHTLYKGEYNPNHPTVIHFWRVVYSFHNEMRARLLQFVTGTSRVPMNGFAELYGSNGPQLFTIEKWGKTAQLPRAHTCFNRIDLPPYDSYQEVRSKLVLALENTQGFY

>Spu4

------------------------------LPEGWQIQKAPNGKKFFHNTRTTSWEDPRLPPRYSRYK-RKYDFLKMHLKKPS-NIPNRFELKVHRNNLLEDSYRGISSIRSADLLKARLWIEFTGETGLDYGGVAREWFFLLSKEMFNPYYGLYEYSAMDNYTLQINPDSGICNEHISYFKFIGRVAGMAVFHGKLLDAFFIRPFYKMMAGKPITLRDMESV------------------------------------------------------------------------------------------------------------------------------------------------------------------------------------------------------------------------------

>Tad2

TPITVSPTNTTNSNGVSPQQVQQLTQYCYPLPHGWEMMFTEQGRPFFHNTKTTSWNDPRLPPRYSRYK-AKYDTFRQWMKVPD-NLPNKFDIRVKRSHILEDSFRSISAVKKPDLLKTRLWIEFDQESGLDYGGLAREWFYLLSHEIFNPYYGLFEYSANDNYTLQINPNSGLCNEHLAYFKFAGRVAGMAVFHGKLLDAFFIAPFYKMMLGKPITLDDMEAVDTEYYNSLQYIMENDPSE--LDLLFSVDEETLGKVNQIDLKPNGKDIPVTEKNKKEYIDLVIKWRFASRIKSQMDKFLEGFRELVSLERLRIFDEREIELLMCGMGDIDVHDWRRNTNYKNGYGDQHLVIQWFWQVVYALEKESRLRLLQFVTGTSRVPMNGFSELYGINGPQRFTIERWGKFDQLPRAHTCFNRIDLPEYKSYQDLHDKLIMAIECTQGYE

>Tad3

NSNLFRGRSSANPNKHYSSPSNQHAPYNGNLPPGWEMRHNPNGRIYFHNTRRTQAIDPR-------------QKWRNLKSDLSTQPQGFTKIEVTRSDVFEASYNQIMKMRPKDLK-KRLTVKFKGEEGLDYGGVAREWLHLLSHEMLNPSYGLFTFSDDDMCCLQINQDSSINTNHLSYFHFVGRVMGMGVFHGHHIDGTFPTPFYKQLLNKACTIEDLESVDPGFYRSLCWLLNNDITDD-LEQNFCVEHQSFGEIVEYDLKPNGSAIRVTNDNKYEYAELLVNWKLTHGIDEQLQALKKGFYEIVPTYLLKNFHEKELELIIGGLKKIDIQDWKANTRLK-HCTPSTDVVKWFWQIVDSYCEEERMRLLQFVTGSSRVPLQGFEALQGATGSRLFTINVVDINTDLPKAHTCFNRLDFPPYENYDKMLQKLTCAIEETCGFA

>Hro4

FGVWEERRFST-GRVLYLNHITRSTQWERPLPDGYEQRTTAQGQVYYTQSGVSSWHDPRMPPRKKRLVQKL-KMLRQELHALQ-PQASHCRIEVSREEIFEESYRAIMKMRSKELK-KRLMVKFRGEEGLDYGGIAREWLYLLSHEMLNPYYGLFQYSRDDIYTLQINPDSGINPEHLSYFHFVGRIIGMAIFHGHYLDGGFTLPFYKQLLGKQISLDDLEAVDPDLYRSLVWILENDITGV-IDNVFCVDHEAFGERKEHELKTGGRDVPVTQENKKEYVKLYVNWRFMRGVEAQFAALQKGLNELVPQQLLKPFDEREIELMIGGLGRIDVEDWRKNTRLK-HCTSSSDVVVWFWRAVEEYDEERRARLLQFVTGSSRVPLQGFKALQGRGGGRLFTIHQIEASTDLPKAHTCFNRIDIPAYESYEKFFDKLTCAVEETCGFT

>Ppi

PAGWAKGKDQNSGRTFYIDHNSRTTSWEKPLPDGWEMRYK-NGRPFFHRTKQTTWHDPRLPPRYSRYK-VKYENFRANLPQPS-SNTAKCEVRVSRQDILANSFDSIMSKTSSDLIK-RLWVVFDGEPGLDYGGVAREWFYSLF-EMFNPYYGLWEYSAIDNYTIQINPNSGMCN------------------------------------------------------------------------------------------------------------------------------------------------------------------------------------------------------------------------------------------------------------------------------

>Aqu

PAGWERRTDPR-GRVYFVDHNTRTTTWQKPLPDNWEKRLLPNGRVYFHKSKTTQWEDPRLPPRYERFKWKV-GHFRTLCSNN--ALPQHIKIHCSRDTIFEDSFQQVMRFQSQDLR-RRLYIMFRGEEGLDYGGVAREWFFHLSHQMLNPMYCLFEYVGDKNYQLQINPASGVNPEHLQYFRFIGRIVAMALYHGKFIDNGFSMAFYKQLLHKKLSLKDLEHIDPEFYNSLVWIRDNNIEECGLEMYFAQDYDVLGVVNSHELVEGGADKLVTDENKHEYIDLVLQWRFSRGVKDQTKSFMEGFEEVVPIEWISIFDERELELMLCGMQEIDVNEWERNTIYR-NYTRGTKQIQWFWQTVREMDNEKRVRLLQFVTGTCRLPVGGFVELMGSNGPQKFCIDRVGKETWLPRSHTCFNRLDLPPYKSYDQLKEKLLFAIEETEGFG

>Nv1

SDGTTSPMEETLSV---SPSILSAETWTAPTPATRQLSLIETDRIHVRRGSEGTISQPRVC-RRTSPRGSLSSERPERSRRRRTTEPRDSSLPCTASTPVSHDQSP-STLSSIQQDSVVLATRLVSTSETSNTESPRSSVVIASTQVLSPGHDSAEQIMDELETVVAVPVEVSNTEAPSEAHDRTVVAGFPLSPGPSTSTNVTPLPSPRLPQRPNYEGDREQLEQVLTRSSRSSRPTPLPTPGHGAQYSSGQEQSNSSTVSGSDTGAIPRQRTLDSRGNITQILRSYIRDTQTQPQLRDDVISYEDRLPSNTHPLVDHRDNEPAVGNEH-IRIRGRRCHEDNSRRYERAAQ---------RQLNLDNTARVEQTSQGQLGLRQSGWRERRGSEEPEGSTRRRRHHRDDQQPQPVHVQIRDQPPEQTEQEREEPPLPPSE------

>Nv2

---------------------------------------------------------------------------------------MAASNAARSAAL--------LNSPAVKFITRPDFVDF-----IKNSGVAGQYYFQ--SQMLKLIVSKIRSDPA----------------------------QFERYRHQVELVRFLNQFADPQMELFPGWDKVDQNGRLYF-----IDHNRTTTF-------IDPRL--PVNTTDRRP--RPGPETEPPRRSRAPPPRPPPPSSR-GHSRSGSTAGSSQALNQQVIGFLSQPNVDEIITKKQPFNRNSALKATVRRRALERLSNNVDFVL--LLSLFEDEIMSFVSSCSGPST-PSTSQPSSP-ARAPNRVPA--MSDSIALPGGAAGGHSVSASPTPYRRDFEAKVSFHRQYHKGYG

>Nv3

TDTQADTHADTQADTHADTHADTLQTRMQTLPPGWAVQRAPNGRLFFHNSRTTTWHDPRLPPRYSRYK-RKCDYFRSKLKRPS-NVPNKYEIHIRRRNLMEDSYRAVLSIVKPEILKSRLWIVFDGETGLDYGGLQREWFYLLSKEVFNPYYGLFEYSASDNYTLQINPNSGLCNEHLSYFKFIGRVAGMAVYHGKLLDAFFIRPFYKMMLGRPITLIDMESVDSEYYNSLNWILENDPED--LDLHFCVDEELFGILSVKDLKPNGSQTNVTNENKREYINLVIKWRFVSRVEDQMRAFMEGFCDLIPHNLIQIFDERELELLMCGLGEIDTVDWRKNSNYRGEYHDKHIVIQWFWKAVNSFDIETRARLLQFVTGTSRVPMNGFSELYGSNGPQRFTIEPWGTPHSLPRAHTCFNRLDLPRYRSYYELRERLRIAIENTQGFE

>Nv4

-----------------------------------------------------------------------------------------------------------MKETARSLQRNRLEIQFAGEEGLDYGGPAREFFFLISRQIFNPYYGLFEYSANDTYTVQVSPVSLYVDNSHEWFRFCGRIVGLVIIHQHLLDAFFTRTFYKALLRSLCDLSDVEALDALFHQSMTWVIENDIEDV-LDLTFSVSEEIFGQVTERELIPGGKDIAVTEQNKGDYVAEMVKWRVERGVSEQMESIVRGFNEVIDPALVNIFDARELELVISGTADIDIKDWRRNTEYRSGYHDNHKVVKWFWKAVSSFDNEQRLRLLQFVTGTSSIPYEGFAALRGSTGPRKFSIERWGDYTKLPRAHTCFNRLDMPIYRSYDELLEKLTYAVEETGSFA

>Nv5

PHGWERRTDSR-GRTYYVDHNTRTTTWQRPLPAGWEKRVETNGRVYFHNTRTTQWEDPRLPPRAERFRWKV-GQFRHLCQAN--TLPSHIKITVSRTNLFEESFQQVMRYQPHDLR-RRLYITFKGEEGLDYGGVAREWFFLLSHEVLNPMYCLFEYANKNNYSLQINAASSVNPDHLMYFKFIGRFIAMALYHGKFIDRGFTLPFYKRMLNKKLLMKDLETIDPEFYNSLVWVKENNIEECGLEMFFTVDMELLGKVTSHDLKPGGTDIAVIEENKEEYISLMTEWRLNRGIEEQTRAFLEGMHEVLPLYWIQYFDERELELMLCGMQEIDVEDWQQNTVYR-HYTRNSKQVMWFWQAVKAYDNEKRIRLLQFVTGTCRLPVGGFTELMGSNGPQKFCIEKVGKETWLPRSHTWWVHIT-------------------------

>Aae1

ERSWPPKHQRKQQLQQGFSFDIIDTDEQSYRLGRLDKNANPDETV--VVEPSASNIKPP--PSLRTTKHTVHVIFPIQSLSPCASPPGSSTRSGSPLEHSPDRSSP-IIMHPKPIRPQPRPLTRVGCTRNDLICPPTPTHHARRLRVLSDNFGPPDLRPRNVF----SPETITSPE-MRYVAVTGNRVQLRVGEGREDEIETDSPVRHLTSTRPPAWEAMDSHGRIFYRTTSWAHSGNGSEQHLDRRYQYDRRDGGGFEPINPRQGASAIAVANAAQIE------NLQFDRAAPALLMICRPDFYSMLNTDAIQIYNRNSLKHMVSRVRR-DPTCFSRYQHNRNCFASPDKDLPSGWE--TKMDQSGKQFFISFMDDCPRAQLIAAAAVAPPIPPRPPALPRIGSPEIVAYNDKVVAFLRQPNIACSRNLRDKISIRVEGTSALD

>Aae2

ENGRHSPEDNNASDRVNNNSNDSSSLLNDTLPPGWTMQYAANGRLFFHINKKTSWVDPRLPPRYSRYK-QKYEYLKSQLKKPA-NVPNKIDIKVRRASILEDSYRIINSVTKVELLKTKLWVEFESEAGLDYGGLAREWFYLLSKEMFNPYYGLFEYSAMDNYTLQINPYSGLCNEHLNYFKFIGRIAGMAVYHGKLLDAFFIRPFYKMMLQKQIDLKDMESVDTEYYNSLLYIKENDPSE--LMLTFSVDEESFGTTSQRDLKPNGANIEVTNDNKDEYIKLVIDWRFVARVKSQMHAFLEGFGSLVPLHLLKIFDENELELLMCGIQSIDVSDWKKNTLYKGDYYANHVVVQWFWRAVLSFNNEMRSRLLQFVTGTSRVPMNGFKELYGSNGPQMFTIEKWGTTDNFPRAHTCFNRLDLPPYESYSHLKDKLVKAIEGSQGFA

>Aae3

VDGRKEEAPPSPQEVLQFGEDDHEVIYNRVLPAGWSMQLAPNGRVFFHNEKKTSWVDPRLPPRYSRYK-RKYEYLKGQLRKPA-NVPNKIEIKVRRVSILEDSYRIINSITKTELLKTKLWIEFEGEAGLDYGGLAREWFYLLSKEMFNPYYGLFEYSAMDNYTLQINPFSGLCNEHLHYFKFIGRVAGMAVYHGKLLDAFFIRPFYKMMLQKPIDLKDMESVDMEYYNSLLWIKENDPSE--LMLTFCVDEETFGYTSQRELKPNGADIEVTNENKDEYIKLVIEWRFVARVKDQMSAFLDGFGQIVPLNLLKIFDENELELLMCGIQSIDVKDWKRNTLYKGDYYANHVIIQWFWKAVLSFSNEMRSRLLQFVTGTSRVPMNGFKELYGSNGPQMFTIEKWGTPENYPRAHTCFNRLDLPPYESYLALKDRLIKAIEGSQGFA

>Aae4

PAGWEMRRDAR-GRVYYVDHNTRTTTWQRPLPDGWEKRVQPDNRVYFHKNRTTQWEDPRLPPRYERFRWKL-SQFRYLCQSN--ALASHIKITLTRQTLFEDSYHQIMRLPAYELR-RRLYIIFRGEEGLDYGGVSREWFFLLSHEVLNPMYCLFEYANKNNYSLQINPASYVNPDHLQYFKFIGRFIAMALYHGRFIYSGFTMPFYKRMLNKKLTTKDIESIDPEFYNSLIWVRDNNIDECGLELWFSVDFEVLGQIIHHELKEEGDKEKVTEENKEEYISLMTEWRMTRGIEEQTKTFLDGFNEVVPLEWLKYFDERELELMLCGMQEIDVDDWQRNSIYR-HYNRNSKQVVWFWQFVRETDNEKRARLLQFVTGTCRVPVGGFAELMGSNGPQRFCIEKVGKDTWLPRSHTCFNRLDLPPYKSYDQLVEKLNYAIEETEGFG

>Aae5

-----------------LDDNNSLAATVLD-----------------LGMVHCLFENEI--PQFRRFEAKLRTFYRKLESKGFGQGPHKLKLHIRRSHLLEDAFRRIMSANKKDLQRGRLAVLWDTEEGLDYGGPSREFFFLLSRELFNPYYGLFEYSANDTYTVQVSPLSAFVDNSHDWFRFSGRVLGLALVHQYLLDAFFTRPFYKALLRLPVALSDLESLDNEFHQSLQWIRDNDIGSGTLGLTFCVTEELLGRVVERELKPGGKNIPVTEKNKREYLERMVKWRLERGVQEQTESLVRGFYEVVDPRLVSVFDARELELVIAGTAEIDLNDWRINTEYRSGYHDGHQVIVWFWHVIEKFSNEQRLRLLQFVTGTSSIPYEGFAALRGSTGPRRFCIEKWGKPNALPRAHTCFNRLDLPPYPTPDILYEKLLLAVEETNTFG

>Gga

------------------MAAAQGPVSCEP---GTYLSSVPIGSM--SVGSLVEKQDLSAPLRRERHQASA-ACYKRDYESDRERSPERCSREHHRGADFSKSSLP--------------------ERGFDKCRIRPSAFKAVTGKGLVSMQGLSSSKGQ----------------KLSK--------SNGSLHTLLSQSTTAAPQHGPLRTHLLHAISLDEASDSSHNSISFPSYGRLKP--AQSQFS---ASMGHINHI----GGSLDRVSRSPRDPLAPEKAPLSCK---SMATLSRLQSPGEPPPPYEFTYSLEDAVKQLEDRLKETELRQLKRSSETEDPFAQAFEDKQRLWDELEDLKQMYMARL-QQVTGQRALQLQLYKAQQEKKRLEELSMQQCEETKLQSQGERVSPKLEETKWEVSPLLSLSVTLQAVPGWGSG

>Gga1

PPNWEARIDSH-GRIFYVDHVNRTTTWQRPLPRGWEMKHDHQGKAFFHNSRTTTFIDPRVPPRYKRFEAKLRNFYRKLETKGYGQGPGKLKLIIRRDHLLEDAFNQIMGYSRKDLQRNKLYVTFVGEEGLDYSGPSREFFFLVSRELFNPYYGLFEYSANDTYTVQISPMSAFVDNHHEWFRFSGRILGLALIHQYLLDAFFTRPFYKALLRILCDLSDLEYLDEEFHQSLQWMKDNDIHDI-LDLTFTVNEEVFGQITERELKPGGANIPVTEKNKKEYIERMVKWRIERGVVQQTESLVRGFYEVVDARLVSVFDARELELVIAGTAEIDLSDWRNNTEYRGGYHDNHIVIRWFWAAVERFNNEQRLRLLQFVTGTSSIPYEGFASLRGSNGPRRFCVEKWGKITALPRAHTCFNRLDLPPYPSFSMLYEKLLTAVEETSTFG

>Gga2

PPSWERRVDNM-GRIYYVDHFTRTTTWQRPLPPGWEKRTDSNGRVYFHNTRITQWEDPRLPPRYVRFKAKV-HYFRFWCQQL--AMPQHIKITVSRKTLFEDSFQQIMSFSPQDLR-RRLWVIFPGEEGLDYGGVAREWFFLLSHEVLNPMYCLFEYAGKDNYCLQINPASYINPDHLKYFRFIGRFIAMALFHGKFIDTGFSLPFYKRILNKPVGLKDLESVDPEFYNSLIWVKENDIEECGLEMFFSVDKEILGEIKSHDLKPNGSNILVTEENKEEYIRLVAEWRLSRGVEEQTQAFFEGFNEILPQQYLQYFDAKELEVLLCGMQEIDLNDWQRHTIYR-HYTRTSRQILWFWQFVKEIDNEKRMRLLQFVTGTCRLPVGGFADLMGSNGPQKFCIEKVGKENWLPRSHTCFNRLDLPPYKNYEQLKEKLLFAIEETEGFG

>Gga3

PPGWEERQDEK-GRSYYIDHNSRTTTWIKPLPKGWEVRHAPNGRPFFHNTKTTTWEDPRLPPRYSRYK-RKYEFFRKKLKKQS-DIPNRFEMKIHRTTILEDSYRRIIAVKRADFLKARLWIEFDGEKGLDYGGVAREWFFLLSKEMFNPYYGLFEYSATDNYTLQINPNSGLCNEHLSYFKFIGRVAGMAVYHGKLLDAFFIRPFYKMMLQKPITLHDMESVDSEYYNSLRWILENDPAE--LDLRFIVDEELFGQTHQHELKSGGSEIVVTNKNKRDYIHLVIQWRFVSRVQKQMAAFKEGFFELIPQDLIKIFDENELELLMCGLGDVDVADWKLHTKYKNGYSVNHQVIQWFWKAVLMMDSEKRIRLLQFVTGTSRVPMNGFAELYGSNGPQLFTVEQWGTPEKLPRAHTCFNRLDLPPYDSFEDLWDKLLLAIENTQGFD

>Gga4

PPNWEARIDSH-GRVFYVDHVNRTTTWQRPLPRGWEIKTDQQGKSFFHNSRATTFIDPRLPPRYRRFEAKLRNFYRKLEAKGYGQGPGKIKLIIRRDHLLEGTFNQVMAYSRKELQRNKLYITFVGEEGLDYSGPSREFFFLLSQELFNPYYGLFEYSANDTYTVQISPMSAFVENHLEWFRFSGRILGLALIHQYLLDAFFTRPFYKALLRLPCDLSDLEYLDEEFHQSLQWMKDNNITDI-LDLTFTVNEEVFGQVTERELKSGGANTAVTEKNKKEYIERMVKWRVERGVVQQTEALVRGFYEVVDSRLVSVFDARELELVIAGTAEIDLNDWRNNTEYRGGYHDGHIVIRWFWAAVERFNNEQRLRLLQFVTGTSSVPYEGFAALRGSNGLRRFCIEKWGKITSLPRAHTCFNRLDLPPYPSYSMLYEKLLTAVEETSTFG

>Gga5

PPGWEKRVDPR-GRYYYVDHNTRTTTWQRPLPPGWEKRQD-NARVYYHNTRTTQWEDPRLPPRYDRFRWKY-HQFRFLCHSN--ALPSHVKISVSRQTLFEDSFQQIMNMKPYDLR-RRLYIIMRGEEGLDYGGIAREWFFLLSHEVLNPMYCLFEYAGKNNYCLQINPASSINPDHLTYFRFIGRFIAMALYHGKFIDTGFTLPFYKRMLNKRPTLKDLESIDPEFYNSIVWTKENSLEECGLELYFIQDMEILGKVTTHELKEGGESIRVTEENKEEYIMLLTDWRFTRGVEEQTKAFLDGFNEVVPLEWLRYFDEKELELMLCGMQEIDMNDWQKNTIYR-HYTKNSKQIQWFWQVVKEMDNEKRIRLLQFVTGTCRLPVGGFAELIGVNGPQKFCIDKVGKETWLPRSHTCFNRLDLPPYKSYEQLKEKLLYAIEETEGFG

>Gga6

PPGWERRVDDR-GRVYYVDHNTRTTTWQRPLPPGWERRVDSNDRVYFHNTKTTQWEDPRLPPRYERFRWKL-AHFRYLCQSN--ALPSHVKINVSRQTLFEDSFQQIMALKPYDLR-RRLYVIFRGEEGLDYGGLAREWFFLLSHEVLNPMYCLFEYAGKSNYCLQINPASTINPDHLSYFCFIGRFIAMALFHGKFIDTGFSLPFYKRMLSKKLTIKDLESIDTEFYNSLIWIRDNNIEECNLEMYFCVDMELLGKVTSHELKSGGSNILVTEENKEEYIGLMAEWRFSRGVREQTKAFLDGFNEVVPLQWLHYFDEKELEVMLCGMQEVDLADWQRNTVYR-HYTRNSKQIIWFWQFVKETDNEVRMRLLQFVTGTCRLPLGGFAELMGSNGPQKFCIEKVGKETWLPRSHTCFNRLDLPPYKSYEQLKEKLLFAIEETEGFG

>Gga7

PSGWEERKDAK-GRTYYVNHNNRTTTWTRPLPPGWEMRIAPNGRPFFHNTKTTTWEDPRLPPRYSRFK-QKYDYFRKKLKKPA-DIPNRFEMKLHRNNIFEESYRRIMSVKRPDVLKARLWIEFESEKGLDYGGVAREWFFLLSKEMFNPYYGLFEYSATDNYTLQINPNSGLCNEHLSYFTFIGRVAGLAVYHGKLLDGFFIRPFYKMMLGKPITLKDMESVDSEYYNSLKWILENDPTE--LDLMFCIDEENFGQTYQVDLKPNGSEIMVTNENKREYIDLVIQWRFVNRVQKQMNAFLEGFTELLPIDLIKIFDENELELLMCGLGDVDVNDWRQHTIYKNGYCPNHPVIQWFWKAVLLMDAEKRIRLLQFVTGTSRVPMNGFAELYGSNGPQLFTIEQWGTPDKLPRAHTCFNRLDLPLYESFDDLREKLLMAVENAQGFE

>Gga8

-------------------------------------------------------------------------------------------------RLLFEVFDE-NRLSRDDFR-GQVDLPLSH---LPTEDPTMERPYTFKDFLLRPRS------------------------HKSH----------------------VKGFLRLKMG---------------Y-----MPKN----------------------------GGQE----EENSNQRNESEHEWDVTDSSDSASQC-----QEVLPPP-----------PLPPGWEE-KVDNLGR-TYYV---NHNNRTTQWHRSLIDVGSDSENNRQINYEAAHRRF---------------------------RSRRHIS-EDLEPEPMETGDILEPWEAISEESTSGDS

>Dre

------------------------------------------------------MSDQR--------------------------------------------YQQ-------------LTNEEESAEG-------------------------FQQTAD-----APPPYSSLGAS------------NAAFFEYKEDEVYPKPPSYNVATS-LPSYDEAERSKAEATVPL--VTDRDEDF--------IARDSFE--DTDQLRVGNDGIFML----TFFMAFLFNWI----------GFFLSF--CLTTSAAGRYGA------ISGFG-LSLVKWVLIVRFSTYFPGYFDGQYWLWVFL----------VVGFLL-----FFRGFVNYSR---------DRADHSMSLPRTR---------------------VLFIY------

>Dre1

PPNWEARIDSH-GRVFYVDHINRTTTWQRPLPRGWEIKTDPQGKSFFHNSRATTFIDPRVPPRYRRFEAKLRNFYRKLEAKGYGQGPGKIKLIVRRDHLLEGTFNQVMAYSRKELQRNKLYITFVGEEGLDYSGPSREFFFLLSQELFNPYYGLFEYSANDTYTVQISPMSAFVENHLEWFRFSGRILGLALIHQYLLDAFFTRPFYKALLRLPTDLSDLEYLDEEFHQSLQWMKENDITDV-LDLTFTVNEEVFGQVTERELKSGGTNVQVTEKNKKEYIERMVKWRVERGVVQQTQALVRGFYEVVDSRLVSVFDARELELVIAGTAEIDLNDWRNNTEYRGGYHDGHIVIRWFWGAVERFNNEQRLRLLQFVTGTSSVPYEGFTALRGSNGLRRFCIEKWGKITSLPRAHTCFNRLDLPPYPSYTMLYEKLLIAVEETSTFG

>Dre2

PPNWEARIDSH-GRIFFVDHVNRTTTWQRPLPRGWEMKHDHTGKAFFHNCRATTFIDPRVPASYKRFEAKLRNFYRKMETKGYGQGPGKVKLIIRRDHLLEDAFNQIMCYSRKDLQRSRLYVSFVGEEGLDYSGPSREFFFLVSRELFNPYYGLFEYSANDTYTVQISPMSAFVDNHHEWFRFSGRILGLALIHQYLLDAFFTRPFYKGLLRIPCDLSDLEFLDEEFHQSLQWMKDNDIEDM-LDLTFTVNEEVFGQITERELKPGGSGIAVSDKNKKEYIERMVKWRIERGVAQQTESLVRGFYEVVDVRLVSVFDARELELVIAGTAEIDLSDWRNNTEYRGGYHDNHIVIRWFWAAVERFNNEQRLRLLQFVTGTSSIPYEGFASLRGSNGPRRFCVEKWGKVTSLPRAHTCFNRLDLPPYPSFSMLYEKMVTAVEETSTFG

>Dre3

PPNWEARIDSH-GRVFYVDHINRTTTWQRPLPRGWEIKTDPQGKSFFHNSRATTFIDPRVPPRYRRFEAKLRNFYRKLEAKGYGQGPGKIKLIVRRDHLLEGTFNQVMAYSRKELQRNKLYITFVGEEGLDYSGPSREFFFLLSQELFNPYYGLFEYSANDTYTVQISPMSAFVENHLEWFRFSGRILGLALIHQYLLDAFFTRPFYKALLRLPTDLSDLEYLDEEFHQSLQWMKENDITDV-LDLTFTVNEEVFGQVTERELKSGGTNVQVTEKNKKEYIERMV----------------------------------------------------------------------------------------------------------------------------------------------------------------

>Dre4

PPNWEARIDSH-GRIFFVDHVNRTTTWQRPLPRGWEMKHDHTGKAFFHNCRATTFIDPRVPASYKRFEAKLRNFYRKMETKGYGQGPGKVKLIIRRDHLLEDAFNQIMCYSRKDLQRSRLYVSFVGEEGLDYSGPSREFFFLVSRELFNPYYGLFEYSANDTYTVQISPMSAFVDNHHEWFRFSGRILGLALIHQYLLDAFFTRPFYKGLLRIPCDLSDLEFLDEEFHQSLQWMKDNDIEDM-LDLTFTVNEEVFGQITERELKPGGSGIAVSDKNKKEYIERMVKWRIERGVAQQTESLVRGFYEVVDVRLVSVFDARELELVIAGTAEIDLSDWRNNTEYRGGYHDNHIVIRWFWAAVERFNNEQRLRLLQFVTGTSSIPYEGFASLRGSNGPRRFCVEKWGKVTSLPRAHTCFNRLDLPPYPSFSMLYEKMVTAVEETSTFG

>Dre5

PPGWEERKDPK-GRTYYVNHNNRSTTWTRPLPPGWEMRIAPNGRPFFHNSRTTTWEDPRLPPRYSRFK-QKYDYFRKKLKKPA-DIPNRFEMKLHRNNIFEESYRRIMSLKRPDSLKARLWIEFESEKGLDYGGVAREWFFLLSKEMFNPYYGLFEYSATDNYTLQINPNSGLCNEHLSYFKFIGRVAGMAVYHGKLLDGFFIRPFYKMMLGKQITLNDMESVDSEYYNSLKWILENDPTE--LDLRFCIDEDNFGQTYQVDLKPSGSDMVVTNDNKKEYIDLVIQWRFVNRVQKQMNAFLEGFTELIPIDLIKIFDENELELLMCGLGDVDVNDWRQHTVYKNGYCPNHPVIQWFWKAVLLMDAEKRIRLLQFVTGTSRVPMNGFAELYGSNGPQLFTIEQWGTPDKLPRAHTCFNRLDLPMYETFEDLREKLLMAVENAQGFE

>Dre6

PPGWEEKQDSK-GRIYFVNHNSRTTTWTRPMPTGWEVRSAPSGRPFFHNTKTTTWDDPRLPYRYSRYK-QKYEYFRKKLKKPA-EIPNRFELSVRRNAVLEDSYRRILSVKRSELLKARLWVEFEGEKGLDYGGVAREWFFLISKEMFNPYYGLFEYSATDNYTLQINPNSGLCNEHLSYFKFIGRVAGMAVYHGKLLDAFFIRPFYKMMLQKPITLQDMESVDSEYFNSLRWILENDPTD--LDLRFTIDEELFGQTHQHELKPGGADIVVNDTNKKEYIHLVMQWRFVDRIQRQMTAFKEGFYELIPQDLIKIFDENELEFLHLRTVTLSHFDWRENTKYKNGYNPNHPAIIWFWKTVLLMDAEKRIRLLQFVTGTSRVPMNGFAELYGSNGPQLFTIEQWGTREKLPRAHTCFDRLDLPPYESFEELRDKLHMAIENAQSFD

>Dre7

PPGWEKRVDQR-GRFYYVDHNTRTTTWQRPLPPGWEKRQDN-GRVYYHNTRTTQWEDPRLPPRYDRFRWKY-HQFRFLCHSN--ALPSHVKISVSRQTLFEDSFQQIMNMKPYDLR-RRLYIIMRGEEGLDYGGIAREWFFLLSHEVLNPMYCLFEYAGKNNYCLQINPASSINPDHLTYFRFIGRFIAMALYHGKFIDTGFTLPFYKRMLNKKPTLKDLESIDPEFYNSIMWVKENDLEECGVELYFAQDMEILGKVTTHQLKDDGENELVTQDNKEEYIGLLTDWRFTRGVEEQTKAFLDGFNEVVPLEWLRYFDEKELELMLCGMQEIDLNDWQKNTIYR-HYTKNSKQIHWFWQVVKEMDNEKRIRLLQFVTGTCRLPVGGFAELIGSNGPQKFCIDKVGKETWLPRSHTCFNRLDLPPYKNLEQLREKLLFAIEETEGFG

>Dre8

------------------------------------------------------------------------------------------TTTTAREHLLVVRRRSPMRYTLSPENLRSLSAQGGSGGGGSRGG--------------APEPMGLQRANSDTDLVTSDSRSSLTAS-----------------------------TYQFTLGRGQNLVI------------SWIKEEDATDW-IGLYH-IDTDETCPANVWDSKNRGVN----GTQRGQIV-----WRLEPGPEPETKICFKYYHGVSLRATTPCITVKNPGVTVGAEGQVDGQSVTEHRKLV-SFTLSDIRAQGLKKGM-FFNPDPYLKM-SIQPG----KRNGFPTFS-HHGQRRTSITNWHGEKYFVALMTDVLEIEVKDKKSRPIIKRFLQLIIPVQRLLE

>Cel1

-----PSDTTIVSNELTAIDDAMRANFERGLPDGWDMQVAPNGRTFFHRTKTTTWTDPRLPPRYSRYK-RKVEYLRSRLPKPN-SNSGKCDMVVHRDTLFEDSYRHIMDKKDYD-LRNKLWIEFFGETGLDYGGVTREWFFLLSHQIFNPYYGLFEYSATDNYTLQINPHSEACNPHLSYFHFIGRIIGMAIYHGKLLDAFFIRPFYKMMLGKKITLFDMESVDNEYYNSLIYVKDNDPAD--LELTFSLDDSIFGETQNIELIPNGANVPVTEDNKEEYIEAV-----------------------VPSNLLRLFDANELELLMCGLQKIDVKDWKANTIYKGGYGPSSQVVHNFWKCILSFDNEMRARVLQFVSGTSRVPMNGFRELYGSNGLQKFTIERWGSADMLPRAHTCFNRLDLPPYTTFKELKSKLLTAIENSEIFS

>Cel2

TPVHKRLSDRSASPRNSPRRTITVRSAGCPLPSGWECITMNN-RTVFHANKETSFYDPRSVLRSANLDITLIKMFYEDMKKEKLKGPSRLCWKVSRDRLLDDAFRIILNVDPFVLKKSRLHIRFEGELALDYGGLSREFFILLSRELFHPKNGYFEYEG-NDYHLQLRPRGCETEKEKKWLILCGRVLALAVIHRCYIDVFFTNVFYKSLQKRPVTLMDFKESDAEFYKSMNWLLENDVVD--LEMSFVVSDLTLSQLAEQELVPGGESQMVTEANKAEFIDLMCQKKAIRGVEKPLEILLTSFNQILNDNLLNSLESSDLKRILSGSLELDLNDWRTNTIYKGGYSDCHIVVEWFWEVIETMTNQERFDLLLFVTGSSSVPFEGFSALRGNEEISKFCIEKWGDATSFPRAHTCFNRLQLPSYNTKQQLKSKLQQAIVNGMSYS

>Cel3

PQGWEMRRDPR-GRVYYVDHNTRTTTWQRPLPEGWEKRQDPNSRMYFHVNRTTQWEDPRLPPRMEKFRWKI-AQFRYLCLSN--SVPNHVKITVSRNNVFEDSFQEIMRKNAVDLR-RRLYIQFRGEEGLDYGGVAREWFFLLSHEVLNPMYCLFMYAGNNNYSLQINPASFVNPDHLKYFEYIGRFIAMALFHGKFIYSGFTMPFYKKMLNKKIVLKDIEQVDSEIYNSLMWIKDNNIDECDMELYFVADYELLGELKTYELKEGGTEIAVTEENKLEYIELLVEWRFNRGVEQQTKAFFTGFNSVFPLEWMQYFDERELELLLCGMQDVDVDDWQRNTVYR-HYAPQSKQVTWFWQWVRSLDQEKRARLLQFVTGTCRVPVGGFSELMGSTGPQLFCIERVGKENWLPRSHTCFNRLDLPPYRSYDQLVEKLSMAIEMTEGFG

>Cin1

PAGWEKRLDSRN-RIYYVDHNTRTTTWQRPLPKGWERRVEANGRVYFHNTRTTQWEDPRMPPRYERFKWKL-GQFRYLCQSN--ALPSHVKINVSRQSIFEDSFSQIMHLQAYDLR-RRLYIMFKGEEGLDYGGVAREWFFLVSHEVLNPMYCLFEYAGSTNYTLQINPASTINPDHMHYFRFVGRFIAMALYHGKFIDTGFSLPFYKRMLNRKLTIKDIESVDEEFYNSLVWIRDNNIEECGLELDFTMDFEVLGKIDTIELKDGGEDIPVTEENKEEYIRLMIDWRFSRGVEKQTKGFLDGFNEVVPLQWLQYFDERELELMLCGMQEFDVEDWSRHSIYR-NYTKNSKQVLWFWQYIREIDNEKRARLLQFVSGTCRIPVGGFAELLGSNGPQKFCIEKVGKETWLPRSHTCFNRLDLPPYKSYEQLKEKLTMAIEETEGFG

>Cin2

PDGWEARVDPKTNRKYFVDHVNKVTSWHKPLPAGWEMKTAPSGRVFFHNRQVTSWDDPRLPPRYSRYK-QKYEFFKSKLHKSVSDIPNRFEMKVDRRTILNDSYRVISRVKKPEFLKSRLWIEFNKEKGLDYGGVAREWFYLLSKEMFNPYYGLFEYSATDNYTLQINPNSGMCNEHFDWFRFIGRVAGMAVYHGKLLDAFFIRPFYKMMLGKPITLRDMESVDSEYYNSLKWILENDPTD--LDLCFTVDEELFGQMKVNELKPGGADIKVNNENKREYIQLVIKWRFVSRVQEQMKSFLQGFNELIPSNLVKIFDENEVELLMCGLGDVDVNDWRRNTNYKGDYSANHIVIQWFWRAVLLMDPELRVRFLQFVTGTSRVPMNGFGELWGSNGPQLFTIEKWGTPEKLPRAHTCFNRLDLPPYKSFEELRKKLLLAIESTQGFE

>Cin3

------------------------------LPSGWEKKVDGHRKPFFHNLRSTTFFDPRVAPT--------VSYNEKVLQQH--AVKHHAKLTVHRDNLLEDAFRKVMLLPRKQLQRSKLFVSFAGEEGLDYSGPSREFFFLISRELFNPYYGLFEYSAVDTYTVQISPLSTFADSPHEWFRFAGRIIGLALVHHCLLDAFFTRPLYKMLLRSKCDLSDLRYEDEQFYQSIMWIKDNDITDV-LDLTFSVNEEMFGKIEERELKPNGKNIAVTEKNKKEYIERMVKWRVVRGTREQTNMLIRGFNEVIDLRLVSVFDANELELVICGTADIDLNDWRQHTEYRGGYYDQHPVVINFWEALDQFDNERRLRLLQFVTGTSSIPYEGFAALRGPNGPKRFCIEKWGKPDCLPRTHTCFNRLDLPPYDTFNLLWEKLVIAIEETNTFG

>Hsa1

PSGWEERKDAK-GRTYYVNHNNRTTTWTRPLPPGWEMRIAPNGRPFFHNTKTTTWEDPRLPPRYSRFK-QKYDYFRKKLKKPA-DIPNRFEMKLHRNNIFEESYRRIMSVKRPDVLKARLWIEFESEKGLDYGGVAREWFFLLSKEMFNPYYGLFEYSATDNYTLQINPNSGLCNEHLSYFTFIGRVAGLAVFHGKLLDGFFIRPFYKMMLGKQITLNDMESVDSEYYNSLKWILENDPTE--LDLMFCIDEENFGQTYQVDLKPNGSEIMVTNENKREYIDLVIQWRFVNRVQKQMNAFLEGFTELLPIDLIKIFDENELELLMCGLGDVDVNDWRQHSIYKNGYCPNHPVIQWFWKAVLLMDAEKRIRLLQFVTGTSRVPMNGFAELYGSNGPQLFTIEQWGSPEKLPRAHTCFNRLDLPPYETFEDLREKLLMAVENAQGFE

>Hsa2

PPGWEEKQDER-GRSYYVDHNSRTTTWTKPLPKGWEVRHAPNGRPFFHNTKTTTWEDPRLPPRYSRYK-RKYEFFRRKLKKQN-DIPNKFEMKLRRATVLEDSYRRIMGVKRADFLKARLWIEFDGEKGLDYGGVAREWFFLISKEMFNPYYGLFEYSATDNYTLQINPNSGLCNEHLSYFKFIGRVAGMAVYHGKLLDGFFIRPFYKMMLHKPITLHDMESVDSEYYNSLRWILENDPTE--LDLRFIIDEELFGQTHQHELKNGGSEIVVTNKNKKEYIYLVIQWRFVNRIQKQMAAFKEGFFELIPQDLIKIFDENELELLMCGLGDVDVNDWREHTKYKNGYSANHQVIQWFWKAVLMMDSEKRIRLLQFVTGTSRVPMNGFAELYGSNGPQSFTVEQWGTPEKLPRAHTCFNRLDLPPYESFEELWDKLQMAIENTQGFD

>Hsa3

PSGWEERKDAK-GRTYYVNHNNRTTTWTRPLPPGWEMRIAPNGRPFFHNTKTTTWEDPRLPPRYSRFK-QKYDYFRKKLKKPA-DIPNRFEMKLHRNNIFEESYRRIMSVKRPDVLKARLWIEFESEKGLDYGGVAREWFFLLSKEMFNPYYGLFEYSATDNYTLQINPNSGLCNEHLSYFTFIGRVAGLAVFHGKLLDGFFIRPFYKMMLGKQITLNDMESVDSEYYNSLKWILENDPTE--LDLMFCIDEENFGQTYQVDLKPNGSEIMVTNENKREYIDLVIQWRFVNRVQKQMNAFLEGFTELLPIDLIKIFDENELELLMCGLGDVDVNDWRQHSIYKNGYCPNHPVIQWFWKAVLLMDAEKRIRLLQFVTGTSRVPMNGFAELYGSNGPQLFTIEQWGSPEKLPRAHTCFNRLDLPPYETFEDLREKLLMAVENAQGFE

>Hsa4

PPNWEARIDSH-GRVFYVDHVNRTTTWQRPLPRGWEIKTDQQGKSFFHNSRATTFIDPRLPPRYRRFEAKLRNFYRKLEAKGFGQGPGKIKLIIRRDHLLEGTFNQVMAYSRKELQRNKLYVTFVGEEGLDYSGPSREFFFLLSQELFNPYYGLFEYSANDTYTVQISPMSAFVENHLEWFRFSGRILGLALIHQYLLDAFFTRPFYKALLRLPCDLSDLEYLDEEFHQSLQWMKDNNITDI-LDLTFTVNEEVFGQVTERELKSGGANTQVTEKNKKEYIERMVKWRVERGVVQQTEALVRGFYEVVDSRLVSVFDARELELVIAGTAEIDLNDWRNNTEYRGGYHDGHLVIRWFWAAVERFNNEQRLRLLQFVTGTSSVPYEGFAALRGSNGLRRFCIEKWGKITSLPRAHTCFNRLDLPPYPSYSMLYEKLLTAVEETSTFG

>HsaItch

PPGWERRVDNM-GRIYYVDHFTRTTTWQRPLPPGWEKRTDSNGRVYFHNTRITQWEDPRLPPRYVRFKAKV-QYFRFWCQQL--AMPQHIKITVTRKTLFEDSFQQIMSFSPQDLR-RRLWVIFPGEEGLDYGGVAREWFFLLSHEVLNPMYCLFEYAGKDNYCLQINPASYINPDHLKYFRFIGRFIAMALFHGKFIDTGFSLPFYKRILNKPVGLKDLESIDPEFYNSLIWVKENNIEECDLEMYFSVDKEILGEIKSHDLKPNGGNILVTEENKEEYIRMVAEWRLSRGVEEQTQAFFEGFNEILPQQYLQYFDAKELEVLLCGMQEIDLNDWQRHAIYR-HYARTSKQIMWFWQFVKEIDNEKRMRLLQFVTGTCRLPVGGFADLMGSNGPQKFCIEKVGKENWLPRSHTCFNRLDLPPYKSYEQLKEKLLFAIEETEGFG

>Hsa5

PPGWEEKQDER-GRSYYVDHNSRTTTWTKPLPKGWEVRHAPNGRPFFHNTKTTTWEDPRLPPRYSRYK-RKYEFFRRKLKKQN-DIPNKFEMKLRRATVLEDSYRRIMGVKRADFLKARLWIEFDGEKGLDYGGVAREWFFLISKEMFNPYYGLFEYSATDNYTLQINPNSGLCNEHLSYFKFIGRVAGMAVYHGKLLDGFFIRPFYKMMLHKPITLHDMESVDSEYYNSLRWILENDPTE--LDLRFIIDEELFGQTHQHELKNGGSEIVVTNKNKKEYIYLVIQWRFVNRIQKQMAAFKEGFFELIPQDLIKIFDENELELLMCGLGDVDVNDWREHTKYKNGYSANHQVIQWFWKAVLMMDSEKRIRLLQFVTGTSRVPMNGFAELYGSNGPQSFTVEQWGTPEKLPRAHTCFNRLDLPPYESFEELWDKLQMAIENTQGFD

>Hsa6

PPGWERRVDDR-RRVYYVDHNTRTTTWQRPLPPGWEKRVDSTDRVYFHNTKTTQWEDPRLPPRYERFRWKL-AHFRYLCQSN--ALPSHVKINVSRQTLFEDSFQQIMALKPYDLR-RRLYVIFRGEEGLDYGGLAREWFFLLSHEVLNPMYCLFEYAGKNNYCLQINPASTINPDHLSYFCFIGRFIAMALFHGKFIDTGFSLPFYKRMLSKKLTIKDLESIDTEFYNSLIWIRDNNIEECGLEMYFSVDMEILGKVTSHDLKLGGSNILVTEENKDEYIGLMTEWRFSRGVQEQTKAFLDGFNEVVPLQWLQYFDEKELEVMLCGMQEVDLADWQRNTVYR-HYTRNSKQIIWFWQFVKETDNEVRMRLLQFVTGTCRLPLGGFAELMGSNGPQKFCIEKVGKDTWLPRSHTCFNRLDLPPYKSYEQLKEKLLFAIEETEGFG

>Hsa7

PPNWEARIDSH-GRIFYVDHVNRTTTWQRPLPRGWEMKHDHQGKAFFHNSRTTTFIDPRVPPRYKRFEAKLRNFYRKLETKGYGQGPGKLKLIIRRDHLLEDAFNQIMGYSRKDLQRNKLYVTFVGEEGLDYSGPSREFFFLVSRELFNPYYGLFEYSANDTYTVQISPMSAFVDNHHEWFRFSGRILGLALIHQYLLDAFFTRPFYKALLRILCDLSDLEYLDEEFHQSLQWMKDNDIHDI-LDLTFTVNEEVFGQITERELKPGGANIPVTEKNKKEYIERMVKWRIERGVVQQTESLVRGFYEVVDARLVSVFDARELELVIAGTAEIDLSDWRNNTEYRGGYHDNHIVIRWFWAAVERFNNEQRLRLLQFVTGTSSIPYEGFASLRGSNGPRRFCVEKWGKITALPRAHTCFNRLDLPPYPSFSMLYEKLLTAVEETSTFG

>Hsa8

EPVYGLSEDEGESRILRVS--------------GIDLAKKDIGAS-----------DPY-------LALVQTKTIKKTLNPKW-NEEFYFRVNPSNHRLLFEVFDE-NRLTRDDFL-GQVDVPLSH---LPTEDPTMERPYTFKDFLLRPRSHKSRVKGFLRLKMAYMPKNGGQDEQRDDMEHGWEVVDSNDSASQHQEELPPPP-------LPPGWEEVDNLGRTYYRTTQWLMDVSESDNNRQINQEAAHRRFRRHISEDLEPEGGDVPEP-------------WETI---SEEVNIAGDSLLALPPASPGSRTSPQELSEELSRRLQIDSNG----EQFS-----------------SLIQREPSSRL-RSCSVTDAVAEQGHLP---PEDPLKFPV--------HMRSKTSLNPNDLGPLEERIHLDGR-TFYIDNSKITQ

>Hsa9

PSGWEERKDAK-GRTYYVNHNNRTTTWTRPLPPGWEMRIAPNGRPFFHNTKTTTWEDPRLPPRYSRFK-QKYDYFRKKLKKPA-DIPNRFEMKLHRNNIFEESYRRIMSVKRPDVLKARLWIEFESEKGLDYGGVAREWFFLLSKEMFNPYYGLFEYSATDNYTLQINPNSGLCNEHLSYFTFIGRVAGLAVFHGKLLDGFFIRPFYKMMLGKQITLNDMESVDSEYYNSLKWILENDPTE--LDLMFCIDEENFGQTYQVDLKPNGSEIMVTNENKREYIDLVIQWRFVNRVQKQMNAFLEGFTELLPIDLIKIFDENELELLMCGLGDVDVNDWRQHSIYKNGYCPNHPVIQWFWKAVLLMDAEKRIRLLQFVTGTSRVPMNGFAELYGSNGPQLFTIEQWGSPEKLPRAHTCFNRLDLPPYETFEDLREKLLMAVENAQGFE

>Hsa10

PPGWEKRTDPR-GRFYYVDHNTRTTTWQRPLPPGWEKRQDN-GRVYYHNTRTTQWEDPRLPPRYDRFRWKY-HQFRFLCHSN--ALPSHVKISVSRQTLFEDSFQQIMNMKPYDLR-RRLYIIMRGEEGLDYGGIAREWFFLLSHEVLNPMYCLFEYAGKNNYCLQINPASSINPDHLTYFRFIGRFIAMALYHGKFIDTGFTLPFYKRMLNKRPTLKDLESIDPEFYNSIVWIKENNLEECGLELYFIQDMEILGKVTTHELKEGGESIRVTEENKEEYIMLLTDWRFTRGVEEQTKAFLDGFNEVAPLEWLRYFDEKELELMLCGMQEIDMSDWQKSTIYR-HYTKNSKQIQWFWQVVKEMDNEKRIRLLQFVTGTCRLPVGGFAELIGSNGPQKFCIDKVGKETWLPRSHTCFNRLDLPPYKSYEQLREKLLYAIEETEGFG

>Xtr1

------------------------------------------------------MASPS-------------SQNRRRCKEP-------------------------MRYSYNPDQFHNMDIRNNPHEGVTMPRSTSDTDLVTS----------------DSRSTLMVSSSYYSIGLVIYWDIKEEVDAGDWIGMYLIDEVLSENF--------LDYKN-RGVNGSHRGQIVWKIEASYFVESTKICFKYYHGVSGALRATT--P-----SITVKNPTAPICLCCNFI-----TCYMSHLMYSYETLF------------LDFQALGLKKMNPDPYLKI-----SIQPGKHSIF---PALPHHGQEKRSKIV--CNTVNPV----WKREHH----KFFAI--FSASLRLRKN----NRKDAE--KIAKDFLGKLSKTVASLVVYL

>Xtr2

-----------------------------------------------RFLGLAAMASPS-------------SQNRRRCKEP-------------------------MRYSYNPDQFHNMDIRNNPHEGVTMPRSTSDTDLVTS----------------DSRSTLMVSSSYYSIGLVIYWDIKEEVDAGDWIGMYLIDEVLSENF--------LDYKN-RGVNGSHRGQIVW-----------------------KIEASSFVE--SETKIC-------------FKYYHGVSGALRATTPSIVKNPTAPIFKNINNEETQ--GQGSRRITLSDFQAL-----GLKKG-----------MFFNPDPYLKI-SIQPGKHSI----FPALP-HHGQKRSKI-------------VC---------NTVNPVWKRE-----------

>Xtr3

PPGWERRVDNM-GRIYYVDHITRTTTWQRPLPVGWEKRMDGNGRVYFHTTRTTQWEDPRLPPRYVRFKAKV-QYFRFWCQQL--YMPQHVKITVNRKTLFEDSFQQIMSFNAQDLR-RRLWIIIPGEEGLDYGGVAREWFFLLSHEVMNPMYCLFEYAGKDNYCLQINPASYINPDHLRYFRFIGRFIAMALFHGKFIDTGFSLPFYKRILNKPVGLKDLESVDPEFYNSLIWIKDNNIEECGLEMFFSVDKEILGEVKSHDLKPDGSNIQVTEENKEEYIRLVAEWRLSRGVEEQTQAFFEGFNEILPQQYLQYFDAKELEVLLCGMQEIDLNDWQRNTIYR-HYTRTSKQIIWFWQFVKEIDNEKRMRLLQFVTGTCRLPVGGFADLMGSNGPQKFCIEKVGKENWLPRSHTCFNRLDLPPYKSYEQLKEKLLFAIEETEGFG

>Xtr4

PPNWEARIDSH-GRVFYVDHVNRTTTWQRPLPRGWEIKTDQQGKSFFHNSRATTFIDPRVPPRYRRFEAKLRNFYRKLEAKGYGQGPGKIKLIIRRDHLLEGTFNQVMAYSRKELQRNKLYITFVGEEGLDYSGPSREFFFLLSQELFNPYYGLFEYSANDTYTVQISPMSAFVENHLEWFRFSGRILGLALIHQYLLDAFFTRPFYKALLRLPCDLSDLEYLDEEFHQSLQWMKDNDITDI-LDLTFTVNEEVFGQVTERELKSGGANIQVSEKNKKEYIEKMVKWRVERGVVQQTEALVRGFYEVVDSRLVSVFDARELELVIAGTAEIDLNDWRNNTEYRGGYHDGHIVIRWFWAAVERFNNEQRLRLLQFVTGTSSVPYEGFAALRGSNGLRRFCIEKWGKITSLPRAHTCFNRLDLPPYPSYSMLHEKLLIAVEETSTFG

>Xtr5

PSGWEERKDAK-GRTYYVNHNNRTTTWTRPLPPGWEMRIAPNGRPFFHNTKTTTW-DPRLPPRYSRFK-QKYDYFRKKLKKPA-DIPNRFEMKLHRNNIFEESYRRIMSVKRPDVLKARLWIEFESEKGLDYGGVAREWFFLLSKEMFNPYYGLFEYSATDNYTLQINPNSGLCNEHLSYFTFIGRIAGLAVFHGKLLDGFFIRPFYKMMLGKQITLKDMESVDSEYYNSLKWILENDPTE--LDLRFCIDEENFGQTYQVDLKPNGSEMVVTNDNKREYIDLVIQWRFVNRVQKQMNAFLEGFTELIPIDLIKIFDENELELLMCGLGDVDVNDWRQHTLYKNGYCPNHPAIQWFWKAVLLMDAEKRIRLLQFVTGTSRVPMNGFAELYGSNGPQLFTIEQWGSPDKLPRAHTCFNRLDLPPYDSFEDLREKLLMAVENAQGFE

>Xtr6

PLGWEKRVDNR-GRFYYVDHNTRTTTWQRPLPNGWEKRQD-NGRVYYHNTRTTQWEDPRLPPRYDRFRWKY-HQFRFLCHSN--ALPSHVKISVSRQTLFEDSFQQIMNMKPYDLR-RRLYIIMRGEEGLDYGGIAREWFFLLSHEVLNPMYCLFEYAGKNNYCLQINPASSINPDHLTYFRFIGRFIAMALYHGKFIDTGFTLPFYKRMLNKKPTLRDLESIDPEFTTLFLYYRDNNLEECELELYFVQDMEILGEVTSHKLKEGGENIRVTEENKEEFISLLTDWRFTRGVEEQTNAFLDGFKEVVPLEWLRYFDEKELELMLCGMQEIDIADWQKNTIYR-HYTKNSKQVQWFWQVVKEMDNEKRIRLLQFVTGTCRLPVGGFVELIGSNGPQKFCIDRVGKDTWLPRSHTCFNRLDLPPYKSYEQLKEKLLFAIEETEGFG

>Xtr7

PPNWEARIDSH-GRVFYVDHVNRTTTWQRPLPRGWEIKTDQQGKSFFHNSRATTFIDPRYPPRYRRFEAKLRNFYRKLEAKGYGQGPGKIKLIIRRDHLLEGTFNQVMAYSRKELQRNKLYITFVGEEGLDYSGPSREFFFLLSQELFNPYYGLFEYSANDTYTVQISPMSAFVENHLEWFRFSGRILGLALIHQYLLDAFFTRPFYKALLRLPCDLSDLEYLDEEFHQSLQWMKDNDITDI-LDLTFTVNEEVFGQVTERELKSGGANIQVSEKNKKEYIEKMVKWRVERGVVQQTEALVRGFYEVVDSRLVSVFDARELELVIAGTAEIDLNDWRNNTEYRGGYHDGHIVIRWFWAAVERFNNEQRLRLLQFVTGTSSVPYEGFAALRGSNGLRRFCIEKWGKITSLPRAHTCFNRLDLPPYPSYSMLHEKLLIAVEETSTFG

>Xtr8

PPGWEEKHDDK-GRSYYIDHNSRTTTWEKPLPKGWEVRHAPTGRPFYHVTKTTTWEDPRLPPRLLRHILPTFHYFWEIWKNKI-DIPNRFEMKLRRTAILEDSYRRIIAVKRPEFLKARLWIEFDNEKGLDYGGVAREWFFLISKEMFNPYYGLFEYSATDNYTLQINPNSGLCNEHLSYFKFIGRVAGMAVYHGKLLDAFFIRPFYKMMLQKPIILYDMESVDSEYYNSLQWILENDPSG--LDLCFTVDEELFGQTHQHELKAGGSQILVTNKNKKEYIHLVIQWRFMNRVQKQMAAFKEGFFELIPQDLIKIFDENELELLMCGLGDVDVNNWREHTKYKNGYSQGHQVIQWFWKAVLMMDAEKRIRLLQFVTGTSRVPMNGFAELYGSNGPQLFTVEKWGTPEKLPRAHTCFNRLDLPPYESFEDLWDKLHIAIENAQGFD

>Xtr9

PPGWEEKHDDK-GRSYYIDHNSRTTTWEKP-------------------------EDPRLPPRYSSYVFPV---FLGKISHQI-DIPNRFEMKLRRTAILEDSYRRIIAVKRPEFLKARLWIEFDNEKGLDYGGVAREWFFLISKEMFNPYYGLFEYSATDNYTLQINPNSGLCNEHLSYFKFIGRVAGMAVYHGKLLDAFFIRPFYKMMLQKPIILYDMESVDSEYYNSLQWILENDPSG--LDLCFTVDEELFGQTHQHELKAGGSQILVTNKNKKEYIHLVIQWRFMNRVQKQMAAFKEGFFELIPQDLIKIFDENELELLMCGLGDVDVNNWREHTKYKNGYSQGHQVIQWFWKAVLMMDAEKRIRLLQFVTGTSRVPMNGFAELYGSNGPQLFTVEKWGTPEKLPRAHTCFNRLDLPPYESFEDLWDKLHIAIENAQGFD

>Sce

PPGWERRTDNF-GRTYYVDHNTRTTTWKRPLPSGWEQRFTPEGRAYFHNTRTTTWVDPRLPPRYKRFR-RKVIYFRSQPALR--ILPGQCHIKVRRKNIFEDAYQEIMRQTPEDLK-KRLMIKFDGEEGLDYGGVSREFFFLLSHEMFNPFYCLFEYSAYDNYTIQINPNSGINPEHLNYFKFIGRVVGLGVFHRRFLDAFFVGALYKMMLRKKVVLQDMEGVDAEVYNSLNWMLENSIDGV-LDLTFSADDERFGEVVTVDLKPDGRNIEVTDGNKKEYVELYTQWRIVDRVQEQFKAFMDGFNELIPEDLVTVFDERELELLIGGIAEIDIEDWKKHTDYR-GYQESDEVIQWFWKCVSEWDNEQRARLLQFTTGTSRIPVNGFKDLQGSDGPRRFTIEKAGEVQQLPKSHTCFNRVDLPQYVDYDSMKQKLTLAVEETIGFG

>Ecu

PAEGRAEVEALICRRLYIDKDTNCYEWPANLSLSFYDRLSPDRPVFFAKMKGVVFKEADLDYRLNRFGNKK-KYLSRMLSVEGAKSSSFYRVYVDRSDVLRSSYFQVMAKSPEEFRTRRLEIKLTGEEGLDYGGLTREWLVLLAKDLLDPNFALFEFATEDKTTVVPCKNSYVNPEHLSYFKFVGRIIAKAIMDGNFINLHLSKFIYQYILGKSCDLQDLESADPEFHKSLVWIRDNPVDKS-LGITFSFDDVSFGVHRTVELVEGGAHVFVDDSNKAEYVKLATQYRLFNGIELQLSALKSGLFEILGSKALEMFDESELELLICGIPDIDVDDWKNNTLYY-GYAENSKTVIWFWRAVKSLDSVSRAKLLQFVTGTSTLPFEGFSHLQGNNEVQKFSIHKVSDRIDLPTAHTCFNQLVLPEYSSYENLLKYLTLAINCSTGFG

>Pfa

PPSWNNRNINRPNRNIFRNNNNNENVFNRHILNSSANSTTNNATEYLNNMNNNNNNNNNLRPEYLRFENKR-HYLRKKLKYLKSIRSDPIRLSIRREKVFTDSYYQ-LRNKSGNDLKGKLVVTFKNEEGVDAGGLTREWYSILAKEIFNPNYALFCREGKKSEFNHPNPLSYINPDHLHFFKFVGKFIAKAIYDGQVIDAYFCRSFYKHMLGRKILPADAESVDPEFYNSLIKISEYKLEDLNLEINFSTEIDEFGKTKVIDLIPNGRNIPVTDENKHKYIELLCELKVTNSIKEQLEAFMDGFKELIQPKLISIFDDKELELLISGIPTIDLNDLKENVEYH-NYTANSIQIIWLWDVLQEFDENKKASFLQFVTGTSRVPLGGFKNLMGMRGAQKMII--YGEDR-LPTAHTCFNQLDLPEYSSKELLKSKLIRAIMGKEGFG

>Bfl

-MEWESYVGESTLASVQEKHDPRQAAAASPLPSGWEERVHTDGRTFFHNTKNTTWEDPR----YSRYKRK-YEYFRSKLRRPANPQNQKFEMKLHRNSILEDSYRTIMACKKADNLKAKLWIDFEGEKGLDYGGVAREWFYLLSHEMFNPYYGLFEYSANDNYTLQINPNSGLCNEHLSYFKFIGRVAGMAVYHGKLLDAFFIRPFYKMMLKKPITLKDMESVDSEYYNSLVWITENDPED--LDLRFCVEEDQFGQMVTKNLKANGEDILVTNSNKKEYIDLVIKWRFSSRVQEQMKALMDGFNELVQQELLSIFDEREVELLMCGLGDIDVNDWRKHTAYRGDYSDKHPIIQWFWKAVILMDPETRVRLLQFVTGTSRVPMNGFAELWGSNGPQKFTIEKWGNPDQLPRAHTCFNRVDLPPYRSFQELWDKLKIAIENTEGFE

>Oca

------------------------------------------------------------------------------------------------------------------------------------------------------------------------------------------------------------------------------------------------E--LDLMFCIDEENFGQTYQVDLKPNGSEIMVTNENKREYIDLVIQWRFVNRVQKQMNAFLEGFTELLPIDLIKIFDENELELLMCGLGDVDVNDWRQHSIYKNGYCPNHPVIQWFWKAVLLMDAEKRIRLLQFVTGTSRVPMNGFAELYGSNGPQLFTIEQWGSPEKLPRAHTCFNRLDLPPYETFEDLREKLLMAVENAQGFE

>Ngr

-------------------------------------------------------------------------------------------MSVRRSQIFTDSYFY-INGLLPEECKGKLQIKFEGEEGYDAGGVKREWFSTLSREMLYPGYALFAPCA-DRTTYHPNPSSYVNQEHLSYFRFTGRIIAMAIYNEQPLDCHFTRSFYKHILGIPITYHDIESIDPSYYKNLKWMLTNSIGDV-LFHTFTHEFDEFGKTKEIELKPNGKNIPVTDENKAEYVRLVTELKMTKSIEKQLEQFLKAFYDIIPRKLIQIFNEQELELLISGLPDIDIQDLKNNTIYGHGYTKDSVHIKWFWNVVESFGKDDKALLLQFVTGTSKVPLGGFSQLIGANGEQLFCIQKVGSQR-LPTAHTCFNQLDLPEYDTEDVLRERLLVALRGSEGFG

>Hma

-------------------------------------------------------------------------------------------------------------------------------HA---------------SDLFYP-----------------------------------------------------------------------------------SKENSIDNCGLDLTFATDMEVLGKLTKHELKVGGKEIEVTDANKSEYIELITDWRFSRGVVDQTKSFLSGFNEVLPMYWLQSFDERELETLLCGMQEYDLDDWQRNTVYR-TYQKTSKQILWFWQAVKSFDNEKKSRLLQFVTGTCRLPVGGFAELIGSNGPQKFCIEKVGKETWLPRSHTCFNRLDLPPYRSYEQLLEKLTFAIEETEGFG

>Pra

-----------------ASETVRLASFVES---NLLVREKPS-----DTSLAALIKIPR-------YFHSAMKRLRQTALRNHGGGSSSVRIPVRREHIFEDSYYA-LRMRSGTELRRKLHISFTGEEGIDAGGVTREWYMILAREMFNPNYVLFTSAA-DSPTFQPNPLSYVNKDHLSYFEFVGKVLGKAVADGQLLDAHFTRSFYKHILQLSISYHDMEAIDPEYYRNLHSILDNSIADLGLELTFSAEQSNFGKVEVVDLIPNGRNVNVTDENKMEYVKLVTHHRMATGIRQQIDAFLKGFHQLVPPELIAIFNENELELLISGMPEIDIDDLKANTEYA-NYKPTDSVIRWFWNVLYSFTHEERALFLQFVTGTSKVPLEGFKALEGMRGTQKFNIHKFGNNSALPSAHTCFNQLDLPEYESEEKLKQCLLLAIRGSEGFG

>Uma1

PAGWERRTDHL-GRTYYVDHNTRSTTWTRPLPAGWEQRHTPEGRPYFHNTRTTTWVDPRLPPRYKRFR-RKLIYFRSQPALR--PIPGQCHIKVRRTHIFEDSYAEIMRQQPNDLK-KRLMIKFDGEDGLDYGGLSREFFFLLSHEMFNPFYCLFEYSAHDNYTLQINPHSGINPEHLNYFKFIGRVLGLAIFHRRFLDAYFIVSFYKMILKKKITLSDLESVDADYHRSLQWMLDNSIEGI-VEETFTAVEDKFGEMVTVELKKGGEEVEVTDENKKEYVDLMTEWRISKRVEEQFKAFISGFTELIPQDLINVFDERELELLIGGMSEIDVDDWKKFTDYR-GFTEQDQVVQWFWQCVRAWPTEKKSRLLQFATGTSRIPVNGFKDLQGSDGPRRFTIEKSGDVNQLPKSHTCFNRIDLPPYPSFETLESKLALAIEEGMGFG

>Uma2

PKSESRKKDFDQSEAALYESFEFDVLWSKLMEDNFFAFTEKH-----KILNIMVRQNPS--GS---FDNKK-NYFTQQLHKGRRHYT-PLSLSVRRNSVFEDSFRYFSRKTGPEVKHGKLNVRFTNEEGIDAGGVTREWFQVLARAMFNPDYALFQPCAADRTTYQPNRMSYVNPDHLSFFKFVGRIIGKAIYDGRLLDAYFTRSFYKHILGKPVDYRDLESIDPEYFKSLEWMLSNDITDI-LDLTFSVDDEEFGETKVVDLKPNGTSISVTEANKQEYVRLVTEQRLTKSIKSQIDAFLGGFNEIIPSDLIRIFSEQELELLISGLPDIDVDAWKNNTELH-GYSSGDAVVQWWWRAVRSFDQTEKAKLLQFITGTSKVPLEGFAHLQGVQGTQRFNIHKAYGADRLPAAHTCFNQLDLPQYESYEKLRSSLLLAMNGGEGFG

>Pso

---------------------MRLANFVES---NLLVREKPS-----DTSLAALIKIPR-------YFHSAMKRLRQTALRNHGGGSSSVRIPVRREHIFEDSYYA-LRMRSGTELRRKLHISFTGEEGIDAGGVTREWYMILAREMFNPNYVLFTSAA-DSPTFQPNPLSYVNKDHLSYFEFVGKVLGKAVADGQLLDAHFTRSFYKHILQLPISYHDMEAIDPEYYRNLHSILDNSIVDLGLELTFSAEQSNFGKVEVVDLIPNGRNVAVTDENKMEYVKLVTHHRMATGIRQQIDAFLKGFHQLVPPEMIAIFNENELELLISGMPEIDIDDLKANTEYA-NYKPTDSVIRWFWNVLYSFTHEERALFLQFVTGTSKVPLEGFKALEGMRGTQKFNIHKFGNNSALP-----FNQLDLPEYESEEKLKQCLLLAIRGSEGFG

>Ehi

VREYNDGSNDDNDEMQFSDHQNNSEVSQHDEPQQIEKKEEKEEEEYVNYSNLLNYVKKYLSSS---FEMKYQDLIKEIGKHK--IYNHFMKIIIDRNNVFNSLFKQ---IKHSEKWDRLFKIEFKHEQGNDNGGLLKECYSLLVKELSNGIEDLFII---ENNYIVPNYQCFKKNQ----FIFIGKCLAKMIYDGFTSDFHLALFVLRLLLSKRNELKDVKMFDKEMSQQINLFLE-DVTE--WGLTFTETERINNKIHEVELKTNGANILVTQENKKEYIDLLLQYKFNTRIEKQIKWIKKGFYSLIDESTIQLFDEKELDRIICG-NNFDIEDLKRNVVLD-GYTKESLQIIWLWEIINEMENSLKGKFIQFVTGSSRAPIGGFVQLKSSHGTLPFTIASYDNPDSMPTAHTCANRLEIPFYSNKDILKSKLTIAIQECCNYE

>Ddi

NSSFKSTRTQDFDDETYLDLDDINKIFNLLIFYEWFNHLSKE---AFTLTNFEQFISVRVPKRYQIHALTQ-NSLRTQFGQMGISAADFLIFKIRREHLIDDTLDKTREENRKSELRKELKVHFMGEEGIDEGGVKREFFQLIVRKIFDPEYGMFKYNT-TTNCFWFNPDSVDYTE----FELIGIIIGLALYNNIILDVHFPLVIFKKLLNLDLTMEDIESLDPEVYQSFKIKTTEDVSD--WSLYFCTYESIYGQTKVHNLKQGGDDIQVTNENRNEYLDLYKDYLLNKSISKQFAPFFKGFRMVCDSPILKILKPEELGNLVCGVEDLNFIELERGTNYEGGYTQDDTTIKNFWKVLHGLSDEDKRRFLTFVTGGDRVPYGGLEK-------MSFTITKTADSDRLPSAHTCFNTLILPAYPEFEKLRDLLTKALTHYEGFG

>Ath

PSSLSASIAQLIEEHFIRASAPVNTVVERESPLGSLDGRAPENAIAASEAVLAALPSPLMEPTFEKFDNKK-AYFRSRIRHQHDHISGPLRISVRRAYVLEDSYNQ-LRMRSPQDLKGRLNVQFQGEEGIDAGGLTREWYQLLSRVIFDKGALLFTTVG-NDATFQPNPNSVYQTEHLSYFKFVGRMVAKALFDGQLLDVYFTRSFYKHILGVKVTYHDIEAVDPDYYKNLKWLLENDVSDI-LDLTFSMDADEEKEVTDYELKPGGRNIRVTEETKHEYVDLVAGHILTNAIRPQINAFLEGFNELIPRELVSIFNDKELELLISGLPEIDFDDLKANTEYT-SYTAGSPVIHWFWEVVKAFSKEDMARFLQFVTGTSKVPLEGFKALQGISGPQRLQIHKYGAPERLPSAHTCFNQLDLPEYQSKEQLQERLLLAIHASEGFG

>Vca

------------------------------------------------------MLVPR-------FDNKR-AWFRSKVRATPDERPGSLRLAVRREHVFEDSFYQ-LRGRPAEEMKLKLNVTFQGEEGIDAGGVTREWYQVMAREMFNPNLALFVAVPDGGSTFQPNPNSHVQNDHLDYFRFVGRVVGKALYDGQLIDAYFTRSFYKHLLGSPLTHVDLEAVDPEYYKALAWMLSNDITDV-LDLTFTAETDFFGRKETVELVPGGKDIRVTESNKREYVNLVARHRMTTSITAQINAFLEGFWQLVPRHLIAIFNDHELELLISGLPDIDVDDLRASTEYS-GYSATSPVVRWFWEAVGEMDKQERAQLVQFVTGTSKVPLEGFKALQGISGPQKFQIHKYGDGSRLPSAHTCFNQLDLPEYESKEQLVERLKVAVHGNVGFG

>Lma

GSGFGERAGHD-GAAARPNGVESTDTWP-PVENRREDDDDDDSHALYTVTPTSSLQQPRLPPRFHSFNFKL-TDFRRRLGSRRGNIL----LRVNRQTCLLDSFKE---LQKVKSFGGQLHIRFHGEEGADAGGLTREWLQLLSEAIVDERYALFIHSQ-DSISFQPNPFSSVNPNHLEYFQFAGVVTGLAIAHNVPIDIHFTRAFYRHIIGHRPVFADLQSFDPELYTNLNWIMENDVTD--LGLTFAVNYDRFGSVEEAELEPNGQNTAVTNANKQQYVRLLCEFYMTKRTEDQLLRFLKGFYSVIPRREIQCFTEKELELVISGMPNIDVEDLRTHTVYE-GYSSTSPQVRWFWEAVGSMSKEDLANLLQFTTGSSKVPHGGFGHLEGSNGRLPFTISRWVTKEDLPQAHTCFNKIDLPVYPSAAVLKEKLMLAITGSMGFT

>Spo1pub

PPGWERRTDNL-GRTYYVDHNTRSTTWIRPLPPGWEQRYTPEGRPYFHNTRTTTWVDPRLPPRYKRFR-RKLIYFLSQPALH--PLPGQCHIKVRRNHIFEDSYAEIMRQSATDLK-KRLMIKFDGEDGLDYGGLSREYFFLLSHEMFNPFYCLFEYSSVDNYTLQINPHSGINPEHLNYFKFIGRVIGLAIFHRRFVDAFFVVSFYKMILQKKVTLQDMESMDAEYYRSLVWILDNDITGV-LDLTFSVEDNCFGEVVTIDLKPNGRNIEVTEENKREYVDLVTVWRIQKRIEEQFNAFHEGFSELIPQELINVFDERELELLIGGISEIDMEDWKKHTDYR-SYSENDQIIKWFWELMDEWSNEKKSRLLQFTTGTSRIPVNGFKDLQGSDGPRKFTIEKAGEPNKLPKAHTCFNRLDLPPYTSKKDLDHKLSIAVEETIGFG

>Spo2pub

GCAWETRIDEF-GHVYYLKSPQLSVISAISLPAGWEMRLSEDYHVYFHSTKTTTWSDPR-------YQ-RKIAYMYDRPEMA--VNDAQLQLKVSRATTFEDAYDIISKLSVSDMK-KKLLIRFRNEDGLDYGGVSREFFYILSHAIFNPGYSLFEYATDDNYGLQISPLSSVNPDFRSYFRFVGRVMGLAIYHRRYLDVQFVLPFYKRILQKPLCLEDVKDVDEVYYESLKWIKNNDVDES-LCLNFSVEENRFGESVTVDLIPNGRNIAVNNQNKMNYLKALTEHKLVTSTEEQFNALKGGLNELIPDSVLQIFNENELDTLLNGKRDIDVQDWKRFTDYR-SYTETDDIVIWFWELLSEWSPEKKAKLLQFATGTSRLPLSGFKDMHGSDGPRKFTIEKVGHISQLPKAHTCFNRLDIPPYNSKEELEQKLTIAIQETAGFG

>Spo3pub

PPGWERRADSL-GRTYYVDHNTRTTTWTRPLPFGWEMRYTDTGRPYFHNTRTTTWVDPRLPPRYKCFR-RKLIYFRSQPGMR--PLPGQCNVKVRRDHIFEDSYAEIMRYSAHDLK-KRLMIRFDGEDGLDYGGLSREFFFLLSHKMFDPIYCLFEYSAVDNYTLQINPHSSINPEHLNYFRFIGRVIGLAIFHRRFLDAFFVVSLYKKLLRKKVSLADMESIDAEFYRSLKWVLENDITGI-LDLTFSVEEDHFGEVRTVELITNGENIEVTEENKKKYVDLVTEWRVSKRVEQQFNAFYSGFVELVSPDLVNVFDERELELLIGGISDVDVEDWKSHTEYR-TYIATDPVIKWFWEIIAGWKNEDRSKLLQFATGTSRIPVNGFRDLQGSDGPRKFTIEKAGTPDQLPVAHTCFNRLDLPDYPSKDTLHEKLSLAVENTVGFG

**NEURALIZED**

>Lgi1

PLCFHD-IHGENIRLNADKTRATRY-ESFCKGICFSNRPIAVNEKVYINFAEVSTSWSGVLRFGFTGTDPMTYNPVELPRYACPDLTNKPGTFAKALSERFAKTNTVMCFYVTRGGDVFFSVNGEDKGLFFNGVN--TNGPLWALLDIYGNTISVEFLESELFSFQTLSLNDSTSLPVRYHANVELTPMTFHAMC-GRNVRLNPDMTIASRTMDEYCNAYAFTSRPLHCGEKIVIQILNIEQLYVGGIAFGLTVCDPASLIMD-D---LPDDSDYLLDRK----EYWVVNKDICRKPDVGDELSFYLTSEGEVR-YSRNNHKVATLMHVDRNL-PLWAFFDVYGNVQ-KIKILPSILAPLHPS-------LPTSLPSSECSVCC--ERSVNSVLYTCGHMCMCFDCALI-VKRE--KGGLCPICRQPIKDVIKTYR

>Lgi2

PLCFHD-IHGENIRLNADKTRATRY-ESFCKGICFSNRPIAVNEKVYINFAEVSTSWSGVLRFGFTGTDPMTYNPVELPRYACPDLTNKPGTFAKALSERFAKTNTVMCFYVTRGGDVFFSVNGEDKGLFFNGVN--TNGPLWALLDIYGNTISVEFLESELFSFQTLSLNDSTSLPVRYHANVELTPMTFHAMC-GRNVRLNPDMTIASRTMDEYCNAYAFTSRPLHCGEKIVIQILNIEQLYVGGIAFGLTVCDPASLIMD-D---LPDDSDYLLDRK----EYWVVNKDICRKPDVGDELSFYLTSEGEVR-YSRNNHKVATLMHVDRNL-PLWAFFDVYGNVQ-KIKILPSILAPLHPS-------LPTSLPSSECSVCC--ERSVNSVLYTCGHMCMCFDCALI-VKRE--KGGLCPICRQPIKDVIKTY-

>Bfl1

--RFHPTCHGNNISVSSDRQTARRA-ESFCNAIVFSDRPFRIQEKIRLKFTQTTDGWSGVIRFGFTTQDPNSIQAASLPRYACPDLTNLPGYWAKALPERYAERNNILCYYVRRDGSVCYSVNNEDKGLFFAGVD--TSSPLWGLVDVYGNSTEIKIIPDILSD----------VWGISRDLLQEM---QFSTTS-GANVIFRENRTVAKRRGNEFCKGVTFTSRPLEVDETIFVQVIEVTPRYLGGLAFGVTSCDPQHLNAFID---LPDDSESLMDRP----EYWV-IYKDLSVPEEGDQLTFTINSEGELR-HHVNGVDKGVLIHVDVSQ-RLWLVFDVYGSTQ-AIRILTHNL----PP-------PPAGVTVGDCTICY--DRPTDSAVYPCGHMCLCNKCGQL-LKRQ--RGGMCPICRGAIRDIIKIFK

>Bfl2

---------GKNIELSSDRRTACRT-DSFCNGITFSHRPIRMGEKVHLKMNKVQLGWRGVLKLGFTNTNPCTVDSESLPEYAVPDTSKQARYWALPMMEKYAQKDNMFSFYLAEKGTVHYSINLENKGVFLEGLD--DTKPLWALVDIFGKTEEISIVEAFEKS-------VGNHKGDGDDDSDHSSRISFNDMR-GTNMRLEDKHKVVRRKRNTFWNSLAFTSRPMVTNEMLFVEVLEVNAQYAGAMGFGVTACDPAKLASDTEKTGLPDNADILVMKRSELSEYWK-LSRELKVPSSGDKLSFMITEEGNVH-YDVNGAGSIVLLNVDLNIGPLWALFDVYGSTQ-AIRLLPKQA---------------------ECSMCYKKERPKDSVVYPCAHVCLCHLCAIK-LKKY-IPKAYCPVCSSPVKEVIR---

>Spu

NHLFHT-THGKNIVLSPNRKLATRS-GSFCNAIVFTHRPLRPQDPLHLQLIQSTQGWSGVIRLGFSCHCPDRLKPHMLPKYACPDLTVKPGYWVKALRETLAANGNVLSFFVNSRGEVYYSINGEPYHMFFNGVD--VSKPLWALIDVYGNTTGIRIVPLSPLP------PLPTQLIPQLPLLERL---PFHSVH-GSNIAFSEGLMAAERKSDVFNGGLVFVCRSLKVDEAVFICIKTVNHRYIGHLGIGLTTCDPKGLRDT-T---IPDDAEHVYDRP----EYWV-LTREFEQPAANDVMGVVFNSEGEVHLLKADGTSKCCLMFVDVTI-PLWLYFDVYGTTQ-AIQTLTGTAGPSKPK-------PPRPSKPPECSICF--EAPVNSVFYKCGHTCCCFECANK-M-----RGSCCPICRAVIADVIRMYK

>Hro

PLLFHP-THSTNIQLSSDQRCAKRL-ETFCNGISFSNRSISVGERVFLRFAEVSTSWSG-------PKNNKKHNNNN-------NVIGNNSFYSNNNHNLQQLQRSTTNYQQQQQPQLHC---GGNLIVVTNNIN------------------------------------------IPVKYNKNLNSVALHQVT-GKNVRLSHDKKIASRLADEYCNGYVFMSRHLKPGEKVVIQILSLDNAFTGGVAFGMTSCDVSSLLPN-E---LPDDSDLLLDRK----EYWVVNKDVC-----------------EVK-YGKNGHTVSTLMHVDMAV-QLTAFFDVYGNTQ-AIKLLPSRQKTTLPQKQQQRNISPTSSSSTECSVCF--EKAPDCVLYMCGHMCMCYECALV-VKEY--KDAQCPICRQPIKDIIKIYR

>Aae

PLKFHY-VHGDNIRISRDASVARRY-ESFCKGITFSARPVRVNERVCVKFLDISNNWSGVIRFGFTCNDPATLRG-NLPKYACPDLTNKPGFWAKALNELYCYRNNVLFYYVTASGDVHFGINGEEKGVFITDVD--SRGPLWAVIDVYGNSTAIEFLAVVSSA-------ASMRYNSPAPGLLPL---PFHPIR-GRNIKFSPDRTIATRAETEFCQGYVFAPHPIKIGERLIIQILKTDTMFVGSLALGLTSCDPATLQLS-D---LPDDSDLLLDRP----EYWVVSKDVASTLVRGDELCFSISVKGEVT-ISKNGGAPSVIMHIDQSL-QMWAFLDVYGSTQ-SVRLFSTPL----PT-------PSMGVNPPDCTICF--EKPIDSVLYMCGHMCMCYDCAIKQWRGI--GGGHCPLCRAVIRDVIRTYK

>Hsa1

PLLFHPHTKGSQILMDLSHKAVKRQ-ASFCNAITFSNRPVLIYEQVRLKITKKQCCWSGALRLGFTSKDPSRIHPDSLPKYACPDLVSQSGFWAKALPEEFANEGNIIAFWVDKKGRVFHRINDSAVMLFFSGVR--TADPLWALVDVYGLTRGVQLLPIPQNS-----LNSQHSRALPAQLDGDL---RFHALRAGAHVRILDEQTVARVEHGRDERALVFTSRPVRVAETIFVKVTRSGGARPGALSFGVTTCDPGTLRPA-D---LPFSPEALVDRK----EFWA-VCRVPGPLHSGDILGLVVNADGELH-LSHNGAAAGMQLCVDASQ-PLWMLFGLHGTIT-QIRILPVSL----PE-------SPVT---PECTICY--EHAVDTVIYTCGHMCLCYACGLR-LKKA--LHACCPICRRPIKDIIKTYR

>Hsa2

APRFHAQAKGKNVRLDGHSRRATRR-NSFCNGVTFTQRPIRLYEQVRLRLVAVRPGWSGALRFGFTAHDPSLMSAQDIPKYACPDLVTRPGYWAKALPENLALRDTVLAYWADRHGRVFYSVNDGEPVLFHCGVA--VGGPLWALIDVYGITDEVQLLPCGPRE-------RPRPASSPALLEADL---RFHATR-GPDVSLSADRKVACAPRPDGGRTLVFSERPLRPGESLFVEVGRPGLAAPGALAFGITSCDPGVLRPN-E---LPADPDALLDRK----EYWV-VARAGPVPSGGDALSFTLRPGGDVL-LGINGRPRGRLLCVDTTQ-ALWAFFAVRGGVAGQLRLLPLSP----PV-------SPVF-SPPECTVCF--DGEVDTVIYTCGHMCLCHSCGLR-LKRQ--ARACCPICRRPIKDVIKIYR

>Cin1

RLLFHP-IHGKNITLSHDRKIARRT-SSFCYGVAFSSRPIRINERISIRLETISTMWSGVMRFGVTCVDPNSLNPNDLPRYLCPDLTNKPGFWAKALSDDFAIENNVISFFVNHTGELHFSVNGNEQETFISGINLRSHQPIWMVVDVYGNSTALRIMSSTLDD--------LRQILPPSLLPNNLSRSSFSTQH-GNDIAF-PDPYNAIKGWRYVGGNIVFSGKPYSSGESWFLKVNSQRPTKIPVLGVGITSCNPDFIDME-S---LNGNADDLMDRL----EYWV-MHEVFPAPNVGDFFSITLSSDGCVM-FQVIGGEEQMLFHCDTSL-PLWFIIDMRGDIS-HLISVPAGNDGEILPGASVSTQGASGIAPPECSLCV--DAPANYAIYDCGHVCLCEACSKKLLQME--RFPKCPICRKPIKDVMKLYH

>cin2

MLRFHP-VHGSNVVLSCDRQVASRKPDSFCNGLVFGHRPVKFGEAIHLRFNKVRPDWRGCIRLGFTPYNPARCNSASLPSYAVPDLSRRSRFWAIPIPEKYATKYNIFSFVCKDDGSVSFMINNEGKGIFATGLS--TKISLWPMLDLYGKVEELAIVDDSDSETDGHFGRKTKRSSLSKSSENVV---AFHDTC-GRNVRMDSRRTVAKR-FDSFWNALTFIAKPLRTSDMTFIEIAKVEANYAGALGVGLTNVNPATLKSE-D---LPDNADLLVVSP---GKYWRISRDI--ETEQGDQLGFILEPDGRMV-YSKNGSKHKVLFDKISTKEHNWLICDLYGSTT-TVKLM-------------------------ECLMCFKKDRRRDCTIQPCGHIALCHWCAITCMVKQQLRFIVCPVCGGPVKDAIRILR

>Cel1

PLQFHC-IHGSNVVILKNGRLAKRR-ESFCKGLAFSNRPIEIDENVCLRLCEVGTNWSGVLRFGVTNDDPEMYRDIPVPTFACPDLTTKDGYWAKALPERYSNEGNILHFYVNAHGELFYGINGSQ---------------------------------------------------------------KMHQNS---------------------------------------------------------------------D---RAATPRHLQSLQ-----------------------------------YHQDPHQL-------------------------ARRLL-----------------------------------------------------------------------------------

>Cel2

PLQFHC-IHGSNVVILKNGRLAKRR-ESFCKGLAFSNRPIEIDENVCLRLCEVGTNWSGVLRFGVTNDDPEMYRDIPVPTFACPDLTTKDGYWAKALPERYSNEGNILHFYVNAHGELFYGINGSQKGMFLTGIN--VHRPMWLILDIYGNSVGVEIIPSSASE-------SSMASRTDDSGKRPL---RFHYVK-GCHITLNPSRNIATRDQAEYSQGYVFTERPIKNNEKVMIMISQVQRLYEGGLAFGVTCCDPASIRVA-G---LPDDSSDLVEMP----EYWVGIKDIALQPKANSILSFWITDSGEVK-FEIDSNGARTCLYVDNSL-ELYMYFDVYGSTL-SIKMLTTAM----MS-------PPSFNPPPECTICM--DAPVNSVLYTCGHMCMCFECGRR-LL-T--TKGTCPICRAPVQDVIKTYK

>Dre1

SPRFHPHAKGKNIRLDAHLRRATRK-NSFCNGITFSQRPVRLYEKVRLRLSGVHTGWSGALRFGFTTLDPGELSLTDIPKYACPDLVTRPGYWAKALPAAF---------------------------------------------------------PCLSTS------------------------------------------------------TYIFT---------------------------------------------LSEA------------LMW----------------------------FSRM-----------------------------IAQLL-----------------VPTF--------------------------------------------------------------

>Dre2

PLCFHANTKGSQIVMDKTQRSVRRI-ASFCNAITFTSRPVGVYEQVRLKITKTQGCWSGALRVGFSIVDPSNISSAWLPRFACPDLVSEQGFWGRALPEEHCEEGTILSFWLDNIGRVFYRVNGGSPIFFFSGVP--AGEPVWGIIDIYGLTRGVHLLPSFSSS------PNALNLLLSHQLSDDL---HFHSVH-GSALRLLTEHIAVRYYNRREACALVFTHRPLRCGECVFLKV---SLRSSGFLSYGFTSCNPAHVNSK-H---LPVDPDELLDRK----EFWA-FSSMTSALVGGDIIGFRATAEGEVL-VSHNGGRARREMCVDNSA-PLWMIFHLQTHIK-QISILSLGT----AF-------GSLSSESPECLICC--DRPVDSVLYACGHMCVCSDCGVK-LTET--SNPSCPVCRSPIRDIIKIYR

>Dre3

-------------------------------------------------ITKKQCCWSGALRLGFTSKDPSRINPDSLPKYACPDLVSQSGFWAKALPEEFANEGNLISFWVDKKGRVFYRINDSSQE------------------------------PIPQNS-----MNSQQSSLLPHTLECDI---RFHQLR-GAHIKILNEQTVARSEHNREERTLVFTDRPLRIGETIFIKVIKSSPARFGSLSYGVTSCDPAVLRPS-D---LPYNPEALVDRK----EFWA-VCRVPTALQSADILGFLVTQEGEVI-LSHNGTNVGMQVCVDNSR-PLWMFFGLHGAVT-QLRILPINL----PK-------SPTF---PECSICY--ENTVDTVIYTCGHMCLCYTCGLR-LKKM--ANASCPICRRAIKDIIKTYR

>Dre4

SPRFHPHAKGKNIRLDAHLRRATRK-NSFCNGITFSQRPVRLYEKVRLRLSGVHTGWSGALRFGFTTLDPGELSLTDIPKYACPDLVTRPGYWAKALPERLAMRDNVLAFWADRHGRVFYSINDGEPILFHCGLS--VGCPVWAIIDIYGITQEVTLLPCCSTSSTPSSSVQRPARSLSLPLDVDL---HFHPVR-GPDVVLSNDRTVACTHFLDSSRTLMFSDRPVRVGETLYLEVGHLGLPYFGALLFGMTSCDPGTLSAG-E---LPADPELLLDRK----EYWV-VYRGFPVPTAGDVLSFTFLANGEVH-HGVNGVARGRLLCVDSSQ-VLWAFFTLHGAVN-RLRILPLSP----PV-------SPSFSSSDECTVCF--DQEVDTVIYTCGHMCLCNDCGQR-LKRQ--INACCPICRRPIKDVIKTYR

>Gga1

PLLFHPHTKGSQIVMDTTQKAVKRQ-ASFCNAITFSNRPIVIYEQVRLKITKKQCCWSGALRLGFTSKDPSRINPDTLPKYACPDLVSQSGFWAKALPEEFANEGNIIAFWVDKKGRVFYRVNDSAAMLFFSGVR--TAEPLWALIDVYGLTRGVQLLPIPQNS-----LNSQHSHLLPSQLESDL---RFHHLR-GAHVKILDDQTVARLEHAREERTLVFTSRPLRINETIFVKINKSNAARTGTLSYGVTSCDPSTLRPS-D---LPYNPESLVDRK----EFWA-VCRVLVPLQSGDILGFMVNSDGGLH-LSHNGASTGMQVCVDSSQ-PLWMFFGLHGAVM-QIRVLPVSL----PE-------SPIS---PECTICY--ENMVDTVIYSCGHMCLCYSCGLK-LKKM--ANACCPICRRAIKDIIKTYR

>Gga2

PLRFHSHAKGKNVRLDTHSRRATRR-NSFCNGITFTNRPIHLYEKVRLKLVAVHHGWSGALRFGFTTHDPSQMSSDDIPKYACPDLVTRPGYWAKALPERFAVRDNVLAFWVDRHGRVFYSINDEEPILFHCGIK--VSGPLWALIDVYGITHEVQILPCCPNR-------RPRVQGVPAFLDTDL---RFHPTH-GPDITFSQDRMVAWTNWQESNRTLVFSDRPLHIGESLFVEVGHLGMPYYGAFSFGITSCDPSTLRTN-E---LPADPDLLLDRK----EYWV-VYRAFPVLNGGDILSFTVLPNGEVH-HGVNGASRGMLMCVDTSQ-SLWVFFAIHGVIN-QLKILPLSP----PI-------SPIF-PPPECTVCF--DNEVDTVIYTCGHMCLCNTCGLK-LKKQ--LNACCPICRRVIKDVIKIYR

>Gga3

PLRFHSHAKGKNVRLDTHSRRATRR-NSFCNGITFTNRPIHLYEKVRLKLVAVHHGWSGALRFGFTTHDPSQMSSDDIPKYACPDLVTRPGYWAKALPERFAVRDNVLAFWVDRHGRVFYSINDEEPILFHCGIK--VSGPLWALIDVYGITHEVQILPCCPNR-------RPRVQGVPAFLDTDL---RFHPTH-GPDITFSQDRMVAWTNWQESNRTLVFSDRPLHIGESLFVEVGHLGMPYYGAFSFGITSCDPSTLRTN-E---LPADPDLLLDRK----EYWV-VYRAFPVLNGGDILSFTVLPNGEVH-HGVNGASRGMLMCVDTSQ-SLWVFFAIHGVIN-QLKILPLSP----PI-------SPIF-PPPECTVCF--DNEVDTVIYTCGHMCLCNTCGLK-LKKQ--LNACCPICRRVIKDVIKIYR

>Xtr1

PLLFHPHAKGSQIIMDTSQKAVKRQ-ASFCNAITFSNRPVVIHEQVRLKITKKQCCWSGALRLGFTSKDPSRINPDTLPKYACPDLVSQSGFWAKALPEEFANEGNIIAFWVDKKGRVFYRVNDFGSMLFFSGVR--ITEPLWALIDVYGLTRGVELLPIPQNS-----LNSQHSHLLPSQLESDL---RFHQLR-GAHIKTLDDQTVARLEHAREERTLVFTNRPLRINETIFVKINKSNSSRSGTLSYGVTSCDPSTLRPS-D---LPYNPESLVDRK----EFWA-VCRVLVPLQSGDILGFVINGEGELH-LSHNGTSTGMQVCVDCSQ-PLWMFFGLHGTIM-QIRILPVSM----PE-------SPLS---PECTICY--ENLVDTVIYSCGHMCLCYTCGLK-LKKM--NNACCPICRRLIKDIIKTYR

>Xtr2

SPRFHCQAKGKNIRLDTYGRKAIRR-NSFCNGITFTSRPIHLYEKVRLKLVSVHHGWSGALRFGFTIHDPAQMKLEDIPKYACPDLVTRPGYWAKALPERFAQRDNILAFWVDRHGRVFYSVNDEEPVLFHCGVK--VSSPLWALIDVYGITQEVQLLPCCPNR-------RQRAQGIPTLLDMEL---HFHPTR-GADITLSPDRTVAFTNWQESNRTIVFTERPVHIGETLFTETSQLPLPYYGSLSFGITSCDPSTLRTY-D---LPANPDYLLDRK----EYWV-VHQGLYTLGNWDIHSFSLLPNGEVH-HGINGNNRGMLMCVDTSQ-PLWMFFSVQGIIN-QIKIMPLSP----PV-------SPVF-APPECAVCF--DNEVETVIYTCGHMCLCSSCGLK-LKRQ—VNACCPICRRVIKDVIKTYR

**NICASTRIN**

>Cel

VFRTLFIGEGNACYRTFNKTHEFGCQANRENENGLIVRIDKQEDFKNLDSCWNSFYPKYSGKYWALLPVNLIRRDTISQLKS-SKCLSGIVLASHDAECPNAASDYYLQDKNEEYCERKINSRGAITRDGLDW-RIQMVFICYSMFNPYCGMSFRLANMAAGNSEICYRRCGAMHSDNIFAFPTPDSFGMIPEISV--GEVSVLTSIISVLAAARSMFAFFNGESLDYIGSGAAAYQMELDYILEVQQIHVDGERYQVPPASWHSFASVLLAPYGKE-YEYQ-RVNSILDIEAVSTAILAAAADYVDK---KLITTIFDCLITSNFWFDCTIFALGCLKSAIVKKVMVSPAVNTRY----STWMESVYIIESVNLYLMIAV-

>Ddi

MYTSL---NSYPCTRIMTLNGQIGCSSSHGGDSGILYLIDSDESYHNY------FSYNQQKDIIVVFDSNYFNKTLVLEMYS-KKKMNGALVYSPEDQYPIKQFGLYPD----------SNLNWNPNGDGFNF-PFPMFALTINRDGPAYGAELDSFMQGAINAETCLRRCEPVGGQSIWSSFSEDATAFFRDLAT--GTDQSGYALTVLLSMLNTLFAMWNSERWGYVGSTNFVNDLNIYAIIEFNQI-----DIYLPPCSSMSFITLVITDHDYQ-YNNP-YFGDEQDLFDMVQVFSKSIDLLADD---LFIREINVCLTQ---SITCATYLTQCLYSNTHYHNAISLAFNTSY----PVFVESNW--DYTTVRLFLVSG

>Dre

IYVELN--KTAPCVRLLNATHQIGCQSSISGDTGVVHVLESESDLD-----W-ILSAGPHPPYMVIMETAFFNRSVMLSMKN-SSRVAGVAVFSPHTTCPNQNTGVYSA--GSEFANC-NGTVWNPLGNGLDF-TFPVFALCYEDHNPLCAMQLFSHMHAVTDTVTCMRRCDPLSDYNVWTSIRPDGRSFFWEEAP--AAEGTVSAIVTLLAAIHALFTFFQGEAFDYIGSSRMVFDMNVHSMLEIGQVHSD--PVSLPPSSFQRFLGLVLTDHEKA-FNNR-YYESMYDLAEVATLVARSLYTQADA---TMVSQLLYGFLV---QKNNLANLTGCVRASVRLSKAVSPAFSSDY----STWTESRW--KSIRARIFLGVG

>Hro

IVYNVKRDDFSICMKFLNSTDQMGCSGDYGGNTGVLMTVSSEKDLD-----W-LRQPGHEKNYIILMGLDFLESRIIEEFID-IEKIVGVLIFSPDLSSPNYEQSIYT-----------RNFDWNMVGNGLDI-PFPMALLCYLNHNPLCAVKMNMAMNADTSSRVCLRRCDTLAGYSYLASLTPDALSLFETRSP--GAVSTVTGLVTLYAIASILFALFNGESLTYMGSSRIVHDLKNKTLRDVGRGQFTGPPVPLPPSSIHAFLGLVVANHDKS-YDNL-FHNSFLDMASVATTVACVVFREVDQ---KMIANLLYCFTQ---DPECFAHVIGCKSLYINVIRTGSPLLSVDF----PTWTLSTFNDKHISIKVFLLLG

>Ppi

IYTKVNF-GDTICFRMNNFNSQTGCQTGRDGDNGILYPVQSESGLE-------SDLQSLDIPIIAVLNASLFTNTTITALKAYSQTKGVLIM-SMEKSCPGNGYNIGESASDVVGRQCLNSHKWNPLGNWLDL-PFGVSLIAFQNLGRRWGGEIKVFMNAAQDTKTCIRRCDPISGVSTHVFMEPDAAAFFKSLSY--GGEASAASIAVVLGIIGTLVSFLQAESWDYSGSQRLAYDLDVKLVIEIGPI-------------------------------------------------------------------------------------------------------------------------------------

>Mbr

------------CTRLINVTHEIGCTSPEDGAVGIFFKIESADELDD-------FRSATESAYIVLLEQNMFAQSAVLRELGRMDKVKGAFV--------------------------SGQTNWNPYGGGFDFGSKAIYYLRYAALNPVWGARLKNFMYGVRDTDLCLRRCDPLSGQNVWGSTRLDAFSFFHDLSY--GANSDASAVVSLIGAARLLFNLFNGESFGYIGSSRLGFDLNIKGYLELSQVH----SYKLPPASLRGFLGVVVTAYDSTGFANK-YHGSHLDLCDTAVLTAKTLVAMADSAFCDFLQEMFDCVAR---NQTCFFNMLSCMNTTAYVSYIMSPAFDPRW----STWTESVW--GAISADMF----

>Tad

VYRDIT--SFNYCVRYTNKNHSIGCTSKYWRNVGVLHLVESFSDMN-----K-LATSKLDIDYVPLMTPKLFNLDNVNFLMS-KRKIAGIII--DDKEIP---DGMYVD--NSQYQSC-KRIEWNPHGSGMSF-DFPIFRVCYLKHNPLCAVQMGDFMFTPSNTEVCMRRCDPLGDSNVWSTLYPDSISQFYNFAP--GTDNDASGFITLLSAAYALFAFFQGESWDYIGSSRM----NIGQFLELRQVHFD--PSSLPPASVQRFLGLVLTDHKKN-FTNA-YYNSHYDLTRLATTVARTLYMLADE---DMTSELLHCFLQ---NASCMMYFTGCYRGTVYYSPAVSPAFSKNY----STWAESRW--QSVDLKMF----

>Spu

IYYDIK--NFGPCVRRFNATHQIGCTSEFNGNNGVLHLIEKVADLD-----W-LLKNGTYSPYIAVMSPQMFTLDNVQRLLD-SKKVNGIMVFSPDKSCPNDNYGMYAG--NPEYSNC-KKVTWNSVGNGMDL-GIPIFALCYEEHNPQCAGEMYDFMFGAKDTPTCMWRCDPLGDWNVWATLLPDIADLFYQALPTQGAENTVSGFVALLAAAQAIFTFFQGESYDYIGSSRMVYDMTVAAVLEVRQLHID--PLSLPPASFQQFLGVVITDHERE-YANK-YYNSRLDLANLATTIARGLYLYADV---NLVNDLLYCFNQ---RPNCLAYYLECVKSSRHLTPAISPAFSTVY----STWTESQW--SSYRIRAFFSVG

>Lgi

IYIDLQ--TQNACFRVLNATHQVGCSSKSGGNVGTLHYLESEEDYN-----W-VLKSGPHTPYIVLITSLDFRKDNVERLVN-TKRVNGILVFSADKSCPNDNYGIYSN--SSDYQHC-KKVKWNAAGDGLDYHDFPMFAICYRKFNPICAAQLNDRMDAAKDTITCIRRCDAMGDKNIVATTKMDSFSMFEYEYQ--NSDTTVTGIVALLSAAKALFTFFQGEAFDYIGSSRMVYEMSISHIIELAQLHTD--PISLPPASVQRFLAIVLTDHKQQ-YTNK-FYNSRLDLTHVSTVLAQFLYKLTDE---LEIAHLLYCFLE---NPVCLAQYLGCIKSTAILSKAQSPAFSEEY----STWTESVW--SVFKVRVFFSLG

>Hma

IYKSI-K-SYSPCILLTNATHQVGCTSNMGGNRGILHVVESDDDVD-----W-LIDKGTNQPYIPLLNSSFFNENVMNKLMN-SKKISGVLVFSPDSKCPLDQFGAYSN--DKENGNC-QKIKWNPTGNDMYY-GIPIFALCYQKHNPLCAIE-------------------------------------------------------------------------------------------------------------------------------------------------------------------------------------------------------------------------------------

>Nve

IYEDIK--GVIPCVLLTNATHQIGCTSTIGGHVGVLHFVQSQDDVD-----W-LLNKGKHAPYIPILTSEMFKGDVLSSLAKKNVINGALVIFSPDYSCPNDNHGLYRG--NADYSGC-KKQIWNTVGNAMYY-GIPIFLLCYEKYNPLCSAQLKDFMFAAKDTPTCMRKCDPLGDLNIWSTLYPDSSSFFHDLVY--GADNDASGVIALLAAAKALFVLFHGESWDYIGSSRMVYEMNIKYFIELNQVHTD--PVSLPPASFQRFLGVVLTDHEAN-FTNK-YYNSRFDVTKVANTIANTLYTLADP---FLVGHLAYCSFY---RGNCMLYFTGCVNGSVYFSKAESPAFSTQY----STWAESTW-NADLSVRIFAEIS

>Bfl

IYVNIT--AQAPCVRLMNATHQIGCTSAVSGNVGVLHYCAEQSDVD-----W-LIQSAKNKPYMPLMVPRLFTYENVQGLRA-SGKISGILVFSPVHKCPNDGYGMYSD--HPEYAHC-KNTTWNPLGSGLDY-DFPMSLLCHNRFNPLCAVQIKDRMDGAKDSITCIRRCDALGDKNVWGTLRPDTWSLFHNVGG--VGANDATGFIALLAAAEALFVFFQGETFDYIGSSRMVYDMNIQYFLEVSQVHSD--PVSLPPASLQRFLGIVLSDHMAA-YSNN-YFMSRLDLTKVATVIARTLYQLASS---STINHLLYCFLQ---HANCLTFYLGCMRAPVQFSTAVSPAFSNEY----STWTESRW--TVLNAKIFLSVG

>Xtr

--------------------------------------------------------------------------------------------------------------------------------------------------------------------------CDTIIVHNVWSSLKPDSHSFFWNLAP--GADSTVSGFVTLLAAAEALFVLFQGEVYDYIGSSRMVYDMNIHSFVELNQVHTD--PISLPPASLQRFLGVVLTDHRTA-YSNS-FCRAVARLAGVATVLANSLYRLADP---NTVTQMLYGFLK---MSNNFANLTGCVRSATHSHVAESPAFSTEY----STWTESRW--KEIKARIFLVVG

>Gga

IYIPLN--KTAPCVRLLNATHQIGCQSSISGDTGVIHVVEKEEDLN-----W-VLADGPHPPYMILLDGNLFNRKVMQQLKG-TSRVSGLAVFSPGLKCPNDGFGVYSKDYGPQYAHC-NRTVWNPVGSGLDF-DFPIFLLCYQDHNPLCAMQLFSHMHAVTSTVTCMRRCDPLLDYNVWSTLHPDSHSFFWNIAP--GAESAVSSFVTHLAAAEALFTFFQGETFDYIGSSRMVYDMNIHSFVELNQVHTD--PVSLPPSSFQRFLGVVLSDHQAS-FQNR-YYQSMYDLAEVATVVARALYRLADP---KTITQMLYGFLI---KMNNLANLTGCVRSTVRLSKALSPAFSTEY----STWTESRW--KEIRARIFLVVG

>Aae

MYTPI---SGAHCFRRLNGTHVTGCSSKLGGSVGVLHFIRSKADID-----F-VVRKHPAPPYAPVLAPHLFTRENILRLRNQGGRHVSAVVFSQESRCPNQ-FSRFAGREEETCDVERPEGSWNPWGTGLDF-PFPIYYVCFEKFNSLCSIEVNAFMSAAVDTRVCLTRCDPLQGKNVFATLFPDTTTMFDGIGL--GAMDSLVPFTVLVSVAHFLFMFFNGESYDYIGSQRFVYDLNIDLMIDLGTMASEL-PVALPPVSSHSFLTVVLTSVPGN----R-YYHSIYDLRNVSSLVGMAIYELINS---VLIDEFLYCFLL---SADCLGLLVGCRWTTQNFTQALSPAFSHRY----STWTESTW--REFSARIFLSIG

>Aqu

---------------------------------------------------M-V---GPRPP------------QALPQLRP----------------------------------------------------------------------------------------C--------------DSSAFFPYLAY--GANNEMAGIVTLLTIAKLLFTFFQGESYDYIGSSRMVYDMKIKAFIELSQVHHN--DQQLPPASAQMFLVVIVTDFDQQ-YNNK-FFGSRYDLANLAGSIARAALTLSDT---ELIVQLLHCFLQ---NSDCLSNFTGCTKAYTHMHTATSPAFSDYY----STWAESRW--TSTAMRIYLIAG

>Ath

MYVAV---DGFPCVRLLNLSGEIGCSNP--GINKVVAPIIKLKDVK--------DLVQPHTILVTADEMEDFFTRVSTDLSF-ASKIGGVLVFSPDKRFPQAQFSPYEN----------VEYKWNSAASSINY-NFPVYLL-ILSKKAEFNMVMETTKAGTHNSEACLQECLPLGGYSVWSSLPPDTASFFRDKSF--GADSPISGLVALLGAVDALFLVLTGETWGYLGSRRFLHEL---TVLEIGSVHKT--RVSIPPSSLMAFMAVVLEDFDTN-FVNK-FYHSHLDVVAAASVVARTLYILANA---SFVEELLTCLLACEPGLSCLADKTSCVVSTTRYVPAYSTRLSDSMGMVDPVWTESNW--DTLRVHVYLVAG

>Vca

MYVELT--DNRACVKLMTSSGPQGCEAP--NEELVTAPLVHRKDL------W-IPYKGKRVVVVPAAEVSTLLSLLLSDSEL-HSRVAGVLVYSTAAKFPSAEYALYDN----------RSYPWNPFGAGFFF-GFPMYMLSYNADN-----RFEVPMSAKGNSVQCITECLPVGSYGVWTALPADSNALFHELSR--GAGTSVSGLISALVALTLLFVALPGEAFDYMGSKRLLYEMLIDQVVEVGQIHTQKGDKYIPPSSLMAFLGLLVADFDTA-FKNP-YYQSEFDIVDASLLLARTLHSLALFTDRALAINLVYCLMTQSPGLQCLGLITACRNTTTSFVPAYSTRLAAYQWPPDPLWAESNWPEKTPKLTIFLVVG

>Hsa5

------------MLKPINTTGTL------------------------------------------------------------------------------------------------------------------------------------------------------------------DSRSFFWNVAP--GAESAVASFVTQLAAAEALFVFFQG-------------------------VHTD--PVSLPPSSLQRFLGVVLADHSGA-FHNK-YYQSIYDLADVATVLGRALYELADP---QTVTRLLYGFLI---KANNLANLTG----------------------------------------------

>Hsa4

IYIPLN--KTAPCVRLLNATHQIGCQSSISGDTGVIHVVEKEEDLQ-----W-VLTDGPNPPYMVLLESKHFTRDLMEKLKGRTSRIAGLAVFSPSVQCPNDGFGVYSNSYGPEFAHC-REIQWNSLGNGLDF-SFPIFLLCYQDH--------------------------------------------------------------------------------------------------------------------------------------------------------------------------------------------------------------------------------------------

>Hsa2

IYIPLN--KTAPCVRLLNATHQIGCQSSISGDTGVIHVVEKEEDLQ-----W-VLTDGPNPPYMVLLESKHFTRDLMEKLKGRTSRIAGLAVFSPSVQCPNDGFGVYSNSYGPEFAHC-REIQWNSLGNGLDF-SFPIFLL---------------------------------------------------------------------------------QETFDYIGSSRMVYDMNVDSFVELGQVHTD--PVS-----------------------NQ----------------------------------------------------------------------------------------------------

>Hsa1

IYIPL-N-KTAPCVRLLNATHQIGCQSSISGDTGVIHVVEKEEDLQ-----W-VLTDGPNPPYMVLLESKHFTRDLMEKLKGRTSRIAGLAVFSPSVQCPNDGFGVYSNSYGPEFAHC-REIQWNSLGNGLDF-SFPIFLLCYQDHNPLCAMQLFSHMHAVISTATCMRRCDPLSDYNVWSMLKPDSRSFFWNVAP--GAESAVASFVTQLAAAEALFVFFQGETFDYIGSSRMVYDMNVDSFVELGQVHTD--PVSLPPSSLQRFLGVVLADHSGA-FHNK-YYQSIYDLADVATVLGRALYELADP---QTVTRLLYGFLI---KANNLANLTGCVRSTARLARALSPAFSTEY----STWTESRW--KDIRARIFLTVG

>Pra

APEHLVT-TGGECVRIFHSGGSVGCRGLSDSDMAPLYPITSSDELQSFVEGQ-AQQQDASSKYVLVMAESLLSYDAIQAGLD---RIGGLFVASYDVVTP-QGDGTVDGALNPFAS---DKTKWNPTGTGLLL-PFPVVMLAQINMKNYYFGPENMNSIKCLNFTNIYGKCDPIGGQSSWAMRGNDATSLSHVLAP--GANTGASGLVALLAAAHALFAAFQAEKFGFVGSRKFLSDLNISYAIAVDQVHVN--PHALPPTPLSSFVSAVLAGYDDS-YTTEGTYNSRHDVVQAAQVLAESVFTLADT---WLVESMLSCIAT---DWSCLASAMVCASKSAFHHEASSPGVNSSM----PIWTEPQW-ASDIGSYSFLAIG

>Pso

APEHLVT-TGGECVRLFHSGGSVGCRSLSAADMAPLYPISSADELQSFVAGQ-AQQQDAASKYVLVMAESLLSYDAISSGID---RIGGLFVGSFDAATP-QGDGTVDGALNPFAS---DKTKWNPTGNGLSL-PFPVVMLAEINLKNYYFGPEEMNSLKCLNFTNIYGNCDPIGGQSSWAMRGNDATSVSHVLAP--GANTGASGVVALLAAAHALFAAFQAEKFGFVGSRKFLSDLNISYAIAVDQVHVN--PHALPPTPLTSFVGAVLAGYDES-YTSKRTYNSRHDVVQAAQILAESVFTLADT---WLVESMLSCISM---DWNCLASAMVCADKSAYHHEASSPGVNSSM----PIWAEPQW-ASDIGSYSFLAVG

**NOTCH**

>Aqu

CPHANCSSLANNSICDLHCFTPACGYDSSDCTFNGNRTWSQCPFTD-CRSSYKDGVCDQKCNNAPCLYDGNDCLFSLPSCPQQNITRCSGLVGNGVCDLYCNTTQCPFDTVDCAK---QVYLPGTLIMILFSLPSVQFSHIEPFRRSLGRLINAEVEVLSYSNISKEEASMYIDN-TRIDAPLSY----WKVTMLINIQYCVQA---CPTSIEQVIRIVRASS--GSVKDDFGIEAIEGSPPPPNNPASTSIIVIIVSVSILIVVLVVGVL--GKRVRDNGGIWHVDGRNNSGSNTQDTTDLRSSSPECVADSFSQTDRRGYI----WQPIKVSVDSVAVDLRRWTPLHCEAVCFSGDNTLLMRDHS--------LDAQGPGGFTPLMIAIVSQEKKNTVRTDSSSDQSENCAPSYSHPGAVDGNITNDYGQTALHLAAKLGREDYIHILLSAKADPNIQDMWGQTALHVAIGAATPGAFKALLAYPKTSMELKSMGGVTPLIMCVKMANHPMLQQLITKNVDIAATDNEGRTAVHWAAMINNIEALKMLIKQGPDKDAPNGRGETALYLACREGATECVRYLLECFANNTLMDMLDKSPLQIAYERQHADVVELLKQAN----IPPPPSSYNHKVHVSS---------------NPMFSRANTIKMEPGLPSP----HIPPSYQ--FPSPPKEVGEYCYPKPHTVDPTADIPSPEDQSVETSSPSYLPQHQSTVLRHAYDYSAATYNP

>Tad

CSIPSCKKVFNNSVCNPECNTYECAWDGFDCSLYL-SPWSNCTRKDGCWKLFHDGRCDQECNSESCLYDGWDCNQAVASCPDNYNQYCEANYGNGYCDKGCNSRECMFDGLDCNDQT-PKYSKGVSYMILATNLESFTNSSKSFLRTLSIAIRTTIEIAKVDGKDDVKEVKVGATSFILNRERRASTVGLRVGIILDNRGC---TGSCFKKTNEAAKFLAALAISSILNLPFKILSLSDEIIPIQDNPLLEWILILSGAAVFIIFLVVGVLQTRKRKETRG-IWFPFGSKLAGPNSNMNNGAYGHKQQMRQEYKNCREVPDWNDGETPPRKRFHYNINHPDDREWNSLHRNESN----PKFLPDQNAKELLSYVEVNAKGKGGITPLMQ--IVRRKSNKKKDQENNDQNSATVMDYLIKSGADKDAQDDNQMTALHFASIERNEIATAKLLEHGANPNIKESTGRTPLHIAITSKAETIFKILAHDSRTDLIATDKTGSTPLIYAARYGMTNIVKKLLELPVKVDTVDNKCRNALHWAASTEHSRIVQILLEHKVAIDAQDTYGETALFKAAKDGRIENVKALLGRNADESIEDIHLRTPYDVAKEKQHRQVAELIRQHQ-SRKVPIPRMTPSTTTNYAKDAQKSQQQTQVQQQQLHQQQQQLHQQQRQQQQQQQQQQQQQQQQQQQYLTPPSQYPNGETPPQCTTTTTTNLNSQSNINYLTPPPESPESWSTSSPLSQSDGSEAMPSP

>Spu1

CSQ-ECLQRKGNSICDDHCNTHLCEWDGKDCSLNL-DPWENCTANIPCWNYFGDGKCDRECNTQGCLFDGFDCA-KPEECMD--NKFCLERYGNGFCDEQCNNIGCLYDGLDCEE--IPEYAHGVLIVYVIIDYQEMLNRSQMFLQDMSVVLNTVVLFAPTADNQNKIEPWESDPRTVEKREVNSSDDGSKCFLLLDNHKCHIDPGNCFEEAADAAAYLAALVTNELLPISLPIVGVGSET-PSSGPGLVNVLIALAGAFVLAALLV----IARKRLRTNG-TWFPSNFTRECPIGNESP--PSTICSSIDAGEDAMNALD------RGAKRQRISLYGQMDSQWTTLTSNRLP---HPSMALTPPR-DQEPSLIIN-KGPDGLTPLMLAVMRST---TVTSDQEEEANAHFIEDLITRGADINNRTETRGETALHLAARFNLPVAARKLLEYSSNTNAEDNMGETPLHAAVRADAIEVFRLLIQNRSTQIDAKTKAGFTPMIIAVRLVVDNMVEELELAGADIAATDNDGRSALMWAAALNHLPALEFLLKKGSNKDLQDNQEQTPLFLAAKEGHVDAVKMLLDHGANREITDHLDMSPREIANRMRLHNVVRLLDTYS-MMSPMDMHNGEANGYS---NGAKPKKGTKNGD--DARRGESKRKGKPPPMPSPPDSVESPLGYNRTLLNAPKLSNGQHRDAMSEKTSFSNQHSHNGDPFPTPSPESPSKWSNPSPISASDWSEAISSP

>Lgi1

CEQYGCEAKANNGKCDNECNMHACDYDNKECSYGM-KPWKECSGMRDCWKVFKDGTCNPQCNNEECLYDGFDCQTKLKECNPIYDSYCKKYYGNGHCDQGCDTEECEWDGLDC--DKPERLAEGTLVIIVLVEPEAFRNMSKEFLRKLGHLLRAVVRVLKDKNGSPMIYPWQQKEGVREKRALLQ---GTKVYLEIDNRGCYKYTDECFDNTDNAAQFVAAALQRNDADLGMPVGKIESLGKEPTVDSPITFVYIVVGAILLITLVVIIVI---SRKRERAVTWFPEGFNRRGPDGEEMKDVQKGSHHSQLDKMDD--RHGWGDEEEPKAKRQRID----DSRQWTQKHMEAANVTNPSILTLTPPQGDDPIDTKINARGPGGMTPLMLASLRGNTLDESGSAEGDNRAADVINNLLFQGASINLQTDRTGETSLHLAARYARADAAKVLLDAGADCNALDSTGRTPLHTAVAADAQGVFQILLRNRSTNLNAKMYDGTTPLILAARLAIEDMVEDLINADADINAFDNNGKTALHWSAAVNNVEATESLLERNANRDAQDNKDETPLFLAAREGSFEAAKALLNHYANREITDHLECLPREVADERHHHDIVQLLDTYKVNSPPSDLPFMQHIHMKQKPKARKNNKPKENGPISKPRKKKTVQNTQSLGVISPLES-QSPQGY---FPTPPSHHSDS-------TPPQHQMFLPD--HFPTPSPDSPGQWSSSSPHSHSDWSEGISSP

>Cin

CLDPTCAALKGNGECDAKCNTHGCEYDGGDCSYGNTDPWAKCPASLQCYNYFHDGACDEACNNADCLFDGFDCETPHEACPADYQDYCDKNYKNNKCDSGCNTFGCGWDGLDCDKDHPQQPAAGELIMIVLIPPEELISRQAEFLRFIGTVLHSVVEFKLTTSGQPYIEAISSQLSKTHTRLRRANTFFSKAHLLVNSQKCKQTSSECIDTTDNAASVISARASTGTLEAEFPINTVASTTNPTPPPQD--YTYIVGGGVV-LVIVIIGVLTHRKRKRETSTLWAPEGFQRREPIGQDDLGLNGSIHPGELTQLDTAGTPFWETSLPQKSNHYHVQFLPNDNRKWTPQHLEAADRAGSACTDLTPPPIDVDED--VNARGPDGVTPLMVASIRGGGVDGISDDESGDAGISMIAGLLLQGASLSAQTDRSGETALHLAARYARADAAKRLLDAGADANMKDHSGRTPLHSAVAADAQGVFQILLRNRATDLDARTNDGTTPMILASRLAVEGMVEELISANADVNAVDDHGKSALHWAAAVNNVDAVSTLLRAGCNRDAQTEREETPLFLAAKEGSYEAIRILLDHYANRDITDHMDRLPRDIAQERLHTDIVQLLDEYNLVRSAMMSRDPYTSQMKQHTTGKKPRRQSNRADGKPTSSQGPPSSVTPNMAIHPITHVESTL-----YPTPPSAHTMSPSSGSENLSPPGPLPPAPKHSYETPSPDSPDPWSSSSPSN-SDWSEGIRSP

>Aae

CVKKGCREKQGNHRCDEECNTYACDFDGNDCSLGI-NPWLNCTASINCWEVFQDGICNEDCNNAQCLFDGRDCEKKLQPCNPVYDAYCQKHYANGFCDYGCNNAECNWDGLDCENDRPAQLAEGAISIILLMDMETFKKNSVQFLRDLGHQLRTTVRIKKNNAGMEMIFPWKGGT-RKHHVVYTERQTGIQVFLELDNRKCIGMGAECFGTASEAAEFLAATASKHSLK-QFPIYQVKGVNPDDDGLPETNTNYVLLGFFILLLFAMCGVLVAN-RKRAHGVTWFPEGFSRRRPDGQEMRNLNPSMVDMANGHMG---NSQWCSDDEPQPKRLRGI---YEPRVWSQQHFEAADIRDHPAM-MTPPTHDGGHN--VDVRGPCGMTPLMVAAVRGGGIDSGEDIDSDDSTAQIISELVAQGAELNATMDKTGETSLHLAARYARADAAKRLLDAGADANSQDNTGRTPLHAAVAADAMGVFQILLRNRATNLNARMHDGTTPLILAARLAIEGMVEDLITADADINAADNSGKTALHWAAAVNNVDAVNILLTHGANRDAQDDKDETPLFLAAREGSFEACKALLDNFANRDITDHMDRLPRDVASERIHQDIVRLLDEH-VPRSTIMSPTTHPHMITHPTKQNKQKKKMNGAVGGNDGSIRRKAVAKLSSVESPLANIPSPYDTVS-YLTPPSQHSGG-------VTPQHLVQTLD--SYPTPSPESPGHWSSSSPHSTSDWSEGVQSP

>Hro1

CAINNCSAKAGNGICDKECDYAECNYDGYDCSYK-SHIYHSCSAVRSCNKLFQNGVCDEACMDESCLYDGMDCLEPKRTCDLLYESYCSNHYADGFCDQGCNTAECGWDGMDCENSLSKNIAEGTLILTLVATPDYFRHVASKFLRSLGLLLGNTPFLIKDESRKELIHPCAIVP----------NVKSNTTFMLFSIQATRRKADDI--AGETASEFIAQSMRKGW-KPIVPIYSVSSHRDGGETSQQITFIILIVCSVALITVLGFVLYKSQ-KKIARGSTWFPVGFKRCVP----SLEMKNIKCIL------------------IGQTSFQTEPQQQ--RKWSERHLMAAGITQKNAIVPTPPSQDGDQLGLVDARGPDGYTALMLASCKRSVPDDSTGSTDDDDGAAMIFSLINQGAAVNATTHRTGETPLHLAARHSKTEAAKNLLEAGGDPNAQDCNGRTPLHSSVAADSRGVFKVLLRHRATKINAQTADGTTPLMLAVKLCLEDVVEELIEADADINVADNNGKTALHWAASVNNHKAVQILLEKGANKDAQDLQDETPFFAACREGSVETVKLLLKYKVAIDITDHLDQLPQTMARKGMFHEIVRLIDEH-----MLLQSSTSTPLLK---SINAERRPNNESDAP--SKKKSSKKKSESQSLLMLNARLNERNFNRQ------------------LTTQQQLNTTTYGGFISNINSNQQQLTHLSVPVDVNYNNV----

>Bfl

CPLDSCASKARDGHCDEECNIHECDWDGTDCALG-NHPWANCTSSLQCWNYFADGNCDQQCNTEECFYDGRDCENPHPTCNPVYDTYCGNFFGDGNCDSGCNNMECGYDGGDCDEEYPPVTAKGYLVMIVAAPPSVLLNNSVPFLRMLSIILRSSMSFKIDDEGNPMVYPWYGEEVGRYRRATESEVQGSIVYLEMDNSKCYRAHDDCFETAKEAADFLAALFLHDGLQLSYPVKSLYSGEDAPPTTQPFNPLYVVVAVGILLVMVGLGVLMTTKRRRESGQLWYPEGFRRRDPVGQE-VGMKSFTPPQGATANVDDNDNEWEDDMVPEAKRMKTQPGGDDHRQWTQQHLHAADIRNPACLALTPPQTEGEADPCVNVRGPDGLTPLMLASFRGGGLEYVEDQEDEDESANVITDLLMQGANINAQTDRTGETSLHLAARYARADRAKRLLDAGADANARDNTGRTPLHAAIASDAQGVFQILLRNRATDLDARTNDGTTPLILAARLAVEGVVEELLNSHADANACDDNGKSALHWAAATNNEEAVISLLSHGVNKDVQDNKEETPLFLAAREGSFQAAKILLDHYANRDITDHMDRLPRDIAQERMHSDIVKLLDEYNLVRSAANCQSNFHHFMNGPATQQKRAKPKRPNKGMDASKMRRKKKMEDSTALSPDGSLGSPHGYNHGLPTPPSQHSHGNPQSHE-CTPTHYLSHAETENYLTPSPDSPDQWSSSSPHSTSDWSDGISSP

>Dre1a

CEIAQCEGRGGNAICDTQCNNHACGWDGGDCSLNFDDPWQNCSAALQCWRYFNDGKCDEQCATAGCLYDGFDCQRLEGQCNPLYDQYCRDHYADGHCDQGCNNAECEWDGLDCADDVPQKLAVGSLVLVVHIPPDELRNRSSSFLRELSSLLHTNVVFRRDANGEALIFPYYGSEHPRTRRELDHEVKGSIVYLEIDNRQCFQQSDECFQSATDVAAFLGALASSGNLNVPYIIEAVTSE-GPP-KTGEMYPMFLVLLALAVLALAAVGVVVSRKRKREHGQLWFPEGFKRREPVGEDSVGLKPLKN-SDSSLMDE--QLSWAEDDT--NKRFRFEIL-EDHRQWTQQHLDAADLRL-NSMAPTPPQGQIENDCMVNVRGPDGFTPLMIASCSGGGLENENGEAEEDPSADVITDFIYHGANLHNQTDRTGETALHLAARYARSDAAKRLLESCADANVQDNMGRTPLHAAVAADAQGVFQILIRNRATDLDARMHDGTTPLILATRLAVEGMVEELINCHADPNAVDDSGKSALHWAAAVNNVDAAVVLLKNGANKDLQNNKEETPLFLAAREGSYETAKVLLDHLANRDIADHLDQLPRDIAHERMHHDIVRLLEEYNLVRSPLCCPNTYLG-IKPSPTAKKTRKPGGKGVGGSGKDIRTKKKKS-VGVLSPVDSLESPHGYLSDFLTPPSQHSY--SNPMD-NTPNHQ-QVPD-HPFLTPSAGSPDQWSSSSPHSLSDWSEGISSP

>Dre1b

CEIEQCKVKKGNKICDSACNNYACDWDGGDCSLNFNDPWKNCSAALQCWRYFNNGKCDEQCHNTGCLYDGFDCQRVEAQCNPLYDQYCKDHFADGYCDQGCNNAECEWDGLDCANDTPEKLAAGLLVVVVHIHPDQLRNNSFGFLRELSRVLHTNVVFRRDSKGQEMIYPYYGNEQGRKRRELEKQVKGSVVYLEIDNRQCYQQTSECFQSANDAAAFLGALASSGSLKMPYVIEAVTSEIDPS--PVELYPVYVVLAGLALLAFVAIGMVASRKRRREHGQLWFPEGFKRREPVGEDSVGLRPLKNCSDISLMDD--NQNWGEEEQSDSKRFRSEML-DDHRQWTQQHLDAADLRI-PSIAPTPPQGEIENDCMVNARGPDGFTPLMIASCSGGGLETGNSEEEEDASANVINDFIYQGANLHNQTDRTGETALHLAARYARSDAAKRLLEASADANIQDNMGRTPLHAAVAADAQGVFQILIRNRATDLDARMHDGTTPLILAARLAVEGMVEELINCHADVNAIDDFGKSALHWAAAVNNVDAAMVLLKNGANKDMQNNKEETPLFLAAREGSYETAKVLLEHFANREITDHMDRLPRDIAQDRMHHDIVRLIDEYNLVRSPLCSPNGFMGNMKPSVQSKKPRKPSTKGIGCDGKDMKVKKKKASSAVLSPVDSLESPHGYISDFLTPPSQHSY--TPALDANTPNHQLQVPDHHPFLTPSPGSPDQWSSSSPNSMSDWSEGISSP

>Xtr1

CENEQCAELADNKICNANCNNHACGWDGGDCSLNFNDPWKNCTQSLQCWKYFNDGKCDSQCNNSGCLYDGFDCQKVEVQCNPLYDQYCRDHFQDGHCDQGCNNAECEWDGLDC-DNMPENLAEGTLLIVVLMPPEKLKNNSVNFLRELSRVLHTNVVFKKDSKGEYKIYPYYGNEEGRRRRELDQEVRGSIVYLEIDNRQCYKSSSQCFTSATDVAAFLGALATHGNLNIPYKIEAVKSEIVTAKPPPPLYAMFSMLVIPLLIIFVIMVVIVNKKRRREHGQLWFPEGFKRREPLGEDSVGLKPLKNLTDGSFMDD--NQNWGDEETLENKRFRFEMLPEDHRQWTQQHLDAADLRI-PSMAPTPPQGEIDADCMVNVRGPDGFTPLMIAACSGGGLETGNSEEEEDASANMISDFIGQGAQLHNQTDRTGETALHLAARYARADAAKRLLESSADANVPDNMGRTPLHAAVAADAQGVFQILIRNRATDLDARMCDGTTPLILAARLAVEGMVEELINAHADVNAVDEFGKSALHWAAAVNNVDAAAVLLKSSANKDMQNNKEETPLFLAAREGSYETAKVLLDHYANRDITDHMDRLPRDIAQERMHHDIVHLLDEHNLVKSPICSPNGYMGNMKPSVQSKKARKPSIKGNGCEAKELKARRKKSSSGVLSPVDSLESPHGYLSDFLTPPSQHSY--SSPMD-NTPSHQLQVPD-HPFLTPSPESPDQWSSSSPHSMSDWSEGISSP

>Hsa1

CELPECQEDAGNKVCSLQCNNHACGWDGGDCSLNFNDPWKNCTQSLQCWKYFSDGHCDSQCNSAGCLFDGFDCQRAEGQCNPLYDQYCKDHFSDGHCDQGCNSAECEWDGLDCAEHVPERLAAGTLVVVVLMPPEQLRNSSFHFLRELSRVLHTNVVFKRDAHGQQMIFPYYGREEGRRRRELDPDVRGSIVYLEIDNRQCVQASSQCFQSATDVAAFLGALASLGSLNIPYKIEAVQSETVPP-PPAQLHFMYVAAAAFVLLFFVGCGVLLSRKRRRQHGQLWFPEGFKRREPLGEDSVGLKPLKNASDGALMDD--NQNWGDED-LETKKFRFEVLPDDHRQWTQQHLDAADLRM-SAMAPTPPQGEVDADCMVNVRGPDGFTPLMIASCSGGGLETGNSEEEEDAPA-VISDFIYQGASLHNQTDRTGETALHLAARYSRSDAAKRLLEASADANIQDNMGRTPLHAAVSADAQGVFQILIRNRATDLDARMHDGTTPLILAARLAVEGMLEDLINSHADVNAVDDLGKSALHWAAAVNNVDAAVVLLKNGANKDMQNNREETPLFLAAREGSYETAKVLLDHFANRDITDHMDRLPRDIAQERMHHDIVRLLDEYNLVRSPLCSPNGYLGSLKPGVQGKKVRKPSSKGLACEAKDLKARRKKSSSGMLSPVDSLESPHGYLSDFLTPPSQHSY--SSPVD-NTPSHQLQVPE-HPFLTPSPESPDQWSSSSPHSVSDWSEGVSSP

>Gga1

CEIAVCASYAGNKICDGKCNNHACGWDGGDCSLNFNDPWKNCSQSLQCWKYFNDGKCDSQCNNAGCLYDGFDCQKYEGQCNPLYDQYCKDHFSDGHCDQGCNNFECEWDGLDCANNMPEKLADGTLVVVVLITPENLKNNSFNFLRELSRVLHTNVVFKKNAKGEYMIFPYYGNEERRQKRELDQDIRGSIVYLEIDNRQCIQSSSQCFQSATDVAAFLGALASLGNLNIPYKIEAVKSETAPA-RNSQLYPMYVVVAALVLLAFIGVGVLVSRKRRREHGQLWFPEGFKRREPLGEDSVGLKPLKNASDGTLMDD--NQNWGDEETLDTKKFRFEMLPDDHRQWTQQHLDAADLRI-SSMAPTPPQGEIDADCMVNVRGPDGFTPLMIASCSGGGLETGNSEEEDDAPA-VISDFIYQGASLHNQTDRTGETALHLAARYSRSDAAKRLLEASADANIQDNMGRTPLHAAVSADAQGVFQILIRNRATDLDARMHDGTTPLILAARLAVEGMLEDLINCHADVNAVDDLGKSALHWAAAVNNVEAAVVLLKNGANKDMQNNKEETPLFLAAREGSYETAKVLLDHFANRDITDHMDRLPRDIAQERMHHDIVRLLDEYNLVRSPLCSPSSYIGNLKPAVQGKKARKPSTKGLSCDSKDLKARRKKSNSSVLSPVDSLESPHGYLSDFLTPPSQHSY--SSPLD-NTPSHQLQVPD-HPFLTPSPESPDQWSSSSPHSVSDWSEGISSP

>Xtr2

CTPSHCPGKAGDGHCDRECNVAACKWDGGDCSLSVDDPWRNCPRQ-ECRSLFNNSQCDKECTSADCLYDNFDCRREERSCNPVYEKYCMDHYGDGRCDKGCNSEECGWDGLDCAKNSKEVLADGVLVVVVLVPPNEFLKQSTAFLQRLSSLLHTSLRFCTDSNGNYKIKPYRKSQ-GRKKRELDSDIIGSEVMLEIDNRICVKEMPDCFPNTETAARFLAALSVLERLQFPYPIKAVRSEKIEEVIDNFFLPLFAV-AGVLILIVAVLGVIVSR-RKRAHSTLWFPEGFDRREPVGQDALGLKNIAK--AERLMED--PSEWTETDCPETKKLKTEELSPDPRPWTQHHLAAADIRLPPATALTPPQGDYESDGMVNVRGPDGFTPLMLASFLGGGLEPESQEEEEEGEASAITDLICQGASLGAQTDRTGETALHLAARYARADAAKRLLDAGADPNAQDHTGRTPLHAAVAADAQGVFQILIRNRSTDLDARMGDGSTALILAARLAVEGMVEELITCHADVNAVDELGKSALHWAAAVNNIEATLALLKNGANKDMQDSKEETPLFLAAREGSYEAAKVLLDHYANREITDHMDRLPRDIAQERLHHDIVQLLEEHNTVRNLLCPPNGFMGSVKQTPQGKKNRRANAKANGAEAKDAKGRGKKLSSVTLSPVDSLDSPRAYSANMSAPPQQT----FAPSQEDTPKHYLHIPNEHPYLTPSPESPEQWSSPSPHSVSEWSDATPSP

>Hsa3

CPRAACQAKRGDQRCDRECNSPGCGWDGGDCSLSVGDPWRQC-EALQCWRLFNNSRCDPACSSPACLYDNFDCHGRERTCNPVYEKYCADHFADGRCDQGCNTEECGWDGLDCASEVPALLARGVLVLTVLLPPEELLRSSADFLQRLSAILRTSLRFRLDAHGQAMVFPYHRPSPPRARRELAPEVIGSVVMLEIDNRLCLQSNDHCFPDAQSAADYLGALSAVERLDFPYPLRDVRGEPLPPEPSVPLLPLLVA-GAVLLLVILVLGVMVAR-RKREHSTLWFPEGFGRREPVGQDALGMKNMAK--GESLMGE--VATWMDTECPEAKRLKVE---MDCRQWTQHHLVAADIRVAPAMALTPPQGDADADGMVNVRGPDGFTPLMLASFCGGALEMPTEEDEDDTSASIISDLICQGAQLGARTDRTGETALHLAARYARADAAKRLLDAGADTNAQDHSGRTPLHTAVTADAQGVFQILIRNRSTDLDARMADGSTALILAARLAVEGMVEELIASHADVNAVDELGKSALHWAAAVNNVEATLALLKNGANKDMQDSKEETPLFLAAREGSYEAAKLLLDHFANREITDHLDRLPRDVAQERLHQDIVRLLDQPS---GLLCPPGAFLPGLKAAQSGSKKSRPGKAGLGP-GPRGRGKKLTLSSVTLSPVDSLDSPRPFGGPYLAVPGHGEEYPVAGAHSSPPKARFRVPSEHPYLTPSPESPEHWASPSPPSLSDWSESTPSP

>Dre3

CPIADCFSKANDGVCDKECNSLDCRWDGGDCSLAV-NPWARCADP-RCWRLFNNSQCDEFCNNAECLFDNFDCINKEKVCNPIYEAYCTDHYADGLCDQGCNTEECGWDGLDCARKIPEDLAEDMLVIVVLLPPEELLRTQTAFLQKLSAILRTTLRFRLDRNGDYMIRPYTGRE-TRIKRELNPEVIGSIVYLEIDNRLCSQGSDDCFRNADSAAEYLGALSAREMLRFPYPIKEVTSEKRPSITEIPEWARLLLVASLFLLVILMVGMLIAR-RKREHSTLWFPEGFNRREPVGQDSLGMKHMPKTVEESLLAD--HSDWIDTDCPEAKRLKVEILSDDSRQWTQHHLAAADIRMPPSMALTPPQGEFDSDCMVNVRGPDGFTPLMLASFCGGGLEEVTEDDDDESSANIISDLIYQGASLAAQTDRTGETALHLAARYARADAAKRLLDAGADANAQDNTGRSPLHAAVAADAQGVFQILIRNRATDLDARMYDGSTALILAARLAVEGMVEELITCHADVNAVDEIGKSALHWAAAVNNVEATIALLKNGANKDMQDLKEETPLFLAAREGSCEAVKVLLAHFANREITDHMDRLPRDIAQERMHHDIVQLLDEYNTVRSLMCPPSNFLQGLKSTPQGKKNRRPGAKGIGGDSAKGRNKRLTLSSVTLSPVDSLDSPRGGASNYPTPPSQHSY--SSAMD-ATPKHYLHVPNEHPYLTPSPESPEPWSSPSPHCVSDWSDSTPSP

>Dre2

CPYPDCEQRAGDKVCDLRCKNPECDWDGSDCTLHWDKPWKNCKASVSCPDLFHNGRCDPECNNAGCLFDSFECDSSVGICK--YDKYCADHYANGHCNQGCNTEACGWDGLDCARDTPPKLANGTLVIVVLLQPKELLADLKGFLRSLGTLLHTNLQVKLDEHNKLMVYPFYRNEQQRGKRELENEIIGSRVHLEIDNRLCTQNSDECISSADQAAAFIAAEYLKSDL--PYPIYSITTKPPETGFN--FLYLVAVAAAIILLI-LVLGVLAAK-RKRKHGTLWLPDGFKRREPVGQDDFGMKSMQKP-QDGGLLDCSSNHWSEED-HLPKKPRMELLPVDRREWTLQHHKAADI------TLTPPQADNDMESLVNVKGPDGFTPLMLASLRNGSSPLGEEEEEDEPGTNVITDLIGQGASLNAQTDRTGETALHLAARYARADAAKRLLDAGADANAHDNMGRTPLHAAVAADAQGVFQILIRNRATDLDARMNDGTTPLILSARLAVEGMVEELVHCHADVNAVDDHGKSALHWAAAVNNVDATLVLLKNGANRDMQDNKEETPLFLAAREGSFEAAQILLDHYSNRDITDHLDRLPRDTAQERMHHDIVRLLDEYNLVHSMVCGTNGTILGMRPGPQGKKNRRAGGKANGVDLKEMKAKRRKKSSVTMSPVDSLESPHSYAGEYPTPPSQHSYATAGSEG-TTPGHPAQKPSEHPYLTPSPESPDPWSSSSPHSNSDWSDVTTSP

>Xtr3

CHLHHCPDKARDGYCDQECNTHECLWDGGDCSLTMEDPWANCSSSLKCWEYF-NGQCDELCNTPECLFDNFECQEKESICK--YDKYCEDHYANGHCDKSCNTKECGWDGLDCSTDKPENLAEGTLVIVVLMPPKELLKNSRNFLRELGSILRTNLRIKRNPDGSPMVFPYFREKSLRVVRELEEQPIGSKVSLEIDNQQCVLDSDQCFKTTDSAAAMLAAHAVQGTL--NYPLVSVGGNRYTGKNRETHYPAFF--CVYRLYVPAPKSISFIRVQAQVAG----VEGCRKYQPYATYKLFLRNLSVPIVDGNLLDSNQNDWSDDRGPQPKKSKSELLSGDSRQWTQQHLVAADIRMTPSLALTPPQAEQDMDVIVNVRGPDGCTPLMLASLRMGSPHIEEDDEEEDPSANVITDLIYQGACLQAQTDRTGETALHLAARYSRADAAKRLLDAGADANSQDSMGRTPLHAAVSADAQGVFQILIRNRVTDLDARMNDGTTPLILAARLAVEGMVADLINCQADVNAVDDHGKSALHWAAAVNNVEATIVLLKNGANRDMQDNKEETPLFLAAREGSFEAAKMLLDYFANREITDHMDRLPRDIAKDRMHHDIVRLLDEYNLVHSAVCGSNRSFLNLKHAAQTKKARRPSAKSVAPEPKDTKNRRRKKSSVTLSPVDSLESPH-----YPTPPSQHSYTSTD----KTPNHNGHLPGEHPYLTPSPESPDQWSSSSPNSASDWSDVTTSP

>Hsa2

CLSQYCADKARDGVCDEACNSHACQWDGGDCSLTMENPWANCSSPLPCWDYINN-QCDELCNTVECLFDNFECQGNSKTCK--YDKYCADHFKDNHCDQGCNSEECGWDGLDCAADQPENLAEGTLVIVVLMPPEQLLQDARSFLRALGTLLHTNLRIKRDSQGELMVYPYYGEKSRRSLPGEQEEVAGSKVFLEIDNRQCVQDSDHCFKNTDAAAALLASHAIQGTL--SYPLVSVVSE--SLTPERTLLYLLAV-AVVIILFIILLGVIMAK-RKRKHGSLWLPEGFKRREPVGQDAVGLKNLSVQVSEANLIGTGTSEWVDDEGPQPKKVKAELLSEDRRPWTQQHLEAADIRRTPSLALTPPQAEQEVDVLVNVRGPDGCTPLMLASLRGGSSDLSDEDEDEDSSANIITDLVYQGASLQAQTDRTGEMALHLAARYSRADAAKRLLDAGADANAQDNMGRCPLHAAVAADAQGVFQILIRNRVTDLDARMNDGTTPLILAARLAVEGMVAELINCQADVNAVDDHGKSALHWAAAVNNVEATLLLLKNGANRDMQDNKEETPLFLAAREGSYEAAKILLDHFANRDITDHMDRLPRDVARDRMHHDIVRLLDEYNVTPSVICGPNRSFLSLKHTPMGKKSRRPSAKSTMPEAKDAKGSRRKKSSVTLSPVDSLESPHTYVSDYPTPPSQHSYASSNAAE-RTPSHSGHLQGEHPYLTPSPESPDQWSSSSPHSASDWSDVTTSP

>Gga2

CLDSQCAEKARDGYCDEDCNTHACQWDGGDCSLTMEDPWANCSSSLRCWMLF-NGQCDEFCNTPECLFDNFECQQNSRMCK--YDKYCADHYGDGRCDQGCNSEECGWDGLDCAGDKAERLAEGTLIIVVLMRPDELLRDVRSFLRTLGTLLHTNLRIKLDSQGNPMVFPYYGEKSVRKHRELEQEVIGTRVFLEIDNRQCAEDSEQCFHNTEAAAALLAAQAIKGML--PYPFVSVQSE--PLLPPKTLLYLLAV-AALIILLILLLGVMMAK-RKRKHGSLWLPEGFKRREPVGEDAVGLKNLSVQIPEGNMADSGPTEWAGDGGPQPKRVKTELLPEDQRQWTQQHLEAADVCGSTSLALTPPQADQEVDVLVNVRGPDGCTPLMLASLRVGGSDISEDDEDEDSSANIITDLIYQGANLQAQTDRTGEMALHLAARYSRADAAKRLLDAGADANARDNMGRTPLHAAVAADAQGVFQILIRNRVTDLDARMNDGTTPLILAARLAVEGMVAELINCQADVNAVDDHGKSALHWAAAVNNVEATLVLLKNGANRDMQDNKEETPLFLAAREGSFEAAKILLDHFANRDITDHMDRLPRDVAQDRMHHDIVQLLNEYNVAHSVICGPNRSFLNLKHASLSKKSRKPNAKGIMPLTKDAKRRRKKSSSVTLSPVDSLESPHAFASEYPTPPSQHSC--YSSATERTPSHNRHLQGEHPYLTPSPESPDQWSSSSPHSASDWSDVATSP

>Nve

CPVPNCAKKFDGGKCNPKCNTHECNWDGTTCSLGI-EPWSNCTTVTKCYQVFANGVCDRECNTGGCLFDGFDCKPSVPKCG--ADKYCAARFANAECDAICNNVACQNDGLDCSFKKPE-IVEGTLVLVLLVVPEAFMNGSRVFMRELSRTLNTIAFIKKDSEGKELVKVYPLPPSERRKRSAEK--IWVQVQINLDNRGC---ETDCFQSTEQAAKYLGAQQSTGKLNLPYPVYSEKPTVEETGFQPELWIAILCVGVPLFIVGVLAG------GKRVYTKLWLPEGFLRRDPVGQEHSMRNKSSDLEEEGAVGG----DTPPQEARDAKRVKLEQRVKDTRQWTRLHREAADVRNCTALALTPPQGESEKPGIVDARGPGGFTPLHLASCRGTLVDGCSIDDDDDSGGAMVSDLLALGASYGARTDIEKETPLHLAARHSRADAAKRLLHAGADPNARDKLGRTPLHLAVGADAQGVFQILLRNRTTDLEAAMEDGTTPLILAARLDLLDIVKDLIKASCKVNNVDAQGKSALHWAAAVNSHEVTSELCKNGAKKDMQDDKGQTPLFLGAREGSLEAVRILLLSYANRMIADNMDKTPEEVARQRAHNDIVEL---------------------------------------------------------------------------------------------------------------------------LSDWS------

>Hro2

CIFNGCHWKAGDGVCDEECNNKNCLNDGGDCEST--DPFQSCNVS-TCSQTFNDSVCNQECNNIDCLFDGFDCLDRRQTCF--HEDYCSYHYADGNCD---------------------------------------------------------------------------------------------------------------------------------------------------------------------------------------------------------------------------------------------------------------------------------------------------------------------------------------------------------------------------------------------------------------------------------------------------------------------------------------------------------------------------------------------------------------------------------------------------------------------------------------------------------------------------

>Hsa4

CQKPGCEGRSGDGACDAGCSGPGGNWDGGDCSLGVPDPWKGCPSHSRCWLLFRDGQCHPQCDSEECLFDGYDCE-TPPACTPAYDQYCHDHFHNGHCEKGCNTAECGWDGGDCRPEDDPEWGPSLALLVVLSPP-ALDQQLFALARVLSLTLRVGLWVRKDRDGRDMVYPYPGARAGKETDSLSA---GFVVVMGVDLSRCGPDASRCPWDPGLLLRFLAAMAAVGALPLPGPLLAVHPHAGTAPPANQPWPVLCSPGVILLALGALLVLQLIRRRRREHGALWLPPGFRRRPPLGEDSIGLLKPKAEVDEDGVVMCSGPE--GEEV---------TGPPTCQLWS---LSGGCGALPQAAMLTPPQSEMEAP--LDTRGPDGVTPLMSAVCCGEVQ-SGTFQGACPEPW---EPLLDGGACPQAHTVGTGETPLHLAARFSRPTAARRLLEAGANPNQPDRAGRTPLHAAVAADAREVCQLLLRSRQTAVDARTEDGTTPLMLAARLAVEDLVEELIAAQADVGARDKWGKTALHWAAAVNNARAARSLLQAGADKDAQDNREQTPLFLAAREGAVEVAQLLLGLGAARELRDQAGLAPADVAHQRNHWDLLTLLEEARHKATVSVPPHGGLPRCRTLS-------AGAGPRGGWSVDLAAR----GGGAYSHCRSL-SGVGAGGGIPIPP--------------------------PCLTPSPESPQLDCGP--------PALQEMP

>Cel1

CEKRKCSERANDGNCDADCNYAACKFDGGDCS-GKREPFSKCRYGNMCADFFANGVCNQACNNEECLYDGMDCLPAVVRCPVKIREHCASRFANGICDPECNTNGCGFDGGDCDNETATIITN--IRITVQMDPKEFQVTGGQSLMEISSALRVTVRIQRDEEG-PLVFQWNGESESISRKIKRSTNIGVVVYLEVQE-NCD--TGKCYKDAQSVVDSISARLAKKGIDFGIPIEALVAEPRSGNNTGFSWNALLLIGCLIVMVVLMLGALRTRKRRMINASVWMP-------PMENEEKNRHQSITSSQHSLLEA--SYDYI-------KRQRNELYP-NPQGYGNGNDFLGDFNHTNLQIPTEPEPESPIKLHTEAAGSYAITPITRESIDPRHNRIASNSSAEDLIVHEAKECIAAGADV-NAMDCDENTPLMLAVLARRRRLVAYLMKAGADPTIYNKSERSALHQAAANRDFGMMVYMLNSTKGDIEELDRNGMTALMIVAHLLVEKGAKVDYDGAARKDSEKYKGRTALHYAAQVSNMPIVKYLVEKGSNKDKQDEDGKTPIMLAAQEGRIEVVMYLIQQGASVEAVDATDHTARQLAQANNHHNIVDIFDRCR-------PEREYDLHIQHTHQPQPSRK--------APKKQTSRSKKEDSTHLTP-------------YLSPEYQTEAGSSE-----------------AFQPQCGANGEMWYTRASTS---YTQMQNEP

>Cel2

CEQRKCMDLASNGICNPECNLEECNFDGGDCSGG-QRPFSKCQYPARCADQFANGVCNQECNNEECLYDGLDCQSELFRCPAHIRKHCIERRGDGVCNLECSFIGCGFDGGDC--NNTEAIILSDIRIKVQIDPIEFQATGGETLMQISANLRATVRIQRDELG-PLVFRWDGEHESHHVRRYRQVVTGIVLYLEVEE-ICK--PEFCFSTAQSVVDLIAAGLVKDGRSLGLPIEAMVAVPKGWSRSQVLFACIAFLGTVVAGVIAKNGPERSRKRKMVNATVWMP-------PMESTNEGRRNQSNHSSQCSLLDNSAYY------PNTKRHCSDIYPQYPGDYNELNFDFQS----ETFAP----ADLPADEIVQAAGPDAITPITNESVDSKYRRLAANVRGEDVITTEAIRCLKAGADV-NARDCDENTALMLAVRAHRVRLSVVLLREGANPTIFNNSERSALHEAVVNKDLRILRHLLTDKLKEIDELDRNGMTALMLVARLLLSKGAKLDYDGAARKDSNKYKGRTALHYAAMHDNEEMVIMLVRRSSNKDKQDEDGRTPIMLAAKEGCEKTVQYLALNDASLGIVDSMDMTAAQVAEASYHHELAAFLRQVA----NERHRNDIQQIVKSGHGAKSGRQKNIKRAGSTPTSAASSRETNSDGSFSS----PSPHYYPTTYTTPP--------------------------PYQDPNYRPPNTAFQN--------AEQMNGS

**NOTCHLESS**

>Spu

------------------------------------------------------------------------------------------------------------------------------------------------------------------------------------------------------------------------------------------------------------MALSTDYVMRTSWFDPEELSKIAQDRYDTV-K--GSEPERLVTGSDDFTLFLWQP------EKEKTSVARMTGHMQLINDVAFSPDTRLVASASFDKSIKLWNGHTG---------------------NRLLCSGSSDSTLKVWDVKTNKMSGDLPGHADEVYAVDWSPDGQRVASGGKDKVLKM-

>Bfl

------------------------------------------------------------------------------------------------------------------------------------------------------------------------------------------------------------------------------------------------------------MALSTDYAIRTGAYDPEEIKKKALERYNTA-K--GTGTERLVSGSDDFTLFLWQP------SLDKKSLARMTGHMALINEVCFSPDARLIASASFDKSVKLWDGKTGKYLTSLRGHVNAVYQVAWSADSRLLVSGSSDSTLKVWDVRTRKLTVDLPGHADEL--ENMTTQCSQENCTIQKKVLL--

>Vca

-------------------------------------------------------------------------------------------------------------TPQ----GHRNWVLVVAWSPDAAYVASGDMNGVVHLWEPGKL-GTCSGHGKWITSLAWQPAHKALPSQRFVSGSKDNTIKVWDALTRRCVLSMSNHTQMVTAVKWGGEGLIYSASRDCTISAWDDTDGKLVRVFKGHGHWVNTLALSSEYVLRTGAFDHEAAKQKAAERYAEA-T--AGKPERLVSGSDDFTLCLYEP------STSKTPIARMTGHVQLINQVVFSPDGRYILSASFDKSVKLWDGARGGFLATFRGHVGPVYQVAWSADSRMFVSGSKDSTLKVWDLRTRKLKVDLPGHSDEVFTVDWSPDGGSVASGGKDRVLKLW

>Tva

FVDSNGVSTGEQISLPATTTVKNLNDLINQLLPYTFIVLGGLDKLITEKVMQIVYRPESIYSVRSVSFCSATLPGHTHIVLCIAFSADGLELATGGGDGSVIFWDVGTQNLKQKIKAEKFWIQCIQWYSDSKTVAVSGTEGKIIILKKTFQTNTFKATNGGVCALEWEPMHLKSPHPRLAAATTNGEVAIFCSGTGRRLVALNGHTNLVMGFAWNGQGVIFSSSNDHTLKAFDSNTGEQLDVRSDKCSELRTLSISSQHVLRTGGWEYPDMKVAAEKRYKNFLR--TCPRENVAVGDSMGRIFLYKFQ-----NGKFEDMKLLTGHTNIIHHVLFSPNGYWLASAGDDKTVRLFDGKTGKFICNLRGHIKAVYRLSWSADSRLLISASEDTTLKVWDIARQKMKNDLPGHEDAVYAVDWSPAGGRAASGGKDKKVKLW

>Tth

LINGENSEPSQVFEIPEETNTKELNALVNKLNPYSFQITDNI----AEQVVTITYYPQAVFRCRPISRSTHTMTGHSNSILSCKFSQDSSKLGSCSGDHTVRVWDLNTCTPICTLKGHGDWVLNLDWHYGNKLLASGDKLGKVIVWELGTDGKQLWSHKNFISDIQWKPEVF-GDSAIFASSSRDMSSKIYDARRGEVIRTLGGHTQGVTSVRWSGENILYTSSRDRMINVYDMRQANPIHVLKGHSHWINGISTSTEYVLRQGGYDPDKGIDGAKKRLDKIMG--CGGGERLVSASDDGTLYMWMP------LQSQKPVHRLVGHSSQVMSCKFSPDSRIIASTGCDKNMRIWDGFTGSCLHTYRGHVQTIYGCAWSPDSRMLVSASKDSTVKLWNVVPRKLMTNLPGHLDEVFSIDWSLDGSSVATASYDHTIKIW

>Lma

LLDEHGTPSSTQILLPASATPKQLDELLSSLLPYAFQINRSVQDILEETVVEIMYKPQAVFRVRPVTRCAGTLDGHSEAVLVVSFSPDSQVLATGGGDKEIRIWDMNTLTPVEELKAHTSWVQVLSWSPDGRYLVSGSKDGILANWTHGNFCKKHKAHTQYVSHVSWEPLHRNPQCDRFVSASKDASLKMWNMATG-LERSLSGHQSCVTCVKWGGEDRIYSSSQDRTVIVWDAGTGSPWCVLRGHAHWVNFLALSTDLVTRTGVFDHEDMCAHARKRYDAVVTRFG-GSERLVSCSDDNTMFLWNP------QQSVTPVARMTGHQGVVFHIQFSPDGTMLASCSADKSVKLWNAEDGRFITTFRGHVAAVYHVSWSLDSRMLVSGSKDTTVKLWSVAKRELVEDMSGHSDEIYATDWSPDGQKVATGSKDKRVRIW

>Mbr

LKTEQGEAAGPPLELPIDVTPQQLQLLANSLLPYAFEIVETLQALLAESIVSIVCQPQAIFKVRGISQCSATIPGHADNIVDAYFSPNGQQLATGSGDKTVRFWDVNTQTPKSTCKGHRHWVQCIAWSPDGLYVASGGRDNELRIWDGHEMGKPLVGHRAYITWIAWQPLHLKGKSQLVASSSKDAQTKIWDLSKGRCVITLSQHTKCVTCVKWGGSDLIYTASQDTTIKVWRASDGVLCRTLQGHAHWVNSLSLSTDFAMRTGPYDEGDAIAAAKDKYDAVVK---AVGEKLVSGSE----------------------------------------------------------------------VRPILQ----------------------------------------------------------------

>Uma

FQSGQGSLLGPTITLPGATGQKELEAIINQLRPYAFEETEQLVKSNQEDTLTVVFEPQAVFKVRPVSRCSSTMSGHASPILCSTFSPTGSLLATGAGDKTARLWDLDTETPMHTLVGHSNWVLCAEWEGRERKLATGGMDGEVWIWEAGEQGKPLRGHTKWITSLSWEPIHMNPTQPRLASSSKDGTVRVWNATLRRCEYVLGGHTASVNCVRWGGEGAIYTASSDRTVKVWSADGGRLLRTLNEHAHWVNTIALSTDFVVRTGPFDHEDAQACALKRYNQA-TSNGTRPETIITGSDDHTLFLWPPQMNGSASTPKKPVARLTGHQKTVNHVAFSPDANKIASASFDNSVKLWDAQTGKFIATLRGHVASVYRLAWSSDSRLLVSASKDSTLKLWDIKTFKIRKDLPGHTDEVYCVDFVAD--KVASGGRDKNVKIW

>Ngr

------------------MSKLQLNQLLNSNLPYSFEIQASVRDTLQDAPLKIVYHPQALFRVRPVSRCTSTLEGHSEAILSVNFSPNGEQLATGSGDNTVRIWDLNTETPQHTLKGHESWVLCVAWSPDAKKLASGSKDCNIKIWYGGKKGKVLKGHKKWITGLSWEPFLKNTACKRLASCSKDGTIIIWDTVMTQKEFTLSGHSKGVSGIMWGLNDILYSCSQDCTIRLWNTDTKQCVQVLSGHAHWVNTMSVSTEYFLRRGAFDHEEVDKRKEDLLSLYNKTFGSQPIRIITGSDDNTLILWEP------SKSDKPIARLTGHKGVVNQVKFSPDGRLIASASFDKTIKLWDAQTGKYLCSMRGHVGAVYQCCWSGDSRLLLSASKDSTAKVWDVQKRQLLNDMPGHYDEVFAVDWSPGGDKAASGGKDRMLKIW

>Ppi

FVSSEGVESGAPLEIPLAVCAAQLQVIVNALL-YQFEVTDTLQKTLTEKAIEIRYEPQAVFRVRAVTRCSSTVPGHAEALINASFSHDGTQLASGSGDATVRFWDLTTESPLHVCKGHKQWVLVTAWHPSGQFLASGCKKGDVCVWDPGKQGKVLSGHKKYVGTLAWQPLHCTENGIFLASGSKDNTIKIWEITRGQCTVTLSSHTQGITAIRWSGDNLIYSASQDRTIKVWRPKDGVLCRTLQGHAHWVNTMALSTDYAIRIGSWDPNPASTDALTVLQKF-A--GAGEEFMGSGSDDFT-----------------------------------------------------------------------------------------------------------------------------------------

>Ehi

FKNAEGEKVDNILQVPLTTKAKELEAMINEIL-YTFEIKTSIDDFATEVTLDITFHPQSLFFVRPITRQSSSLPGHTDSVLSVQFSPDGQNLASGSGDTTVRFWDVNTELPKETGKGHRNWVLVIQWSPNGKMLASGDMNGDICLWDPGKQGLTMKGHNKWITSISWEPLHMNKDCELFASASKDASIRIWSSRSQQCCICFGGHSKSITKVLWGGQGLIYSSSEDTTIKVWK-KDGSMEKELKGHAHWVNTICLSTEHALRTGYFDEEDQQKKALEKYTKL-K--GSKNERLVSGSDDNTLYMWDP------VDSRKPIIRLTGHTKPVNHVQFSPDGRYFISASFDKNLKLWDGFNGAYIASFRGHVASVYQIAWSPDNRLFVSGSKDSTMKVWDIKTKKLMFDLPGHADEVYGVDWSPDGLKVCSGGKDKLLKIW

>Spo

FRASDDSNELASLLVPGNSSVRQLEALLNQLLPYNFEIQDNLYTSVTEDHLTLLYTPQAVFRVRAVTRCTASMNGHDGTIISAQFSPTSSRLVTGSGDFTARLWDCDTQTPIATMKGHTNWVSCVAWAPDASIIATGSMDNTIRFWDPGSPGDALRRHTKPIMALCWQPLHLDSGPYLLASGSKDNTVRIWNVKLRTLLFTLSGHTAPITCVRWGGQNWIYSSSYDKTIRIWDAKDGKCLHILKGHAARVNHLSLSTEHVLRSGAYDHSDERRKAKERYEACLK---QSGERLVSASDDLQLILWDP------QKSTKPITKMHGHQKVVNHASFSPDGRCIATASFDSSVRLWDGKTGKFLATLRGHVAAVYQCAWSTDSRLLVSSSQDTTLKVWDVRSKKMKFDLPGHEDQVFAVDWSPDGQRVASGGADKAVRIW

>Sce

FQALDGDNVGGALRVPGAISEKQLEELLNQLNPYTFDITDNLYSSLTEDQITLLYTPRAVFKVKPVTRSSSAIAGHGSTILCSAFAPTSSRMVTGAGDNTARIWDCDTQTPMHTLKGHYNWVLCVSWSPDGEVIATGSMDNTIRLWDPGQCGDALRGHSKWITSLSWEPIHLPGSKPRLASSSKDGTIKIWDTVSRVCQYTMSGHTNSVSCVKWGGQGLLYSGSHDRTVRVWDNSQGRCINILKSHAHWVNHLSLSTDYALRIGAFDHEEAQKKALENYEKICKKNGNSEEMMVTASDDYTMFLWNP------LKSTKPIARMTGHQKLVNHVAFSPDGRYIVSASFDNSIKLWDGRDGKFISTFRGHVASVYQVAWSSDCRLLVSCSKDTTLKVWDVRTRKLSVDLPGHKDEVYTVDWSVDGKRVCSGGKDKMVRLW

>Aae

-------------------------MLVSLLNPYLFEVRDSIQQTLVENVLDIVYQPQAVFRVRPVTRCTSSMPGHAEAIVSLSFSPNSLHLASGSGDTTLRLWDLTTETPHFTCTGHRNWVLSVAWSPDSLKVASADKAGEIRVWCPGKLGRPLVGHKKWVSCLSWEPYHKNPECRYLASAGNDNDVRIWDVVLGTCTKTIAGHTAPVTAVRWGGSGLLYTSSRDRSIKMWRAEDGVLCKTFTGHAHWVNNLALNTDYVLRTGPFHPTEMQKSALERFEKV-C--PDGVESFVSCSDDFTLYLWK-------SSQKQFITRMTGHQNVVNDVKYSPDVKLIASASFDKSVRLWRAGDGAFICAFRGHVQAVYTVAWSADSRLILSGSKDSTLKVWSVKERKLAQELPGHADEVFGVDWAPDGSRVASGGKDKVLKLW

>Ath

LTDPEGTHLGSAMYIPQKAGPLQLTQLVNRFLPYSFELLVPVGTYLVEKVLTIVYQQQAVFRIRPVNRCSQTIAGHAEAVLCVSFSPDGKQLASGSGDTTVRLWDLYTETPLFTCKGHKNWVLTVAWSPDGKHLVSGSKSGEICCWNPGELGSPLTGHKKWITGISWEPVHLSSPCRRFVTSSKDGDARIWDITLKKSIICLSGHTLAVTCVKWGGDGIIYTGSQDCTIKMWETTQGKLIRELKGHGHWINSLALSTEYVLRTGAFDHNEEKQKALERYNKT-K--GDSPERLVSGSDDFTMFLWEP------SVSKQPKKRLTGHQQLVNHVYFSPDGKWIASASFDKSVRLWNGITGQFVTVFRGHVGPVYQVSWSADSRLLLSGSKDSTLKIWEIRTKKLKQDLPGHADEVFAVDWSPDGEKVVSGGKDRVLKLW

>Tad

FKSETGELVGSPLDVPIEVDVKRLQLICNAVIPYAFEILDDLQTTITESIIEIVYQPQAVFRVKSVTRCSSTLPGHTEAVISVAYSPDGSYLASGSGDTTVRFWDINTETPDYTCKGHKHWILSIAWSPDGEKLASGCKKGEICLWDPGKQGHVLVGHKQWITYLSWEPMHKDSDCRFLASSSKDASVRVWDTVTGITKYVLSGHLQCVTCVKWGGEGLIYTSSQDRTIKVWRASDGILCRTLQGHGHWVNTMALNTDYAMRIGAYDPKNRQEKSLQKYNLALK--LTGKELMVSGSDDYTLFLWHP------AKEKTPIARMTGHQQLVNMVCFSPDTRLIASASFDKSVKLWDGITGRFITSLRGHVKSVYQVSWSADSRLFLSSSADSTLKVWDVKSKKLHMDLPGHADEVYAIDWSPDGEKVASGGKDRVLKLY

>Hro

LKDEAGETLGSTFNLPLNTSVEKLQNICKKLKLYSFEISTSLYETICESVLDIIYQPQANFKVLAVTRCSSSIPGHAGAILDVQCSPDGRYLASSSGDTTVRFWDVNTDTPQFMCKGHKHWVLFVAWSPCGRKLASGCKNGLVMIWDPGKQGRPLSGHSKWIMSIVWKPLHLDGSSRILASSSKDATIRIWDTVLGTCLMILSSHLQSVTCIRWSGANLIYSSSQDRTIKVWQPDKGTLCRTLEGHGHWVNHVALNTDYVMRTGAFDPSQLDGLLIKNLTKREQ--KLCPEKMVSCSDDHTMSLWQP------EQDKKPLCKMVGHQQAINEAVFSPDGRLLASASFDKSVKLWNGVSGTFIVTLRGHIQEVYQVCWSADSRMLCSASADSTLKVWDSK-GKLLYDLPGHADQVYALDWSPDGQRVVSGGKDKVLKVL

>Cel

FVSEDNELGGSGILVPVDISTNELQILCNQLLPISFEIVDSIRKSLFETTLKLVYQPQAVFRVRPVTRCSASIPGHGEPVISAQFSPDGRGLASGSGDQTMRIWDIELELPLHTCKSHKSWVLCIAWSPDATKIASACKNGEICIWNAGEQGKTLKRHKQWITSLAWQPMHKDPTCRLLASCGKDGNIFIWDTVQGTVVRCLSGHTASVTCLRWGGEGLIYSGSQDRTVKMWRADDGVMCRNMTGHAHWINTLALNTDYALRTSCFEPEECQKVAQTRYEAALE--IAGGERLVSGSDDFTLFMWNP------KETKQSINRMTGHMQLVNQVVFSPDTRYIASASFDKSVKLWCGRTGKYLASLRGHVGPVYQVAWSADSRLLVSGSADSTLKVFELKTKSLYYDLPGHGDEVFTVDWSPEGTKVVSGGKDKVLKLW

>Aqu

FKSEEGALAGPPFDLPVDTNRDSLHILCNSLLPYEFEVEGTVSDTLSERLLEIVYRPQALFRVRAVSRCTSSMPGHTEAVISVQFSPNGRHLASGSGDTTVRFWDVTTETPLYTCSAHKHWILFISWSPDGMKLASGCKKGQVCVWDPGKQGRTMTGHRQWITCLSWQPLHSDPSCRHLASASKDSTIRLWDVVLGNTLLILSGHSQCVTGIKWGGEGLLYSSSQDRTIKVWRA-DGVLCRSLEGHGHWVNTMCLNTDYVLRTGAFEPEEIGRLAQERYLKV-K--GDQPERLVSGSDDFTLFLWQP------SVSKKHLARMTGHQQLINDVKFSPDSRFIASASFDKSVKLWDGKTGKFLASLRGHVGPVYQISWSADSRLICSGSSDSTLK-------------------VFSVDWSPDGERVASGGRDRVLKIW

>Cin

MCNEDKVATGPPLDVPLTVTAQQLQLLCNSILPYAFEILSDLQSIITEGTIQIIYLPQAVFKVRAVTRCTSSIEGHAEAVLSVKFSPNGGYLASGSGDTTVRFWDLSTETPHYTCKSHRHWVLCIEWSPDGRTLASGCKNGQVCLWDPGKQGKTLTGHKQWITHLCWAPLHLDGTCRKLASSSKDTTIRIWDTNTCQCSIILSSHLQSVTCIRWSGEDLIYSASQDRTIKVWRPNDGVLCRTLQGHGHWVNVLALSTDYVMRTGAFEPAQLQKEALKRYNIV-K--GNTGERMVSGSDDFTLFLWNP------STEKKPIARMTGHQALVNDVKFSPDARLIASASFDKSIKLWNAKSGKFIVSLRGHVNSVYQLAWSADSRLLVSGSGDSTLKVWDTHKNKLLLDLPGHADEVYAVDWSTDGQRVASGGKDRVLKIW

>Hma

FVNEAHESIGAPLDLPTDLTHESLQNLCNALLPYAFEITSTLGYLLTEKIVQILYQPQAVFRVKAVTRCTSTIPGHAEAVISVAFSPDGKYLASGSGDTTVRFWDVNTETPHFTCKGHKHWILAIAWAPNACKLASGCKSSEIRIWDPGNQGKTLVGHKQWITMLAWEPIHRNVECRRLASSSKDGTIRIWDTILCKTLLVLSGHLQSVTCIRWGGEGLIYSASQDRTVKVWRDQDGALCRTLEGHGHWVNTMALNTDYAIRTGAFEP-------------------------------------------------------------------------------DNEI---------------------------------------------------------------------------------------

>Lgi

FKSEAGEVTGSPFDLPVNINVHKLELICNAILPYSFEITESLEKTLTEKVLDIIYQPQSVFKVRAVTRCTGTIEGHADAVISVAFSPDGRYLASGSGDTTVRFWDVNTETPQFTCKGHKHWILCIAWSPDGLKLASGCKNSQVCIWDPGQQGKSLIGHKQWITWLAWKPLHLDPECRYLASASKDSTVRIWDTLKSECHLTLSGHLQCISCVKWGGSNLIYTSSQDRTIKVYRAEDGVLCRTLQGHGHWVNTMALSTDYAMRTGWFDPEELGKKAQARYDAA-K--GSEPERLVSGSDDFTLFLWTP------ETEKKSVARMTGHQQLINEVQFSPDTRLIASASFDKSVKLWEGRSGKFLASLRGHVNRVYQVAWSADSRLLCSGSSDSTLKVWDILEKKLLFDLPGHADEVYAVDWSPDGQRVASGGKDKVLKIW

>Nve

FKSEDGELVGAPFDLPVDITPDKLELVCNAVLPYSFEVIGSLQDTLTEKVVEIVYQPQAVFKVRAVTRCTSSIPGHTEAVISVAFSPDGRYLASGSGDTTVRFWDVTTETPHFTCKGHMHWILHIAWSPDGKKLASGCKNGEIRIWDPGKQGKTLKGHLKWITWLSWEPLHRNPDCRYLASSSKDSSVKIWDTATGSVDKTFSSHTQSVTCVKWGGEGLIYSASQDRTIKVWRAEDGVLCRTLQGHAHWVNHMALNTDYVLRTGAFEPETLQSEASKRYKEA-K--GSKAERLVSGSDDFTLFLWEP------EAKTKPIARMTGHQALINQVCFSPDGRLIASAAFDKSVKLWNGETGKFITSLRGHVNCVYQIAWSADCRLICSGSADSTLKVWDMKTKKLLYDLPGHADEVYSVDWSPDGARVGSGGKDKVLKM-

>Dre

LQDEAGEVLGSPFDVPLDISPDKLQLVCNALLPFLFELVSSLGSCITEQVLPVVYQPQAVFRVRAVARCTSSLEGHTEAVISVAFSPTGKYLASGSGDTTVRFWDLSTETPHHTSRGHTHWVLSIAWSPDGKKLASGCKNSQIFLWDPGKQGKTLTGHTKWITWLCWEPLHLNPECRYLASTSKDCTIRIWDTVLGRYDKILTGHTHSVTCVKWGGDGLLYTSSQDRTIKVWRAKDGVQCRTLQGHAHWVNTLALSTDYVLRTGAFEPEEIKEKALKRYNSV-R--GEGHERLVSGSDDFTMFLWNP------AEDKKPVARLTGHQALVNEVLFSPDTRLIASASFDKSIKIWDGKTGKYLNSLRGHVGPVYQVAWSADSRLLVSGSSDSTLKVWDIKTGKLNADLPGHADEVFAVDWSPDGQRVASGGKDKCLRIW

>Hsa

FQDEGGQLLGSPFDVPVDITPDRLQLVCNALLPLAFEIVSSLGKTLTEKVLDIIYQPQAIFRVRAVTRCTSSLEGHSEAVISVAFSPTGKYLASGSGDTTVRFWDLSTETPHFTCKGHRHWVLSISWSPDGRKLASGCKNGQILLWDPGKQGRTLAGHSKWITGLSWEPLHANPECRYVASSSKDGSVRIWDTTAGRCERILTGHTQSVTCLRWGGDGLLYSASQDRTIKVWRAHDGVLCRTLQGHGHWVNTMALSTDYALRTGAFEPQELKERALSRYNLV-R--GQGPERLVSGSDDFTLFLWSP------AEDKKPLTRMTGHQALINQVLFSPDSRIVASASFDKSIKLWDGRTGKYLASLRGHVAAVYQIAWSADSRLLVSGSSDSTLKVWDVKAQKLAMDLPGHADEVYAVDWSPDGQRVASGGKDKCLRIW

>Xtr

FKDENGEVLGSPFDVPVDITSDKLQLVCNALLPLAFEIVTSLDKTLTEKVIDIIYQPQAVFKVRAVTRCTSSLEGHTEAVISVAFSPTGKYLASGSGDTTVRFWDLNTETPHFTSKGHTHWILSIAWSPDGRKLASGCKNSQIFIWDPGKQGKPLTGHSKWITWLCWEPLHLNPECRYLASASKDCTIRIWDTVMGQCQKILTSHTQSVTAVKWGGDGLLYSSSQDRTIKAWRAQDGVLCRTLQGHAHWVNTMALSTDYVLRTGAFNPEDLKEKALKRYNEV-R--GQGPERLVSGSDDFTLFLWAP------AEEKKPLQRMTGHQALINEVLFSPDTRIIASASFDKSVKLWDGKTGKFLASLRGHVSAVYQIAWSADSRLLVSGSSDSTLKVWDSKTKKLLVDLPGHADEVYSVDWSPDGQRVASGGKDKCLRIW

>Gga

FRDEAGEPLGSPFDVPVSITPDKLQLVCNALLPLAFEIVVSLEKTLTEKVLDIIYQPQAVFRVRAVTRCTSSLEGHTEAVISVAFSPTGKYLASGSGDTTVRFWDLSTETPQFTAKGHRHWVLSIAWSPDGKKLASGCKNSQIFLWDPGNQGRVLTGHSKWITCLCWEPLHINPECRYLASASKDGSIRIWDTLMGRCDKILTGHTQSVTCVKWGGDGLLYSSSQDRTIKVWRSQDGILCRTLQGHAHWVNTMALSTDYVLRTGAFEPAELKDKAQQRYDKV-R--GQGPERLVSGSDDFTLFLWRP------AEDKKPLERMTGHQALINQVLFSPDTRIIASASFDKSIKLWEGRTGKYLTSLRGHVSAVYQIAWSADSRLLVSGSSDSTLKVWDAETKKLAIDLPGHADEVFATDWSPDGQRVASGGKDKCLRIW

>Pra

-----GTNVGPQLEIPTSSNVRQMEELVNELLPYSLEVTTSLKATVTETALTITFQPLAAFRVRPVTRCSDTLQGHSEAILHVSFSPDGKRLASGGGDATVRFWDTNTCMPKHTGRGHKNHVLCTAWSPDGARFASADRNGEIRLWDPGKQGQPMKGHKQWVNSLTWEPMHRNATCERFASSSKDGSIKVWNARTGRSIASLSGHTDSVECIKWGGEGLLYSASRDRTIKVWSEGEGKLVRTLVGHGHRINTLALNVDYVCRSGPFGHEEMQEAALKRYQEV-R--KGQPERLVSGSDDFTLFLWEP------AENKKPLARLTGHQQPVNHLSFSPDGRYFASASFDKKVKIWNGQTGKFIATLTGHVGAVYQVCWSSDARLIVTASKDSTVKVWESAPKNAKTTLSGHADEVYALDWSPNGDMVASGSKDRTIKMY

>Pso

----------------MSSNVKQMEELVNELLPYSLEVTTSLKATVTETALTITFQPLAAFRVRPVTRCSDTLQGHSEAILHVSFSPDGKRLASGGGDATVRFWDTNTCMPKHTGRGHKHHVLCTAWSPDGTRFASADRNGEIRLWDPGKQGQPMKGHKQWVNSLTWEPMHRNATCERFASSSKDGSIKVWNARTGRSIASLSGHTDSVECIKWGGEGLLYSASRDRTIKVWAEGEGKLVRTLIGHGHRINTLALNVDYVCRSGPFSHEEMQQAALKRYQEV-R--KGQPERLVSGSDDFTLFFWEP------SESKKPLARLTGHQQPVNHLCFSPDGRYFASASFDKKVKIWNGQNGKFVATLTGHVGAVYQVCWSSDSRLIVTASKDSTVKVWESEPKNAKTTLSGHADEVYALDWSPNGDMVASGSKDRTIKM-

**NUMB**

>Tad

MKKFTRTLSFRRKHA----PKTTSSE---DQRSSYWEEDSYKVK-HGQIVFPVKYLGSLEVTKSRGTDICHEAAMAM-KNHKRKKTSLVININGVRVTDE-NTKQLLLDQTVEKISFCTPDPKDDRLFSYICRDGTSMKWLCHSFLTDKASGERISNALGSAFSESYLRKEDLRKKEVANEVCSVSLLLFDF------------------------------------------------------IFIFFKTMCLI------------------------------------------------------------------------------------------------YIYI

>Cel

MDRLRRSLRLPKKRRDRSHDRHLSPDVTGGSKTEQWQPDEGAVR-TGTCCFNVKYLGSVEVYESRGMQVCEGALKSLKR-RKPVKAVLYVSGDGLRVVDQGNSRGLLVDQTIEKVSFCAPDRQTDKGFAYICRDGASRRWMCHGFLATKETGERLSHAVGCAFSICLEKKKR-RDEETAQVNVQSAQESTSSTPPKDIFHPNWEDNTSEGRRISKPRPTGNPALFLRQGSLRAPPDMPSS------SDQFKRNMSLRTVSNNPTERSPDEDPL-------VVTKTSGSLSNNGLDGHNANGDFVAAWPQRVSMSMSPTS--PKSGPPPAH----DPFDVQWNTH-------------

>Hma

FRAIIRSLSFRKRKN----KKGD-SK---SGSQQSWEHDSVTIKQQGGCSFAVKYLGCLEVSESRGTQVCSQAAHQMKG-KRKQRVTLWVTEDTLRVTDD-ETKSLVVDQVIEKVSFCTPDPHDDKLFSYICRDGTSRRWLCHSFKGIKDSGERISHAVGCAFAACLAKKQQ-QNQQNQ------NLQRNTSTRKTFEKRTNG--------------------VVLPQQSVETLQKLNQE------------EYNGR-VNQANQSLEIEPVLA------------------------PKQADPSFARPR------------ALPPRLPVS----NPFSPP-NPFNE--------KFV

>Hro

MDKFKRTLSLKKKKE----SSG--GE---SIKPHQWQEDERKVR-EGSCSFQVKYLGCIEVFESRGIQVCEDAVKALKG---HHMAVLYVTGDALRVVDE-ISKGLIVDQTIEKVSFCAPDRNHEKGFAYICRDGTTRRWMCHGFLAVKESGERLSHAVGCAFAICLEKKQQ-REKEK--VTVTYNDNGTSFSRLGSFRQATMTERL---------------------------------------TDPQSAILAGRSL-----------------------------------------------------------------------------------------------IHL

>Lgi

MQSLKRRLSF--RKK----KDHV-PE---CSKPHQWQEDEKKVR-EGTCSFQVRYLGCIEVFDSRGMQVCEEAVKALKAKGKVQRAVLYVSGDALRVVDE-ISKSMIVDQTIEKVSFCAPDRNHEKGFAYICRDGTTRRWMCHGFMAVKESGERLSHAVGCAFAVCLEKKQK-RDKETG-VTVTFSEDRTSFTRTGSFRQTSMTERLADPFAVQRPHATPE--LFNRQGSFRGFEKLQEA------SSPFKRTLSLR-YSDLPSTLQRIHEVSSIAQMCQQLTQ--GLSALTTDETHHQHSAPSFQPPQPT----------QFPRPPPAHQRSNDPFDVAWNPYGG-KTVKKFEVQL

>Spu2

MKRLRRSFR---SKD----TSYV-PP---ASKPHQWQQDEKLVR-AGTCNFTVKYLGSIEVGESRGMQICEDAARQLRT-RKKLRAVLWVSSDGLRVVEE-ESKGLIVDQTIEKVSFCAPDRNNDKGFSYICRDGTTRRWLCHCFHSLREPGERLSHAVGCAFAACLERKQQ-RDKLCG-VKVEFDVNKTSFTRQGSFRMPVAKNVAPLPHAIPRRHASSD--MLIRQGSVR-LGKINE-------ATPFKRQLSLN-LKELPSTLQRTSPQESISSACAQLSS--GLFQLSKNEATQGVPQPGILRPEPSLNPSSRQNDQQVHGGAMQNGV--QQQSPAWNPFNN--AVKTFEINL

> Spu1

MKRLRRSFR---SKD----TSYV-PP---ASKPHQWQQDEKLVR-AGTCNFTVKYLGSIEVGESRGMQICEDAARQLRT-RKKLRAVLWVSSDGLRVVEE-ESKGLIVDQTIEKVSFCAPDRNNDKGFSYICRDGTTRRWLCHCFHSLREPGERLSHAVGCAFAACLERKQQ-RDKLCG-VKVEFDVNKTSFTRQGSFRMPVAKNVAPLPHAIPRRHASSD--MLIRQGSVR-LGKINE-------ATPFKRQLSLN-LKELPSTLQRTSPQESISSACAQLSS--GLFQLSKNEATQGVPQPGILRPEPSLNPSSRQNDQQVHGGAMQNGV--QQQSPAWNPFNN--AVKTFEINL

>Aae

MDRLRKSFRDSFRRR----KDRV-PE---AAKPHQWQADEQAVR-SATCTFPVKYLGCVEVFESRGMQVCEEALKVLRR-RRAIRAQLHVSGDGLRVVEE-DTKGLIVDQTIEKVSFCAPDRNTDRGFSYICRDGTTRRWMCHGFLASKDTGERLSHAVGCAFAVCLERKQR-RDKECG-VTMTFDMKNSTFTRTGSFRQQTMTERLANAFAIERPHATPS--MLERQGSFRGFTQIGT-------ASPFKRQMSLR-INDLPSNAERVSPIPTVSQLCQELSQGLSLLTRNDADDTTSTTTVTSAAIVDLPFSSTLP---SSVGTGPTPQ---DPFDAEWPPKPP---AQSFQVHL

>Nve

---------------------RI-PE---SHRPQVWENDSYKVR-NGGVSFPVKYVGAIEVTESRGTQVCAEAFRKMRGVHKKKRMNLLVTSDCIRVVDE-ETKSLTIDQTIEKVSFCTPDPSDDRVFSYICREGTTRRWMCHCFIAIRDTGERLSHAVGCAFTACLQRKQK-----------------------------------------------------------------------------------------------------------------------------------------------------------------------------------------

>Bfl

MNRLRKSFR---RSK----EPHV-PE---CSKPHQWESDEKAVR-SGTCNFHVK--------DSKGVR---GFFRRGKG-RKKTRAVLWVTADALRVVDE-DSKGLIVDQTIEKVSFCAPDRTYERGFSYICRDGTTRRWMCHGFMAIKDSGERLSHAVGCAFAACLERKQK-REKDCG-VTVTWNADKTSFTRQGSFRQTTMTERMDQEAAIPRQQAPLSTVALQRQGSFRFFPKLSSK------SSPFKRQLSLR-LNELPSNLQRAPPPPSISAMCKMIQS--DLFTLSSTDDRPPPPVDASAYVEWVNAPPIPPPRQNTEPFDPRSVNGDGSISDPWQPYDPFAVEKTFELQL

>Cin

MRTLRQSFR---RKK----KPKI-PE---SSKPHQWQQDEETVK-SAKCSFHVKYLGNIEVEESRGMAVCEQAVKQLKK-KKKIRAMLYVSPDALRVVED-STKALLLDQTIEKVSFCAPDRNYERAFSYICRDGTTRRWICHSFFAVKDSGERLSHAVGCAFAACLEKKQK-REKETG-VTVTYDQNRTTFTREGSFRVKTLTEQQEEASAIPRRHAPL---HLIRQASQRTFVSKNKQDGDKALNSPFKRNYSLP-LNNLPSNTSRVPELEDISGICSQITN--T-FS-NNNDD-------------PMPRLSSPT---PVVETTPMSNLNN--LNQQYPPWSN---KSGFEVKI

>Hsa

MNKLRQSLR---RRK----PAYV-PE---ASRPHQWQADEDAVR-KGTCSFPVRYLGHVEVEESRGMHVCEDAVKKLKG-RKSVKSVLWVSADGLRVVDD-KTKDLLVDQTIEKVSFCAPDRNLDKAFSYICRDGTTRRWICHCFLALKDSGERLSHAVGCAFAACLERKQR-REKECG-VTAAFDASRTSFAREGSFRLSGGGRPAEREAAIPRRHAPLE--QLVRQGSFRGFPALSQK------NSPFKRQLSLR-LNELPSTLQRVPEMESINALCTQISS--S-FA------HKRTPSEAERWLEQVAVFLPPPHMQPVVGITPSQMVANDPFEAQWNPFSG-DLQKTFEIEL

>Xtr1

MNKLRQSLR---RKK----PTYV-PE---ASRPHQWGADEEAVR-RGKCSFPVRYLGHVEVEESRGMHVCEDAVKKLKG-KKSVKAILWVSADGLRVVDD-KTKDLIVDQTIEKVSFCAPDRNFDKAFSYICRDGTTRRWICHCFMALKDSGERLSHAVGCAFAACLERKQK-REKECG-VTASFDASRTSFAREGSFRVTSASQQAEREHAIPRRHAPLE--QLVRQGSFRGFPALSQK------NSPFKRQLSLR-LNELPSTLQRVIELEGINDLCNQISS--S-FANKPSEDHRRTPSEAERWLEPVGMFITPH-MQPVVGITPSQMVANDQFEAQWNPFSN-ELQKTFQIEL

>Xtr2

MNKLRQSLR---RKK----PTYV-PE---ASRPHQWGADEEAVR-RGKCSFPVRYLGHVEVEESRGMHVCEDAVKKLKG-KKSVKAILWVSADGLRVVDD-KTKDLIVDQTIEKVSFCAPDRNFDKAFSYICRDGTTRRWICHCFMALKDSGERLSHAVGCAFAACLERKQK-REKECG-VTASFDASRTSFAREGSFRVTSASQQAEREHAIPRRHAPLE--QLVRQGSFRGFPALSQK------NSPFKRQLSLR-LNELPSTLQRVIELEGINDLCNQISS--S-FANKPSEDHRRTPSEAERWLEPVGMFITPH-MQPVVGITPSQMVANDQFEAQWNPFSN-ELQKTFQIEL

>Dre1

MNKLRQSLR---RKK----PTYV-PE---ASRPHQWQADEEAVR-KGKCNFPVRYLGLVEVDESRGMHVCEEAVKKLKG-KKTVKAVLWVSADGLRVVDD-KTKDLTVDQTIEKVSFCAPDRNYDKAFSYICRDGTTRRWMCHCFMALKDSGERLSHAVGCAFAACLERKQR-REKECG-VTASFDASRTSFVREGSFRVSSAAQQSDREHAIPRRHAPIE--QLVRQGSFRGFPQLSQK------NSPFKRQLSLR-LNDLPSTLQRVPEMDSINALCSQINS--S-FT-KPSEDHKRTPSEAERWLEPVSMFTQQP-LQPVVGITPSQMVANEHFDVKWNPFSN-DLQKTFEIEL

>Dre2

MNKLRQSFR---RKK----DVYV-PE---SSRPHQWQTDEEAVR-GGKCSFAVRYLGHVEVEESRGMHICEDAVKKLKG-KKAVRAVLWVSADGLRVVDD-KTKDLILDQTIEKVSFCAPDRNFEHAFSYICRDGTTRRWICHCFMAIKDSGERLSHAVGCAFAACLERKQK-REKECG-VTATFDANRTTFTREGSFRVTTATEQAEREHVIPRRHAPVE--ALARQGSFRGFPALSQK------TSPFKRQMSLR-MNELPSTMQRVPEVESISSLCTQITS--T-FS-GPPEDHKRTPSEADRWLEPSAVPSVPP-RQPVVGITPSQMVANDAFEAQWNPFSS-ELHKTFEIQL

>Xtr3

MNKLRQSFR---RKK----DIYV-PE---ASRPHQWQTDEESVR-NGKCSFQVKYLGHVEVEESRGMHICEEAVKRLKG-KKAIKAVLWVSADGLRVVDE-KTKDLLVDQTIEKVSFCAPDRNFDRAFSYICRDGTTRRWICHCFMAVKDTGERLSHAVGCAFAACLERKQK-REKECG-VTATFDASRTTFTREGSFRVTTATEQAEREHAIPRRHAPVE--QLARQGSFRGFPALSQK------MSPFKRQLSLR-INELPSTVQRVAEMESISALCSQITN--T-FS-MPPEDHKRTPSEADRWLESMPVSVVPG-LHPVVGITPSQMVANDPFEAQWNPFSS-DLQKTFEIEL

>Gga

MNKLRQSFR---RKK----DVYV-PE---ASRPHQWQTDEEGVR-TGKCSFPVKYLGHVEVDESRGMHICEDAVKRLKG-KKAVKAVLWVSADGLRVVDE-KTKDLIVDQTIEKVSFCAPDRNFDRAFSYICRDGTTRRWICHCFMAVKDTGERLSHAVGCAFAACLERKQK-REKECG-VTATFDASRTTFTREGSFRVTTATEQAEREHAIPRRHAPIE--QLARQGSFRGFPALSQK------MSPFKRQLSLR-INELPSTVQRVPEVESISALCSQITS--A-FS-TPSEDHRRTPSEADRWLEPVPVGVVPP-MQPVVGITPSQMVANDPFDAQWNPFSS-DLQKTFEIEL

>Hsa

MNKLRQSFR---RKK----DVYV-PE---ASRPHQWQTDEEGVR-TGKCSFPVK------------------------G-KKAVKAVLWVSADGLRVVDE-KTKDLIVDQTIEKVSFCAPDRNFDRAFSYICRDGTTRRWICHCFMAVKDTGERLSHAVGCAFAACLERKQK-REKECG-VTATFDASRTTFTREGSFRVTTATEQAEREHAIPRRHAPIE----------------------------------------------------------------------------------------------------------------------------------------

>Hsa4

MNKLRQSFR---RKK----DVYV-PE---ASRPHQWQTDEEGVR-TGKCSFPVKYLGHVEVDESRGMHICEDAVKRLKG-KKAVKAVLWVSADGLRVVDE-KTKDLIVDQTIEKVSFCAPDRNFDRAFSYICRDGTTRRWICHCFMAVKDTGERLSHAVGCAFAACLERKQK-REKECG-VTATFDASRTTFTREGSFRVTTATEQAEREHAIPRRHAPIE--QLARQGSFRGFPALSQK------MSPFKRQLSLR-INELPSTMQRVPEVESISSLCSQITN--A-FS-TP-EDHRRTPSEADRWLEPVPVGVVPA-LQPVVGITPSQMVANDPFEAQWNPFSS-DLQKTFEIEL

>Hsa3

MNKLRQSFR---RKK----DVYV-PE---ASRPHQWQTDEEGVR-TGKCSFPVKYLGHVEVDESRGMHICEDAVKRLKG-KKAVKAVLWVSADGLRVVDE-KTKDLIVDQTIEKVSFCAPDRNFDRAFSYICRDGTTRRWICHCFMAVKDTGERLSHAVGCAFAACLERKQK-REKECG-VTATFDASRTTFTREGSFRVTTATEQAEREHAIPRRHAPIE--QLARQGSFRGFPALSQK------MSPFKRQLSLR-INELPSTMQRVPEVESISSLCSQITN--A-FS-TP-EDHRRTPSEADRWLEPVPVGVVPA-LQPVVGITPSQMVANDPFEAQWNPFSS-DLQKTFEIEL

**PEN2**

>Ehi

MFFDTLNNIKARKICKILFICGFFFMPVMWMV-LIWF----LFKYVSPLASYRNRYIILSAVLIIIEFSLLLLWNIIFQYKWSTWGAVGDILSVNKYQGQL

>Ath

MEATRLTEEASVDYARRFYKFGFALLPWLWFVNCFYFWPVLRHSRAF---PQIRNYVVRSAIGFSVFTALLSAWALTFSIGGEQFGPLYDKLVMYNLSGLA

>Hro

M--RKLTSEEKLHICKMYFRIGFFLLPLVWIMNFLWFFKEVFRKTPTPMRKQMRKYLVLSLVGAICYSTLMLIWVIVYNLKRFDWDRFGDKLTLIFPVGEY

>CAEEL

MDISKLTDVKKVDLCKKYFLIGACFLPLVWIVNTFWFFSDAFCKPINAHRRQIRKYVIASIVGSIFWIIVLSAWEIFFQHYRAQGLVWTDFLTFVFPTGRV

>Ddi

-------------------------LTWVWLINILYFYRNSL-------NDKVKWYLKFSLIGFLGYSTIFMGWMGIYLVNRNKWGAFGDDISITIPFG--

>Mle

----------------KYFLAGLPLLPWVWIVNVLWFWREA-TKP--DHIPEVRKYVMYSAIGALAYLTVFGTWVGVYQSQRVAWGALGDRISIFVPDGEP

>Aqu

MNLESVSSEEKLLICRRYFIIGCFGLPFVWLVNGLWFIREGFFVKS-EVTTRIRRYVLFSWLGALIWIAAFILWTSIYQTQRQNWGKSGIYLSFVLPGGVA

>Tad

MNLEKTTDEEKVNVCRLYTNVGFLFLPFAWVINIVWFYKYAFTTPPFQGQAEIKKYVIRSAIGTIIFLIAFIIWNVEFQRNRQSWGAIGDTLSFVIPKGA-

>Aae

MDLNRVPNERKLYLCKWYFKVGFALLPFVWAINTVWFFSEAFRKPAYDEQKEIRKYVIFSAIGSLIWFTLILTWVMTFQLKRTEWGDFADSISFIIPLGRA

>Hma

MDLKKVKDEEKVRLSRIYMYGGFVFLPFLWFINTVWFFRDAFCKEEFEGQSLIRRSVIISAMGAVIWSIGISVWVVMYQVNRANWEEIGDKLSFLIPLGQP

>Nve

MDLRKVSDEEKVKLCRKYTLVGLAFLPFLWLVNAIWFFREAFCRESFPGQKSMRSNVIKSFIGFSIWAAGLSTWIVMYQTNRASWGELGDTLSFLIPLGEP

>Hsa

MNLERVSNEEKLNLCRKYYLGGFAFLPFLWLVNIFWFFREAFLVPAYTEQSQIKGYVWRSAVGFLFWVIVLTSWITIFQIYRPRWGALGDYLSFTIPLGTP

>Xtr

MNLERVPNEEKLQLCRKYYLGGFALLPFLWLVNVVWFFKEAFFKPAYTEQPLIQSYVKRSALGLFVWVVILTTWISVYQTHRAGWGATGDYLSFTIPLGIP

>Dre

MNLERIPNEEKLSLCRRYYLGGFAFLPFLWLVNILWFFKEAFLKPAYTEQPQIKSYVKKSALGLLLWVAVLTTWITVFQHFRAQWGEVGDYLSFTIPLGTA

>Lgi

MDLRRVQNEDKLQLCRKYYIGGFALLPFLWCINSIWFFNEAFRKPHYEEQRQIKTYVIRSMIGTVIWIAIIIAWVTIFQMNRSSWGATADYMSFIIPKGEV

>Bfl

MNLNRVKDEDKLELCRKYYYGGFFALPFLWLVNVVWFFKQAFIRPAFEQQQEIKSYIIKSLVGCILWTAVLVTWMTIFQMYRADWGEIADNMSFIIPKGIA

>Cin

MDLEKVTAEEKLKLCKIYYIGGFFALPFLWFVNAIWFSREAFFKPAFEQQQQIRTYVIRSGIGSLVWIAALLAWNIVFQLNRAAWGATGDYISFIIPTGIP

>Spu MNLAKVSDEEKLSLCRKYFIGGIFALPFLWLVNTVWFFREAFLRSAFEQQKKIRSYVTWSLIGCLVWTTGLIAWITVYQVKRAEWGETGDRLSFIIPRGRP

**PRESENILIN**

>Aqu

EEALRFGAQQMILLIFPVFICMALVVAIQLSVEKNVT--SSGT--LIYTPFDE--DTASNDGFVLLF-ALANVAIVITLVVVMTIILVCLYN---------------------------------------------EVLQVHYLFIDWPSFLVLIWNFGGMGVLVIHW-KG-PLRLQQAYLIFCSALTANIFVKYLPNWTAWILLAAISLYDLIAVLCPKGPLRVLVETARERNETIFPSLIYSTTMVW-LVGMAERQG--FKLGLGDFIFYSILVGKAAHDSTGDWVVISSCFVAILIGLCMTIIILGIVR---RALPALPISIFCGLIFYFSSQYVIAPFAQVLATTQTFI

>Tva

HTFLEFYARKVAKIAVPVVLTLILDAVCIRFIERTHGSAELNRN-FVDTMSRN--DSGISVTASIWS-----AVGIIFMIIVVTAILLTLYYFGCMKIIFGWLILAVSLLLS---------MYFL-VGFG-------TYPSIVNIPVDYLSLAVFLLNLVVVGNMSIFW-RA-PQRITQAFLVLISILTSIVF-RYLPDWTVWILLVLLIIYDACVVLCPNGLLILLLKKSEERGDAI-PALLYSAAA-WEEADPEDNEG--IKLGLGDFVFYGILVSRAARI---GWDITILCIFAVILGLSLTLVCLAVWE---RPLPALPFSLALGIVFFIIGAMTFRQFCEHMRWGLVAF

>Pra

YDDLMHGINSFWAIVFPVCVTMIIASLVVVNYRSSSIEASMSTY-LVYGDSGADTSSSSSSGGSGIGESLVNALVIIGAIAVLTFGMALLYKYNCMKFLSGYIMFASTAILS---------FVGGQLVDE-------IVNDQFGWAVDWPSFLFVMINFGFVGVISIFYQKGTPKFVQNGYLVMVSVILAWEFSMW-PEWTTITFCVMFACYDLCAVLTPCGPLKYLIGLIQEKQAPL-PGLLYEADV---RDGVSDDKT--IKLGLGDFIFYSVLVGRAAIK---DFSTFAVTFVCIVMGLGGTLFLLAVLH---KALPALPISIFLATIFYFLTEYIFIDFCSFMMTFPAAV

>Pso

YDDLMHGINSFWAIVFPVCVTMIVASLVVVNYRSSSIEASMSTY-LVYGNSGS--DSSSSSGGAGIGESLVNALVIIGAIAVLTFGMALLYKYNCMKFLSGYIMFASTAILS---------FVGGQLVDE-------IVNDQFGWAVDWPSFLFVMINFGFVGVISIFYQKGTPKFVQNGYLVMVSVILAWEFSMW-PEWTTITFCVMFACYDLCAVLTPCGPLKYLIGLIQEKQAPL-PGLLYEADV---RDGVSDDKT--IKLGLGDFIFYSVLVGRAAIK---DFSTFAVTFVCIVMGLGGTLFLLAVLH---KALPALPISIFLATIFYFLTEYIFIDFCSFMMTFPAAV

>Lma

ARLSRGVGIRFVSLVVPVTVTMLAVVWSLSCLSPIYV--NSQAPPLPVVVNENDAG---TAGEKFVY-SLVAALIVVGCVVVATFATVVLYHFHLQFVLYGWLAFSAVSMFF---------MLLW-IWLD-------LFCTYFQIPYNVISMGIFVWNFGVVGLIALFY-YS-HPTVTQVYLVIASILTAWSL-TALPEWSTWALLMCIATYDILAVLWQQGPLHRLIKIAQERDEPI-PGFVYSSAH---SIVPIHATP--FKLGLGDFIFYSLLVGRASFSGFVSWGF---CMVSILAGMLGTLLSLLLFRNSLRALPALPCSIFLSTVVFVLCRLIVESLSSFTSHHLLVL

>Vca

PSLLDNLGEEVTGIVAPVSLCMAVTVLLVRLLNPEGSSSSSSVL-IANIAYQE--QATDSSGKKLGG-ALLNAIIFVAVIGGMTILLFLLFKYKCYKFIYAYMGFAVFNIFF---------FLTGALFIQ--------VMQVIKLHIDAFSLAYGLLNFSVVGTMGLLF-VPIPLLMKQLYLIWVGIIVAYIF-TWIPEWTAWVILVLMALYDIAAVLIPGGPLKALVEMAIERKQDL-PALVYEARP----AGGRLPDA--IKLGLGDFIFYSMLVGRAAMY---DFMTVFSAYLAIIAGLGLTLLCLAIYQ---KALPALPFSIALGVAFYFLTRLTLEPFLVPMSTHLTYF

>Ath

RSILDSLGEELIAILTPVSICMFTVVLLVCILNSDPSSSSASFSSIATAAYSES-DSD-SSWDKFVG-ALLNSVVFVAAITVATFVLVLLFYLRCVKFLKFYMGFSAFIVLG---------NLGGEILVL--------LIDRFRFPIDSITFLILLFNFSVVGVFAVFMSKF-SILITQGYLVWIGVLVAYFF-TLLPEWTTWVLLVALALYDIAAVLLPVGPLRLLVEMAISRDEDI-PALVYEARP---VIRNDSSGA--IKLGLGDFIFYSVLVGRAAMY---DLMTVYACYLAIIAGLGITLMLLSVYQ---KALPALPVSIMLGVVFYFLARLLLEVFVVQCSSNLVMF

>Ehi

MITFAEYSESVNAVITPVAITLLLTVIIVKLLERSNDLYSQATS-YIFSE-----GIESSTTIPIWLIAIIVSVIFIIMIVIVTFVFVLLFYYRCMKLIVGWLLFSVILLLM---------LFGG-SIMK-------SLLSVFNWPVDWISFAFLLFNFGVLGVISIFY-IS-PMKLNQFYMIIISVFMA-SFFTNLPEWTTWTLLIGMACYDLVAVLCPKGPLRILVNLAQERKEPI-PALVYSTA-VW--MGLAHGKG--VKLGLGDFVFYSVLVGRCAMY---DLTIVFSGSVAVLSGLFGTLILLVVFN---KALPALPISIFFGTLIYCVSRWAIVPLVTTANLYGYVA

>Ddi

EVSLQDFSSMIVSIIIPVSITMMAVVFFVKYLNNQTLYASTLSYTIAGGSSGGGSGADSITGNSFVD-SLIVAGIVLGMIIVTTVAFVLLYKYRCLKILYGWLFLSVGMMLG---------SFGT-TFFQ-------AMLSAANLPLDYITFAFLIFNFTVCGIIGVFW-YA-HQYVNQLYLVIISVLMAISLTR-LPQWTIFTLLVIVAIYDLFAVLCPRGPLKVLVELSQERNENI-PALVY------------SDSN--LKLGLGDFIFYSLLISRAALV---HMSCVFSTFIAILTGLFLTLLCLAIFK---KALPALPISIFLGILFYYLSNNFLTPFIEALTLSQIFV

>Hgi2

EEDLQYGAESIIMLFIPVSICISFVIASVQALDFYKE---EGLP-LPFTPFVP--EEKDEVKDTLWK-SLANSLIIVGVVIIMTILLFLLFKYRCYKVCAFWMFLATLLLLF---------IFSY-IYYS-------EFCRYLNTATDFISTGFNLWNFGLMGILAIHW-KS-PLIVQQFYLIVVSALMALTFIKHLPGWTAWALLIVISVWDLIAVLTPKGPLKGLVNISSERRENLFPALVYSSTPFSAVVATSYNAG--IKLGLGDFIFYSLLVGKVSQL--EEWNTTTACFISVLIGLSLTILLLVLYE---QALPALPISVFFGVAFYFGGYYTIDPFVSVCVSRQAFI

>Hgi1

ELDLKYGAGHVIQLFIPVSLCMAAVVCGINFIEFYNV---KDVY-LMYTPFEQSVTPETSTGTRIWQ-SLANALIMLSVVIVMTVVLLLLYKYRCYKFIAGWLFMSSFMLLF---------FFTF-IFYS-------ELCRTMNIPMDYITTAVFLWNFGVLGIMCIHW-KG-PLLLQQFFLIVVSALMALTFIKHLPDWTTWVLLAVISIWDLVAVLAPKGPLRSLVKLAQERNDPIFPALIYSTTMAW-LVEPSEDSG--VKLGLGDFIFYSVLVGKASLL--GDWNTTVACFIAILMGLCFTLLLLASFR---KALPALPVSIAFGLIFYFATSQVVLPFMVMCSLNQVFI

>Mbr

ELDLKYDAESVLALIKPVSACMIVVIATIRSITYFSQ---NDTQ-FAYTPFESNGGAGESSGERFGG-AVLNALIVVGIVIVMTFILVMLYIYEYYKIIYGWLALSALLLLY---------FFSY-QYIECVMCTRRQVLIAHNASIDWITMAFIIWNFGTVGFIAIFW-RS-PLAVQQVYLVIVSALMALVLIKNLPDWTTWVLLAAIAIYDLFAVLSPCGPLKCLVEVAQERNQPLFPSLIYSSTMMW-TVTMADDSG--VKLGLGDFIFYSVLVGKAATA--HEWTTILACYVAILIGLACTLLLLSIFK---KALPALPISIFFGLCFFFLTSEVLDPLIDRLNERQIFL

>Ppi

VETLKYGAKHVMMLIVPVSVCMMLVIAIIRTVTMYDQGPQQQVY-LVYTPFNEN-NKDISTGSPVSD-AVLNMLIVVSLVVVMTCFLVLLYKYRCYKIIYGWLFLSSLILLF---------MFSF-IYLQ-------EVIVRFNVAMDYITCGYIVWNFGVMGIISIFW-KG-PLILQQAYLVITSAFMALVFIKFLPEWTLWFLLGGIAIYDLFAVLCPKGPLRILVELAESRNEPIFPALIYSSTVMWTITGMTSDPN------------YNQLNESNSSL-----------------------------------------------------------------------

>Tad

----------------------------------------------------------------------------------MTVLVVLLYKYRCYKIIHGWLLITSVLLLF---------FFNF-IFFL-------NILEVYNVSLDWISAGLVMWNFGLIGLLAVHW-KG-PLMLQQIYLIIISALLALTFIKYLPDWTTWFVLGAISLWDLVAVLCPFGPLRILVETAQERNEPIFPSLIYSSTVAW-IVNMADQSG--VKLGLGDFIFYSILVGKAASN--RDWNTTIACFVAILIGLCMTLLLLAIFK---RALPALPISIAFGLIFNFATSGLIAPFSDSLQSNQVYI

>Hma

EEMLQYGAKSVLMLIIPVSICLLVVVATISSVTYYTQ--NAGTY-LVYTPFHE--EGNISHAEKFGQ-ALANSLIVIGVILVMTIILVILYKCRCYRIIEAWLILSSLMLLF---------FFST-IYLQ-------ELLRVYNVAMDYITVVLLIWNFGVVGMICIHW-KG-PLLLQQAYLIIISALMALVFIKYLPDWTLWFILAAVSIYDLFAVLCPKGPLRILVQTAQERNEQLFPSLIYSSTFMYPTVVNQEERG--VKLGLGDFIFYSVLVGKASSY--KDWNTTLACFVAILIGLCLTLLLLAIYR---KALPALPISITFGLIFNFVTSYFVKPFTDHLAGEQAFI

>Nve1

-----------------------------------------------------------------------------------------------------------------------------------------------------------------------MW----------------------------------------------------------------------------------TVGMAEEKG--VKLGLGDFIFYSVLVGKASSY--KDWNTTIACFVAILIENSAGENEEEVER----SGEGPTSEEARNAVRNLGEAGQQQPQQNGEEEESE--

>Nve2

EEMLKYGARSVMMLIIPVSTCMLVVVATISSVTYYTE--NSGQY-LVYTPFHE--ETGISNAQKAGE-AIANALIVIGVVLVLTIILVVLYKFRCYCIISGWLVLSSLMLLF---------FFGY-IYFQ-------ELLRVYNVAMDYITLSLILWNFGVVGMICIHW-KG-PLILQQAYLILVSALMALVFIKYLPDWTTWAILAAISLYDLFAVLCPKGPLKILVQTAQERDEPLFPSLIYSSTMMW-TVGMAEEKG--VKLGLGDFIFYSVLVGKASSY--KDWNTTIACFVAILIGLCLTLLLLAIYR---KALPALPISITFGLIFNFATKELVKPFMDSLSSKQAFI

>Aga

EEGLKYGAQHVIKLFVPVTLCMMVVVATISSINFYTI---KDVY-LVYTPFHE--LTD-DTGTKIWN-ALANSLILMTVIVIMTILLIVLYKHRCYKVIHGWLILSSLLLLF---------LFSG-LYLF-------EILRAYNVPMDWFTAGLLVWNFGVVGMISIHW-QG-PLRLQQGYLIFVAALMALVFIKYLPEWTTWAVLAVISIWDLIAVLTPKGPLRILVETAQERNEQIFPALIYSCKMHH-KKRLSQLGG--IKLGLGDFIFYSVLVGKASSY--GDWNTTIACFVAILVGLCLTLLLLAIFR---KALPALPISIFFGLIFCFVTSVIVKPFTEALTLEQVFI

>Spu3

DEMLKYGAKHVIMLFVPVSLCMLVVVATISTVSFYTE--SGDVY-LIYTPFHE--KSD-QAGTKAWN-ALANALIIIGIVLIMTIFLVVLYKYRCYKVIHGWLVLSSLLLLF---------FFTF-FYLQ-------ELLVTYNIPMDYFTIAVIMWNFGMVGMVSIHW-KG-PLRLQQLYLIVISALMALIFIKYLPEWTLWTILAAIAVYDLFAVLCPKGPLRMLVETAQERDEQIFPALIYSSTMVW-LVGMAEERG--VKLGLGDFIFYSVLVGKASAS--GDWTTTIACFVAILIGLCLTLILLAIFK---KALPALPISIAFGLVFYFCTSNLVFPFTDELASQQVYI

>Spu1

DEMLKYGAKHVIMLFVPVSLCMLVVVATISTVSFYTE--SGDVY-LIYTPFHE--KSD-QAGTKAWN-ALANALIIIGIVLIMTIFLVVLYKYRCYKVIHGWLVLSSLLLLF---------FFTF-FYLQ-------ELLVTYNIPMDYFTIAVIMWNFGMVGMVSIHW-KG-PLRLQQLYLIVISALMALIFIKYLPEWTLWTILAAIAVYDLFAVLCPKGPLRMLVETAQERDEQIFPALIYSSTMVW-LVGMAEERG--VKLGLGDFIFYSVLVGKASAS--GDWTTTIACFVAILIGLCLTLILLAIFK---KALPALPISIAFGLVFYFCTSNLVFPFTDELASQQVYI

>Spu2

DEMLKYGAKHVIMLFVPVSLCMLVVVATISTVSFYTE--SGDVY-LIYTPFHE--KSD-QAGTKAWN-ALANALIIIGIVLIMTIFLVVLYKYRCYKVIHGWLVLSSLLLLF---------FFTF-FYLQ-------ELLVTYNIPMDYFTIAVIMWNFGMVGMVSIHW-KG-PLRLQQLYLIVISALMALIFIKYLPEWTLWTILAAIAVYDLFAVLCPKGPLRMLVETAQERDEQIFPALIYSSTMVW-LVGMAEERG--VKLGLGDFIFYSVLVGKASAS--GDWTTTIACFVAILIGLCLTLILLAIFK---KALPALPISIAFGLVFYFCTSNLVFPFTDELASQQVYI

>Bfl

EMTLKYGAKHVIMLFAPVSLCMAVVVATISSITFYTE--KNG-Y-LIYTPFHE--EGA-STASKVGD-SLANGAIMVGVILVMTVFLVILYKYRCYKFIHGWLILSSLMLLF---------LFAY-IYLG-------EVLQAYNIPCDYITLAIVMWNFGAVGMVCIHW-KG-PLLLQQAYLIVISALMALVFIKYLPDWTTWFILGAISLYDLVAVLCPKGPLKVLVETAQERNEPIFPALIYSSTMMW-FVGMAEERG--VKLGLGDFIFYSVLVGKASSN--GDWNTTLACFVAILIGLCLTLLLLAIFK---KALPALPISITFGLIFNFATANLVTPFTDALASQQVYV

>Cin1

EEELRYGASTVISLFIPVTLCMLVVVATITSVSYYTD--QSQQQFLVYTPFHT--TNA-APAQVVWE-SIANSLIMIGVIVVMTVFLVLLYKYRCYKVIHGWLILSTLLLVF---------LFAY-LYLA-------EVLRAYNTAVDYITVVIVLWNFGVVGMICIHW-KG-PLLLQQAYLIIVSSLMALVFIKYLPEWTAWVILGFISIYDLLAVLCPKGPLRMLVETAQSRNESLFPALIYSSGMMW-TVGMAEDKG--VKLGLGDFIFYSVLVGKASAT--GDWNTTIACFVAILIGLCLTLILLAIYR---KALPALPISITFGLVFYFSTDYVVRPYMDSINSAQVYL

>Cin2

EEELRYGASTVISLFIPVTLCMLVVVATITSVSYYTD--QSQQQFLVYTPFHT---TNAAPAQVVWE-SIANSLIMIGVIVVMTVFLVLLYKYRCYKVIHGWLILSTLLLVF---------LFAY-LYLA-------EVLRAYNTAVDYITVVIVLWNFGVVGMICIHW-KG-PLLLQQAYLIIVSSLMALVFIKYLPEWTAWVILGFISIYDLLAVLCPKGPLRMLVETAQSRNESLFPALIYSSGMMW-TVGMAEDKG--VKLGLGDFIFYSVLVGKASAT--GDWNTTIACFVAILIGLCLTLILLAIYR---KALPALPISITFGLVFYFSTDYVVRPYMDSINSAQVYL

>Lgi

EESFMYGANSVIMLFVPVTLCMAVVVATISSVTFYTE--KTDGY-LIYTPFHD--KTD-DTGTKLWQ-SFANAFILLGVICVLTIFLLLLYKFRCYKVIHGWLIVSSLMLLF---------LFSY-IYLG-------EVLRAYNVPMDYVTVIILMWNFGVVGMVCIHW-KG-PLLLQQAYLIMISALMALIFIKYLPDWTTWVVLGVMVVWDLVAVLCPKGPLRVLVETAQERNEPIFPALIYSSTVMYQTITMAEERG--VKLGLGDFIFYGVLVGKASSY--GDWNTTLACFVAILIGLCFTLLLLAIFK---KALPALPISITFGLVFNFATSSLVQPFMDSLAAEQVYL

>Xtr2

ELTLKYGAKHVIMLFVPVTLCMVVVVATIKSVSFYTE--KDGQ--LIYTPFSE--DTT-SVGQRLLN-SVLNTLIMISVIIVMTIFLVLLYKYRCYKFIHGWLILSSLMLLF---------MFTY-IYLS-------EVFKTYNIAMDYPTLFMVIWNFGAVGMICIHW-KG-PLQLQQAYLIMISALMALVFIKYLPEWSAWVILGAISVYDLLAVLCPKGPLRMLVETAQERNEPIFPALIYSSAMVW-TVGMAEERG--VKLGLGDFIFYSVLVGKAAATASGDWNTTLACFVAILIGLCLTLLLLAVFK---KALPALPISITFGLIFYFSTDNIVRPFMDTLASHQMYI

>Xtr1

ELTLKYGAKHVIMLFVPVTLCMVVVVATIKSVSFYTR--YDGQ--LIYTPFTE--DTD-SVGQRALN-SILNTAIMISVIIVMTILLVVLYKYRCYKVIHGWLIISSLLLLF---------FFSY-IYLG-------EVFKTYNVAMDYITLALMIWNFGVVGMICIHW-KG-PLLLQQAYLIMISALMALVFIKYLPEWTTWLILAVISVYDLIAVLSPKGPLRMLVETAQERNETLFPALIYSSTMIW-LVKMAEERG--VKLGLGDFIFYSVLVGKASATASGDWNTTLACFVAILIGLCLTLLLLAIFK---KALPALPISITFGLVFYFATDYLVQPFMDQLAFHQFYI

>Dre2

ELTLKYGAKHVIMLFIPVTLCMVVVVATIKSVSFYTE--KSGQR-LIYTPFEE--DPN-SVGQRLLN-SVLNTLVMISVIVFMTIILVLLYKYRCYKFIHGWLILSSLMLLF---------WFSF-MYLG-------EVFKTYNVAMDYPTLLMIIWNFGVVGMICIHW-KG-PLRLQQAYLIVISALMALIFIKYLPEWSAWVILGAISIYDLIAVLCPKGPLRMLVETAQERNEPIFPALIYSSAMVW-MVGMAEDRG--VKLGLGDFIFYSVLVGKAAAT-GGDWNTTLACFVAILIGLCLTLLLLAIFK---KALPALPISITFGLVFYFSTDNLVRPFMDSLAAHQYYI

>Dre3

ELTLKYGAKHVIMLFIPVTLCMVVVVATIKSVSFYTQ--KDGQQ-LIYTPFRE--DTE-TVGQRALH-SMLNAIIMISVIVVMTLVLVVLYKYRCYKVIQAWLFFSNLLLLF---------FFSL-IYLG-------EVFKTYNVAMDYFTLALIIWNFGVVGMICIHW-KG-PLRLQQAYLIMISALMALVFIKYLPEWTAWLILAAISVYDLLAVLCPKGPLRILVETAQERNEAIFPALIYSSTMVW-LFNMAQDRG--VKLGLGDFIFYSMLVGKASATASGDWNTTLACFVAILIGLCLTLLLLAIFK---KALPALPISITFGLVFYFATDNLVRPFMDQLAVHQFYI

>Dre1

ELTLKYGAKHVIMLFIPVTLCMVVVVATIKSVSFYTQ--KDGQQ-LIYTPFRE--DTE-TVGQRALH-SMLNAIIMISVIVVMTLVLVVLYKYRCYKVIQAWLFFSNLLLLF---------FFSL-IYLG-------EVFKTYNVAMDYFTLALIIWNFGVVGMICIHW-KG-PLRLQQAYLIMISALMALVFIKYLPEWTAWLILAAISVYDLLAVLCPKGPLRILVETAQERNEAIFPALIYSSTMVW-LFNMAEERG--VKLGLGDFIFYSMLVGKASATASGDWNTTLACFVAILIGLCLTLLLLAIFK---KALPALPISITFGLVFYFATDNLVRPFMDQLAVHQFYI

>Gga1

ELTLKYGAKHVIMLFVPVTLCMVVVVATIKSVSFYTR--KDGQ--LIYTPFTE--ETD-TIGQRALN-SILNAAIMISVIIVMTILLVVLYKYRCYKVIHGWLIISSLLLLF---------FFSF-IYLG-------EVFKTYNVAMDYITVALIIWNFGVVGMICIHW-KG-PLRLQQAYLIMISALMALVFIKYLPEWTAWLILAVISVYDLVAVLCPKGPLRMLVETAQERNETLFPALIYSSTMVW-LVNMAEERG--VKLGLGDFIFYSVLVGKASATASGDWNTTLACFVAILIGLCLTLLLLAIFK---KALPALPISITFGLVFYFATDNLVQPFMDQLAFHQFYI

>Gga2

ELTLKYGAKHVIMLFVPVTLCMIVVVATIKSVRFYTE--KNGQ--LIYTPFSE--DTP-SVGQRLLN-SVLNTIIMISVIVVMTVFLVVLYKYRCYKFIHGWLILSSFMLLF---------LFTY-IYLG-------EVLKTYNVAMDYPTVILIIWNFGAVGMICIHW-KG-PLQLQQAYLIMISALMALVFIKYLPEWSAWVILGAISIYDLIAVLCPKGPLRMLVETAQERNEPIFPALIYSSAMIW-TVGMAEERG--VKLGLGDFIFYSVLVGKAAATPSGDWNTTLACFVAILIGLCLTLLLLAVFK---KALPALPISITFGLIFYFSTDNLVRPFMDTLASHQLYI

>Hsa2

ELTLKYGAKHVIMLFVPVTLCMIVVVATIKSVRFYTE--KNGQ--LIYTPFTE--DTP-SVGQRLLN-SVLNTLIMISVIVVMTIFLVVLYKYRCYKFIHGWLIMSSLMLLF---------LFTY-IYLG-------EVLKTYNVAMDYPTLLLTVWNFGAVGMVCIHW-KG-PLVLQQAYLIMISALMALVFIKYLPEWSAWVILGAISVYDLVAVLCPKGPLRMLVETAQERNEPIFPALIYSSAMVW-TVGMAEERG--VKLGLGDFIFYSVLVGKAAATGSGDWNTTLACFVAILIGLCLTLLLLAVFK---KALPALPISITFGLIFYFSTDNLVRPFMDTLASHQLYI

>Hsa1

ELTLKYGAKHVIMLFVPVTLCMVVVVATIKSVSFYTR--KDGQ--LIYTPFTE--DTE-TVGQRALH-SILNAAIMISVIVVMTILLVVLYKYRCYKVIHAWLIISSLLLLF---------FFSF-IYLG-------EVFKTYNVAVDYITVALLIWNFGVVGMISIHW-KG-PLRLQQAYLIMISALMALVFIKYLPEWTAWLILAVISVYDLVAVLCPKGPLRMLVETAQERNETLFPALIYSSTMVW-LVNMAEERG--VKLGLGDFIFYSVLVGKASATASGDWNTTIACFVAILIGLCLTLLLLAIFK---KALPALPISITFGLVFYFATDYLVQPFMDQLAFHQFYI

**STRAWBERRY NOTCH**

>Oca

----------------------------------------------------------------------------------------------------------------------------------------------------------------------------------------------------------------------------------------------------------------------------------------------------------------------------------------------------------------------------------------------------------------------------------------------------------------------------------------------------------------------------------------------------------------------------------------------------------------------------------------------------------------------------------------------GIEVLLQNALFAYFMDTLAAVIEQAKKMGRWDEGILDVGSRGENVLKEKTEVFVERGMKWEEAGFYANVGKFHKLSES-EARSLWEDQFVASLSQCTHLYSRGRCKNVTVGLKCEFGKRRRSYYILAGSVLSVWTEVEQVVRLK-TTDGSKIVG----

>Aqu

EEED---DHYTSADTYATYKPAKLKLGYLHPDPIVESSSLASVEPPQVYYDL--KYPENVIEEVRLSSLQLEAVVYACQQHMNTLADGSRAGFLIGDGAGVGKGRTVAGIIYQNYIEGRKKSLWLS-VSNDLKYDAIRDLHDVGAKK-ISVFALNKFCYGVIFATYSSLIGESTRFTQLLHWL-GPQFDG--VIVFDECHKAKNLVPSGASKPSKTGITVLQLQKRLPKARIVYCSATGASEPKNMAYMSRLGIWGEGTQFPAFQDFIKSVERRGVGAMELVAMDMKLRGLYIARQLSFTGVSFSVEEVSLSKKFIDMYDKAVAFWVEARQMFKDAADLLDYDGRNL-KTMWGQFWAAHQRFFKYLCIAAKVPSVIDLAMQSLKDG-KCVVIGLQSTGE-----ARTLEQLGQSHG------ELDGFVSTAKGVLESLIKRHFPKEELLRKLNELAPLLPSNSLDELIDGLGGPSKVSEMTGRRGRVVQMSDGS-VRYQLRNITEKTRFMDGEKEIAIISEAASS-----GISLQADRRAKNQKRRVHITLELPWSADKAIQQFGRTHRSNQVTSPEYVFLISTLSGEHRFASIVAKRLECLGALTHGDRRA-TESRDLSRYNIDTKYGRQALSPDYNQD---FFDKSHEAMVDVEKESLNMTRFLNRLLGLPVKLQNLVFCYFTDTLGEVIKRAKKAGKWDGGILDFGASGEHVDLVESKEFVERGMSFEDAGFYPNTGKYKRC-DPEDAMKWWTLQYESTLTSCSHSYWQGKCRSSTPTHPCEVGMRRRTYYVLAGSLLGIWIHLEQIVRVH--TSQKKIIGILIP

>Cin

---------MQHTHTYALYMPSKLNIGHPHPDSVVETSSLSSIMPPDIKYQL--SIPQYTIDHCLLSALQLESVVYACQKHQMFLPNGERAGYFIGDGPGVGKGRTVAGVIYENYLLGRKRALWFS-VSNDLKYDAERDLRDIGAKN-IKVYSLNKFKYGVIFGTYSSLIGESTRLKQILNWV-GEDFDG--VIIFDECHKAKNLVPSGSGKSTKTGHTVLQLQTHLPNARVVYASATGASEPKNMAYMSRLGLWGSGTPFPQFTDFIQAVERRGVGAMELVAMDMKLRGSYIARQLSFYGVSFRIEEVPLNNSFISMYNKAVKLWMLARAAFQAAASMIEAEYHMK-KSMWAQFWSAHQRFFKYLCLSAKVDYTVSLVREAVKHG-KCVVIGLQSTGE-----SRTLDQLEKCNG------ELDDFVSTAKAVFETLIENHFPKSELLEKLETLGSCLPNNMLDELIDSLGGPENVAEMTGRKGRVIATDEGN-VEYQTRNMTEKQRFMDGEKNIAIISEAASS-----GISLQADKRARNQRRRLHITLELPWSADRAIQQFGRTHRSNQVNAPEYVFVITELAGEHRFASVVAKRLESLGALTHGDRRA-TESRDFSKYNYDNKYGRKALPKNYKGN---FFKDMEQALQGVGKDYNNIFKFMNRMLGMEVEHQNGLFLFFTQTLNAIVQDAKRSGQFDQGIVDLGAENQIVEVLKTKEFIERGISWVDAGFYPNTGKYKRV-ESDEAEVWWCKHHEASLDQCSHRFWQGSCSKGNS--LCHIGKRRRMYHVLAGSVLAVWRTVEQVVRLK-TQEGNRIVGVLIP

>Spu

-------------------------------------------------------------------------------------------------------------------------------------------------------------------------------------------------------------------------------------------------------------------------------------------------------------------------------------------------------------------------------------------------------------------------------------------------------------------------------------------------------------------------------------------------------------------------------------RTHRSNQVTAPEYVFLISELAGEQRFASIVAKRLESL----------------------------------------------------------------------------------------------------------------------------------------------------------------------------------------------------------------------------------

>Hro

EVEE---EELGHAETYSDYMPTKLKMGCKHPDPVVESASLSSVESPDVTYCL--SIPDICIERGLLSAVQLESITYACQKHESLLPNGERVGFLVGDGAGVGKGRMIAGIIYENYLCGRRRSLWFVLVSNDLKVDAERDLRDIGASE-IEVHSLNKFKYGVIFATYSSLIGESTRLKQLIQWL-GKEFDG--LIIFDECHRAKNLFPNGSSKPTKTGQTVVELQNKLPKSRVVYASATGASEPRNMAYMVRLGLWGPGTPFREFSDYIQAVEKRGVGAMEMVAMEMKQRGTYVARQLSFKGVSFHVEEVEISEEYINVYNKSVQLWVEARDKFQQAAELLGIENRVH-KTMWGQFWSSHQRFFKYLCISVKVQHCVSLANAAIAKG-KSVVIGLQSTGE-----SRTLEMVDEKGD------ELNDFVSTAKGVFQKLVESHFPKQQLLDKIELLGRVLPANALDDLIDKLGGTSHVAEMTGRKGRIVCLKDGT-IRYERRNLTEKQRFMDGEKDIAIISEAASS-----GISLQADRRVANQKRRVHITLELPWSADRAIQQFGRTHRSNQVSAPEYIFLISELAGERRFASTVAKRLECLGALTQGDRRA-AETRDLSQFNIDNKYGQTALPSHYSGN---FFHDVQKSLVGVGKESYNISKFLNRILGIEVETQNALFEYFTSTMKAIVMKAKRDGKWDLGILDLGSSGEKVVEQSCRDFVERGLSWADAGFYANTGKFNKVFDAERVKQSWEEQYNFSEHHCLHVFQYGQCHKAST---CEIGRRSRIYHVLSGSVLSVWTSVEQIIRLR-TTNNRRIVGTLIH

>Lgi

EEAEEEVQELGHAETYFEYMPSKLKIGEKHPDPVVETSSLASVEPVNVKYKI--VIPESTIDMCQLSALQLEAITYASQRHETILPSGERAGFLIGDGAGVGKGRTIAGTIFENYLQGRKRAIWLS-VSNDLKVDAERDLRDIGAGK-IEVYALNKLKYGVVFSTYSSLIGESTRLKQLLKWC-GKDFDG--LIVFDECHKAKNLCPVGSSKPTKTGRTVLELQNHLPLARVVYASATGASEPKNMAYMTRLGIWGPGTPFKEFNDFIQAVEKRGVGAMELVAMDMKLRGMYIARQLSFKGVSFKIEEVTIDNSFQKIYNKAVDLWVDAREKFQKAADLIDAEHRLR-KSMWGQFWSAHQRFFKYLCISAKVNYCVHIAREAVKNG-KCVVIGLQSTGE-----ARTLEQLEESGG------ELNDFVSTAKGVFQTLVEKHFPKSDLLRKMEEIGNTLPSNALDQLIDELGGTDCVAEMTGRKGRIVSNEDGS-INYESRNLTEKQRFMDGEKDIAVISEAASS-----GISLQADRRAVNQKKRVHITLELPWSADRAIQQFGRTHRSNQIHAPEYIFLISELAGERRFASTVAKRLESLGALTHGDRRA-TETRDLSRFNIDNKYGRAALPSNYSGN---FFSDVKKGLVGVGKDYNNISKFLNRILGMDVELQNGLFKYFTETLTAIILEAKRNGRWDMGILDLGSGQEKVRRIETQSFVERGMTWSAAGFYPNTGKYKKV-LPDQCQFWWEDQYYFSAKSCIHSYWRGNCKKVSLGLPCEIGLRTRTYHVLSGSVLSVWSKVEQIIRLR-TEDGQKIVGNLIP

>Bfl

EEEEEEEEELGVAETYAEYTPSKLDIGLGHPDSVVETSSLSSVQPPDVWYKL--AIPEHIIDYGYLSALQLEAITYACQQHEIFLQSGERAGFLIGDGAGVGKGRTIAGIIYENYLLGRKRSLWLS-VSNDLKVDAERDLKDIGAK--VSVHSLNKFKYGVTFATYSSLIGESTRMQQILHWC-GEDFDG--VIVFDECHKAKNLCPVGSSKPTKTGLTVLELQNRLPKARIVYASATGASEPKNMAYMSRLGIWGEGTPFREFNDFIQAVERRGVGAMEIVAMDMKLRGMYMARQLSFAGVSFKIEEIPLEDEFITMYDAAVKLWVDAREYFQKAAELIDAEHRMR-KSMWGQFWSAHQRFFKYLCIASKVKPAVRIAREAVKSG-KCIVIGLQSTGE-----ARTLEELENQGG------ELNDFVSTAKGVFQTLIEKHFPKKELLDRIEKLGGILPPNTLDELIDDLGGPENVAEMTGRKGRVVSNDEGT-ICYESRNLTEKQRFMDGEKNIAIISEAASS-----GISLQADKRVQNQRRRVHITLELPWSADRAIQQFGRTHRSNQVYAPEYMFLISELAGERRFASIVAKRLESLGALTHGDRRA-GEARDLSRFNIDTKYGRTALPEDYQGN---FFHDILHGLIGVGKDYNSMPKFLNRILGLTVNLQNGLFAYFLDTLHEVIQRARRDGRYDMGILDLGSGGDHVRLLETKRFVERGVSWKEAGFYPNTGKYKKV-TPDEAEPHWVSQYNSSLAQCSHAYWRGNCKRVSLGLSCEIGLRRRTYHVLSGSVLQVWSKVEQVVRIK-TQQGKKVVGTLIP

>Vca

-------------------RPAKVTEGPPHPDPIVETASLASVTPPDITYKH--HLHDN-LERAELSNAQLETVLYAFQRFERRLPDGNRAGFFLGDGAGVGKGRQIAAVIKEFWASGGRRVLWVS-TSNDLRYDARRDLSDLGRKDSVPAGNLDRI-YGVLFITYSLLRAEVSRLHQIVSWLGGPKGDGECLIVLDECHKAKNLLDAAGSD--QTGLAVESLQDQLPNARVLYSSATGASEPDNLRYMVRLGAFD----YPHIGDMINALKKSGLGALEMFCMGLKATGTYVSRTLSYKGAEFRTEELEIDPIFSVMYDRSCWLWSLVYNVMRSLPKSKNARGRDMKASL---FWGAHQRFYRQMLIASKVCRCAELAKEALDRG-MCVVIGLQSTGEANLNSARESAAAGSGGGGGGEDDSLEDFVSAPKMILHSAVE----QRDLADH-----ERFPLNPLDHLTSLLGGESQVAEMTGRKVLQVRNDDGR-IQ----NIAEKNDFMSGRKLVAIISDAAST-----GISLQADRRVPNQRRRFHITLELPWSADKAIQQFGRSHRSNQASAPEYCLLVTKCGGEYRFAGAVAKRLTSLGALLRGDRRALGASTDLKPYDVDNKYVRLYDEAGRQGT--------RRGYKPKG----FFFAYLHMVPPLPLICHFALPCFFLP----------------------------------------------------------------------------------------------------------------------------------------------

>Ath

EVEREEDEGGTAGETFMDYRPPKLSIGPPHPDPIVETSSLSAVQPPEPTYDL--KIKEELERSKALSCLQIETLVYACQRHLQHLADGTRAGFFVGDGAGVGKGRTIAGLIWENWKHGRRKALWIS-IGSDLKYDARRDLDDVGATC-VGVNPLNKLPYGVVFLTYNSLIASSSRLQQLVQWC-GPEFDG--LLIFDECHKAKNLVPEAGSQPTRIGQAVVDIQDKIPQARVIYCSATGASEPRNMGYMVRLGLWGAGTSFSDFNKFLGALDKGGTGALELVAMDMKARGMYVCRTLSYKGAEFEIVEARLEAGMEAMYNKSAEFWAELRIELLSASAFLPNE-KPNSSQLWRLYWSSHQRFFRHLCMSAKVPVTVRLAKKALSTN-KCVVIGLQSTGE-----ARTEEAVNKYGL------ELDDFVSGPRELLLKFVEENYPKSKIIEIIRSLN--LPNNPLDDIVDQLGGPEKVAEMTGRRGMLVRASNGKGVTYQARNMHEKQLFMDGKKLVAIISEAGSA-----GVSLQADRRAVNQKRRVHLTLELPWSADRAIQQFGRTHRSNQTSAPEYRLLFTNLGGERRFASIVAKRLETLGALTQGDRRK------VPFLTISYNFGKKSLSIDEPETVKEFLTKARAALVAVGSDMHDVGRFLNRLLGLPPDIQNRLFELFTSILDVLVHNARIEGSFDSGIVDMKA--NSVELLSTPKTLDRGVTWESAGFFPAVGKYRKLSSLEKARTGWEDEYEVSSKQCMHG---PKCK---LGEYCTVGRRIQEVNVVGGLILPIWGTIERVIRIETTTDNQRIVGLSIP

>Tad1

ESDSEDEDEQHRTETYSQYVPSKFKSGKRHPDPVVETSSLSSVLPPDITYNL--NLPKSVIANGMLSSLQLEAVVYACQQHNTWLADGRRAGFLIGDGAGVGKGRTLAAIIYENYIEGRKRALWYYSVSNDLKYDSARDLKDIGAER-IKIYSLNKFKYGVIFATYSSLISESTRLSQIIRWL-GESFDG--VIIFDECHKAKNLYRSASSKPTKTGKTVLALQNKLPKARIVYASATGASEPRNMAYMNRLGIWGKGTPFQEFENFIHAIDKGGVSAMELVAMDMKMSGVYMARQLSFTGVTFNIQEIPLSHDFKEMYNKSVKLWVEARQKFSQAATIMGADSKLH-KNMWGQFWSAHQRFFKYLCIAAKVKVAVDIAKDAVANG-KCVIIGLQSTGE-----AQMMEQLGESNG------EIDDFISTAKGVFTALIEKHFPKEQLLSSFEELAPKLPPNTLDELIDSLGGPKNVAEMTGRKGRVVCNADGT-ISYQSRNLIEKKRFMDGEKLIAVISEAASS-----GISLHADKRVANRRRRVHITLELPWSADRAIQQFGRSHRSNQVSAPDYLYLISELAGEQRFASIIARRLESLGALTHGDRRA-TETRDFSKYNFDNRYGRTALPENYEGD---FFNDISEAMADVGKDYNSIPKFLNRILGIPVVLQNKLFAYFSDTLNKIIQESKKTGRWESGILDFGTDDMTVEKVQSIQFLTRGLSWEKVGFYPNTGKYRKS-SKTAAKSVWIDQYEASLTLCSHAFWKGNCKKVAAGLACEVGLRCRTYHVFSGSVFSVWNCIEQIVRLT-TKEDEKIVGEYQT

>Cel1

EDDE---ENLGYAETYSEYTPAKLRSGMAHPDSVVESASLSSVSPPDVKYQI--SIPEYLIDMGHISALQLEAVIYACQMHERRMPSGERYGYLIGDGAGVGKGRTVACIIFENYLQGRKRAIWLS-VSSDLKFDAERDLRDCGAPN-IPVYALNKMKYGVMFATYTSLIGECSRISQLIQWF-GQDYDG--VIILDECHRAKNLVPTAGAKPTKTGRMVLELQKALPNARVVYASATGATEPRNMAYMTRLGLWGERQAFPEFHDFISAVERRGVGAMEIVAMDMKQRGLYLARQLSFRGVSFAVQEVQLSSEFVKMYDAAVKLWMEARRQFQTVIETMDEEERSTCKTVWGQFWACHQRFFKYLCIAAKVDTCVQLSREAIKAK-KCVVIGLQSTGE-----SATLETLEEMGG------ELNEFVSTAKTVLYGLIDKHFPKTELLAAVERLAPSLPANTLDQLIDEMGGPEYVAEMTGRRGHMVTSETGD-VMYQRRNMEEKEKFMRGEKLIAIISEAASS-----GISLQSDRRAINKRRRVHITLELPWSADKAIQQFGRTHRSNQVSGPEYVFLISELAGEKRFASIVAKRLESLGALTHGDRRA-TETRDLSQFNMDNKYGRVALPKDYKAG--EFFEDMRLYMEGVGKEAATIPKFLNRILGLPVHAQNSLFHYFSEIVAELIAQSKHDGTYDTGIMDLGTGDDQVRKLETRVFVERGVSWEEAGFYPSTGRFHKV-SIDEAKEVWKQQYDSAANMCQHNYVYGKCRTESNGTYCEVGRRTRTYFVLSGSVLSVWPIVEQVIRVR-TEQDQKIVGKLVV

>Cel2

EDDE---ENLGYAETYSEYTPAKLRSGMAHPDSVVESASLSSVSPPDVKYQI--SIPEYLIDMGHISALQLEAVIYACQMHERRMPSGERYGYLIGDGAGVGKGRTVACIIFENYLQGRKRAIWLS-VSSDLKFDAERDLRDCGAPN-IPVYALNKMKYGVMFATYTSLIGECSRISQLIQWF-GQDYDG--VIILDECHRAKNLVPTAGAKPTKTGRMVLELQKALPNARVVYASATGATEPRNMAYMTRLGLWGERQAFPEFHDFISAVERRGVGAMEIVAMDMKQRGLYLARQLSFRGVSFAVQEVQLSSEFVKMYDAAVKLWMEARRQFQTVIETMDEEERSTCKTVWGQFWACHQRFFKYLCIAAKVDTCVQLSREAIKAK-KCVVIGLQSTGE-----SATLETLEEMGG------ELNEFVSTAKTVLYGLIDKHFPKTELLAAVERLAPSLPANTLDQLIDEMGGPEYVAEMTGRRGHMVTSETGD-VMYQRRNMEEKEKFMRGEKLIAIISEAASS-----GISLQSDRRAINKRRRVHITLELPWSADKAIQQFGRTHRSNQVSGPEYVFLISELAGEKRFASIVAKRLESLGALTHGDRRA-TETRDLSQFNMDNKYGRVALPKDYKAG--EFFEDMRLYMEGVGKEAATIPKFLNRILGLPVHAQNSLFHYFSEIVAELIAQSKHDGTYDTGIMDLGTGDDQVRKLETRVFVERGVSWEEAGFYPSTGRFHKV-SIDEAKEVWKQQYDSAANMCQHNYVYGKCRTESNGTYCEVGRRTRTYFVLSGSVLSVWPIVEQVIRVR-TEQDQKIVGKLVV

>Hma2

--------------------------------------------------------------------------------------------------------------------------------------------------------------------------------------------------------------------------------------------------------------------------------------------------------------------------------------------------------------------------------------------------------------------------------------------------------------------------------------------------------------------------------------------------------------------------------------------------------------------------------------------------------------------------------------------------------------------------------------------------------------------KYEKV-KPSDVEVIWNEQYEYSEKNCSHAFLRGYCKRKIVGLPCEVGTRKRTCYILCGSVLSVWSKVEQINRLK-LDNGVKLVGMSIP

>Hma1

--------------------PTR----------------------------------------------------------------------------------------------------------------------------------------------------------------------------------------------------------------------------------------------------------------------------------------------------------PVKLWPDARDKFETAADIVGLDSKGK-KTMWGQYWSAHQRFFKYLCIAAKVKSAVEIAKEAIEKG-KCILFGLQSTGE-----ARTLDQLDEMGG------ELNDFISTTKGVFSTLVEKHFP--------------------------------------------------------------------------------------TVDSKANHNNTDCKRK---------------RDWKKSHKQK--------------------------------------------------------------RRRYQDG----------------------------------------VEFMSSSDSSDSENESKNEASDLILSDSS------------------------DFS--------LSESDE--DAWMK----------------------------------------------------------DSSEKKI------

>Mbr

EEEGQEEEGHDLSEVFVPYRPQETGWGKPHPAQIVEPASLASVHLPKATYPLRKSLPASVIDDGKLSALQLEGVMYACMQHERILPNGYRRAILIGDGAGVGKGRQIAGILFENLLHERKRHIWFS-ISTDLCADAERDLRDIGCQV-NVIDGCQE---GVLFSTYATLVSPGSRYEQLVQWC-GPEFDG--CIVFDECHKAKNLHLEKPGASSKVALRVNDLQQAFPKARVVYCSATGVTDLRNLAYMDRLGMWGPGQAFANFEDFKREMSQNGIAAAEMLAMELKAQGVYLSRTLSYESAEFNNCDISLTEAQMDLYNRAADFW----GRLYRAASRLGKVPR--------AYYAAHQRFFKQLCLSIKVPQLVARVKEALASGDHCVVIGLQSTGE-----ARLQAAFARESG------YRNSLVSLTEEIARAFIESTFPGAQLLEELKNL--RLPPSPLDDLIDQLGGPSAVAELTGRKQRQVRVGHTSSFVIEERNIVEKNAFLDGRKLIAIISDAASTGLMSLGISLHADVRRENQHRRVHFTFELPWSADKAVQQLGRSHRSNQACGPRYELMSTELAGERRFAAAVARRLRFMGALTRGDRRA-ASGQDMGSWDIDTNYGRLALEGRLRARVCQLFPTDDALFQGLKQAFKSVSAFLNGVLGLPVEGQNIMFGALMVLHRRTIERAKEEGKYSEGIHEI-TGD-KIQLVSERDIRDRGMSFEEARFMPNIG-YNAV-EPSAVKSTWQRIHSEAERLCLHG---PKCTIPN----CSFGKRRQRFHILSGRLVKYWKTLSNIARC--SLGQRSFIGLLWP

>Tad2

EINRIADEDYGQEKNREVFLPYRITNYKSHPGNVVEAASLAAQILPPTNYPLKDALPPHLILDCKLSDLQLEGILHACQRHQMILPKGARAGFYLGDAAGVGKGRQIAGIIINNYVRGRRKHLWFS-TSHDLRIDAERDLKDINCFI-KVIDGCQELDRGILFNTYATLISSGSRIQQILDWC-GDNFNG--CLIFDECHKAKQFIPGNEAASTKAAVAAIALQRQLPKARVVYCSATGVTNVKNMAFMERLGLWGDNTAFKNVENFIQAMTTRGLGASEMLAMEMKAAGMYVSRGLSFESAEFHTVEINLTDEQIRVYNLSARVWQQLAEAIKKAISHTKSSA----SRIWTVYWSCHQRFFKQLCMSLKVPAIVREAKAALDEG-YCVVIGLQTTGE-----ASLDNEITRQGN------NTHGYISTAKEILVRFILQHFP-DALLQFIDNIN--LPNCSLDELIQQLGGTNCVAEMTGRRGRIVCINGSSTPVYQLRNVREKNLFMQGKKLIAVLSDAAST-----GISLHSDTRVANNRRRIHITMELPWSAEKAVQQLGRSHRSNQSSAPIYKLMTTNVGGEKRFVAAVAQRLLSLGALTRGDRRA-ATGADFSEFNFNTSYGRSALLDDSSYTISQFYIDMKHCLLDMGKDATDVQRFLNRILGLTIERQNLIFSYFCQCLTTSIERAKKEGKYNEGVTDI-KGTSIVMVDSPKEIVDRGIDWPTVGFYPNTGKYHKL-DLSDAENGWKEQYNSTELACIHG---GSCK---FGSYCTVGIRINRVHIICGGIIPMLSLLERIVRVQ-LDDGRKIVGLRYP

>Aae

EEEAEE-EEMGVAETYAEYWPAKLKIGKKHPDPVVETASLSSVEPSDVYYKI--SIPPEPINAGQLSALQLESITYASQAHDHLLADGSRAGFLVGDGAGVGKGRTIAGIIYENYLKGRKKSIWIS-VSNDLRYDAERDLRDIGASR-INVYALNKLKYGVIFGTYSALIGESSRLKQLLQWC-GEDFDG--VIVFDECHKAKNLCPVGSSKPTKTGLTALELQNKLPKARVVYASATGASEPRNMAYMVRLGIWGMGTPFPNFMDFITAVEKRGVGAMEIVAMDMKLRGMYIARQLSFHGVTFKIEEVPLTKDFKRVYDESVELWVVAMQKFTEAAELIDAENRMK-KTMWGQFWSAHQRFFKYLCIASKVNHAVRVAREAIKYG-KCVVIGLQSTGE-----ARTLEQLERDDG------ELTDFVSTAKGVFQSLVEKHFPKDELLAKIERLGDRLPANTLDQLIDELGGPENVAEMTGRKGRVVQTDDG--VQYESRNITEKQRFMDGEKDVAIISEAASS-----GISLQSDRRVRNQRRRVHITLELPWSADRAIQQFGRTHRSNQVNAPEYMFLISDLAGERRFASTVAKRLESLGALTHGDRRA-TETRDLSQFNIDNKYGRSALPSDYKGD---FFKDIAGALVGVGKDYNNISKFLNRILGMPVELQNRLFKYFTDTLAAIIEQAKKRGRFDLGILDLGAAGENVTRIKITKFVERGMIWQEAGFYPNTGKYKKV-LSDEAEPHWTKQFDASVNTCSHSYWKGMCRYVTMGQDCEIGLRRRTYFVLSGSVLSVWSRVEQVIRLK-TKEGSKIVGTLIP

>Nve3

---------------------------------------------------------------------------------------------------------------------------------------------------------------------------------------------------------------------------------MLPLARVVYCSATGVTDVKNMAFMERLGLWGEGTAFKSFESFLESITKRGLGTKEI----------------------------------------------------KTSLEYALARTNTTNTRIWSLFWSSHQRFFKQLCMSMKVPLIVKEAKAALNDG-YSVVIGLQTTGE-----ASLESELNRNGG------RINRFISICEEIMTRFITQYFPKSLLLGFVKKIN--LPISPLDDLIDKMGGPDCVAEMTGRRGRVVRTAQDREPHYEARNIQERNSFMSGSKLVAIISDAAST-----GISLHADLRAANQRRRVHVTIELPWSADKAVQQLGRSHRSNQSSGPIYKLVTTNLGGERRFAAAVARRLQSLGTYT------------------------------------------------------------------------------------------------------------------------------------------------------------------------------------------------------------------------------

>Nve2

AEDDEEEEELGHTDTYAEYMPCKRKLNIRHPDSVVETSSLASVTPPNIWYKL--KLPPEVIDRTFLSALQLEAVVYACQQHEIFLESGERAGFLIGDGAGVGKGRTVAGIIYENYLLGRKRAIWLS-VSNDLKYDAIRDLRDIGC-H-IHVNALNKFKYGVIFATYSSLIGESTRLKQLLHWC-GSDFEG--VIILDECHKAKNLVPTGSSKPTKTGLTVLALQNSLPKARVVYCSATGASEPKNMAYMSRLGIWGPGTPFPDFTDFIRAVERRGVGAMEIVAMDMKLRGMYMARQLSFAGVGFNIRDIKLEQKFINMYDKAVLLWNEAREKFEKAADLMMADNRTK-KTLWGQFWSAHQRFFKYLCIAAKVHEAVRMAHEAIDLG-KCVVIGLQSTGE-----ARTLEQLEEAGG------ELNDFVSTAKGVLQTLIEKQFPKKELLDKLDTLGPLLPPNTLDELVDSLGGPDKVAEMTGRKGRVVSHEDGR-VXYESRNVEEKKRFMNGEKLVAIISEAASS-----GISLQADRRAQNQRRRVHITLELPWSADKAIQQFGRTHRSNQVSAPEYVFLISELAGERRFASVVAKRLESLGALTHGDRRA-TESRDLSRYNFDNKYGRAALPSSYKGN---FFQDVRRALFGVGKDYTNMSRFLNRILGMEVVLQNELFSYLMDTFTAVIQQAKRSGRWDEGILDLGSHGEEVNELSVQTFVERGMSWDDAGFYPNTGKYKKV-TAPEAQQHWCSQYDSSLSLCSHAYWKGNCKRATLGLWCEVGLRRRSYNILSGSVLSVWSKVEQIIRCK-RSDGKKIVGCLIP

>Nve1

-------RDLHDIDCYVK-----VIEGCQHLD---------------------------------------------------------------------------------------------------------KETKVFGLPP------------GVVFSTYATLVSSVTRLQQLMDWCGGETFDG--CLIFDESHKAKHFIPGKEENSTKIALAVTTLQRMLPLARVVYCSATGVTDVKNM------------------------------------------------------------------------------------------------------------------------------------------------------------------------------------------------------------------------------------------------------------------------------------------------------------------------------------------------------------------------------------------------------------------------------------------------------------------------------------------------------------------------------------------------------------------------------------------------------

>Dre1

---------------------------------------------------------------------------------------------------------------------------------------------------------------------------------------------------------------------------------------------------------------------------------------------------------------------------------------------------------------------------------------------------------------------------------------------------------------------------------------------------------------------------------------------------------------------------------------------------------------------------------------------------------------------------------------------------------------------------------------------------VDRGMPWKEAGFYPNIGRYYKV-LPEDAKDVWESQFDFSFTNCSHANWKGKCKQIEEGQECFLGMRLRQYHMLCGALLRVWKRVSQIVRLK-TKQNSKQVGIKIP

>Dre2

EADDEETEELGHADTYAEYKPSKSTIGISHPDIVVETNTLSSVPPPDITYTL--SIPDSTVSTGLLSALQLEAIIYACQQHEVILQNGQRAGFLIGDGAGVGKGRTVAGIILENFLKGRKRALWFS-VSNDLKYDAERDLKDIDAPN-IPVFALNKIKYGVLFATYSALIGESTRLKQILDWC-KPSFDG--VIVFDECHKAKN------ATSTKMGKAVLDLQSKLPRARVVYASATGASEPKNMIYMSRLGIWGQGTPFKTFDDFLHAIEKRGVGAMEIVAMDMKVSGMYIARQLSFSGVSFRIEEIALDEEFKLVYNKAARLWAEALELFNRAADTLGLASR---KSLWGQFWSSHQRFFKYLCIAAKVRRLVDLAHMEMQQG-KCIVIGLQSTGE-----ARTREVLDENDG------HLDKFISAAERVFQSLVQKHFPKQRLLHRIAELGKELPLNTLDELIDRFGGPEKVSEMTGRKGRVVRRPDGS-VRYESRNVREKERFMSGEKLIAIISEAASS-----GISLQADKRVQNRRRRVHMTLELPWSADRAIQQFGRTHRSNQVTAPEYIFLISELAGERRFASIVAKRLESLGALTHGDRRA-TESRDLSQYNFENKYGTKALPKGYPGGGLMFFRDMKKGMIDVGKD-CTLTKFLNRILGLEVHHQNFLFQYFTENFDYLIEKDKKEGKYDMGILDLAPGNDEIHEETQEKFIGRCIIFD-------------------------------------------------------------------------------------------------

>Xtr1

EEEDEDTEELGHTETYADYVPSKSKIGRQHPDRVVETSTLSSVPPPDITYSL--SLPSDVIEQGLLSALQLESIVYACQQHEVILPSGQRAGFLIGDGAGVGKGRTVSGIILENFSKGRKKALWFS-VSNDLRCDAERDLRDINAGH-IAVHALNKIKYGVLFATYSALIGESTRIKQIRDWC-GEKFDG--VIVFDECHKAKN------ASSTKMGRAVLELQNNLPMARVVYASATGASEPKNMIYMSRLGIWGEGTSFSTFDDFLHAIEKRGVGAMEIVAMDMKVSGMYIARQLSFSGVTFRIEEIPLEESYKHMYNRAAQLWAEALVVFQTAADWLGLESR---KSLWGQFWSAHQRFFKYLCIASKVQRLVTLVKQELAKG-KCVVIGLQSTGE-----ARTREVMDENDG------HLDCFVSAAEGVFRSLILKHFP------------------------------EKAMQKRKRRGK------------------RSGKMSRGDLGLIRISEESSS--------------------------------------------------------------------------------------------------------------DSEGG----------------SDFNSSPE---------------------------------------------------------------------VIY--------------------------------------------------------------------------------------------

>Xtr2

EEEDEDTEELGHTETYADYVPSKSKIGRQHPDRVVETSTLSSVPPPDITYSL--SLPSDVIEQGLLSALQLESIVYACQQHEVILPSGQRAGFLIGDGAGVGKGRTVSGIILENFSKGRKKALWFS-VSNDLRCDAERDLRDINAGH-IAVHALNKIKYGVLFATYSALIGESTRIKQIRDWC-GEKFDG--VIVFDECHKAKN------ASSTKMGRAVLELQNNLPMARVVYASATGASEPKNMIYMSRLGIWGEGTSFSTFDDFLHAIEKRGVGAMEIVAMDMKVSGMYIARQLSFSGVTFRIEEIPLEESYKHMYNRAAQLWAEALVVFQTAADWLGLESR---KSLWGQFWSAHQRFFKYLCIASKVQRLVTLVKQELAKG-KCVVIGLQSTGE-----ARTREVMDENDG------HLDCFVSAAEGVFRSLILKHFPKMELLAKVETLGKELPLNTLDELIDQFGGPEKVAEMTGRKGRVVRKSDRT-VQFESRNLREKERFMNGEKLVAIISEASSS-----GISLQADKRVPNKRRRVHMTLELPWSADRAIQQFGRTHRSNQVSAPEYVFLISELAGEKRFASIVAKRLESLGALTHGDRRA-TESRDLSKYNFENKYGTRALPRDYPGSSKEFFQEIKQGLLSVGKE-CSVTKFLNRMLGLEVHKQNSLFQFFSDTFDYLIEKDKKEGKYDMGILDLAPGVDEIYQESKETFVDRGMSWAKAGFYPNIGKYHKV-SPEEAMEHWEDQYNFSLHNCSHTMWNGNCKGLQQGKKCVQGLRLRYYYMLCGSLLRVWSRMAQIVRLK-TKDKHKQVGIKIP

>Gga2

EGDEEETEELGQTETYAEYIPSKSKIGKHHPDLVVETSTLSSVPPPDITYSL--SLPSSVADKGSLSALQLEAIIYACQQHEVLLPNGQRAGFLIGDGAGVGKGRTVAGIIFENYLKGRKKALWFS-VSNDLKYDAERDLKDIEASH-IPVHALNKIRYGVLFATYSALIGESTRLKQILDWC-RENFDG--VIVFDECHKAKN------ASSTKMGKAVLDLQNKLPQARVVYASATGASEPKNMIYMSRLGIWGEGTPFRAFDEFLHAIEKRGVGAMEIVAMDMKVSGMYIARQLSFTGVTFRIEEIPLDQQYKIVYDKAAKLWAEALMVFQQAADLIGLESR---KSLWGQFWSAHQRFFKYLCIAAKVRRLVELAKEELAKD-KCIVIGLQSTGE-----ARTREVLDENDG------HLNCFVSAAEGVFLSLIQKHFPKQDLLAKVKALGKELPLNTLDELINHFGGPEHVAEMTGRKGRVVCRPDGS-VMFESRNLKEKERFMNGEKLVAIISEASSS-----GISLQADRRVKNQKRRVHMTLELPWSADRAIQQFGRTHRSNQVSAPEYVFLISELAGERRFASIVAKRLESLGALTHGDRRA-TESRDLSKYNFENKYGAKALPKSYERGEAAFFQEMKQGLISVGKD-CSITKFLNRILGLEVDKQNMLFQYFSDTFDYLIEKDKKEGKYDMGILDLAPGIDEIYEESKEVFVDRGLKWEEAGFYPNIGKYRQV-TPEEAKEHWESCYHFSLKKCNHAVWNRSCKLIQEGKECFQGMRLRHYYMLCGALLRVWSRIAQIVRLK-TKEKKKQVGIKIP

>Gga1

EPEEEDEEEMGHAETYAEYMPIKLKIGLRHPDPVVETSSLSSVTPPDVWYQT--SISEETIDNGWLSALQLEAITYAAQQHETFLPNGDRAGFLIGDGAGVGKGRTIAGIIYENYLLGRKRAVWFS-VSNDLKYDAERDLRDIGAKN-ILVHSLNKFKYGVIFATYSSLIGESTRLKQLLHWC-GEDFDG--VIVFDECHKAKNLCPVGSSKPTKTGLAVLELQNKLPKARVVYASATGASEPRNMAYMNRLGIWGEGTPFREFSDFIQAVERRGVGAMEIVAMDMKLRGMYIARQLSFSGVTFKIDEVLLSQEYVKMYNKSVKLWVSARERFQQAADLIDAEQRMK-KSMWGQFWSAHQRFFKYLCIASKVKRVVQLAREEIKNG-KCVVIGLQSTGE-----ARTLEALEEGGG------ELNDFVSTAKGVFQSLIEKHFPKKELLDKLEKLAEDLPPNTLDELIDELGGPENVAEMTGRKGRVVSNDDGS-ISYESRNITEKQRFMDGDKNIAIISEAASS-----GISLQADRRAKNQRRRVHMTLELPWSADRAIQQFGRTHRSNQVTAPEYVFLISELAGEQRFASIVAKRLESLGALTHGDRRA-TETRDLSRFNFDNKYGRNALPPDFPGD---FFKDVRQGLIGVGKDYNNIGKFLNRILGMEVHQQNALFQYFSDTLNAVIQNAKKNGRYDMGILDLGSGDEKVRKADVKKFVERGMSWDEAGFY------KEV-NPKKKLFWYTDQILGNNSN---------------------------------------------------------------

>Hsa2

EAEEEEAEELGHTETYADYVPSKSKIGKQHPDRVVETSTLSSVPPPDITYTL--ALPS---DSGALSALQLEAITYACQQHEVLLPSGQRAGFLIGDGAGVGKGRTVAGVILENHLRGRKKALWFS-VSNDLKYDAERDLRDIEATG-IAVHALSKIKYGVLFATYSALIGESTRLRQILDWC-GEAFEG--VIVFDECHKAKN------AGSTKMGKAVLDLQNKLPLARVVYASATGASEPRNMIYMSRLGIWGEGTPFRNFEEFLHAIEKRGVGAMEIVAMDMKVSGMYIARQLSFSGVTFRIEEIPLAPAFECVYNRAALLWAEALNVFQQAADWIGLESR---KSLWGQFWSAHQRFFKYLCIAAKVRRLVELAREELARD-KCVVIGLQSTGE-----ARTREVLGENDG------HLNCFVSAAEGVFLSLIQKHFPKQDLLDKVRRLGRELPVNTLDELIDQLGGPQRVAEMTGRKGRVVSRPDGT-VAFESRNLREKQRFMSGEKLVAIISEASSS-----GVSLQADRRVQNQRRRVHMTLELPWSADRAIQQFGRTHRSNQVSAPEYVFLISELAGERRFASIVAKRLESLGALTHGDRRA-TESRDLSKYNFENKYGTRALPQGYPGGVPTFFRDMKQGLLSVGKD-CSITKFLNRILGLEVHKQNALFQYFSDTFDHLIEMDKREGKYDMGILDLAPGIEEIYEESQQVFVDRGLKWEDAGFYPNIGKFHRV-TAEEAKEPWESGYALSLTHCSHSAWNRHCRLAQEGKDCLQGLRLRHHYMLCGALLRVWGRIAQIVRLK-TKDRKKQVGIKIP

>Hsa1

EPEEEDEEEMGHAETYAEYMPIKLKIGLRHPDAVVETSSLSSVTPPDVWYKT--SISEETIDNGWLSALQLEAITYAAQQHETFLPNGDRAGFLIGDGAGVGKGRTIAGIIYENYLLSRKRALWFS-VSNDLKYDAERDLRDIGAKN-ILVHSLNKFKYGVIFATYSSLIGESTRLKQLLHWC-GDDFDG--VIVFDECHKAKNLCPVGSSKPTKTGLAVLELQNKLPKARVVYASATGASEPRNMAYMNRLGIWGEGTPFREFSDFIQAVERRGVGAMEIVAMDMKLRGMYIARQLSFTGVTFKIEEVLLSQSYVKMYNKAVKLWVIARERFQQAADLIDAEQRMK-KSMWGQFWSAHQRFFKYLCIASKVKRVVQLAREEIKNG-KCVVIGLQSTGE-----ARTLEALEEGGG------ELNDFVSTAKGVLQSLIEKHFPKKDLLDKLEKLAEDLPPNTLDELIDELGGPENVAEMTGRKGRVVSNDDGS-ISYESRNITEKQRFMDGDKNIAIISEAASS-----GISLQADRRAKNQRRRVHMTLELPWSADRAIQQFGRTHRSNQVTAPEYVFLISELAGEQRFASIVAKRLESLGALTHGDRRA-TESRDLSRFNFDNKYGRNALPPDYPGE---FFKDVRQGLIGVGKDYNNIGKFLNRILGMEVHQQNALFQYFADTLTAVVQNAKKNGRYDMGILDLGSGDEKVRKSDVKKFVERGMSWEEAGFYPNTGKYKKV-VSDDALMHWLDQYNSSADTCTHAYWRGNCKKASLGLVCEIGLRCRTYYVLCGSVLSVWTKVEQIVRLR-TEDGQRIVGLIIP

**SUH**

>Aqu

LTREAMRQYLKDRTVVVLHAKVAQKSYGNEKRFFCPPPSLYLMGKGWKSRKKENEKKKAEGQRNCCFVGI---GN-HDQEMQHLSLEEK--FCIAKTLYISD-SD-KRKHFQLQCKVYL-GDG---RD-LGFLSQRIKVISKPSKKKQSLKNPE---LCIPSGSSVALFNRLRSQTVSTRYLHVEG----NNFHASSQQWGSFSIHLVDEDAA--EEEFTVQ---EGY--IKYGSTVKLVCSTSGMALPRLVIRKVDKQTVLLDADD-----PVSQLHKVAFYKDTER----M-------YLCLSQDRI---IQFQATQCPGEPHREINDGASWTIISTDKAEYTFSEG---------MGPVPVTPIPIAKDI-RLIGGDDLQMLEIQGEGF---------TPDLRVWFADVEAETM-YRCA---ECMAVVPPIDQF-------QKD-------SKKDDSIKVPLLIVRI-DGIIYNTHMKFQYIS--SD

>Mbr

------------KLT-ILHPRVVQKSYGNEKRYFCPPPQIHLSGRNW--------NHPQLG-RVFVSMHLNYEHT-HQTEIPLSRLA-DSEHGTASQLFIPD-TD-KRKSFCLQLKVFFQNDESNEVRDVGFPSKPLRVISKPSKKKQSNKSHSD--MYMESGCEALFFHRSRSTNAATYYFRLEE----GQPDCSDEGWHA--LKLVNDHTNVRENEANLASRNDRY--IVYDRQVRMKCAQTNKEM-VCTIHRCDRNAVVPVDNE-----CVSQLQKVAISKETE-------------YLKFQSSNGSASVFFERARSKADKGSGLSEQSIWTVCTGARLEYSFFDHTIDRFVQHIEGEMPITPVPLVDEV-KSLG----PMVELYGEHF---------STMLTVFFGPAPACTY-YRCE---ELFCKPPSYAEA-------VES---SEAVCTA--SYTVPLLLVRN-DGVIYNTFKTYKYQP--QP

>Tad

-----MRKYLAGRSIVILHAKVAQKSYGNEKRFFCPPPCVYLKGDRW------KLNRRYIKTNCRAFIGI---GN-SDQEMQPLSIE-E-NYGAAKALYISD-QD-KRKHFQLSVKLYNMDNNQIIRD-IGFNSKRIKVISKPSKKKQSLKNVD---LCIPSGSQVALFNRLRSQTVSTRYLHVDN----DVFHASSQQWGSFTIHLLDSNED--EVEFTTR---EGY--IQYGSTVKLVCTVTGLALPRLVIRKVDKQTALLDADD-----PVSQLHKVAFYKDTER----M-------YLCLSQERI---IQFQATPCPREPNREINDGASWTIISTDEAEYKFYEA---------LGPIPVTPVPVVHSL-QVIGGGNGASLELTGSNF---------SDNLKVWFGDVESETM-YR------KHCIVPDIGSF-------HGG---WQFVRQP---TKVPVTLVRS-DGVTYTTGFNFTYTP--EP

>Lgi

LTKEAMRKYLRDRILVILHAKVAQKSYGNEKRFFCPPPCIYLFGKGW------KRKHDQMEEEGCAFMGI---GN-SDQEMVQLHLE-D-KYCAAKTLYISD-SD-KRKHFMLSVKMFY-GNGQ---D-IGFLGKRIKVISKPSKKKQSLKNAE---LCIASGTKVALFNRLRSQTVSTRYLHVENYNGKCNFHASSTQWGAFTIHLLDDNEG--EEEFTVR---DGY--IHYGSTVKLVCSVTGMALPRLVIRKVDKQTALLDADD-----PVSQLHKCAFFKDTER----M-------YLCLSQERI---IQFQATPCPKEPNKEINDGASWTIISTDKAEYTFYEG---------MGPVSLTPVPVVNSL-HLNGGGDVAMLELNGENF---------NPSLKVWFGDVEAETM-FRAE---DSLCVVPDIAAF-------RPG---WKWVRQP---LQAPVSLVRL-DGVIYATGLTFTYTP--EP

>Hro

-----MAEYLRCRHLKILHAKVAQKSYGSEKRFFCPPPCIYLSGQGW------ERPLKPST---CAYMGI---GN-SDQDMVPLNLD-G-KYCAAKTLFISD-SD-KRKHFMLSVRMFY-SNAQ---P-IGFQSKRIKVISKPSKKKQSIKNAD---LCIGSGSKVSLFNRLRSQTVSTRYLHVEK----GNFHASATQWGAFTVHLLDDDES--EEEFSVK---DGF--IHYGSTIKLVCVETGMALPRLVIRKVDKQTVILDTDE-----PVSQLHKCAFYKDTDH----M-------YLCLAHEKI---IQFQATPCPKEPNKEINDGALWTIISTDCAEYAFYEG---------MGPVPITPVPTVQSL-QLNGGGDVAMLELTGENF---------TANLKVWFGEVEAETM-HRCC---EILCVVPDISAF-------RSQ---WKYVREP---LQVPVMLVRN-DGIIYATGLTFTYTP--EP

>Hpu

LTKESMKKYLEDRTVVIQHAKVAQKSYGNEKRFFCPPPCIYLLNPGW------KRKREQMLKDGCAFIGI---GN-SDQDMQQLNLE-G-KYCAAKTLYISD-SD-KRKHFFLSVKMFY-GNGE---D-IGFNSKRIKVISKPSKKKQSLKNAD---LCIQSGSTVALFNRLRSQTVSTRYLHVEN----NNFKASSQQWGAFTIHLLDDDES--EEEFTVR---EGY--IHYGSTIKLVDTITGMALPRLIIRKVDKQMALMDADD-----PVSQLHKCAFYKDTDK----M-------YLCLSQERI---IQFQATPSPKDPKREINDGASWTIISTDRAEYSFYEG---------MGPVPVTPVPLVSDL-HLNGGGDVAMLELRGENF---------TPDLKVWFGCVEAETM-YRNN---DCLCVVPDISAL-------CGE---WRYIQRP---TQVNIMLVRQ-DGIIYPTGRMFTYTP--EP

>Bfl

LTRDAMSRYLKDKTLVIQHAKVAQKSYGSEKRFFCPPPCVYLQGPGW------KNRKEQMEKDGHAFIGI---GN-SDQEMQQLHLE-G-KYCAAKTLYISD-SD-KRKHFMLSVKMFC-GNED---D-IGFFSKRIKVISKPSKKKQSLKNAD---LCIASGTKVALFNRLRSQTVSTRYLHVEG----GNFHASSQQWGAFSIHLLDDAES--EEEFTVR---DGY--IHYGQTVKLVCSKTGMALPRLIIRKVDKQTALLDADD-----PVSQLHKCAFYKDTER----M-------YLCLSQERI---IQFQATPCPKEPNKEINDGASWTIISTDKAEYTFYEG---------MGPVPLTPVPVVDSL-QLNGGGDVAMLEVQGENF---------TPALKVWFGNVEAETM-YRCG---ENLCVVPDISAF-------REG---WRWVRQP---IQVPISLVRN-DGVIYPTSLNFTYTP--EP

>Xtr

LTREAMRNYLKERTVLILHAKVAQKSYGNEKRFFCPPPCVYLMGSGW------KKKKEQMERDGCAFIGI---GN-SEQEMQQLNLE-G-KYCTAKTLYISD-SD-KRKHFMLSVKMFY-GNSD---D-IGFLSKRIKVISKPSKKKQSLKNAD---LCIASGTKVALFNRLRSQTVSTRYLHVEG----GNFHASSQQWGAFYIHLLDDEES--EEEFTVR---DGY--IHYGQTVKLVCSVTGMALPRLIIRKVDKQTALLDADD-----PVSQLHKCAFYKDTER----M-------YLCLSQERI---IQFQATPCPKEPNKEINDGASWTIISTDKAEYTFYEG---------MGPIPVTPVPVVESL-QLNGGGDVAMLELTGQNF---------TPNLRVWFGDVEAETM-YRCA---ESLCVVPDISAF-------REG---WRWVRQP---VQVPVTLVRN-DGVIYSTSLTFTYTP--EP

>Uma1

RVEKALSEYIASITIRCSHASVAQKSYGSEKRFFNPPPIISITGPLR-----------RYAEHGQCRVSLVVRGDTDEYFSNENVAVLDGRETKMRSLYVTP-TG-RSKSFRLQLNLLRPSGLEPHLPIFSFESAPVTIISKSSKKTAKPRGEL---NQVKNGSMISLYNRINSQTARTKYMRSNG----GDLTVHNGDWSTYRITLISRPPL---AELAGAEADS----VTYGSTIVLTDVNTGFSSDPLVVCKVDKGQVQLGADDLNLFGPVGQMQKVALMGDSEHLESNFSTPRS--YLCISPEKV----GQQSLPAHTSDKHHVDDAFVWTVIGVSRFEYSYFDT-SATQASKDEGPCPMTPFPLITSM-PI-YDQTRHTLVTTVTNFIVPAAITSDPIPLDVWAGSLGPLKVSHSTNAPSTSTVTLPAIREMIKVALTARRSSPTSAPIPPH---VLLPLIFVNDSDGTAYHSGRHIVCEDLVQL

>Uma2

FIQDYVRQYISTSNVLIMTTRVAQKSYGAEKRFLCPPPLVILIGSSWWNVCPASTRPSPFAPAGRVTINI--SGEPGVQDGALEWASSSGRRCIGKQLYISD-VDEKRRHVEALVAISVPGSSPMDRRLLGFPSRPIKVISKPSKKRQSSRNTE---LCVNHGSVISLFHRLRSQTVSTRYLCVKGSEPPSCFVAKMSSWDPFIIYLVDPKKRADANALPTTGLTSSQMTIHYNQPIVLQCLNTAVVSPVMVVRKVEKGTTVLGGGD-----PVSQLHKIALEPTGDSGESNWPGNSGPFLACMSPTSA---AAYAASPSASTGSHDVADSDIWTVVGTDIARHSFYVPPQIVGGSQPQTNIPITPIPIISNMVAPQGEQSPGLVTILGENF---------HPNLFIFFGDWKSTHVDVRTR---QTVCAAPP--SF-------DQG------LPRG----RVPILIVRH-DGVIFPTDFHYQC------

>Hma

-------------HIRC-----------------------------------------------CVMDNY---------------------------------------------------------------------------------------QC-------------------RKYLQV---------------------------------------------------------------------------------------------------REL--------------------------VQFKA----------------------KLLQFHLFLI-------------------------CIPEGGDVVTMEVNGEKF---------TSNLKVWFADVEAETM-YRSS---ETICLVPDVSLF-------KDR---WKYKQSD---FSVPVSLVRD-DGVIYPTDCTFTFTP--EL

>Hsa1

ILRGGVRRCLQQQTVRILHAKVAQKSYGNEKRFFCPPPCVYLSGPGW----RVKPGQDQAHQAGCGYMGLDSASG-SATETQKLNFEQQPDFGCAKTLYISD-AD-KRKHFRLVLRLVLRGGRE-----LGFHSRLIKVISKPSQKKQSLKNTD---LCISSGSKVSLFNRLRSQTVSTRYLSVED----GAFVASARQWAAFTLHLADGHSAQ--GDFPPR---EGY--VRYGSLVQLVCTVTGITLPPMIIRKVAKQCALLDVDE-----PISQLHKCAFQPGSPPGGGGT-------YLCLATEKV---VQFQASPCPKEANRALNDSSCWTIIGTESVEFSFSTS---------LACTPVTPVPLISTL-ELSGGGDVATLELHGENF---------HAGLKVWFGDVEAETM-YSPR----SVCVVPDVAAF-------CSD---WRWLRAP---ITIPMSLVRA-DGLFYPSAFSFTYTP--EY

>Gga1

LLRDGVRRYLQLPTVLILHAKVAQKSYGNEKSF-PSPP---LPSPSS------PFVPARD----WGYMGLDTMGS-SLMETQKLSFEEQPDFGCAKALYISD-AD-KRKHFRLVLKLFF-SNGQ---E-IGFHSKLIKVISKPSQKKQSLKNTD---LCISSGSKVSLFNRLRSQTVSTRYLSVEG----GAFIASARQWAAFTLHLADERCT---SEFPLR---EGY--IRYGSVVQLICTATGITLPPLIIRKVSKQYAMLDVDE-----PISQLHKCAFQQGSDH----M-------YLCLSTEKV---IQFQASPCPKEANRELNDGSCWTIIGTETVEYSFSES---------LACAPVSPVPLITAL-QLSGGGDVAMLEVQGEHF---------HAHLKVWFGDVEAETM-YRSP---KSMCVVPDVSAF-------SSD---WRWLRYP---ITVPLLLVRD-DGLIYSSSFTFTYTP--EQ

>Gga2

LTREAMRNYLKERTVLILHAKVAQKSYGNEKRFFCPPPCVYLMGSGW------KKKKEQMERDGCAFIGI---GN-SDQEMQQLNLE-G-KYCTAKTLYISD-SD-KRKHFMLSVKMFY-GNSD---D-IGFLSKRIKVISKPSKKKQSLKNAD---LCIASGTKVALFNRLRSQTVSTRYLHVEG----GNFHASSQQWGAFYIHLLDDDES--EEEFTVR---DGY--IHYGQTVKLVCSVTGMALPRLIIRKVDKQTALLDADD-----PVSQLHKCAFYKDTER----M-------YLCLSQERI---IQFQATPCPKEPNKEINDGASWTIISTDKAEYTFYEG---------MGPVPVTPVPVVESL-QLNGGGDVAMLELTGQNF---------TPNLRVWFGDVEAETM-YRCA---ESLCVVPDISAF-------REG---WRWVRQP---VQVPVTLVRN-DGIIYSTSLTFTYTP--EP

>Cin1

LLGDIVRRYLRKRKVVILHAKVAQKSYGSEKRFFCPPPCVHLLGTGW------KTKQRRKMTSSCLFVGV---GG-GENELQQVFLDPPAKYAAAKTLHTPD-SD-KRKHFEIHVKMFY-GDGS---E-IGFHSQRIKVISKPSKKKQTVKNTD---LCIPSGSKVSLFNRLRSQTVSTRYLHVTK----DGYRASATQWGAFAIHLIHENQP--ENEFEVH---DGY--VHYGTSVKLVCSVTGYALPRLIIRKVDRQMASVLSDE-----PVSQLHKCALYKDTDR----Q-------YLCLSQEKI---IVHKATQSTKNPSMDLSDGACWTIISTEKAEYRWSDS---------SFNLPVTPVPVVKNL-EINGGGEVAMAEFTGTNF---------APNLTVWFSDVETETM-FRCS---ESMCFIPDISKI-------QKG---WKYVKKT---VKVPLLLVRD-DGVIYPCGVNFYYTP--EP

>Cin2

LTRDAMRRYLKDPTLIVLHAKVAQKSYGNEKRFFCPPPCMYLLGNGW------KRKQQILEEEEHAFIGI---GS-SEQEMQQLHLD-G-KFCTAKTLYISD-TD-KRKHFMLNVKMFFGGGGA---D-VGFSSKRIKVISKPSKKKQSLKNAD---LCIASGTKVALFNRLRSQTVSTRYLHVEK----GNFHASSIQWGCFAIHLLDDDES--EEEFSVV---DGY--IHYGQTVKLVCSNTGMALPRLIIRKVDKQTAILDADD-----PVSQLHKCAFYKDTER----M-------YLCLSQERI---IQFQATPCPKETNKEINDGASWTIISTDKAEYTFCDG---------MGPTPVTPVPNVHSL-QLNGGGDVAMLEVNGECF---------TSNLKVWFGEIEADTM-FRCA---EGLCVVPDISAF-------REG---WKWVKES---VQVPINLVRN-DGVIYPTNLTFTFTP--EP

>Hsa2

LTREAMRNYLKERTVLILHAKVAQKSYGNEKRFFCPPPCVYLMGSGW------KKKKEQMERDGCAFIGI---GN-SDQEMQQLNLE-G-KYCTAKTLYISD-SD-KRKHFMLSVKMFY-GNSD---D-IGFLSKRIKVISKPSKKKQSLKNAD---LCIASGTKVALFNRLRSQTVSTRYLHVEG----GNFHASSQQWGAFFIHLLDDDES--EEEFTVR---DGY--IHYGQTVKLVCSVTGMALPRLIIRKVDKQTALLDADD-----PVSQLHKCAFYKDTER----M-------YLCLSQERI---IQFQATPCPKEPNKEINDGASWTIISTDKAEYTFYEG---------MGPVPVTPVPVVESL-QLNGGGDVAMLELTGQNF---------TPNLRVWFGDVEAETM-YRCG---ESLCVVPDISAF-------REG---WRWVRQP---VQVPVTLVRN-DGIIYSTSLTFTYTP--EP

>Dre1

FSREAMRNYLKERTVLILHAKVAQKSYGNEKRFFCPPPCVYLMGCGW------KKKREQMERDGCAFIGI---GN-SEQEMQQLNLE-G-KYCTAKTLYISD-SD-KRKHFMLSVKMFY-GNSA---D-IGFLSKRIKVISKPSKKKQSLKNAD---LCIASGTKVALFNRLRSQTVSTRYLHVEG----GNFHASSQQWGAFFIHLLDDEES--EEEFTVR---DGY--IHYGQTVKLVCSVTGMALPRLIIRKVDKQTALLDADD-----PVSQLHKCAFYKDTER----M-------YLCLSQERI---IQFQATPCPKEPNKEINDGASWTIISTDKAEYTFYEG---------MGPVPVTPVPVVESLQQLNGGGDVAMLELTGQNF---------SPTLRVWFGDVEAETM-YRCG---ESLCVVPDISAF-------REG---WRWVRQP---VQVPVTLVRN-DGIIYSTTLTFTYTP--EP

>Dre2

LTREAMRNYLKERTVLILHAKVAQKSYGNEKRFFCPPPCVYLMGCGW------KKKKEQMEREGCAFIGI---GN-SEQEMQQLNLE-G-KFCTAKTLYISD-SD-KRKHFMLSVKMFY-GNSA---D-IGFLSKRIKVISKPSKKKQSLKNAD---LCIASGTKVALFNRLRSQTVSTRYLHVEG----GNFHASSQQWGAFYIHLLDDDES--EEEFTVR---DGY--IHYGQTVKLVCSVTGMALPRLIIRKVDKQTALMDADD-----PVSQLHKCAFYKDTER----M-------YLCLSQERI---IQFQATPCPKETNKEINDGASWTIISTDKAEYTFYEG---------MGPVPVTPVPVVESL-QLNGGGDVAMLELTGQNF---------TPNLRVWFGDVEADTM-YRCG---ESLCVVPDISAF-------REG---WRWVRQP---VQVPVTLVRN-DGIIYSTALTFTYTP--EP

>Cel

LTSDRMIDFLSNKVISIFHAKVAQKSYGNEKRFFCPPPCIYLIGQGW------KLKKDRVAQLYVAYIGI---GS-DTSERQQLDFSTG-KYCAAKTLYISD-SD-KRKYFDLNAQFFY-GCGM---E-IGFVSQRIKVISKPSKKKQSMKNTDCKYLCIASGTKVALFNRLRSQTVSTRYLHVEG----NAFHASSTKWGAFTIHLFDDERGLQEDNFAVR---DGF--VYYGSVVKLVDSVTGIALPRLRIRKVDKQQVILDAEE-----PVSQLHKCAFQIDNEL----V-------YLCLSHDKI---IQHQATAI--NEHRHINDGAAWTIISTDKAEYRFFEA---------MGQVPISPCPVVGSL-EVDGHGEASRVELHGRDF---------KPNLKVWFGATPVETT-FRSE---ESHCSIPPVSQV-------RNEQTHWMFTNRTTGDVEVPISLVRD-DGVVYSSGLTFSYKS--LE

>Spu1

-------------------------------------------------------------------------------------------------------------------------------------------------------------------------------------------------------------------------------------------------------------------------ND-----------------------------------C----------------------------------------------------------------------------------------------------------------------------LCVVPDISAL-------CGE---WRYIQRP---TQVNIMLVRQ-DGIIYPTGRMFTYTP--EP

>Spu2

----------------------------------------------------------------------------------------------------------------------------------------------------------------------------------------------------------------------------------------------------------------------------------------------------------------------------ATPSPKDPKREINDGASWTIISTDRAEYSFYEG---------MGPVPVTPVPLVSDL-H-------------------------------------------------------------------------------------------------------------------

>Aae

LTREAMEKYLRDRVIVILHAKVAQKSYGNEKRFFCPPPCIYLFGEGW------RLRKEQMLRRGCAFIGI---GS-SDQDMQPLDLN-NSKYCAAKTLFISD-SD-KRKHFMLSVKMFY-GSGH---D-IGFQSKRIKVISKPSKKKQSLKNAD---LCIASGTKVALFNRLRSQTVSTRYLHVED----GHFHASSTQWGAFTIHLLDDNES--EEEFQVR---DGY--VHYGATVKLVCSVTGMALPRLVIRKVDKQMALLEADD-----PVSQLHKCAFHKDTER----M-------YLCLSQEKI---IQFQATPCPKEPNKEINDGACWTIISTDKAEYQFYEG---------MGPVPVTPVPIVNSL-HLNGGADVAMLEITGDNF---------TPSLQAWFGDVEAETM-YRCA---ESLCVVPDISQF-------RGE---WSWVRQP---TQVPISLVRN-DGIIYATGLTFTYTP--EP

>Nve

LTDVVHRNGL---TVVIFHAKVAQKSYGNEKRFFCPPPCVYLFGEGW------RNRQRLLQQAGVAFIGI---GNSSEQEMQQLIIE-E-KYGAAKTLFISD-SD-KRKHFELSVKLLY-PNGD---H-VGFNSKRIKVISKPSKKKQSLKNAD---LCIQSGSTVALFNRLRSQTVSTRYLHVEN----GNFHASSQQWGAFTIHLLDDEES--EEEFTVR---EGY--IHYGSTVKLVCSVTGMALPRLIVRKVDKQTSLLDADD-----PVSQLHKVAFYKDTER----M-------YLCLSQERI---IQFQATPCPKESNKEINDGASWTIISTDKAECNFYEG---------MGPVPVTPVPFVSHL-QLNGGGDVAMLELTGENF---------TANLKVWFGDVEAETM-YRCG---ESLCVVPDISAI-------RGG---WRFVRQP---TEVAVSLVRC-DGVIYPTGLTFTYTP--EP

>Spo

LNEDSVHKISLTRTVSCRHSSVIQKSYGSEKRYLCPPPMVYINGNYS----------SIFNQSF---ISI---MNDFGQCSQPISEEYTGQCMIFRSLHISSLVAAKSKNLRLSLDMFSNVNN----QLLSLVTSSISIVSKPSKKGSKLKISN---ITLRSGSVVSLYNRINSQTVRTKYTSIEA----GQFCLRGDRWVPLRINLLLPDEN---GKLKVCDDVDNPEPIKYGSIVELVDEATGTTSDPLIIRRVEKDHIAE--ED----GYVNQMHRIVLEKIAEHSSLAYSNNISVRWFLASSEAI-LPIEWEAVGNLSSNEMTVGDSVCWTIVGISHFDCTMMLP-------FNQNPVTVTDYPYIEE--PPEYLESSRSLQFKIGGY---------SVGLQIWLGVHGPLSYSFTAAADTSTGTVTLGLSQI------------SYDPSCAE---QKYPLLFVIP-GGIVIIGKCEILLTS----
