# Supplementary figures and images for: Origin and evolution of the Notch signalling pathway: an overview from eukaryotic genomes
Source: BMC Evol Biol. 2009 Oct 13;9:249. doi: 10.1186/1471-2148-9-249 (PMC2770060; doi:10.1186/1471-2148-9-249)

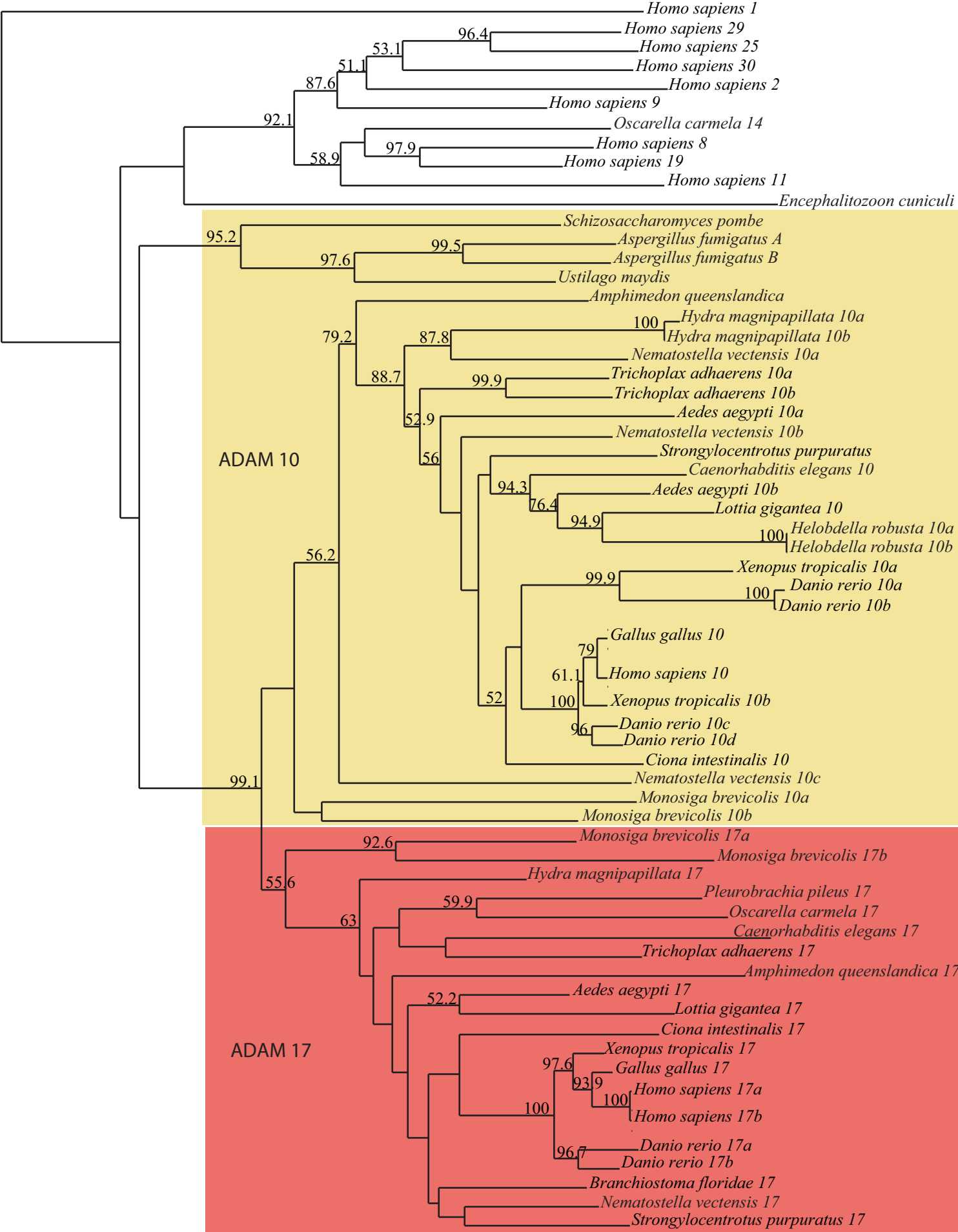

Aph1

0.2

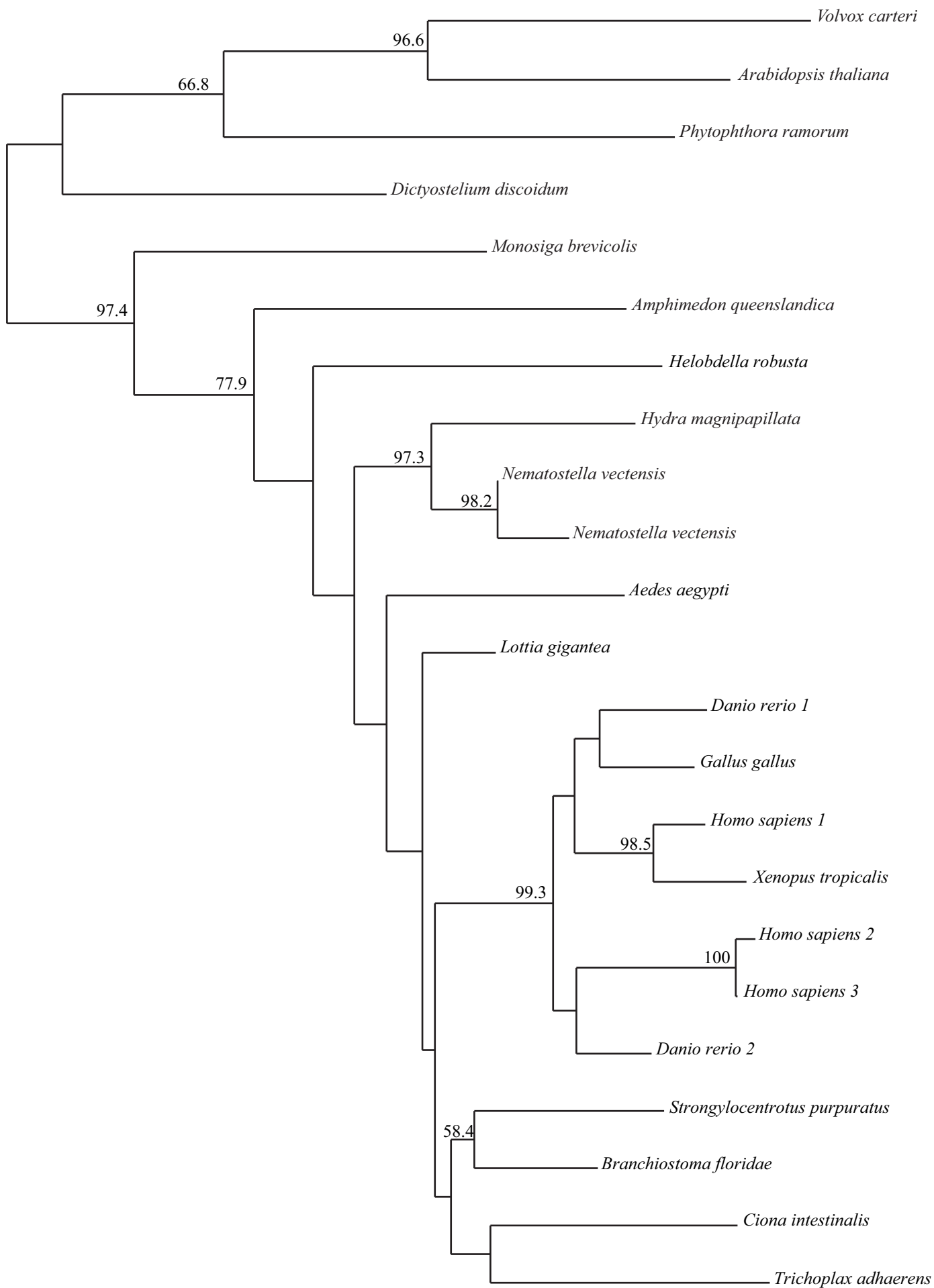

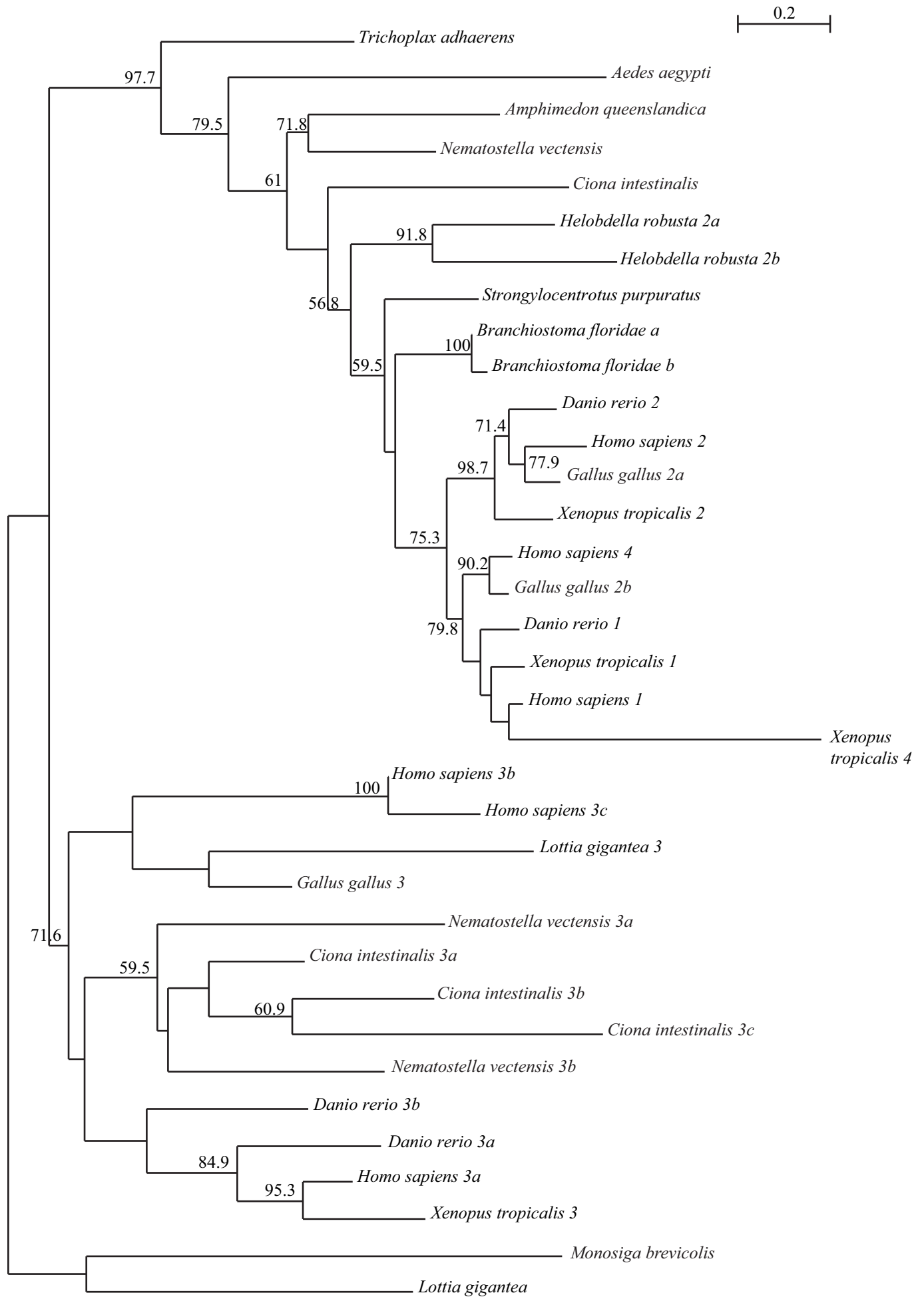

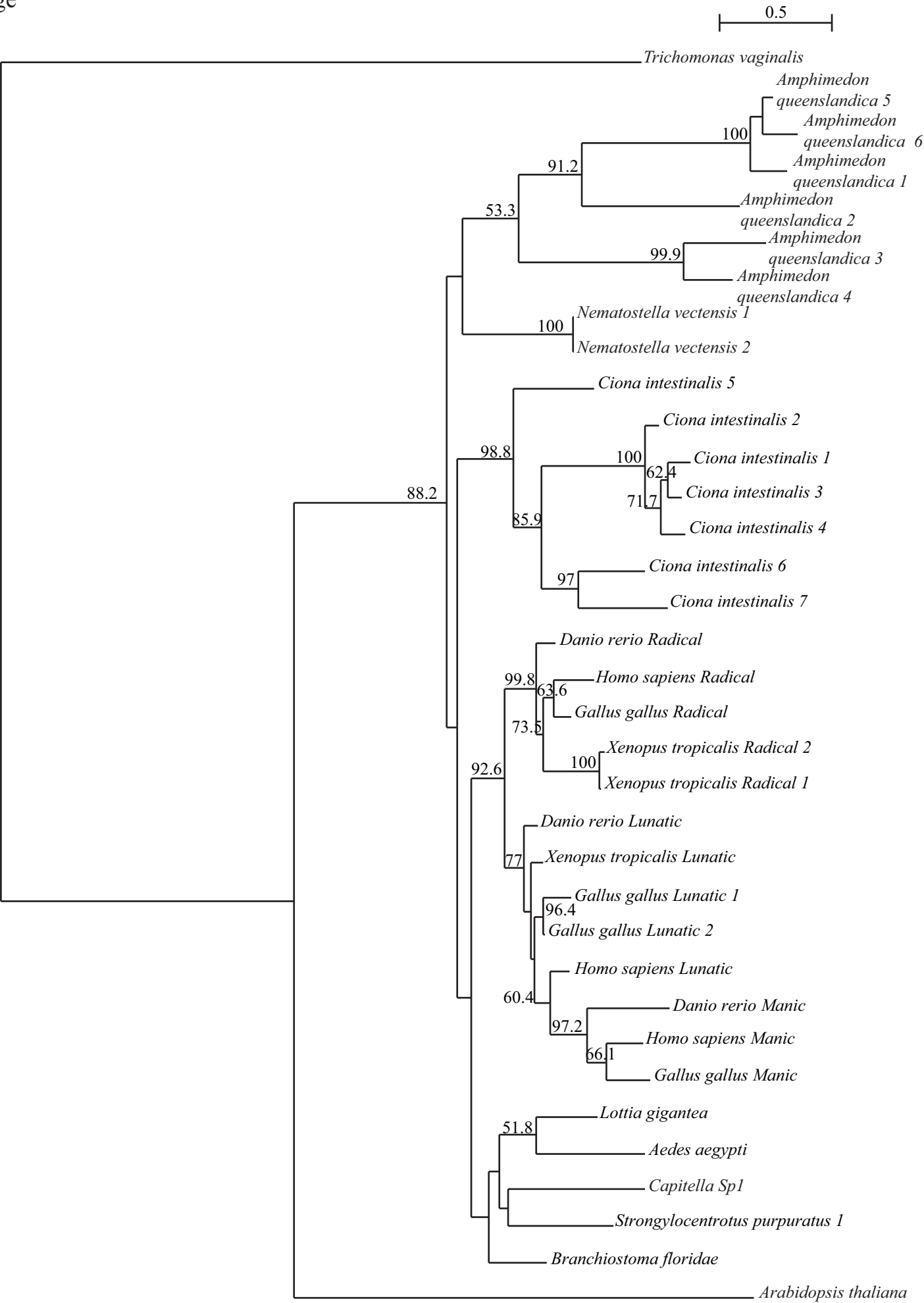

# Furin

0.2

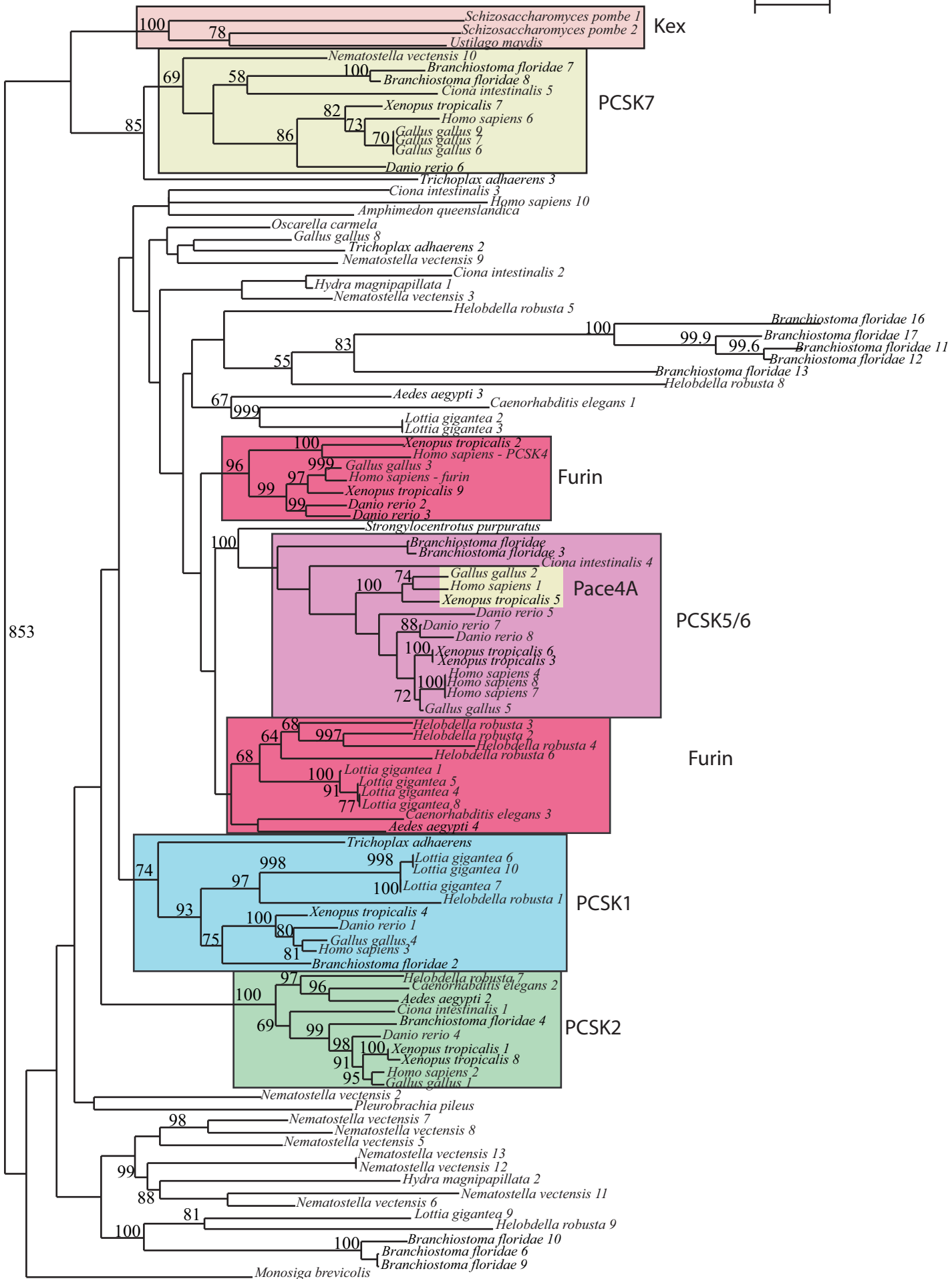

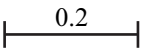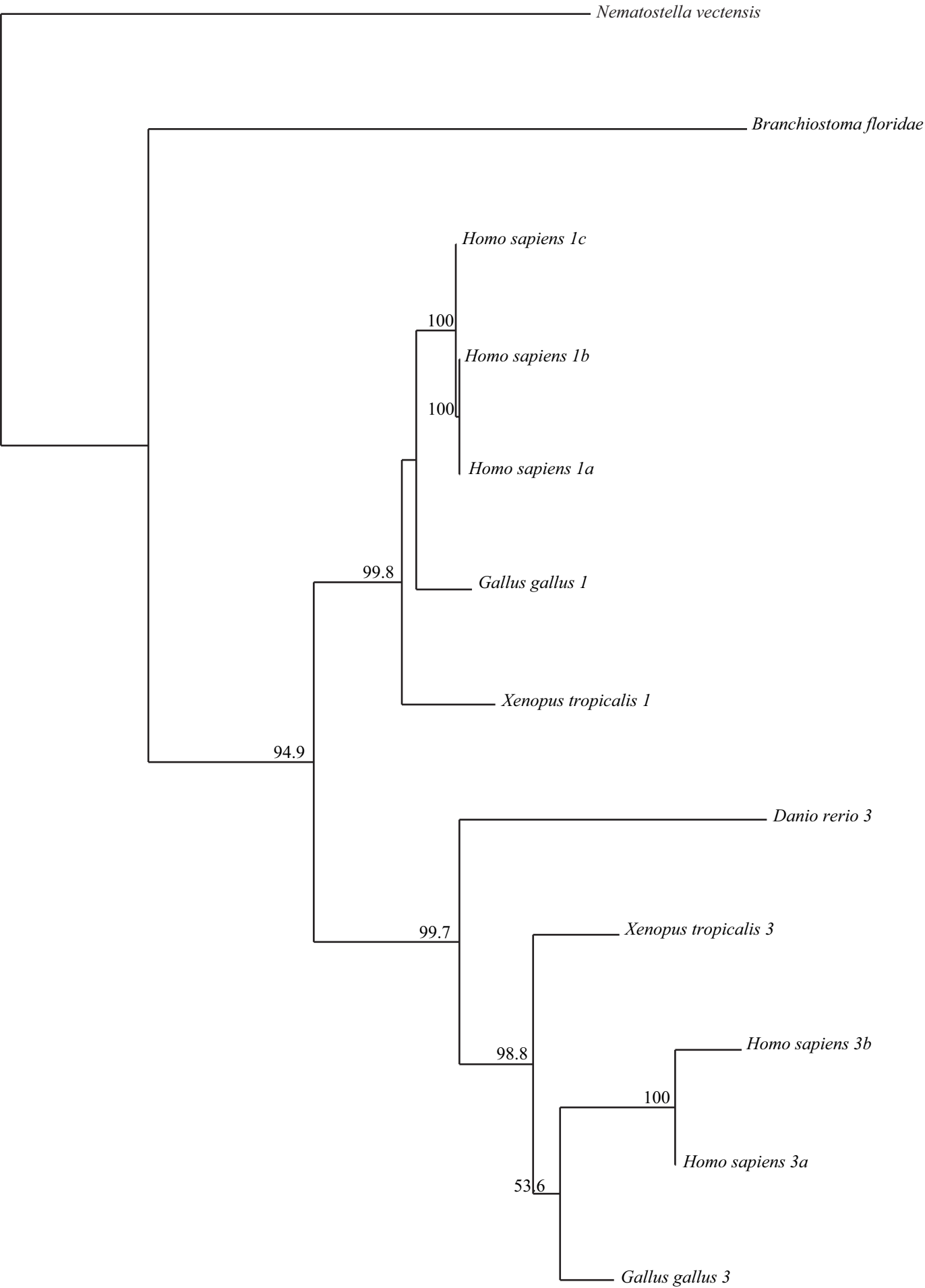

0.1

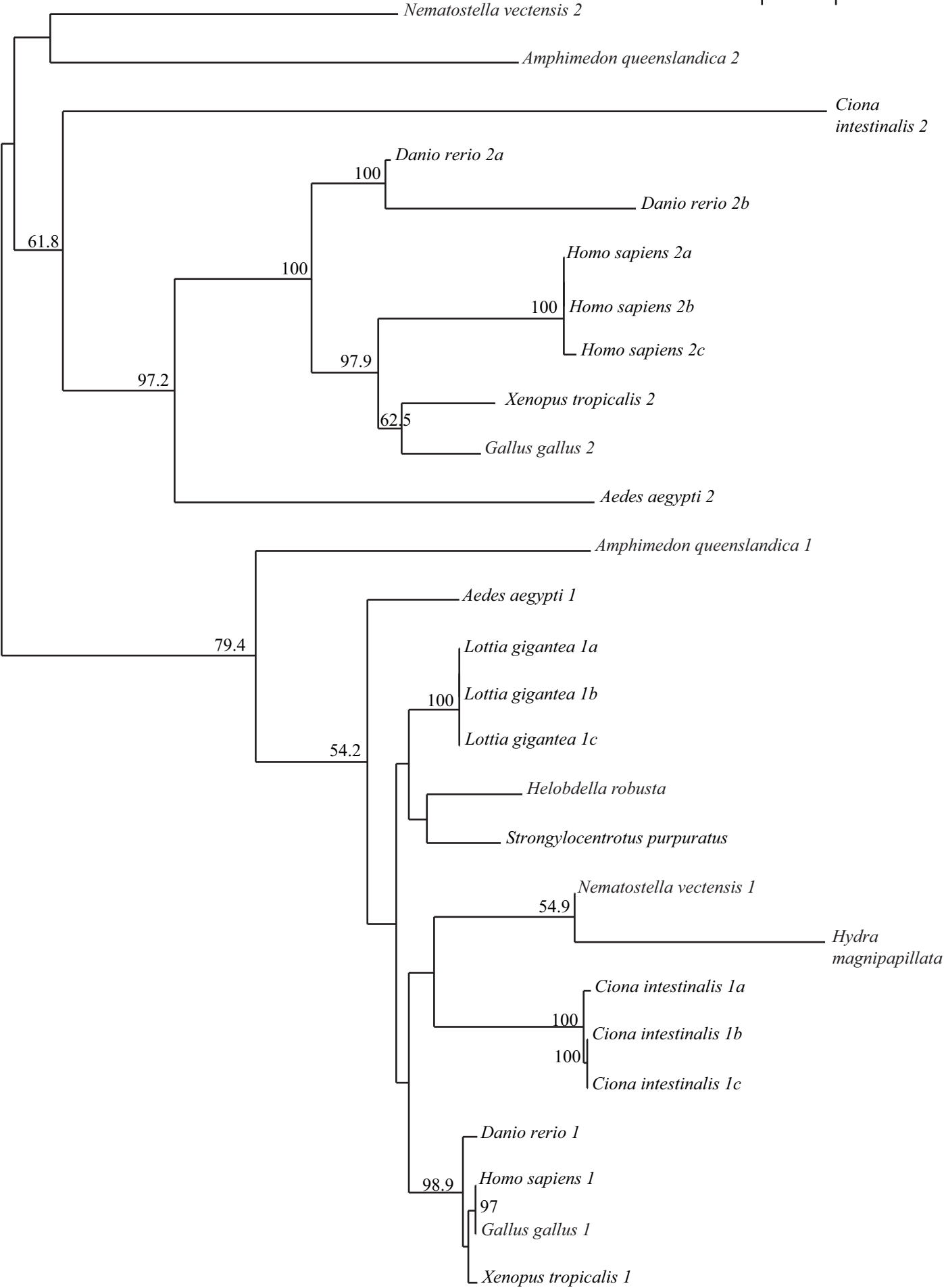

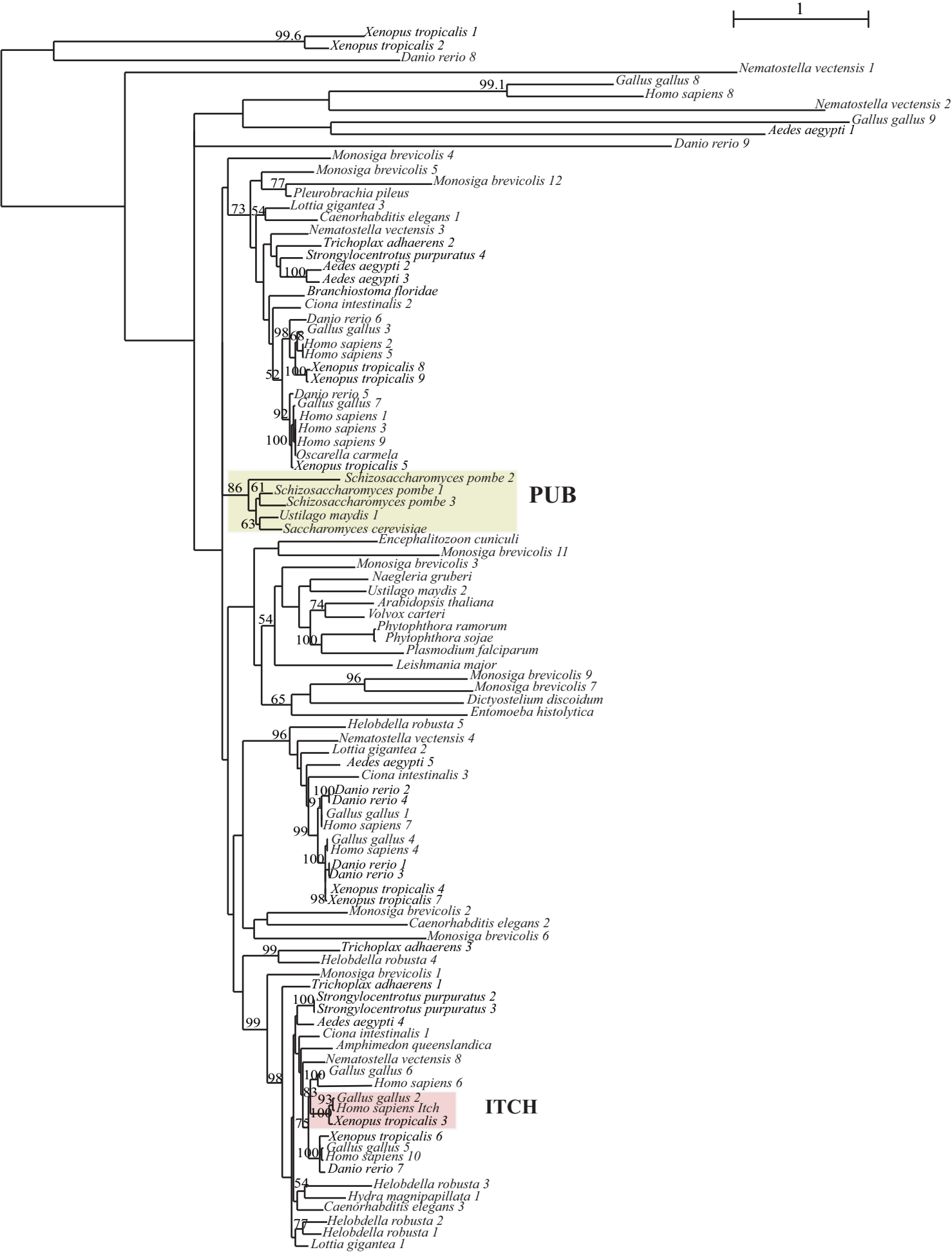

Neuralized

0.2

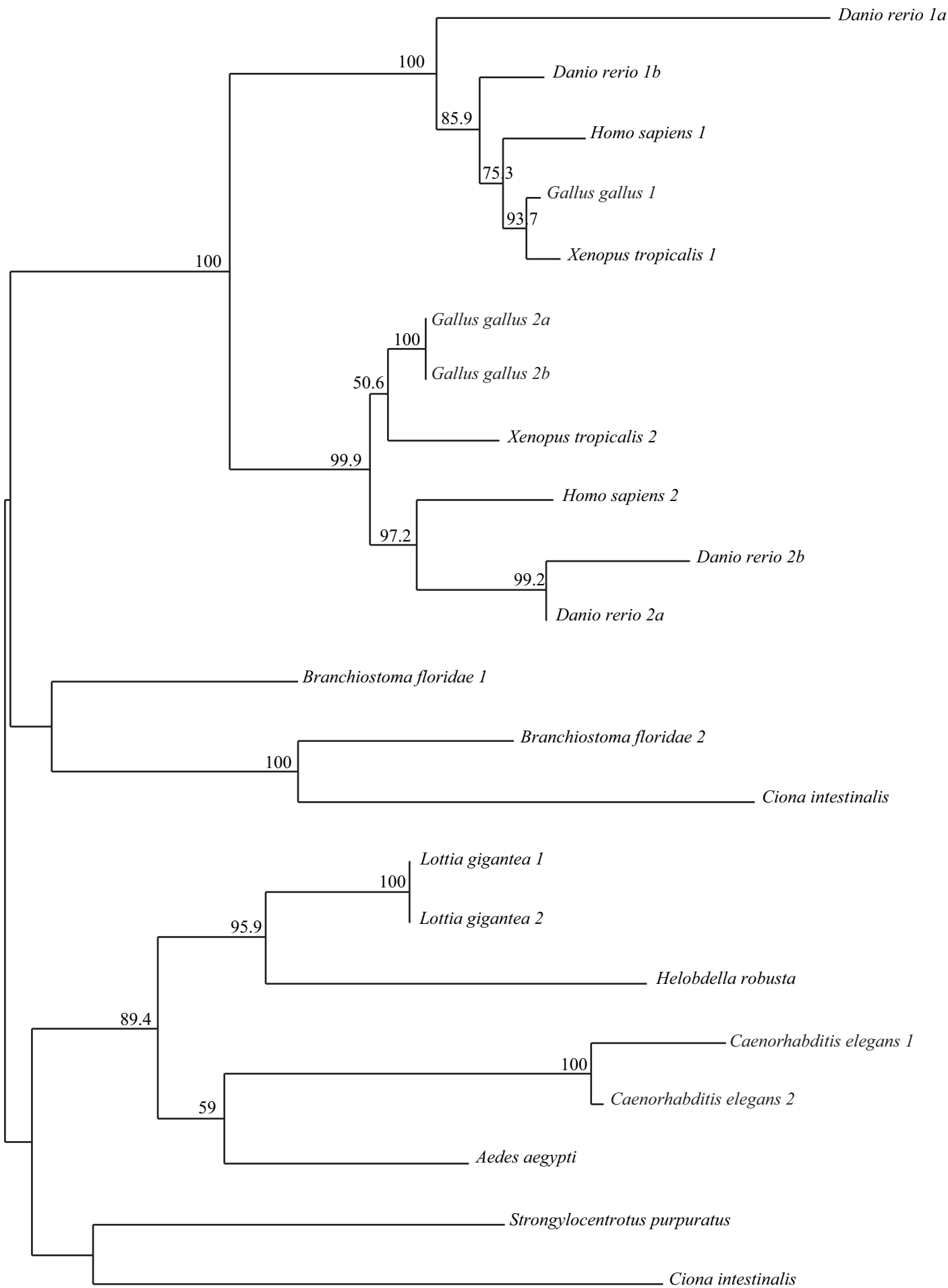

Nicastrin

0.2

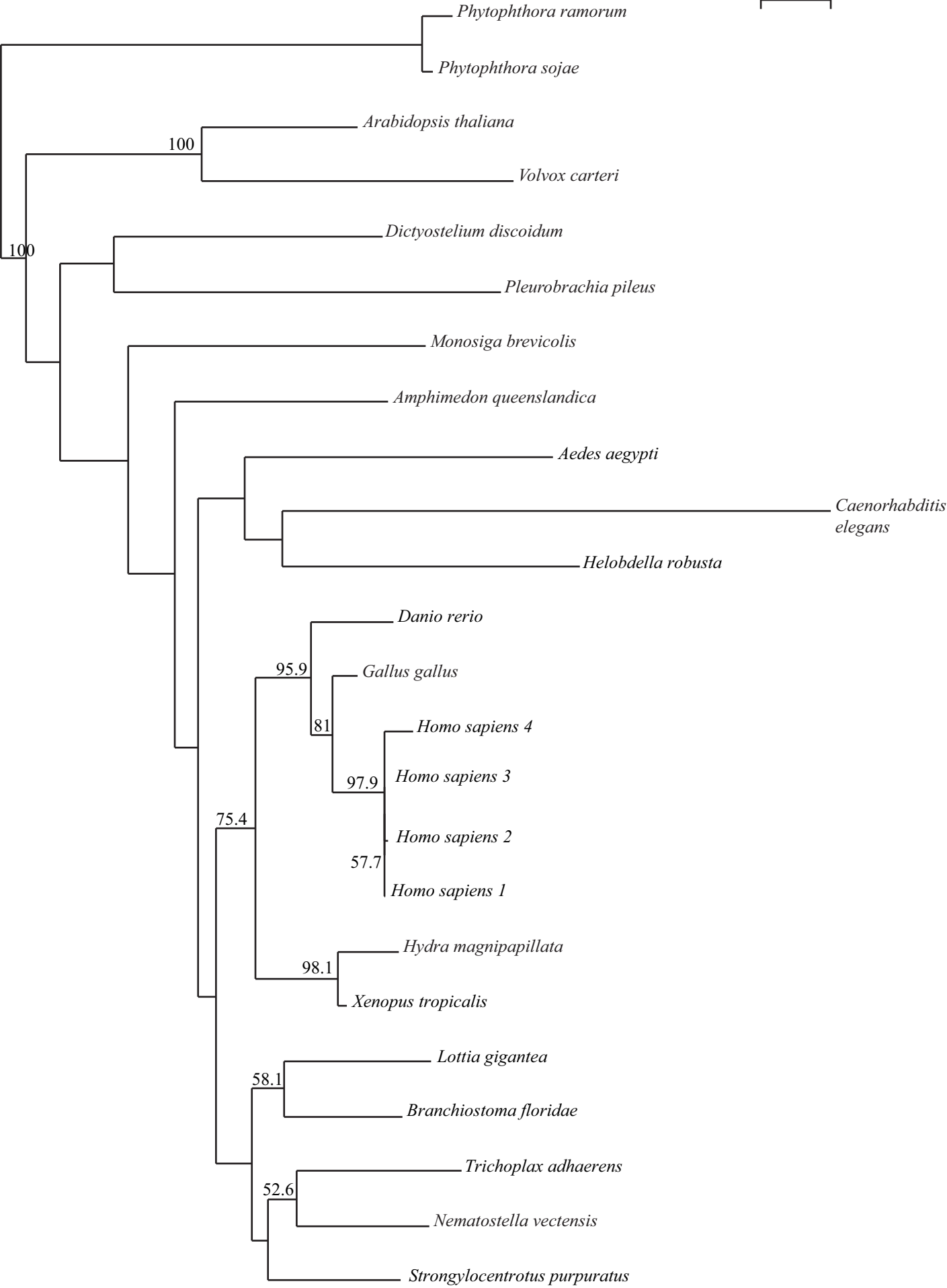

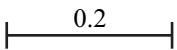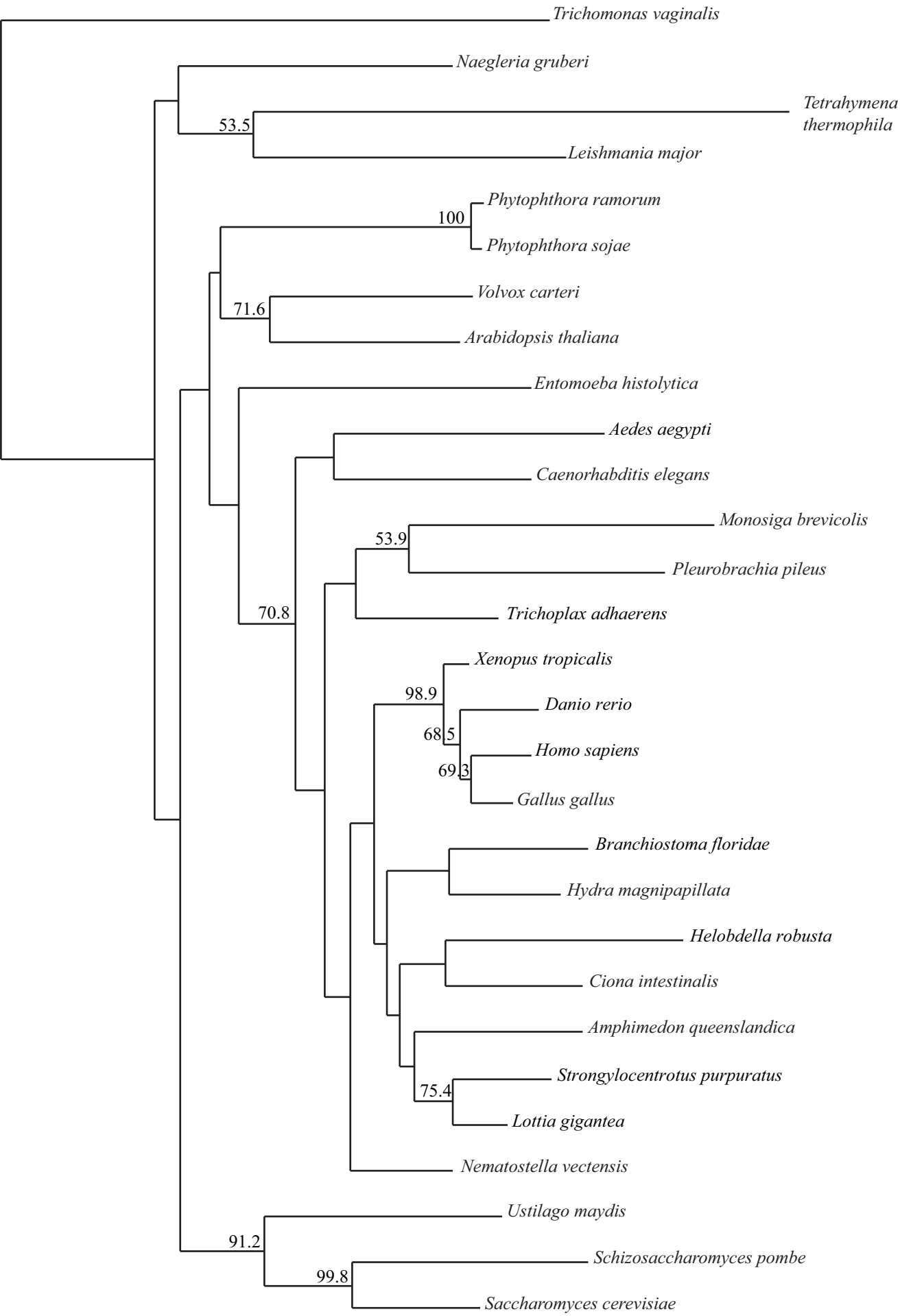

NUMB

0.2

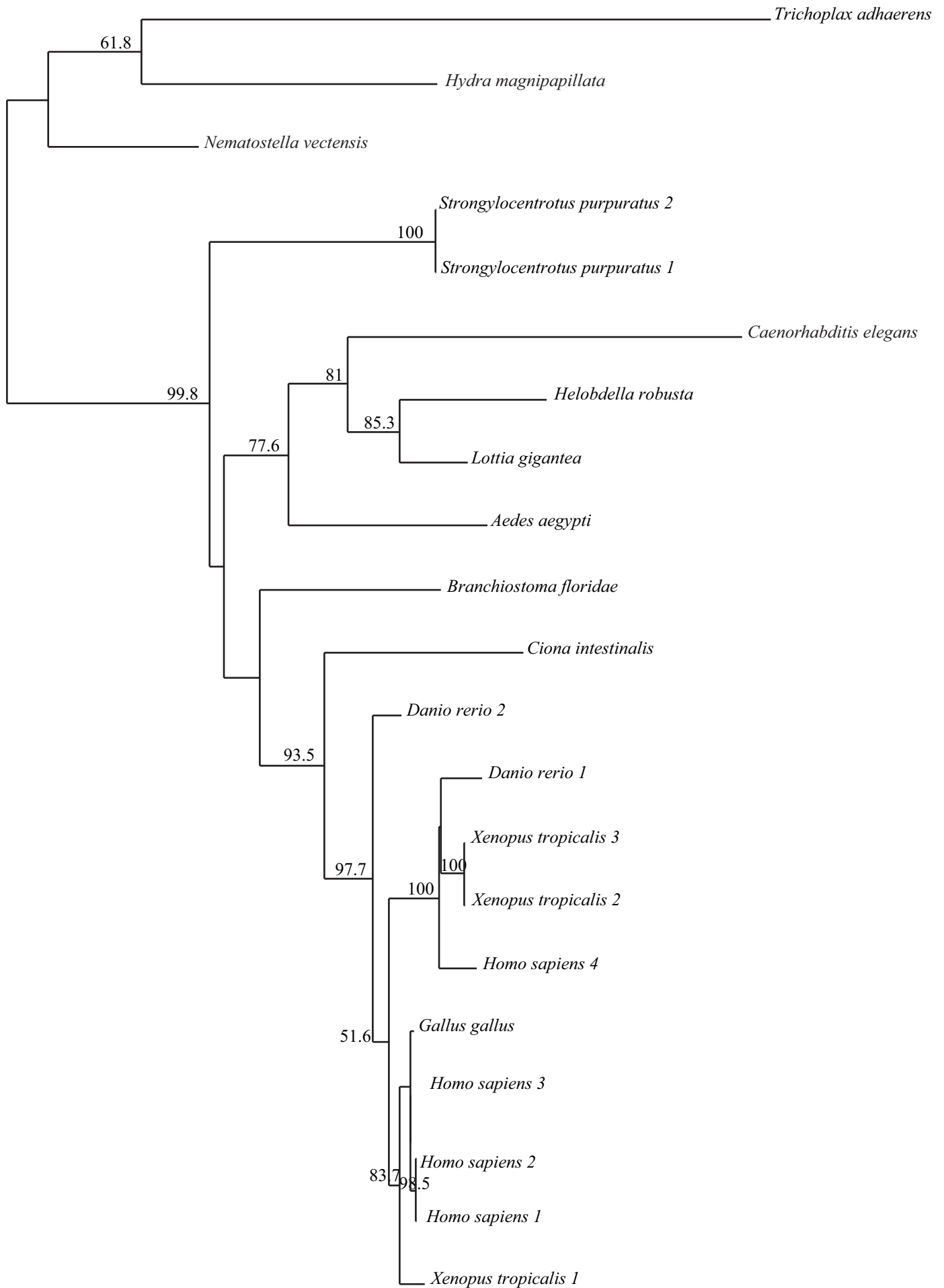

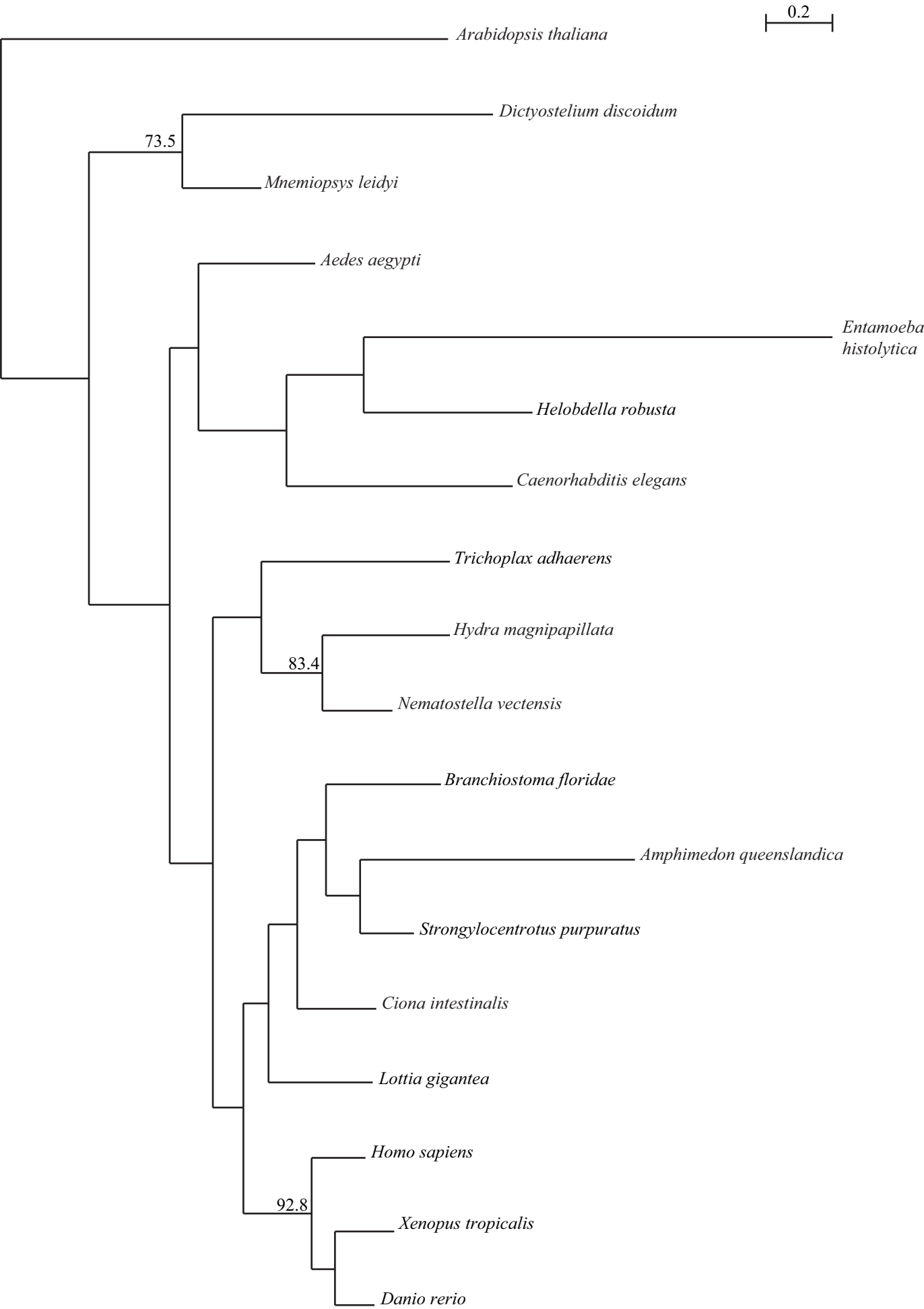

Presenilin

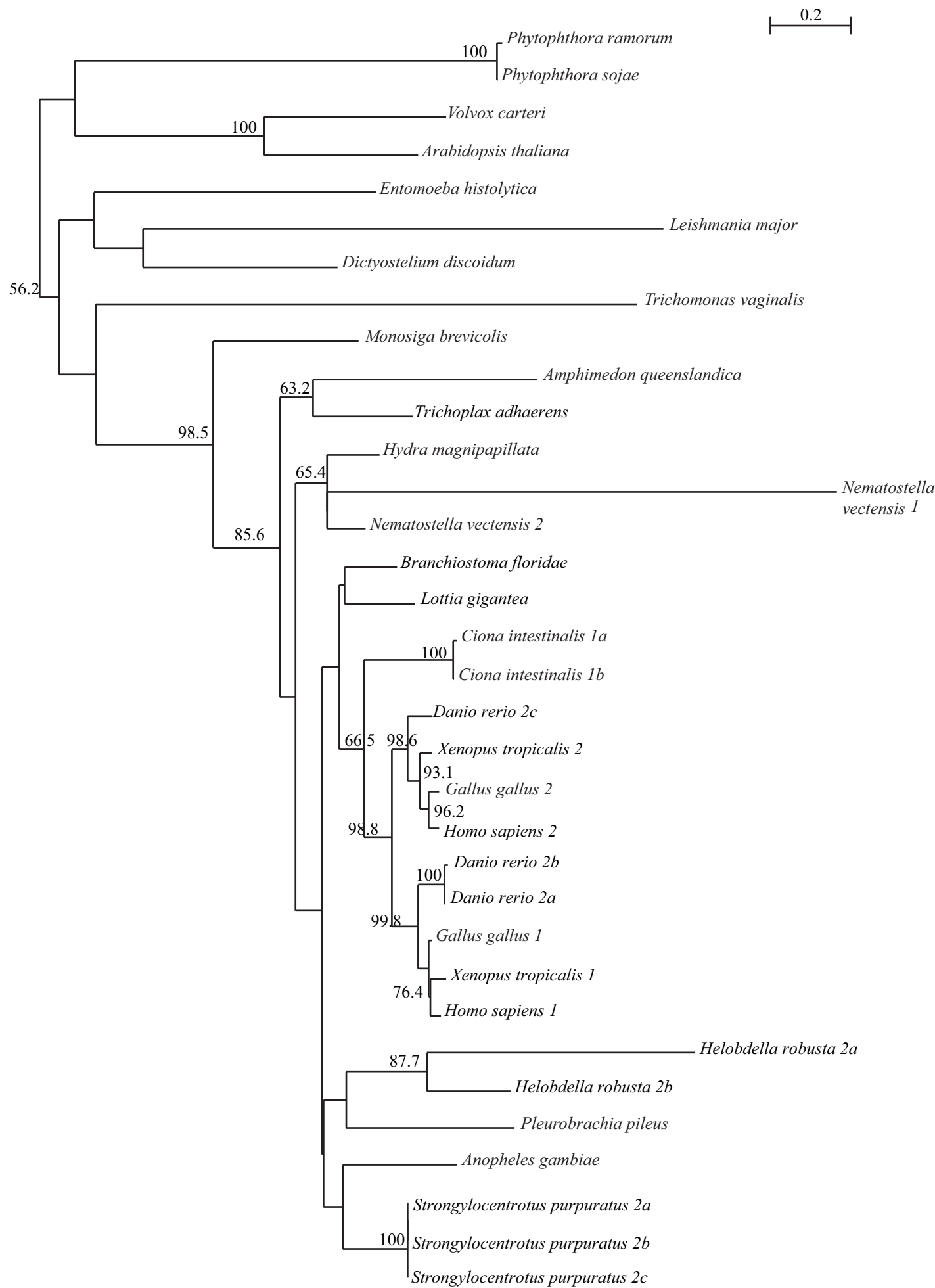

Strawberry Notch

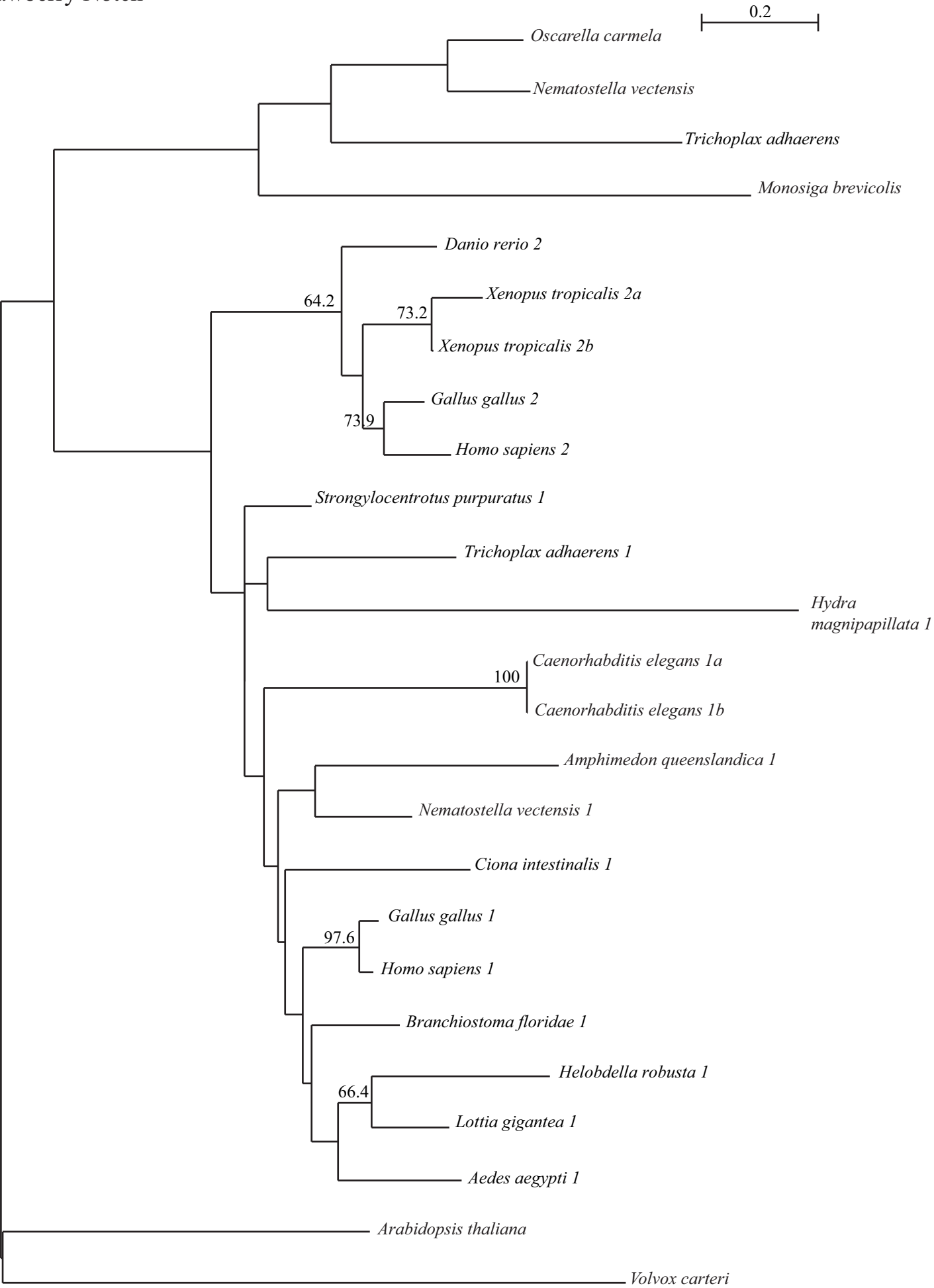

Su(H)

0.2

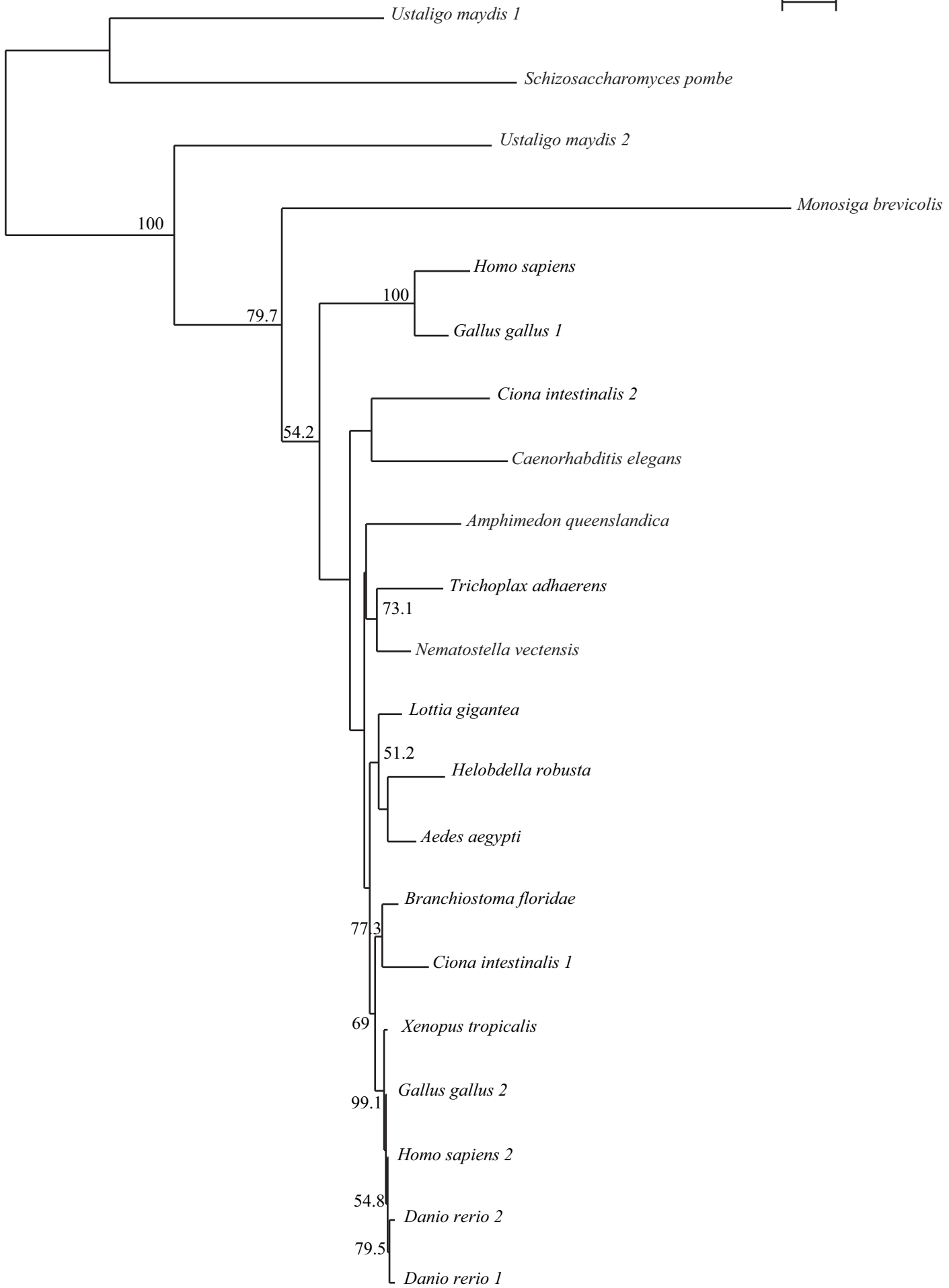

Supplement: Additional file 2 — Phylogenetic analyses. In this file we provide the phylogenetic trees constructed from the protein alignments using the maximum likelihood method (ML) with the PHYML program for 16 Notch components (excepted Notch and Delta/Jagged). [file 1471-2148-9-249-S2.PDF]

Notch Phylogenetic tree obtained by ML method

0.2

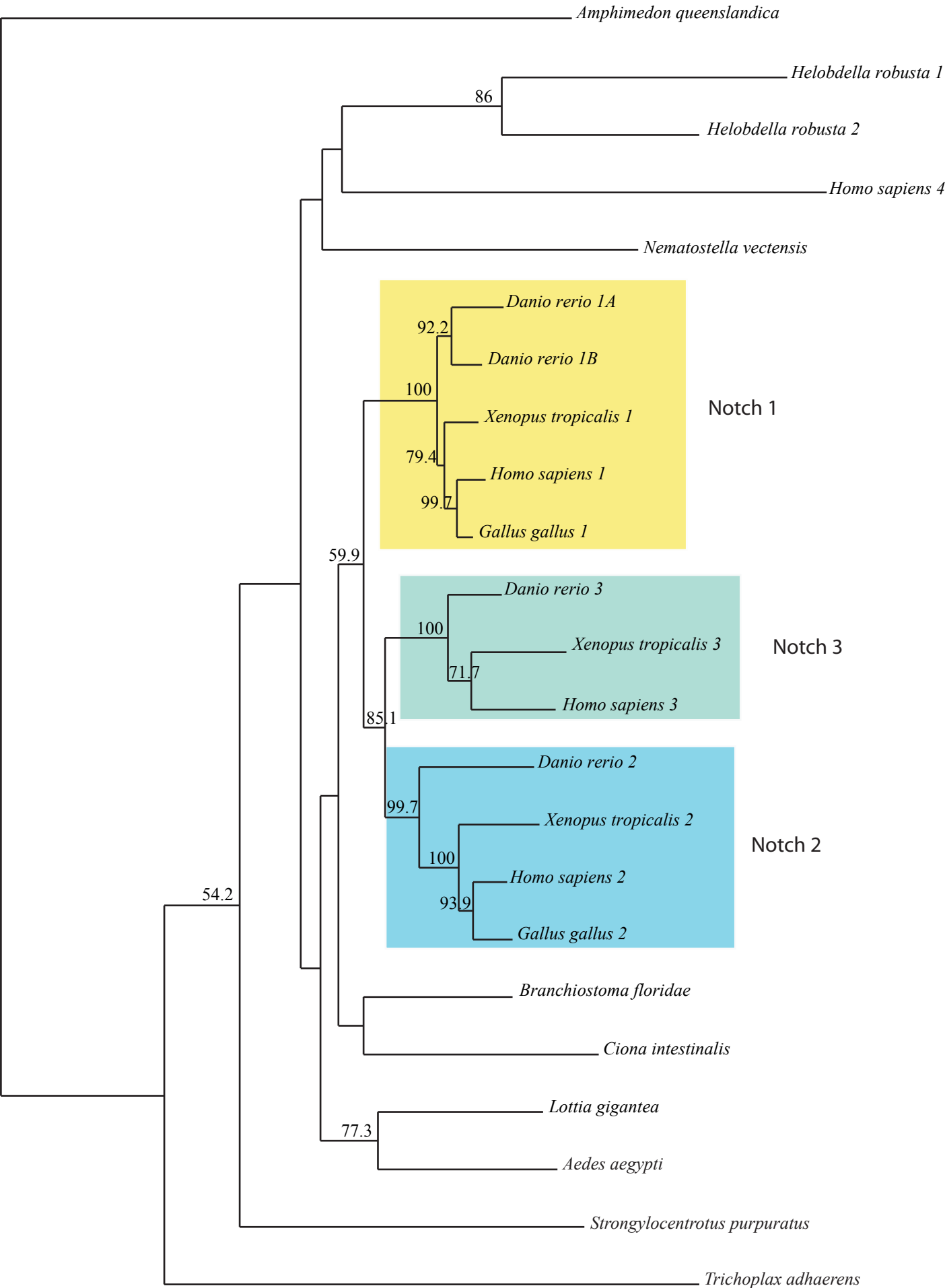

Supplement: Additional file 5 — Notch phylogenetic tree. Notch phylogenetic tree constructed from the protein alignments using the maximum likelihood method (ML) with the PHYML program. [file 1471-2148-9-249-S5.PDF]

DLS proteins phylogenetic tree obtained by ML method

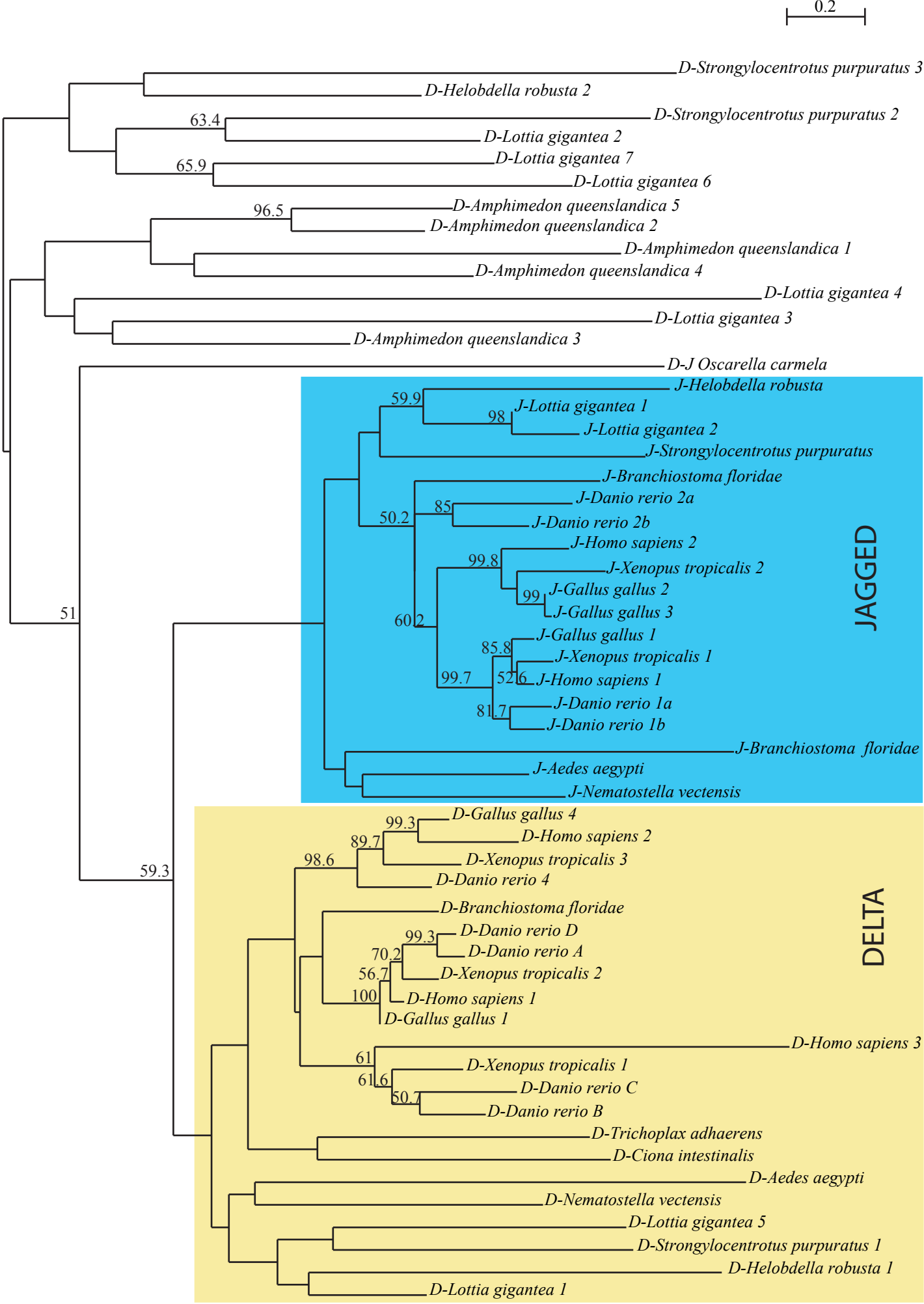

Supplement: Additional file 6 — DSL phylogenetic tree. DSL proteins phylogenetic tree constructed from the protein alignments using the maximum likelihood method (ML) with the PHYML program. [file 1471-2148-9-249-S6.PDF]
