# Supplementary material for: Origin and evolution of the Notch signalling pathway: an overview from eukaryotic genomes
Source: BMC Evol Biol. 2009 Oct 13;9:249. doi: 10.1186/1471-2148-9-249 (PMC2770060; doi:10.1186/1471-2148-9-249)
Supplement: Additional file 3 — Diagnostic domains table. In this table we report the presence or absence of the domains that compose each protein in all species. [file 1471-2148-9-249-S3.DOC]

**ADAM 10**

| **Species** | **Domains** | | **Species** | **Domains** | |
| --- | --- | --- | --- | --- | --- |
|  | **ZnMc** | **Disin** |  | **ZnMc** | **Disin** |
| *Aae* | + | + | *Hro* | + | + |
| *Aqu* | + | + | *Hsa* | + | + |
| *Bfl* | + | + | *Lgi* | + | + |
| *Cel* | + | + | *Mbr* | + | + |
| *Cin* | + | + | *Nve* | + | + |
| *Dre* | + | + | *Spu* | + | - |
| *Gga* | + | + | *Tad* | + | + |
| *Hma* | + | + | *Xtr* | + | + |

**ADAM 17**

| **Species** | **Domains** | | **Species** | **Domains** | |
| --- | --- | --- | --- | --- | --- |
|  | **ZnMc** | **Disin** |  | **ZnMc** | **Disin** |
| *Aae* | + | + | *Lgi* | + | + |
| *Aqu* | + | + | *Mbr* | + | - |
| *Bfl* | + | + | *Nve* | + | + |
| *Cele* | + | + | *Oca* | + | - |
| *Cin* | + | + | *Ppi* | + | + |
| *Dre* | + | + | *Spo* | + | + |
| *Ecu* | + | + | *Spu* | + | + |
| *Gga* | + | + | *Tad* | + | + |
| *Hma* | + | - | *Uma* | + | + |
| *Hro* | + | + | *Xtr* | + | + |
| *Hsa* | + | + |  | | |

**APH1**

| **Species** | **Domain** | **Species** | **Domain** |
| --- | --- | --- | --- |
|  | **APH1 superfamily** |  | **APH1 superfamily** |
| *Aae* | + | *Hro* | + |
| *Aqu* | + | *Hsa* | + |
| *Ath* | + | *Lgi* | + |
| *Bfl* | + | *Mbr* | + |
| *Cel* | + | *Nve* | + |
| *Cin* | - | *Pra* | + |
| *Ddi* | + | *Spu* | + |
| *Dre* | + | *Tad* | + |
| *Gga* | + | *Vca* | + |
| *Hma* | + | *Xtr* | + |

**Delta**

| **Species** | **Domains** | | |
| --- | --- | --- | --- |
|  | **MNNL** | **DSL** | **EGF** |
| *Aae* | - | + | + |
| *Aqu* | + | + | + |
| *Bfl* | + | + | + |
| *Cel* | - | + | + |
| *Cin* | + | + | + |
| *Dre* | + | + | + |
| *Gga* | + | + | + |
| *Hma* | - | + | - |
| *Hro* | - | + | + |
| *Hsa* | + | + | + |
| *Lgi* | + | + | + |
| *Nve* | + | + | + |
| *Oca* | + | - | - |
| *Spu* | + | + | + |
| *Tad* | + | + | + |
| *Xtr* | + | + | + |

**Deltex**

| **Species** | **Domains** | | **Species** | **Domains** | |
| --- | --- | --- | --- | --- | --- |
|  | **ZN finger ring** | **WWE** |  | **ZN finger ring** | **WWE** |
| *Aae* | + | + | *Lgi* | + | - |
| *Aqu* | + | + | *Mbr* | - | + |
| *Bfl* | + | + | *Mov* | + | + |
| *Cin* | + | + | *Nve* | + | - |
| *Dre* | + | - | *Spu* | + | + |
| *Gga* | + | + | *Tad* | + | - |
| *Hro* | + | - | *Xtr* | + | + |
| *Hsa* | + | + |  | | |

**FRINGE**

| **Species** | **Domain** | **Species** | **Domain** |
| --- | --- | --- | --- |
|  | **Fringe superfamily** |  | **Fringe superfamily** |
| *Aae* | - | *Hsa* | + |
| *Aqu* | + | *Lgi* | + |
| *Ath* | - | *Nve* | + |
| *Bfl* | + | *Spu* | - |
| *Cin* | + | *Tva* | + |
| *Dre* | + | *Xtr* | + |
| *Gga* | + |  | |

**FURIN**

| **Species** | **Domains** | | |
| --- | --- | --- | --- |
|  | **Furin like repeat** | **P-proprotein** | **subtilisin** |
| *Aae* | + | + | + |
| *Bfl* | - | + | + |
| *Cel* | + | + | + |
| *Cin* | + | + | + |
| *Dre* | + | + | + |
| *Gga* | + | + | + |
| *Hma* | - | + | + |
| *Hro* | - | + | + |
| *Hsa* | + | + | + |
| *Lgi* | + | + | + |
| *Mbr* | + | + | + |
| *Nve* | - | + | + |
| *Oca* | - | - | + |
| *Ppi* | - | + | + |
| *Spu* | + | + | + |
| *Xtr* | + | + | + |

**Mastermind**

| **Species** | **Domain** | **Species** | **Domain** |
| --- | --- | --- | --- |
|  | **Maml - 1** |  | **Maml - 1** |
| *Bfl* | + | *Hsa* | + |
| *Dre* | + | *Nve* | + |
| *Gga* | + | *Xtr* | + |

**Mindbomb**

| **Species** | **Domains** | | | |
| --- | --- | --- | --- | --- |
|  | **Znf ZZ ou ZZ mind** | **Zn finger ring** | **ANK** | **Mib / herc2** |
| *Aae* | + | + | + | + |
| *Aqu* | + | + | + | + |
| *Bfl* | - | - | - | - |
| *Cel* | - | + | + | - |
| *Cin* | + | + | + | + |
| *Dre* | + | + | + | + |
| *Gga* | + | + | + | + |
| *Hma* | - | - | + | - |
| *Hro* | + | + | + | + |
| *Hsa* | + | + | + | + |
| *Lgi* | + | + | + | + |
| *Nve* | + | - | - | + |
| *Spu* | + | - | + | + |
| *Xtr* | + | + | + | + |

**Nedd4/Sudx**

| **Species** | **Domains** | | | **Species** | **Domains** | | |
| --- | --- | --- | --- | --- | --- | --- | --- |
|  | **C2** | **WW** | **HECT** |  | **C2** | **WW** | **HECT** |
| *Aae* | + | + | + | *Mbr* | - | + | + |
| *Aqu* | + | + | + | *Nve* | + | + | + |
| *Bfl* | + | + | + | *Oca* | - | - | + |
| *Cel* | + | + | + | *Ppi* | + | + | + |
| *Cin* | + | + | + | *Sce* | + | + | + |
| *Dre* | + | + | + | *Spo* | + | + | + |
| *Gga* | + | + | + | *Spu* | - | + | - |
| *Hma* | - | - | + | *Tad* | + | + | + |
| *Hro* | + | + | + | *Uma* | + | + | + |
| *Hsa* | + | + | + | *Xtr* | + | + | + |
| *Lgi* | + | + | + |  | | | |

**Neuralized**

| **Species** | **Domains** | | **Species** | **Domains** | |
| --- | --- | --- | --- | --- | --- |
|  | **Neuralized** | **RING** |  | **Neuralized** | **RING** |
| *Aae* | + | + | *Hro* | + | + |
| *Aqu* | + | - | *Hsa* | + | + |
| *Bfl* | + | + | *Lgi* | + | + |
| *Cel* | + | - | *Nve* | + | - |
| *Cin* | + | - | *Spu* | + | + |
| *Dre* | + | + | *Tad* | + | - |
| *Gga* | + | + | *Xtr* | + | + |

**Nicastrin**

| **Species** | **Domain** | **Species** | **Domain** |
| --- | --- | --- | --- |
|  | **M20 dimer family** |  | **M20 dimer family** |
| *Aae* | + | *Lgi* | + |
| *Aqu* | + | *Mbr* | + |
| *Ath* | + | *Nve* | + |
| *Bfl* | + | *Ppi* | + |
| *Cel* | + | *Pra* | + |
| *Ddi* | + | *Pso* | - |
| *Dre* | + | *Spu* | + |
| *Gga* | + | *Tad* | + |
| *Hma* | - | *Vca* | + |
| *Hro* | + | *Xtr* | + |
| *Hsa* | + |  | |

**Notch**

| **Species** | **Domains** | | |
| --- | --- | --- | --- |
|  | **EGF** | **NL/LNR** | **ANK** |
| *Aae* | + | + | + |
| *Aqu* | + | + | + |
| *Bfl* | + | + | + |
| *Cel* | + | + | + |
| *Cin* | + | + | + |
| *Dre* | + | + | + |
| *Gga* | + | + | + |
| *Hma* | + | - | - |
| *Hro* | + | + | - |
| *Hsa* | + | + | + |
| *Lgi* | + | + | + |
| *Nve* | + | + | + |
| *Oca* | + | - | - |
| *Spu* | + | - | - |
| *Tad* | + | + | + |
| *Xtr* | + | + | + |

**Notchless**

| **Species** | **Domains** | | **Species** | **Domains** | |
| --- | --- | --- | --- | --- | --- |
|  | **NLE** | **WD40** |  | **NLE** | **WD40** |
| *Aae* | - | + | *Mbr* | + | + |
| *Aqu* | + | + | *Ngr* | - | + |
| *Ath* | + | + | *Nve* | + | + |
| *Bfl* | - | + | *Ppi* | + | + |
| *Cel* | + | + | *Pra* | + | + |
| *Cin* | + | + | *Pso* | - | + |
| *Dre* | + | + | *Sce* | - | + |
| *Ecu* | - | + | *Spo* | - | + |
| *Ehi* | + | + | *Spu* | - | + |
| *Gga* | + | + | *Tad* | + | + |
| *Hma* | + | + | *Tth* | + | + |
| *Hro* | + | + | *Tva* | + | + |
| *Hsa* | + | + | *Uma* | - | + |
| *Lgi* | + | + | *Vca* | - | + |
| *Lma* | - | + | *Xtr* | + | + |

**Numb**

| **Species** | **Domains** | | **Species** | **Domains** | |
| --- | --- | --- | --- | --- | --- |
|  | **numbF** | **PH-like** |  | **numbF** | **PH-like** |
| *Aae* | + | + | *Hro* | + | + |
| *Bfl* | + | + | *Hsa* | + | + |
| *Cel* | + | + | *Lgi* | + | + |
| *Cin* | + | + | *Nve* | - | + |
| *Dre* | + | + | *Spu* | + | + |
| *Gga* | + | + | *Tad* | - | + |
| *Hma* | - | + | *Xtr* | + | + |

**Presenilin**

| **Species** | **Domain** | **Species** | **Domain** |
| --- | --- | --- | --- |
|  | **Peptidase A22 b** |  | **Peptidase A22 b** |
| *Aga* | + | *Lgi* | + |
| *Aqu* | + | *Lma* | + |
| *Ath* | + | *Mbr* | + |
| *Bfl* | + | *Nve* | + |
| *Cel* | + | *Ppi* | + |
| *Cin* | + | *Pra* | + |
| *Ddi* | + | *Pso* | + |
| *Dre* | + | *Spu* | + |
| *Ehi* | + | *Tad* | + |
| *Gga* | + | *Tva* | + |
| *Hma* | + | *Vca* | + |
| *Hro* | + | *Xtr* | + |
| *Hsa* | + |  | |

**SMRT**

| **Species** | **Domain** |
| --- | --- |
|  | **SANT** |
| *Aae* | + |
| *Bfl* | + |
| *Cel* | + |
| *Dre* | + |
| *Gga* | + |
| *Hro* | + |
| *Hsa* | + |
| *Lgi* | + |
| *Spu* | + |
| *Xtr* | + |

**Strawberry Notch**

| **Species** | **Domains** | **Species** | **Domains** |
| --- | --- | --- | --- |
|  | **Dex-Hc = ABC-ATPase** |  | **Dex-Hc = ABC-ATPase** |
| *Aae* | + | *Hsa* | + |
| *Aqu* | + | *Lgi* | + |
| *Ath* | - | *Mbr* | + |
| *Bfl* | + | *Nve* | + |
| *Cel* | + | *Oca* | + |
| *Cin* | + | *Spu* | - |
| *Dre* | + | *Tad* | + |
| *Gga* | + | *Vca* | + |
| *Hma* | + | *Xtr* | + |
| *Hro* | + |  | |

**SUH**

| **Species** | **Domains** | | |
| --- | --- | --- | --- |
|  | **Lag1** | **IPT-RBJ-kappa** | **Beta-trefoil** |
| *Aae* | + | + | + |
| *Aqu* | + | + | + |
| *Bfl* | + | + | + |
| *Cel* | + | + | + |
| *Cin* | + | + | + |
| *Dre* | + | + | + |
| *Gga* | + | + | + |
| *Hma* | - | + | - |
| *Hro* | + | + | + |
| *Hsa* | + | + | + |
| *Lgi* | + | + | + |
| *Mbr* | + | + | - |
| *Mov* | + | + | + |
| *Nve* | + | + | + |
| *Spo* | + | - | + |
| *Tad* | + | + | + |
| *Uma* | + | + | + |
| *Xtr* | + | + | + |
