# Supplementary material for: Origin and evolution of the Notch signalling pathway: an overview from eukaryotic genomes
Source: BMC Evol Biol. 2009 Oct 13;9:249. doi: 10.1186/1471-2148-9-249 (PMC2770060; doi:10.1186/1471-2148-9-249)
Supplement: Additional file 4 — "Notch-like" in Monosiga brevicollis. The sequence of Monosiga brevicollis presenting a domain arrangement of a Notch gene is provided. [file 1471-2148-9-249-S4.DOC]

>estExt_fgenesh1_pg.C_30300 [Monbr1:35907]

MIVPHPAYLLALLLGLLAVLVPVEAQQACSTNAECRALYPCYQLTACLQQKCVIDSPRDSDTPCQLGEHA

GFCVASGHCRLTACQSEQRCRYGGLCEQDWLATEGNASAFTQDSSNTTSPLGSPLFGPTSEGCVCFPGYA

GPDCGQILPIAGSTNLSRLDSLASSTGANLSDWDPWRTCPACRASYADGVCNSACNTPSCFFDGGDCVAI

GTSLTKPCPAHCAATFADGRCDSECGGLATCAFDGGDCVGYDQEFVPIVQLLVDGVATVLDLLTLELELP

MLLRGPVLSLGEANPSTRAPTTAPTPGTRRLPTGRWFELAFAPGCTARSGPSACLNTSAQAAAALNAAVA

AGHAPRGLWGARAVASGLSLVTPEPGRLDNTSSVAKPQSRSPWARPLVIALVCVAAVCVILVGTVVRQAK

RRQHERLAPAPEEDGAVAGSASGDSPTRIVVANVHRVSKRPHIHHDTADRAAEYIDLAAQPQGRAARQSA

SRTSSRARPGMHGRLRHNQVTDIDTCAPPTPPPRDYDEALESDDSHVPADAWTEDRSLPTKRSMAPPHLP

TQHLAPAVSPPVLPDSESLRPSAALLTAPARDVSLPGTANSPYSDDLDFLGALADRENGDGPSLGHYTAR

AGLPAESTTPSSSMGMTSPLHADPWLAGAQPNAEPSIFSIDSGYTRSIASGVTDGSGHDQRKGQDHARYP

SSPAARATSRHRSGDHALWFQAAAQGREEAIRQLFARDPKGINRPDDVGRCPAHFACTATNLTMLRLLVE

ELGAFVDGAAPGWHGQSPLHVACAQDWAEGVNFLIQQDADLNLRDENGVTPLMVCVKLKAQSCLMPLLRS

TRHYIFLEANKGPSRLRARKADAPAALLRAGQRLPFTVVDATDQQGWTALHYAAATGNMAAAMALLRHQA

NVNAANKHGATALHLAAREHHEPVIRALLQHGAERHIVDDSLRSAHDCLPTFVQGDLRVALCPPAPAVS*
