# Supplementary material for: Origin and evolution of the Notch signalling pathway: an overview from eukaryotic genomes
Source: BMC Evol Biol. 2009 Oct 13;9:249. doi: 10.1186/1471-2148-9-249 (PMC2770060; doi:10.1186/1471-2148-9-249)
Supplement: Additional file 7 — Unusual arrangement of DSL domains in Nematostella vectensis. In this file we report the sequences of Nematostella vectensis presenting an unusual arrangement of DSL domains. [file 1471-2148-9-249-S7.DOC]

>jgi|Nemve1|212534|fgenesh1_pg.scaffold_157000053

MVIRYSVQCTRDYYGPNCTTHCIPRDGTSGHYTCDLKTGGRVCRPGWHGPQCRVYCIPRDDDVNGHFTCQ

TGTGLKICLANWYGVLCKTYCAPINNSDAGYNCDVTGSRVCLPGWYPREQCETLCIPHNDSTASYTCDSQ

TGSKTCLSGWSGPECDCRPRLDASAGYTCNETTGQKICYPGWYGRNCNVACSPRNDSTGHYNCNADTGAK

ECLESWIGEQCDVYCVPDNQTHSCLANGTRKCQQHWYGQQCHVFCQPRPIQYTCQSTTGAKVCSQGWYGE

DCSTFCKARDSDWFGHFDCDRNGSRVCHMYWHGPECKAYCKPHRNSTLGYYRCDDHGNPLCERDWYGTKC

TVYCRPRDDEEGHYSCGAVDGRRVCLEDWYGENCTVHCVADKEGHFTCDGKTGQRVCLPGWGGQDCQQGK

AHLGRTLVGRVAQIQKKTE*

>jgi|Nemve1|11703|gw.288.14.1

SCNATTGEKICHAGWYGNLXEKICHAGWYGNLCDTYCIPTNDSSGHYSCNATTGEKICHAGWYGRLCDTY

CIPTNDSSGHYSCNVTTGEKICHAGWYGRLXEKICHAGWYGRLCDIYCIPTNDSSGHYSCNATTGEKICH

AGWYGSLCDIYCIPTNDSSGHYSCNVTTGEKICHAGWYGRLCDIYCIPTNDSSGHYSCNATTGEKICHAG

WYGNLCDIYCIPTNDSSGHYSCNATTGEKICHVGWHGKLCDTYXMICHAHWYGESCSTNCSPQNNSLGHF

ECDPQSGIKICHSGWFGPICLKRCHDNWYGENCTVFCKALSSDHQHYNCDNTTGSKLCHPDWFGENCTRI

CLDDWYGGNCTTYCKSSNGSYYYCKHDSGKRVCLDDWHGERCNKICLPNWYGVNCSKYCKEKDDNSGNYR

CDNSTGEKICLPNWYGKICLPNWYGINCIKYCKEKDDNSGNYRCDNSTGEKICLPNWYGVNCSKYCKEKG

DNSGNYRCDNSTGEKICLPNWYGVNCSKYCK

>jgi|Nemve1|60816|gw.21.273.1

WYGPDCTTNCTPRNDSFGHYECDSSTGAVVCHSGWYGPTCTVNWAKICRNDWYGVNCTEYCTPRNKVGGH

YSCDMATGAKICDSNWYGLNCTRFCKPQDSLLGHYNCSVNGSKVCHREWYGATCDVHCVPRNDSSGHFSC

NTTSGEKVCLRDWYGDRCEIFCLSRNDSLGHYSCNANGTKVCHKDWYGDLCARICFPNWYGVNCTRFCKP

DNESTFFCDKSTGTRVCNAGWYGINCTTQCICHENWYGQACTTFCEARNDSTGHYSCNLTNGQKVCHRDW

YGPFCDVNCNSNSSHFTCNKTTGARVCHSNWYGTYCDVYCNSNSSSHFACNKTTGARVCHRDWYGSLCNV

SFCLKNWFGPRCDVFCESDNSRYVCAPGNGSKMCLPGWTGANCLNP

>jgi|Nemve1|212543|fgenesh1_pg.scaffold_157000062

MGPGEISHIWRQFSYKQGDLYHNEGPQVFRVRDFAFSCDRDTESSREHKRRDSGEMLRTNLKVFRVAGPS

VFLRSGYEFSRRTHAGFEKMLRMNCTLVSQSLERRFTNECVVASYHGSEKETFDAWDRESVEAESGGHVS

VVFKRYRNPTRRRYDGHCCEHVFWSRCGTCDTYLKICLTDFANPRSIKHCPLGSVRTKRLGRDDFRFHLR

ITRAFTRFKGNIGLYVESWDHDTFTADDLVDKLSLTLHLTRPDPNNHVTSLYRRTLQGRRASLSAELNVY

CDPHYYGTACATYCRARDDQYGHYTCNLHGEKVCRPTWHGANCLARCVEYDDSARGHSKCDANGRRVCRE

GWYGKNCLKYCMERDDHQGHYTCDMMGEKVCREGWYGDDCRIHCVPTESDVQGHYSCDGAGRRVCNDGWH

GDKCSVYCKETDDERGHYTCGVKGQRVCHVGWALPNCKTCAQGYNPPNCTAPCVPANNSTQGHYTCDKQD

NKKCRMWWHGVNCTTYCVPHMDPVHGHYTCDSHGNKTCHPGWYGDSCSKKCEDASTELSCQGLKQCKPGF

ARPDCTECEPGFFGVNCSKSCLVGKLGNVVCGASGERKCRKWWYGVDCDVYCVPHDDDTNGHFTCDSNGS

WRCILGWAVPECKLKESHKAMAREADVGLFSVN*

>jgi|Nemve1|119841|e_gw.165.66.1

QCNKNYYGPECSMKCQQKDNIEGHYTCDEATGRKICLPGNVLASCTGIKTCLPGWHGDRCNIYCRKTPTS

ICDGSGKKYCLPNWYGKDCSKYCAPTGNFACNSLTGERGCVAGWYGDQCDRYCIATHDDTAAFSCSETGE

KVCHKGWYGTNCERHCVRTRNSVCSNITGERICNKGWHGVNCDTYCMPRNDQNAAYSCDKDGKKACFPLW

YGSECDKSCKFGWYGPQCDCSPKDDKTAGYTCDKLTGVRVCQPGWTGFMCDSRGN*

>jgi|Nemve1|148964|e_gw.2717.2.1

RCDNSTGEKICLPNWYGVNCSKYCKEKDENSGNYRCDNSTGEKICLPNWYGVNCIKYCKEKDDNSGNYRC

DNSTGEKICLPNWYGVNCSKYCKEKDDNSGNYRCDNSTGEKICLPNWYGVNCIKYCKEKDDNSGNYRCEN

STGEKICLPNWYGVNCSKYCKEKDENSGNYRCENSTGEKICLPNWYGVNCSQYCKKKDDNSGHYKCDD*
