# Supplementary material for: Origin and evolution of the Notch signalling pathway: an overview from eukaryotic genomes
Source: BMC Evol Biol. 2009 Oct 13;9:249. doi: 10.1186/1471-2148-9-249 (PMC2770060; doi:10.1186/1471-2148-9-249)
Supplement: Additional file 8 — Strawberry notch sequences. In this file we report the Strawberry notch sequences identified in two heterokonts. [file 1471-2148-9-249-S8.DOC]

*Strawberry notch* sequences from Heterokontae

*Emiliana huxleyi*:

>1

MEDDGDVEITGGSGVNALRDMPHPRHDCVVHKWPQGPPAQNKDACKQFCAKCFCFVCDIEASRCEHWDQHCLADGSVEWLKKRQQRQRTEEARRKGAQAGYVPRPADEMRRSFAEAANEENRQQDMSQDEEAAAQVREQAAVEDEDAEDMFTATYEPFHFKRGQPHPDPVVETTSLSFAEPPPIKYDLNLPDGIFEPRDELDNPAGGALSNLQLETVAYACQRHESIMANGCRAGFFLGDGVGLGKGRQLAGVIVENWLCGRKRHLWVSVSADLMEDAKRDLKDIGFSHIEVKNITKLDYGKLDKKGPKGIREGVVFATYSALVSSQRGGKTRAAQIAEWLGGAQAEGCILFDESHKAKNLAPEKNEKGKPTTKKSSKMALTCQKLQADCPRSRVVYCSATGASSLQNMAYMERLGLWGAHTAFPDFNAFDKAIGGGGVGAMELIALDLKRRGMYLCRQLSFSAASFDTKMVDLTPRQEKMYDEAAAFWSELLCAFNYAVHEAFGPPPRGGAASSRATRREVLNLKEVKGPPNPTTGKPKPHPSSRVIGHYWGCHQRFFRSLCMAMKVPELVKMAKGALADNKCVVVGLQTTGEARLNDAVKSGEDLEEFAGMKECVRFLLKKFPTGDYTNQHPESDLDDGDESDDDDLTREVAGATSRVNAGKVARAAVKRSGRLANGGRGSDEEGSDDEDDDLDGFIVRDGQEEESEDEDYEATDDEEGDGEQVRLPAACKKMPAGQLRQLLRAAKMNDQQCTGRAKLVEQLKQVERQARSSGGQRVADLLAKAGLGGSKRPSPDSAQASGRPKRRLQRGAEKKSYIELSSEEEEEEEEDVEMGEAETGDYFVGKELSIEQEGGGRRVATVVAHYPSTGHHRVRYADGGGGAEDRVLLKEVKWDYHRKQAAPGASGARRAVQDSDSEALPEAARARDLLEAACSGASENAPPQRSSKGKARKSAGKARRVAADDSDASWSGGSGDEDEEEVARPTNRASRSKSPLPAPTKSAAKPKPKRRKSYGSDDDLLSESEEEEEDLSWAQHSNPAEIKMLQRLKKQLVRKLNALKMPDNPLDKLIEELGGAESVAELTGRKGRLVRDEEGRTVYQKRNANEVDSDTGRMVAMERINLAEKKAFMDGRKLVAIISEAASRCIQQCGRTHRSNQIHGPEYQLFMTACGGERRFASTVAKRLQQLGAITKGDRRAADASDLSMFDVDTKWGKKAYDELIELLSNPLFSSTRPPPPYVRRALGLKANDELHARWKEYLDDAEEAVASVGINTDDKGSVKGFLNRLLGMCVKMQNKVFDHFMAILEEKVRVAKQNGEFDDGVVDIRGESVKVAANFPREMKKDPISGVPLLHFKLDVDRGLSFKRAMEMLDEKAKSNETGKLYPREGFYVSKRPLIGREKEGPNGVKALAFLIQRPMSAFAVPSTYIHTYGIHRPNTGLGATDSFESFTEGITPRFSKLTPEAAKDKWEALYRDGLRMCSHGPNCKHGSRCTVGKRVQQQHLICGAVIPFWAVIKEHLEYQKKGGRDGSLQMVKVMRIVRVRCVGADGREQRLVGVEISDKGVAKLDAALNNQPAPQDVKPDVKPDLHQLGRGGASSSSSHGGGASSSDVKPDVRGLRVGAHVMVHGLATDAGKKLNGKRGKIERWDAAMQPSGRWIVRIDGQQGTNALQAKNLGAL

> 2

MIPAQPPPPSDGDAAADPDAAAEARRQQLDEANLGVVSEELFVSFEAAAETPGLPHPADVAEPASLAATPPPQCTYPLLDALPSNLVSSGALSSLQLQFVGLACQRHLTVMPTDPPTRAGFFLGDGAGVGKGRQLAAVILDSLARGRPRHIWFSSSADLRNDAIRDLTDLGCHCPAGVLFSTYATLARGSSRLSQLVAWCGGLAFDGCLLFDECHKAKNWTGKEETSSKVAAAVRELQRALPLARVVYCSATGASDSGLSLACTTTCLCAEFELKEAPLSAEQASAFDAAAAFWSQQLLPALEAAAERTGAQAGATVRAFWGAHQRFFRQLCVGFKVPALVAEVRAALGSGSCVVVGLQSTGEAALERVGDSASGELYSLCAEMLRSFIAHNFPTTRHADKELAKSLAKAEAAAARAIEAHAAARAAAARPEASRSATARAEAAEAVRGAAAMAAAAAAAVQRLRAEAAADAEAAGQADPECEVAKARLLQAAAELELPPAALDLLIDECGGAAAVAEMTGRKARVALNVTERNAFMDGKKLVAVISDAASTGISLHADARSANQRRRVHITLELAWSADKAVQQLGRSHRANQTSAPRYVLLCTDVGGEARFGSSVARKLEAMGALTKGDRRAEIGSLDTLDVDTAPSSLDTFNLDTAWGRKALKALLAAAYAGDSSVLPHLAADSAAIEQLFDLFAAALREVVSAAKRDGRYDAGVADISAGARLLEPEALIWRDPITGARAHAATVEVDRGVSFADALSKLRSWRAAAAAAKRNTDGGDAAADSREGGGAEEGGGVEAGEEEACVEEAGEEAAPAAARSGDVEDGEEEEGEGEGEEEGGGIFRSRRPQYGEHLLLLALPKAREPHMAVLTRPNTGLSLYEEAWTDLHRKYARVLPPSAAEADWTAAYNGACNERIGGRLTRIQLITGSTLPMLPVLEEVAKKHSANLTARDRAVGAVRVILDGRRLLGVRFARSCMADLKEELLKWNLARGATHRKVETEPAAPVDPKALARALRPPATITSFFAARPASQAAAPAGTGTGGKQPAGGRGGAGAGGKKRSVLDMMKGSSSSSSAAASSALAAVPKATKAPKASGGLDVRSFFTSKSADSGPRSPATRPSSEEPIDLTSPGGD

>3

METSLALLRYEPTTPCLDATLTVAESSRGGDKQTGHVNVVCDIEASRCEHWDQHCLADGSVEWLKKRQQRQRTEEARRKGAQAANEENRQQDMSQDEEAAAQVREQAAVEDEDAEDMFTATYEPFHFKRGQPHPDPVVETTSLSFAEPPPIKYDLNLPDGIFEPRDELDNPAGGALSNLQLETVAYACQRHESIMANGCRAGFFLGDGVGLGKGRQLAGVIVENWLCGRKRHLWVSVSADLMEDAKRDLKDIGFSHIEVKNITKLDYGKLDKKGPKGIREGVVFATYSALVSSQRGGKTRAAQIAEWLGGAQAEGCILFDESHKAKNLAPEKNEKGKPTTKKSSKMALTCQKLQADCPRSRVVYCSATGASSLQNMAYMERLGLWGAHTAFPDFNAFDKAIGGGGVGAMELIALDLKRRGMYLCRQLSFSAASFDTKMVDLTPRQEKMYDEAAAFWSELLCAFNYAVHEAFGPPPRGGAASSRATRREVLNLKEVKGPPNPTTGKPKPHPSSRVIGHYWGCHQRFFRSLCMAMKVPELVKMAKGALADNKCVVVGLQTTGEARLNDAVKSGEDLEEFAGMKECVRFLLKKFPTGDYTNQHPESDLDDGDESDDDDLTREVAGATSRVNAGKVARAAVKRSGRLANGGRGSDEEGSDDEDDDLDGFIVRDGQEEESEDEDYEATDDEEGDGEQVRLPAACKKMPAGQLRQLLRAAKMNDQQCTGRAKLVEQLKQVERQARSSGGQRVADLLAKAGLGGSKRPSPDSAQASGRPKRRLQRGAEKKSYIELSSEEEEEEEEDVEMGEAETGDYFVGKELSIEQEGGGRRVATVVAHYPSTGHHRVRYADGGGGAEDRVLLKEVKWDYHRKQAAPGASGARRAVQDSDSEALPEAARARDLLEAACSGASENAPPQRSSKGKARKSAGKARRVAADDSDASWSGGSGDEDEEEVARPTNRASRSKSPLPAPTKSAAKPKPKRRKSYGSDDDLLSESEEEEEDLSWAQHSNPAEIKMLQRLKKQLVRKLNALKMPDNPLDKLIEELGGAESVAELTGRKGRLVRDEEGRTVYQKRNANEVDSDTGRMVAMERINLAEKKAFMDGRKLVAIISEAASRCIQQCGRTHRSNQIHGPEYQLFMTACGGERRFASTVAKRLQQLGAITKGDRRAADASDLSMFDVDTKWGKKAYDELIELLSNPLFSSTRPPPPYVRRALGLKANDELHARWKEYLDDAEEAVASVGINTDDKGSVKGFLNRLLGMCVKMQNKVFDHFMAILEEKVRVAKQNGEFDDGVVDIRGESVKVAANFPREMKKDPISGVPLLHFKLDVDRGLSFKRAMEMLDEKAKSNETGKLYPREGFYVSKRPLIGREKEGPNGVKALAFLIQRPMSAFAVPSTYIHTYGIHRPNTGLGATDSFESFTEGITPRFSKLTPEAAKDKWEALYRDGLRMCSHGPNCKHGSRCTVGKRVQQQHLICGAVIPFWAVIKEHLEYQKKGGRDGSLQMVKVMRIVRVRCVGADGREQRLVGVEISDKGVAKLDAALNNQPAPQDVKPDVKPDLHQLGRGGASSSSSHGGGASSSDVKPDVRGLRVGAHVMVHGLATDAGKKLNGKRGKIERWDAAMQPSGRWIVRIDGQQGTNALQAKNLGAL

>4

MDTTGTSAVWALRDMPHPRHDCVVHKWPQGPPAQNKDACKQFCAKCFCFVCDIEASRCEHWDQHCLADGSVEWLKKRQQRQRTEEARRKGAQAANEENRQQDMSQDEEAAAQVREQAAVEDEDAEDMFTATYEPFHFKRGQPHPDPVVETTSLSFAEPPPIKYDLNLPDGIFEPRDELDNPAGGALSNLQLETVAYACQRHESIMANGCRAGFFLGDGVGLGKGRQLAGVIVENWLCGRKRHLWVSVSADLMEDAKRDLKDIGFSHIEVKNITKLDYGKLDKKGPKGIREGVVFATYSALVSSQRGGKTRAAQIAEWLGGAQAEGCILFDESHKAKNLAPEKNEKGKPTTKKSSKMALTCQKLQADCPRSRVVYCSATGASSLQNMAYMERLGLWGAHTAFPDFNAFDKAIGGGGVGAMELIALDLKRRGMYLCRQLSFSAASFDTKMVDLTPRQEKMYDEAAAFWSELLCAFNYAEAAEKESCLQRQFSFWAASFDTKMVLNLKEVKGPPNPTTGKPKPHPSSRVIGHYWGCHQRFFRSLCMAMKVPELVKMAKGALADNKCVVVGLQTTGEARLNDAVKSGEDLEEFAGMKECVRFLLKKFPTGDYTNQHPESDLDDGDESDDDDLTREVAGATSRVNAGKKMPAGQLRQLLRAAKMNDQQCTGRAKLVEQLKQTGDYFVGKELSIEQEGGGRRVATVVAHYPSTGHHRVRYADGGGGAEDRVLLKEDLLEAACSGASENAPPQRSSKGKARKSAGKARRVAADDSDASWSGGSGDEDEEEVARPTNRASRSKSPLPAPTKSAAKPKPKRRKSYGSDDDLLSESEEEEEDLSWAQHSNPAEIKMLQRLKKQLVRKLNALKMPDNPLDKLIEELGGAESVAELTGRKGRLVRDEEGRTVYQKRNANEVDSDTGRMVAMERINLAEKKAFMDGRKLVAIISEAASSGISLQADRRCIQQCGRTHRSNQIHGPEYQLFMTACGGERRFASTVAKRLQQLGAITKGDRRAADASDLSMFDVDTKWGKKAYDELIELLSNPLFSSTRPPPPYVRRALGLKANDELHARWKEYLDDAEEAVASVGINTDDKGSVKGFLNRLLGMCVKMQNKVFDHFMAILEEKVRVAKQNGEFDDGVVDIRGESVKVAANFPREMKKDPISGVPLLHFKLDVDRGLSFKRAMEMLDEKAKSNETGKLYPREGFYVSKRPLIGREKEGPNGVKALAFLIQRPMSAFAVPSTYIHTYGIHRPNTGLGATDSFESFTEGITPRFSKLTPEAAKDKWEALYRDGLRMCSHGPNCKHGSRCTVGKRVQQQHLICGAVIPFWAVIKEHLEYQKKGGRDGSLQMVKVMRIVRVRCVGADGREQRLVGVEISDKGVAKLDAALNNQPAPQDVKPDVKPDLHQLGRGGASSSSSHGGGASSSDVKPDVRGLRVGAHVMVHGLATDAGKKLNGKRGKIERWDAAMQPSGRWIVRIDGQQGTNALQAKNLGAL

>5

MTSPHKSKPTYAAREETALEAAVNREWNAAQRLLSVLATAESVETHVPELHGAIFCGHLVADLDSVAGAIGAAALYGGVPARASEVNSETEFALATWGIDLSRIRPVEQLLEEQGDSARVCLVDFQQTTQLHPAIQPSKIVGVIDHHALQSSTIVTQRPIFVDIRPWGSMSTIIAHSFVAPVHVAFAVVETTDAAGMLARVPELELSSKGKPVDAFFLAVVDIVELETHPLSSETLKLAGKESARSLYRLAAGQVSRKADFVPPLTDAVAQGWSPKLLQPNRSEFDLAALAEQSEDATAPSGKAGAQKSVLDPLERCARGDSRFAVAEEIDGINRAQGVHHDDVTVQNLSTHLLQSQGATGGANHGSNLTNARFVNAFLGEADFGEALAPGGDFTGAEATKADFIKADLRGASFRSANCVDANFGGASIAGTDFLGAELRKAVLEPRHQHERAAGSSRGFDAGSLASMLQKQQHHAPSVHHLAQQSHQSHQLAAVARAEQQAQYEQLQQRRAQAQAQARAQQAQQAQQAQLQMRRQLQLQQQQQQSRHGAPSAYGGRPGSGGMPQQQQQQPKPQPTPQQLLAYAERVMGPGNLGLPPEKFMKNMEALRQMSLPQLYENAKALKVKLDQHKMMQRQQVLQQQQQQLMMQRQQAQQQVMQQHALQQQKAKEAAAEARCPSHPTSEYATDFKFSRFYAEPLATSGPALLLDDMSTAVYPLHKLPYGPLDPADYGDGVMFCTYQSLIAQNKHSERRIDQLVTWAAASASGDPARFEGCICFDEAHRAKNLALDRGGKGTKTGEMVLGIQKQLPQARVLYVSATAAAEVKDLGYMSRLGLWGRGTPFPDFSTFAAKIERAGVGAMELLAMDMKARGMFVSRMLAFTGCSFEVRECTLDQQNERLYDEAVELWDDMLTALEKCVDMCGSDGAHPLRLYWGAHQSFFKQLLNSIKARNAIDETEEALRQATISPHISRISPHISLTEEALGRERKGKCVVIGLLSTGEAKANEAAERENRAGRELDSEVSTPQEIARSLIEQHLPTTRAEGAPRDGGGLTNGFELLEQPAVPEAVQIRDKLLRRLDAIDMPSNALDLVIGHFGVEHVAEMTGRKMRFVKDGAGGTRWEARAQNGVSQDQINIAEKDAFMGGTKRVAIISDAASSGISLHADPRFANTRQRLHITLELAWSADKMLQQFGRTHRSNQITAPHYVLLVSNVGGEKRFASSVARRLEMLGAITRGDRRGGHGAAADLVQYNLDTVHGHTALALMFDAVAEKSEAAALWERALRMLQCHRQGVPTLLGLAVVAVAQKKQAQGNRDVARLPQGLQKYVLKLREWASQRQSGSSRQRPQERNVLKRMRVLNANLATQTDADRHNINKFLNRLLGLPVQTQNALFEYYTSVFKWVVVTAKAGRLEPPCPFPLMDGEPAPAHAQATGRLEPSIETISGESVSLATEPEVLHKEPNSSALTVHYTLSVDSGLSLASANGALTEALKSRAVKSTQQGGATGFWQQTRSQRLILALEMSSGGEAAAPTRRSDIAPRAGQRLFRVLRPTLHTSTKPQYIRHKDLRARYRPAEFVFANEDEYGPGTIVDEDDDNAVLEVAAEQARAADSLAAAMKSNDTARLRESLVAARAASVDAALLASGDARLAWLVAAEDDDQFDDDDLMNMLE

>6

MADLDDDVEFTGSTGTNPLRDMPHMRHDCLVHPWPRPATAQSCAECCAKCFCYVCDAPASECKLWDEHCLADGSAEWVRKRAQAKRKREAAQRAQAAGRQVDSAEAVRQRFAAAADTDDGGAPQPAPDGADAGRPTRDEAEVEDEESEETFATYAPCHFARCRAGFFLGDGVGLGKGRQLAGVIAENWLCGRKRHLWVSVSADLMEDAKRDLKDIGFSHIKVFNITKLPRGRLAAMRRGGNGVPHDWSADCVVFSTYSALIAKDRSKQTRAEQLVAWLGSAQAEGCILFDESHKAKNLAPEKGKKCSQTAADCPSARVVYCSATGASSLQNMGYMQRLGLWGAATPFADFRAFTKALGQHGKKGEAPVGELTAACGDARVARGERAVSRTVGAMELVALDLKRRGMYLSRQLSFSAASFDKAMAARFWADMLACFNYALHDVLRVKNDFPDRPKDAKHPSWAVISSFWGCHQRFFRSLCMAMKVPELVKKAKEALAANKCVVVGLQSTGEARLTDAVKSGEDLEEFAGMKECVRFLLKRFPTGGHVHEPAPTAGEWCDYMNMHPESEGEDEDEDEEDVMAEVAEGAARAADSAADAARRMRRKRQGGVGDSDEEEEEDDDLDGFIVRDGDGEESEESGSGSESGSEACGAEEGGGGGEGTSRRGRLPAAVRKQLKAMDAAQLRKLLQAANVADHQCFERGQLIERLKEHERAAAHQGGASLRVAALLVKAGFGDKGKRAAPETGGARAAAPQRRRLSTSSLPRYDAGDSEGEEGEDGGGGSPMDLDATDGEHGLVGRQISLRGEGGARRRATVESYDSRTQLHGVRYADTSAADQVDLASGADWSFLRTQRPPGTNPERGADSDSEDLLEQALRGGGKSAGKPGVKSGGGKPGARRAAAGGVRRASAGDESESSDPEWRGSDDESDGEDGEDGSGSEADFIRERAVAARPPAPSASRSAAPSASKPASSSATKAAPPPVRRPPAGSDDEAAAEDLSWAQHGNVADIKRLQRLRRQLFRKLRSLRMPSNPLDNIIHELGGPDAVAELTGRKGRLVRDEEGRTVYKRRNVDLGCSMERINLAEKQAFMDGRKLVAIISEAASSGISLQADRRVPNTRKRLHITLELPCASSSAAALTARTKSTRRFASTVAKRLQQLGAITKGDRRGADGQQENLSAFDVDTEYGQAALTDLIEVFENPIHNASIVPPGHIRRAIGLRATHELQQKWSEYIDEAEEAMASAGIFGMENLSVRRFLNRLLGMPVRMQNTIFQHYTAILDENVKAAKAAGRYDDGVVDIRGESVSVKSGSPRTISTDRQSGVPLSHYALGVDRGLSFERASEMLRAKAEGNEAGALLEGEGFYISRNFKFGNGCKGVTLLLRERVSVFAASNWIPTFKIYRPNQGALPGTLALLAPPSTPPARASPDAAGLCGRTFLREHLNSNLPLSSNGARGPWTRLHDATRDKCTHGHKCRDLQCFVGKRMQTEHLVCGAVLPFWSTIQQRVLSKTVRTSGGGFQLVQQMRVVRVRCQRADGTETRLVGSPLPGYQRRSIHMTHGGAARVQAIEMDIRGADDADGKPDLKPDLKPDLHSLGRGKQAGKGKGPAAPRQPSHGRCQECGAAHDGGYGSGRFCSQPCRSRHNGRPISSAAPPAPGVPRFGSSRPSSSSASSSSSGRGGRPSSSSSSAASSASAPSSSSAPARVKLGARVVLHGLCSAAGAALNGLYGTEVARVSFDSRGGPPFLLVRHAAASVRSIGALTRPRGTERDSRAGISCSMLRIRASDPATSSPMWISRSVGAKAALAVASITAALTFFPPRRSSVRLAAPTQSSSRVSPLLLTASEDRIHMRESAVRQREAELREALASPSGYGAGTHGGCGEPLWDDSVTAAVEVERCRADSPYLWQLAEPSQYSAALERVLARPGGPAMLCGAGGEDGLFGAFTVSVPAAGGSGQTVTVSRDLLDSANELAFLESRLGCSSWPLGTAVVDLGAGYGRLEHRAAAALPHLRLLATDAVPLSLALCEAYLAARGVPCSRAKVVLPAALPAEAAAAQPRLAVAVHSLQEMPLAAVRWWLEALAAARVRHLFVATNTAALEPPTATLRLATDAGEDLLAALEEAGFRLARAEPKYSPGSGGGGGGGDAPATVPAFGDCTYCLFELAGPLPAQLTPAQLLPARPPAGSASVVNLASRADRRRWVEASCATALRELGVEAGAGRDPPEIRPATSARDPPEICPRQLSEGDLSTLLLPLAQVRLVDACSPEDVRISGEGGGGGDGSSAWRPFAGWALGAAQLERLCDAWEAMGMPRPADAELRTYHGRAPLAGAPGKRAVSAREATGHDYVLFLEDDASVAGASGEVPSAADNRRGWARAWGTLQRQASLLAARGVAWDLLYLGRHRLACDSAAEGEALVELPLQSAAMPVDELLPALYAPHPRADVAAWAREALRGTDPFAAFAARDDLVHQLESLSDDGKGNWPGLPSWAQLARRVSRGLRAAVDEPTLWRGLVRASGGSRDQPRRLSAVRTLHRGGRAAVEDEVDLAAAAAAEPGRQLGRGQGGKEASGKQQQQQQQQQQATASDPIDLSRLLSGRRQLPPEAPRRRASPAWLREPRLLRGTPPVRSLERWALEGLLAAFAPHSFAVADQGAASAPLVRMSLLDFVAYARLQAGAAPALCVGGGAGACGAVVGCPLEAGQRGGEEANGAEEGPGKGRWADGEPLVLFERELPSELLADVAVPDAFAQDLLAEAAEAVAAQRESRGEGCGGGDGGGTEAAPLEVGVDLLNEREWLVVSPSRSGTRWHTDPYETSLLHGRKLWCLLPPSAALPPGVAIGASSTAAAGASLDAPAASEWFRQRLAPQDSPGDTSGTRHDVSPLWIDQRPGDVVFVPRGWWHTTLALDTCVAFTQNMVLPADARATLQALDAEMFQARAAGALRRAASALMLSVARWPRLMEATPSSQPRITVPAPSVKEKGARSRELSKTSSDLASRPV

>7

MPARLGTEPMINAQPPPPPPAPPSDGDAAADPDAAAEARRQQLDEANLGVVSEELFVSFEAAAETPGLPHPADVAEPASLAATPPPQCTYPLLDALPSNLVSSGALSSLQLQFVGLACQRHLTVMPTDPPTRAGFFLGDGAGVGKGRQLAAVILDSLARGRPRHIWFSSSADLRNDAIRDLTDLGCHCPAGVLFSTYATLARGSSRLSQLVAWCGGPAFDGCLLFDECHKAKNWTGKEETSSKVAAAVRELQRALPLARVVYCSATGASDSGLSLACTTTCLCAEFELKEAPLSAEQASAFDAAAAFWSQQLLPALEAAAERTGAQAGATVRAFWGAHQRFFRQLCVGFKVPALVAEVRAALGSGSCVVVGLQSTGEAALERVGDSASGELYSLCAEMLRSFIAHNFPTTRHADKELAKSLAKAEAAAARAIEAHAAARAAAARPEASRSATARAEAAEAVRGAAAMAAAAAAAVQRLRAEAAADAEAAGQADPECEVAKARLLQAAAELELPPAALDLLIDECGGAAAVAEMTGRKARVALNVTERNAFMDGKKLVAVISDAASTGISLHADARSANQRRRVHITLELAWSADKAVQQLGRSHRANQTSAPRYVLLCTDVGGEARFGSSVARKLEAMGALTKGDRRAEIGSLDTLDVDTAPSSLDTFNLDTAWGRKALKALLAAAYAGDSSVLPHLAADSAAIESARAGLDAAQKGSEKELEVRRFLNRMLGLPLAQQRQRFHLSAAPRCGGGVLIGVVVESAHAATVEVDRGVSFADALSKLRSWRAAAAAAKRNTDGGDAAADSREGGGAEEGGGVEAGEEEACVEEAGEEAAPAAARSGDVEDGEEEEGEGEGEEEGGGIFRSRRPQYGEHLLLLALPKAREPHMAVLTRPNTGLSLYEEAWTDLHRKYARVLPPSAAEADWTAAYNGACNERIGGRLTRIQLITGSTLPMLPVLEEVAKKHSANLTARDRAVGAVRVILDGRRLLGVRFARSCMADLKEELLKWNLARGATHRKVETEPAAPVDPKALARALRPPATITSFFAARPASQAAAPAGTGTGGKQPAGGRGGAGAGGKKRSVLDMMKGSSSSSSAAASSALAAVPKATKAPKASGGLDVRSFFTSKSADSGPRSPATRPSSEEPIDLTSPGGD

>8

MSRLGLWGRGTPFPDFSTFAAKIERAGVGAMELLAMDMKARGMFVSRMLAFTGCSFEVRECTLDQQNERLYDEAVELWDDMLTALEKCVDMCGSDGAHPLRLYWGAHQSFFKQLLNSIKARNAIDETEEALRQGKCVVIGLLSTGEAKANEAAERENRAGRELDSEVSTPQEIARSLIEQHLPTTRAEGAPRDGGGLTNGFELLEQPAVPEAVQIRDKLLRRLDAIDMPSNALDLVIGHFGVEHVAEMTGRKMRFVKDGAGGTRWEARAQNGVSQDQINIAEKDAFMGGTKRVAIISDAASSGISLHADPRFANTRQRLHITLELAWSADKMLQQFGRTHRSNQITAPHYVLLVSNVGGEKRFASSVARRLEMLGAITRGDRRGGHGAAADLVQYNLDTVHGHTALALMFDAVAEKSEAAALWERALRMLQCHRQGVPTLLGLAVVAVAQKKQAQGNRDVARLPQGLQKYVLKLREWASQRQSGSSR

>9

MSLPQLYENAKALKARCPSHPTSEYATDFKFSRFYAEPLATSGPALLLDDMSTAGRYRSIWVSLSTALYYDAKRDFRDLGRADINVYPLHKLPYGPLDPADYGDGVMFCTYQSLIAQNKHSERRIDQLVTWAAASASGDPARFEGCICFDEAHRAKNLALDRGGKGTKTGEMVLGIQKQLPQARVLYVSATAAAEVKDLGYMSRLGLWGRGTPFPDFSTFAAKIERAGVGAMELLAMDMKARGMFVSRMLAFTGCSFEVRECTLDQQNERLYDEAVELWDDMLTALEKCAKANEAAERENRAGRELDSEPAVPEAVQIRDKLLRRLDAIDMPSNALDLVIGHFGET

*Aureococcus anophaegefferens*:

>1

MEEEIEQVEFQTYVPHKLLKSMPDAKEHPDKVVENATLAAVESPDIDVENAEIKISKKVVEQGLLSGLQLETVVYAAMRHEKTLANGSRAGFALWDGAGMGKGRQLAGIIHNNWRCGRKKHVWVSISADLVEDARRDLKDVNEPKIEVRALNDWKASKKPTLKEGVLFVTYSLLISKDSDGKRRLDQLAKWCGKDFDGCLFFDEAHRAKNLYPTAGVNGQLPKRPTKTGQAVKDIQARLPRARVVYASATAATEPNHVGYCCRLGLWRREAGDDDDELDDAPTGQTAPFESFLDFLAAVRGANATAMMEATAMALKKEGALLSRSLSFADCSFELIDGVMEASSEQIFDNAAELWSELHRAITFLSETEQLRSDFKGVDSDSDDDMEGPTKEAKLWSAFWGAHQRFFKELCVATKVPAVVQQTKLALAAGKCVVIGLLGTGEARATAARAEFGDEFDDFVSAPLATLLGFIKRAFSLPAKMEGTLERRAPLGKSAAKRADRAADNVELGKTRVRLVCGGRTRVGVLTEWDAPFYTVEFLKGSPRFDKITKSRAAGSIEYDDDYSGDDGSGDEKPKKRAPPEGEPAAAKKTRLADGSAKAPRAKPAPRKAKGLLDDSSSDDDAPAPRKRPPAREKRGLLDDSDDDDVEAPPTRATRAKPVVSYAEPSLKKKLRRPKAEDASSASDRASATSSGRASPASSEASPAPEAVVVDDDDDAMSDGTEGYVTSADFVKPEPRAAPAKPTRAAPAAAAAAPAAAERGSAALPLDISSDDDDDAAPAPFRGPCSAEKPLDLTGDDDVAPHGGGWTCREAACGAQNDALRNFCSRCSAYKPSDPTEVVEVYEPAAWQRRSYQRKPQKSDLQAKAAKAKEGKPALAKAPAKVGALDNVDFELNLELRRTFEDAAHALRLPNSVLDRLIHELGGSDKVAEMTGRQGGLVRDGDKVRHEVRRVSEGARNKANLYEREQFNSGKKLVAIISEAASAGISLHADKRVPARFRNTRRRVHVTMELPWSADKAIQQLGRSHRSNQASAPEYKFLISSVGGERRFASAVAKRLMTLGAVTQGDRRATVGAAGLGMTQFNYDSPEGSRALLDMFRVLAGVQTPPFALPAVPQREAAREKDRIMDLIDGGVKEQKDGGIHVSLSFLEDDDGGEQDGAEEAEEEEDDDVVMTAAPAQLAHKRHDCALHPFGKLGAYRRACARCYCAVCDVPVAECRKWSDHCRATDKGPDADLWKKKRDALRDIDLPVAARFWFDRLGMDVDGWAKLDKPGARTSHVARFLNRLLGMPLGQQNLLFELFARLTEDVIEKRREEGALDEGIRTLKGASVKVAARADVGGGVERVEVHVDRGCDFEAARAELDEAASFADALKRREAAQRKDWGVVNDLKSAISRDAFRNSFYLARDKQAKLKREPAFKQVLLAVRVASYSPGDTERLRVVRPATGARQMTYKELKRNYAPISTADAEKHWRAEHRDGDRGCIHKNCAIVGCDFGKRTQSVHAITGDSLLDVMCRAVEAKKRAGVEGRLDVVRVCESGAPAEAASTVAIKLPRGSHEVIIDQLQAEADDAPEDDDDEDAVFG

>2

MEDREKQRKEREEQRKRAEAEAEKKKLAAAEAEADAGEGGEDATDDVQFSDYQPLKLRGGLAHPDPVVENASLSAVRPPDLGGDVLANLDASIIAQGKLSALQLESVAYARQRFDMRLESGARCGFFIGDGAGMGKGRQLAGIIAQHWNEGVERHLWVSVSNDLKFDAERDLRDLGCAHIPVIPLNKCDYGTLKEKKGVIFCTYSALIAARKGKKPQRRLDQILAWCGRASFGGCLLFDESHKAKNLFGGSGGGKPTKVGQAVMELQTSLPDARVVYCSATGASEPRHLGYMDRLGLWGGDACPFASFRSFLTEVDKRGVGMMELVAMHLKQRGALVCRALSFKNCTFELMDGIMNVHVEKVYDRAAELWDVLKRVMEEMLRAGELADPFAVSEEDLAEGKKARRGHGMLWRYFWGAHQRFFKDLCVASKVPAVIDVAKSALADGKCVVIGLQSTGEARTKDAIEEYGDVMDDFVSAPRATLDRFVRKVFACPD

>3

MEDVDFSNPTAVDDDDDDDDEEAGGQGDEAQTVEEDVRYEAYTPRVVKIKGMKRHPSDAVENGTLATVAPPAVTYALKLAEERPKVVKEGRLSDLQLEFVTYACQRHDEDLASGARAGFFLGDGAGMGKGRQLAGLILENVLQGRKKHIWVSTNIDLHEDAARDLGDVGVDEVSVFQLPARQKTVIKQDAGVLFCTYSGLSKGCSAKSIERGTPARLDQIVAWCGGEDFDGCLLFDEAHRAKNLISEVPGTETAAGKAVFEIQKLLPRARVVYCSATAVSEPRNYGYMTRLGLWGPRSPFPTCEADGDENSMHAVKNFIKLAELRGVGAMEMCALHLKREGALLCRTLSYTGAAFDVVEATLSPEQIAQYDKAAQLWQILHTRCESKLAKMATIAEHSEDEDGSLARAYREARQSYHSSLWGGHQRFFRSLITGFKVPTLVKLARAALDDGKCVVIGLQSTGEA

>4

MQNMHEKKCFCDGEKLVAIISEAASAGISLQADRRIPNQRRRVHLTLELPWSADKAIQQLGRSHRSNQSSAPEYKFLISSVGGEKRFACAVARRLESLGALTQGDRRATVGAKGLGLQSFNFDTKWGRQALATLMRVVVRAANSPFTLPTLPKDEKDELMQAFSKDGTIFSGGGADPELSVGIDASLDTEKKASVGVFLNRLLGLTLVRQNLIFDYFSKLVDHHVRVARREGKYDEAIAEVKGHRVEITDETVLQAETSTRGELVHLNVEVDRGIPWDEALAVLASATEDAAHEKAAKEAQAQHRGRNAFTDMRSLRGRAEVAGFYRSNIPNPATKKHYVALAIVGKTYTAGDTSGTIRVYRPNTGISQMQKVDLLQRYGKIDAADVKKEWKALYDNGDKHCSHGPKCKHGAKCQVGKRTQQKHVLQGSLLVSMRKAEAIIQKRTGNAIRLGVCRVVETDDAGKSVLGIDLPTCYCQNVMDELQEEDM

>5

MTGRSGRVVRGPSGKFKYEARGGDSGDHSLNVAERKAFMDGRKHVAIISDAASTGVSLHAARGSPAAGRRRVHITLELAWSADKSIQQLGRSHRANQESAPVYKLLTTNLGGEARFASAVAKRLASLGALTKGDRRAASGQDLSDFDLDTRHGRAAL

*Thalassiosira pseudonana :*

>1

MSAAAAVAAAAASSTEVAKPAATTAAAGANKDKEDGVNAEQEITYTKYKPAKLKFGMDHPGEEVPTSVSCNHHQTQLTMQQIDPVVENSTLSAVEPPDIKYNLAMPSSIIAKGKLSNLQLEAIVYGCQRHDTDLPTEKKSRGKENDEPSDDITEDAMNVKGEGKTEGEEKGKDRTPIRAGFLLGDGAGMGKGRTLAGFAVENIARGRKRHVWISVSSDLYEDAKRDLSDLGLTDYAENNCYNLGKLPYGNLVDPPTSANGSKKNNGGKKGGKKTASKKVSGDYEEGVMFVTYSTLIGKSNIGYTRLEQLIDWCGGEEFDGLIMLDECHKAKSVDLDENGNAKRAGKNSACSQIAAKVVELQNALPRARVVYCSATSVSEPKNLGFMSRLGLWGPGTEHPTGFNAFLQGIKRLGTGAMELHAMHLKASGALVARTLSYEDCEFALEEGVTDPKIHDVYNASTELWTDLYHQLADRCAKLKSKEGMQERIDNILGADNMSLNEELREHISLHEDSDSEGSDDDDEGIAEQRNLRRKFRNRKPGHLKGLFWGAHQRFFRSLCIASKVDKAIEFAKEAIEDGHCCVIGLQTTGEARAKDAAKAAGFDKDDGGDFDSFVSAPNEDLKRIIMQMFPLPPKPKGVIAPEFLNPLKKDEIIWGDGAESTEGDEERPFRAARHKHFDYAEGVDKEGNIIEKSKTSISGKKRNKALKRARKKKSRRSSTSTSATTVDPAADDSDMSRELGLRSDDSASDYSLSSDSESESEYVEKIKSTRRIPWNEITVGKDQTTMTRAERIDYQRKILYKKEVERVQKWLATVDDLHLPPNPLDRLLNELGGSEKVAELTGRKTRQVKRYDPMEDKMMVVFEKRRADDGPVDKINIEEKNNFQNGDKLIAILSEAASTGISLHADRRVKNQRRRVHRQFCVSYAVFLLASNTVTLELPWSADKAIQQLGRTHRSNQVTGPIYKFLISEVGGEARFAAAVARRLALLGALTQGDRRATGSANSLGLGNFDMDNDYGKRALRKMLDNIFNNSATSIVDGSNEHYSNTIEVIDKHLTIKLGTDRDIDLEEAIRPSEGDSKADQTYDTMMYNLLLGPLKAVAEDRSRAVRENRTIAGYVTSLQDGTETIATVKPKIDAEIKRAKDAGLNFNNICGLWLYDVGIFSDMWEQGGIKGSFGVPKFLNRCLGLRLSKQAALTKYFLKSLEAEIKEAKAAGLYDTGIKVSDVIQPKTIQGNSIEFVGKPRVFCFRGLAAKEEKVYLYTVAIDHGVHSDDALTMYNKMNEQAPDPNSRSPITTGFYVNLPPWWPNKGRTFLSKDMVHKMINEKKVWKGGYSVSQAIDIWSKEFSKADRPVSETYQFSCTGRHDEAHIFSGRIVPLLNKMLSCAEVSATPEGIHGRPFEIVRVETTKATYANTKQEEAESNDKMQIETHFTYSCVDVNGEDAVGKGIARPLDRKRKQCIIRGKITKYLSDEEMFCGHYSDGTRLKLDEEQARGGRVAFEKECKRLVGAGIPQEDASNMSCRTVGASKAMHKVRQPVLEDGEDEDPSSREKIFEEVFNGEIPDTIVGLQFAQHDVNFKNYRVPLWDKVLMSMSEHLVKENVMSALQIAAFEKSENELFKEESSLEVV
